# Novel Sulfonium Reagents for the Modular Synthesis of Spiro[2.3]Hexanes and Heteroatom‐Containing Analogues: Synthesis, Application, and Evaluation as Bioisosteres

**DOI:** 10.1002/anie.202521633

**Published:** 2025-12-09

**Authors:** Philipp Natho, Annarita Vicenti, Fabrizio Mastrolorito, Francesca De Franco, Lee Walsh‐Benn, Marco Colella, Ernesto Mesto, Emanuela Schingaro, Orazio Nicolotti, Antimo Gioiello, Renzo Luisi

**Affiliations:** ^1^ Department of Pharmacy‐Drug Sciences University of Bari “A. Moro” Via E. Orabona 4 Bari 70125 Italy; ^2^ Tes Pharma S.r.l. Via Giovine Italia, 1, Solomeo Corciano (PG) 06073 Italy; ^3^ CAS – A division of the American Chemical Society ACS International, Ltd. 2540 Olentangy River Road Columbus Ohio 43202 USA; ^4^ Department of Earth and Geoenvironmental Sciences University of Bari “A. Moro” Via E. Orabona 4 Bari 70125 Italy; ^5^ Department of Pharmaceutical Sciences University of Perugia Via del Liceo, 1 Perugia 06123 Italy

**Keywords:** Azetidines, Cyclobutanes, Heterocycles, Oxetanes, Sulfonium salts, Spiro[2.3]hexanes, Synthetic methodologies

## Abstract

Molecular scaffolds with a high fraction of *sp^3^*‐hybridized centers have attracted considerable attention in medicinal chemistry as bioisosteres for a wide range of aromatic and nonstrained heterocycles. In particular, strained spiro‐heterocycles have garnered popularity for this purpose, although access to spiro[2.3]hexane analogues is underrepresented. We herein report modular access to nine different spiro[2.3]hexane analogues, including previously underdeveloped 5‐oxa‐1‐azaspiro[2.3]hexane and 1,5‐diazaspiro[2.3]hexane motifs. Our synthetic approach leverages novel cyclobutane‐, oxetane‐, and azetidine‐substituted sulfonium salts, which can undergo Johnson–Corey–Chaykovsky type reactions with alkenes, carbonyls and imines to provide access to the desired spiro[2.3]hexanes. Here, we also report the first comprehensive computational and predictive in silico evaluation of their bioisosteric potential, with validation provided by in vitro experiments.

## Graphical abstract

Strained spirocyclic spiro[2.3]hexane and its heteroatom‐containing derivatives, including previously underrepresented 5‐oxa‐1‐azaspiro[2.3]hexanes and 1,5‐diazaspiro[2.3]hexanes, were synthesized via a modular approach of insertion of cyclobutane‐, oxetane‐, and azetidine‐containing sulfonium reagents into alkenes, carbonyls and imines. A combined in silico unsupervised learning and predictive analytics approach was used to investigate their potential as bioisosteres and our hypothesis was validated through in vitro studies.

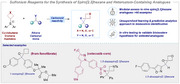

## Introduction

Spurred by breakthrough drug discovery concepts such as *escape from flatland*,^[^ ^1^, ^2^, ^3^ ^]^ or *conformational restriction*,^[^ ^4^ ^]^ the medicinal chemistry community continues to pursue the development of strained *sp^3^*‐rich (and ideally patentable) chemical space to replace aromatic moieties or nonstrained saturated heterocycles.^[^ ^5^, ^6^, ^7^, ^8^, ^9^ ^]^ Incorporation of a higher fraction of *sp^3^*‐atoms can lead to lower off‐target binding promiscuity, improved solubility, and in turn, increase the probability of lead compounds successfully advancing into clinical trials.^[^ ^1^, ^2^, ^10^ ^]^ To address this need, spirocyclic compounds have garnered particular popularity in drug discovery, as their structural features, characterized by a high fraction of sp^3^‐hybridized centers, three‐dimensionality, and an advantageous balance between conformational rigidity and flexibility, are well‐aligned with current strategies in rational drug design.^[^ ^11^, ^12^ ^]^

**Figure 1:**
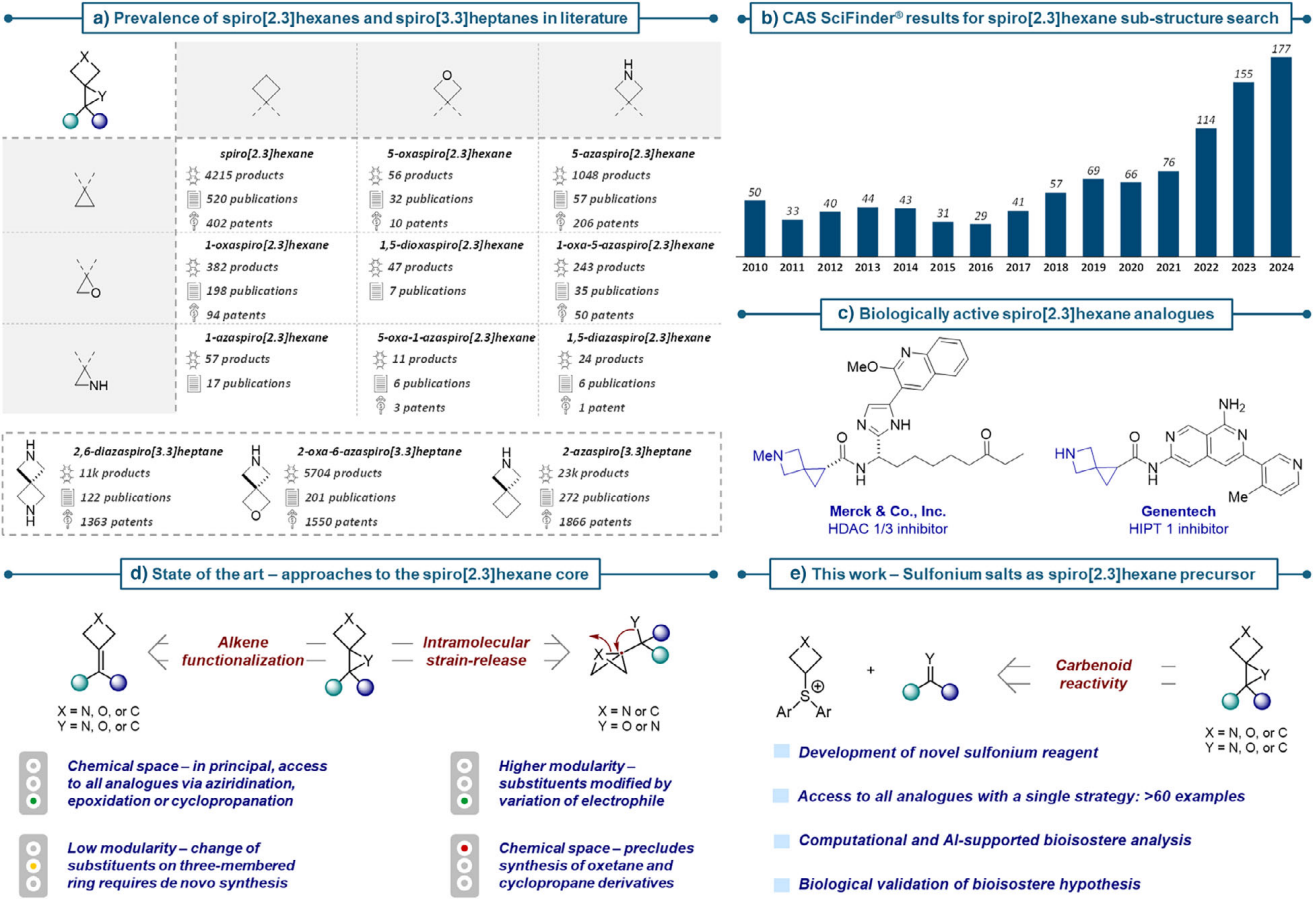
a) Prevalence of spiro[2.3]hexanes and spiro[3.3]heptanes (incl. heteroatom‐containing analogues) in academic and patent literature. Data obtained from CAS SciFinder^®^ through sub‐structure search and further filtering for “available as product/preparation available”. Reviews were excluded. Accessed: 19/06/2025. b) Number of reports (incl. reviews) per year relating to spiro[2.3]hexane and its heteroatom‐containing analogues. Data obtained from CAS SciFinder^®^ through sub‐structure search. Accessed: 19/06/2025. c) Biologically active lead compounds bearing heteroatom‐containing spiro[2.3]hexanes. d) State of the art approaches for the synthesis of spiro[2.3]hexanes. e) This work – sulfonium salts as precursors to spiro[2.3]hexane and its heteroatom‐containing analogues.

For a large part, heteroatom‐containing spiro[3.3]heptanes have been explored as bioisosteres of common motifs in drug discovery (e.g., 2,6‐diazaspiro[3.3]heptane as a piperazine bioisostere, or 2‐oxa‐6‐azaspiro[3.3]heptane as a morpholine analogue) and validated by incorporation into lead compounds successfully advanced in clinical trials.^[^ ^11^, ^13^, ^14^, ^15^, ^16^, ^17^, ^18^, ^19^, ^20^ ^]^ This strong interest is reflected not only by the number of analogues synthetically available for some of these spiro[3.3]heptane derivatives (up to 23k compounds), but also by the high amount of patent literature (>1300 patents per scaffold) and academic literature (>120 publications per moiety) describing these selected cores (Figure 1a).^[^ ^21^ ^]^

Although they have attracted increased academic attention, especially over the last five years, as reflected by the rising number of related publications (Figure 1b), their ring‐contracted lower homologues containing a three‐membered ring—spiro[2.3]hexanes—remain relatively underexplored.^[^ ^21^ ^]^ This is evident from the limited number of products reported in academic publications or patents (Figure 1a).^[^ ^21^ ^]^ For example, only eleven 5‐oxa‐1‐azaspiro[2.3]hexane analogues are described in six publications and three patents, or 47 1,5‐dioxaspiro[2.3]hexane analogues in seven academic publications, with the motif being completely absent from the patent literature so far, reflecting a lack of systematic and general reports towards these scaffolds. This is surprising, as spiro[2.3]hexanes exhibit similar physicochemical properties as their larger homologues, yet occupy a different 3D‐space and less crowded intellectual space, representing an unprecedented opportunity for medicinal chemists and drug discovery programs.^[^ ^22^ ^]^ Their potential in biologically active lead compounds has been validated by an HDAC1/3 inhibitor^[^ ^23^ ^]^ and a HIPT 1 inhibitor^[^ ^24^ ^]^ incorporating the 5‐azaspiro[2.3]hexane motif (Figure 1c), although no systematic evaluation of their bioisosteric potential of common heterocycles in drug discovery has hitherto been reported.

Nonetheless, the adoption of these strained spirocycles in drug discovery programs remains marginal. As the molecular architecture of drugs is inextricably linked to advancements in chemical synthesis,^[^ ^25^, ^26^, ^27^, ^28^ ^]^ this incongruity may arise from synthetic difficulties in efficiently incorporating such motifs (cf. Figure 1a) and the lack of data on their chemical reactivity and stability. To address this, we first examined existing strategies to identify important design aspects that currently hinder wider adoption. From a synthetic standpoint, either the construction of the four‐membered ring or of the three‐membered ring are conceivable.^[^ ^11^, ^12^ ^]^ Both approaches are known, with the latter strategy being more popular. Indeed, for a large part heteroatom‐containing spiro[2.3]hexane derivatives are accessed by construction of the three‐membered ring moiety by epoxidation, aziridination or cyclopropanation of the four‐membered ring bearing an exocyclic double bond (Figure 1d);^[^ ^29^ ^]^ and most recently an enantioselective version has been reported.^[^ ^30^ ^]^ Although well‐studied, and allowing access to all nine analogues, these transformations typically demand harsh reaction conditions limiting the inclusion of sensitive functional groups. In addition, any alteration of the substituents on the three‐membered ring requires the de novo synthesis of the four‐membered ring‐fragment, reducing efficiency in large library synthesis. In recent publications the Aggarwal^[^ ^31^, ^32^ ^]^ and Saha^[^ ^33^, ^34^ ^]^ groups have demonstrated an alternative approach by intramolecular strain release‐driven epoxidation/aziridination of “spring‐loaded” bridgehead bonds of bicyclo[1.1.0]butanes or azabicyclo[1.1.0]butanes (Figure 1d). Whereas this strategy addresses the aspect of modularity, it requires the in situ preparation of highly reactive ABB‐Li or BCB‐Li intermediates, although the preparation can be controlled under flow conditions.^[^ ^20^, ^35^, ^36^ ^]^ More importantly, however, this strategy precludes the synthesis of cyclopropane‐ and oxetane‐containing spiro[2.3]hexanes (i.e., five out of nine analogues). With regards to our own involvement in this field, we have reported an enantioselective synthesis of 1‐oxaspiro[2.3]hexanes,^[^ ^37^ ^]^ and most recently a protocol for the synthesis of 1,5‐dioxaspiro[2.3]hexanes through a regioselective radical C–H functionalization of 3‐iodooxetane which, however, could not be expanded to other spiro[2.3]hexane derivatives.^[^ ^38^ ^]^

For our ongoing research into the synthesis and biological evaluation of spiro heterocycles, we required a more general, systematic approach that would not only be complementary but combine advantages of existing strategies: i) increased modularity by facile alteration of a wide range of substituents, ii) use of stable and scalable precursors, and iii) access to all nine analogues from a single, mild, and general synthetic strategy, in line with the community's move towards the development of protocols for the mild construction of small rings.^[^ ^39^, ^40^ ^]^ We envisioned that such a strategy would be of value to the wider chemistry community. Drawing from our expertise, and recent renaissance of such approaches, we hypothesized that a carbenoid strategy^[^ ^41^, ^42^, ^43^, ^44^, ^45^ ^]^ could offer the solution to combine our objectives and thus postulated the use of sulfur ylide chemistry (Figure 1e).^[^ ^46^, ^47^, ^48^, ^49^, ^50^, ^51^ ^]^ We proposed the synthesis of novel cyclobutyl‐, oxetanyl‐, or azetidinyl‐substituted sulfonium salts that could undergo Johnson–Corey–Chaykovsky reactivity^[^ ^52^, ^53^ ^]^ with carbonyls, imines, and alkenes upon ylid formation by deprotonation, to provide the desired spirocycles with flexibility in the modification of the substituents on the three‐membered ring. Realization of this proposed concept would rapidly expand the limited palette of spiro[2.3]hexanes available by addressing the aforementioned design objectives. Herein, we report our studies on the development of these proposed reagents, as well as their broad applicability across a wide array of π‐electrophiles. In addition, we present for the first‐time preliminary results on their computationally and AI‐supported evaluation as bioisosteres, including validation through in vitro screening.

## Results and Discussion

### Synthesis of Sulfonium Salts

**Scheme 1:**
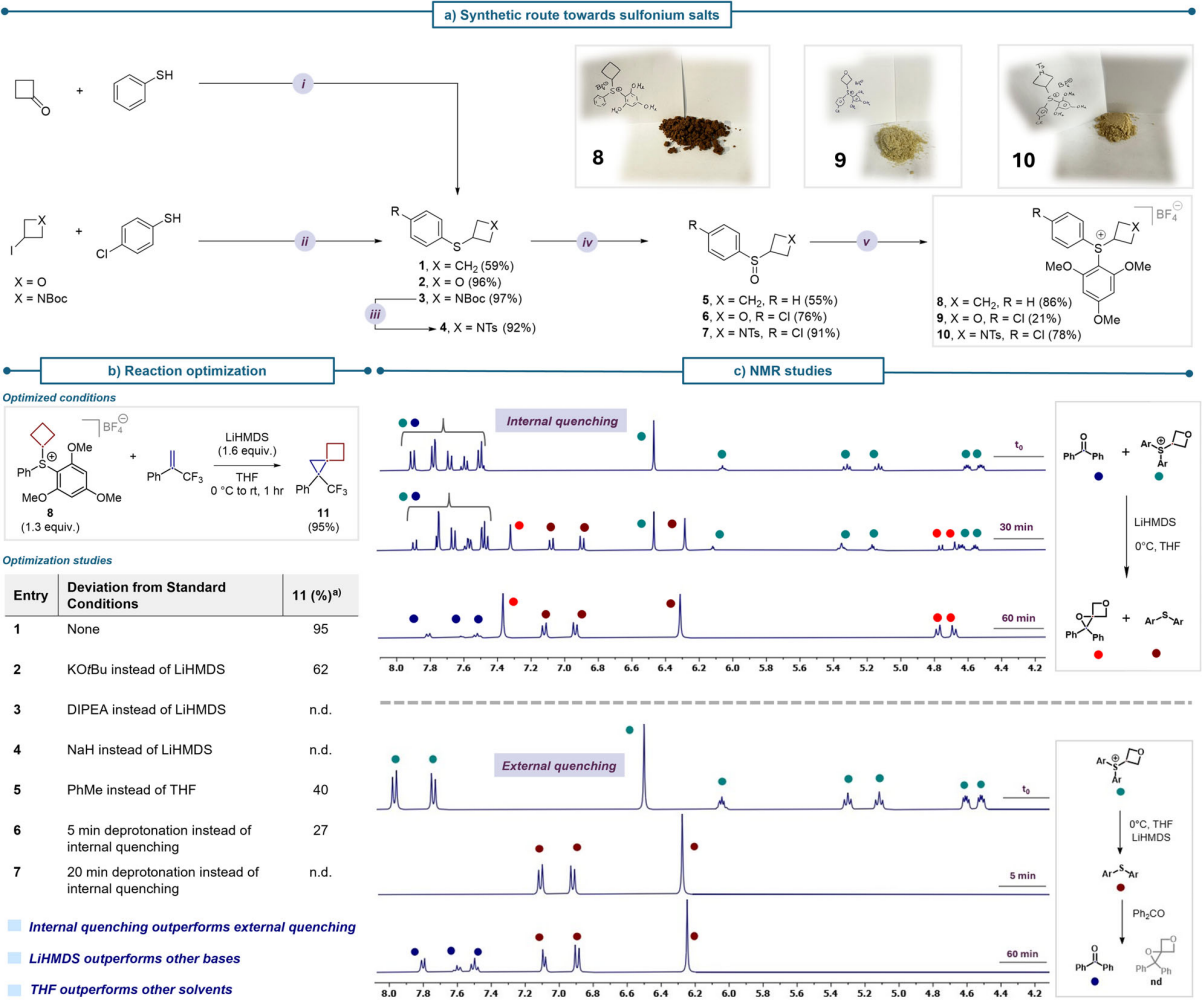
a) Synthesis of sulfonium salts **8**–**10**: i) Et_3_SiH, TFA, CH_2_Cl_2_, rt, 10 h; ii) K_2_CO_3_, DMF, 50 °C, 6 h; iii) TFA, CH_2_Cl_2_, rt, 1 h, then DMAP, Et_3_N, TsCl, rt, 12 h; iv) NBS, MeOH, H_2_O, 0 °C to rt, 2 h; v) 1,3,5‐Trimethoxybenzene, Tf_2_O, Et_2_O, 0 °C, 2 h, then 1 M NaBF_4(aq)_, CH_2_Cl_2_, rt, 1 h. b) Reaction optimization – optimized conditions and optimization studies. ^a)^ Yields were determined by quantitative ^1^H NMR analysis. c) ^1^H‐NMR study on ylid stability and reaction progression in d_8_‐THF. For improved visualization, only selected peaks are shown. Full spectra are available in the Supporting Information.

At the outset of the study, we investigated the general strategy for the synthesis of the required sulfonium salts **8**–**10**. The optimized route is shown in Scheme 1a. The synthesis begins with the preparation of sulfide intermediates **1**–**3**, accessible by triethylsilane‐mediated reductive thiolation from cyclobutanone for the cyclobutyl analogue **1** in 59% yield^[^ ^54^ ^]^ or a base‐induced nucleophilic substitution for the oxetane‐ and azetidine analogues **2** and **3** in almost quantitative yields in both cases. A change of the azetidine protecting group from Boc‐group to the tosyl‐group was performed, which was found essential at this stage to ensure compatibility with subsequent chemistry (see Supporting Information for complete exploration of the synthetic route towards the azetidine analogue).

Selective oxidation of the sulfide to the sulfoxide by *N*‐bromosuccinimide provided the desired intermediates **5**–**7** in 55%–91% yield. Last, an interrupted Pummerer reaction with 1,3,5‐trimethoxybenzene and trifluoromethanesulfonic anhydride provided the desired sulfonium salts **8**–**10**, after anion exchange with lithium tetrafluoroborate. Anion exchange from triflate to tetrafluoroborate was found necessary to obtain the desired salts as air‐ and moisture‐stable free‐flowing solids, instead of hygroscopic waxy oils. To our delight, all sulfonium salts (**8**–**10**) were obtained in high purity and high yields even on a gram scale. It is worth noting that more direct approaches of forming the sulfonium salts **8**–**10**, for example by replacement of a suitable leaving group on the four‐membered ring with diphenyl sulfide under thermal conditions, or Lewis‐ or Brønsted‐acid catalysis failed (see Supporting Information).

### Optimization

With the three sulfonium salts **8** – **10** in hand, we tested the proof of concept and chose 3,3,3‐trifluoro‐2‐phenylpropene as an electrophilic model substrate in combination with the cyclobutyl sulfonium salt **8** (Scheme 1b). Upon optimization of the reaction conditions (see Supporting Information), we found that treatment of a mixture of the electrophile (1 equiv.) and a slight excess of the sulfonium salt (1.3 equiv.) in THF at 0 °C with LiHMDS (1.6 equiv.) proved optimal to afford the desired spiro[2.3]hexane **11** in 95% yield within 1 h.^[^ ^55^ ^]^ Replacement of the base with potassium *tert*‐butoxide reduced the efficiency of the process to 62% yield, whereas the use of DIPEA or sodium hydride, often employed in similar transformations,^[^ ^51^, ^56^, ^57^, ^58^ ^]^ returned only starting material. Conducting the reaction in toluene also had unfavourable effects on the reaction outcome, and reduced product formation to 40% yield. Last, we tested an external quenching protocol – consisting of prior deprotonation of the sulfonium salt **8** before addition of the electrophile. To our surprise, this had detrimental effects on the reaction, with a 5 min deprotonation time reducing the yield of desired product to 27%, and a 20 min deprotonation time leading to cessation of product formation. Intrigued by this result, we undertook ^1^H‐NMR studies to rationalize this observation (Scheme 1c). The reaction between oxetane sulfonium salt **9** and benzophenone was selected for this purpose to facilitate monitoring of the reaction. Under internal quenching conditions, product formation was cleanly observed. Indeed, the progress of product formation and consumption of the starting materials could be monitored over time. Under external quenching conditions, consisting of deprotonation of the sulfonium salt for 5 min, followed by addition of benzophenone, no product formation was observed. Curiously, deprotonation of sulfonium salt **9** led to rapid ylide decomposition, affording the bis‐aryl sulfide as the only detectable product, rationalizing the requirement for an internal quenching protocol to trap the unstable putative ylid.

### Scope Evaluation

**Scheme 2:**
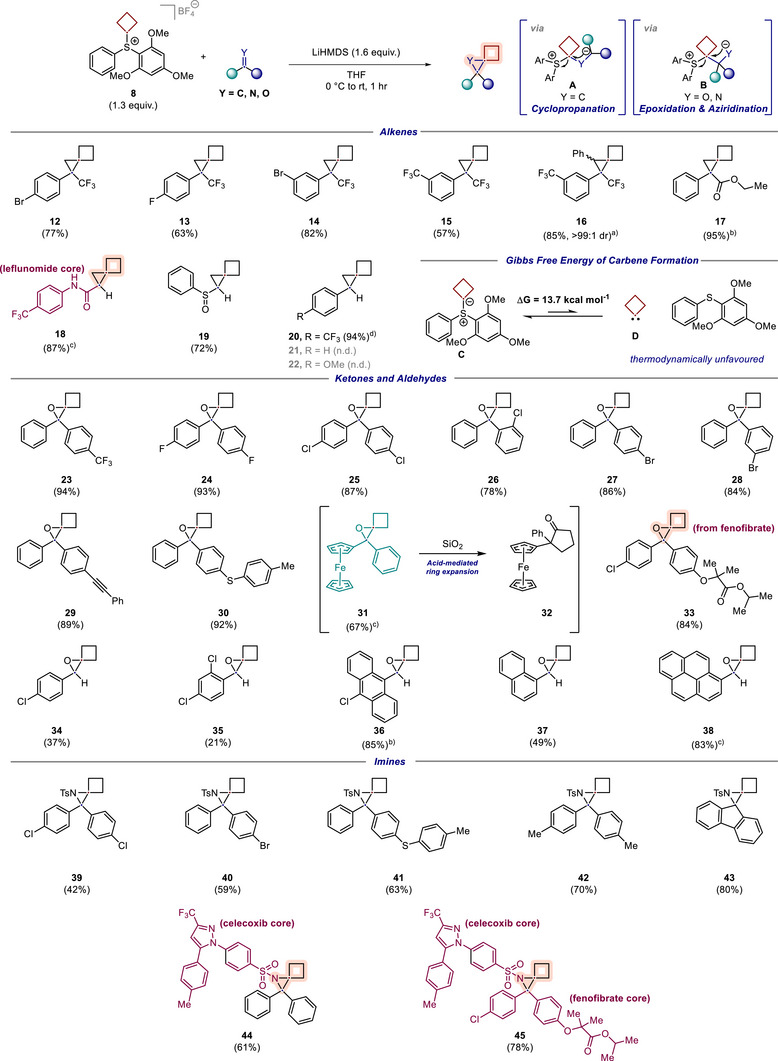
Scope exploration for cyclobutyl sulfonium salt **8**. Yields are quantitative ^1^H‐NMR yields. ^a)^ Using a modified procedure with electrophile (1 equiv.), sulfonium salt **8** (4.0 equiv.), LiHMDS (4.7 equiv.), stir overnight; ^b)^ Using a modified procedure with electrophile (1 equiv.), sulfonium salt **8** (2.6 equiv.), LiHMDS (3.2 equiv.), stir overnight. ^c)^ Using a modified procedure with electrophile (1 equiv.), sulfonium salt **8** (1.9 equiv.), LiHMDS (3.4 equiv.). ^d)^ Using a modified procedure with electrophile (1 equiv), sulfonium salt **8** (3.8 equiv.), LiHMDS (4.8 equiv).

With a reliable protocol for the synthesis of spiro[2.3]hexanes from the sulfonium salt in hand, we sought to evaluate the scope of the present transformation. Following our optimization studies, we continued our exploration with cyclobutyl‐analogue **8** and alkenes to provide spiro[2.3]hexanes (Scheme 2). We found that in addition to 3,3,3‐trifluoro‐2‐phenylpropene, also its substituted derivatives afforded cyclopropanes **12**–**15** in up to 85% yield. Of particular note is cyclopropane **16**, which was obtained as a single diastereomer, derived from the diastereoselective addition onto a trisubstituted alkene. To our delight, high functional‐group compatibility was observed with other alkenes bearing electron‐withdrawing substituents, such as acrylates, acrylamides or styrenes, being well tolerated. For example, cyclopropanes **17** and **18** were obtained in up to 95% from the reactions with an acrylic ester and an unprotected acrylamide bearing the leflunomide core, respectively, whereas phenyl vinyl sulfoxide provided the cyclopropane **19** in 72% yield. Last, the reaction with 4‐trifluoromethylstyrene delivered the desired spiro[2.3]hexane **20** in 94% yield. This is particularly surprising as styrene derivatives are not usually prone to undergo Johnson‐Corey‐Chaykovsky reactivity with sulfonium ylids. Indeed, a test reaction with trimethylsulfonium iodide failed to deliver the corresponding cyclopropane from this substrate under our standard conditions (see Supporting Information). In contrast, more electron‐rich styrene derivatives (styrene and 4‐methoxystyrene) failed to deliver the corresponding spiro[2.3]hexane derivatives **21** and **22**. Previously reported dihalomethylsulfonium salts are known carbene precursors and, in contrast to our sulfonium salt, react with more electron‐rich styrene derivatives.^[^ ^51^, ^55^, ^56^, ^58^ ^]^ The difference in reactivity of our sulfonium salt suggested to us that the reaction indeed most likely does not proceed *via* carbene insertion, but instead by nucleophilic attack of the ylid to the electrophile, followed by intramolecular ring closure (Scheme 2, intermediates **A** and **B**). To further probe this, we conducted density functional theory (DFT) calculations on the α‐elimination of the ylid **C** to the carbene **D** and the sulfide by‐product, using the M06‐2X level of theory (Def2‐SVP basis set) and an implicit solvent (THF) model (Scheme 2, see Supporting Information for further information). These calculations showed that α‐elimination on the ylid **C** to deliver the cyclobutyl carbene **D** is thermodynamically unfavorable (*ΔG* = 13.7 kcal mol^−1^), which is in agreement with our experimental results. The experimental evidence in conjunction with the computational calculations thus suggests that a carbene pathway is unlikely, and that the reaction likely involves a nucleophilic carbenoid and proceeds *via* intermediates **A** or **B** (Scheme 2).^[^ ^43^, ^44^ ^]^

We then turned our attention to other π‐bond electrophiles, and subsequently selected to investigate carbonyls to provide 1‐oxaspiro[2.3]hexanes. We were pleased to find that various aryl ketones bearing electron‐donating and electron‐withdrawing substituents gave the corresponding epoxides **23** – **30** in synthetically useful yields (78%– 94%). For example, epoxide **24** derived from 4,4′‐diflluorobenzophenone was formed in 93% yield. Ketones bearing halogens in ortho‐, meta‐, or para‐positions on the aryl ring were competent reactants (**26** – **28**), as were benzophenone derivatives bearing alkyne and sulfide‐substituents to afford epoxides **29** and **30** in 89% and 92% yield, respectively. Last, we tested aromatic ketones bearing strongly electron‐donating substituents. As such, benzoyl ferrocene underwent the reaction smoothly to afford epoxide **31** in 67% yield. It is worth noting that, in line with observations made previously by us and others,^[^ ^32^, ^38^ ^]^ this epoxide bearing a strongly electron‐donating substituent underwent an acid‐mediated rearrangement to cyclopentanone **32** during purification by flash column chromatography, which could also not be suppressed through neutralization of the silica gel. To our delight, fenofibrate, a drug for the treatment of abnormal blood lipid levels, was successfully transformed into spiro‐epoxide **33** in 84% yield.

Aromatic aldehydes were also effective in our protocol. 4‐Chlorobenzaldehyde and 2,4‐dichlorobenzaldehyde were competent reactants, affording epoxides **34** and **35** in moderate yields (37% and 21%, respectively). The use of other aromatic aldehydes, including 10‐chloro‐9‐anthraldehyde, 1‐naphthaldehyde and pyrene‐1‐aldehyde, provided the epoxides **36**–**38** in up to 85% yield.

To further highlight the versatility of the method, we extended the protocol beyond carbonyls and alkenes to include imines as electrophiles to provide a novel avenue to 1‐azaspiro[2.3]hexanes. Satisfyingly, various aziridines resulting from *N*‐tosyl imines bearing electron‐withdrawing substituents (**39** and **40**) and electron‐donating substituents (**41** and **42**) were obtained in 42%–70% yield. Notably, also sterically demanding vicinal spiro‐centers could be readily accessible from a fluorenone derivative to deliver 1‐azaspiro[2.3]hexane **43** in 80% yield. As the imine nitrogen offered a useful anchor point for functionalization, this allowed us to extend this methodology to employ a range of pharmaceuticals, and we were delighted to find that this protocol was amenable to such complex settings. For example, the celecoxib core, a COX‐2 inhibitor and NSAID, was successfully functionalized to provide the corresponding spiro‐aziridine **44** in 61% yield. Notably, the spiro[2.3]hexane was also installed into a complex imine containing the cores of both celecoxib and fenofibrate, affording 1‐azaspiro[2.3]hexane **45** in 78% yield – demonstrating how this methodology could link drug‐like molecules on a spiro[2.3]hexane core.

**Scheme 3:**
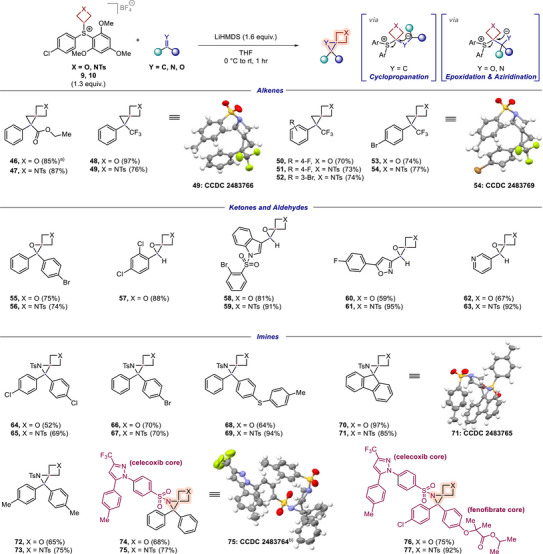
Scope exploration for oxetanyl‐ and azetidinyl sulfonium salts **9** and **10**. Yields are quantitative ^1^H‐NMR yields. ^a)^ Not fully characterized due to isolation with co‐eluting impurities. ^b)^ The compound **75** showed positional disorder on the –CF_3_ group, which was successfully modeled, as well as one molecule of ethyl acetate solvent in the unit cell with an occupancy of 0.88 (not shown). See Supporting Information for further information.

After assessing the scope for the spiro‐cyclobutyl series, we explored the substrate scope of the reaction with respect to the sulfonium salt by preparation of oxetane‐ and azetidine‐bearing spiro[2.3]hexanes from sulfonium salts **9** and **10**, respectively (Scheme 3). These are of particular importance given a) the prevalence and importance of the oxetane and azetidine rings in drug development^[^ ^9^, ^59^, ^60^, ^61^, ^62^, ^63^, ^64^, ^65^ ^]^ and b) the comparative underdevelopment of spiro[2.3]hexane scaffolds bearing these four‐membered rings compared to their cyclobutyl analogues. With a holistic picture of suitable electrophiles from our study on the cyclobutyl analogue in hand, a series of selected electrophiles was tested.

We began this investigation with the synthesis of 5‐oxaspiro[2.3]hexanes and 5‐azaspiro[2.3]hexanes from various alkenes. Comparable to our findings with the cyclobutyl analogue, alkenes bearing electron‐withdrawing substituents (e.g., acrylates or 3,3,3‐trifluoro‐2‐arylpropenes) smoothly underwent the desired transformation, under our standard conditions, providing cyclopropanes **46**–**54** in generally synthetically useful yields (70%–97% yield). Moreover, the structures of 5‐azaspiro[2.3]hexanes **49** and **54**, bearing trifluoromethyl‐ and aryl‐substituents, were confirmed by single crystal X‐ray crystallography.

Next, we focused on the synthesis of 1,5‐dioxaspiro[2.3]hexanes and 1‐oxa‐5‐azaspiro[2.3]hexanes by the treatment of ketones and aldehydes with oxetanyl sulfonium salt **9** and azetidinyl sulfonium salt **10**, respectively. Excitingly, the desired epoxides **55** – **63** were obtained in good to excellent yields with marginally higher yields generally obtained from the azetidine analogue. Notably, pharmaceutically relevant building blocks such as indole (**58** and **59**) and 5‐(4‐fluorophenyl)isoxazole (**60** and **61**) were successfully incorporated with high efficiency, as was pyridine (**62** and **63**).

To complete our objective of accessing all nine different spiro[2.3]hexane analogues (*cf*. Figure 1a), we last focused on the synthesis of 5‐oxa‐1‐azaspiro[2.3]hexanes and 1,5‐diazaspiro[2.3]hexanes by the reaction of both sulfonium salts **9** and **10** with imines. This was a particularly important investigation as the desired products are currently almost absent from the literature (*cf*. Figure 1a). To our delight, various electronically diverse imines could be transformed successfully into the corresponding aziridines **64**–**77** in up to 97% yield. These examples represent some of the first isolated structures of this substituted motif, thus showing the potential of this methodology to expand the currently available chemical space. The structure of 1,5‐diazaspiro[2.3]hexane **71** has been confirmed by single crystal X‐ray crystallography. Last, to showcase its potential in the context of drug‐like molecules, the spirocyclic unit was installed in a motif bearing the biologically relevant celecoxib and fenofibrate cores in 68%–92% yield (**74**–**77**). The structure of celecoxib‐bearing derivative **75** was furthermore confirmed by single crystal X‐ray crystallography.

In line with our objectives defined at the outset, we have thus successfully demonstrated the usefulness of the novel sulfonium reagents for the synthesis of all nine spiro[2.3]hexane cores, including underdeveloped 5‐oxa‐1‐azaspiro[2.3]hexanes and 1,5‐diazaspiro[2.3]hexanes, with a wide substrate scope (> 60 examples). The developed method is practically simple, modular, and efficient with a wide variety of π‐electrophiles bearing diverse functional groups, and additionally enables the late‐stage incorporation of spiro[2.3]hexane motifs into drug‐like structures.

### Unsupervised Learning‐ and AI‐Supported Bioisostere Identification and In Vitro Validation

**Scheme 4:**
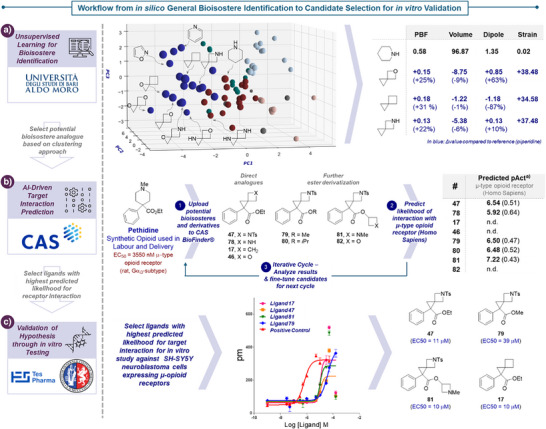
Workflow from in silico bioisostere identification to in vitro validation. a) Unsupervised Learning for Bioisostere Identification: 3D space of Principal Component Analysis (PCA) and clustering (left) and the calculated Plane of Best Fit (PBF), volume, dipole, and strain energy of piperidine are compared with those of 5‐oxaspiro[2.3]hexane, spiro[2.3]hexane, and 5‐azaspiro[2.3]hexane reported as differences in percentage (Δ Value %) (right). Atomic positions optimized by DFT (ωB97X−D3BJ/6–31++G(d,p)) See Supporting Information for full details. b) AI‐Driven Target Interaction Prediction using CAS BioFinder^®^. See Supporting Information for full details. c) Validation of hypothesis through in vitro testing using SH‐SY5Y neuroblastoma cells expressing μ‐opioid receptors. ^a)^ Predicted pAct reported, including confidence score in brackets.

With a streamlined, unified strategy to access all nine spiro[2.3]hexane analogues in hand, we wanted to investigate if these motifs – in analogy to their more popular spiro[3.3]heptane counterparts – could also potentially function as bioisosteres for common heterocycles typically found in medicinal chemistry libraries for drug discovery. Although preliminary evidence exists^[^ ^22^ ^]^ a more systematic study to the best of our knowledge outstanding. Our approach towards bioisostere identification relied on three steps (Scheme 4): a) Clustering approach to identify common heterocycles with similar properties; b) AI‐driven target interaction prediction to narrow down candidate selection for specific biological target, and c) in vitro testing of prioritized ligands with high likelihood of interaction with the chosen target.

Following Leonori's approach for in silico bioisostere identification,^[^ ^66^ ^]^ we elected an unsupervised learning approach in which the (heteroatom‐containing) spiro[2.3]hexane cores were compared to a virtual database of over 70 common heterocycles in drug discovery (see Supporting Information) (Scheme 4a). In general, this three‐step approach consists of i) optimization of structures by DFT calculations, ii) creation of high‐dimensional dataset from relevant global and local molecular descriptors, and iii) visualization and clustering of dataset through dimensionality reduction.

Following this method, first, all structures were optimized using *ab‐initio* DFT calculations (WB97X‐D3BJ, 6–31++G(d,p)) to obtain the corresponding 3D conformation and structural properties. Next, we focused on the selection of a series of one‐dimensional (e.g., molecular weight, number of heteroatoms), two‐dimensional (e.g., drug likeness, logP, topological polar surface) and three‐dimensional (e.g., dipole moment, plane of best fit, asphericity) descriptors computed by using RDKit^[^ ^67^ ^]^ to best allow comparison of physicochemical properties and assess drug‐likeness of the chosen cores. Manual outlier removal of individual descriptors ensured relevant statistical analysis (descriptor removed if log_10_(VIF) ≥ 5).^[^ ^68^ ^]^

Last, visualization and clustering of the high‐dimensional data set was required and achieved through dimensionality reduction by principal component analysis (PCA) and *k*‐Medoids^[^ ^69^ ^]^ clustering. The PCA served to reduce the dimensionality of the chemical space defined by organic cores and to evaluate the relative contribution of each molecular descriptor. Finally, the *k*‐Medoids algorithm was applied, leading to the identification of five distinct clusters (*k* = 5) of chemical analogues by a preliminary statistical analysis based on Silhouette score.

After completion of the clustering, we proceeded to evaluate the cluster containing eight out of nine spiro[2.3]hexane analogues (Cluster 1, see Supporting Information). We were intrigued to find that the same cluster contained two heterocycles commonly found in pharmaceuticals: isoxazole, a heterocycle incorporated in disease‐modifying antirheumatic drug leflunomide for which the spiro[2.3]hexane‐analogue **18** was prepared during the scope‐evaluation, as well as pyridine. In addition, although not in the same cluster, piperidine was placed in spatial proximity to some spiro[2.3]hexane cores. For example, a closer inspection of the 3D PCA space shows that piperidine and 5‐azaspiro[2.3]hexane span average intra‐cluster distances between 3.20 ± 0.76 and 2.28 ± 1.11, respectively. A shorter distance of 1.69 was instead measured between the two scaffolds, a value confirming their physicochemical similarity. Similarly, spiro[2.3]hexane spans an average intracluster distance of 3.59 ± 1.18, while displaying a distance of only 3.18 to piperidine. Compared to piperidine, spiro[2.3]hexane, 5‐azaspiro[2.3]hexane and 5‐oxaspiro[2.3]hexane were characterized by similar molecular volumes, a key determinant in binding site fitting, and higher 3D drug‐likeness indicators (PBF).^[^ ^70^ ^]^ In addition, 5‐azaspiro[2.3]hexane was found to have a comparable dipole moment, while expectedly showing a large increase in strain energy. Given the spatial proximity in the cluster analysis, as well as the similarity in important molecular descriptors, these findings support the hypothesis that in particular 5‐azaspiro[2.3]hexane could be a suitable strained piperidine bioisostere.

Building on this result, we wanted to validate our hypothesis through biological testing. To streamline our experimental in vitro efforts and focus on the most promising candidates, we aimed to prioritize substrates with a high predicted likelihood of target interaction. Given the increasing availability of data linking between drugs and biological events, several integrative systems‐based approaches have emerged to support the prediction of binding potential.^[^ ^71^, ^72^ ^]^ These methods can significantly enhance the selection process for subsequent biological testing. To this end, we employed the recently introduced AI‐enhanced predictive analytics functionality in CAS BioFinder^®^ (Scheme 4b). This solution enables rapid in silico prediction of pharmacological activity and bioactivity by modeling protein–ligand interactions based on data extracted from the scientific literature. As a testing ground, we selected pethidine—a piperidine‐containing, μ‐opioid receptor agonist commonly used during labor and delivery. This choice was driven by two factors: i) structure – its molecular structure and substitution pattern are ideal to allow for the “replacement” of the piperidine ring with spiro[2.3]hexane analogues with our methodology by reaction of the sulfonium salt with the corresponding acrylate; ii) clinical limitations – a limitation of pethidine is that its metabolite (norpethidine) is significantly more toxic than other opioids, demanding the development of equally effective, yet less toxic alternatives.

For the predictive analytics investigation, we followed an iterative approach consisting of predicting receptor interaction for an analogue, analyzing the result, followed by modification of the structure according to the result to identify further candidates. In line with our results from the unsupervised learning process for bioisostere identification, we initially screened pethidine analogues featuring the 5‐azaspiro[2.3]hexane core against μ‐type opioid receptors (Homo Sapiens). The results of these predictions (pAct and confidence scores) are presented in Scheme 4b. To our delight, the 5‐azaspiro[2.3]hexane analogues **47** and **78** were predicted to have a pAct of 6.54 and 5.92 vs. the μ‐opioid receptor, respectively. In addition, we tested the spiro[2.3]hexane‐ and 5‐oxaspiro[2.3]hexane analogues **17** and **46**, which from our clustering approach appeared less promising potential piperidine bioisosteres. Indeed, no predicted activity against the chosen target could be identified. Based on these results, we focused on testing further 5‐azaspiro[2.3]hexane derivatives by focusing on the modulation of the ester side chain. Replacement of the ethyl ester with a methyl ester (**79**) or isopropyl ester (**80**) reduces the predicted pAct slightly between 6.50 and 6.48, respectively. Surprisingly, replacement of the ethyl ester with an azetidine ester (**81**) or oxetane ester (**82**) had very different outcomes – whereas for the azetidine ester **81** a significant increase in pAct against the μ‐opioid receptor was predicted (7.22), no predicted activity against the chosen panel targets could be identified for the oxetane ester (**82**).

To evaluate the strengths and limitations of this predictive analytics tool, we tested in vitro the three ligands with the highest predicted pAct against the μ‐opioid receptor (compounds **47**, **79,** and **81**), as well as spiro[2.3]hexane **17** as a control, since no activity was predicted for this scaffold. In addition to compounds **17** and **47**, which were already available from our scope evaluation, ligands **79** and **81** were prepared using our developed methodology between 75% and 32% yield, respectively.

All compounds were tested using an in vitro binding assay based on label‐free detection technology (EnSpire platform, PerkinElmer). This label‐free system utilizes optical biosensors to monitor molecular interactions in real time, without the need for fluorescent or radioactive labels. In this study, SH‐SY5Y neuroblastoma cells expressing μ‐opioid receptors were seeded and exposed to serial dilutions of the chosen ligands (**17**, **47**, **79**, **81**), ranging from 0.03 to 150 µM. Binding events were detected as changes in the refractive index, recorded in picometers (pm) of refracted light and normalized to baseline values. These shifts reflect alterations in mass distribution near the sensor surface, indicating the presence and strength of ligand‐cell interactions. A known μ‐opioid receptor agonist, DAMGO was used as a positive control to validate the assay. To our delight, the resulting dose–response curves (Scheme 4c) for the tested ligands demonstrated a micromolar binding activity (10 – 39 µM), confirming their ability to interact with the μ‐opioid receptor. This indicates that in particular this 5‐azaspiro[2.3]hexane scaffold might be a suitable bioisostere for 4‐substituted piperidine, as predicted through our in silico studies. Whereas the unsupervised learning approach delivered a general indication for suitable cores, the AI‐driven target prediction (CAS BioFinder) successfully narrowed down the candidate search for in vitro testing. Indeed, candidates predicted to have a target interaction with the μ‐opioid receptor (ligands **47**, **79**, **81**) were found have micromolar binding activity. To our surprise, also ligand **17**, for which despite the spatial proximity of the spiro[2.3]hexane core to piperidine in the cluster analysis, no activity against the μ‐opioid receptor was predicted, showed micromolar binding activity. This demonstrates that such AI‐driven target prediction tools currently provide a useful guide for these workflows, yet have still limitations with cores for which no extensive underlying data is available.

## Conclusion

We developed a simple, general, and functional group‐tolerant strategy towards underdeveloped spiro[2.3]hexane analogues by use of three novel sulfonium salts as reagents. Our synthetic investigation revealed that electron‐deficient alkenes, carbonyls (ketones and aldehydes), as well as imines are suitable partners for the synthesis of spirocyclic cyclopropanes, epoxides, and aziridines, respectively, and we report over 60 examples demonstrating the wide applicability of this process. Furthermore, we showed through in silico investigation, supported by predictive analytics solutions, that particularly 5‐azaspiro[2.3]hexane exhibits structural characteristics rendering it potentially suitable as a piperidine bioisostere. Using pethidine, a piperidine‐containing, μ‐opioid receptor agonist commonly used during labor and delivery, as a model compound, we validated this hypothesis through in vitro testing. We expect that these novel reagents will find further application in other areas of organic chemistry, and that this route to spiro[2.3]hexanes will allow further studies into their application as potential bioisosteres. Investigations into these topics are currently underway in our laboratory.

## Conflict of Interests

The authors declare no conflict of interest.

All code, raw data and additional information required to reproduce the results of the computational studies described in this manuscript (i.e., Gibbs Free Energy of Carbene Formation, and Bioisostere Analysis through Clustering Approach) are available on GitHub at the following URL: https://github.com/f48r1/spirohexane4bioisostere

## Supporting information



Supporting information

Supporting information

## Data Availability

The data that support the findings of this study are available in the Supporting Information of this article.^[^ ^73^ ^]^
