# Supplementary material for: Novel Sulfonium Reagents for the Modular Synthesis of Spiro[2.3]Hexanes and Heteroatom‐Containing Analogues: Synthesis, Application, and Evaluation as Bioisosteres
Source: Angew Chem Int Ed Engl. 2025 Dec 9;65(5):e21633. doi: 10.1002/anie.202521633 (PMC12851011; doi:10.1002/anie.202521633)

Novel Sulfonium Reagents for the Modular Synthesis of Spiro[2.3]hexanes and Heteroatom-Containing Analogues: Synthesis, Application, and Evaluation as Bioisosteres

Supplementary Information

Philipp Natho,[a] Annarita Vicenti,[a] Fabrizio Mastrolorito,[a] Francesca De Franco,[b] Lee Walsh-Benn,[c] Marco Colella,[a] Ernesto Mesto,[d] Emanuela Schingaro,[d] Orazio Nicolotti,[a] Antimo Gioiello,[e] Renzo Luisi[a],*

[a] Dr. P. Natho, A. Vicenti, F. Mastrolorito, Dr. M. Colella, Prof. O. Nicolotti, Prof. R. Luisi
Department of Pharmacy-Drug Sciences
University of Bari "A. Moro"
Via E. Orabona 4, 70125 Bari, Italy
Renzo.Luisi@uniba.it

[b] Dr. F. De Franco
Tes Pharma S.r.l.
Via Giovine Italia, 1, 06073 Solomeo, Corciano (PG), Italy

[c] Dr. L. Walsh-Benn
CAS – A division of the American Chemical Society, ACS International, Ltd.
2540 Olentangy River Road, Columbus, Ohio, 43202, United States of America

[d] Dr. E. Mesto, Prof. E. Schingaro
Department of Earth and Geoenvironmental Sciences,
University of Bari "A. Moro"
Via E. Orabona 4, 70125 Bari, Italy

[e] Prof. Antimo Gioiello
Department of Pharmaceutical Sciences
University of Perugia
Via del Liceo, 1, 06123 Perugia, Italy
Via Palmiro Togliatti, 20, 06073 Taverne di Corciano, Italy

Table of Contents

1. General Information S3

2. Synthesis of Sulfonium Salts S5

2.1. Unsuccessful direct approaches S5

2.2. Unsuccessful approaches towards the preparation of azetidine sulfonium salts S6

2.3. Optimized Synthesis S7

*2.3.1. Synthesis of cyclobutane analogue **8** S7*

*2.3.2. Synthesis of oxetane analogue **9** S9*

*2.3.3. Synthesis of azetidine analogue **10** S11*

3. Optimization Studies S14

4. NMR Studies S15

5. General Procedures S19

5.1. General Procedure 1: Synthesis of N-tosyl-imine. S19

5.2. General Procedure 2: Synthesis of α-trifluoromethyl alkenes. S19

5.3. General Procedure 3: Esterification of 2-phenylacrylic acid S20

5.4. General Procedure 4: Synthesis of spiro[2.3]hexanes and heteroatom-containing analogues S20

6. Electrophile collection S22

6.1. Alkene collection S22

6.2. Carbonyl collection S23

6.3. Imine collection S24

7. Synthesis & characterization of compounds S25

8. Additional Supporting Experiments S108

8.1. Carbene Pathway S108

8.2. Reactivity Comparison S109

9. X-Ray Crystallography S110

10. Predictive Analytics-Supported Target Interaction S113

11. In vitro studies S116

12. Computational Studies S117

12.1. GitHub Repository S117

12.2. Calculation of Gibbs Free Energy for Carbene Formation S117

12.3. Bioisostere Identification through Clustering Approach S135

13. References S153

14. NMR spectra S155

1. General Information

Solvents, Reagents & Reactions

Chemical symbols are used with their standard meanings. SI units and corresponding standard abbreviations are applied. Solvent evaporation was carried out using a Büchi R-300 rotary evaporator under reduced pressure (0–1000 mbar) with a bath temperature maintained between 35 and 40 °C. Reagents were purchased from commercial sources (Merck, BLD, ThermoFisher Scientific, Fluorochem) and used as received, unless otherwise stated. Lithium bis(trimethylsilyl)amide solution (1M in THF) and dry tetrahydrofuran were purchased from Merck and used as received.

Chromatography

Thin-layer chromatography (TLC) was performed on aluminum-backed silica gel plates (0.25 mm, pre-coated with fluorescent indicator 60 F254, Merck) and visualized under UV light (λ = 254 nm). Flash column chromatography was carried out using silica gel (40–63 µm, Geduran, Merck) with pressure applied via head bellows. All chromatography solvents were obtained from commercial suppliers and used as received.

Analysis, Spectroscopy and Spectrometry of compounds

Nuclear magnetic resonance (NMR) spectra were acquired on a Bruker Ascend-400 spectrometer operating at 400 MHz for ^{1}H NMR, 101 MHz for ^{13}C{^{1}H} NMR, and 377 MHz for ^{19}F NMR. Measurements were conducted at room temperature, with chemical shifts referenced to the residual non-deuterated solvent peak. Chemical shifts (δ) are given in parts per million (ppm), reported to two decimal places for ^{1}H and ^{19}F signals, and to one decimal place for ^{13}C signals. Signal multiplicities are denoted as singlet (s), doublet (d), triplet (t), quartet (q), pentet (p), multiplet (m), broad (br), or appropriate combinations. Coupling constants (J) are provided in hertz (Hz), rounded to the nearest 0.1 Hz. Infrared (IR) spectra were obtained using a ThermoScientific Nicolet Summit Pro FTIR spectrometer. Samples were analyzed neat, and characteristic absorption bands (ν_{max}) are reported in wavenumbers (cm^{-1}). High-resolution mass spectrometry (HRMS) analyses were conducted via electrospray ionization (ESI).

Naming of compounds

Compound names were generated using ChemDraw Professional 20.0 (PerkinElmer), in accordance with IUPAC nomenclature conventions.

2. Synthesis of Sulfonium Salts

2.1. Unsuccessful direct approaches

1)

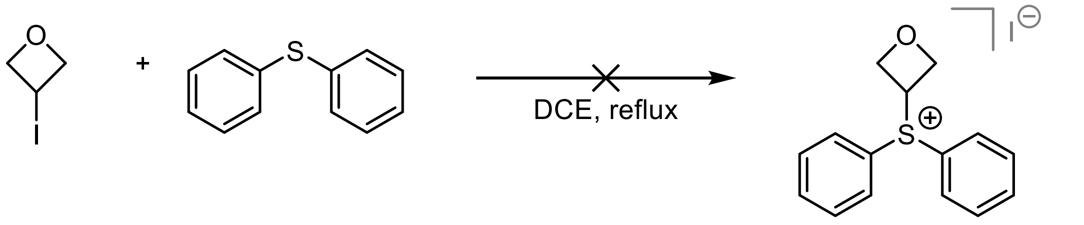

2)

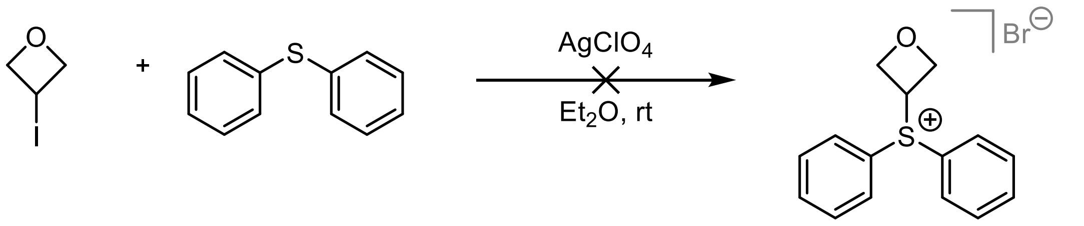

3)

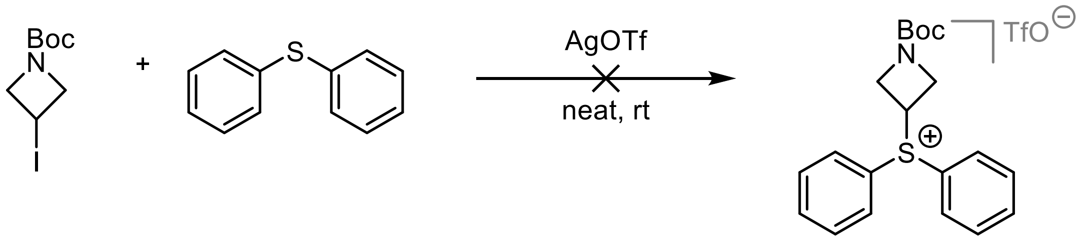

4)

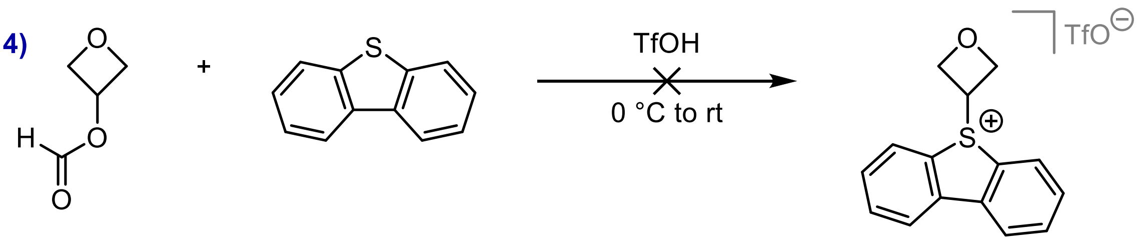

5)

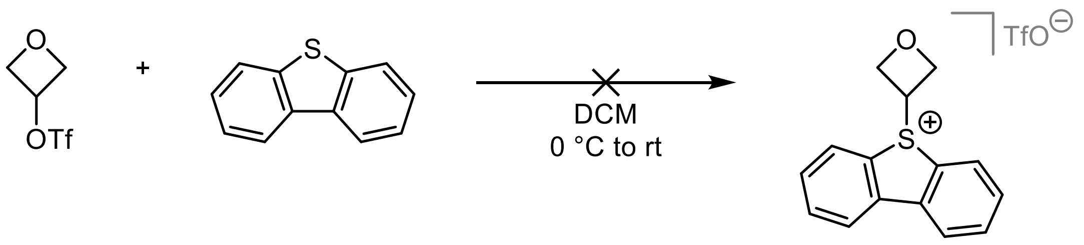

2.2. Unsuccessful approaches towards the preparation of azetidine sulfonium salts

1)

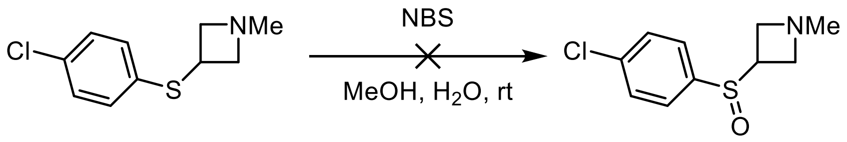

2)

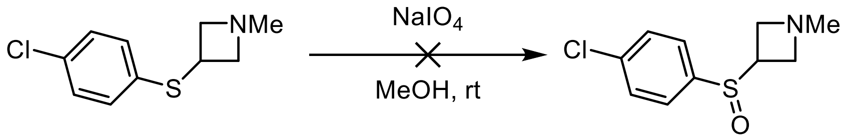

3)

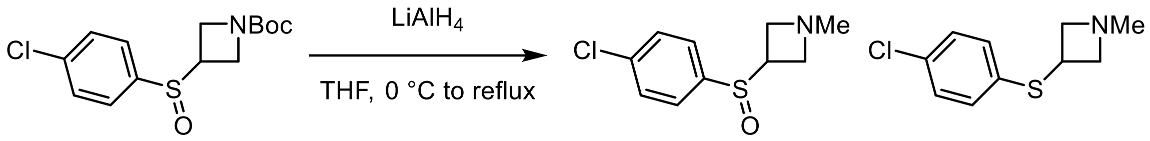

Desired product *not detected*

Undesired product *exclusive product*

4)

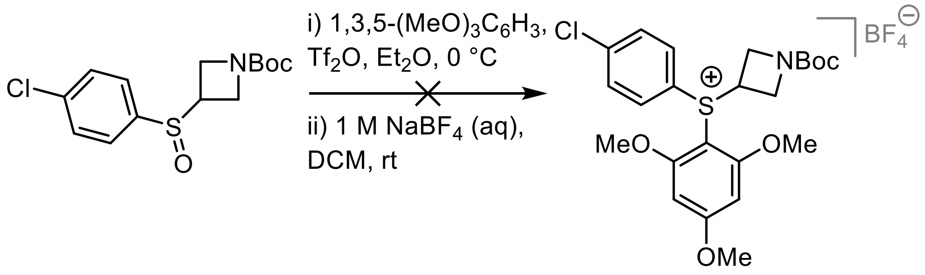

5)

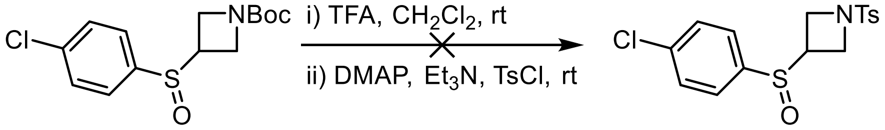

2.3. Optimized Synthesis

2.3.1. Synthesis of cyclobutane analogue **8**

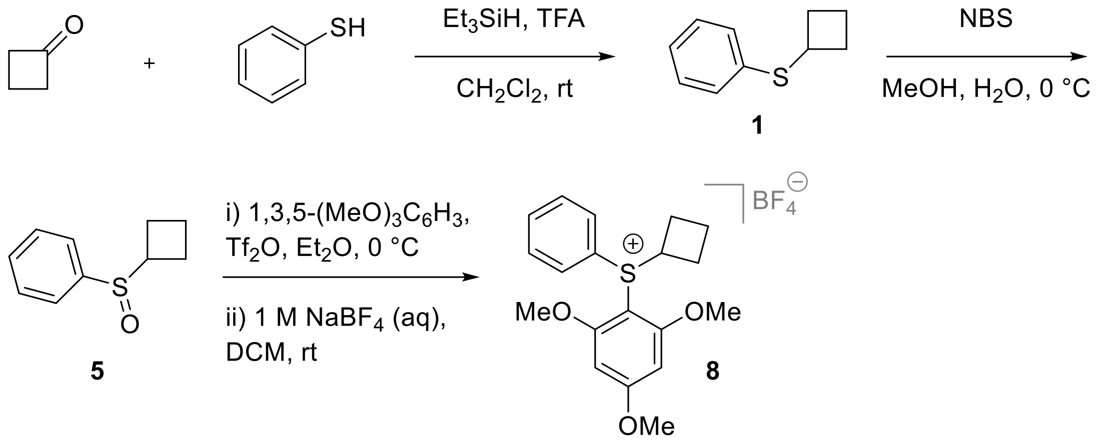

a) **Synthesis of cyclobutyl(phenyl)sulfane 1**

Prepared according to a modified literature procedure[1]

To a solution of cyclobutanone (2.5 g, 35 mmol) and thiophenol (3.0 mL, 29 mmol) in dichloromethane (30 mL) cooled to 0 °C, was added dropwise trifluoroacetic acid (20 mL). To the resulting solution, triethylsilane (8.0 mL, 50 mmol) was then added dropwise over three minutes while maintaining the internal temperature below 10 °C. The reaction mixture was then stirred overnight while allowing it to warm to room temperature. Upon completion of the reaction, water (50 mL) and dichloromethane (40 mL) were added. The aqueous phase was extracted with dichloromethane (3 x 20 mL), and the combined organic phase was washed with water (3 x 50 mL), followed by saturated aqueous sodium carbonate solution (2 x 50 mL), and saturated aqueous sodium chloride solution (2 x 50 mL), before being dried over Na_2SO_4, filtered, and concentrated under reduced pressure. The crude material was purified by flash column chromatography (hexane) to afford cyclobutyl(phenyl)sulfane **1** as a colourless oil (2.81 g, 17.1 mmol, 59%).

^{1}H-NMR (400 MHz, $CDCl_3$): δ_H 7.30 – 7.22 (5H, m), 3.93 – 3.81 (1H, m), 2.51 – 2.41 (2H, m), 2.17 – 1.91 (4H, m)

^{13}C{^{1}H}-NMR (101 MHz, $CDCl_3$): δ_C 137.1, 129.2, 128.9, 125.8, 40.4, 30.8, 18.9

The spectroscopic data are in agreement with those previously reported.[1]

b) **Synthesis of (cyclobutylsulfinyl)benzene 5**

To a solution of cyclobutyl(phenyl)sulfane **1** (2.50 g, 15 mmol) in methanol (40 mL) and de-ionized water (5 mL) cooled to 0 °C, was added *N*-bromosuccinimide (5.30 g, 30 mmol) in three portions. The resulting solution was allowed to stir for two hours while allowing to warm to room temperature, before dichloromethane (100 mL) was added. The organic phase was washed with de-ionized water (50 mL), saturated aqueous sodium hydrogen carbonate solution (2 x 50 mL), and saturated aqueous sodium chloride solution (50 mL), before being dried over Na_2SO_4, filtered, and concentrated under reduced pressure. The crude material was

purified by flash column chromatography (50% EtOAc/hexane) to afford (cyclobutylsulfinyl)benzene **5** (1.50 g, 8.32 mmol, 55%) as a yellow oil.

^{1}H-NMR (400 MHz, $CDCl_3$): δ_H 7.55 (2H, m), 7.50 – 7.42 (3H, m), 3.45 (1H, m), 2.57 (1H, m), 2.46 (1H, m), 2.14 (1H, m), 2.00 – 1.85 (2H, m), 1.71 (1H, m)

^{13}C{^{1}H}-NMR (101 MHz, $CDCl_3$): δ_C 142.7, 130.8, 129.1, 124.1, 57.5, 22.5, 19.0, 17.4

IR (neat, ν cm^{-1}): 3470, 3055, 2985, 2942, 1478, 1443, 1086

HRMS (ESI+): m/z calcd for $C_{10}H_{13}OS$ $[M+H]^+$ 181.0687, found 181.0681

c) Synthesis of cyclobutyl(phenyl)(2,4,6-trimethoxyphenyl)sulfonium tetrafluoroborate 8

To a solution of (cyclobutylsulfinyl)benzene **5** (1.50 g, 8.32 mmol) and 1,3,5-trimethoxybenzene (1.54 g, 9.15 mmol) in freshly distilled diethyl ether (70 mL) cooled to 0 °C, was added trifluoromethanesulfonic anhydride (2.58 g, 1.63 mL, 9.15 mmol) dropwise over 15 minutes. The resulting red solution was stirred at 0 °C for further one hour, before the solvent was decanted from the formed solid residues. The solid was carefully washed with diethyl ether (3 x 50 mL), before being dissolved in dichloromethane (50 mL). An aqueous solution of sodium tetrafluoroborate (1M, 30 mL) was then added, and the reaction mixture was allowed to stir for 30 mins, before being diluted with deionized water (50 mL) and dichloromethane (50 mL). The aqueous phase was extracted with dichloromethane (3 x 30 mL), and the combined organic phase was washed with water (2 x 40 mL), and saturated aqueous sodium chloride solution (40 mL), before being dried over Na_2SO_4, filtered and concentrated under reduced pressure (*Note: additional solvation/evaporation cycles with diethyl ether were performed*). To the resulting brown oil, diethyl ether (40 mL) was added, before being sonicated for one hour. The formed solid was filtered and dried under reduced pressure to obtain the title compound **8** (3.00 g, 7.15 mmol, 86%) as a brown solid.

^{1}H-NMR (400 MHz, $CDCl_3$): δ_H 7.72 – 7.66 (2H, m), 7.63 – 7.58 (3H, m), 6.24 (2H, s), 5.60 – 5.55 (1H, m), 3.95 (6H, s), 3.92 (3H, s), 2.84 – 2.80 (1H, m), 2.51 – 2.40 (1H, m), 2.45 – 2.30 (2H, m), 2.30 – 2.15 (2H, m)

^{13}C{^{1}H}-NMR (101 MHz, $CDCl_3$): δ_C 168.6, 163.0, 133.2, 130.9, 129.3, 125.5, 92.5, 87.0, 57.2, 56.6, 47.3, 28.1, 26.3, 18.5

IR (neat, ν cm^{-1}): 2952, 1594, 1518, 1476, 1419, 1352, 1268

HRMS (ESI+): m/z calcd for $C_{19}H_{23}O_3S$ $[M]^+$ 331.1362, found 331.1361

2.3.2. Synthesis of oxetane analogue **9**

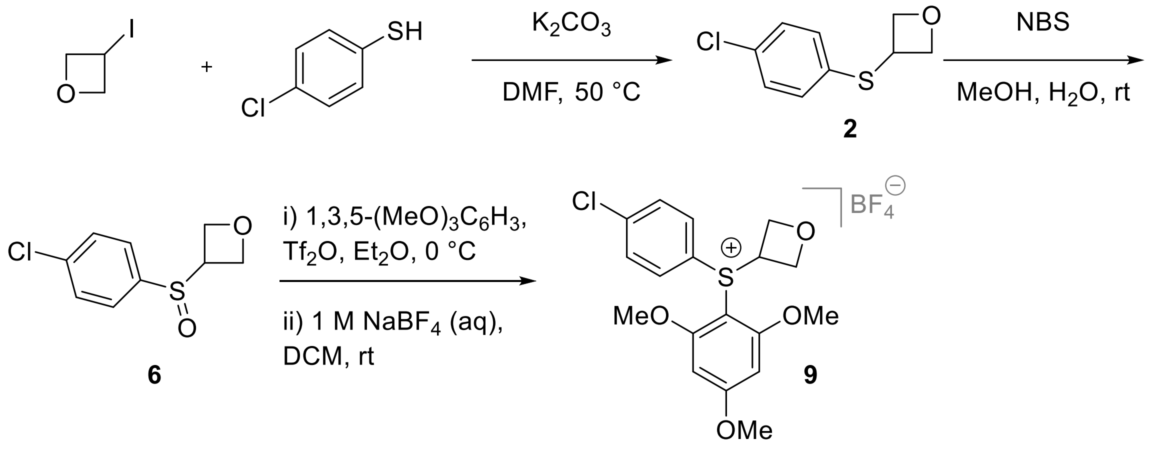

a) **Synthesis of 3-((4-chlorophenyl)thio)oxetane 2**

To a solution of 3-iodooxetane (2.0 g, 11 mmol) and 4-chlorothiophenol (2.3 g, 16 mmol) in *N,N*-dimethylformamide (10 mL) was added potassium carbonate (3.0 g, 22 mmol) at room temperature. The resulting reaction mixture was heated to 60 °C for six hours, before being cooled to room temperature and poured into ice-water (50 mL). The aqueous phase was extracted with diethyl ether (3 x 40 mL), before the combined organic phase was washed with deionized water (3 x 50 mL) and saturated aqueous sodium chloride solution (2 x 50 mL). The organic phase was dried over Na_2SO_4, filtered, and concentrated under reduced pressure to afford 3-((4-chlorophenyl)thio)oxetane **2** (2.11 g, 10.5 mmol, 96%) as an off-white solid, which was used in the next step without further purification.

^{1}H-NMR (400 MHz, $CDCl_3$): δ_H 7.28 (2H, d, *J* = 6.5 Hz), 7.17 (2H, d, *J* = 6.5 Hz), 5.03 (2H, m), 4.64 (2H, t, *J* = 6.5 Hz), 4.44 (1H, m)

^{13}C{^{1}H}-NMR (101 MHz, $CDCl_3$): δ_C 133.2, 131.3, 129.5, 129.5, 78.0, 39.9

IR (neat, ν cm^{-1}): 2953, 2872, 1475, 1095, 1010

HRMS: *exact mass not found in ESI+ or ESI- mode*

b) **Synthesis of 3-((4-chlorophenyl)sulfinyl)oxetane 6**

To a solution of 3-((4-chlorophenyl)thio)oxetane **2** (2.30 g, 11.5 mmol) in methanol (35 mL) and de-ionized water (4 mL) cooled to 0 °C, was added *N*-bromosuccinimide (4.10 g, 23 mmol) in three portions. The resulting solution was allowed to stir for two hours while allowing to warm to room temperature, before dichloromethane (80 mL) was added. The organic phase was washed with de-ionized water (50 mL), saturated aqueous sodium hydrogen carbonate solution (2 x 50 mL), and saturated aqueous sodium chloride solution (50 mL), before being dried over Na_2SO_4, filtered, and concentrated under reduced pressure. The crude material was purified by flash column chromatography (70% EtOAc/hexane) to afford 3-((4-chlorophenyl)sulfinyl)oxetane **6** (1.90 g, 8.77 mmol, 76%) as a white solid.

^{1}H-NMR (400 MHz, $CDCl_3$): δ_H 7.56 – 7.48 (4H, m), 5.02 (1H, m), 4.90 – 4.82 (2H, m), 4.59 (1H, t, *J* = 7.5 Hz), 4.05 (1H, app. tt, *J* = 7.9, 6.3 Hz)

^{13}C{^{1}H}-NMR (101 MHz, $CDCl_3$): δ_C 140.0, 138.0, 130.0, 125.4, 70.2, 65.4, 57.2

IR (neat, ν cm^{-1}): 2947, 2878, 1711, 1475, 1312, 1166

HRMS (ESI+): m/z calcd for $C_9H_9{}^{35}ClO_2SNa$ $[M+Na]^+$ 238.9904, found 238.9896

c) Synthesis of (4-chlorophenyl)(oxetan-3-yl)(2,4,6-trimethoxyphenyl)sulfonium tetrafluoroborate 9

To a solution of 3-((4-chlorophenyl)sulfinyl)oxetane **6** (1.90 g, 8.77 mmol) and 1,3,5-trimethoxybenzene (1.63 g, 9.69 mmol) in freshly distilled diethyl ether (100 mL) cooled to 0 °C, was added trifluoromethanesulfonic anhydride (2.70 g, 1.73 mL, 9.69 mmol) dropwise over 15 minutes. The resulting red solution was stirred at 0 °C for further one hour, before the solvent was decanted from the formed solid residues. The solid was carefully washed with diethyl ether (3 x 50 mL), before being dissolved in dichloromethane (50 mL). An aqueous solution of sodium tetrafluoroborate (1M, 30 mL) was then added, and the reaction mixture was allowed to stir for 30 mins, before being diluted with deionized water (50 mL) and dichloromethane (50 mL). The aqueous phase was extracted with dichloromethane (3 x 30 mL), and the combined organic phase was washed with water (2 x 40 mL), and saturated aqueous sodium chloride solution (40 mL), before being dried over Na_2SO_4, filtered and concentrated under reduced pressure. The resulting crude material was purified by flash column chromatography (0.5% to 1% MeOH/CH_2Cl_2) to afford (4-chlorophenyl)(oxetan-3-yl)(2,4,6-trimethoxyphenyl)sulfonium tetrafluoroborate **9** (837 mg, 1.84 mmol, 21%) as an off-white solid.

^{1}H-NMR (400 MHz, $CDCl_3$): δ_H 7.75 (2H, d, *J* = 8.8 Hz), 7.61 (2H, d, *J* = 8.7 Hz), 6.24 (2H, s), 6.08 (1H, m), 5.43 (1H, dd, *J* = 8.3, 6.6 Hz), 5.23 (1H, m), 4.51 (2H, m), 3.97 (6H, s), 3.91 (3H, s)

^{13}C{^{1}H}-NMR (101 MHz, $CDCl_3$): δ_C 169.2, 163.0, 140.8, 131.5, 131.4, 122.2, 92.6, 86.0, 73.0, 72.0, 57.3, 56.6, 49.0

IR (neat, ν cm^{-1}): 2948, 2883, 1593, 1577, 1475, 1418, 1351, 1210, 1127

HRMS (ESI+): m/z calcd for $C_{18}H_{20}{}^{35}ClO_4S$ $[M]^+$ 367.0765, found 367.0777

2.3.3. Synthesis of azetidine analogue **10**

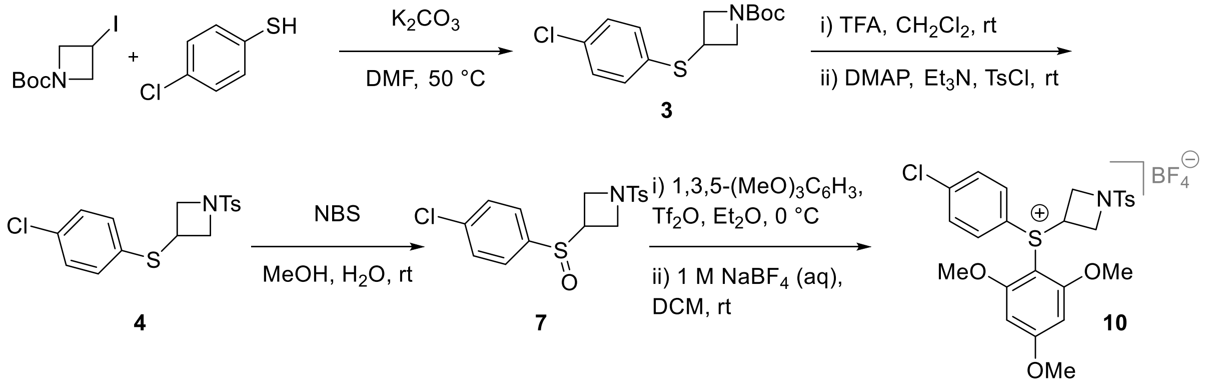

a) **Synthesis of *tert*-butyl 3-((4-chlorophenyl)thio)azetidine-1-carboxylate 3**

To a solution of 1-Boc-3-iodoazetidine (2.0 g, 7.1 mmol) and 4-chlorothiophenol (1.2 g, 8.3 mmol) in *N,N*-dimethylformamide (10 mL) was added potassium carbonate (1.93 g, 14 mmol) at room temperature. The resulting reaction mixture was heated to 60 °C for six hours, before being cooled to room temperature and poured into ice-water (50 mL). The aqueous phase was extracted with diethyl ether (3 x 40 mL), before the combined organic phase was washed with deionized water (3 x 50 mL) and saturated aqueous sodium chloride solution (2 x 50 mL). The organic phase was dried over Na_2SO_4, filtered, and concentrated under reduced pressure to afford *tert*-butyl 3-((4-chlorophenyl)thio)azetidine-1-carboxylate **3** (2.07 g, 6.90 mmol, 97%) as an off-white solid, which was used in the next step without further purification.

^{1}H-NMR (400 MHz, $CDCl_3$): δ_H 7.28 (2H, m), 7.18 (2H, m), 4.31 (2H, dd, *J* = 9.1, 7.9 Hz), 3.96 (1H, m), 3.85 (2H, dd, *J* = 9.1, 5.4 Hz), 1.43 (9H, s)

^{13}C{^{1}H}-NMR (101 MHz, $CDCl_3$): δ_C 156.1, 133.2, 133.2, 131.2, 129.5, 80.1, 56.0 (broad), 34.2, 28.5

IR (neat, ν cm^{-1}): 2976, 2881, 1700, 1477, 1391, 1366, 1137, 1096

HRMS (ESI+): m/z calcd for $C_{14}H_{18}{}^{35}ClNO_2SNa$ $[M+Na]^+$ 322.0639, found 322.0633

b) **Synthesis of 3-((4-chlorophenyl)thio)-1-tosylazetidine 4**

To a solution of *tert*-butyl 3-((4-chlorophenyl)thio)azetidine-1-carboxylate **3** (3.20 g, 10.7 mmol) in dichloromethane (50 mL) cooled to 0 °C, was added trifluoroacetic acid (8 mL, 107 mmol) dropwise to maintain the internal temperature below 10 °C. The reaction mixture was stirred at 0 °C for 1 hour, before triethylamine (17.6 mL, 130 mmol), 4-dimethylaminopyridine (136 mg, 1.12 mmol), and 4-toluenesulfonyl chloride (2.45 g, 12.8 mmol) were added consecutively. The solution was allowed to stir at room temperature overnight, before it was diluted with deionized water (100 mL) and dichloromethane (50 mL). The aqueous phase was extracted with dichloromethane (3 x 30 mL), and the combined organic phase was washed with deionized water (2 x 40 mL), saturated aqueous sodium hydrogen carbonate solution (2 x 40 mL) and saturated aqueous sodium chloride solution (50 mL), before being dried over Na_2SO_4, filtered, and concentrated under reduced pressure to afford 3-((4-chlorophenyl)thio)-

1-tosylazetidine **4** (3.51 g, 9.92 mmol, 92%) as a white solid, which was used in the next step without further purification.

^{1}H-NMR (400 MHz, $CDCl_3$): δ_H 7.66 (2H, d, *J* = 8.0 Hz), 7.35 (2H, d, *J* = 8.0 Hz), 7.21 (2H, m), 7.10 (2H, m), 4.13 (2H, app. t, *J* = 8.3 Hz), 3.84 (1H, m), 3.63 (2H, dd, *J* = 8.6, 6.6 Hz), 2.47 (3H, s)

^{13}C{^{1}H}-NMR (101 MHz, $CDCl_3$): δ_C 144.5, 113.9, 132.6, 131.6, 131.5, 129.9, 129.5, 128.4, 57.2, 34.2, 21.8

IR (neat, ν cm^{-1}): 3429, 2926, 1476, 1344, 1158, 1095

HRMS (ESI+): m/z calcd for $C_{16}H_{16}{}^{35}ClNO_2S_2Na$ $[M+Na]^+$ 376.0209, found 376.0263

c) Synthesis of 3-((4-chlorophenyl)sulfinyl)-1-tosylazetidine 7

To a solution of 3-((4-chlorophenyl)thio)-1-tosylazetidine **4** (3.47 g, 9.80 mmol) in methanol (40 mL) and de-ionized water (4 mL) cooled to 0 °C, was added *N*-bromosuccinimide (3.48 g, 19.6 mmol) in three portions. The resulting solution was allowed to stir for two hours while allowing to warm to room temperature, before dichloromethane (80 mL) was added. The organic phase was washed with de-ionized water (50 mL), saturated aqueous sodium hydrogen carbonate solution (2 x 50 mL), and saturated aqueous sodium chloride solution (50 mL), before being dried over Na_2SO_4, filtered, and concentrated under reduced pressure, to afford 3-((4-chlorophenyl)sulfinyl)-1-tosylazetidine **7** (3.30 g, 8.92 mmol, 91%) as a yellow solid, which was used in the next step without purification.

^{1}H-NMR (400 MHz, $CDCl_3$): δ_H 7.71 (2H, m), 7.47 (2H, m), 7.45 – 7.35 (4H, m), 4.11 (1H, dd, *J* = 8.8, 6.5 Hz), 4.00 (1H, t, *J* = 8.8 Hz), 3.93 (1H, dd, *J* = 9.0, 6.6 Hz), 3.69 (1H, m), 3.49 (1H, m), 2.47 (3H, s)

^{13}C{^{1}H}-NMR (101 MHz, $CDCl_3$): δ_C 144.8, 139.5, 138.3, 131.6, 130.1, 130.0, 128.6, 125.4, 50.7, 50.1, 47.9, 21.8

IR (neat, ν cm^{-1}): 2942, 1716, 1475, 1342, 1156, 1087, 1053

HRMS (ESI+): m/z calcd for $C_{16}H_{16}{}^{35}ClNO_3S_2Na$ $[M+Na]^+$ 392.0152, found 392.0188

d) Synthesis of (4-chlorophenyl)(1-tosylazetidin-3-yl)(2,4,6-trimethoxyphenyl)sulfonium tetrafluoroborate 10

To a solution of 3-((4-chlorophenyl)sulfinyl)-1-tosylazetidine **7** (2.11 g, 5.70 mmol) and 1,3,5-trimethoxybenzene (1.15 g, 6.84 mmol) in freshly distilled diethyl ether (100 mL) cooled to 0 °C, was added trifluoromethanesulfonic anhydride (1.95 g, 1.20 mL, 6.84 mmol) dropwise over 15 minutes. The resulting red solution was stirred at 0 °C for further one hour, before the solvent was decanted from the formed oily residues. The oil was carefully washed with diethyl ether (3 x 50 mL), before being dissolved in dichloromethane (50 mL). An aqueous solution of sodium tetrafluoroborate (1M, 20 mL) was then added, and the reaction mixture was allowed to stir for 30 mins, before being diluted with deionized water (50 mL) and dichloromethane

(50 mL). The aqueous phase was extracted with dichloromethane (3 x 30 mL), and the combined organic phase was washed with water (2 x 40 mL), and saturated aqueous sodium chloride solution (40 mL), before being dried over Na_2SO_4, filtered and concentrated under reduced pressure (*Note: additional solvation/evaporation cycles with diethyl ether were performed*), to obtain the title compound **10** (2.71 g, 4.46 mmol, 78%) as a brown solid.

^{1}H-NMR (400 MHz, $CDCl_3$): δ_H 7.74 (2H, m), 7.66 (2H, d, *J* = 8.0 Hz), 7.58 (2H, m), 7.41 (2H, d, *J* = 8.0 Hz), 6.18 (2H, s), 5.61 (1H, m), 4.41 (1H, dd, *J* = 10.3, 7.0 Hz), 4.17 (1H, dd, *J* = 10.2, 7.4 Hz), 3.95 (6H, s), 3.89 (3H, s), 3.83 – 3.75 (2H, m), 2.47 (3H, s)

^{13}C{^{1}H}-NMR (101 MHz, $CDCl_3$): δ_C 169.2, 163.0, 145.7, 141.2, 131.7, 131.5, 130.5, 129.9, 128.5, 121.9, 92.4, 86.1, 57.3, 56.5, 53.7, 52.7, 42.9, 21.8

IR (neat, v cm^{-1}): 2948, 1595, 1476, 1419, 1351, 1161, 1057, 1008

HRMS (ESI+): m/z calcd for $C_{25}H_{27}{}^{35}ClNO_5S_2$ $[M]^+$ 520.1014, found 520.0989

3. Optimization Studies

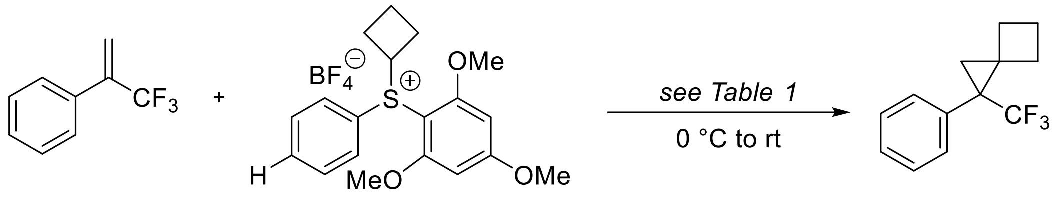

(1 equiv.) (1.3 equiv.)

Table 1: Optimization of reaction conditions

entry	solvent	base	equiv. base	time of deprotonation [min]	quant. ^{1}H-NMR yield (%)
**1**	THF	LiHMDS	1.6	20	0
**2**	THF	LiHMDS	1.6	5	27
**3**	DCM	DIPEA	1.6	5	0
**4**	THF	KO^{t}Bu	1.6	5	68
**5**	PhMe	LiHMDS	1.6	internal quenching	40
**6**	THF	LiHMDS	1.6	internal quenching	95
**7**	THF	DIPEA	1.6	internal quenching	0
**8**	THF	NaH	1.6	internal quenching	0
**9**	THF	KO^{t}Bu	1.6	internal quenching	62

4. NMR Studies

In order to assess the stability of the sulfonium ylid in solution, ^{1}H NMR studies were conducted. The objective was to observe the progress of the reaction under internal quenching conditions, as well as external quenching conditions (deprotonation time = 5 min). In line with the optimized conditions, the study was performed in d_8-THF. The reaction between oxetane sulfonium salt **9** and benzophenone was selected for this purpose to facilitate monitoring of the reaction.

Internal quenching conditions

^{1}H-NMR spectrum of benzophenone and sulfonium salt **9**

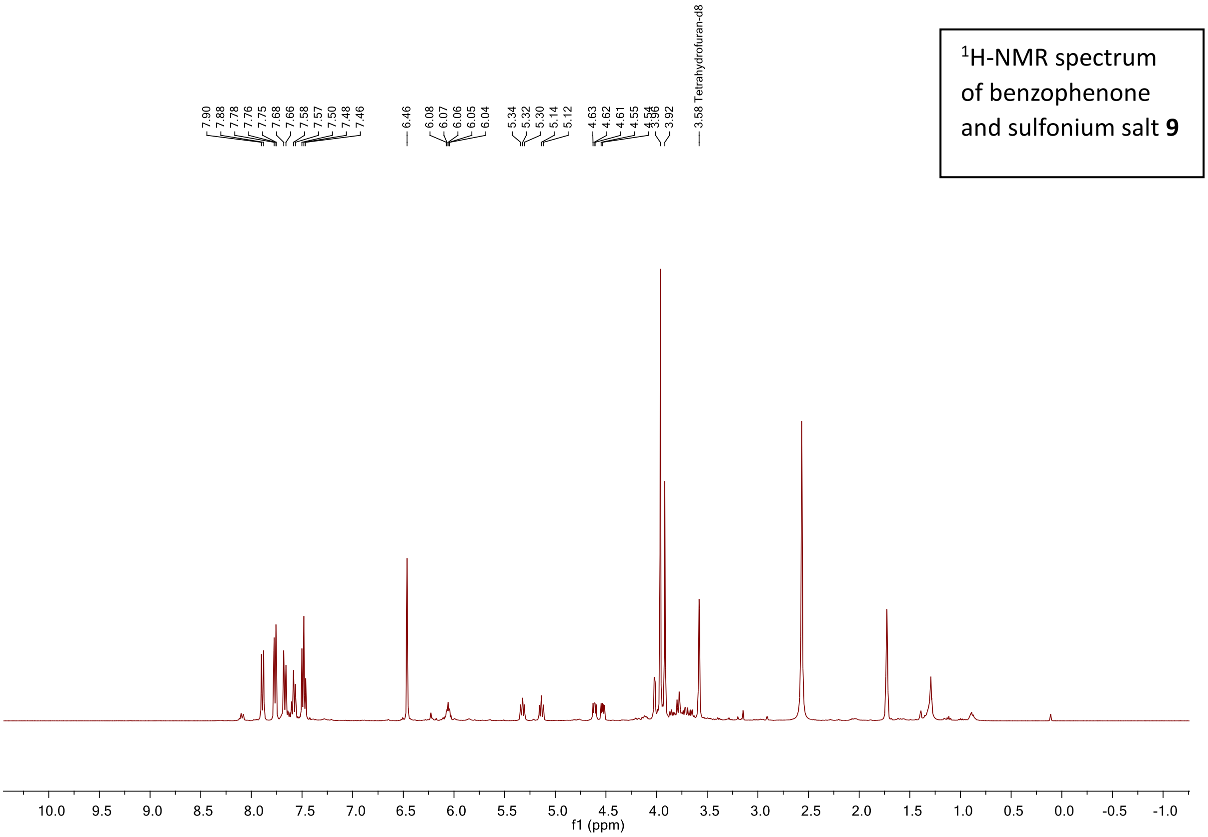

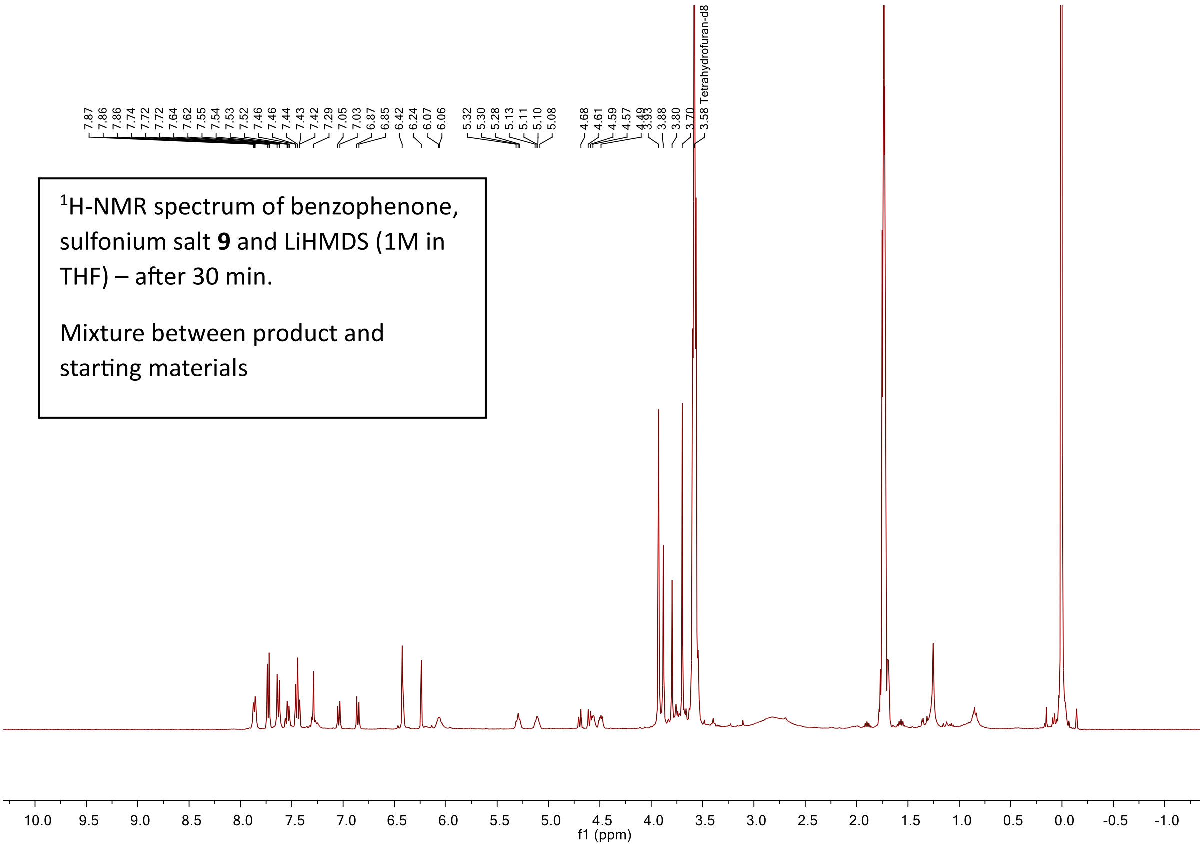
7.87 7.86 7.86 7.74 7.72 7.72 7.64 7.62 7.55 7.54 7.53 7.52 7.46 7.46 7.44 7.43 7.42 7.29 7.05 7.03 6.87 6.85 6.42 6.24 6.07 6.06 5.32 5.30 5.28 5.13 5.11 5.10 5.08 4.68 4.61 4.59 4.57 4.49 3.93 3.88 3.80 3.70 3.58 Tetrahydrofuran-d8
^{1}H-NMR spectrum of benzophenone, sulfonium salt **9** and LiHMDS (1M in THF) – after 30 min.
Mixture between product and starting materials
f1 (ppm)

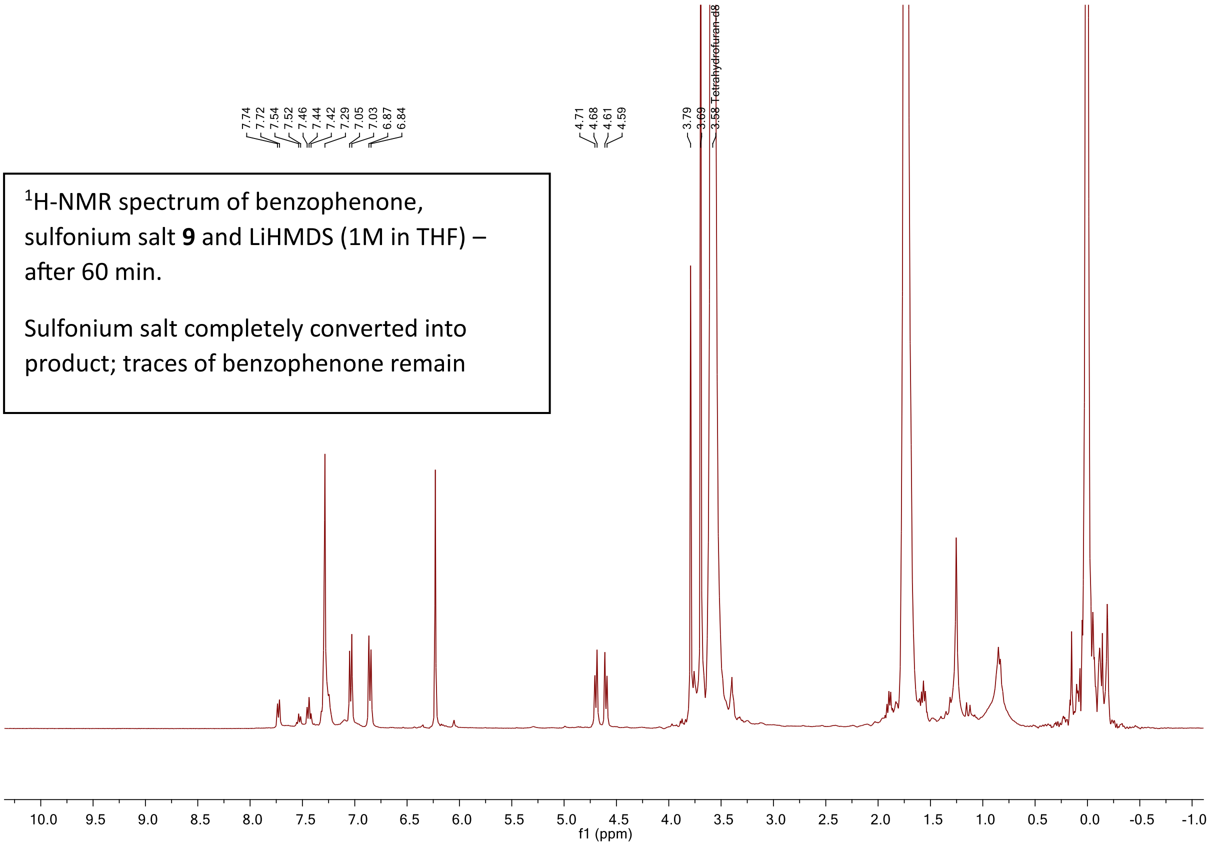
7.74 7.72 7.54 7.52 7.46 7.44 7.42 7.29 7.05 7.03 6.87 6.84 4.71 4.68 4.61 4.59 3.79 3.69 3.58 Tetrahydrofuran-d8
^{1}H-NMR spectrum of benzophenone, sulfonium salt **9** and LiHMDS (1M in THF) – after 60 min.
Sulfonium salt completely converted into product; traces of benzophenone remain
f1 (ppm)

External quenching conditions

^{1}H-NMR spectrum of sulfonium salt **9**

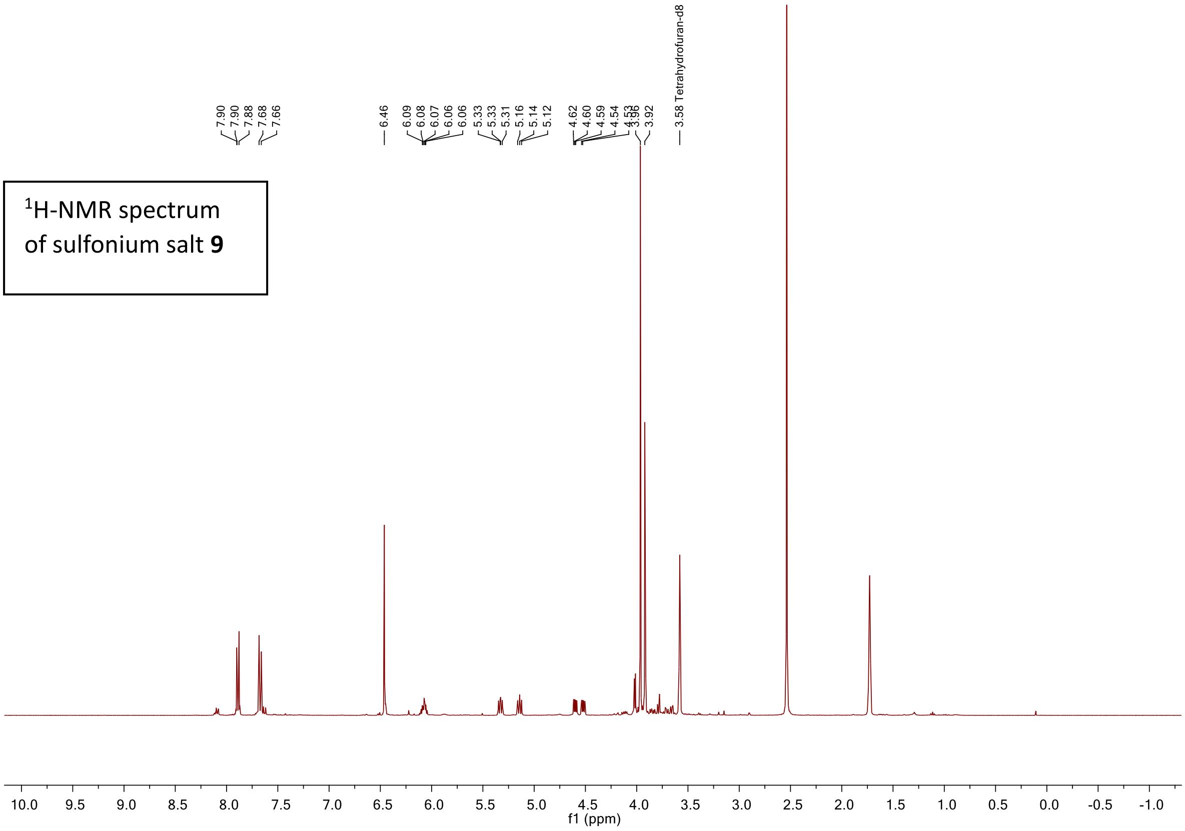

^{1}H-NMR spectrum of sulfonium salt **9** and LiHMDS (1M in THF) – 5 minutes

Decomposition of sulfonium ylid into sulfide by-product

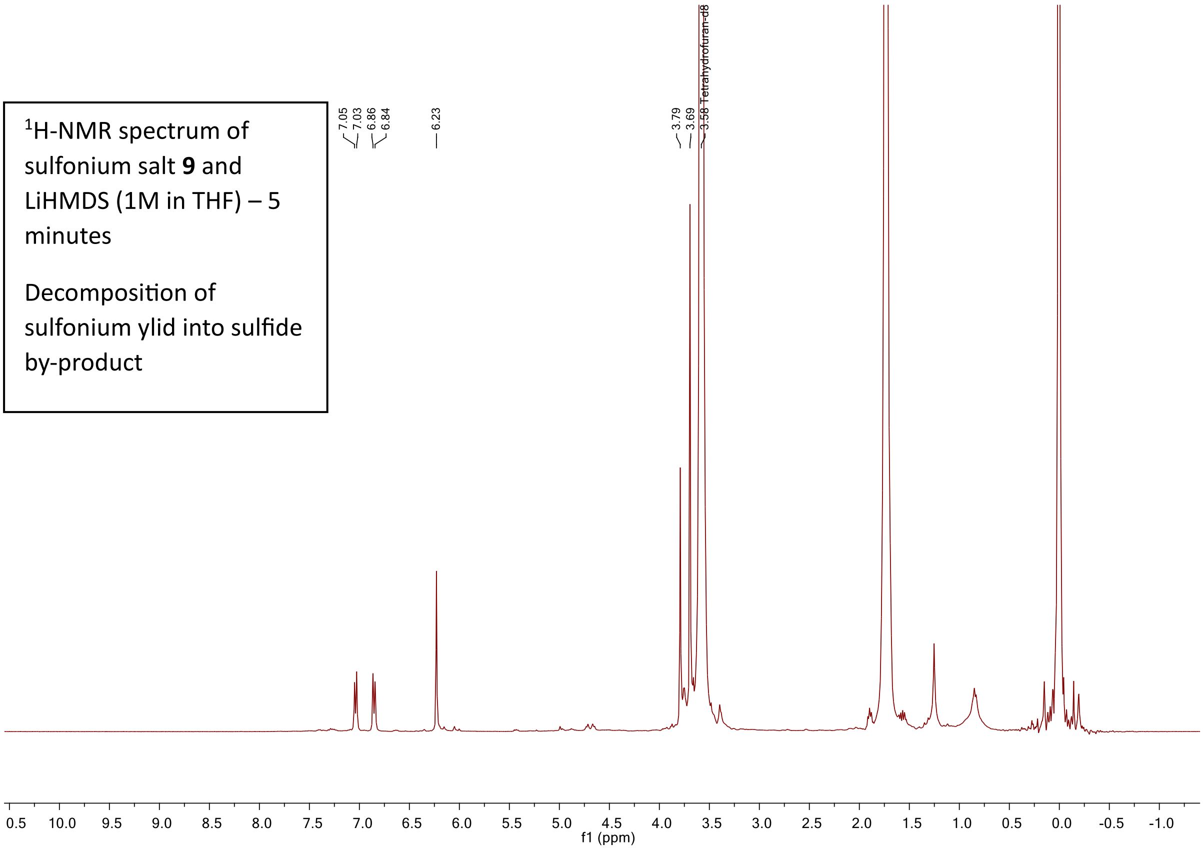

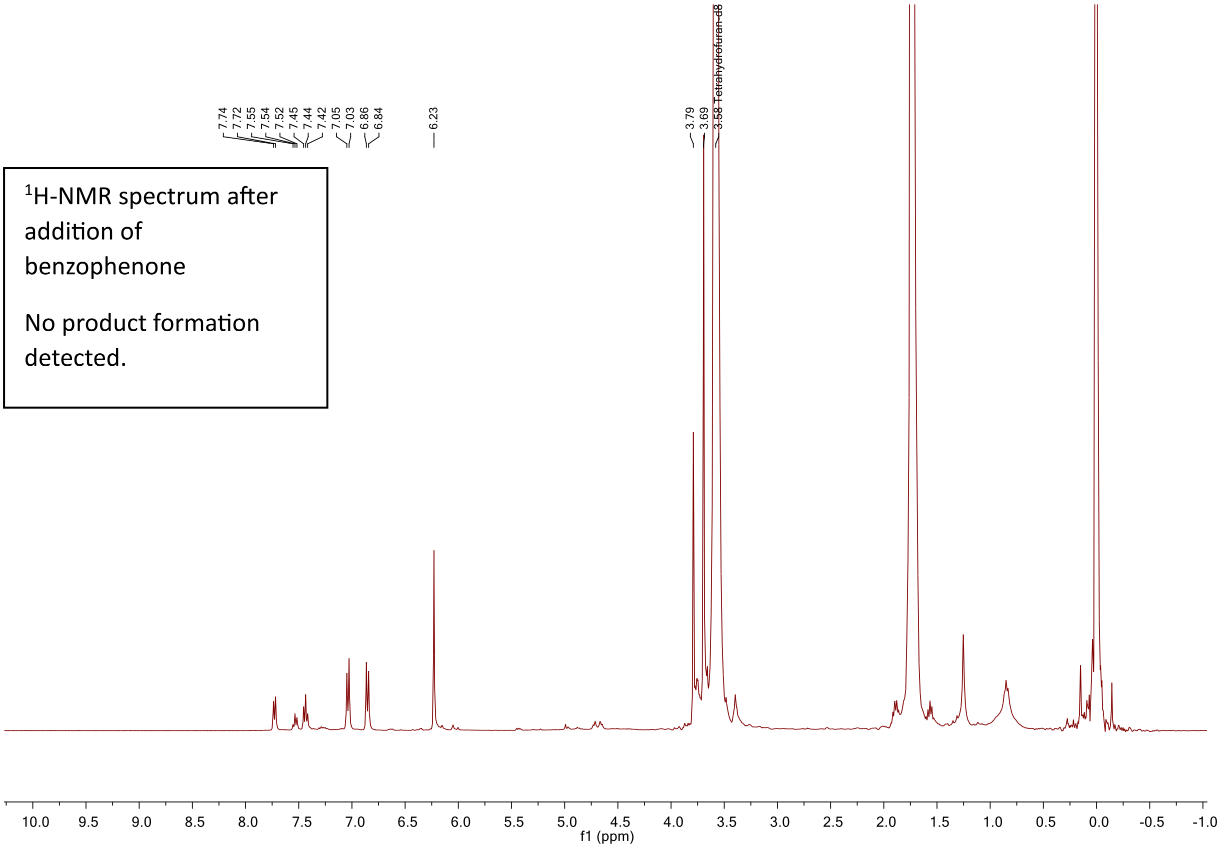
7.74
7.72
7.55
7.54
7.52
7.45
7.44
7.42
7.05
7.03
6.86
6.84
6.23
3.79
3.69
3.58 Tetrahydrofuran-d8
^{1}H-NMR spectrum after addition of benzophenone
No product formation detected.
10.0 9.5 9.0 8.5 8.0 7.5 7.0 6.5 6.0 5.5 5.0 4.5 4.0 3.5 3.0 2.5 2.0 1.5 1.0 0.5 0.0 -0.5 -1.0
f1 (ppm)

5. General Procedures

5.1. General Procedure 1: Synthesis of *N*-tosyl-imine.

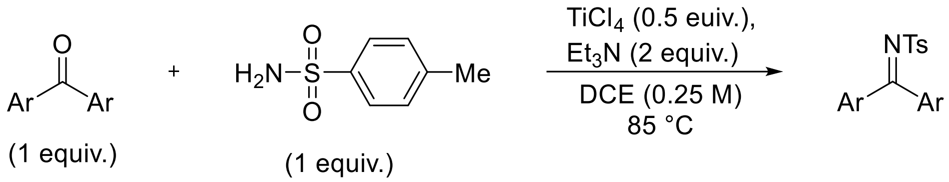

To a solution of ketone (1 equiv.) in 1,2-dichloroethane (0.25 M) were added 4-methylbenzenesulfonamide (1 equiv.), triethylamine (2 equiv.), and titanium tetrachloride (1.0 M in CH_2Cl_2, 0.5 equiv.) carefully dropwise. The resulting solution was stirred at room temperature for five minutes until gas evolution ceased. The resulting solution was then heated to reflux overnight, before it was cooled to room temperature, filtered through a pad of Celite, and concentrated under reduced pressure. Purification by flash column chromatography affords the pure title compounds.

5.2. General Procedure 2: Synthesis of α-trifluoromethyl alkenes.

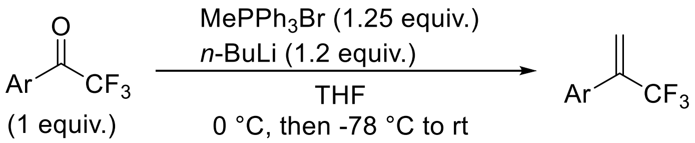

To a solution of methyltriphenylphosphonium bromide (1.25 equiv.) in THF (0.5 M) cooled to 0 °C, was added dropwise a solution of *n*-butyllithium (2.5 M in hexane, 1.2 equiv.). The reaction mixture was stirred at 0 °C for 10 minutes, then further cooled to –78 °C before a solution of the ketone (1 equiv., 1 M in THF) was added dropwise. The mixture was stirred overnight while allowing it to warm to room temperature. The reaction was quenched with water, and the aqueous phase was extracted with diethyl ether (3 × 20 mL). The organic phase was dried over sodium sulfate, filtered, and concentrated under reduced pressure to afford the crude material, which was purified by flash column chromatography.

5.3. General Procedure 3: Esterification of 2-phenylacrylic acid

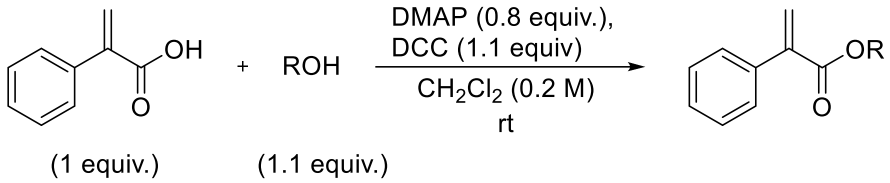

To a solution of 2-phenylacrylic acid (1 equiv.) and alcohol (1.1 equiv.) in dichloromethane (0.2 M) were added 4-dimethylaminopyridine (0.8 equiv.) and N,N'-dicyclohexylcarbodiimide (1.1 equiv.). The resulting solution was allowed to stir overnight at room temperature, before it was diluted with dichloromethane, and consecutively washed with saturated aqueous ammonium chloride solution twice and saturated aqueous sodium chloride solution twice. The organic phase was dried over sodium sulfate, filtered, and concentrated under reduced pressure to afford the crude material, which can be purified by flash column chromatography, if necessary.

5.4. General Procedure 4: Synthesis of spiro[2.3]hexanes and heteroatom-containing analogues

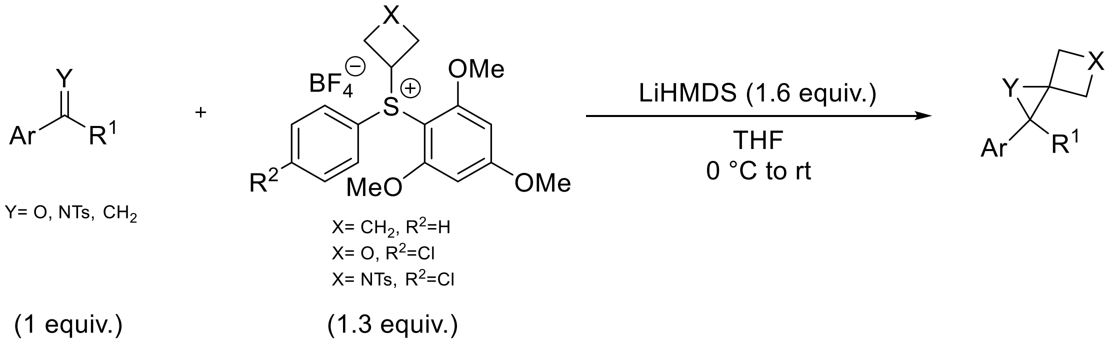

To a solution of electrophile (0.1 mmol, 1 equiv.) and the sulfonium salt (0.3 mmol, 1.3 equiv.) in THF (0.03 M) at 0 °C was added a solution of LiHMDS (1.0 M in THF, 1.6 equiv.) dropwise. The mixture was stirred at 0 °C for 10 minutes, then allowed to warm to the room temperature and stirred for an additional hour. The reaction was quenched with methanol (0.2 mL) and concentrated under reduced pressure to afford the crude material, which was purified by flash column chromatography as indicated in each entry.

Note: Particular caution is advised during the isolation of the spiro[2.3]hexanes by column chromatography, since:

a) *Many products cannot or can only poorly be visualized by UV light or $KMnO_4$ solution or iodine stain; however, some can be detected using an ethanolic phosphomolybdic acid solution*

b) Many spiro[2.3]hexanes have been found to have similar polarity compared to the sulfide by-product;

c) Some decomposition/rearrangement of the 1,5-dioxaspiro[2.3]hexane-core was observed upon purification with silica gel, which can only sometimes be prevented by pre-treatment with triethylamine.

6. Electrophile collection

6.1. Alkene collection

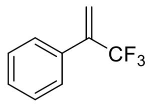

A

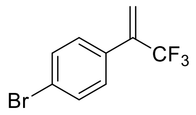

B

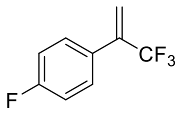

C

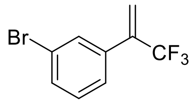

D

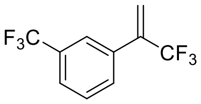

E

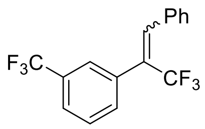

F

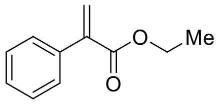

G

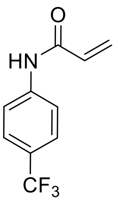

H

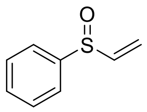

I

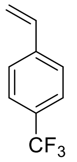

J

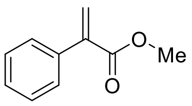

K

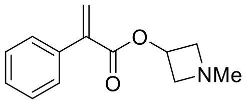

L

Alkenes **A**, **I** & **J** are commercially available.

For synthesis of **B** – **H** & **K** – **L** see **Synthesis and Characterization of Compounds**.

6.2. Carbonyl collection

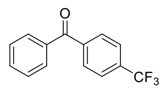
M

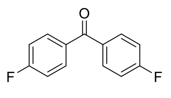
N

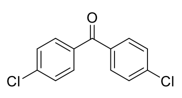
O

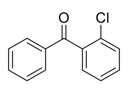
P

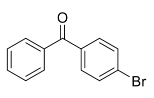
Q

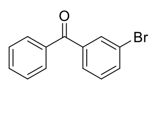
R

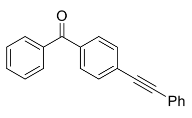
S

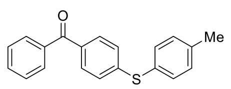
T

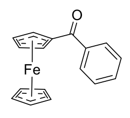
U

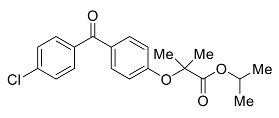
V

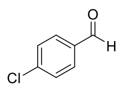
W

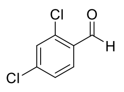
X

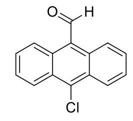
Y

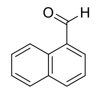
Z

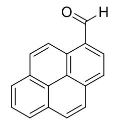
AA

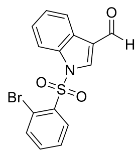
AB

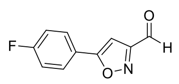
AC

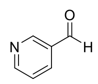
AD

Ketones **M** – **AA** & **AC** – **AD** are commercially available

For synthesis of **AB**, see **Synthesis & Characterization of compounds.**

6.3. Imine collection

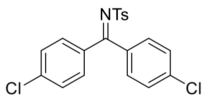

AE

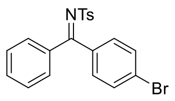

AF

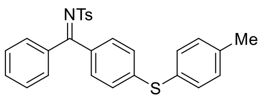

AG

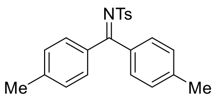

AH

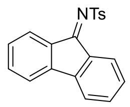

AI

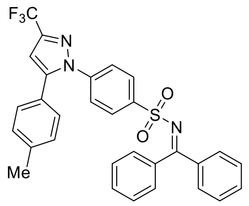

AJ

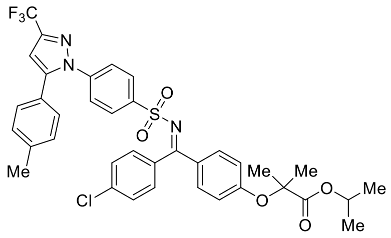

AK

See **Synthesis and Characterization of Compounds** for details on imine synthesis.

7. Synthesis & characterization of compounds

1-Bromo-4-(3,3,3-trifluoroprop-1-en-2-yl)benzene, B

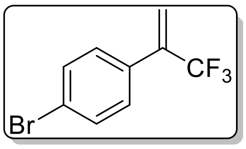

Prepared according to a modified literature procedure[2]

Prepared following **General Procedure 2** from 1-(4-bromophenyl)-2,2,2-trifluoroethan-1-one (1.01 g, 4.00 mmol). Purification by flash column chromatography (10% Et_2O/hexane) affords the title compound **B** (500 mg, 1.99 mmol, 50%) as a colourless oil.

^{1}H-NMR (400 MHz, $CDCl_3$): δ_H 7.56 – 7.51 (2H, m), 7.36 – 7.31 (2H, m), 5.99 (1H, q, *J* = 1.3 Hz), 5.79-5.77 (1H, m).

^{19}F-NMR (377 MHz, $CDCl_3$): δ_F -64.93

^{13}C{^{1}H}-NMR (101 MHz, $CDCl_3$): 138.2 (q, *J* = 30.4 Hz), 132.6, 131.9, 129.1, 123.5, 123.2 (q, *J* = 273.9 Hz), 121.0 (q, *J* = 5.7 Hz)

The spectroscopic data are in agreement with those previously reported[2]

1-Fluoro-4-(3,3,3-trifluoroprop-1-en-2-yl)benzene, C

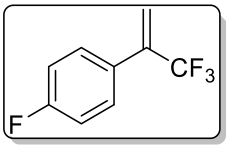

Prepared following **General Procedure 2** from 2,2,2-trifluoro-1-(4-fluorophenyl)ethan-1-one (1.00 g, 5.20 mmol). Purification by flash column chromatography (hexane) affords the title compound **C** (206 mg, 1.08 mmol, 21%) as a colourless oil.

^{1}H-NMR (400 MHz, $CDCl_3$): δ_H 7.46 – 7.40 (2H, m), 7.12 – 7.04 (2H, m), 5.95 (1H, q, *J* = 1.4 Hz), 5.73 (1H, q, *J* = 1.7 Hz).

^{19}F-NMR (377 MHz, $CDCl_3$): δ_F -65.12 (3F, s), -112.46 (1F, s).

^{13}C{^{1}H}-NMR (101 MHz, $CDCl_3$): 163.3 (d, *J* = 248.9 Hz), 138.2 (q, *J* = 30.3 Hz), 129.9 (br. s), 129.5 (d, *J* = 8.3 Hz), 123.3 (q, *J* = 274.0 Hz), 120.7 (m), 115.8 (d, *J* = 21.7 Hz)

The spectroscopic data are in agreement with those previously reported[3]

1-Bromo-3-(3,3,3-trifluoroprop-1-en-2-yl)benzene, D

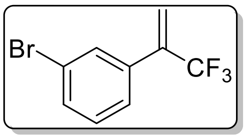

Prepared according to a modified literature procedure[4]

Prepared following **General Procedure 2** from 1-(3-bromophenyl)-2,2,2-trifluoroethan-1-one (1.01 g, 4.00 mmol). Purification by flash column chromatography (10% Et_2O/hexane) affords the title compound **D** (50 mg, 2.17 mmol, 54%) as a yellow oil.

^{1}H-NMR (400 MHz, $CDCl_3$): δ_H 7.60 (1H, s), 7.55 – 7.50 (1H, m), 7.41 – 7.35 (1H, m), 7.26 (1H, t, *J* = 7.9 Hz), 6.00 (1H, d, *J* = 1.4 Hz), 5.78 (1H, d, *J* = 1.7 Hz).

^{19}F-NMR (377 MHz, $CDCl_3$): δ_F -64.90 (s)

^{13}C{^{1}H}-NMR (101 MHz, $CDCl_3$): 138.0 (q, *J* = 30.6 Hz), 135.7, 132.2, 130.6, 130.2, 126.2, 123.2 (q, *J* = 272.7 Hz), 122.8, 121.7 (q, *J* = 5.7 Hz)

The spectroscopic data are in agreement with those previously reported.[5]

1-(Trifluoromethyl)-3-(3,3,3-trifluoroprop-1-en-2-yl)benzene, E

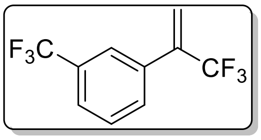

Prepared according to a modified literature procedure[4]

Prepared following **General Procedure 2** from 2,2,2-trifluoro-1-(3-(trifluoromethyl)phenyl)ethan-1-one (630 mg, 2.6 mmol). Purification by flash column chromatography (hexane) affords the title compound **E** (187 mg, 0.78 mmol, 30%) as a colourless oil.

^{1}H-NMR (400 MHz, $CDCl_3$): δ_H 7.66 (3H, m), 7.53 (1H, t, *J* = 7.8 Hz), 6.06 (1H, d, *J* = 1.4 Hz), 5.84 (1H, d, *J* = 1.6 Hz).

^{19}F-NMR (377 MHz, $CDCl_3$): δ_F -62.84 (3F, s), -64.97 (3F, s).

^{13}C{^{1}H}-NMR (101 MHz, $CDCl_3$): 138.2 (q, *J* = 30.6 Hz), 134.6, 131.4 (q, *J* = 32.5 Hz), 130.9, 129.3, 125.9 (q, *J* = 3.8 Hz), 124.5 (q, *J* = 2.4 Hz), 124.0 (q, *J* = 273.7 Hz), 123.1 (q, *J* =274.7 Hz), 122.1 (q, *J* = 5.7 Hz)

The spectroscopic data are in agreement with those previously reported.[6]

1-(3,3,3-Trifluoro-1-phenylprop-1-en-2-yl)-3-(trifluoromethyl)benzene, F

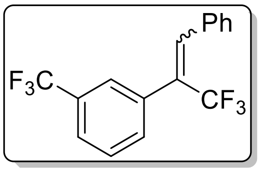

Prepared following **General Procedure 2** from 2,2,2-trifluoro-1-(3-(trifluoromethyl)phenyl)ethan-1-one (1.26 g, 5.20 mmol) and benzyltriphenylphosphonium bromide (2.90 g, 6.50 mmol). Purification by flash column chromatography (5% Et_2O/hexane) affords the title compound **F** (975 mg, 3.08 mmol, 60%) as a colourless oil as a single stereoisomer.

The absolute stereochemistry of the double bond could not be unambigiously determined through 2D-NMR experiments.

^{1}H-NMR (400 MHz, $CDCl_3$): δ_H 7.67 (1H, dtd, *J* = 7.6, 1.8, 0.8 Hz), 7.57 (1H, s), 7.54 – 7.45 (2H, m), 7.34 – 7.30 (1H, m), 7.25 – 7.14 (3H, m), 6.99 – 6.95 (2H, m).

^{19}F-NMR (377 MHz, $CDCl_3$): δ_F-62.80 (3F, s), -65.60 (3F, d, *J* = 1.8 Hz).

^{13}C{^{1}H}-NMR (101 MHz, $CDCl_3$): δ_C 134.6 (q, *J* = 5.8 Hz), 133.7, 133.6 (br. s), 133.0, 131.6 (q, *J* = 32.6 Hz), 130.1, 129.7, 129.5, 129.0 (q, *J* = 29.8 Hz), 128.6, 127.0 (q, *J* = 3.8 Hz), 125.8 (q, *J* = 3.8 Hz), 123.9 (q, *J* = 273.7 Hz), 123.6 (q, *J* = 273.7 Hz)

IR (neat, *v* cm^{-1}): 1323, 1270, 1112, 1073

HRMS: *exact mass not found in ESI+ or ESI- mode*

Ethyl 2-phenylacrylate, G

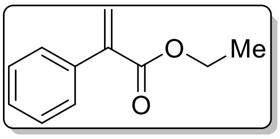

Prepared following **General Procedure 3** from 2-phenylacrylic acid (450 g, 3.00 mmol) and ethanol (0.87 mL, 15 mmol). Purification by flash column chromatography (5% EtOAc /hexane) affords the title compound **G** (211 mg, 1.19 mmol, 40%) as a colourless oil.

^{1}H-NMR (400 MHz, $CDCl_3$): δ_H 7.46 – 7.43 (2H, m), 7.39 – 7.33 (3H, m), 6.36 (1H, d, *J* = 1.3 Hz), 5.90 (1H, d, *J* = 1.3 Hz), 4.31 (2H, q, *J* = 7.1 Hz), 1.34 (3H, t, *J* = 7.1 Hz, 2H).

^{13}C{^{1}H}-NMR (101 MHz, $CDCl_3$): δ_C 166.8, 141.6, 136.8, 128.3, 128.1, 128.1, 126.4, 61.1, 14.2.

The spectroscopic data are in agreement with those previously reported[7]

***N*-(4-(trifluoromethyl)phenyl)acrylamide, H**

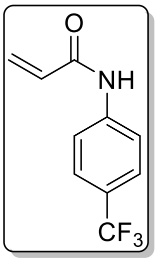

Prepared according to a modified literature procedure[8]

To a solution of 4-(trifluoromethyl)aniline (1.5 g, 10 mmol, 1.0 equiv.) in DCM (15 mL) and acryloyl chloride (0.98 mL, 12 mmol, 1.2 equiv.) cooled to 0 °C, was added triethylamine (1.6 mL, 12 mmol, 1.2 equiv.). The mixture was stirred overnight while allowing it to warm to room temperature, then diluted with DCM (20 mL) and washed with water (2 x 20 mL). The organic layer was dried over Na_2SO_4, filtered, and concentrated under reduced pressure to afford the crude material, which was purified by precipitation from DCM (1.7 g, 8.1 mmol, 81%) to afford the title compound **H** as a white solid.

^{1}H-NMR (400 MHz, $CDCl_3$): δ_H 7.72 (2H, d, *J* = 8.5 Hz), 7.59 (2H, d, *J* = 8.6 Hz), 7.52 (1H, s), 6.47 (1H, dd, *J* = 16.8, 1.2 Hz), 6.27 (1H, dd, *J* = 16.8, 10.2 Hz), 5.83 (1H, dd, *J* = 10.2, 1.2 Hz).

^{19}F-NMR (377 MHz, $CDCl_3$): δ_F-62.19 (s).

^{13}C{^{1}H}-NMR (101 MHz, MeOD-d_4): δ_C 166.3, 143.4, 132.2, 128.6, 127.0 (q, *J* = 3.9 Hz), 126.8 (q, *J* = 32.3 Hz), 125.6 (q, *J* = 266.7 Hz), 120.9

The spectroscopic data are in agreement with those previously reported[9]

Methyl 2-phenylacrylate, K

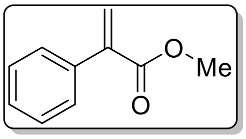

Prepared following **General Procedure 3** from 2-phenylacrylic acid (900 g, 6.07 mmol) and methanol (1.80 mL, 44.50 mmol). The organic phase was concentrated under reduced pressure to afford the title compound **K** (255mg, 1.52 mmol, 25%) as a light-blue oil, which was used without further purification.

^{1}H-NMR (400 MHz, $CDCl_3$): δ_H 7.43 (2H, m), 7.39 – 7.33 (3H, m), 6.38 (1H, d, *J* = 1.0 Hz), 5.91 (1H, d, *J* = 1.0 Hz), 3.83 (3H, s).

^{13}C{^{1}H}-NMR (101 MHz, $CDCl_3$): δ_C 167.3, 141.4, 136.8, 128.4, 128.3, 128.2, 126.9, 52.2.

The spectroscopic data are in agreement with those previously reported[10]

1-Methylazetidin-3-yl 2-phenylacrylate, L

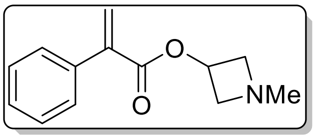

Prepared following **General Procedure 3** from 2-phenylacrylic acid (333 mg, 2.24 mmol) and 1-methylazetidin-3-ol (215 mg, 2.47 mmol). The organic phase was concentrated under reduced pressure to afford the title compound **L** (60 mg, 0.27 mmol, 12%) as a colourless oil, which was used without further purification.

^{1}H-NMR (400 MHz, $CDCl_3$): δ_H 7.45 – 7.38 (2H, m), 7.39 – 7.32 (3H, m), 6.39 (1H, d, *J* = 1.2 Hz), 5.94 (1H, d, *J* = 1.2 Hz), 5.15 (1H, p, *J* = 5.8 Hz), 3.86 – 3.67 (2H, m), 3.16 – 3.02 (2H, m), 2.38 (3H, s).

^{13}C{^{1}H}-NMR (101 MHz, $CDCl_3$): δ_C 166.2, 141.0, 136.6, 128.4, 128.4, 128.3, 127.4, 64.9, 63.0, 46.3.

IR (neat, *v* cm^{-1}): 2934, 2834, 2773, 1719, 1648

HRMS: *exact mass not found in ESI+ or ESI- mode*

1-((2-Bromophenyl)sulfonyl)-1*H*-indole-3-carbaldehyde, AB

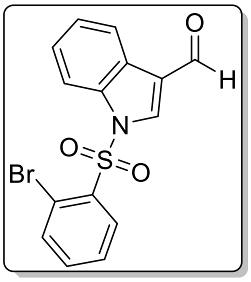

To a solution of 1*H*-indole-3-carbaldehyde (0.72 g, 5.0 mmol, 1.0 equiv.) and 2-bromobenzenesulfonyl chloride (1.40 g, 5.50 mmol, 1.10 equiv.) in dichloromethane (0.5 M) at room temperature was added triethylamine (1.4 mL, 10 mmol, 2.0 equiv.). The reaction mixture was stirred at room temperature overnight, then diluted with dichloromethane and washed twice with water. The organic phase was dried over sodium sulfate, filtered, and concentrated under reduced pressure to afford the title compound **AB** (1.49 g, 4.00 mmol, 80%) as an off-white solid, which was used without further purification.

^{1}H-NMR (400 MHz, $CDCl_3$): δ_H 10.14 (1H, s), 8.48 (1H, s), 8.40 (1H, dd, *J* = 8.0, 1.7 Hz), 8.33 – 8.24 (1H, m), 7.70 (1H, dd, *J* = 7.9, 1.3 Hz), 7.63 – 7.55 (2H, m), 7.49 (1H, td, *J* = 7.7, 1.7 Hz), 7.39 – 7.28 (2H, m).

^{13}C{^{1}H}-NMR (101 MHz, $CDCl_3$): δ_C 185.5, 138.7, 136.7, 136.5, 135.9, 135.0, 132.5, 128.3, 126.4, 126.3, 125.3, 123.0, 121.4, 121.2, 112.8.

IR (neat, *v* cm^{-1}): 3124, 3066, 1674, 1543, 1383

N-(bis(4-chlorophenyl)methylene)-4-methylbenzenesulfonamide, AE

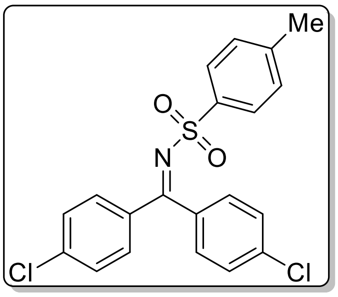

Prepared following **General Procedure 1** from bis(4-chlorophenyl)methanone (1.25 g, 5.00 mmol) and 4-methylbenzenesulfonamide (855 mg, 5.00 mmol). Purification by flash column chromatography (30% Et_2O/hexane) affords the title compound **AE** (710 mg, 1.7 mmol, 35%) as an off-white solid.

^{1}H-NMR (400 MHz, $CDCl_3$): δ_H 7.84 – 7.80 (2H, m), 7.52 – 7.38 (8H, m), 7.31 (2H, d, *J* = 8.1 Hz), 2.45 (3H, s).

^{13}C{^{1}H}-NMR (101 MHz, $CDCl_3$): δ_C 176.3, 143.9, 138.2, 131.5, 129.6, 128.9, 128.8, 127.5, 21.7.

IR (neat, *v* cm^{-1}): 1596, 1578, 1321, 1087,1913.

HRMS: *exact mass not found in ESI+ or ESI- mode*

***N*-((4-Bromophenyl)(phenyl)methylene)-4-methylbenzenesulfonamide, AF**

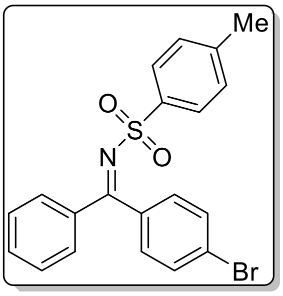

Prepared following **General Procedure 1** from (4-bromophenyl)(phenyl)methanone (1.30 g, 5.00 mmol) and 4-methylbenzenesulfonamide (855 mg, 5.00 mmol). Purification by flash column chromatography (30% Et_2O/hexane) affords the title compound **AF** (620 mg, 1.50 mmol, 30%) as an off-white solid.

^{1}H-NMR (400 MHz, $CDCl_3$): δ_H 7.85 – 7.79 (2H, m), 7.61 – 7.38 (9H, m), 7.30 (2H, d, *J* = 8.1 Hz), 2.44 (3H, s).

^{13}C{^{1}H}-NMR (101 MHz, $CDCl_3$): δ_C 177.6, 143.7, 138.4, 131.6, 129.6, 128.4, 127.5, 21.7.

The spectroscopic data are in agreement with those previously reported[11]

4-Methyl-*N*-(phenyl(4-(*p*-tolylthio)phenyl)methylene)benzenesulfonamide, AG

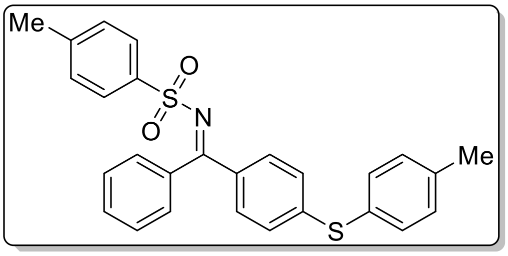

Prepared following **General Procedure 1** from phenyl(4-(*p*-tolylthio)phenyl)methanone (1.52 g, 5.00 mmol) and 4-methylbenzenesulfonamide (855 mg, 5.00 mmol). Purification by flash column chromatography (30% Et_2O/hexane) affords the title compound **AG** (730 mg, 1.60 mmol, 32%) as a yellow solid.

^{1}H-NMR (400 MHz, $CDCl_3$): δ_H 7.82 – 7.78 (2H, m), 7.54 – 7.38 (9H, m), 7.29 – 7.24 (2H, m), 7.22 (2H, d, *J* = 7.9 Hz), 7.07 (2H, d, *J* = 8.2 Hz), 2.42 (3H, s), 2.38 (3H, s).

^{13}C{^{1}H}-NMR (101 MHz, $CDCl_3$): δ_C 178.1, 143.4, 139.6, 138.8, 134.9, 130.7, 129.5, 128.2, 127.7, 127.4, 126.2, 21.7, 21.4.

IR (neat, *v* cm^{-1}): 2922, 1589, 1564, 1319, 1152

HRMS (ESI+): *m/z* calcd for $C_{27}H_{23}NO_2S_2Na$ $[M+Na]^+$ 480.1062, found 480.1027

N-(Di-p-tolylmethylene)-4-methylbenzenesulfonamide, AH

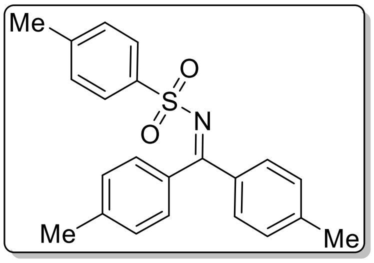

Prepared following **General Procedure 1** from di-*p*-tolylmethanone (1.05 g, 5.00 mmol) and 4-methylbenzenesulfonamide (855 mg, 5.00 mmol). Purification by flash column chromatography (30% Et_2O/hexane) affords the title compound **AH** (690 mg, 1.90 mmol, 38%) as a white solid.

^{1}H-NMR (400 MHz, $CDCl_3$): δ_H 7.87 – 7.81 (2H, m), 7.45 (4H, d, *J* = 7.7 Hz), 7.29 (2H, d, *J* = 8.1 Hz), 7.21 (4H, d, *J* = 7.8 Hz), 2.43 (3H, s), 2.42 (6H, s).

^{13}C{^{1}H}-NMR (101 MHz, $CDCl_3$): δ_C 179.1, 143.2, 139.1, 129.4, 128.9, 127.4, 21.8, 21.7.

IR (neat, *v* cm^{-1}): 3028, 1608, 1580, 1547, 1321, 1152

HRMS (ESI+): *m/z* calcd for $C_{22}H_{21}NO_2SNa$ $[M+Na]^+$ 386.1185, found 386.1201

N-(9H-Fluoren-9-ylidene)-4-methylbenzenesulfonamide, AI

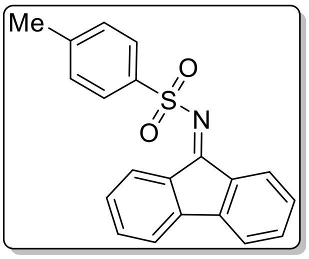

Prepared according to a modified literature procedure[12,13]

Prepared following **General Procedure 1** from 9*H*-fluoren-9-one (900 mg, 5.00 mmol) and 4-methylbenzenesulfonamide (855 mg, 5.00 mmol). Purification by flash column chromatography (30% Et_2O/hexane) affords the title compound **AI** (700 mg, 2.10 mmol, 42%) as a yellow solid.

^{1}H-NMR (400 MHz, $CDCl_3$): δ_H 8.03 – 7.98 (2H, m), 7.50 – 7.42 (5H, m), 7.41 – 7.35 (3H, m), 7.30 – 7.22 (2H, m), 2.48 (3H, s).

^{13}C{^{1}H}-NMR (101 MHz, $CDCl_3$): δ_C 172.6, 143.7, 139.1, 134.9, 129.6, 129.1, 127.3, 120.4, 21.8.

The spectroscopic data are in agreement with those previously reported[13]

N-(Diphenylmethylene)-4-(5-(p-tolyl)-3-(trifluoromethyl)-1H-pyrazol-1-yl)benzenesulfonamide, AJ

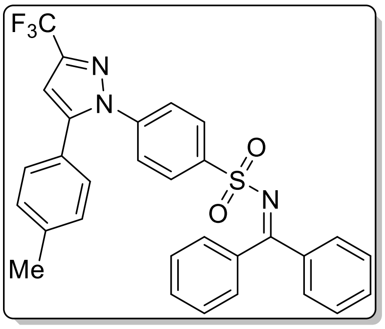

Prepared according to a modified literature procedure[14]

Prepared following **General Procedure 1** from benzophenone (911 mg, 5.00 mmol) and celecoxib (4-(5-(*p*-tolyl)-3-(trifluoromethyl)-1*H*-pyrazol-1-yl)benzenesulfonamide) (1.91 g, 5.00 mmol). Purification by flash column chromatography (20% Et_2O/hexane) affords the title compound **AJ** (680 mg, 1.25 mmol, 25%) as a yellow solid.

^{1}H-NMR (400 MHz, $CDCl_3$): δ_H 8.00 – 7.92 (2H, m), 7.60 – 7.51 (6H, m), 7.50 – 7.41 (6H, m), 7.18 (2H, d, *J* = 8.0 Hz), 7.16 – 7.08 (2H, m), 6.75 (1H, s), 2.38 (3H, s).

^{19}F-NMR (377 MHz, $CDCl_3$): δ_F -62.43 (s)

^{13}C{^{1}H}-NMR (101 MHz, $CDCl_3$): 179.9, 145.4, 144.2 (q, *J* = 38.6 Hz), 142.6, 141.1, 139.9, 129.9, 128.9, 128.5, 128.4, 128.4, 125.9, 125.4, 121.3 (q, *J* = 269.2 Hz), 106.4 (br. s), 21.5

The spectroscopic data are in agreement with those previously reported[14]

Isopropyl 2-(4-((4-chlorophenyl)(((4-(5-(*p*-tolyl)-3-(trifluoromethyl)-1*H*-pyrazol-1-yl)phenyl)sulfonyl)imino)methyl)phenoxy)-2-methylpropanoate, AK

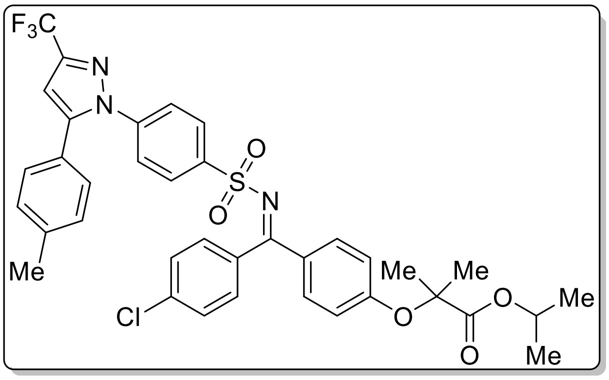

Prepared following **General Procedure 1** from fenofibrate (isopropyl 2-(4-(4-chlorobenzoyl)phenoxy)-2-methylpropanoate (1.80 g, 5.00 mmol) and celecoxib (4-(5-(*p*-tolyl)-3-(trifluoromethyl)-1*H*-pyrazol-1-yl)benzenesulfonamide) (1.91 g, 5.00 mmol). Purification by flash column chromatography (35% to 40% Et_2O/hexane) affords the title compound **AK** (420 mg, 0.58 mmol, 12%) as a yellow solid.

^{1}H-NMR (400 MHz, $CDCl_3$): δ_H 7.96 – 7.91 (2H, m), 7.53 – 7.40 (8H, m), 7.20 – 7.16 (2H, m), 7.15 – 7.10 (2H, m), 6.79 (2H, d, *J* = 8.6 Hz), 6.75 (1H, s), 5.06 (1H, hept, *J* = 6.2 Hz), 2.38 (3H, s), 1.64 (6H, s), 1.20 (3H, s), 1.18 (3H, s).

^{19}F-NMR (377 MHz, $CDCl_3$): δ_F -62.44 (s).

^{13}C{^{1}H}-NMR (101 MHz, $CDCl_3$): 177.7, 172.9, 145.4, 144.2 (q, *J* = 37.4 Hz), 142.6, 141.3, 139.9, 129.9, 128.9, 128.6, 128.4, 125.9, 125.4, 117.4, 106.4 (br. s), 79.8, 69.6, 25.5, 21.7, 21.5

Signal for -$\underline{C}F_3$ not clearly resolved.

IR (neat, *v* cm^{-1}): 2984, 1730, 1543, 1151, 1094

HRMS: *exact mass not found in ESI+ or ESI- mode*

1-Phenyl-1-(trifluoromethyl)spiro[2.3]hexane, 11

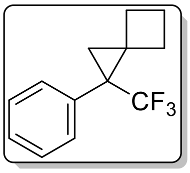

Prepared following **General Procedure 4** from (3,3,3-trifluoroprop-1-en-2-yl)benzene **A** (24 mg, 0.14 mmol) and cyclobutyl(phenyl)(2,4,6-trimethoxyphenyl)sulfonium tetrafluoroborate **8** (75 mg, 0.18 mmol). Work-up affords the crude material with a quantitative ^{1}H-NMR yield of 95%. Purification by flash column chromatography (hexane) affords the title compound **11** (19 mg, 0.084 mmol, 60%) as a colourless oil.

^{1}H-NMR (400 MHz, $CDCl_3$): δ_H 7.43 – 7.27 (5H, m), 2.70 – 2.56 (1H, m), 2.24 – 2.08 (3H, m), 2.00 (1H, m), 1.59 – 1.50 (2H, m), 1.21 (1H, m).

^{19}F-NMR (377 MHz, $CDCl_3$): δ_F -65.43 (s)

^{13}C{^{1}H}-NMR (101 MHz, $CDCl_3$): δ_C 134.1, 131.3, 128.5, 128.1, 126.5 (q, J = 274.9 Hz), 29.9, 28.5 (br. s), 28.2, 21.9 (q, J = 2.8 Hz), 16.7

IR (neat, v cm^{-1}): 2935, 1377, 1341, 1125, 1066.

HRMS: *exact mass not found in ESI+ or ESI- mode*

1-(4-Bromophenyl)-1-(trifluoromethyl)spiro[2.3]hexane, 12

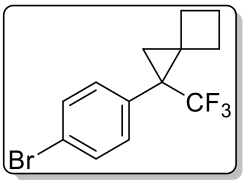

Prepared following **General Procedure 4** from 1-bromo-4-(3,3,3-trifluoroprop-1-en-2-yl)benzene **B** (35 mg, 0.14 mmol) and cyclobutyl(phenyl)(2,4,6-trimethoxyphenyl)sulfonium tetrafluoroborate **8** (75 mg, 0.18 mmol). Work-up affords the crude material with a quantitative ^{1}H-NMR yield of 77%. Purification by flash column chromatography (2% Et_2O/hexane) affords the title compound **12** (20 mg, 0.066 mmol, 47%) as a colourless oil.

^{1}H-NMR (400 MHz, $CDCl_3$): δ_H 7.52 – 7.44 (2H, m), 7.30 – 7.23 (2H, m), 2.60 (1H, m), 2.23 – 2.06 (3H, m), 2.03 – 1.91 (1H, m), 1.62 – 1.50 (2H, m), 1.17 (1H, m).

^{19}F-NMR (377 MHz, $CDCl_3$): δ_F -65.53(s)

^{13}C{^{1}H}-NMR (101 MHz, $CDCl_3$): δ_C 133.1, 133.0, 126.1 (q, *J* = 274.8 Hz), 122.4, 34.1 (q, *J* = 32.1 Hz), 30.0, 28.4, 28.2, 22.0 (q, *J* = 2.7 Hz), 16.7 (d, *J* = 1.0 Hz)

IR (neat, *v* cm^{-1}): 2934, 2856, 1491, 1343, 1153

HRMS: *exact mass not found in ESI+ or ESI- mode*

1-(4-Fluorophenyl)-1-(trifluoromethyl)spiro[2.3]hexane, 13

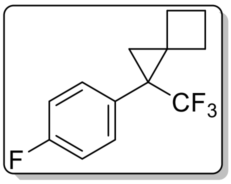

Prepared following **General Procedure 4** from 1-fluoro-4-(3,3,3-trifluoroprop-1-en-2-yl)benzene **C** (27 mg, 0.14 mmol) and cyclobutyl(phenyl)(2,4,6-trimethoxyphenyl)sulfonium tetrafluoroborate **8** (75 mg, 0.18 mmol). Work-up affords the crude material with a quantitative ^{1}H-NMR yield of 63%. Purification by flash column chromatography (hexane) affords the title compound **13** (15 mg, 0.061 mmol, 44%) as a colourless oil.

^{1}H-NMR (400 MHz, $CDCl_3$): δ_H 7.41 – 7.31 (2H, m), 7.09 – 6.96 (2H, m), 2.66 – 2.56 (1H, m), 2.21 – 2.08 (3H, m), 1.99 (1H, m), 1.63 – 1.52 (2H, m), 1.16 (1H, m).

^{19}F-NMR (377 MHz, $CDCl_3$): δ_F -65.73 (s, 3F), -114.00 (s, 1F)

^{13}C{^{1}H}-NMR (101 MHz, $CDCl_3$): δ_C 162.6 (d, *J* = 247.0 Hz), 133.0 (d, *J* = 8.3 Hz), 129.9 (m), 126.3 (qd, *J* = 275.7, 1.01 Hz), 115.5 (d, *J* = 22.2 Hz), 33.8 (q, *J* = 32.1 Hz), 30.0, 28.5 (m), 28.2 (br. s), 22.1 (q, *J* = 2.8 Hz), 16.7 (m)

IR (neat, *v* cm^{-1}): 2956, 1608, 1511, 1343, 1251.

HRMS: *exact mass not found in ESI+ or ESI- mode*

1-(3-Bromophenyl)-1-(trifluoromethyl)spiro[2.3]hexane, 14

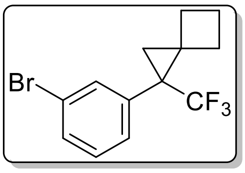

Prepared following **General Procedure 4** from 1-bromo-3-(3,3,3-trifluoroprop-1-en-2-yl)benzene **D** (35 mg, 0.14 mmol) and cyclobutyl(phenyl)(2,4,6-trimethoxyphenyl)sulfonium tetrafluoroborate **8** (75 mg, 0.18 mmol). Work-up affords the crude material with a quantitative ^{1}H-NMR yield of 82%. Purification by flash column chromatography (2% Et_2O/hexane) affords the title compound **14** (21 mg, 0.069 mmol, 49%) as a colourless oil.

^{1}H-NMR (400 MHz, $CDCl_3$): δ_H 7.52 (1H, d, *J* = 2.0 Hz), 7.47 – 7.42 (1H, m), 7.33 (1H, d, *J* = 7.7 Hz), 7.23 (1H, t, *J* = 7.8 Hz), 2.66 – 2.54 (1H, m), 2.23 – 2.07 (3H, m), 2.04 – 1.93 (1H, m), 1.62 – 1.51 (2H, m), 1.20 (1H, m).

^{19}F-NMR (377 MHz, $CDCl_3$): δ_F -65.37 (s)

^{13}C{^{1}H}-NMR (101 MHz, $CDCl_3$): δ_C 136.3, 134.3, 131.4, 130.0, 126.1 (q, *J* = 274.8 Hz), 122.4, 34.2 (q, *J* = 32.2 Hz), 30.1, 28.4, 28.2, 21.9 (q, *J* = 2.6 Hz), 16.7 (d, *J* = 1.0 Hz)

IR (neat, *v* cm^{-1}): 2938, 1595, 1477, 1339, 1123.

HRMS: *exact mass not found in ESI+ or ESI- mode*

1-(Trifluoromethyl)-1-(3-(trifluoromethyl)phenyl)spiro[2.3]hexane, 15

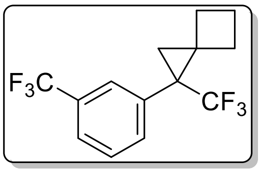

Prepared following **General Procedure 4** from 1-(trifluoromethyl)-3-(3,3,3-trifluoroprop-1-en-2-yl)benzene **E** (34 mg, 0.14 mmol) and cyclobutyl(phenyl)(2,4,6-trimethoxyphenyl)sulfonium tetrafluoroborate **8** (75 mg, 0.18 mmol). Work-up affords the crude material with a quantitative ^{1}H-NMR yield of 57%. Purification by flash column chromatography (hexane) affords the title compound **15** (12 mg, 0.041 mmol, 29%) as a colourless oil.

^{1}H-NMR (400 MHz, $CDCl_3$): δ_H 7.65 – 7.55 (3H, m), 7.48 (1H, app. t, *J* = 7.7 Hz), 2.63 (1H, tdd, *J* = 13.1, 9.1, 5.4 Hz), 2.25 – 1.94 (4H, m), 1.61 – 1.50 (2H, m), 1.29 – 1.15 (1H, m).

^{19}F-NMR (377 MHz, $CDCl_3$): δ_F -62.64 (3F, s), -65.49 (3F, s).

^{13}C{^{1}H}-NMR (101 MHz, $CDCl_3$): δ_C 135.1 (m), 134.8, 131.1 (q, *J* = 32.4 Hz), 129.0, 128.0 (q, *J* = 3.8 Hz), 126.1 (q, *J* = 275.7 Hz), 125.1 (q, *J* = 3.8 Hz), 124.1 (q, *J* = 273.7 Hz), 34.3 (q, *J* = 32.3 Hz), 30.2 (q, *J* = 1.0 Hz), 28.5 (m), 28.2 (br. s), 22.0 (q, *J* = 2.8 Hz), 16.7 (q, *J* = 1.3 Hz)

IR (neat, *v* cm^{-1}): 2935, 1669, 1322, 1157, 1124

HRMS: *exact mass not found in ESI+ or ESI- mode*

2-Phenyl-1-(trifluoromethyl)-1-(3-(trifluoromethyl)phenyl)spiro[2.3]hexane, 16

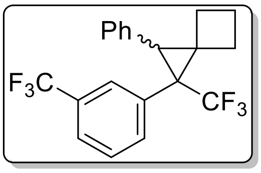

Prepared following a modification of **General Procedure 4** from 1-(3,3,3-trifluoro-1-phenylprop-1-en-2-yl)-3-(trifluoromethyl)benzene **F** (20 mg, 0.06 mmol) and cyclobutyl(phenyl)(2,4,6-trimethoxyphenyl)sulfonium tetrafluoroborate **8** (100 mg, 0.240 mmol, 4.00 equiv.), and LiHMDS (1M in THF, 4.7 equiv.) stirred overnight. Work-up affords the crude material with a quantitative ^{1}H-NMR yield of 85%. Purification by flash column chromatography (hexane) affords the title compound **16** as a single diastereoisomer (15 mg, 0.040 mmol, 67%) as a colourless oil.

^{1}H-NMR (400 MHz, $CDCl_3$): δ_H 7.62 – 7.55 (1H, m), 7.39 (1H, t, *J* = 7.8 Hz), 7.33 (1H, s), 7.25 (1H, d, *J* = 8.2 Hz), 7.20 – 7.09 (3H, m), 6.51 – 6.44 (2H m), 2.79 (1H s), 2.70 (1H, m), 2.50 – 2.42 (1H, m), 2.36 – 2.28 (1H, m), 2.20 (2H, m), 2.07 – 1.92 (1H, m).

^{19}F-NMR (377 MHz, $CDCl_3$): δ_F -62.84 (3F, s), -65.37 (3F, d, *J* = 1.8 Hz).

^{13}C{^{1}H}-NMR (101 MHz, $CDCl_3$): δ_C 137.3, 135.2, 130.8, 130.6 (q, *J* = 3.9 Hz), 130.5, 129.7, 128.6, 127.7, 126.5, 126.0 (q, *J* = 276.7 Hz), 125.2 (q, *J* = 3.8 Hz), 124.0 (q, *J* = 272.7 Hz), 39.5 (q, *J* = 30.9 Hz), 35.5, 35.3 (q, *J* = 3.0 Hz), 29.1, 24.6, 16.5

IR (neat, *v* cm^{-1}): 2945, 1603, 1306, 1122, 1074.

HRMS: *exact mass not found in ESI+ or ESI- mode*

Ethyl 1-phenylspiro[2.3]hexane-1-carboxylate, 17

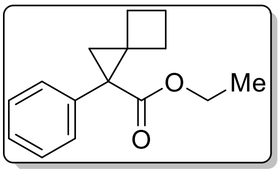

Prepared following a modification of **General Procedure 4** from ethyl 2-phenylacrylate **G** (25 mg, 0.14 mmol) and cyclobutyl(phenyl)(2,4,6-trimethoxyphenyl)sulfonium tetrafluoroborate **8** (150 mg, 0.360 mmol, 2.60 equiv.) and LiHMDS (1M in in THF, 3.2 equiv.) stirred overnight. Work-up affords the crude material with a quantitative ^{1}H-NMR yield of 95%. Purification by flash column chromatography (5% EtOAc/hexane) affords the title compound **17** (20 mg, 0.086 mmol, 62%) as a colourless oil.

^{1}H-NMR (400 MHz, $CDCl_3$): δ_H 7.36 – 7.22 (5H, m), 4.19 – 3.99 (2H, m), 2.45 – 2.33 (1H, m), 2.33 – 2.19 (1H, m), 2.06 (2H, dtd, *J* = 9.0, 7.4, 5.9 Hz), 2.00 – 1.87 (1H, m), 1.86 (1H, d, *J* = 4.7 Hz), 1.64 – 1.52 (1H, m), 1.40 (1H, d, *J* = 4.7 Hz), 1.18 (3H, t, *J* = 7.1 Hz).

^{13}C{^{1}H}-NMR (101 MHz, $CDCl_3$): δ_C 172.5, 137.2, 131.0, 128.0, 126.9, 60.7, 37.4, 36.0, 29.3, 28.4, 26.7, 16.1, 14.5.

IR (neat, *v* cm^{-1}): 2979, 1714, 1241, 1215

HRMS (ESI+): *m/z* calcd for $C_{15}H_{18}O_2Na$ $[M+Na]^+$ 253.1199, found 253.1201

_N_-(4-(Trifluoromethyl)phenyl)spiro[2.3]hexane-1-carboxamide, 18

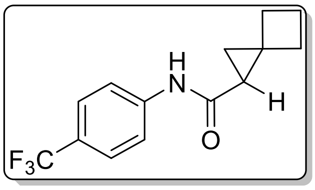

Prepared a modification of following **General Procedure 4** from _N_-(4-(trifluoromethyl)phenyl)acrylamide **H** (40 mg, 0.19 mmol) and cyclobutyl(phenyl)(2,4,6-trimethoxyphenyl)sulfonium tetrafluoroborate **8** (150 mg, 0.36 mmol, 1.9 equiv.) and LiHMDS (1M in THF, 3.4 equiv.). Work-up affords the crude material with a quantitative ^{1}H-NMR yield of 87%. Purification by flash column chromatography (hexane → 1:2:7 Et_2O/DCM/hexane) affords the title compound **18** (8 mg, 0.03 mmol, 16%) as a white solid.

^{1}H-NMR (400 MHz, $CDCl_3$): δ_H 7.65 (2H, d, _J_ = 8.6 Hz), 7.56 (2H, d, _J_ = 8.5 Hz), 7.43 (1H, s), 2.34 – 2.20 (3H, m), 2.18 – 1.97 (3H, m), 1.47 (1H, dd, _J_ = 8.2, 5.2 Hz), 1.34 (1H, t, _J_ = 4.9 Hz), 1.06 (1H, dd, _J_ = 8.2, 4.6 Hz).

^{19}F-NMR (377 MHz, $CDCl_3$): δ_F -62.07 (s).

^{13}C{^{1}H}-NMR (101 MHz, $CDCl_3$): δ_C 170.3, 141.4, 126.4 (q, _J_ = 3.8 Hz), 124.3 (q, _J_ = 271.5 Hz), 119.1 (br. s), 30.9, 30.8, 28.1, 27.7, 20.8, 17.1

IR (neat, _v_ cm^{-1}): 3305, 2933, 1663, 1537, 1323

HRMS (ESI+): _m/z_ calcd for $C_{14}H_{15}F_3NO$ $[M+H]^+$ 270.1105, found 270.1101

1-(Phenylsulfinyl)spiro[2.3]hexane, 19

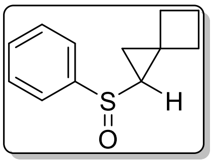

Prepared following **General Procedure 4** from (vinylsulfinyl)benzene **I** (34 mg, 0.19 mmol) and cyclobutyl(phenyl)(2,4,6-trimethoxyphenyl)sulfonium tetrafluoroborate **8** (100 mg, 0.245 mmol). Work-up affords the crude material with a quantitative ^{1}H-NMR yield of 72%. Purification by flash column chromatography (10% EtOAc/DCM) affords the title compound **19** (21 mg, 0.10 mmol, 53%) as a pink solid.

^{1}H-NMR (400 MHz, $CDCl_3$): δ_H 7.72 – 7.63 (2H, m), 7.55 – 7.44 (3H, m), 2.88 – 2.79 (1H, m), 2.37 – 2.27 (2H, m), 2.23 – 2.11 (3H, m), 1.67 (1H, s), 1.10 – 0.98 (2H, m).

^{13}C{^{1}H}-NMR (101 MHz, $CDCl_3$): δ_C 145.1, 130.8, 129.2, 124.2, 45.2, 30.5, 28.5, 27.3, 17.7, 16.9.

IR (neat, *v* cm^{-1}): 3057, 2931, 1442, 1087, 1042.

HRMS (ESI+): *m/z* calcd for $C_{12}H_{14}OSNa$ $[M+Na]^+$ 229.0658, found 229.0653

1-(4-(Trifluoromethyl)phenyl)spiro[2.3]hexane, 20

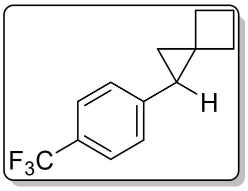

Prepared a modification of following **General Procedure 4** from 1-(trifluoromethyl)-4-vinylbenzene **J** (33 mg, 0.19 mmol) and cyclobutyl(phenyl)(2,4,6-trimethoxyphenyl)sulfonium tetrafluoroborate **8** (300 mg, 0.72 mmol, 3.8 equiv.) and LiHMDS (1M in THF, 4.8 equiv.). Work-up affords the crude material with a quantitative ^{1}H-NMR yield of 94%. Purification by flash column chromatography (hexane) affords the title compound **20** (20 mg, 0.088 mmol, 47%) as a colourless oil.

^{1}H-NMR (400 MHz, $CDCl_3$): δ_H 7.50 (2H, d, *J* = 8.1 Hz), 7.03 (2H, d, *J* = 8.1 Hz), 2.32 – 2.22 (1H, m), 2.21 – 2.01 (3H, m), 1.98 – 1.86 (3H, m), 1.16 (1H, dd, *J* = 8.8, 5.4 Hz), 0.92 (1H, t, *J* = 5.6 Hz).

^{19}F-NMR (377 MHz, $CDCl_3$): δ_F -62.16 (s)

^{13}C{^{1}H}-NMR (101 MHz, $CDCl_3$): δ_C 146.0, 127.3 (q, *J* = 32.3 Hz), 126.8, 124.7 (q, *J* = 272.7 Hz), 125.0 (q, *J* = 3.8 Hz), 31.5, 29.8, 28.3, 26.5, 21.5, 16.6

IR (neat, *v* cm^{-1}): 2924, 2853, 1619, 1462, 1325

HRMS: *exact mass not found in ESI+ or ESI- mode*

2-Phenyl-2-(4-(trifluoromethyl)phenyl)-1-oxaspiro[2.3]hexane, 23

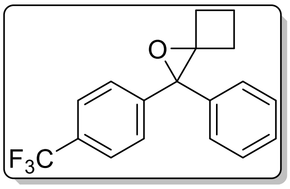

Prepared following **General Procedure 4** from phenyl(4-(trifluoromethyl)phenyl)methanone **M** (24 mg, 0.095 mmol) and cyclobutyl(phenyl)(2,4,6-trimethoxyphenyl)sulfonium tetrafluoroborate **8** (50 mg, 0.12 mmol). Work-up affords the crude material with a quantitative ^{1}H-NMR yield of 94%. Purification by flash column chromatography (0.5-1% Et_2O/hexane) affords the title compound **23** (16 mg, 0.053 mmol, 55%) as a colourless oil.

^{1}H-NMR (400 MHz, $CDCl_3$): δ_H 7.62 – 7.57 (2H, m), 7.46 (2H, d, *J* = 8.1 Hz), 7.41 – 7.29 (5H, m), 2.50 – 2.40 (2H, m), 2.20 (1H, m), 2.13 – 2.04 (1H, m), 1.92 (1H, dtt, *J* = 11.3, 10.3, 4.1 Hz), 1.74 (1H, app. dp, *J* = 11.3, 8.9 Hz).

^{19}F-NMR (377 MHz, $CDCl_3$): δ_F -62.54 (s).

^{13}C{^{1}H}-NMR (101 MHz, $CDCl_3$): δ_C 142.7, 137.6, 129.8 (q, *J* = 32.4 Hz), 128.5, 128.0, 127.7, 125.2 (q, *J* = 3.8 Hz), 124.3 (q, *J* = 272.7 Hz), 71.8, 68.0, 30.2, 30.0, 12.6

IR (neat, *v* cm^{-1}): 2939, 1620, 1322, 1066, 1017

HRMS: *exact mass not found in ESI+ or ESI- mode*

2,2-Bis(4-fluorophenyl)-1-oxaspiro[2.3]hexane, 24

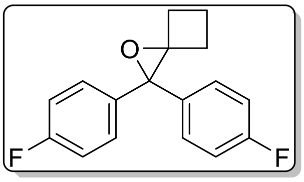

Prepared following **General Procedure 4** from bis(4-fluorophenyl)methanone **N** (30.5 mg, 0.14 mmol) and cyclobutyl(phenyl)(2,4,6-trimethoxyphenyl)sulfonium tetrafluoroborate **8** (75 mg, 0.18 mmol). Work-up affords the crude material with a quantitative ^{1}H-NMR yield of 93%. Purification by flash column chromatography (0.25% Et_2O/hexane) affords the title compound **24** (22 mg, 0.081 mmol, 58%) as a colourless oil.

^{1}H-NMR (400 MHz, $CDCl_3$): δ_H 7.31 – 7.25 (4H, m), 7.07 – 7.00 (4H, m), 2.48 – 2.38 (2H, m), 2.13 (2H, m), 1.91 (1H, dtt, *J* = 11.2, 10.2, 4.1 Hz), 1.72 (1H, app. dp, *J* = 11.2, 8.8 Hz).

^{19}F-NMR (377 MHz, $CDCl_3$): δ_F -114.55 (s).

^{13}C{^{1}H}-NMR (101 MHz, $CDCl_3$): δ_C 162.3 (d, *J* = 246.5 Hz), 134.2 (d, *J* = 3.2 Hz), 129.4 (d, *J* = 8.2 Hz), 115.3 (d, *J* = 21.6 Hz), 71.6, 67.5, 30.1, 12.6

IR (neat, *v* cm^{-1}): 2937, 1605, 1507, 1221, 1155

HRMS (ESI-): *m/z* calcd for $C_{17}H_{13}F_2O$ [M-H]$^-$ 271.0940, found 271.0960

2,2-Bis(4-chlorophenyl)-1-oxaspiro[2.3]hexane, 25

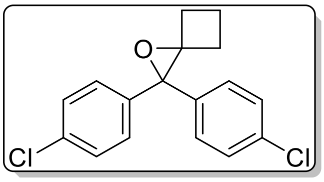

Prepared following **General Procedure 4** from bis(4-chlorophenyl)methanone **O** (36 mg, 0.14 mmol) and cyclobutyl(phenyl)(2,4,6-trimethoxyphenyl)sulfonium tetrafluoroborate **8** (75 mg, 0.18 mmol). Work-up affords the crude material with a quantitative ^{1}H-NMR yield of 87%. Purification by flash column chromatography (0.5% Et_2O/hexane) affords the title compound **25** (29 mg, 0.095 mmol, 68%) as a colourless oil.

^{1}H-NMR (400 MHz, $CDCl_3$): δ_H 7.36 – 7.29 (4H, m), 7.26 – 7.22 (4H, m), 2.47 – 2.37 (2H, m), 2.12 (2H, dddd, *J* = 12.2, 9.1, 4.1, 3.1 Hz), 1.91 (1H, dtt, *J* = 11.2, 10.3, 4.1 Hz), 1.72 (1H, app. dp, *J* = 11.3, 8.8 Hz).

^{13}C{^{1}H}-NMR (101 MHz, $CDCl_3$): δ_C 136.6, 133.8, 129.0, 128.6, 71.8, 67.4, 30.0, 12.5.

IR (neat, *v* cm^{-1}): 2990, 1596, 1490, 1091, 1013

HRMS (ESI-): *m/z* calcd for $C_{17}H_{13}{}^{35}Cl_2O$ [M-H]$^-$ 303.0349, found 303.0346

2-(2-Chlorophenyl)-2-phenyl-1-oxaspiro[2.3]hexane, 26

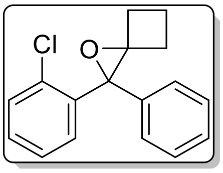

Prepared following **General Procedure 4** from (2-chlorophenyl)(phenyl)methanone **P** (31 mg, 0.14 mmol) and cyclobutyl(phenyl)(2,4,6-trimethoxyphenyl)sulfonium tetrafluoroborate **8** (75 mg, 0.18 mmol). Work-up affords the crude material with a quantitative ^{1}H-NMR yield of 78%. Purification by flash column chromatography (1% Et_2O /hexane) affords the title compound **26** (20 mg, 0.070 mmol, 52%) as a colourless oil.

^{1}H-NMR (400 MHz, $CDCl_3$): δ_H 7.52 – 7.43 (2H, m), 7.34 – 7.23 (5H, m), 7.19 – 7.15 (2H, m), 2.65 (1H, m), 2.40 – 2.26 (2H, m), 2.13 (1H, m), 1.95 – 1.76 (2H, m).

^{13}C{^{1}H}-NMR (101 MHz, $CDCl_3$): δ_C 137.3, 137.2, 133.6, 130.6, 129.3, 129.3, 128.0, 127.7, 127.6, 127.5, 72.1, 67.6, 30.8, 29.6, 13.1

IR (neat, ν cm^{-1}): 3061, 2990, 1295, 1058, 756.

HRMS (ESI+): *m/z* calcd for $C_{17}H_{15}{}^{35}ClONa$ $[M+Na]^+$ 293.0709, found 293.0745

2-(4-Bromophenyl)-2-phenyl-1-oxaspiro[2.3]hexane, 27

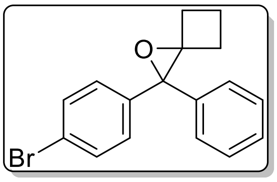

Prepared following **General Procedure 4** from (4-bromophenyl)(phenyl)methanone **Q** (25 mg, 0.095 mmol) and cyclobutyl(phenyl)(2,4,6-trimethoxyphenyl)sulfonium tetrafluoroborate **8** (50 mg, 0.12 mmol). Work-up affords the crude material with a quantitative ^{1}H-NMR yield of 86%. Purification by flash column chromatography (1% Et_2O/hexane) affords the title compound **27** (17 mg, 0.054 mmol, 57%) as a colourless oil.

^{1}H-NMR (400 MHz, $CDCl_3$): δ_H 7.49 – 7.45 (2H, m), 7.39 – 7.28 (5H, m,), 7.23 – 7.19 (2H, m), 2.43 (2H, m), 2.21 – 2.07 (2H, m), 1.90 (1H, dtt, *J* = 11.3, 10.3, 4.1 Hz), 1.72 (1H, app. dp, *J* = 11.3, 8.9 Hz).

^{13}C{^{1}H}-NMR (101 MHz, $CDCl_3$): δ_C 137.9, 137.7, 131.4, 129.4, 128.4, 127.8, 127.7, 121.7, 71.6, 68.0, 30.2, 30.0, 12.6.

IR (neat, *v* cm^{-1}): 3060, 2988, 2935, 1486, 1447

HRMS (ESI+): *m/z* calcd for $C_{17}H_{15}{}^{79}BrONa$ $[M+Na]^+$ 337.0198, found 337.0195

2-(3-Bromophenyl)-2-phenyl-1-oxaspiro[2.3]hexane, 28

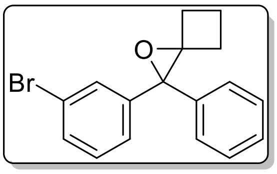

Prepared following **General Procedure 4** from (3-bromophenyl)(phenyl)methanone **R** (27 mg, 0.095 mmol) and cyclobutyl(phenyl)(2,4,6-trimethoxyphenyl)sulfonium tetrafluoroborate **8** (50 mg, 0.12 mmol). Work-up affords the crude material with a quantitative ^{1}H-NMR yield of 84%. Purification by flash column chromatography (1% Et_2O/hexane) affords the title compound **28** (19 mg, 0.060 mmol, 63%) as a colourless oil.

^{1}H-NMR (400 MHz, $CDCl_3$): δ_H 7.49 (1H, t, *J* = 1.8 Hz), 7.45 – 7.24 (7H, m), 7.21 (1H, t, *J* = 7.8 Hz), 2.49 – 2.35 (2H, m), 2.14 (2H, m), 1.95 – 1.85 (1H, m), 1.80 – 1.67 (1H, m).

^{13}C{^{1}H}-NMR (101 MHz, $CDCl_3$): δ_C 141.0, 137.8, 130.8, 130.7, 129.8, 128.4, 127.9, 127.7, 126.3, 122.5, 71.7, 67.8, 30.2, 30.0, 12.6.

IR (neat, *v* cm^{-1}): 3061, 2932, 1593, 1473, 1073.

HRMS (ESI+): *m/z* calcd for $C_{17}H_{15}{}^{79}BrONa$ $[M+Na]^+$ 337.0198, found 337.0191

2-Phenyl-2-(4-(phenylethynyl)phenyl)-1-oxaspiro[2.3]hexane, 29

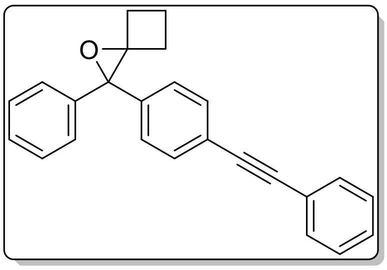

Prepared following **General Procedure 4** from phenyl(4-(phenylethynyl)phenyl)methanone **S** (54 mg, 0.19 mmol) and cyclobutyl(phenyl)(2,4,6-trimethoxyphenyl)sulfonium tetrafluoroborate **8** (100 mg, 0.250 mmol). Work-up affords the crude material with a quantitative ^{1}H-NMR yield of 89%. Purification by flash column chromatography (2% Et_2O/hexane) affords the title compound **29** (53 mg, 0.16 mmol, 83%) as a white solid.

^{1}H-NMR (400 MHz, $CDCl_3$): δ_H 7.56 – 7.50 (4H, m), 7.41 – 7.30 (10H, m), 2.51 – 2.40 (2H, m), 2.23 – 2.10 (2H, m), 1.91 (1H, dtt, *J* = 11.2, 10.3, 4.0 Hz), 1.74 (1H, app. dp, *J* = 11.3, 8.9 Hz).

^{13}C{^{1}H}-NMR (101 MHz, $CDCl_3$): δ_C 138.8, 138.0, 131.7, 131.5, 128.5, 128.4, 128.3, 127.8, 127.7, 127.7, 123.3, 122.5, 89.9, 89.3, 71.7, 68.2, 30.3, 30.1, 12.6.

IR (neat, *v* cm^{-1}): 3059, 2988, 1596, 1513, 1443.

HRMS (ESI+): *m/z* calcd for $C_{25}H_{20}ONa$ $[M+Na]^+$ 359.1406, found 359.1421

2-Phenyl-2-(4-(*p*-tolylthio)phenyl)-1-oxaspiro[2.3]hexane, 30

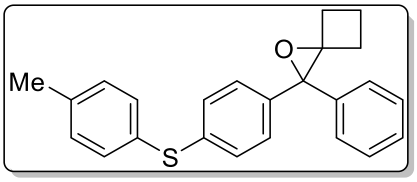

Prepared following **General Procedure 4** from phenyl(4-(*p*-tolylthio)phenyl)methanone **T** (58 mg, 0.19 mmol) and cyclobutyl(phenyl)(2,4,6-trimethoxyphenyl)sulfonium tetrafluoroborate **8** (100 mg, 0.245 mmol). Work-up affords the crude material with a quantitative ^{1}H-NMR yield of 92%. Purification by flash column chromatography (2% Et_2O/hexane) affords the title compound **30** (55 mg, 0.15 mmol, 81%) as a colourless oil.

^{1}H-NMR (400 MHz, $CDCl_3$): δ_H 7.40 – 7.25 (7H, m), 7.21 (4H, d, *J* = 1.0 Hz), 7.16 – 7.07 (2H, m), 2.50 – 2.36 (2H, m), 2.35 (3H, s), 2.19 – 2.09 (2H, m), 1.95 – 1.82 (1H, m), 1.72 (1H, m).

^{13}C{^{1}H}-NMR (101 MHz, $CDCl_3$): δ_C 138.2, 138.0, 136.8, 132.8, 130.8, 130.3, 129.1, 128.3, 128.3, 127.7, 127.7, 71.6, 68.1, 30.2, 30.1, 21.3, 12.6

IR (neat, *v* cm^{-1}): 3025, 2931, 1490, 1446, 1397.

HRMS (ESI-): *m/z* calcd for $C_{24}H_{21}OS$ $[M-H]^-$ 357.1319, found 357.1296

Cyclopenta-2,4-dien-1-yl(2'-oxo-1'-phenyl-[1,1'-bi(cyclopentane)]-3,5-dien-2-yl)iron, 32

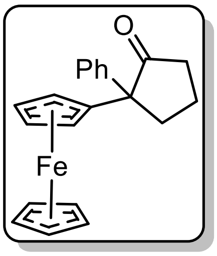

Prepared following a modification of **General Procedure 4** from (2-benzoylcyclopenta-2,4-dien-1-yl)(cyclopenta-2,4-dien-1-yl)iron **U** (40 mg, 0.14 mmol) and cyclobutyl(phenyl)(2,4,6-trimethoxyphenyl)sulfonium tetrafluoroborate **8** (150 mg, 0.360 mmol, 2.6 equiv.), and LiHMDS (1M in THF, 3.2 equiv.) and stirred overnight to obtain the cyclopenta-2,4-dien-1-yl(2-(2-phenyl-1-oxaspiro[2.3]hexan-2-yl)cyclopentan-1-yl)iron **31** as crude material (a copy of the spectrum is included in the NMR spectra section) with a quantitative ^{1}H NMR yield of 67%. The crude product could not be purified by flash column chromatography on SiO_2 due to its instability, as it underwent partial Meinwald rearrangement. This rearrangement could not be prevented even when silica was deactivated with triethylamine. Purification by flash column chromatography (10% Et_2O/hexane, 1% Et_3N) affords the title compound **32** (25 mg, 0.074 mmol, 53%) as an orange solid.

^{1}H-NMR (400 MHz, $CDCl_3$): δ_H 7.33 – 7.24 (4H, m), 7.21 – 7.16 (1H, m), 4.41 (1H, dt, *J* = 2.6, 1.4 Hz), 4.16 (1H, td, *J* = 2.5, 1.3 Hz), 4.12 (1H, td, *J* = 2.5, 1.4 Hz), 4.09 (3H, s), 3.92 (1H, dt, *J* = 2.6, 1.4 Hz), 2.76 – 2.71 (2H, m), 2.51 – 2.46 (2H, m), 2.16 – 2.06 (1H, m), 1.93 – 1.78 (1H, m).

^{13}C{^{1}H}-NMR (101 MHz, $CDCl_3$): δ_C 217.1, 142.4, 128.5, 126.9, 126.8, 92.5, 68.8, 68.5, 67.5, 67.3, 67.3, 56.4, 38.9, 37.9, 19.3.

IR (neat, *v* cm^{-1}): 3081, 2925, 1736, 1492

HRMS: *exact mass not found in ESI+ or ESI- mode*

Isopropyl 2-(4-(2-(4-chlorophenyl)-1-oxaspiro[2.3]hexan-2-yl)phenoxy)-2-methylpropanoate, 33

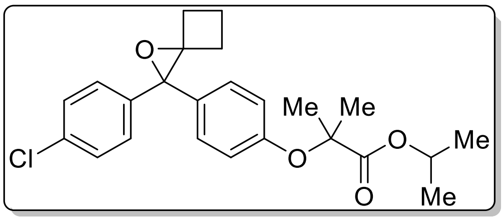

Prepared following **General Procedure 4** from fenofibrate (isopropyl 2-(4-(4-chlorobenzoyl)phenoxy)-2-methylpropanoate) **V** (51 mg, 0.14 mmol) and cyclobutyl(phenyl)(2,4,6-trimethoxyphenyl)sulfonium tetrafluoroborate **8** (75 mg, 0.18 mmol). Work-up affords the crude material with a quantitative ^{1}H-NMR yield of 84%. Purification by flash column chromatography (15% Et_2O/hexane) affords the title compound **33** (31 mg, 0.075 mmol, 53%) as a colourless oil.

^{1}H-NMR (400 MHz, $CDCl_3$): δ_H 7.32 – 7.28 (2H, m), 7.26 – 7.21 (2H, m), 7.17 – 7.11 (2H, m), 6.83 – 6.79 (2H, m), 5.06 (1H, hept, *J* = 6.3 Hz), 2.40 (2H, m), 2.21 – 2.12 (1H, m), 2.07 (1H, m), 1.94 – 1.83 (1H, m), 1.77 – 1.63 (1H, m), 1.58 (6H, s), 1.19 (6H, d, *J* = 6.3 Hz).

^{13}C{^{1}H}-NMR (101 MHz, $CDCl_3$): δ_C 173.7, 155.3, 137.4, 133.4, 131.3, 129.1, 128.5, 128.4, 118.6, 79.3, 71.6, 69.1, 67.6, 30.1, 30.1, 25.6, 25.5, 21.7, 12.5.

IR (neat, *v* cm^{-1}): 2982, 1728, 1508, 1102, 1013.

HRMS (ESI+): *m/z* calcd for $C_{24}H_{27}{}^{35}ClO_4Na$ $[M+Na]^+$ 437.1490, found 437.1505

2-(4-Chlorophenyl)-1-oxaspiro[2.3]hexane, 34

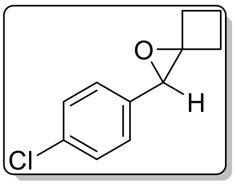

Prepared following **General Procedure 4** from 4-chlorobenzaldehyde **W** (27 mg, 0.19 mmol) and cyclobutyl(phenyl)(2,4,6-trimethoxyphenyl)sulfonium tetrafluoroborate **8** (100 mg, 0.25 mmol). Work-up affords the crude material with a quantitative ^{1}H-NMR yield of 37%. Purification by flash column chromatography (3% Et_2O/hexane) affords the title compound **34** (9 mg, 0.05 mmol, 24%) as a colourless oil.

^{1}H-NMR (400 MHz, $CDCl_3$): δ_H 7.33 – 7.29 (2H, m), 7.13 – 7.09 (2H, m), 3.82 (1H, s), 2.67 – 2.57 (1H, m), 2.51 – 2.37 (2H, m), 1.99 – 1.84 (2H, m), 1.75 – 1.62 (1H, m)

^{13}C{^{1}H}-NMR (101 MHz, $CDCl_3$): δ_C 135.4, 133.7, 128.5, 127.6, 66.9, 61.9, 31.5, 28.4, 12.6

IR (neat, *v* cm^{-1}): 2969, 2933, 1493, 1089, 1014.

HRMS (ESI+): *m/z* calcd for $C_{11}H_{11}{}^{35}ClONa$ $[M+Na]^+$ 217.0391, found 217.0377.

2-(2,4-Dichlorophenyl)-1-oxaspiro[2.3]hexane, 35

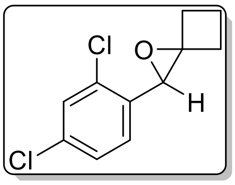

Prepared following **General Procedure 4** from 2,4-dichlorobenzaldehyde **X** (33 mg, 0.19 mmol) and cyclobutyl(phenyl)(2,4,6-trimethoxyphenyl)sulfonium tetrafluoroborate **8** (100 mg, 0.25 mmol). Work-up affords the crude material with a quantitative ^{1}H-NMR yield of 21% Purification by flash column chromatography (2% Et_2O/hexane) affords the title compound **35** (7 mg, 0.03 mmol, 16%) as a colourless oil.

^{1}H-NMR (400 MHz, $CDCl_3$): δ_H 7.39 (1H, d, *J* = 2.0 Hz), 7.22 (1H, dd, *J* = 8.3, 2.0 Hz), 7.05 (1H, d, *J* = 8.3 Hz), 4.08 (1H, s), 2.65 (1H, ddd, *J* = 12.7, 10.2, 7.6 Hz), 2.52 – 2.43 (1H, m), 2.41 – 2.28 (1H, m), 1.88 (1H, dddd, *J* = 15.6, 9.0, 6.3, 4.0 Hz,), 1.82 – 1.69 (2H, m).

^{13}C{^{1}H}-NMR (101 MHz, $CDCl_3$): δ_C 133.9, 133.7, 133.6, 128.9, 128.1, 127.2, 66.6, 59.9, 31.5, 28.4, 12.9.

IR (neat, *v* cm^{-1}): 2991, 2936, 1591, 1478, 1378

HRMS (ESI+): *m/z* calcd for $C_{11}H_{10}{}^{35}Cl{}^{37}ClONa$ $[M+Na]^+$ 252.9973, found 252.9995

2-(10-Chloroanthracen-9-yl)-1-oxaspiro[2.3]hexane, 36

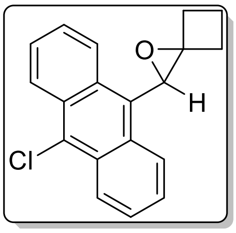

Prepared following a modification of **General Procedure 4** from 10-chloroanthracene-9-carbaldehyde **Y** (34 mg, 0.14 mmol) and cyclobutyl(phenyl)(2,4,6-trimethoxyphenyl)sulfonium tetrafluoroborate **8** (150 mg, 0.360 mmol, 2.6 equiv.). Work-up affords the crude material with a quantitative ^{1}H-NMR yield of 85%. Purification by flash column chromatography on SiO_2 deactivated with triethylamine (1%) (5% Et_2O /hexane, 1% triethylamine) affords the title compound **36** (24 mg, 0.081 mmol, 58%) as a yellow solid.

^{1}H-NMR (400 MHz, $CDCl_3$): δ_H 8.61 – 8.42 (3H, m), 7.66 – 7.54 (5H, m), 4.64 (1H, s), 2.93 – 2.76 (2H, m), 2.19 (1H, dddd, *J* = 13.0, 9.7, 8.6, 1.2 Hz), 2.05 – 1.87 (2H, m), 1.66 – 1.57 (1H, m).

^{13}C{^{1}H}-NMR (101 MHz, $CDCl_3$): δ_C 130.6, 129.7, 128.6, 127.6, 126.7, 126.2, 125.5, 65.3, 60.6, 31.3, 29.5, 13.5.

IR (neat, *v* cm^{-1}): 2962, 1622, 1441, 1262, 1025

HRMS (ESI+): *m/z* calcd for $C_{19}H_{15}{}^{35}ClONa$ $[M+Na]^+$ 317.0709, found 317.0748.

2-(Naphthalen-1-yl)-1-oxaspiro[2.3]hexane, 37

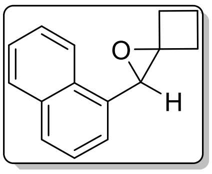

Prepared following **General Procedure 4** from 1-naphthaldehyde **Z** (30 mg, 0.19 mmol) and cyclobutyl(phenyl)(2,4,6-trimethoxyphenyl)sulfonium tetrafluoroborate **8** (100 mg, 0.25 mmol). Work-up affords the crude material with a quantitative ^{1}H-NMR yield of 49%. Purification by flash column chromatography (3% Et_2O /hexane) affords the title compound **37** (12 mg, 0.06 mmol, 30%) as a colourless oil.

^{1}H-NMR (400 MHz, $CDCl_3$): δ_H 8.07 (1H, m), 7.94 – 7.87 (1H, m), 7.79 (1H, d, *J* = 8.2 Hz), 7.55 (2H, m), 7.46 (1H, dd, *J* = 8.2, 7.1 Hz), 7.35 (1H, app. dt, *J* = 7.1, 1.1 Hz), 4.46 (1H, s), 2.80 – 2.69 (1H, m), 2.66 – 2.56 (1H, m), 2.34 (1H, m), 1.94 – 1.84 (1H, m), 1.80 – 1.63 (2H, m).

^{13}C{^{1}H}-NMR (101 MHz, $CDCl_3$): δ_C 133.4, 132.7, 131.4, 129.0, 127.8, 126.4, 125.9, 125.6, 123.0, 122.7, 66.7, 60.8, 31.7, 28.6, 13.0.

IR (neat, *v* cm^{-1}): 3057, 2934, 1595, 1509, 1308

HRMS (ESI-): *m/z* calcd for $C_{15}H_{13}O$ $[M-H]^-$ 209.0972, found 209.0965.

2-(Pyren-1-yl)-1-oxaspiro[2.3]hexane, 38

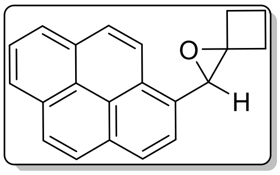

Prepared following a modification of **General Procedure 4** from pyrene-1-carbaldehyde **AA** (32 mg, 0.14 mmol) and cyclobutyl(phenyl)(2,4,6-trimethoxyphenyl)sulfonium tetrafluoroborate **8** (150 mg, 0.360 mmol, 2.6 equiv.). Work-up affords the crude material with a quantitative ^{1}H-NMR yield of 83%. Purification by flash column chromatography on SiO_2 deactivated with triethylamine (1%) (5% Et_2O/hexane, 1% triethylamine) affords the title compound **38** (24 mg, 0.084 mmol, 60%) as a colourless oil.

^{1}H-NMR (400 MHz, $CDCl_3$): δ_H 8.33 (1H, d, *J* = 9.2 Hz), 8.25 – 8.15 (4H, m), 8.08 – 8.00 (3H, m), 7.88 (1H, dd, *J* = 8.0, 0.7 Hz), 4.76 (1H, s), 2.87 – 2.78 (1H, m), 2.70 (1H, m), 2.41 (1H, m), 2.00 – 1.86 (1H, m), 1.85 – 1.74 (1H, m), 1.69 (1H, m).

^{13}C{^{1}H}-NMR (101 MHz, $CDCl_3$): δ_C 131.5, 130.9, 130.8, 130.4, 128.7, 128.1, 127.7, 127.4, 126.2, 125.5, 125.3, 124.9, 124.9, 124.6, 123.3, 122.2, 67.0, 61.2, 31.8, 28.8, 13.1.

IR (neat, *v* cm^{-1}): 3041, 2931, 1915, 1604, 1422

HRMS (ESI-): *m/z* calcd for $C_{21}H_{16}O$ $[M-H]^-$ 283.1128, found 283.1128

2,2-Bis(4-chlorophenyl)-1-tosyl-1-azaspiro[2.3]hexane, 39

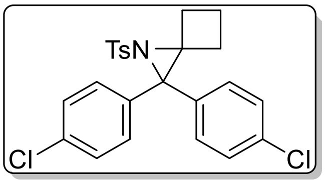

Prepared following **General Procedure 4** from *N*-(bis(4-chlorophenyl)methylene)-4-methylbenzenesulfonamide **AE** (56 mg, 0.14 mmol) and cyclobutyl(phenyl)(2,4,6-trimethoxyphenyl)sulfonium tetrafluoroborate **8** (75 mg, 0.18 mmol). Work-up affords the crude material with a quantitative ^{1}H-NMR yield of 42%. Purification by flash column chromatography (5% EtOAc/hexane) affords the title compound **39** (19 mg, 0.041 mmol, 30%) as an off-white solid.

^{1}H-NMR (400 MHz, $CDCl_3$): δ_H 7.91 (2H, d, *J* = 8.2 Hz), 7.40 – 7.26 (10, m), 2.55 – 2.39 (5H, m), 2.29 (2H, ddd, *J* = 10.5, 8.2, 4.3 Hz,), 1.90 (1H, m), 1.84 – 1.73 (1H m).

^{13}C{^{1}H}-NMR (101 MHz, $CDCl_3$): δ_C 144.4, 138.4, 136.0, 134.2, 130.2, 129.8, 128.7, 127.8, 60.1, 58.3, 29.0, 21.8, 15.5.

IR (neat, *v* cm^{-1}): 2952, 1596, 1489, 1323, 1156

HRMS (ESI+): *m/z* calcd for $C_{24}H_{21}{}^{35}Cl_2NO_2SNa$ $[M+Na]^+$ 480.0562, found 480.0583

2-(4-Bromophenyl)-2-phenyl-1-tosyl-1-azaspiro[2.3]hexane, 40

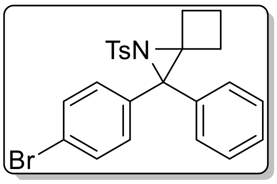

Prepared following **General Procedure 4** from *N*-((4-bromophenyl)(phenyl)methylene)-4-methylbenzenesulfonamide **AF** (40 mg, 0.095 mmol) and cyclobutyl(phenyl)(2,4,6-trimethoxyphenyl)sulfonium tetrafluoroborate **8** (50 mg, 0.12 mmol). Work-up affords the crude material with a quantitative ^{1}H-NMR yield of 59%. Purification by flash column chromatography (2.5-5% Et_2O/hexane) affords the title compound **40** (13 mg, 0.027 mmol, 29%) as an off-white solid.

^{1}H-NMR (400 MHz, $CDCl_3$): δ_H 7.92 (2H, d, *J* = 8.3 Hz), 7.46 – 7.39 (4H, m), 7.36 – 7.27 (7H, m), 2.53 – 2.35 (5H, m), 2.39 – 2.25 (2H, m), 1.99 – 1.89 (1H, m), 1.84 – 1.73 (1H, m).

^{13}C{^{1}H}-NMR (101 MHz, $CDCl_3$): δ_C 144.2, 138.6, 137.3, 137.1, 131.5, 130.5, 129.8, 128.7, 128.5, 128.1, 127.9, 122.1, 61.0, 58.2, 29.1, 29.0, 21.8, 15.5.

IR (neat, *v* cm^{-1}): 2952, 1597, 1486, 1321, 1155.

HRMS (ESI+): *m/z* calcd for $C_{24}H_{22}{}^{81}BrNO_2SNa$ $[M+Na]^+$ 492.0429, found 492.0468

2-Phenyl-2-(4-(*p*-tolylthio)phenyl)-1-tosyl-1-azaspiro[2.3]hexane, 41

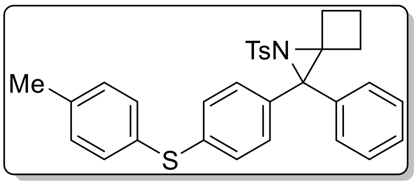

Prepared following **General Procedure 4** from 4-methyl-*N*-(phenyl(4-(*p*-tolylthio)phenyl)methylene)benzenesulfonamide **AG** (55 mg, 0.095 mmol) and cyclobutyl(phenyl)(2,4,6-trimethoxyphenyl)sulfonium tetrafluoroborate **8** (50 mg, 0.12 mmol). Work-up affords the crude material with a quantitative ^{1}H-NMR yield of 63%. Purification by flash column chromatography (10% Et_2O/hexane) affords the title compound **41** (22 mg, 0.043 mmol, 45%) as an off-white solid.

^{1}H-NMR (400 MHz, $CDCl_3$): δ_H 7.91 (2H, d, *J* = 8.0 Hz), 7.41 (2H, d, *J* = 7.1 Hz), 7.37 – 7.24 (9H, m), 7.14 (4H, dd, *J* = 8.0, 5.8 Hz), 2.50 – 2.38 (5H, m), 2.37 – 2.18 (5H, m), 1.89 (1H, dt, *J* = 10.9, 8.6 Hz), 1.77 (1H, m).

^{13}C{^{1}H}-NMR (101 MHz, $CDCl_3$): δ_C 144.1, 138.7, 138.3, 137.5, 135.9, 133.3, 130.3, 130.2, 129.7, 129.4, 128.8, 128.5, 128.4, 127.9, 127.9, 61.4, 58.2 , 29.1, 29.1, 21.8, 21.3, 15.5.

IR (neat, *v* cm^{-1}): 2923, 1596, 1490, 1322, 1156

HRMS (ESI+): *m/z* calcd for $C_{31}H_{29}NO_2S_2Na[M+Na]^+$ 534.1532, found 534.1543

2,2-Di-*p*-tolyl-1-tosyl-1-azaspiro[2.3]hexane, 42

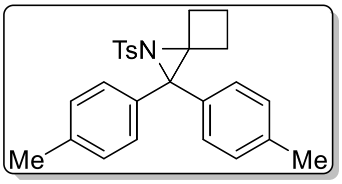

Prepared following **General Procedure 4** from *N*-(di-*p*-tolylmethylene)-4-methylbenzenesulfonamide **AH** (35 mg, 0.095 mmol) and cyclobutyl(phenyl)(2,4,6-trimethoxyphenyl)sulfonium tetrafluoroborate **8** (50 mg, 0.12 mmol). Work-up affords the crude material with a quantitative ^{1}H-NMR yield of 70%. Purification by flash column chromatography (8% EtOAc/hexane) affords the title compound **42** (15 mg, 0.036 mmol, 38%) as an off-white solid.

^{1}H-NMR (400 MHz, $CDCl_3$): δ_H 7.93 (2H, d, *J* = 8.3 Hz), 7.34 (2H, d, *J* = 8.1 Hz), 7.30 (4H, d, *J* = 8.2 Hz), 7.11 (4H, d, *J* = 7.9 Hz), 2.53 – 2.40 (5H, m), 2.39 – 2.26 (8H, m), 1.95 – 1.84 (1H, m), 1.82 – 1.70 (1H, m).

^{13}C{^{1}H}-NMR (101 MHz, $CDCl_3$): δ_C 143.9, 139.0, 137.4, 135.2, 129.6, 129.0, 128.6, 127.9, 61.7, 58.2, 29.1, 21.8, 21.3, 15.5.

IR (neat, *v* cm^{-1}): 2921, 1628, 1510, 1321, 1155.

HRMS (ESI+): *m/z* calcd for $C_{26}H_{27}NO_2SNa$ $[M+Na]^+$ 440.1655, found 440.1650

1'-Tosyldispiro[cyclobutane-1,2'-aziridine-3',9''-fluorene], 43

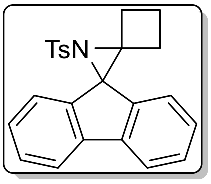

Prepared following **General Procedure 4** from *N*-(9H-fluoren-9-ylidene)-4-methylbenzenesulfonamide **AI** (30 mg, 0.095 mmol) and cyclobutyl(phenyl)(2,4,6-trimethoxyphenyl)sulfonium tetrafluoroborate **8** (50 mg, 0.12 mmol). Work-up affords the crude material with a quantitative ^{1}H-NMR yield of 80%. Purification by flash column chromatography (8% Et_2O/hexane) affords the title compound **43** (19 mg, 0.049 mmol, 52%) as an off-white solid.

^{1}H-NMR (400 MHz, $CDCl_3$): δ_H 7.84 – 7.72 (3H, m), 7.52 – 7.37 (3H, m), 7.36 – 7.19 (6H, m), 3.13 – 2.99 (2H, m), 2.65 – 2.47 (2H, m), 2.41 (3H, s), 1.99 – 1.90 (1H, m), 1.65 (1H, m).

^{13}C{^{1}H}-NMR (101 MHz, $CDCl_3$): δ_C 141.3, 139.1, 129.7, 128.8, 128.7, 128.0, 127.3, 127.2, 125.3, 120.2, 60.4, 58.5, 28.4, 21.7, 13.9.

IR (neat, *v* cm^{-1}): 2927, 2158, 1450, 1330, 1160.

HRMS (ESI+): *m/z* calcd for $C_{24}H_{21}NO_2Na$ [M+Na]$^{+}$ 410.1185, found 410.1207

2,2-Diphenyl-1-((4-(5-(*p*-tolyl)-3-(trifluoromethyl)-1*H*-pyrazol-1-yl)phenyl)sulfonyl)-1-azaspiro[2.3]hexane, 44

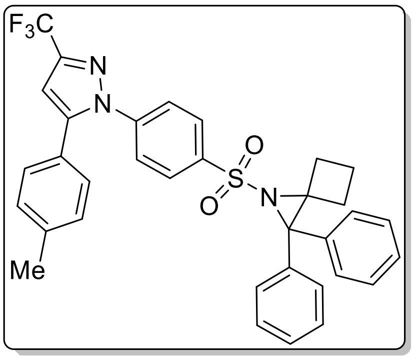

Prepared following **General Procedure 4** from *N*-(diphenylmethylene)-4-(5-(*p*-tolyl)-3-(trifluoromethyl)-1*H*-pyrazol-1-yl)benzenesulfonamide **AJ** (51 mg, 0.095 mmol) and cyclobutyl(phenyl)(2,4,6-trimethoxyphenyl)sulfonium tetrafluoroborate **8** (50 mg, 0.12 mmol). Work-up affords the crude material with a quantitative ^{1}H-NMR yield of 61%. Purification by flash column chromatography (15% EtOAc/hexane) affords the title compound **44** (18 mg, 0.030 mmol, 31%) as a white solid.

^{1}H-NMR (400 MHz, $CDCl_3$): δ_H 8.07 – 7.97 (2H, m), 7.57 – 7.48 (2H, m), 7.43 – 7.36 (4H, m), 7.36 – 7.25 (7H, m), 7.16 (2H, d, *J* = 8.0 Hz), 7.15 – 7.06 (2H, m), 2.48 – 2.29 (7H, m), 1.94 (1H, m), 1.85 – 1.77 (1H, m).

^{19}F-NMR (377 MHz, $CDCl_3$): δ_F -62.44 (s)

^{13}C{^{1}H}-NMR (101 MHz, $CDCl_3$): δ_C 145.5, 142.9, 141.0, 140.0, 138.8, 137.6, 129.9, 128.9, 128.9, 128.7, 128.4, 128.0, 125.9, 125.6, 106.5 (br. s), 62.5, 58.7, 29.2, 21.5, 15.5

Note: CF_3 and *C*-CF_3 not resolved

IR (neat, *v* cm^{-1}): 2927, 1597, 1471, 1237, 1160

HRMS (ESI+): *m/z* calcd for $C_{34}H_{28}F_3N_3O_2SNa$ $[M+Na]^+$ 622.1747, found 622.1760.

Isopropyl 2-(4-(2-(4-chlorophenyl)-1-((4-(5-(*p*-tolyl)-3-(trifluoromethyl)-1*H*-pyrazol-1-yl)phenyl)sulfonyl)-1-azaspiro[2.3]hexan-2-yl)phenoxy)-2-methylpropanoate, 45

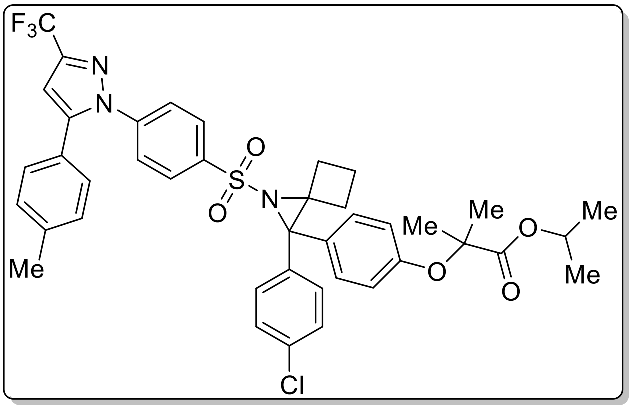

Prepared following **General Procedure 4** from isopropyl 2-(4-((4-chlorophenyl)(((4-(5-(*p*-tolyl)-3-(trifluoromethyl)-1*H*-pyrazol-1-yl)phenyl)sulfonyl)imino)methyl)phenoxy)-2-methylpropanoate **AK** (69 mg, 0.095 mmol) and cyclobutyl(phenyl)(2,4,6-trimethoxyphenyl)sulfonium tetrafluoroborate **8** (50 mg, 0.12 mmol). Work-up affords the crude material with a quantitative ^{1}H-NMR yield of 78%. Purification by flash column chromatography (25% Et_2O/hexane) affords the title compound **45** (25 mg, 0.032 mmol, 34%) as a white solid.

^{1}H-NMR (400 MHz, $CDCl_3$): δ_H 8.04 – 7.96 (2H, m), 7.62 – 7.49 (2H, m), 7.28 (4H, s), 7.26 – 7.14 (4H, m), 7.11 (2H, d, *J* = 8.2 Hz), 6.80 – 6.73 (3H, m), 5.05 (1H, hept, *J* = 6.0 Hz), 2.38 (5H, m), 2.32 – 2.18 (2H, m), 1.96 – 1.74 (2H, m), 1.58 (6H, s), 1.17 (6H, d, *J* = 6.2 Hz).

^{19}F-NMR (377 MHz, $CDCl_3$): δ_F -62.14 (s)

^{13}C{^{1}H}-NMR (101 MHz, $CDCl_3$): δ_C 173.6, 155.6, 144.3 (q, *J* = 38.7 Hz), 143.0, 140.8, 140.0, 136.3, 134.0, 130.1, 130.1, 129.9, 129.6, 128.9, 128.9, 125.9, 125.6, 121.2 (q, *J* = 269.3 Hz), 118.4, 106.5 (br. s), 79.3, 69.1, 29.1, 29.1, 25.6, 21.7, 21.5, 15.5

IR (neat, *v* cm^{-1}): 2983, 1728, 1236, 1159, 1096

HRMS (ESI+): *m/z* calcd for $C_{41}H_{39}{}^{35}ClF_3N_3O_5SNa$ $[M+Na]^+$ 800.2148, found 800.2160.

Ethyl 1-phenyl-5-oxaspiro[2.3]hexane-1-carboxylate, 46

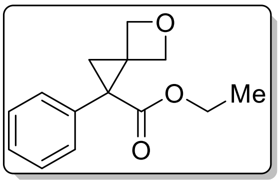

Prepared following **General Procedure 4** from ethyl 2-phenylacrylate **G** (35 mg, 0.19 mmol) and (4-chlorophenyl)(oxetan-3-yl)(2,4,6-trimethoxyphenyl)sulfonium tetrafluoroborate **9** (110 mg, 0.25 mmol). Work-up affords the crude material with a quantitative ^{1}H-NMR yield of 85%. Partial purification by flash column chromatography (10% DCM/hexane to 40% EtOAc/hexane) affords the title compound **46** (22 mg) as an off-white solid, which co-elutes with an inseparable impurity.

Note: Indicative peaks were used for partial characterization.

^{1}H-NMR (400 MHz, $CDCl_3$): δ_H 7.37 – 7.27 (5H, m), 4.95 – 4.85 (2H, m), 4.50 (1H, d, *J* = 6.5 Hz), 4.36 (1H, d, *J* = 6.5 Hz), 4.23 – 3.99 (2H, m), 1.91 (1H, d, *J* = 5.4 Hz), 1.58 (1H, d, *J* = 5.4 Hz), 1.18 (3H, t, *J* = 7.1 Hz).

^{13}C{^{1}H}-NMR (101 MHz, $CDCl_3$): δ_C 171.7, 135.0, 130.7, 128.4, 77.7, 75.7, 61.3, 35.9, 34.2, 23.2, 14.4

HRMS(ESI+): *m/z* calcd for $C_{14}H_{16}O_3Na$ $[M+Na]^+$ 255.0997, found 255.0996.

Ethyl 1-phenyl-5-tosyl-5-azaspiro[2.3]hexane-1-carboxylate, 47

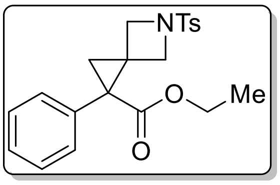

Prepared following **General Procedure 4** from ethyl 2-phenylacrylate **G** (33 mg, 0.19 mmol) and (4-chlorophenyl)(1-tosylazetidin-3-yl)(2,4,6-trimethoxyphenyl)sulfonium tetrafluoroborate **10** (152 mg, 0.24 mmol). Work-up affords the crude material with a quantitative ^{1}H-NMR yield of 87%. Purification by flash column chromatography (30% to 70% DCM/hexane) affords the title compound **47** (26 mg, 0.067 mmol, 35%) as a white solid.

^{1}H-NMR (400 MHz, $CDCl_3$): δ_H 7.81 – 7.68 (2H, m), 7.44 – 7.39 (2H, m), 7.27 – 7.23 (3H, m), 6.89 (2H, ddd, *J* = 5.6, 3.0, 1.6 Hz), 4.15 – 3.95 (4H, m), 3.55 (1H, d, *J* = 9.0 Hz), 3.44 (1H, d, *J* = 8.9 Hz), 2.51 (3H, s), 1.77 (1H, d, *J* = 5.6 Hz), 1.45 (1H, d, *J* = 5.6 Hz), 1.14 (3H, t, *J* = 7.1 Hz).

^{13}C{^{1}H}-NMR (101 MHz, $CDCl_3$): δ_C 171.3, 144.4, 134.3, 131.9, 130.5, 129.9, 128.7, 128.5, 127.9, 61.5, 57.2, 55.3, 35.9, 28.8, 23.5, 21.8, 14.4.

IR (neat, *v* cm^{-1}): 2979, 1712, 1345, 1230, 1158

HRMS(ESI+): *m/z* calcd for $C_{21}H_{23}NO_4SNa$ $[M+Na]^+$ 408.1240, found 408.1241.

1-Phenyl-1-(trifluoromethyl)-5-oxaspiro[2.3]hexane, 48

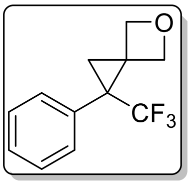

Prepared following **General Procedure 4** from (3,3,3-trifluoroprop-1-en-2-yl)benzene **A** (16 mg, 0.095 mmol) and (4-chlorophenyl)(oxetan-3-yl)(2,4,6-trimethoxyphenyl)sulfonium tetrafluoroborate **9** (56 mg, 0.12 mmol). Work-up affords the crude material with a quantitative ^{1}H-NMR yield of 97%. Purification by flash column chromatography (8% Et_2O/hexane) affords the title compound **48** (13 mg, 0.057 mmol, 60%) as a colourless oil

^{1}H-NMR (400 MHz, $CDCl_3$): δ_H 7.48 – 7.44 (2H, m), 7.41 – 7.34 (3H, m), 5.06 (1H, dd, *J* = 6.7, 1.6 Hz), 4.84 (1H, d, *J* = 6.6 Hz), 4.66 (1H, d, *J* = 6.4 Hz), 4.39 (1H, d, *J* = 6.4 Hz), 1.70 (1H, d, *J* = 6.4 Hz), 1.38 (1H, dd, *J* = 6.5, 1.9 Hz).

^{19}F-NMR (377 MHz, $CDCl_3$): δ_F – 67.23 (s)

^{13}C{^{1}H}-NMR (101 MHz, $CDCl_3$): δ_C 131.8, 131.2, 128.9, 128.9, 125.7 (q, *J* = 275.2 Hz), 76.3 (q, *J* = 2.1 Hz), 75.6, 33.7 (q, *J* = 32.5 Hz), 29.0, 18.4 (q, *J* = 3.0 Hz)

IR (neat, *v* cm^{-1}): 2960, 2878, 1968, 1343, 1231

HRMS (ESI+): *m/z* calcd for $C_{12}H_{11}F_3ONa$ $[M+Na]^+$ 251.0654, found 251.0669.

1-Phenyl-5-tosyl-1-(trifluoromethyl)-5-azaspiro[2.3]hexane, 49

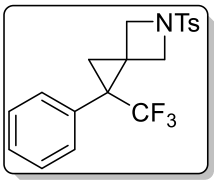

Prepared following **General Procedure 4** from (3,3,3-trifluoroprop-1-en-2-yl)benzene **A** (33 mg, 0.19 mmol) and (4-chlorophenyl)(1-tosylazetidin-3-yl)(2,4,6-trimethoxyphenyl)sulfonium tetrafluoroborate **10** (152 mg, 0.25 mmol). Work-up affords the crude material with a quantitative ^{1}H-NMR yield of 76%. Purification by flash column chromatography (hexane to 50% DCM/hexane) affords the title compound **49** (23 mg, 0.060 mmol, 32%) as a white solid.

A sample suitable for x-ray crystallography was obtained by slow evaporation from ethyl acetate.

^{1}H-NMR (400 MHz, $CDCl_3$): δ_H 7.79 (2H, d, *J* = 8.3 Hz), 7.46 (2H, d, *J* = 8.0 Hz), 7.35 – 7.25 (3H, m), 6.99 – 6.90 (2H, m), 4.23 (1H, d, *J* = 9.3 Hz), 4.06 (1H, d, *J* = 9.3 Hz), 3.66 (1H, d, *J* = 9.0 Hz), 3.49 (1H, d, *J* = 9.0 Hz), 2.52 (3H, s), 1.58 (1H, d, *J* = 6.6 Hz), 1.26 (1H, dd, *J* = 6.5, 2.0 Hz).

^{19}F-NMR (377 MHz, $CDCl_3$): δ_F -66.96 (s).

^{13}C{^{1}H}-NMR (101 MHz, $CDCl_3$): δ_C 144.6, 131.5, 131.0, 130.9, 130.0, 129.0, 128.8, 128.7, 125.3 (q, *J* = 275.4 Hz), 55.9, 55.3, 33.7 (q, *J* = 32.5 Hz), 23.5 (br. s), 21.8, 18.6 (q, *J* = 2.9 Hz)

IR (neat, *v* cm^{-1}): 2951, 1597, 1341, 1154, 1109

HRMS(ESI+): *m/z* calcd for $C_{19}H_{18}F_3NO_2SNa$ $[M+Na]^+$404.0903, found 404.0918

1-(4-Fluorophenyl)-1-(trifluoromethyl)-5-oxaspiro[2.3]hexane, 50

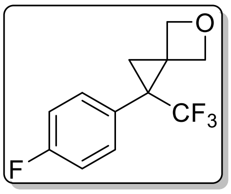

Prepared following **General Procedure 4** from 1-fluoro-4-(3,3,3-trifluoroprop-1-en-2-yl)benzene **C** (18 mg, 0.095 mmol) and (4-chlorophenyl)(oxetan-3-yl)(2,4,6-trimethoxyphenyl)sulfonium tetrafluoroborate **9** (56 mg, 0.12 mmol). Work-up affords the crude material with a quantitative ^{1}H-NMR yield of 70%. Purification by flash column chromatography (15% Et_2O/hexane) affords the title compound **50** (9 mg, 0.036 mmol, 38%) as a colourless oil

^{1}H-NMR (400 MHz, $CDCl_3$): δ_H 7.44 (2H, dd, *J* = 8.5, 5.4 Hz), 7.14 – 7.04 (2H, m), 5.04 (1H, dd, *J* = 6.5, 1.7 Hz), 4.84 (1H, d, *J* = 6.6 Hz), 4.63 (1H, d, *J* = 6.5 Hz), 4.39 (1H, d, *J* = 6.5 Hz), 1.70 (1H, d, *J* = 6.5 Hz), 1.34 (1H, dd, *J* = 6.5, 1.9 Hz).

^{19}F-NMR (377 MHz, $CDCl_3$): δ_F -67.49 (3F, s), -112.39 (1F, tt, *J* = 8.5, 5.3 Hz).

^{13}C{^{1}H}-NMR (101 MHz, $CDCl_3$): δ_C 163.0 (d, *J* = 248.6 Hz), 133.1 (d, *J* = 8.4 Hz), 127.7 (m), 125.6 (q, *J* = 275.9 Hz), 116.0 (d, *J* = 21.7 Hz), 76.2 (q, *J* = 2.1 Hz), 75.5, 33.0 (q, *J* = 32.8 Hz), 29.1 (br. s), 18.5 (q, *J* = 2.9 Hz)

IR (neat, *v* cm^{-1}): 2962, 2880, 1607, 1513, 1345

HRMS: *exact mass not found in ESI+ or ESI- mode*

1-(4-Fluorophenyl)-5-tosyl-1-(trifluoromethyl)-5-azaspiro[2.3]hexane, 51

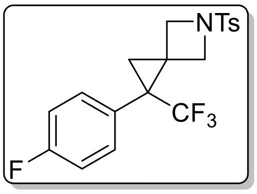

Prepared following **General Procedure 4** from 1-fluoro-4-(3,3,3-trifluoroprop-1-en-2-yl)benzene **C** (36 mg, 0.19 mmol) and (4-chlorophenyl)(1-tosylazetidin-3-yl)(2,4,6-trimethoxyphenyl)sulfonium tetrafluoroborate **10** (152 mg, 0.25 mmol). Work-up affords the crude material with a quantitative ^{1}H-NMR yield of 73%. Purification by flash column chromatography (hexane to 70% DCM/hexane) affords the title compound **51** (26 mg, 0.065 mmol, 34%) as a colourless oil.

^{1}H-NMR (400 MHz, $CDCl_3$): δ_H 7.79 (2H, d, *J* = 8.3 Hz), 7.45 (2H, d, *J* = 8.0 Hz), 7.02 – 6.90 (4H, m), 4.22 (1H, d, *J* = 9.3 Hz), 4.05 (1H, d, *J* = 9.3 Hz), 3.63 (1H, d, *J* = 9.1 Hz), 3.51 (1H, d, *J* = 9.0 Hz), 2.52 (3H, s), 1.59 (1H, d, *J* = 2.3 Hz), 1.23 (1H, dd, *J* = 6.7, 1.9 Hz).

^{19}F-NMR (377 MHz, $CDCl_3$): δ_F -67.19 (3F, s), -111.90 (1F, m).

^{13}C{^{1}H}-NMR (101 MHz, $CDCl_3$): δ_C 163.0 (d, *J* = 249.3 Hz), 144.7, 132.8 (d, *J* = 8.4 Hz), 131.6, 130.0, 128.8, 126.9 (d, *J* = 3.5 Hz), 125.2 (q, *J* = 275.2 Hz), 116.0 (d, *J* = 21.7 Hz), 55.8 (q, *J* = 2.0 Hz), 55.1, 33.1 (q, *J* = 32.8 Hz), 23.7 (br. s), 21.8, 18.8 (q, *J* = 2.9 Hz)

IR (neat, *v* cm^{-1}): 2951, 1511, 1342, 1228, 1155

HRMS(ESI+): *m/z* calcd for $C_{19}H_{17}F_4NO_2S$ $[M+Na]^+$ 422.0808, found 422.0821

1-(3-Bromophenyl)-5-tosyl-1-(trifluoromethyl)-5-azaspiro[2.3]hexane, 52

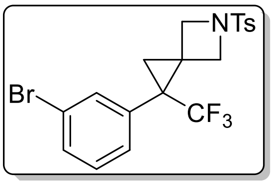

Prepared following **General Procedure 4** from 1-bromo-3-(3,3,3-trifluoroprop-1-en-2-yl)benzene **D** (35 mg, 0.14 mmol) and (4-chlorophenyl)(1-tosylazetidin-3-yl)(2,4,6-trimethoxyphenyl)sulfonium tetrafluoroborate **10** (109 mg, 0.18 mmol). Work-up affords the crude material with a quantitative ^{1}H-NMR yield of 74%. Purification by flash column chromatography (hexane to 70% DCM/hexane) affords the title compound **52** (14 mg, 0.030 mmol, 22%) as a colourless oil.

^{1}H-NMR (400 MHz, $CDCl_3$): δ_H 7.79 (2H, d, *J* = 8.4 Hz), 7.48 (3H, dd, *J* = 8.6, 5.2 Hz), 7.18 (1H, t, *J* = 7.9 Hz), 7.08 (1H, s), 6.94 (1H, d, *J* = 7.7 Hz), 4.18 (1H, d, *J* = 9.4 Hz), 4.05 (1H, d, *J* = 9.3 Hz), 3.63 (1H, d, *J* = 9.1 Hz), 3.53 (1H, d, *J* = 9.1 Hz), 2.53 (3H, s), 1.59 (1H, d, *J* = 5.5 Hz), 1.39 – 1.24 (1H, m).

^{19}F-NMR (377 MHz, $CDCl_3$): δ_F -66.89 (s).

^{13}C{^{1}H}-NMR (101 MHz, $CDCl_3$): δ_C 144.7, 133.7, 133.3, 132.4, 131.1, 130.5, 130.2, 129.7, 128.7, 125.0 (q, *J* = 275.5 Hz), 122.8, 55.9, 55.1, 33.4 (q, *J* = 32.9 Hz), 23.7, 21.9, 18.8 (q, *J* = 2.9 Hz)

IR (neat, *v* cm^{-1}): 2925, 1596, 1340, 1157, 1110

HRMS(ESI+): *m/z* calcd for $C_{19}H_{18}{}^{79}BrF_3NO_2S$ $[M+H]^+$ 460.0193, found 460.0191

1-(4-Bromophenyl)-1-(trifluoromethyl)-5-oxaspiro[2.3]hexane, 53

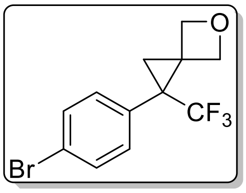

Prepared following **General Procedure 4** from 1-bromo-4-(3,3,3-trifluoroprop-1-en-2-yl)benzene **D** (50 mg, 0.19 mmol) and (4-chlorophenyl)(oxetan-3-yl)(2,4,6-trimethoxyphenyl)sulfonium tetrafluoroborate **9** (110 mg, 0.25 mmol). Work-up affords the crude material with a quantitative ^{1}H-NMR yield of 74%. Purification by flash column chromatography (10-60% DCM/hexane) affords the title compound **53** (31 mg, 0.10 mmol, 53%) as a colourless oil

^{1}H-NMR (400 MHz, $CDCl_3$): δ_H 7.56 – 7.49 (2H, m), 7.35 – 7.28 (2H, m), 5.03 (1H, d, J = 6.7 Hz), 4.83 (1H, d, J = 6.6 Hz), 4.61 (1H, d, J = 6.5 Hz), 4.39 (1H, d, J = 6.5 Hz), 1.71 (1H, d, J = 6.5 Hz), 1.36 – 1.32 (1H, m).

^{19}F-NMR (377 MHz, $CDCl_3$): δ_F -67.29 (s).

^{13}C{^{1}H}-NMR (101 MHz, $CDCl_3$): δ_C 132.9, 132.2, 130.8, 123.4, 76.1 (br. s), 75.4, 29.9, 18.4 (br. s)

Note: CF_3 not resolved

IR (neat, v cm^{-1}): 2916, 2878, 1492, 1344, 1230

HRMS: *exact mass not found in ESI+ or ESI- mode*

1-(4-Bromophenyl)-5-tosyl-1-(trifluoromethyl)-5-azaspiro[2.3]hexane, 54

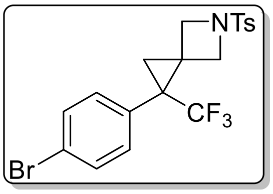

Prepared following **General Procedure 4** from 1-bromo-4-(3,3,3-trifluoroprop-1-en-2-yl)benzene **B** (35 mg, 0.14 mmol) and (4-chlorophenyl)(1-tosylazetidin-3-yl)(2,4,6-trimethoxyphenyl)sulfonium tetrafluoroborate **10** (109 mg, 0.18 mmol). Work-up affords the crude material with a quantitative ^{1}H-NMR yield of 77%. Purification by flash column chromatography (hexane to 70% DCM/hexane) affords the title compound **54** (35 mg, 0.076 mmol, 54%) as a white solid.

A sample suitable for x-ray crystallography was obtained by slow evaporation from ethyl acetate.

^{1}H-NMR (400 MHz, $CDCl_3$): δ_H 7.78 (2H, d, *J* = 7.8 Hz), 7.44 (4H, m), 6.83 (2H, d, *J* = 8.0 Hz), 4.21 (1H, d, *J* = 9.4 Hz), 4.05 (1H, d, *J* = 9.3 Hz), 3.61 (1H, d, *J* = 9.1 Hz), 3.50 (1H, d, *J* = 9.1 Hz), 2.52 (3H, s), 1.33 – 1.18 (2H, m).

^{19}F-NMR (377 MHz, $CDCl_3$): δ_F -67.02 (s)

^{13}C{^{1}H}-NMR (101 MHz, $CDCl_3$): δ_C 144.7, 132.6, 132.2, 131.6, 130.1, 130.0, 128.8, 125.0 (q, *J* = 275.7 Hz), 123.5, 55.8, 55.1, 33.3 (q, *J* = 32.7 Hz), 23.6, 21.8, 18.7 (q, *J* = 2.8 Hz)

IR (neat, ν cm^{-1}): 2925, 1596, 1491, 1342, 1157

HRMS(ESI+): *m/z* calcd for $C_{19}H_{18}{}^{79}BrF_3NO_2S$ $[M+H]^+$ 460.0193, found 460.0190

2-(4-Bromophenyl)-2-phenyl-1,5-dioxaspiro[2.3]hexane, 55

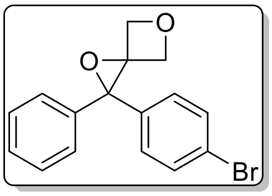

Prepared following **General Procedure 4** from (4-bromophenyl)(phenyl)methanone **Q** (37 mg, 0.14 mmol) and (4-chlorophenyl)(oxetan-3-yl)(2,4,6-trimethoxyphenyl)sulfonium tetrafluoroborate **9** (80 mg, 0.18 mmol). Work-up affords the crude material with a quantitative ^{1}H-NMR yield of 75%. Purification by flash column chromatography (30% to 70% DCM/hexane) affords the title compound **55** (14 mg, 0.044 mmol, 32%) as a white solid.

^{1}H-NMR (400 MHz, $CDCl_3$): δ_H 7.52 – 7.47 (2H, m), 7.40 – 7.33 (3H, m), 7.31 – 7.25 (2H, m), 7.20 – 7.16 (2H, m), 4.89 (2H, m), 4.73 (1H, dd, J = 8.4, 1.5 Hz), 4.70 (1H, m)

^{13}C{^{1}H}-NMR (101 MHz, $CDCl_3$): δ_C 136.3, 135.9, 131.7, 129.0, 128.8, 128.5, 127.3, 122.7, 77.9, 77.8, 70.1, 66.7

The spectroscopic data are in agreement with those previously reported.[15]

2-(4-Bromophenyl)-2-phenyl-5-tosyl-1-oxa-5-azaspiro[2.3]hexane, 56

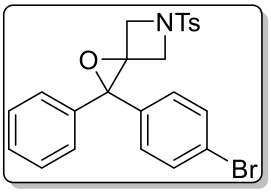

Prepared following **General Procedure 4** from (4-bromophenyl)(phenyl)methanone **Q** (37 mg, 0.14 mmol) and (4-chlorophenyl)(1-tosylazetidin-3-yl)(2,4,6-trimethoxyphenyl)sulfonium tetrafluoroborate **10** (109 mg, 0.18 mmol). Work-up affords the crude material with a quantitative ^{1}H-NMR yield of 74%. Purification by flash column chromatography (30% to 70% DCM/hexane) affords the title compound **56** (36 mg, 0.077 mmol, 55%) as a white solid.

^{1}H-NMR (400 MHz, $CDCl_3$): δ_H 7.75 – 7.71 (2H, m), 7.50 – 7.44 (2H, m), 7.39 (2H, d, *J* = 7.9 Hz), 7.35 – 7.32 (3H, m), 7.16 – 7.13 (2H, m), 7.08 – 7.03 (2H, m), 4.01 – 3.93 (3H, m), 3.92 – 3.88 (1H, m), 2.49 (3H, s).

^{13}C{^{1}H}-NMR (101 MHz, $CDCl_3$): δ_C 144.8, 135.5, 135.2, 131.9, 131.7, 130.1, 128.9, 128.8, 128.7, 128.6, 127.1, 122.8, 67.0, 65.5, 57.5, 57.4, 21.8

IR (neat, *v* cm^{-1}): 2926, 1596, 1348, 1160, 1011

HRMS(ESI+): *m/z* calcd for $C_{23}H_{20}{}^{81}BrNO_3SNa$ $[M+Na]^+$ 494.0221, found 494.0246.

2-(2,4-Dichlorophenyl)-1,5-dioxaspiro[2.3]hexane, 57

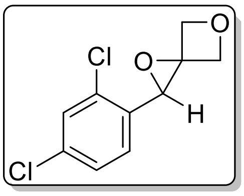

Prepared following **General Procedure 4** from 2,4-dichlorobenzaldehyde **X** (17 mg, 0.095 mmol) and (4-chlorophenyl)(oxetan-3-yl)(2,4,6-trimethoxyphenyl)sulfonium tetrafluoroborate **9** (56 mg, 0.12 mmol). Work-up affords the crude material with a quantitative ^{1}H-NMR yield of 88%. Purification by flash column chromatography (20% Et_2O/hexane) affords the title compound **57** (12 mg, 0.052 mmol, 55%) as a white solid.

^{1}H-NMR (400 MHz, $CDCl_3$): δ_H 7.43 (1H, d, J = 2.1 Hz), 7.23 (1H, dd, J = 8.3, 2.1 Hz), 6.96 (1H, d, J = 8.4 Hz), 5.09 – 5.02 (2H, m), 4.83 (1H, d, J = 8.2 Hz), 4.45 (1H, dd, J = 8.1, 1.2 Hz), 4.26 (s, 1H).

^{13}C{^{1}H}-NMR (101 MHz, $CDCl_3$): δ_C 134.8, 133.8, 131.5, 129.5, 127.6, 127.1, 78.4, 77.0, 65.3, 57.4.

IR (neat, v cm^{-1}): 2944, 2871, 1592, 1379, 1099

HRMS (ESI+): m/z calcd for $C_{10}H_8{}^{35}Cl_2O_2Na$ $[M+Na]^+$ 252.9799, found 253.0039.

1-((2-Bromophenyl)sulfonyl)-3-(1,5-dioxaspiro[2.3]hexan-2-yl)-1*H*-indole, 58

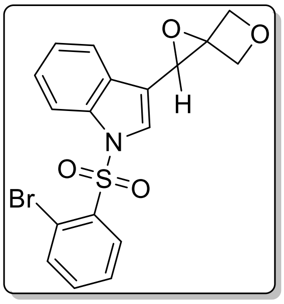

Prepared following **General Procedure 4** from 1-((2-Bromophenyl)sulfonyl)-1*H*-indole-3-carbaldehyde **AB** (35 mg, 0.095 mmol) and (4-chlorophenyl)(oxetan-3-yl)(2,4,6-trimethoxyphenyl)sulfonium tetrafluoroborate **9** (56 mg, 0.12 mmol). Work-up affords the crude material with a quantitative ^{1}H-NMR yield of 81%. Purification by flash column chromatography deactivated with triethylamine (1%) (50% Et_2O/hexane, 1% triethylamine) affords the title compound **58** (26 mg, 0.062 mmol, 65%) as a white solid.

^{1}H-NMR (400 MHz, $CDCl_3$): δ_H 8.20 (1H, dd, *J* = 8.0, 1.7 Hz), 7.69 – 7.57 (4H, m), 7.51 (1H, m), 7.42 (1H, m), 7.29 – 7.24 (2H, m), 5.11 – 4.92 (3H, m), 4.64 (1H, dd, *J* = 8.1, 1.3 Hz), 4.22 (1H, s).

^{13}C{^{1}H}-NMR (101 MHz, $CDCl_3$): δ_C 137.8, 136.3, 135.2, 135.0, 131.9, 128.8, 128.1, 125.7, 125.3, 123.7, 120.9, 119.9, 115.4, 113.5, 78.4, 77.7, 65.4, 54.8.

IR (neat, v cm^{-1}): 2944, 2871, 1573, 1447, 1179

HRMS (ESI+): *m/z* calcd for $C_{18}H_{14}{}^{81}BrNO_4SNa$ $[M+Na]^+$ 443.9700, found 443.9697.

2-(1-((2-Bromophenyl)sulfonyl)-1*H*-indol-3-yl)-5-tosyl-1-oxa-5-azaspiro[2.3]hexane, 59

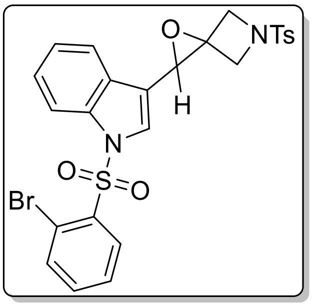

Prepared following **General Procedure 4** from 1-((2-bromophenyl)sulfonyl)-1*H*-indole-3-carbaldehyde **AB** (70 mg, 0.19 mmol) and (4-chlorophenyl)(1-tosylazetidin-3-yl)(2,4,6-trimethoxyphenyl)sulfonium tetrafluoroborate **10** (152 mg, 0.25 mmol). Work-up affords the crude material with a quantitative ^{1}H-NMR yield of 91%. Purification by flash column chromatography (30% EtOAc/hexane) affords the title compound **59** (32 mg, 0.055 mmol, 29%) as a white solid.

^{1}H-NMR (400 MHz, $CDCl_3$): δ_H 8.22 (1H, dd, *J*= 8.0, 1.7 Hz), 7.77 – 7.74 (2H, m), 7.70 (1H, dd, *J*= 7.8, 1.3 Hz), 7.67 – 7.63 (1H, m), 7.56 – 7.51 (2H, m), 7.50 – 7.42 (2H, m), 7.38 (2H, d, *J*= 8.0 Hz), 7.32 – 7.23 (2H, m), 4.31 – 4.21 (2H, m), 4.18 – 4.06 (2H, m), 3.84 (1H, dd, *J*= 10.0, 1.3 Hz), 2.49 (3H, s).

^{13}C{^{1}H}-NMR (101 MHz, $CDCl_3$): δ_C 144.6, 137.5, 136.2, 135.1, 134.7, 131.7, 131.4, 130.0, 128.4, 128.0, 125.6, 125.2, 123.6, 120.7, 119.7, 114.6, 113.3, 60.8, 57.9, 56.9, 55.0, 21.7.

IR (neat, *v* cm^{-1}): 3068, 2925, 1447, 1346, 1179

HRMS(ESI+): *m/z* calcd for $C_{25}H_{21}{}^{81}BrN_2O_5S_2Na$ $[M+Na]^+$ 596.9949, found 597.0000.

5-(4-Fluorophenyl)-3-(1,5-dioxaspiro[2.3]hexan-2-yl)isoxazole, 60

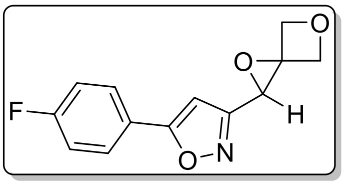

Prepared following **General Procedure 4** from 5-(4-fluorophenyl)isoxazole-3-carbaldehyde **AC** (28 mg, 0.15 mmol) and (4-chlorophenyl)(oxetan-3-yl)(2,4,6-trimethoxyphenyl)sulfonium tetrafluoroborate **9** (85 mg, 0.19 mmol). Work-up affords the crude material with a quantitative ^{1}H-NMR yield of 59%. Purification by flash column chromatography deactivated with triethylamine (1%) (40% Et_2O/hexane, 1% triethylamine) affords the title compound **60** (11 mg, 0.044 mmol, 30%) as a colourless oil

^{1}H-NMR (400 MHz, $CDCl_3$): δ_H 7.75 (2H, dd, *J* = 8.9, 5.1 Hz), 7.17 (2H, app. t, *J* = 8.6 Hz), 6.09 (1H, s), 5.12 – 5.02 (3H, m), 4.91 – 4.80 (1H, m), 4.19 (1H, s).

^{19}F-NMR (377 MHz, $CDCl_3$): δ_F -108.56 (s)

^{13}C{^{1}H}-NMR (101 MHz, $CDCl_3$): δ_C 170.2, 164.2 (d, *J* = 252.1 Hz), 160.9, 128.2 (d, *J* = 8.7 Hz), 123.3 (d, *J* = 3.5 Hz), 116.5 (d, *J* = 22.2 Hz), 96.5, 78.1, 77.3, 64.7, 52.8

IR (neat, *v* cm^{-1}): 2943, 2874, 1614, 1495, 1445

HRMS (ESI+): *m/z* calcd for $C_{13}H_{10}FNO_3Na$ $[M+Na]^+$ 270.0537, found 270.0537.

2-(5-(4-Fluorophenyl)isoxazol-3-yl)-5-tosyl-1-oxa-5-azaspiro[2.3]hexane, 61

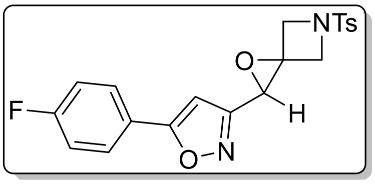

Prepared following **General Procedure 4** from 5-(4-fluorophenyl)isoxazole-3-carbaldehyde **AC** (38 mg, 0.19 mmol) and (4-chlorophenyl)(1-tosylazetidin-3-yl)(2,4,6-trimethoxyphenyl)sulfonium tetrafluoroborate **10** (152 mg, 0.25 mmol). Work-up affords the crude material with a quantitative ^{1}H-NMR yield of 95%. Purification by flash column chromatography on SiO_2 deactivated with triethylamine (1%) (20% EtOAc/hexane, 1% triethylamine) affords the title compound **61** (25 mg, 0.062 mmol, 33%) as a white solid.

^{1}H-NMR (400 MHz, $CDCl_3$): δ_H 7.78 – 7.70 (4H, m), 7.39 – 7.36 (2H, m), 7.20 – 7.13 (2H, m), 6.09 (1H, s), 4.30 – 4.16 (3H, m), 4.10 – 4.03 (2H, m), 2.47 (3H, s).

^{19}F-NMR (377 MHz, $CDCl_3$): δ_F -108.35 (s)

^{13}C{^{1}H}-NMR (101 MHz, $CDCl_3$): δ_C 170.3, 164.2 (d, *J* = 253.5 Hz), 160.3, 144.9, 131.8, 130.2, 128.5, 128.2 (d, *J* = 8.7 Hz), 123.2, 116.6 (d, *J* = 22.2 Hz), 96.6, 60.4, 57.9, 56.9, 53.0, 21.8

IR (neat, *v* cm^{-1}): 2925, 1613, 1445, 1345, 1159

HRMS(ESI+): *m/z* calcd for $C_{20}H_{17}FN_2O_4SNa$ $[M+Na]^+$ 423.0785, found 423.9791.

3-(1,5-Dioxaspiro[2.3]hexan-2-yl)pyridine, 62

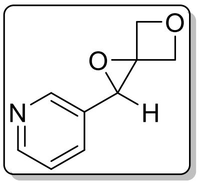

Prepared following **General Procedure 4** from nicotinaldehyde **AD** (20 mg, 0.19 mmol) and (4-chlorophenyl)(oxetan-3-yl)(2,4,6-trimethoxyphenyl)sulfonium tetrafluoroborate **9** (110 mg, 0.25 mmol). Work-up affords the crude material with a quantitative ^{1}H-NMR yield of 67%. Purification by flash column chromatography deactivated with triethylamine (1%) (55% EtOAc/hexane, 1% triethylamine) affords the title compound **62** (16 mg, 0.098 mmol, 52%) as a colourless oil

^{1}H-NMR (400 MHz, $CDCl_3$): δ_H 8.59 (1H, dd, *J* = 4.8, 1.7 Hz), 8.49 (1H, d, *J* = 2.3 Hz), 7.38 – 7.27 (2H, m), 5.08 – 4.96 (3H, m), 4.59 – 4.55 (1H, m), 4.03 (1H, s).

^{13}C{^{1}H}-NMR (101 MHz, $CDCl_3$): δ_C 150.1, 148.0, 133.1, 130.6, 123.6, 78.4, 77.1, 65.6, 58.0.

IR (neat, v cm^{-1}): 2940, 2871, 1576, 1418, 1417.

HRMS (ESI+): *m/z* calcd for $C_9H_{10}NO_2$ $[M+H]^+$ 164.0706, found 164.0696.

2-(Pyridin-3-yl)-5-tosyl-1-oxa-5-azaspiro[2.3]hexane, 63

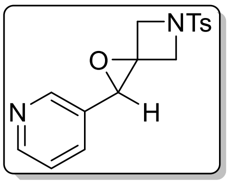

Prepared following **General Procedure 4** from nicotinaldehyde **AD** (20 mg, 0.19 mmol) and (4-chlorophenyl)(1-tosylazetidin-3-yl)(2,4,6-trimethoxyphenyl)sulfonium tetrafluoroborate **10** (152 mg, 0.25 mmol). Work-up affords the crude material with a quantitative ^{1}H-NMR yield of 92%. Purification by flash column chromatography deactivated with triethylamine (1%) (55% EtOAc/hexane, 1% triethylamine) affords the title compound **63** (36 mg, 0.11 mmol, 59%) as a white solid.

^{1}H-NMR (400 MHz, $CDCl_3$): δ_H 8.60 (1H, dd, *J* = 4.2, 2.3 Hz), 8.38 (1H, t, *J* = 1.6 Hz), 7.76 – 7.73 (2H, m), 7.38 (2H, d, *J* = 8.0 Hz), 7.30 (2H, dt, *J* = 4.3, 1.5 Hz), 4.30 – 4.18 (2H, m), 4.11 (1H, d, *J* = 10.1 Hz), 3.95 (1H, s), 3.75 (1H, dd, *J* = 10.1, 1.3 Hz), 2.49 (3H, s).

^{13}C{^{1}H}-NMR (101 MHz, $CDCl_3$): δ_C 150.3, 147.9, 144.8, 133.0, 131.6, 130.1, 129.9, 128.5, 123.5, 61.2, 58.2, 58.1, 56.4, 21.8.

IR (neat, *v* cm^{-1}): 2924, 1596, 1344, 1157

HRMS(ESI+): *m/z* calcd for $C_{16}H_{16}N_2O_3SNa$ $[M+Na]^+$ 339.0774, found 339.0781.

2,2-Bis(4-chlorophenyl)-1-tosyl-5-oxa-1-azaspiro[2.3]hexane, 64

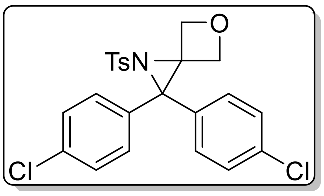

Prepared following **General Procedure 4** from *N*-(bis(4-chlorophenyl)methylene)-4-methylbenzenesulfonamide **O** (36 mg, 0.095 mmol) and (4-chlorophenyl)(oxetan-3-yl)(2,4,6-trimethoxyphenyl)sulfonium tetrafluoroborate **9** (56 mg, 0.12 mmol). Work-up affords the crude material with a quantitative ^{1}H-NMR yield of 52%. Purification by flash column chromatography (20% Et_2O/hexane) affords the title compound **64** (12 mg, 0.026 mmol, 27%) as an off-white solid.

^{1}H-NMR (400 MHz, $CDCl_3$): δ_H 7.97 – 7.86 (2H, m), 7.48 – 7.31 (10H, m), 4.95 – 4.87 (2H, m), 4.82 – 4.71 (2H, m), 2.49 (3H, s).

^{13}C{^{1}H}-NMR (101 MHz, $CDCl_3$): δ_C 145.2, 137.6, 134.9, 134.5, 130.2, 129.9, 129.1, 127.8, 75.5, 58.3, 56.1, 21.9.

IR (neat, *v* cm^{-1}): 2925, 1596, 1491, 1331, 1160.

HRMS (ESI+): *m/z* calcd for $C_{23}H_{19}{}^{35}Cl_2NO_3SNa$ $[M+Na]^+$ 482.0355, found 482.0360.

2,2-Bis(4-chlorophenyl)-1,5-ditosyl-1,5-diazaspiro[2.3]hexane, 65

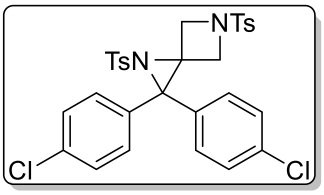

Prepared following **General Procedure 4** from *N*-(bis(4-chlorophenyl)methylene)-4-methylbenzenesulfonamide **O** (38 mg, 0.095 mmol) and (4-chlorophenyl)(1-tosylazetidin-3-yl)(2,4,6-trimethoxyphenyl)sulfonium tetrafluoroborate **10** (75 mg, 0.12 mmol). Work-up affords the crude material with a quantitative ^{1}H-NMR yield of 69%. Purification by flash column chromatography (20% EtOAc/hexane) affords the title compound **65** (27 mg, 0.044 mmol, 46%) as a white solid.

^{1}H-NMR (400 MHz, $CDCl_3$): δ_H 7.81 – 7.76 (2H, m), 7.74 – 7.71 (2H, m), 7.40 (2H, d, *J* = 8.0 Hz), 7.33 (6H, dd, *J* = 8.7, 2.3 Hz), 7.30 – 7.24 (4H, m), 4.02 (4H, m), 2.54 (3H, s), 2.50 (3H, s,).

^{13}C{^{1}H}-NMR (101 MHz, $CDCl_3$): δ_C 144.9, 144.7, 137.0, 134.9, 133.9, 131.3, 130.0, 129.9, 129.7, 129.0, 128.6, 127.5, 58.5, 55.3, 51.0, 21.8, 21.8.

IR (neat, *v* cm^{-1}): 2928, 1596, 1491, 1333, 1160

HRMS(ESI+): *m/z* calcd for $C_{30}H_{26}{}^{35}Cl_2N_2O_4S_2Na$ $[M+Na]^+$ 635.0603, found 635.0609.

2-(4-Bromophenyl)-2-phenyl-1-tosyl-5-oxa-1-azaspiro[2.3]hexane, 66

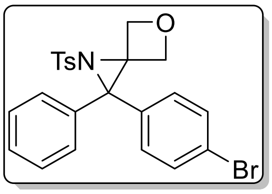

Prepared following **General Procedure 4** from *N*-((4-bromophenyl)(phenyl)methylene)-4-methylbenzenesulfonamide **AF** (39 mg, 0.095 mmol) and (4-chlorophenyl)(oxetan-3-yl)(2,4,6-trimethoxyphenyl)sulfonium tetrafluoroborate **9** (56 mg, 0.12 mmol). Work-up affords the crude material with a quantitative ^{1}H-NMR yield of 70%. Purification by flash column chromatography (20% Et_2O/hexane) affords the title compound **66** (11 mg, 0.023 mmol, 25%) as an white solid.

^{1}H-NMR (400 MHz, $CDCl_3$): δ_H 7.94 (2H, d, *J* = 8.3 Hz), 7.50 – 7.47 (2H, m), 7.45 – 7.33 (9H, m), 4.94 – 4.87 (2H, m), 4.81 (2H, dt, *J* = 8.4, 1.5 Hz), 2.48 (3H, s).

^{13}C{^{1}H}-NMR (101 MHz, $CDCl_3$): δ_C 145.0, 137.8, 135.9, 135.5, 131.9, 130.3, 130.1, 128.8, 128.7, 128.5, 127.8, 122.9, 75.6, 75.5, 59.1, 56.0, 21.8.

IR (neat, *v* cm^{-1}): 2952, 2874, 1597, 1330, 1161

HRMS(ESI+): *m/z* calcd for $C_{23}H_{20}{}^{79}BrNO_3SNa$ $[M+Na]^+$ 492.0239, found 492.0245.

2-(4-Bromophenyl)-2-phenyl-1,5-ditosyl-1,5-diazaspiro[2.3]hexane, 67

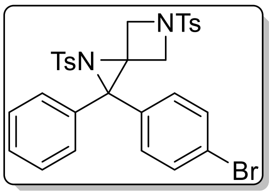

Prepared following **General Procedure 4** from *N*-((4-bromophenyl)(phenyl)methylene)-4-methylbenzenesulfonamide **AF** (41 mg, 0.095 mmol) and (4-chlorophenyl)(1-tosylazetidin-3-yl)(2,4,6-trimethoxyphenyl)sulfonium tetrafluoroborate **10** (75 mg, 0.12 mmol). Work-up affords the crude material with a quantitative ^{1}H-NMR yield of 70%. Purification by flash column chromatography (35% Et_2O/hexane) affords the title compound **67** (20 mg, 0.032 mmol, 34%) as a white solid.

^{1}H-NMR (400 MHz, $CDCl_3$): δ_H 7.78 – 7.74 (2H, m), 7.72 – 7.68 (2H, m), 7.47 – 7.43 (2H, m), 7.40 – 7.36 (2H, m), 7.34 – 7.26 (7H, m), 7.22 – 7.18 (2H, m), 4.09 – 3.93 (4H, m), 2.51 (3H, s), 2.47 (3H, s).

^{13}C{^{1}H}-NMR (101 MHz, $CDCl_3$): δ_C 144.9, 144.8, 137.4, 135.4, 135.0, 131.9, 131.4, 130.2, 130.0, 128.8, 128.8, 128.4, 127.6, 123.0, 59.4, 55.6, 55.5, 51.0, 21.9, 21.9.

IR (neat, *v* cm^{-1}): 2924, 1596, 1331, 1158, 1087.

HRMS(ESI+): *m/z* calcd for $C_{30}H_{27}{}^{81}BrN_2O_4S_2Na$ $[M+Na]^+$ 647.0470, found 647.0479

2-Phenyl-2-(4-(*p*-tolylthio)phenyl)-1-tosyl-5-oxa-1-azaspiro[2.3]hexane, 68

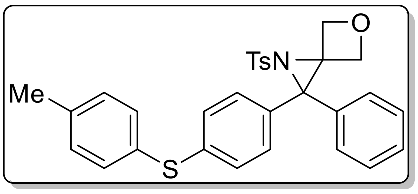

Prepared following **General Procedure 4** from 4-methyl-*N*-(phenyl(4-(*p*-tolylthio)phenyl)methylene)benzenesulfonamide **AG** (45 mg, 0.095 mmol) and (4-chlorophenyl)(oxetan-3-yl)(2,4,6-trimethoxyphenyl)sulfonium tetrafluoroborate **9** (56 mg, 0.12 mmol). Work-up affords the crude material with a quantitative ^{1}H-NMR yield of 64%. Purification by flash column chromatography (10% to 25% Et_2O/hexane) affords the title compound **68** (24 mg, 0.047 mmol, 49%) as a white solid.

^{1}H-NMR (400 MHz, $CDCl_3$): δ_H 7.97 – 7.89 (2H, m), 7.47 – 7.42 (2H, m), 7.41 – 7.28 (9H, m), 7.20 – 7.11 (4H, m), 4.92 – 4.87 (2H, m), 4.81 (2H, ddd, J = 9.5, 8.2, 1.2 Hz), 2.48 (3H, s), 2.36 (3H, s).

^{13}C{^{1}H}-NMR (101 MHz, $CDCl_3$): δ_C 144.8, 138.8, 138.6, 138.0, 136.1, 134.1, 133.7, 130.4, 130.0, 129.6, 129.1, 128.7, 128.6, 128.6, 128.3, 127.8, 75.6, 59.5, 56.0, 21.8, 21.3.

IR (neat, *v* cm^{-1}): 2951, 2873, 1596, 1491, 1329

HRMS(ESI+): *m/z* calcd for $C_{30}H_{27}NO_3S_2Na$ $[M+Na]^+$ 536.1325, found 536.1342

2-Phenyl-2-(4-(*p*-tolylthio)phenyl)-1,5-ditosyl-1,5-diazaspiro[2.3]hexane, 69

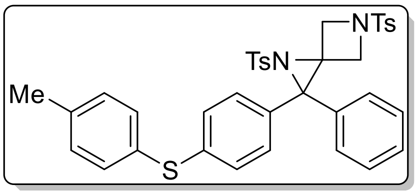

Prepared following **General Procedure 4** from 4-methyl-*N*-(phenyl(4-(*p*-tolylthio)phenyl)methylene)benzenesulfonamide **AG** (43 mg, 0.095 mmol) and (4-chlorophenyl)(1-tosylazetidin-3-yl)(2,4,6-trimethoxyphenyl)sulfonium tetrafluoroborate **10** (75 mg, 0.12 mmol). Work-up affords the crude material with a quantitative ^{1}H-NMR yield of 94%. Purification by flash column chromatography (30% to 40% Et_2O/hexane) affords the title compound **69** (32 mg, 0.048 mmol, 49%) as a white solid.

^{1}H-NMR (400 MHz, $CDCl_3$): δ_H 7.80 – 7.75 (2H, m), 7.74 – 7.70 (2H, m), 7.41 – 7.35 (4H, m), 7.35 – 7.29 (7H, m), 7.23 – 7.14 (4H, m), 7.14 – 7.08 (2H, m), 4.05 – 4.00 (4H, m), 2.52 (3H, s), 2.49 (3H, s), 2.39 (3H, s).

^{13}C{^{1}H}-NMR (101 MHz, $CDCl_3$): δ_C 139.0, 138.6, 137.4, 135.5, 133.7, 133.3, 131.3, 130.3, 129.9, 129.9, 129.3, 128.9, 128.6, 128.6, 128.5, 128.4, 128.1, 127.4, 125.0, 59.6, 55.5, 50.9, 40.5, 21.7, 21.7, 21.2.

IR (neat, *v* cm^{-1}): 3027, 2923, 1596, 1331, 1086.

HRMS(ESI+): *m/z* calcd for $C_{37}H_{32}N_2O_4S_3Na$ $[M+Na]^+$ 689.1573, found 689.1559.

1'-Tosyldispiro[fluorene-9,2'-aziridine-3',3''-oxetane], 70

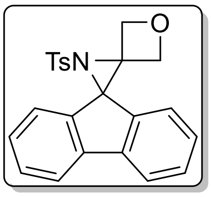

Prepared following **General Procedure 4** from *N*-(9*H*-fluoren-9-ylidene)-4-methylbenzenesulfonamide **AI** (31 mg, 0.095 mmol) and (4-chlorophenyl)(oxetan-3-yl)(2,4,6-trimethoxyphenyl)sulfonium tetrafluoroborate **9** (56 mg, 0.12 mmol). Work-up affords the crude material with a quantitative ^{1}H-NMR yield of 97%. Purification by flash column chromatography (30% Et_2O/hexane) affords the title compound **70** (22 mg, 0.056 mmol, 59%) as a yellow solid.

^{1}H-NMR (400 MHz, $CDCl_3$): δ_H 7.89 – 7.84 (2H, m), 7.78 (2H, m), 7.45 (2H, m), 7.35 – 7.29 (4H, m), 7.28 – 7.24 (2H, m), 5.46 (2H, d, *J* = 9.1 Hz), 4.86 (2H, d, *J* = 9.1 Hz), 2.45 (3H, s).

^{13}C{^{1}H}-NMR (101 MHz, $CDCl_3$): δ_C 144.6, 141.5, 137.9, 137.6, 130.0, 129.5, 127.7, 127.4, 124.4, 120.7, 75.6, 58.9, 56.2, 21.0.

IR (neat, *v* cm^{-1}): 3063, 2949, 2874, 1597, 1450

HRMS(ESI+): *m/z* calcd for $C_{23}H_{19}NO_3SNa$ $[M+Na]^+$ 412.0978, found 412.1007

1,1'-Ditosyldispiro[azetidine-3,2'-aziridine-3',9''-fluorene], 71

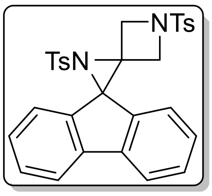

Prepared following **General Procedure 4** from *N*-(9*H*-fluoren-9-ylidene)-4-methylbenzenesulfonamide **AI** (31 mg, 0.095 mmol) and (4-chlorophenyl)(1-tosylazetidin-3-yl)(2,4,6-trimethoxyphenyl)sulfonium tetrafluoroborate **10** (75 mg, 0.12 mmol). Work-up affords the crude material with a quantitative ^{1}H-NMR yield of 85%. Purification by flash column chromatography (45% Et_2O/hexane) affords the title compound **71** (16 mg, 0.029 mmol, 31%) as a white solid.

A sample suitable for x-ray crystallography was obtained by slow evaporation from ethyl acetate.

^{1}H-NMR (400 MHz, $CDCl_3$): δ_H 7.72 (4H, m), 7.46 (4H, m), 7.34 – 7.27 (4H, m), 7.19 (2H, m), 6.96 (2H, d, *J* = 7.9 Hz), 4.79 – 4.74 (2H, m), 4.12 – 4.07 (2H, m), 2.47 (3H, s), 2.37 (3H, s).

^{13}C{^{1}H}-NMR (101 MHz, $CDCl_3$): δ_C 144.6, 144.0, 141.2, 137.5, 136.8, 130.7, 129.9, 129.5, 129.4, 127.9, 127.6, 127.1, 124.1, 120.3, 58.5, 55.4, 51.0, 21.7.

IR (neat, *v* cm^{-1}): 2924, 1596, 1450, 1339, 1160

HRMS(ESI+): *m/z* calcd for $C_{30}H_{26}N_2O_4S_2Na$ $[M+Na]^+$ 565.1226, found 565.1275.

2,2-Di-*p*-tolyl-1-tosyl-5-oxa-1-azaspiro[2.3]hexane, 72

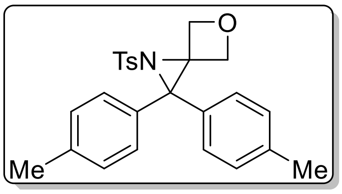

Prepared following **General Procedure 4** from *N*-(di-*p*-tolylmethylene)-4-methylbenzenesulfonamide **AH** (35 mg, 0.095 mmol) and (4-chlorophenyl)(oxetan-3-yl)(2,4,6-trimethoxyphenyl)sulfonium tetrafluoroborate **9** (56 mg, 0.12 mmol). Work-up affords the crude material with a quantitative ^{1}H-NMR yield of 65%. Purification by flash column chromatography (20% Et_2O/hexane) affords the title compound **72** (23 mg, 0.055 mmol, 58%) as a white solid.

^{1}H-NMR (400 MHz, $CDCl_3$): δ_H 7.96 – 7.93 (2H, m), 7.38 (2H, d, *J* = 8.1 Hz), 7.35 – 7.30 (4H, m), 7.14 (4H, d, *J* = 7.9 Hz), 4.94 – 4.88 (2H, m), 4.86 – 4.81 (2H, m), 2.48 (3H, s), 2.33 (6H, s).

^{13}C{^{1}H}-NMR (101 MHz, $CDCl_3$): δ_C 144.6, 138.2, 138.2, 133.7, 130.0, 129.3, 128.4, 127.8, 75.8, 59.8, 56.0, 21.8, 21.3.

IR (neat, *v* cm^{-1}): 2951, 2873, 1329, 1159, 1088.

HRMS(ESI+): *m/z* calcd for $C_{25}H_{25}NO_3SNa$ [M+Na]$^+$ 442.1447, found 442.1457.

2,2-Di-*p*-tolyl-1,5-ditosyl-1,5-diazaspiro[2.3]hexane, 73

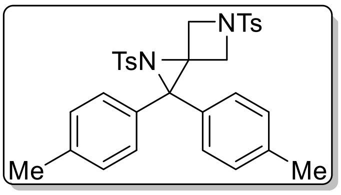

Prepared following **General Procedure 4** from *N*-(di-*p*-tolylmethylene)-4-methylbenzenesulfonamide **AH** (34 mg, 0.095 mmol) and (4-chlorophenyl)(1-tosylazetidin-3-yl)(2,4,6-trimethoxyphenyl)sulfonium tetrafluoroborate **10** (75 mg, 0.12 mmol). Work-up affords the crude material with a quantitative ^{1}H-NMR yield of 75%. Purification by flash column chromatography (20% EtOAc/hexane) affords the title compound **73** (6 mg, 0.010 mmol, 11%) as a white solid.

^{1}H-NMR (400 MHz, $CDCl_3$): δ_H 7.79 – 7.75 (2H, m), 7.71 – 7.67 (2H, m), 7.38 (2H, dd, J = 8.0, 3.7 Hz), 7.30 – 7.27 (2H, m), 7.17 – 7.13 (4H, m), 7.10 (4H, d, J = 8.2 Hz), 4.02 (4H, s), 2.51 (3H, s), 2.47 (3H, s), 2.31 (6H, s).

^{13}C{^{1}H}-NMR (101 MHz, $CDCl_3$): δ_C 144.6, 144.6, 138.4, 137.8, 133.1, 131.5, 130.0, 129.9, 129.3, 128.8, 128.3, 127.6, 60.1, 57.1, 55.8, 51.0, 21.9, 21.9, 21.3.

IR (neat, *v* cm^{-1}): 2924, 1596, 1511, 1160, 1088

HRMS(ESI+): *m/z* calcd for $C_{32}H_{32}N_2O_4S_2Na$ $[M+Na]^+$ 595.1700, found 595.1770.

2,2-Diphenyl-1-((4-(5-(*p*-tolyl)-3-(trifluoromethyl)-1*H*-pyrazol-1-yl)phenyl)sulfonyl)-5-oxa-1-azaspiro[2.3]hexane, 74

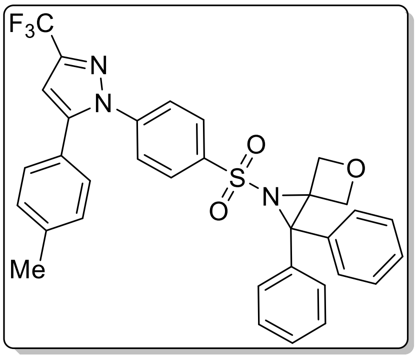

Prepared following **General Procedure 4** from *N*-(diphenylmethylene)-4-(5-(*p*-tolyl)-3-(trifluoromethyl)-1*H*-pyrazol-1-yl)benzenesulfonamide **AJ** (51 mg, 0.095 mmol) and (4-chlorophenyl)(oxetan-3-yl)(2,4,6-trimethoxyphenyl)sulfonium tetrafluoroborate **9** (56 mg, 0.12 mmol). Work-up affords the crude material with a quantitative ^{1}H-NMR yield of 68%. Purification by flash column chromatography (25% Et_2O/hexane) affords the title compound **74** (26 mg, 0.043 mmol, 45%) as a white solid.

^{1}H-NMR (400 MHz, $CDCl_3$): δ_H 8.08 – 8.02 (2H, m), 7.61 – 7.54 (2H, m), 7.45 – 7.40 (4H, m), 7.38 – 7.32 (6H, m), 7.18 (2H, d, *J* = 8.1 Hz), 7.13 (2H, d, *J* = 8.2 Hz), 6.76 (1H, s), 4.91 (2H, d, *J* = 8.9 Hz), 4.87 (2H, d, *J* = 1.2 Hz), 2.38 (3H, s).

^{19}F-NMR (377 MHz, $CDCl_3$): δ_F -62.49 (s).

^{13}C{^{1}H}-NMR (101 MHz, $CDCl_3$): δ_C 145.5, 144.4 (q, *J* = 38.7 Hz), 140.1, 140.1, 136.1, 130.0, 128.9, 128.9, 128.7, 128.7, 128.5, 125.8, 125.7, 121.2 (q, *J* = 269.2 Hz), 106.6 (br. s), 75.5, 60.5, 56.6, 21.5

IR (neat, *v* cm^{-1}): 2953, 1596, 1471, 1236, 1161

HRMS(ESI+): *m/z* calcd for $C_{33}H_{26}F_3N_3O_3SNa$ [M+Na]$^+$ 624.1539, found 624.1549

2,2-diphenyl-1-((4-(5-(*p*-tolyl)-3-(trifluoromethyl)-1*H*-pyrazol-1-yl)phenyl)sulfonyl)-5-tosyl-1,5-diazaspiro[2.3]hexane, 75

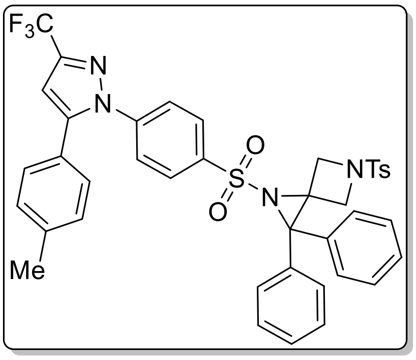

Prepared following **General Procedure 4** from *N*-(diphenylmethylene)-4-(5-(*p*-tolyl)-3-(trifluoromethyl)-1*H*-pyrazol-1-yl)benzenesulfonamide **AJ** (54 mg, 0.095 mmol) and (4-chlorophenyl)(1-tosylazetidin-3-yl)(2,4,6-trimethoxyphenyl)sulfonium tetrafluoroborate **10** (75 mg, 0.12 mmol). Work-up affords the crude material with a quantitative ^{1}H-NMR yield of 77%. Purification by flash column chromatography (35% Et_2O/hexane) affords the title compound **75** (23 mg, 0.030 mmol, 32%) as a white solid.

A sample suitable for x-ray crystallography was obtained by slow evaporation from ethyl acetate.

^{1}H-NMR (400 MHz, $CDCl_3$): δ_H 7.87 – 7.82 (2H, m), 7.69 – 7.63 (2H, m), 7.48 – 7.43 (2H, m), 7.38 – 7.30 (12H, m), 7.20 (2H, d, *J* = 8.0 Hz), 7.15 – 7.09 (2H, m), 6.78 (1H, s), 4.08 – 4.01 (2H, m), 3.99 – 3.94 (2H, m), 2.39 (6H, d, *J* = 4.2 Hz).

^{19}F-NMR (377 MHz, $CDCl_3$): δ_F -62.44 (s).

^{13}C{^{1}H}-NMR (101 MHz, $CDCl_3$): δ_C 145.5, 144.9, 144.5 (q, *J* = 38.7 Hz), 143.3, 140.2, 139.7, 135.6, 131.2, 130.1, 130.0, 128.9, 128.8, 128.7, 128.5, 128.4, 125.7, 125.6, 121.2 (q, *J* = 268.9 Hz), 106.7 (br. s), 60.9, 55.6, 51.4, 21.6, 21.5

IR (neat, *v* cm^{-1}): 3061, 1596, 1338, 1236, 1158, 1096

HRMS(ESI+): *m/z* calcd for $C_{40}H_{33}F_3N_4O_4S_2Na$ $[M+Na]^+$ 777.1793, found 777.1793.

Isopropyl 2-(4-(2-(4-chlorophenyl)-1-((4-(5-(*p*-tolyl)-3-(trifluoromethyl)-1*H*-pyrazol-1-yl)phenyl)sulfonyl)-5-oxa-1-azaspiro[2.3]hexan-2-yl)phenoxy)-2-methylpropanoate, 76

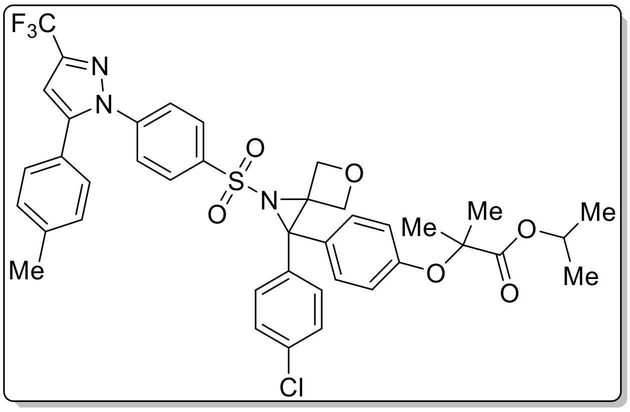

Prepared following **General Procedure 4** from isopropyl 2-(4-((4-chlorophenyl)(((4-(5-(*p*-tolyl)-3-(trifluoromethyl)-1*H*-pyrazol-1-yl)phenyl)sulfonyl)imino)methyl)phenoxy)-2-methylpropanoate **AK** (69 mg, 0.095 mmol) and (4-chlorophenyl)(oxetan-3-yl)(2,4,6-trimethoxyphenyl)sulfonium tetrafluoroborate **9** (56 mg, 0.12 mmol). Work-up affords the crude material with a quantitative ^{1}H-NMR yield of 75%. Purification by flash column chromatography (30% Et_2O/hexane) affords the title compound **76** (25 mg, 0.032 mmol, 34%) as a white solid.

^{1}H-NMR (400 MHz, $CDCl_3$): δ_H 8.05 – 7.97 (2H, m), 7.60 – 7.54 (2H, m), 7.31 (4H, s), 7.19 (4H, dd, *J* = 8.4, 1.5 Hz), 7.16 – 7.09 (2H, m), 6.81 – 6.74 (3H, m), 5.05 (1H, hept, *J* = 6.5 Hz), 4.88 (2H, dd, *J* = 8.5, 3.9 Hz), 4.78 (2H, td, *J* = 8.4, 1.1 Hz), 2.39 (3H, s), 1.59 (6H, s), 1.17 (6H, d, *J* = 6.3 Hz).

^{19}F-NMR (377 MHz, $CDCl_3$): δ_F -62.51(s)

^{13}C{^{1}H}-NMR (101 MHz, $CDCl_3$): δ_C 173.5, 156.1, 144.5 (q, *J* = 38.9 Hz), 143.5, 140.1, 139.9, 134.8, 132.7, 130.0, 129.4, 129.2, 128.9, 128.9, 128.8, 128.4, 127.2, 125.8, 125.7, 118.5, 106.7 (br. s), 79.4, 75.5, 75.4, 69.2, 59.4, 56.7, 25.6, 25.5, 21.7, 21.5. *Note:* *C*F_3 not clearly resolved

Note: The ^{13}C{^{1}H}-NMR spectrum reveals an unresolved co-eluting impurity which was not removed even with additional column chromatography.

IR (neat, *v* cm^{-1}): 2982, 1728, 1596, 1237, 1161

HRMS(ESI+): *m/z* calcd for $C_{40}H_{37}{}^{35}ClF_3N_3O_6SNa$ $[M+Na]^+$ 802.1941, found 802.1961

Isopropyl 2-(4-(2-(4-chlorophenyl)-1-((4-(5-(*p*-tolyl)-3-(trifluoromethyl)-1*H*-pyrazol-1-yl)phenyl)sulfonyl)-5-tosyl-1,5-diazaspiro[2.3]hexan-2-yl)phenoxy)-2-methylpropanoate, 77

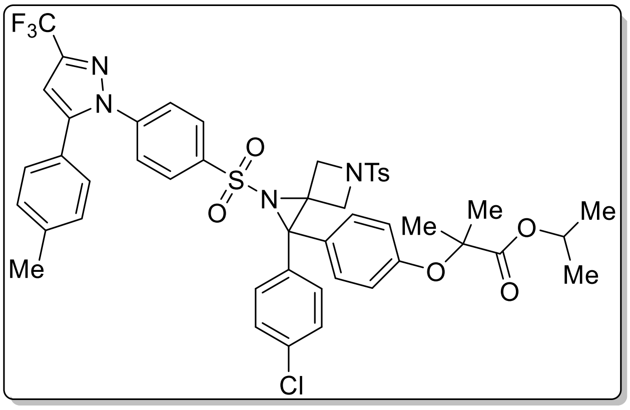

Prepared following **General Procedure 4** from isopropyl 2-(4-((4-chlorophenyl)(((4-(5-(*p*-tolyl)-3-(trifluoromethyl)-1*H*-pyrazol-1-yl)phenyl)sulfonyl)imino)methyl)phenoxy)-2-methylpropanoate **AK** (69 mg, 0.095 mmol) and (4-chlorophenyl)(1-tosylazetidin-3-yl)(2,4,6-trimethoxyphenyl)sulfonium tetrafluoroborate **10** (75 mg, 0.12 mmol). Work-up affords the crude material with a quantitative ^{1}H-NMR yield of 92%. Purification by flash column chromatography (15% EtOAc/hexane) affords the title compound **77** (25 mg, 0.027 mmol, 28%) as a white solid.

^{1}H-NMR (400 MHz, CDCl$_3$): δ_H 7.81 (2H, d, *J* = 8.7 Hz), 7.66 (2H, d, *J* = 8.3 Hz), 7.48 – 7.42 (2H, m), 7.34 (2H, d, *J* = 8.0 Hz), 7.32 – 7.27 (2H, m), 7.24 – 7.18 (4H, m), 7.14 – 7.10 (4H, m), 6.77 (3H, d, *J* = 8.7 Hz), 5.06 (1H, p, *J* = 6.2 Hz), 4.01 – 3.92 (4H, m), 2.39 (6H, d, *J* = 3.7 Hz), 1.58 (6H, s), 1.20 – 1.17 (6H, m).

^{19}F-NMR (377 MHz, CDCl$_3$): δ_F -62.45 (s).

^{13}C{^{1}H}-NMR (101 MHz, CDCl$_3$): δ_C 173.4, 156.2, 145.5, 145.0, 144.5 (q, *J* = 38.7 Hz), 143.3, 140.2, 139.5, 134.9, 134.3, 131.1, 130.1, 130.0, 129.9, 129.3, 129.0, 128.8, 128.7, 128.5, 127.9, 125.7, 125.6, 121.2 (d, *J* = 269.4 Hz), 118.5, 106.7 (br. s), 79.4, 69.2, 66.0, 59.7, 55.4, 51.6, 25.6, 25.0, 21.7, 21.7, 21.6, 21.5

IR (neat, *v* cm^{-1}): 2984, 1728, 1596, 1236, 1159

HRMS (ESI+): *m/z* calcd for $C_{47}H_{44}{}^{35}ClF_3N_4O_7S_2Na$ [M+Na]$^+$ 955.2189, found 955.2268.

Methyl 1-phenyl-5-tosyl-5-azaspiro[2.3]hexane-1-carboxylate, 79

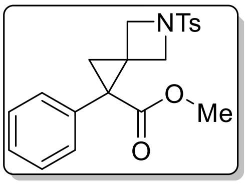

Prepared following **General Procedure 4** from methyl 2-phenylacrylate **K** (23 mg, 0.14 mmol) and (4-chlorophenyl)(1-tosylazetidin-3-yl)(2,4,6-trimethoxyphenyl)sulfonium tetrafluoroborate **10** (109 mg, 0.18 mmol). Work-up affords the crude material with a quantitative ^{1}H-NMR yield of 75%. Purification by flash column chromatography (50% DCM/hexane to 40% EtOAc/hexane) affords the title compound **79** (13 mg, 0.035 mmol, 25%) as a white solid.

^{1}H-NMR (400 MHz, $CDCl_3$): δ_H 7.81 – 7.70 (2H, m), 7.41 (2H, dt, *J* = 7.9, 0.7 Hz), 7.27 (3H, tt, *J* = 4.0, 2.7 Hz), 6.96 – 6.89 (2H, m), 4.14 – 4.03 (2H, m), 3.58 (4H, d, *J* = 7.7 Hz), 3.45 (1H, dd, *J* = 9.0, 0.8 Hz), 2.51 (3H, s), 1.77 (1H, d, *J* = 5.6 Hz), 1.45 (1H, d, *J* = 5.6 Hz).

^{13}C{^{1}H}-NMR (101 MHz, $CDCl_3$): δ_C 171.9, 144.4, 134.2, 132.0, 130.5, 129.9, 128.7, 128.6, 128.0, 57.2, 55.2, 52.6, 35.8, 28.9, 23.8, 21.8.

IR (neat, *v* cm^{-1}): 2951, 1715, 1345, 1235, 1157

HRMS(ESI+): *m/z* calcd for $C_{20}H_{21}NO_4SNa$ $[M+Na]^+$ 394.1089, found 394.1085.

1-Methylazetidin-3-yl 1-phenyl-5-tosyl-5-azaspiro[2.3]hexane-1-carboxylate, 81

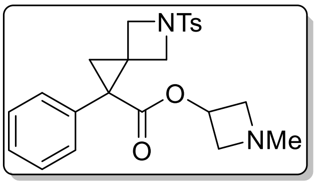

Prepared following **General Procedure 4** from 1-methylazetidin-3-yl 2-phenylacrylate **L** (43 mg, 0.19 mmol) and (4-chlorophenyl)(1-tosylazetidin-3-yl)(2,4,6-trimethoxyphenyl)sulfonium tetrafluoroborate **10** (152 mg, 0.24 mmol). Work-up affords the crude material with a quantitative ^{1}H-NMR yield of 32%. Purification by flash column chromatography (50% DCM/hexane to 5% MeOH/DCM) affords the title compound **81** (15 mg, 0.035 mmol, 19%) as a white solid.

^{1}H-NMR (400 MHz, $CDCl_3$): δ_H 7.81 – 7.70 (2H, m), 7.42 (2H, d, *J* = 8.0 Hz), 7.27 (3H, dd, *J* = 6.0, 3.0 Hz), 6.99 – 6.88 (2H, m), 4.98 – 4.92 (1H, m), 4.06 (1H, d, *J* = 5.5 Hz), 3.83 – 3.71 (2H, m), 3.57 (2H, d, *J* = 9.0 Hz), 3.44 (1H, d, *J* = 9.1 Hz), 3.01 – 2.91 (2H, m), 2.51 (3H, s), 2.38 (3H, s), 1.78 (1H, d, *J* = 5.7 Hz), 1.49 (1H, d, *J* = 5.7 Hz).

^{13}C{^{1}H}-NMR (101 MHz, $CDCl_3$): δ_C 170.7, 144.4, 133.7, 131.9, 130.5, 130.0, 128.7, 128.6, 128.1, 64.5, 62.4, 62.1, 57.0, 55.2, 45.2, 35.6, 29.3, 23.8, 21.8.

IR (neat, *v* cm^{-1}): 2941, 1716, 1345, 1229, 1158.

HRMS(ESI+): *m/z* calcd for $C_{23}H_{27}N_2O_4S$ $[M+H]^+$ 427.1691, found 427.1693.

8. Additional Supporting Experiments

8.1. Carbene Pathway

To study if the reaction potentially proceeded through a carbene intermediate, we used a series of electronically diverse styrene analogues as electrophiles. In line with related reports,[16,17] we hypothesized that a carbene intermediate would preferentially react with electronically rich styrene analogues, whereas an ylid intermediate would be unreactive with electron-rich derivatives, and preferentially react with electron-deficient analogues. Our experimental results indicate, based on the observed reactivity, that a carbene intermediate is unlikely.

1)

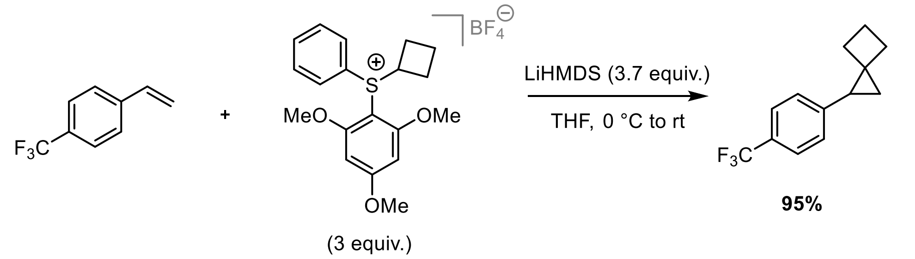

2)

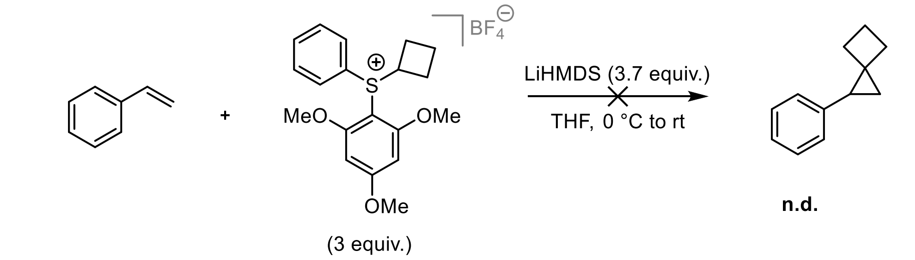

3)

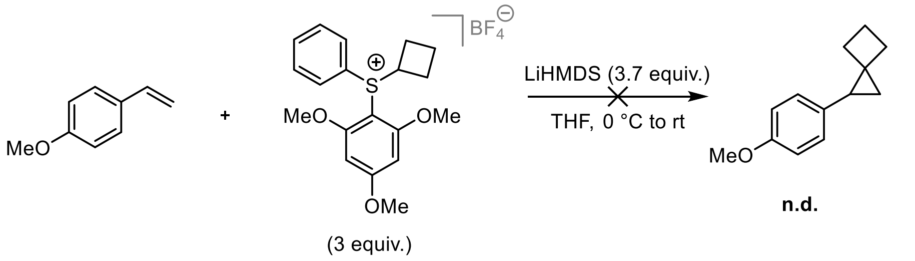

4) Competition Experiment

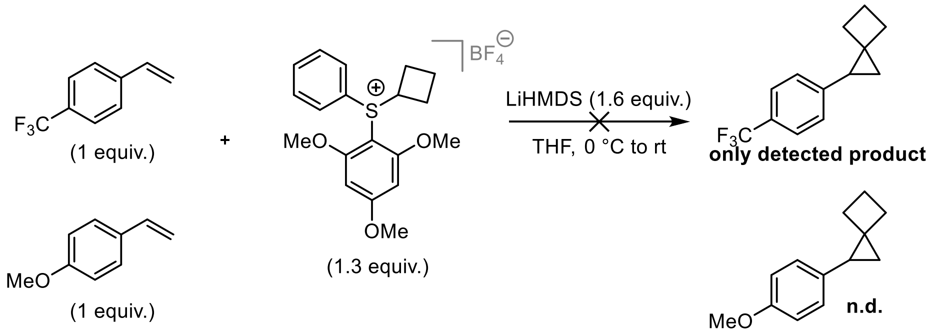

8.2. Reactivity Comparison

Intrigued by the successful reaction with 4-trifluoromethylstyrene, we wanted to investigate how this compared with known Johnson-Corey-Chaykovsky reagents. We thus tested the reactivity of trimethylsulfonium iodide with 4-trifluoromethylstyrene under our standard conditions. 4-Trifluoromethylstyrene was recovered exclusively; no product formation could be detected. This highlighted the carbenoid-like reactivity of our reagents, as also alkenes, which are not usually prone to undergo such reactivity, are successful substrates for cyclopropanation.

1)

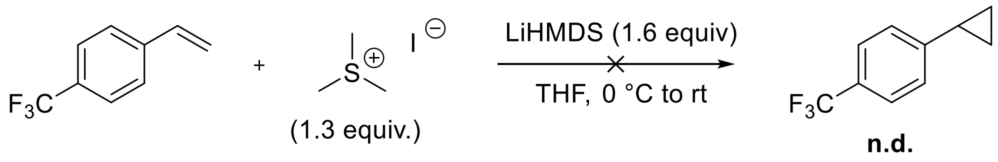

2)

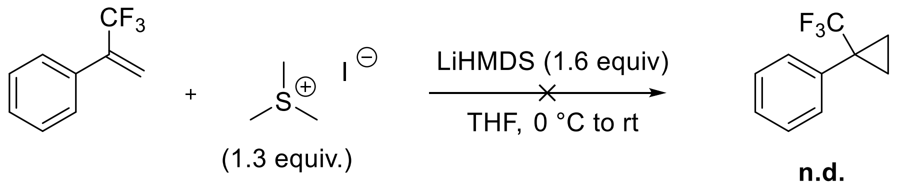

9. X-Ray Crystallography

The X-ray data were acquired at room temperature with a Bruker AXS X8 APEXII automated diffractometer, equipped with a CCD detector and graphite-monochromatized Mo Kα radiation (λ = 0.71073 Å) operating at 50 kV, 30 mA and 40 mm crystal-to-detector distance. Relevant crystallographic data of the investigated crystals is reported in **Table S2**. The whole Ewald sphere (±h,±k,±l) was recorded up to θ~30° with a scan width 0.8°/frame and an exposure time ranging from 120 to 240 s/frame. The APEX program suite allowed to optimize the collection strategy;[18] the SAINT package[19] was used for the integration of the intensities of reflections and the correction of the Lorentz and polarization effects; the SADABS software[20] was employed for the empirical absorption correction; XPREP[21] was used for the subsequent analysis of the intensity data and the assignment of the space group. Structure was solved by charge flipping method[22] and refined in $P2_1/c$ space group by full-matrix least-square analysis using the program CRYSTALS.[23] All reflections are included in the refinement. Overall scale factor, atomic positions and anisotropic atomic displacement parameters of -not-hydrogen atoms were refined. All hydrogen atoms were located and refined isotropically through ride restraints conditions.[24] The compound **75** showed positional disorder on the –CF_3 group, which was successfully modeled, as well as one molecule of ethyl acetate solvent in the unit cell with an occupancy of 0.88.

Table S1. X-ray crystal data of the studied crystals.

	**75**	**71**	**49**	**54**
Crystal size (mm)	0.59 × 0.37 × 0.03	0.45 × 0.33 × 0.13	0.55 × 0.52 × 0.14	0.58 × 0.30 × 0.05
Chemical formula	$C_{40}H_{33}F_{3.00}N_4O_4S_2$	$C_{30}H_{26}N_2O_4S_2$	$C_{19}H_{18}F_3NO_2S$	$C_{19}H_{17}BrF_3NO_2S$
M_r	754.84	542.68	381.42	460.31
Crystal system, space group	Triclinic, $P\bar{1}$	Triclinic, $P\bar{1}$	Monoclinic, $C2/c$	Monoclinic, $P2_1/c$
a, b, c (Å)	10.6928 (3), 13.2150 (4), 16.0077 (5)	10.2743 (4), 10.3274 (5), 13.5824 (5)	26.3339 (18), 9.6915 (7), 16.8433	8.8514 (6), 28.062 (2), 7.8311 (5)
α, β, γ (°)	73.945 (1), 76.710 (1), 87.792 (2)	82.838 (2), 88.739 (2), 68.553 (2)	90, 121.778 (2), 90	90, 92.261 (2), 90
V (Å^3)	2114.78 (11)	1330.49 (10)	3654.3 (4)	1943.6 (2)
$R[F^2 > 2\sigma(F^2)]$, $wR(F^2)$, S	0.079, 0.241, 095	0.074, 0.175, 0.60	0.047, 0.142, 0.91	0.058, 0.169, 1.03
$\Delta\rho_{max}$, $\Delta\rho_{min}$ (e Å^{-3})	0.74, -0.61	0.79, -1.03	0.32, -0.33	0.61, -0.66

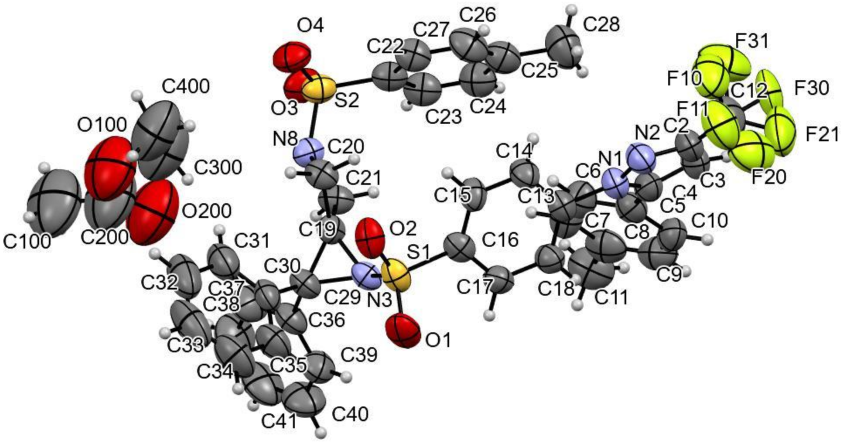

Figure S1. ORTEP drawing of compound **75**. The displacement ellipsoids are drawn at the 50% probability level. Hydrogen atoms labels are omitted for clarity.

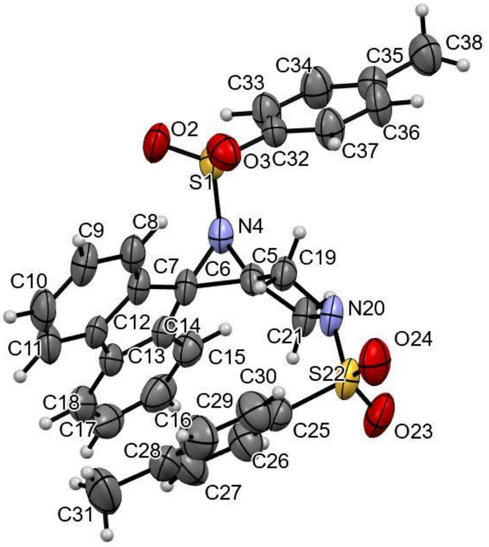

Figure S2. ORTEP drawing of compound **71**. The displacement ellipsoids are drawn at the 50% probability level. Hydrogen atoms labels are omitted for clarity.

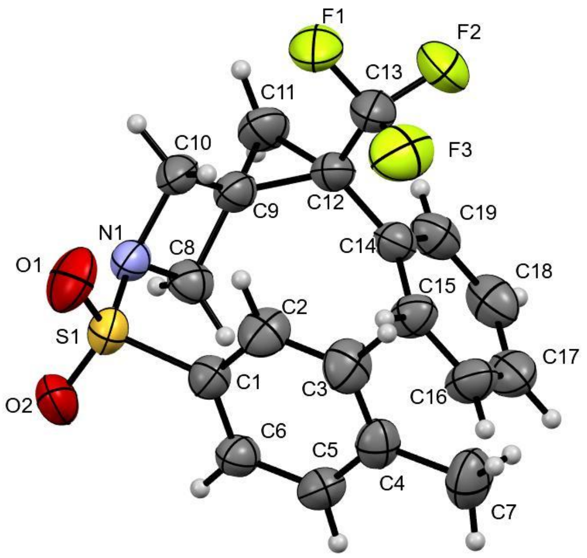

Figure S3. ORTEP drawing of compound **49**. The displacement ellipsoids are drawn at the 50% probability level. Hydrogen atoms labels are omitted for clarity.

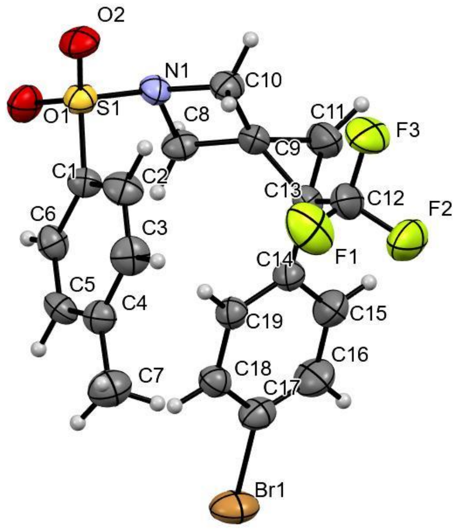

Figure S4. ORTEP drawing of compound **54**. The displacement ellipsoids are drawn at the 50% probability level. Hydrogen atoms labels are omitted for clarity.

10. Predictive Analytics-Supported Target Interaction

Using the *Predictive Analytics* tool on CAS BioFinder® (https://www.cas.org/solutions/biofinder-discovery-platform) several molecular structures were screened *in silico* for their predicted interaction (pAct). The calculation of pAct is based on the combination of an ensemble of multiple models based on different methodologies that are used together to generate the final prediction. These methodologies are:

- **Similarity-based method (SIM)**: A kNN (k nearest neighbors) method based on the weighted average of the affinities of molecules in the training set similar to the input molecule.
- **Similarity ensemble approach (SEA)**: Based on the distribution of similarities of the input molecule to all the training molecules that are active to a particular target. If this distribution is similar to the average distributions of all those molecules when compared against each other then the input molecule is considered active.
- **Simplest Active Substructure (SAS)**: Given a biological target, a SAS is defined as the simplest active subgraph containing the minimum pharmacophoric features to achieve activity. These subgraphs are detected using MMPs and multiple SAS can be defined for any given target reflecting the diversity of chemical series in active molecules. If the input molecule presents one or more SAS an affinity will be derived based on the affinities of the molecules in the training set presenting that same substructure.
- **Machine Learning Method (MLM)**: This methodology uses molecular descriptors to train machine learning models, with methods such as XGBoost and gNN, and produces estimated affinity values for the input molecule. For each target there is a classifier model that separates molecules into active and inactive and then for the active ones a secondary regressor model produces the predicted affinity value.
- **Cross-Pharmacology Index (XPI)**: This methodology is based on the activity profile of the input molecule including known data, if any, as well as the results produced by the other methods. Known and predicted annotations are used to impute missing target activities for targets related via cross-pharmacology.

All these methods are trained once with each of two different orthogonal molecular descriptors including ECFP4 and FPD, an inhouse implementation similar in concept to MACCS fingerprints. This means that in the end there are 12 models involved in a single prediction.

The final consensus takes into account the affinities predicted by the different methods together with their respective model metrics to aggregate them into a single affinity value. The confidence score is produced by an additional ML model that learns to project the native confidence scores of the different methodologies into the error space so that for a confidence score of 0.5 the expected error is of 0.5 log units or lower.

Before training the different models, input data is standardized and thoroughly deduplicated at the molecular fingerprint level. For model building a ratio of 10 to 1 between negatives and positives is forced on every model, adding random molecules as negatives if the original data does not have enough. Finally, a five-fold validation is used to generate the metrics of all the individual models as well as the consensus.

Using this model, the molecular structures were screened against a personalized set containing the following receptors

- Sigma non-opioid intracellular receptor 1 (Rattus horvegicus)
- Kappa-type opioid receptor (Homo sapiens)
- Delta-type opioid receptor (Homo sapiens)
- Mu-type opioid receptor (Homo sapiens)
- Mu-type opioid receptor (Cavia porcellus)
- Mu-type opioid receptor (Rattus norvegicus)

The ligands were subsequently ranked according to their predicted activity for the mu-type opioid receptor (Homo sapiens).

Table 1: Ligand-Target Predictions

Ligand	Target	Predicted pAct	Confidence
**78**	Sigma non-opioid intracellular receptor 1 (Rattus norvegicus)	8.53	0.36
**81**	Kappa-type opioid receptor (Homo sapiens)	7.68	0.39
**80**	Delta-type opioid receptor (Homo sapiens)	7.42	0.65
**47**	Delta-type opioid receptor (Homo sapiens)	7.38	0.63
**79**	Delta-type opioid receptor (Homo sapiens)	7.37	0.58
**81**	Mu-type opioid receptor (Homo sapiens)	7.22	0.43
**81**	Delta-type opioid receptor (Homo sapiens)	6.67	0.67
**79**	Kappa-type opioid receptor (Homo sapiens)	6.64	0.42
**80**	Kappa-type opioid receptor (Homo sapiens)	6.63	0.47

**47**	Kappa-type opioid receptor (Homo sapiens)	6.62	0.46
**47**	Mu-type opioid receptor (Homo sapiens)	6.54	0.51
**79**	Mu-type opioid receptor (Homo sapiens)	6.50	0.47
**80**	Mu-type opioid receptor (Homo sapiens)	6.48	0.52
**78**	Mu-type opioid receptor (Cavia porcellus)	6.35	0.44
**78**	Sigma non-opioid intracellular receptor 1 (Cavia porcellus)	6.10	0.36
**78**	Mu-type opioid receptor (Homo sapiens)	5.92	0.64
**78**	Kappa-type opioid receptor (Homo sapiens)	5.63	0.60
**17**	Sigma non-opioid intracellular receptor 1 (Cavia porcellus)	5.05	0.53
**78**	Delta-type opioid receptor (Homo sapiens)	5.00	0.70
**78**	Mu-type opioid receptor (Rattus norvegicus)	4.95	0.52
**82**	No predicted activity for this ligand against panel targets	-	-
**46**	No predicted activity for this ligand against panel targets	-	-

11. In vitro studies

Cell Culture: Human SH-SY5Y cells (ATCC® CRL-2266™) were cultured in Eagle's Minimum Essential Medium (EMEM; ATCC® 30-2003™) supplemented with 10% fetal bovine serum (FBS; Gibco, USA) and 1% penicillin-streptomycin (Thermo Fisher Scientific, USA). Cells were maintained at 37 °C in a humidified incubator with 5% CO_2.

Label-Free Assay: Label-free measurements were performed using the EnSpire™ instrument (PerkinElmer, USA). Briefly, SH-SY5Y cells were seeded at 30,000 cells per well in a 384-well optical sensor plate and allowed to adhere for 24 h. Prior to compound stimulation, cells were equilibrated in HBSS buffer supplemented with 10 mM HEPES for 1 h. Stock solutions of test compounds were prepared in DMSO, and dispensed at final assay concentrations ranging from 0.03 to 150 µM using an HP-D300 Digital Dispenser (Tecan Trading AG, Switzerland). Each well's response was recorded in picometers (pm) and normalized to the baseline signal. DAMGO was included as a positive control.

Data Analysis: Concentration vs response curves were fitted using a log(agonist) vs. response model with variable slope (four-parameter logistic) in GraphPad Prism software (GraphPad Software, USA).

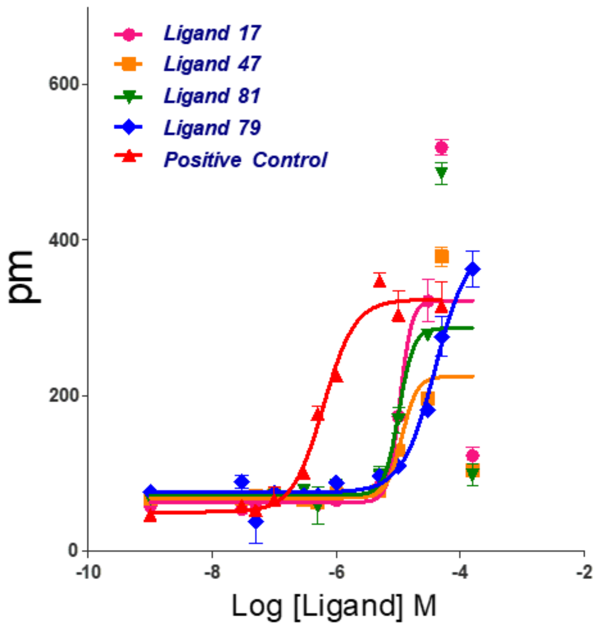

12. Computational Studies

12.1. GitHub Repository

All code, raw data and additional information required to reproduce the results of the computational studies described in **12.2. Calculation of Gibbs Free Energy for Carbene Formation** and **12.3. Bioisostere Identification through Clustering Approach** are available on GitHub at the following URL: https://github.com/f48r1/spirohexane4bioisostere

12.2. Calculation of Gibbs Free Energy for Carbene Formation

Organic reactions leading to the formation of a carbene structure among the chemical products were considered to determine the DG reaction computationally (Figure 1).

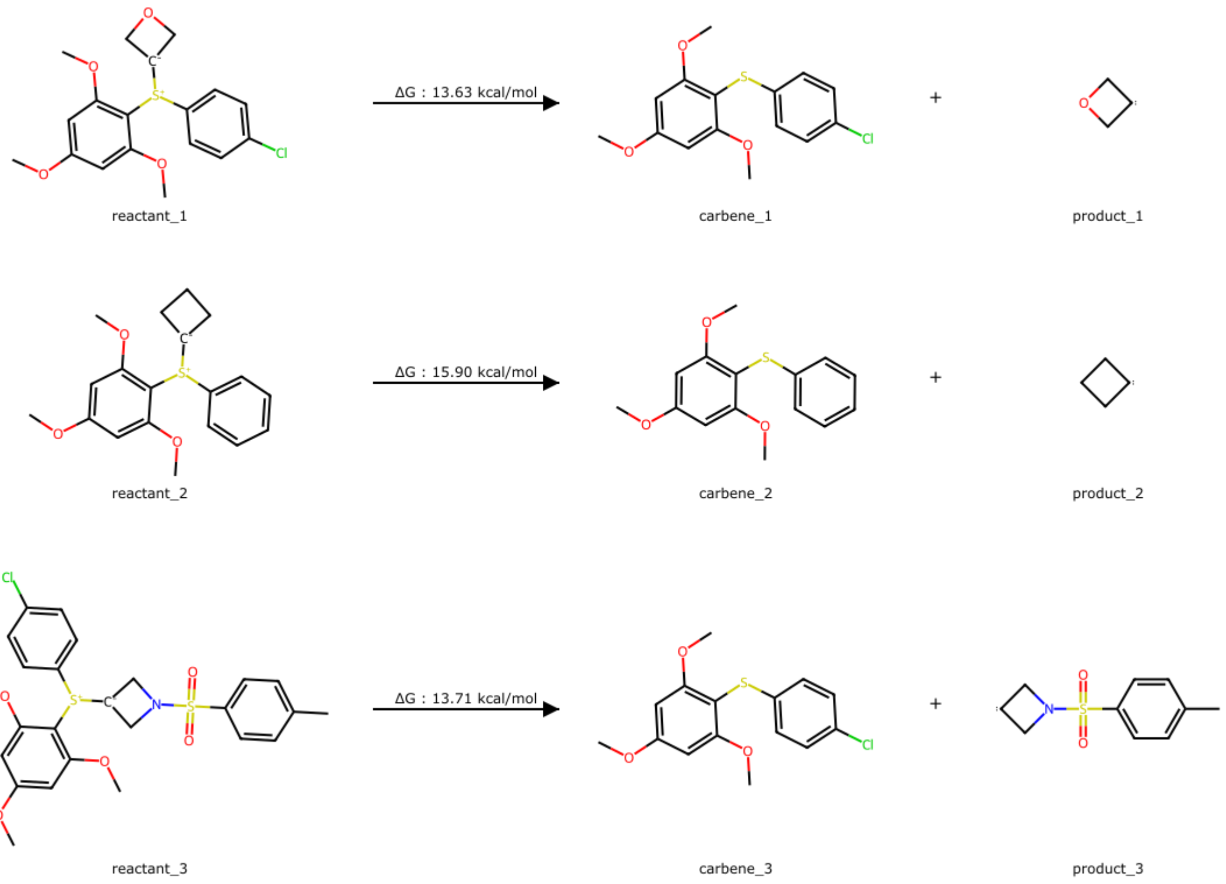

Figure 1. Gibbs free energy of carbene formation computationally calculated for 3 organic reactions.

Specifically, ORCA (version 5.0.4) software has been employed for Geometry optimizations and vibrational frequency calculations using the M06-2X meta-hybrid density functional in combination with the def2-SVP basis set for all atoms. Tight self-consistent field convergence criteria (TightSCF) were applied to improve the robustness and accuracy of the electronic structure convergence. Solvent effects were included through the CPCM implicit solvation model, using tetrahydrofuran (THF) as the dielectric medium. All optimized structures were confirmed to be true minima by the absence of imaginary frequencies in the subsequent

harmonic vibrational analysis. All carbene species were considered in their singlet spin state. Final Gibbs free energy (as reported in each file output attached below) was converted into *kcal/mol* units and employed for chemicals.

Gibbs free energies of carbene formation were calculated as follows:

$$\Delta G_i = G(carbene\_i) + G(product\_i) - G(reactant\_i)$$

where *i* corresponds to reaction index.

Reactant_1

```
--------
ENTHALPY
--------
The enthalpy is H = U + kB*T
                kB is Boltzmann's constant
Total free energy                 ...  -1854.67076843 Eh
Thermal Enthalpy correction       ...      0.00094421 Eh       0.59 kcal/mol
-----------------------------------------------------------------------
Total Enthalpy                    ...  -1854.66982422 Eh

Note: Only C1 symmetry has been detected, increase convergence thresholds
      if your molecule has a higher symmetry. Symmetry factor of 1.0 is
      used for the rotational entropy correction.

Note: Rotational entropy computed according to Herzberg
Infrared and Raman Spectra, Chapter V,1, Van Nostrand Reinhold, 1945
Point Group:  C1, Symmetry Number:   1
Rotational constants in cm-1:     0.008597     0.004619     0.003974

Vibrational entropy computed according to the QRRHO of S. Grimme
Chem.Eur.J. 2012 18 9955

-------
ENTROPY
-------

The entropy contributions are T*S = T*(S(el)+S(vib)+S(rot)+S(trans))
     S(el)   - electronic entropy
     S(vib)  - vibrational entropy
     S(rot)  - rotational entropy
     S(trans)- translational entropy
The entropies will be listed as multiplied by the temperature to get
units of energy

Electronic entropy                ...      0.00000000 Eh      0.00 kcal/mol
Vibrational entropy               ...      0.03725204 Eh     23.38 kcal/mol
Rotational entropy                ...      0.01690466 Eh     10.61 kcal/mol
Translational entropy             ...      0.02071212 Eh     13.00 kcal/mol
-----------------------------------------------------------------------
Final entropy term                ...      0.07486882 Eh     46.98 kcal/mol

In case the symmetry of your molecule has not been determined correctly
or in case you have a reason to use a different symmetry number we print
out the resulting rotational entropy values for sn=1,12 :
 --------------------------------------------------------
|  sn= 1 | S(rot)=       0.01690466 Eh     10.61 kcal/mol|
|  sn= 2 | S(rot)=       0.01625020 Eh     10.20 kcal/mol|
|  sn= 3 | S(rot)=       0.01586737 Eh      9.96 kcal/mol|
|  sn= 4 | S(rot)=       0.01559574 Eh      9.79 kcal/mol|
|  sn= 5 | S(rot)=       0.01538506 Eh      9.65 kcal/mol|
|  sn= 6 | S(rot)=       0.01521291 Eh      9.55 kcal/mol|
|  sn= 7 | S(rot)=       0.01506737 Eh      9.45 kcal/mol|
|  sn= 8 | S(rot)=       0.01494129 Eh      9.38 kcal/mol|
|  sn= 9 | S(rot)=       0.01483008 Eh      9.31 kcal/mol|
|  sn=10 | S(rot)=       0.01473060 Eh      9.24 kcal/mol|
|  sn=11 | S(rot)=       0.01464061 Eh      9.19 kcal/mol|
|  sn=12 | S(rot)=       0.01455846 Eh      9.14 kcal/mol|
 --------------------------------------------------------

-------------------
GIBBS FREE ENERGY
-------------------

The Gibbs free energy is G = H - T*S

Total enthalpy                    ...  -1854.66982422 Eh
Total entropy correction          ...     -0.07486882 Eh    -46.98 kcal/mol
-----------------------------------------------------------------------
Final Gibbs free energy         ...  -1854.74469304 Eh

For completeness - the Gibbs free energy minus the electronic energy
G-E(el)                           ...      0.28918513 Eh    181.47 kcal/mol
```

```
43
Coordinates from ORCA-job file
  C   3.03450204335741      0.17976985189716     -3.97796273024957
  O   3.41432454193950     -0.02108662283047     -2.63231054652083
  C   2.46522551630109     -0.01180756148494     -1.68080479866971
  C   2.92376695155898     -0.21017824853707     -0.36997285625879
  C   2.00855271847908     -0.21104441432372      0.68093813917097
  O   2.35753320360071     -0.37924282300761      1.96780530454113
  C   3.72478549545659     -0.54433477524743      2.28602842662889
  C   0.63519407379963     -0.02960400273823      0.42217164600512
  S  -0.48401953939758      0.13384462397779      1.82272493041062
  C  -1.77761935013895     -1.02178837887054      1.31293468790629
  C  -1.41368480604875     -2.30175666225828      0.89764235315872
  C  -2.40327623899417     -3.21695571052754      0.54756194411319
  C  -3.74220406533486     -2.83226287519900      0.62475065493994
  Cl -4.98200039922776     -3.96993354236468      0.17662799864386
  C  -4.10721815278251     -1.55791863274313      1.05372904042063
  C  -3.11212645020482     -0.64659872776632      1.40617022811575
  C  -1.15803488876840      1.66146314902061      1.98475625438133
  C  -1.21597155613977      2.72630380781859      0.90008254159146
  O  -0.68141913209342      3.70174178398628      1.80724392274353
  C  -0.44551346388907      2.71926112608250      2.82636379426137
  C   0.19621086847720      0.18088093840024     -0.89446837805513
  O  -1.12355876304797      0.36519778614592     -1.05576832063780
  C  -1.63562777308925      0.62945891119760     -2.34531858643614
  C   1.10729817532124      0.18426412915767     -1.95884330418508
  H   2.55547328168623      1.16201249881651     -4.11196937094032
  H   3.95467005684838      0.14220655560356     -4.57004862590024
  H   2.34759893237052     -0.60992435196578     -4.31922915461677
  H   3.99089832026880     -0.35405436996941     -0.21613788317046
  H   4.31158918649542      0.33351905929062      1.97570271830688
  H   4.13557408900055     -1.44676198701102      1.80829858152573
  H   3.77698356713099     -0.65186821035051      3.37414698882836
  H  -0.36080430792016     -2.58664318610768      0.83590628224539
  H  -2.14208836296676     -4.22311677556409      0.21868172141154
  H  -5.16098957000982     -1.28192187179260      1.10482129403269
  H  -3.35457675165826      0.36328607379854      1.74375459907601
  H  -2.21009272184795      3.02583375971544      0.52020014954210
  H  -0.54542332950208      2.57640561745597      0.03003799374212
  H   0.63597583388991      2.57180117502466      3.01448841387657
  H  -0.92467004611747      3.01103172322707      3.77860950552178
  H  -1.21698335770750      1.56411355989729     -2.74974889588202
  H  -1.42003663661287     -0.20058273405000     -3.03485151297354
  H  -2.71939244835066      0.73389618929992     -2.22898254455403
  H   0.76127525586858      0.34289414489611     -2.97606260609157
```

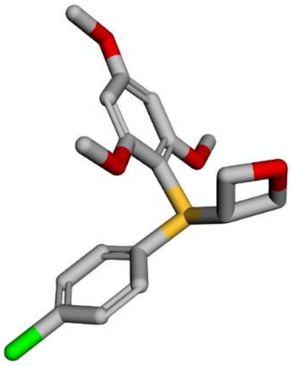

Product_1 = Product_3

```
--------
ENTHALPY
--------

The enthalpy is H = U + kB*T
                kB is Boltzmann's constant
Total free energy                 ...  -1663.15556253 Eh
Thermal Enthalpy correction       ...      0.00094421 Eh       0.59 kcal/mol
-----------------------------------------------------------------------
Total Enthalpy                    ...  -1663.15461832 Eh


Note: Only C1 symmetry has been detected, increase convergence thresholds
      if your molecule has a higher symmetry. Symmetry factor of 1.0 is
      used for the rotational entropy correction.


Note: Rotational entropy computed according to Herzberg
Infrared and Raman Spectra, Chapter V,1, Van Nostrand Reinhold, 1945
Point Group:  C1, Symmetry Number:   1
Rotational constants in cm-1:     0.014649     0.004934     0.004596

Vibrational entropy computed according to the QRRHO of S. Grimme
Chem.Eur.J. 2012 18 9955


-------
ENTROPY
-------

The entropy contributions are T*S = T*(S(el)+S(vib)+S(rot)+S(trans))
     S(el)   - electronic entropy
     S(vib)  - vibrational entropy
     S(rot)  - rotational entropy
     S(trans)- translational entropy
The entropies will be listed as multiplied by the temperature to get
units of energy

Electronic entropy                ...      0.00000000 Eh      0.00 kcal/mol
Vibrational entropy               ...      0.02773635 Eh     17.40 kcal/mol
Rotational entropy                ...      0.01655329 Eh     10.39 kcal/mol
Translational entropy             ...      0.02047724 Eh     12.85 kcal/mol
-----------------------------------------------------------------------
Final entropy term                ...      0.06476688 Eh     40.64 kcal/mol

In case the symmetry of your molecule has not been determined correctly
or in case you have a reason to use a different symmetry number we print
out the resulting rotational entropy values for sn=1,12 :
 --------------------------------------------------------
|  sn= 1 | S(rot)=       0.01655329 Eh     10.39 kcal/mol|
|  sn= 2 | S(rot)=       0.01589883 Eh      9.98 kcal/mol|
|  sn= 3 | S(rot)=       0.01551600 Eh      9.74 kcal/mol|
|  sn= 4 | S(rot)=       0.01524438 Eh      9.57 kcal/mol|
|  sn= 5 | S(rot)=       0.01503369 Eh      9.43 kcal/mol|
|  sn= 6 | S(rot)=       0.01486154 Eh      9.33 kcal/mol|
|  sn= 7 | S(rot)=       0.01471600 Eh      9.23 kcal/mol|
|  sn= 8 | S(rot)=       0.01458992 Eh      9.16 kcal/mol|
|  sn= 9 | S(rot)=       0.01447871 Eh      9.09 kcal/mol|
|  sn=10 | S(rot)=       0.01437923 Eh      9.02 kcal/mol|
|  sn=11 | S(rot)=       0.01428924 Eh      8.97 kcal/mol|
|  sn=12 | S(rot)=       0.01420709 Eh      8.92 kcal/mol|
 --------------------------------------------------------


-------------------
GIBBS FREE ENERGY
-------------------

The Gibbs free energy is G = H - T*S

Total enthalpy                    ...  -1663.15461832 Eh
Total entropy correction          ...     -0.06476688 Eh    -40.64 kcal/mol
-----------------------------------------------------------------------
Final Gibbs free energy         ...  -1663.21938520 Eh

For completeness - the Gibbs free energy minus the electronic energy
G-E(el)                           ...      0.22850481 Eh    143.39 kcal/mol
```

```
35
Coordinates from ORCA-job file
  C   -4.15728386213231      1.69069191566204     -1.69099275657362
  O   -3.73737319971456      1.15326410693904     -0.45555925560136
  C   -2.58586165224700      0.45770468465474     -0.40618235944504
  C   -2.22705069464212     -0.02360483880424      0.85996044487138
  C   -1.05745284340278     -0.76877572476417      1.00218213184012
  O   -0.64119857295580     -1.25788085096792      2.18053580125454
  C   -1.43852865306975     -1.04356824425981      3.32552746845235
  C   -0.23761462461624     -1.03861778886633     -0.11776587315692
  S    1.18947112585239     -2.06868660607660      0.06032138461083
  C    2.42725561617068     -0.91064763810505      0.60903247062293
  C    2.53509253092762      0.37368044330632      0.06361632577305
  C    3.54873126043079      1.23190524149333      0.48221932213954
  C    4.46675311650504      0.79111684710168      1.43534336891674
  Cl   5.74511862036224      1.85919065388922      1.94963889461111
  C    4.37745857158133     -0.48582388850669      1.98387421155362
  C    3.35088460513171     -1.33367300587558      1.56979391542537
  C   -0.60753646080721     -0.52471966913991     -1.37301811079978
  O    0.22723436766483     -0.77210493207037     -2.39725503495372
  C   -0.08164811939841     -0.26490815293794     -3.67797956630205
  C   -1.78463498580824      0.22401462003442     -1.52748723807284
  H   -3.42431710334233      2.41406856300319     -2.08062330282282
  H   -4.31550854379915      0.89783947336480     -2.43866148539658
  H   -5.10655723691544      2.20218313021506     -1.50115368229624
  H   -2.88301167082501      0.20250947479911      1.69745790844897
  H   -0.92514219102292     -1.53970559303641      4.15563965242502
  H   -1.53869248529530      0.03052343100794      3.54483136020595
  H   -2.44003223480582     -1.48284211800299      3.19858361830333
  H    1.82728864838517      0.69894472615509     -0.70146342255684
  H    3.63509387706609      2.23651787590420      0.06680948383257
  H    5.09474378368045     -0.80873152581797      2.73890101968166
  H    3.26557455270401     -2.33259260537462      2.00194366543536
  H    0.73511548625959     -0.57774241103185     -4.33672187605662
  H   -1.03139820043806     -0.67640359506721     -4.05284510113433
  H   -0.13965021903517      0.83428002513978     -3.66692094245354
  H   -2.06522260844831      0.61269397603572     -2.50178244078216
```

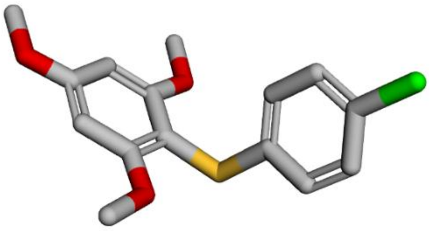

Carbene_1

```
--------
ENTHALPY
--------

The enthalpy is H = U + kB*T
                kB is Boltzmann's constant
Total free energy                 ...   -191.47340432 Eh
Thermal Enthalpy correction       ...      0.00094421 Eh       0.59 kcal/mol
-----------------------------------------------------------------------
Total Enthalpy                    ...   -191.47246011 Eh


Note: Only C1 symmetry has been detected, increase convergence thresholds
      if your molecule has a higher symmetry. Symmetry factor of 1.0 is
      used for the rotational entropy correction.


Note: Rotational entropy computed according to Herzberg
Infrared and Raman Spectra, Chapter V,1, Van Nostrand Reinhold, 1945
Point Group:  C1, Symmetry Number:   1
Rotational constants in cm-1:     0.475223     0.454664     0.254984

Vibrational entropy computed according to the QRRHO of S. Grimme
Chem.Eur.J. 2012 18 9955


-------
ENTROPY
-------

The entropy contributions are T*S = T*(S(el)+S(vib)+S(rot)+S(trans))
     S(el)   - electronic entropy
     S(vib)  - vibrational entropy
     S(rot)  - rotational entropy
     S(trans)- translational entropy
The entropies will be listed as multiplied by the temperature to get
units of energy

Electronic entropy                ...      0.00000000 Eh      0.00 kcal/mol
Vibrational entropy               ...      0.00220373 Eh      1.38 kcal/mol
Rotational entropy                ...      0.01087927 Eh      6.83 kcal/mol
Translational entropy             ...      0.01805168 Eh     11.33 kcal/mol
-----------------------------------------------------------------------
Final entropy term                ...      0.03113468 Eh     19.54 kcal/mol

In case the symmetry of your molecule has not been determined correctly
or in case you have a reason to use a different symmetry number we print
out the resulting rotational entropy values for sn=1,12 :
 --------------------------------------------------------
|  sn= 1 | S(rot)=       0.01087927 Eh      6.83 kcal/mol|
|  sn= 2 | S(rot)=       0.01022481 Eh      6.42 kcal/mol|
|  sn= 3 | S(rot)=       0.00984198 Eh      6.18 kcal/mol|
|  sn= 4 | S(rot)=       0.00957036 Eh      6.01 kcal/mol|
|  sn= 5 | S(rot)=       0.00935967 Eh      5.87 kcal/mol|
|  sn= 6 | S(rot)=       0.00918753 Eh      5.77 kcal/mol|
|  sn= 7 | S(rot)=       0.00904198 Eh      5.67 kcal/mol|
|  sn= 8 | S(rot)=       0.00891590 Eh      5.59 kcal/mol|
|  sn= 9 | S(rot)=       0.00880469 Eh      5.53 kcal/mol|
|  sn=10 | S(rot)=       0.00870521 Eh      5.46 kcal/mol|
|  sn=11 | S(rot)=       0.00861522 Eh      5.41 kcal/mol|
|  sn=12 | S(rot)=       0.00853307 Eh      5.35 kcal/mol|
 --------------------------------------------------------


-------------------
GIBBS FREE ENERGY
-------------------

The Gibbs free energy is G = H - T*S

Total enthalpy                    ...   -191.47246011 Eh
Total entropy correction          ...     -0.03113468 Eh    -19.54 kcal/mol
-----------------------------------------------------------------------
Final Gibbs free energy         ...   -191.50359479 Eh

For completeness - the Gibbs free energy minus the electronic energy
G-E(el)                           ...      0.03342877 Eh     20.98 kcal/mol
```

```
8
Coordinates from ORCA-job file
  C   0.00797364679635      0.06037777026170      1.10810231763502
  C   -0.98020127323693      0.02009077566154     -0.00510231646636
  O   -0.00754698283806     -0.06056371251686     -1.05055330645627
  C   0.97998315808614     -0.02263823104944     -0.01692331553945
  H   -1.61352914196896      0.92768756005221     -0.04782756419533
  H   -1.65469862438453     -0.85616033589958      0.04969977306681
  H   1.65028057344393      0.85758761086338     -0.06678151030009
  H   1.61773864410206     -0.92648143737295      0.02928592225567
```

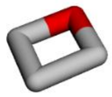

Reactant_2

```
--------
ENTHALPY
--------

The enthalpy is H = U + kB*T
                kB is Boltzmann's constant
Total free energy                 ...  -1359.32872163 Eh
Thermal Enthalpy correction       ...      0.00094421 Eh       0.59 kcal/mol
-----------------------------------------------------------------------
Total Enthalpy                    ...  -1359.32777742 Eh


Note: Only C1 symmetry has been detected, increase convergence thresholds
      if your molecule has a higher symmetry. Symmetry factor of 1.0 is
      used for the rotational entropy correction.


Note: Rotational entropy computed according to Herzberg
Infrared and Raman Spectra, Chapter V,1, Van Nostrand Reinhold, 1945
Point Group:  C1, Symmetry Number:   1
Rotational constants in cm-1:     0.009936     0.006242     0.005716

Vibrational entropy computed according to the QRRHO of S. Grimme
Chem.Eur.J. 2012 18 9955


-------
ENTROPY
-------

The entropy contributions are T*S = T*(S(el)+S(vib)+S(rot)+S(trans))
     S(el)   - electronic entropy
     S(vib)  - vibrational entropy
     S(rot)  - rotational entropy
     S(trans)- translational entropy
The entropies will be listed as multiplied by the temperature to get
units of energy

Electronic entropy                ...      0.00000000 Eh      0.00 kcal/mol
Vibrational entropy               ...      0.03533083 Eh     22.17 kcal/mol
Rotational entropy                ...      0.01652254 Eh     10.37 kcal/mol
Translational entropy             ...      0.02056406 Eh     12.90 kcal/mol
-----------------------------------------------------------------------
Final entropy term                ...      0.07241743 Eh     45.44 kcal/mol

In case the symmetry of your molecule has not been determined correctly
or in case you have a reason to use a different symmetry number we print
out the resulting rotational entropy values for sn=1,12 :
 --------------------------------------------------------
|  sn= 1 | S(rot)=       0.01652254 Eh     10.37 kcal/mol|
|  sn= 2 | S(rot)=       0.01586808 Eh      9.96 kcal/mol|
|  sn= 3 | S(rot)=       0.01548525 Eh      9.72 kcal/mol|
|  sn= 4 | S(rot)=       0.01521363 Eh      9.55 kcal/mol|
|  sn= 5 | S(rot)=       0.01500294 Eh      9.41 kcal/mol|
|  sn= 6 | S(rot)=       0.01483080 Eh      9.31 kcal/mol|
|  sn= 7 | S(rot)=       0.01468525 Eh      9.22 kcal/mol|
|  sn= 8 | S(rot)=       0.01455917 Eh      9.14 kcal/mol|
|  sn= 9 | S(rot)=       0.01444796 Eh      9.07 kcal/mol|
|  sn=10 | S(rot)=       0.01434848 Eh      9.00 kcal/mol|
|  sn=11 | S(rot)=       0.01425849 Eh      8.95 kcal/mol|
|  sn=12 | S(rot)=       0.01417634 Eh      8.90 kcal/mol|
 --------------------------------------------------------


-------------------
GIBBS FREE ENERGY
-------------------

The Gibbs free energy is G = H - T*S

Total enthalpy                    ...  -1359.32777742 Eh
Total entropy correction          ...     -0.07241743 Eh    -45.44 kcal/mol
-----------------------------------------------------------------------
Final Gibbs free energy         ...  -1359.40019484 Eh

For completeness - the Gibbs free energy minus the electronic energy
G-E(el)                           ...      0.32345182 Eh    202.97 kcal/mol
```

45
Coordinates from ORCA-job file

C	5.10298180240276	0.23968014347146	-0.36267014378335
O	4.18241734587517	0.18545188389013	-1.43152189656109
C	2.86880218770285	0.08711417028504	-1.15873375454431
C	2.36186118472846	0.01199534019389	0.14309420583845
C	0.97388879628404	-0.07942412774101	0.31978341454182
O	0.39611641978115	-0.16938415371834	1.52799501482910
C	1.17903791163158	0.01534606536161	2.68646666504529
C	0.10763097468644	-0.09761377843098	-0.78424616759631
S	-1.68317906073259	0.03888774077914	-0.57545944616248
C	-1.97374122675162	-1.39185619082212	0.48834282127500
C	-1.46486458101095	-2.62988057556195	0.09608878090916
C	-1.74654854182988	-3.75643714674001	0.86744933548770
C	-2.53068383872971	-3.64069672849549	2.01756113833518
C	-3.03627937018248	-2.39548943321106	2.39290012793795
C	-2.76545901981508	-1.26229353538965	1.62367476254680
C	-2.17762436191051	1.43925990037027	0.22536956829709
C	-1.36152465698697	2.23925145748359	1.23173406403739
C	-2.10449052422330	3.48594301714784	0.69847430056487
C	-2.46480117272193	2.70448368306423	-0.58875260466459
C	0.64222280996576	-0.02223930813617	-2.08581526916614
O	-0.24434623953006	-0.03871928644729	-3.09683076968966
C	0.23606863700738	0.04290065723723	-4.42285532106000
C	2.02087141444043	0.06353139900985	-2.27583418659099
H	6.09529965112399	0.34780364271267	-0.81293686857657
H	4.90191208409120	1.10374002785361	0.28922858677828
H	5.07284373252396	-0.68255484804949	0.23832323422533
H	3.02465019160377	0.02181168095323	1.00360348003985
H	0.48781386148613	-0.02191469749643	3.53528136475187
H	1.68430324927094	0.99385240684279	2.66618901519806
H	1.93024383285443	-0.78196754191240	2.79472054409836
H	-0.84966015153237	-2.71009067599869	-0.80350389857701
H	-1.35582243606379	-4.72914537669853	0.56386664927134
H	-2.75278442956479	-4.52356491394781	2.61911357022804
H	-3.64920978093711	-2.30455034580141	3.29152470953896
H	-3.15300087166772	-0.27636122686033	1.88713726340405
H	-0.28094867294271	2.29167881981823	0.98463500091859
H	-1.45251895997187	2.00555116932407	2.30599254628713
H	-1.51712464011033	4.40700754160217	0.57849485818267
H	-3.00068427559464	3.70404653075718	1.29517340069397
H	-3.47680113031193	2.85195659912438	-1.00014000978050
H	-1.73039611863277	2.91764377806304	-1.38990048438691
H	0.88123028341927	-0.81562698305902	-4.66435010964052
H	0.79646894540047	0.97644472233617	-4.58387649415542
H	-0.64624828631963	0.03109222436953	-5.07080932514924
H	2.47217703179455	0.12333627246681	-3.26388167317723

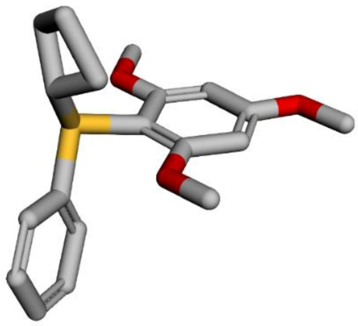

Product_2

```
--------
ENTHALPY
--------

The enthalpy is H = U + kB*T
                kB is Boltzmann's constant
Total free energy                 ...  -1203.70026197 Eh
Thermal Enthalpy correction       ...      0.00094421 Eh       0.59 kcal/mol
-----------------------------------------------------------------------
Total Enthalpy                    ...  -1203.69931776 Eh


Note: Only C1 symmetry has been detected, increase convergence thresholds
      if your molecule has a higher symmetry. Symmetry factor of 1.0 is
      used for the rotational entropy correction.


Note: Rotational entropy computed according to Herzberg
Infrared and Raman Spectra, Chapter V,1, Van Nostrand Reinhold, 1945
Point Group:  C1, Symmetry Number:   1
Rotational constants in cm-1:     0.017331     0.007525     0.006454

Vibrational entropy computed according to the QRRHO of S. Grimme
Chem.Eur.J. 2012 18 9955


-------
ENTROPY
-------

The entropy contributions are T*S = T*(S(el)+S(vib)+S(rot)+S(trans))
     S(el)   - electronic entropy
     S(vib)  - vibrational entropy
     S(rot)  - rotational entropy
     S(trans)- translational entropy
The entropies will be listed as multiplied by the temperature to get
units of energy

Electronic entropy                ...      0.00000000 Eh      0.00 kcal/mol
Vibrational entropy               ...      0.02438138 Eh     15.30 kcal/mol
Rotational entropy                ...      0.01611430 Eh     10.11 kcal/mol
Translational entropy             ...      0.02031088 Eh     12.75 kcal/mol
-----------------------------------------------------------------------
Final entropy term                ...      0.06080656 Eh     38.16 kcal/mol

In case the symmetry of your molecule has not been determined correctly
or in case you have a reason to use a different symmetry number we print
out the resulting rotational entropy values for sn=1,12 :
 --------------------------------------------------------
|  sn= 1 | S(rot)=       0.01611430 Eh     10.11 kcal/mol|
|  sn= 2 | S(rot)=       0.01545985 Eh      9.70 kcal/mol|
|  sn= 3 | S(rot)=       0.01507701 Eh      9.46 kcal/mol|
|  sn= 4 | S(rot)=       0.01480539 Eh      9.29 kcal/mol|
|  sn= 5 | S(rot)=       0.01459470 Eh      9.16 kcal/mol|
|  sn= 6 | S(rot)=       0.01442256 Eh      9.05 kcal/mol|
|  sn= 7 | S(rot)=       0.01427701 Eh      8.96 kcal/mol|
|  sn= 8 | S(rot)=       0.01415093 Eh      8.88 kcal/mol|
|  sn= 9 | S(rot)=       0.01403973 Eh      8.81 kcal/mol|
|  sn=10 | S(rot)=       0.01394025 Eh      8.75 kcal/mol|
|  sn=11 | S(rot)=       0.01385026 Eh      8.69 kcal/mol|
|  sn=12 | S(rot)=       0.01376810 Eh      8.64 kcal/mol|
 --------------------------------------------------------


-------------------
GIBBS FREE ENERGY
-------------------

The Gibbs free energy is G = H - T*S

Total enthalpy                    ...  -1203.69931776 Eh
Total entropy correction          ...     -0.06080656 Eh    -38.16 kcal/mol
-----------------------------------------------------------------------
Final Gibbs free energy         ...  -1203.76012432 Eh

For completeness - the Gibbs free energy minus the electronic energy
G-E(el)                           ...      0.24082498 Eh    151.12 kcal/mol
```

35
Coordinates from ORCA-job file

C	-4.02724172602746	-2.05514899268927	1.53328366594920
O	-3.71829287657244	-1.20715601942160	0.44780107021456
C	-2.55996912124402	-0.52203920056256	0.46649887012719
C	-1.64388191759093	-0.59413818393020	1.52020438932200
C	-0.46774364036008	0.16811515781357	1.44559125495915
O	0.46899946251669	0.14508448915419	2.40642034648796
C	0.26286350612291	-0.64909102800117	3.55502904157993
C	-0.21043235883439	0.99491519687894	0.33616435370895
S	1.23926131425598	1.99909488345725	0.28712668333736
C	2.41776868955106	0.97050957081918	-0.56085090781549
C	2.13269613824109	-0.31364686854857	-1.03513174131865
C	3.12399854016638	-1.04756844540716	-1.68968942883326
C	4.39912722422622	-0.51478647863381	-1.87675270770835
C	4.68030069854935	0.76877612495676	-1.40295515471554
C	3.69899753517519	1.50964056462900	-0.74895814324325
C	-1.14766558871844	1.03556790147024	-0.72074369429221
O	-0.83843405394209	1.81357892403114	-1.77008564683462
C	-1.75388036896973	1.90312434734816	-2.84206315476004
C	-2.31912411675717	0.28149319060031	-0.65568661364707
H	-3.27204141419249	-2.84851713814371	1.64633820196963
H	-4.99925162067543	-2.50702000999449	1.31004993316759
H	-4.09714177012884	-1.48612737098337	2.47343186888809
H	-1.83706922576913	-1.22590082619375	2.38193477610765
H	1.13340945089538	-0.48697217117109	4.19893287262702
H	-0.64835148189708	-0.34590474535675	4.09324849832236
H	0.19474834236824	-1.71656017364589	3.29453383095452
H	1.13828476805395	-0.74110876062694	-0.89393818049151
H	2.89113353229340	-2.04965609381050	-2.05518316220827
H	5.17010781468856	-1.09242121677241	-2.38875339003455
H	5.67372209183362	1.19952837017706	-1.54235919792540
H	3.92367685455098	2.51360793370394	-0.38093207153254
H	-2.72588519975554	2.29036190546169	-2.49970379410141
H	-1.90106386764942	0.92242600481904	-3.32004286953534
H	-1.31763997394108	2.60024194681227	-3.56483783868023
H	-3.06408564046322	0.28779721176047	-1.44802196004544

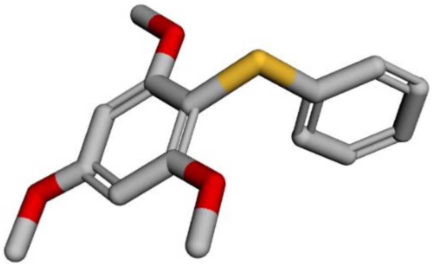

Carbene_2

```
--------
ENTHALPY
--------

The enthalpy is H = U + kB*T
                kB is Boltzmann's constant
Total free energy                 ...   -155.58589180 Eh
Thermal Enthalpy correction       ...      0.00094421 Eh       0.59 kcal/mol
-----------------------------------------------------------------------
Total Enthalpy                    ...   -155.58494759 Eh


Note: Only C1 symmetry has been detected, increase convergence thresholds
      if your molecule has a higher symmetry. Symmetry factor of 1.0 is
      used for the rotational entropy correction.


Note: Rotational entropy computed according to Herzberg
Infrared and Raman Spectra, Chapter V,1, Van Nostrand Reinhold, 1945
Point Group:  C1, Symmetry Number:   1
Rotational constants in cm-1:     0.417595     0.394261     0.228763

Vibrational entropy computed according to the QRRHO of S. Grimme
Chem.Eur.J. 2012 18 9955


-------
ENTROPY
-------

The entropy contributions are T*S = T*(S(el)+S(vib)+S(rot)+S(trans))
     S(el)   - electronic entropy
     S(vib)  - vibrational entropy
     S(rot)  - rotational entropy
     S(trans)- translational entropy
The entropies will be listed as multiplied by the temperature to get
units of energy

Electronic entropy                ...      0.00000000 Eh      0.00 kcal/mol
Vibrational entropy               ...      0.00073254 Eh      0.46 kcal/mol
Rotational entropy                ...      0.01105882 Eh      6.94 kcal/mol
Translational entropy             ...      0.01800097 Eh     11.30 kcal/mol
-----------------------------------------------------------------------
Final entropy term                ...      0.02979233 Eh     18.69 kcal/mol

In case the symmetry of your molecule has not been determined correctly
or in case you have a reason to use a different symmetry number we print
out the resulting rotational entropy values for sn=1,12 :
 --------------------------------------------------------
|  sn= 1 | S(rot)=       0.01105882 Eh      6.94 kcal/mol|
|  sn= 2 | S(rot)=       0.01040437 Eh      6.53 kcal/mol|
|  sn= 3 | S(rot)=       0.01002153 Eh      6.29 kcal/mol|
|  sn= 4 | S(rot)=       0.00974991 Eh      6.12 kcal/mol|
|  sn= 5 | S(rot)=       0.00953922 Eh      5.99 kcal/mol|
|  sn= 6 | S(rot)=       0.00936708 Eh      5.88 kcal/mol|
|  sn= 7 | S(rot)=       0.00922153 Eh      5.79 kcal/mol|
|  sn= 8 | S(rot)=       0.00909545 Eh      5.71 kcal/mol|
|  sn= 9 | S(rot)=       0.00898424 Eh      5.64 kcal/mol|
|  sn=10 | S(rot)=       0.00888476 Eh      5.58 kcal/mol|
|  sn=11 | S(rot)=       0.00879477 Eh      5.52 kcal/mol|
|  sn=12 | S(rot)=       0.00871262 Eh      5.47 kcal/mol|
 --------------------------------------------------------


-------------------
GIBBS FREE ENERGY
-------------------

The Gibbs free energy is G = H - T*S

Total enthalpy                    ...   -155.58494759 Eh
Total entropy correction          ...     -0.02979233 Eh    -18.69 kcal/mol
-----------------------------------------------------------------------
Final Gibbs free energy         ...   -155.61473992 Eh

For completeness - the Gibbs free energy minus the electronic energy
G-E(el)                           ...      0.05638603 Eh     35.38 kcal/mol
```

```
10
Coordinates from ORCA-job file
  C   -0.00823933894506     -1.25526235924789     -0.64155891650021
  C   1.03989384950752     -0.25716060713420     -0.22525520218404
  C   0.00459459976979      0.72019415253518      0.36687075809519
  C   -1.04349592225059     -0.32217400203659     -0.07136291662022
  H   1.63252108803263      0.08495729988659     -1.09355407504744
  H   1.77411833609645     -0.70882553735091      0.46665930647750
  H   0.07990647423449      0.88321493328528      1.45015834702053
  H   -0.06401398278630      1.69449323583109     -0.13488190216277
  H   -1.78129797399263     -0.02048540332345     -0.83719486510423
  H   -1.63408712966632     -0.81905171244511      0.72021946602569
```

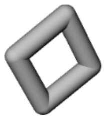

Reactant_3

```
--------
ENTHALPY
--------

The enthalpy is H = U + kB*T
                kB is Boltzmann's constant
Total free energy                 ...  -2653.01550853 Eh
Thermal Enthalpy correction       ...      0.00094421 Eh       0.59 kcal/mol
-----------------------------------------------------------------------
Total Enthalpy                    ...  -2653.01456432 Eh


Note: Only C1 symmetry has been detected, increase convergence thresholds
      if your molecule has a higher symmetry. Symmetry factor of 1.0 is
      used for the rotational entropy correction.


Note: Rotational entropy computed according to Herzberg
Infrared and Raman Spectra, Chapter V,1, Van Nostrand Reinhold, 1945
Point Group:  C1, Symmetry Number:   1
Rotational constants in cm-1:     0.004011     0.002075     0.001747

Vibrational entropy computed according to the QRRHO of S. Grimme
Chem.Eur.J. 2012 18 9955


-------
ENTROPY
-------

The entropy contributions are T*S = T*(S(el)+S(vib)+S(rot)+S(trans))
     S(el)   - electronic entropy
     S(vib)  - vibrational entropy
     S(rot)  - rotational entropy
     S(trans)- translational entropy
The entropies will be listed as multiplied by the temperature to get
units of energy

Electronic entropy                ...      0.00000000 Eh      0.00 kcal/mol
Vibrational entropy               ...      0.05316595 Eh     33.36 kcal/mol
Rotational entropy                ...      0.01803049 Eh     11.31 kcal/mol
Translational entropy             ...      0.02120635 Eh     13.31 kcal/mol
-----------------------------------------------------------------------
Final entropy term                ...      0.09240278 Eh     57.98 kcal/mol

In case the symmetry of your molecule has not been determined correctly
or in case you have a reason to use a different symmetry number we print
out the resulting rotational entropy values for sn=1,12 :
 --------------------------------------------------------
|  sn= 1 | S(rot)=       0.01803049 Eh     11.31 kcal/mol|
|  sn= 2 | S(rot)=       0.01737603 Eh     10.90 kcal/mol|
|  sn= 3 | S(rot)=       0.01699320 Eh     10.66 kcal/mol|
|  sn= 4 | S(rot)=       0.01672157 Eh     10.49 kcal/mol|
|  sn= 5 | S(rot)=       0.01651088 Eh     10.36 kcal/mol|
|  sn= 6 | S(rot)=       0.01633874 Eh     10.25 kcal/mol|
|  sn= 7 | S(rot)=       0.01619319 Eh     10.16 kcal/mol|
|  sn= 8 | S(rot)=       0.01606712 Eh     10.08 kcal/mol|
|  sn= 9 | S(rot)=       0.01595591 Eh     10.01 kcal/mol|
|  sn=10 | S(rot)=       0.01585643 Eh      9.95 kcal/mol|
|  sn=11 | S(rot)=       0.01576644 Eh      9.89 kcal/mol|
|  sn=12 | S(rot)=       0.01568428 Eh      9.84 kcal/mol|
 --------------------------------------------------------


-------------------
GIBBS FREE ENERGY
-------------------

The Gibbs free energy is G = H - T*S

Total enthalpy                    ...  -2653.01456432 Eh
Total entropy correction          ...     -0.09240278 Eh    -57.98 kcal/mol
-----------------------------------------------------------------------
Final Gibbs free energy         ...  -2653.10696710 Eh

For completeness - the Gibbs free energy minus the electronic energy
G-E(el)                           ...      0.41233296 Eh    258.74 kcal/mol
```

60
Coordinates from ORCA-job file

C	-3.88161923948404	-0.17481829373088	3.79293441468980
O	-3.26277694191335	-1.38670206052819	3.41314618607412
C	-2.66927932326888	-1.46450485359491	2.20986927756936
C	-2.64619715229885	-0.40254641281150	1.29793028577474
C	-1.99197743055783	-0.58485985800177	0.07231297452602
O	-1.91140950475158	0.37042354411579	-0.86728670923657
C	-2.47038080159470	1.64391487229131	-0.61897406472686
C	-1.38730834206401	-1.81288866653048	-0.24125432511722
S	-0.41386991670752	-2.01297876620030	-1.73778712033197
C	-1.64505394618903	-1.50711454697649	-2.97160139319739
C	-2.94611594354643	-2.00038683651012	-2.88633354010160
C	-3.87440394153684	-1.65962511047633	-3.86695074285528
C	-3.47805974678526	-0.83642012692877	-4.92180432144547
Cl	-4.63861955297089	-0.39921473525694	-6.14396136816903
C	-2.17341211156902	-0.35690231960574	-5.01663152641241
C	-1.24833569212319	-0.70130621953013	-4.03146574074289
C	0.90111588236840	-0.98517928883396	-1.82052543823975
C	1.10858666926036	0.28662469270387	-1.01094442106132
N	2.46715703652213	-0.21786235584099	-0.64969152743463
S	3.79150565665687	0.70238304173899	-1.03406207176642
O	4.93909364466437	-0.19382327470894	-0.93158278069193
O	3.59641591551152	1.43729887294723	-2.28364446469466
C	3.86019353293644	1.89201425463410	0.27514347618064
C	3.66143616519662	3.23793604458006	-0.01565293439668
C	3.79768693610837	4.17339313784707	1.00946150706297
C	4.13603795321047	3.77591172678677	2.30788548244577
C	4.33119610607141	4.79214696786923	3.40024136160116
C	4.32195440639400	2.40819584453081	2.56946131451920
C	4.19240187037882	1.46131484212712	1.56088648629050
C	2.29721576580683	-1.47423016335490	-1.44609145523608
C	-1.42436397982314	-2.87044059087696	0.69017504265610
O	-0.82868919283341	-4.01680158147960	0.32253988912165
C	-0.80838148101397	-5.09608132215007	1.23576957469767
C	-2.06718327513863	-2.69631083799403	1.91439670208529
H	-4.70042313959831	0.08506729373956	3.10470169322194
H	-3.15397867839646	0.65092029117713	3.82167539921335
H	-4.28966623044554	-0.33431483037994	4.79641995229921
H	-3.11683077410873	0.54775422555682	1.53223470378810
H	-2.00473284557228	2.11352600388534	0.26092312465480
H	-2.25993096935798	2.24679874888467	-1.50845052149797
H	-3.55819441361444	1.57531731730000	-0.46912709394812
H	-3.24566277745031	-2.63723630883842	-2.05106070139904
H	-4.89998688520551	-2.02611310498135	-3.81605498029757
H	-1.88698988551424	0.28076275794231	-5.85353223046990
H	-0.21697458507204	-0.34381321302284	-4.06828734261149
H	0.48691686536841	0.41799690693611	-0.10882243458586
H	1.13348032099010	1.23797568271330	-1.57091441425387
H	3.41305484936680	3.54144220091128	-1.03326929654723
H	3.64543894585564	5.23290862738992	0.79349097379075
H	3.94544915010127	4.42053667909316	4.35914484218867
H	5.40385482573191	5.00121912111994	3.53543989552278
H	3.83182887319101	5.73871457794581	3.15698231187424
H	4.58251582375122	2.08669496378696	3.58010575378084
H	4.35188832341527	0.39997383235231	1.75976665910229
H	3.02394959448321	-1.55618047438340	-2.27308589153871
H	2.38868499396034	-2.37215778096945	-0.81344864707731
H	-0.27094540714413	-4.82239938155397	2.15633028077766
H	-0.28218992394822	-5.91394535152237	0.73323346876592
H	-1.82978107546871	-5.41727399222787	1.48975286989850
H	-2.12533500023434	-3.48473441310540	2.66144359591121

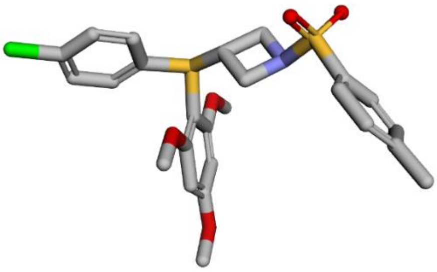

Carbene_3

```
--------
ENTHALPY
--------

The enthalpy is H = U + kB*T
                kB is Boltzmann's constant
Total free energy                 ...   -989.81359129 Eh
Thermal Enthalpy correction       ...      0.00094421 Eh       0.59 kcal/mol
-----------------------------------------------------------------------
Total Enthalpy                    ...   -989.81264708 Eh


Note: Only C1 symmetry has been detected, increase convergence thresholds
      if your molecule has a higher symmetry. Symmetry factor of 1.0 is
      used for the rotational entropy correction.


Note: Rotational entropy computed according to Herzberg
Infrared and Raman Spectra, Chapter V,1, Van Nostrand Reinhold, 1945
Point Group:  C1, Symmetry Number:   1
Rotational constants in cm-1:     0.036922     0.014454     0.013298

Vibrational entropy computed according to the QRRHO of S. Grimme
Chem.Eur.J. 2012 18 9955


-------
ENTROPY
-------

The entropy contributions are T*S = T*(S(el)+S(vib)+S(rot)+S(trans))
     S(el)   - electronic entropy
     S(vib)  - vibrational entropy
     S(rot)  - rotational entropy
     S(trans)- translational entropy
The entropies will be listed as multiplied by the temperature to get
units of energy

Electronic entropy                ...      0.00000000 Eh      0.00 kcal/mol
Vibrational entropy               ...      0.01806483 Eh     11.34 kcal/mol
Rotational entropy                ...      0.01510784 Eh      9.48 kcal/mol
Translational entropy             ...      0.01991705 Eh     12.50 kcal/mol
-----------------------------------------------------------------------
Final entropy term                ...      0.05308972 Eh     33.31 kcal/mol

In case the symmetry of your molecule has not been determined correctly
or in case you have a reason to use a different symmetry number we print
out the resulting rotational entropy values for sn=1,12 :
 --------------------------------------------------------
|  sn= 1 | S(rot)=       0.01510784 Eh      9.48 kcal/mol|
|  sn= 2 | S(rot)=       0.01445339 Eh      9.07 kcal/mol|
|  sn= 3 | S(rot)=       0.01407056 Eh      8.83 kcal/mol|
|  sn= 4 | S(rot)=       0.01379893 Eh      8.66 kcal/mol|
|  sn= 5 | S(rot)=       0.01358824 Eh      8.53 kcal/mol|
|  sn= 6 | S(rot)=       0.01341610 Eh      8.42 kcal/mol|
|  sn= 7 | S(rot)=       0.01327055 Eh      8.33 kcal/mol|
|  sn= 8 | S(rot)=       0.01314448 Eh      8.25 kcal/mol|
|  sn= 9 | S(rot)=       0.01303327 Eh      8.18 kcal/mol|
|  sn=10 | S(rot)=       0.01293379 Eh      8.12 kcal/mol|
|  sn=11 | S(rot)=       0.01284380 Eh      8.06 kcal/mol|
|  sn=12 | S(rot)=       0.01276164 Eh      8.01 kcal/mol|
 --------------------------------------------------------


-------------------
GIBBS FREE ENERGY
-------------------
The Gibbs free energy is G = H - T*S

Total enthalpy                    ...   -989.81264708 Eh
Total entropy correction          ...     -0.05308972 Eh    -33.31 kcal/mol
-----------------------------------------------------------------------
Final Gibbs free energy         ...   -989.86573680 Eh

For completeness - the Gibbs free energy minus the electronic energy
G-E(el)                           ...      0.15305747 Eh     96.04 kcal/mol
```

```
25
Coordinates from ORCA-job file
  C   3.41196604220277      1.03532087394917      1.55413854990317
  C   2.21388301287426      0.36360257610511      0.94104510156519
  C   2.02402477274826      0.36730554591040     -0.44910217312045
  C   0.93285187551521     -0.27453151464625     -1.02759506358490
  C   0.01249112960281     -0.91979979804412     -0.19731789257935
  S  -1.42270244227077     -1.68503545387674     -0.90563312429048
  O  -1.96626975442251     -2.62141188043065      0.06652450417007
  O  -1.09767310022794     -2.10244251763091     -2.25995217324181
  N  -2.55827647662528     -0.48090758424162     -1.06092565456325
  C  -3.04089611499425      0.33388232224558      0.06776487781899
  C  -3.08892420858891      1.57147565283549     -0.77523476656030
  C  -2.33119571427362      0.77715408989364     -1.79356988986044
  C   0.17870783482881     -0.94511794019769      1.18746793245430
  C   1.28061116821764     -0.30285774066975      1.74583066391086
  H   3.23049818181677      1.27962850284374      2.60865602918404
  H   3.66992926019486      1.95505067292367      1.01239336113270
  H   4.28764128644582      0.36913734437310      1.50916414823340
  H   2.74723521845292      0.87803055391685     -1.08855379774668
  H   0.80048031830492     -0.28864026564655     -2.11120384421451
  H  -3.96061730829540     -0.00361063475613      0.56958038934895
  H  -2.27158514254651      0.52675107713838      0.84991160839970
  H  -1.28386323224566      1.15249375567554     -1.73656539623356
  H  -2.64709624801272      0.81074865898898     -2.84739257643950
  H  -0.54143679738909     -1.47508918445294      1.81265359246185
  H   1.42021643868763     -0.32113711220630      2.82851559385202
```

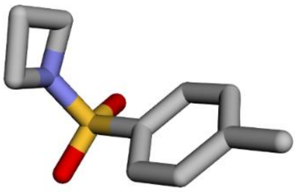

12.3. Bioisostere Identification through Clustering Approach

Representative cycle-types

Each structure was labelled based on its representative cycle-type (i.e., spiro, bridged, bridged&fused, fused, one-cycle and relative aromatic versions) as shown in Figure 2.

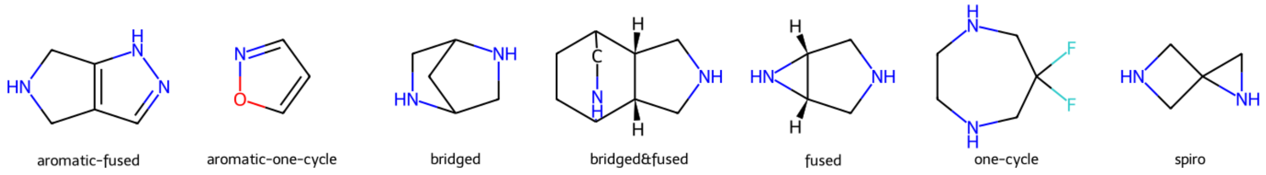

Figure 2. Representative structure for each cycle-type.

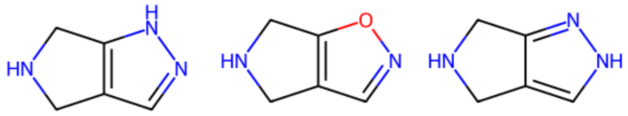

Figure 3. Piperazine bioisosteres labeled as aromatic-fused cores.

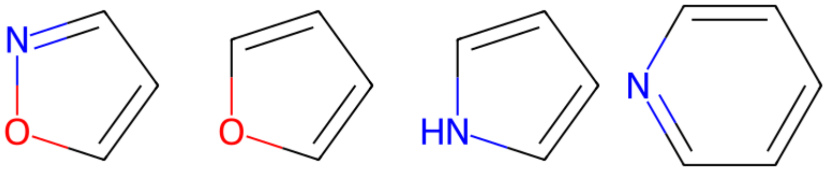

Figure 4. Piperazine bioisosteres labeled as aromatic-one-cycle cores.

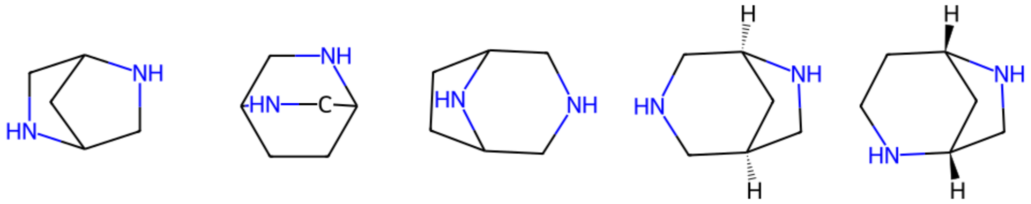

Figure 5. Piperazine bioisosteres labeled as bridged cores.

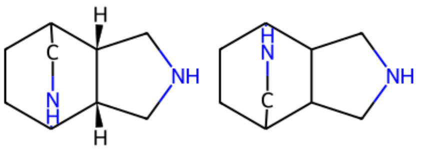

Figure 6. Piperazine bioisosteres labeled as bridged&fused cores.

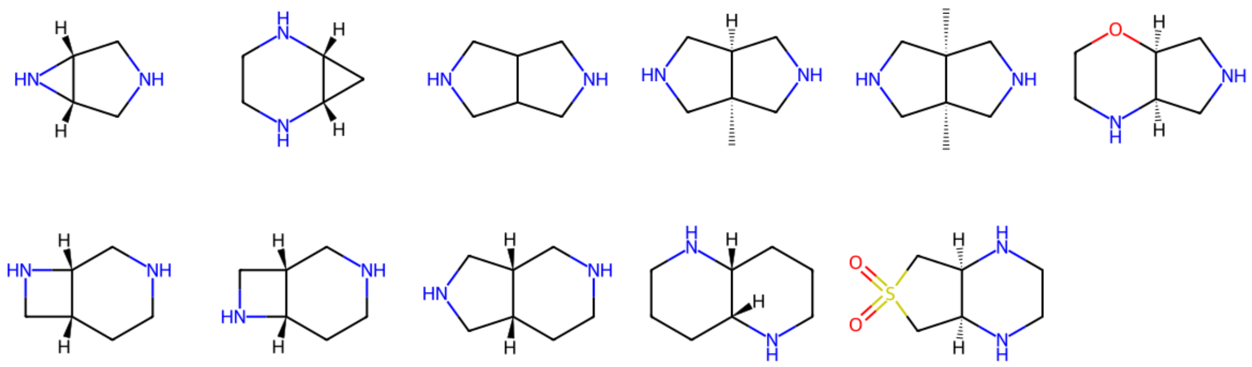

Figure 7. Piperazine bioisosteres labeled as fused cores.

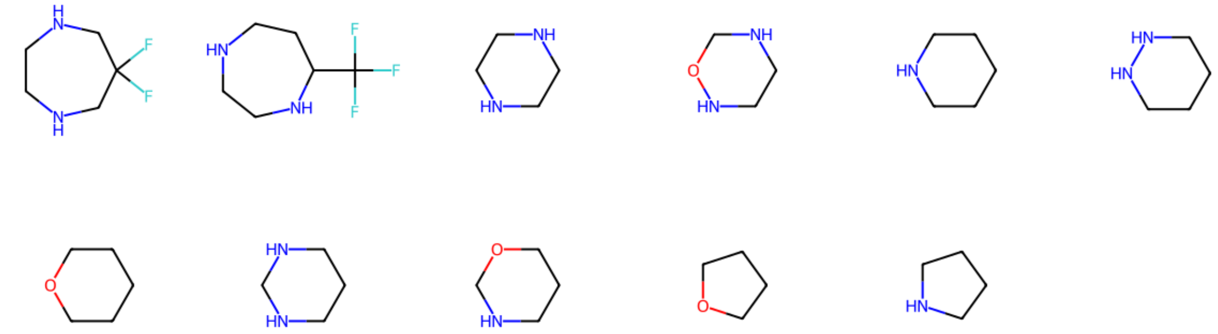

Figure 8. Piperazine bioisosteres labeled as one-cycle cores.

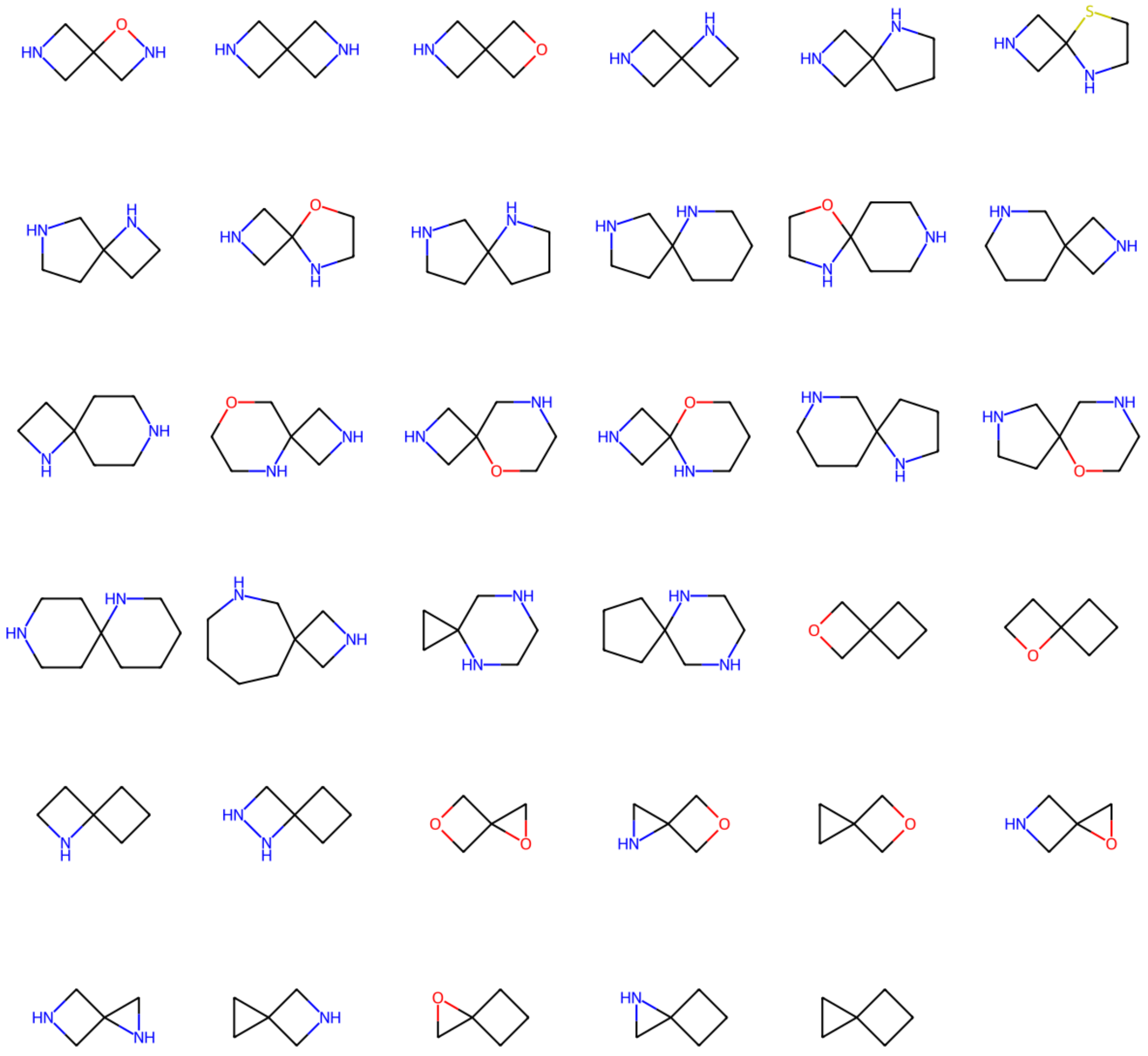

Figure 9. Piperazine bioisosteres labeled as spiro cores.

QM and Programming Language

3D optimization

Ab-initio calculations using Density Functional Theory (DFT) were carried out to determine the atomic positions. All quantum-chemical calculations were performed with ORCA (version 5.0.4). Geometry optimizations were carried out using the WB97X-D3BJ hybrid density functional, which includes the D3(BJ) dispersion correction. The 6-31++G(d,p) basis set was employed for all atoms.

Geometry optimizations were performed without symmetry constraints. The Hessian matrix was explicitly computed at each optimization step to ensure a reliable characterization of stationary points and to improve optimization robustness. A maximum number of 500 geometry optimization steps was allowed.

Convergence criteria were kept at ORCA defaults unless otherwise stated. All optimized structures were confirmed as true minima by verifying the absence of imaginary vibrational frequencies.

Structural optimization was conducted using Density Functional Theory (DFT) adopting WB97X-D3BJ as density functional with the 6-31++G(d,p) as basis set.

Strain Energy

The ring strain energy (RSE) can be evaluated using Bader's Quantum Theory of Atoms in Molecules (QTAIM). As reported in previous studies, the kinetic energy density at the ring critical point (3,+1) provides a quantitative descriptor for estimating ring strain. To obtain the RSE values, the regression relationship

$$RSE = 337.72 \times G(r) - 8.115$$

was applied, following the procedure outlined in Amsterdam Density Functional (Theoretical Chemistry, Vrije Universiteit, Amsterdam, The Netherlands, http://www.scm.com).

To obtain the values of G(r) required for the ring strain energy evaluation, the electronic wavefunctions generated with ORCA were first processed using the orca_2aim utility to produce the files suitable for QTAIM analysis. Critical point identification was then carried out with Multiwfn (version 3.7), from which the Lagrangian kinetic energy density at each ring critical point was computed.

In cases where more than one ring critical point (3,+1) was detected, the corresponding kinetic energy densities were averaged to yield a single representative G(r) value for the ring.

Python library

Molecular descriptors were computed using Python (version 3.13) and the RDKit cheminformatics library (version 2024.9). For each structure, a set of 2D and 3D descriptors—including topological indices, physicochemical properties, and geometry-based features—was generated using the standard RDKit descriptor calculators.

All data handling, preprocessing, and organization of the descriptor matrices were performed with pandas (version 2.2.3), employing the library's built-in functionalities for tabular data manipulation and NumPy (version 2.3.2). Statistical analyses and dimensionality-reduction procedures (e.g., PCA) were conducted within the same Python environment employing SciKit Learn (version 1.6.1).

Plots, visualizations, and graphical summaries of the descriptor space were produced using matplotlib (version 3.10.3), following standard non-interactive plotting routines. All scripts used for experiments and reproducibility are available at GitHub URL https://github.com/f48r1/spirohexane4bioisostere.

Molecular Structure Evaluation

Shape evaluation

Molecular shape is commonly evaluated by the normalized principal moments of inertia, defined as:

$$PM_x = \frac{I_x}{I_z} \; ; \; PM_y = \frac{I_y}{I_z}$$

where Ix, Iy and Iz are the moment of inertia along x, y and z axes, respectively.

The triangle vertices of coordinates (0,1), (0.5, 0.5) and (1,1) represent rod, disc and sphere molecular shapes, respectively.

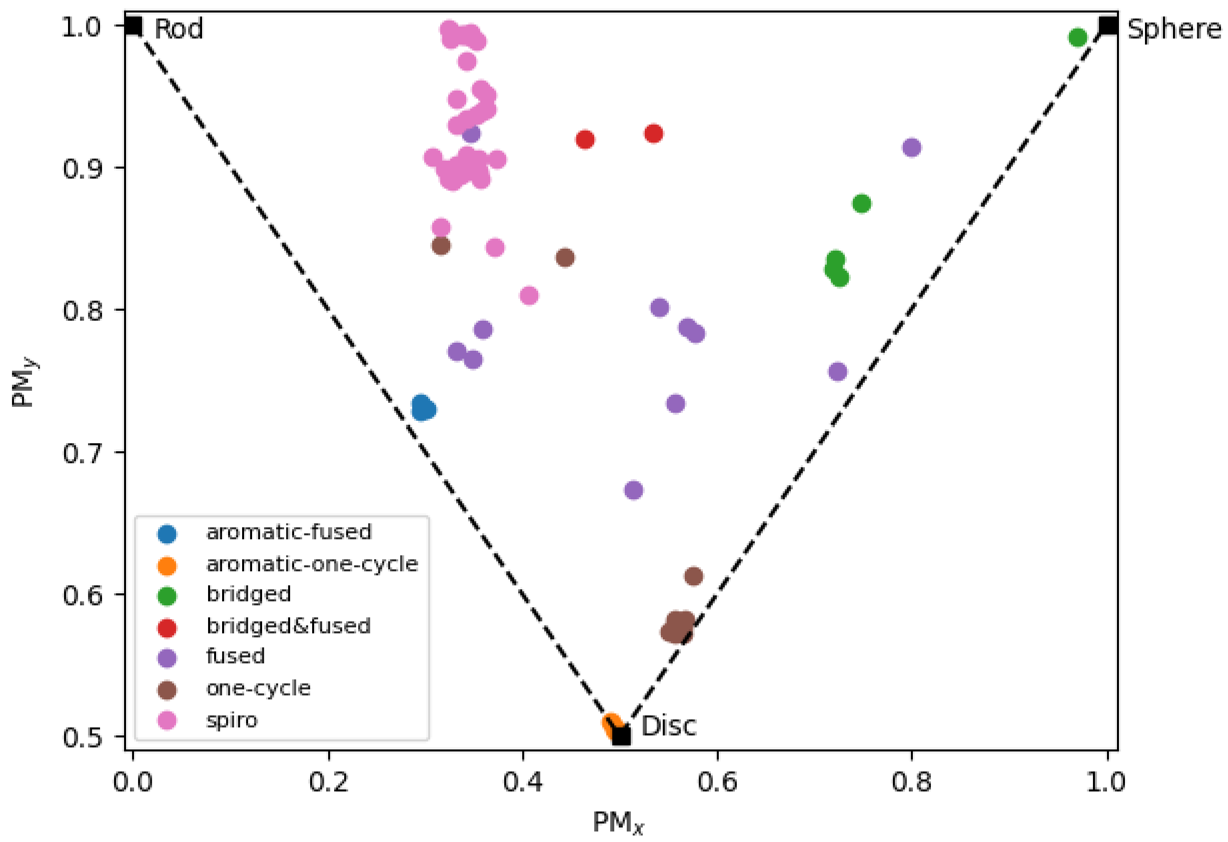

Figure 10. Scatter plot of normalized principal moments of inertia for each structure colored-based on its cycle-type.

The sum of the normalized principal moments is equal to the 3D score, whose distribution is shown in Figure 11.

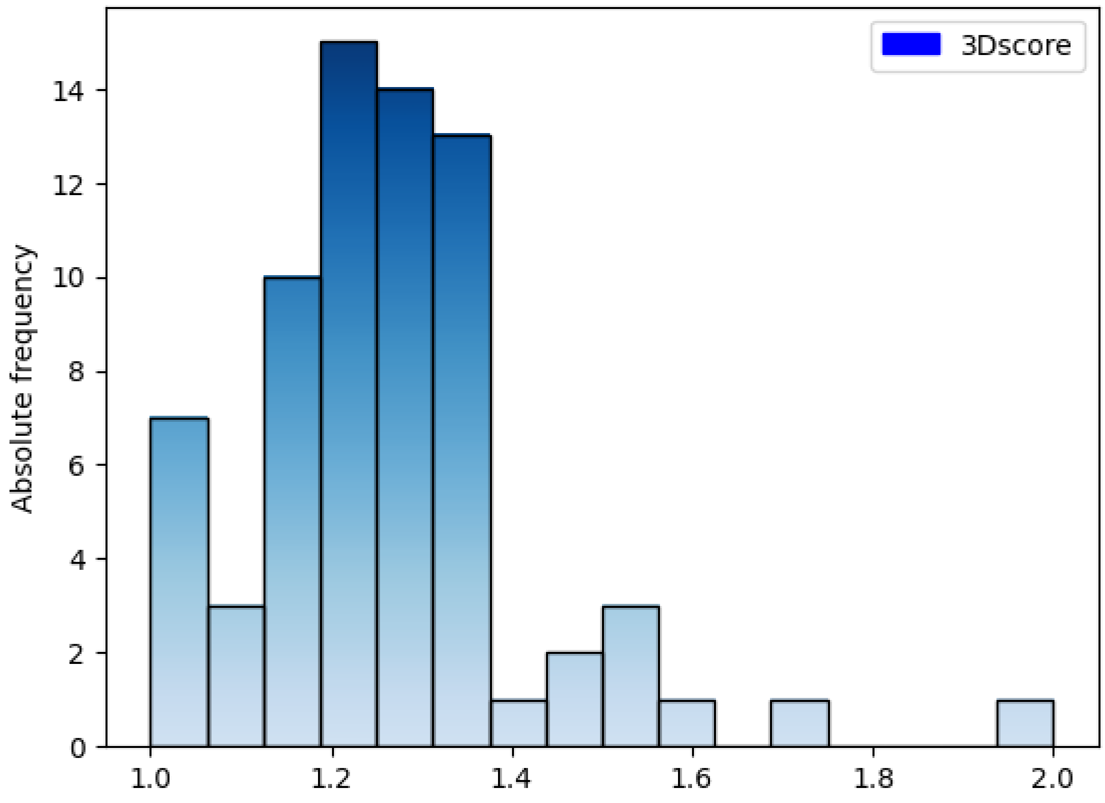

Figure 11. Distribution of 3D score.

Drug-likeness descriptors

The plane of best fit (PBF) quantifies and characterizes the 3D character of molecules. The quantitative estimate of drug likeness (QED) provides an overall measure of drug efficacy.

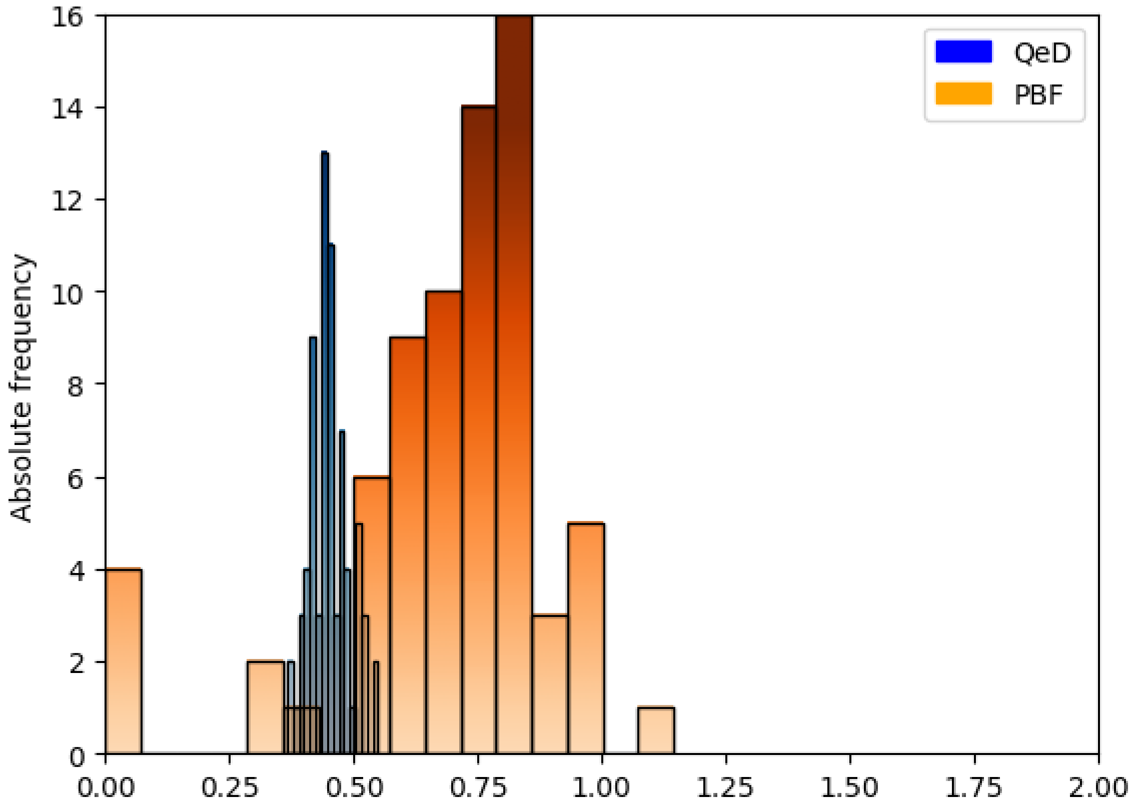

Figure 12. Distributions of QED and PBF.

The distribution in Figure 12 shows how the calculated drug-likeness varies when viewed in both 2D and 3D. Bioisosteres can therefore improve or worsen drug-likeness compared to piperidine.

List of molecular descriptors

Table 1. List of molecular descriptors. The star/hash indicates the not 3/3 dimensional character.

Descriptor	*Description*
*QeD**	Quantitative estimate of drug-likeness; evaluates how drug-like a molecule is.
*logP**	Octanol–water partition coefficient; measure of lipophilicity.
*TPSA**	Topological polar surface area; reflects polar interactions and permeability.
*SMR_VSA3**	Sum of van der Waals surface areas of atoms in a specific molar refractivity range.

*SlogP_VSA3**	Sum of van der Waals surface areas of atoms in a specific logP contribution range.
*MR**	Molar refractivity; related to polarizability and volume.
*NumNatoms**	Total number of atoms in the molecule.
*NumOatoms**	Number of oxygen atoms in the molecule.
dipole#	Molecular dipole moment; measure of molecular polarity.
3Dscore#	Three-dimensional score; quantifies molecular three-dimensionality.
PMI1#	First principal moment of inertia; reflects molecular shape.
PMI2#	Second principal moment of inertia; reflects molecular shape.
PMI3#	Third principal moment of inertia; reflects molecular shape.
NPR1#	Normalized principal moment ratio 1; dimensionless shape descriptor.
NPR2#	Normalized principal moment ratio 2; dimensionless shape descriptor.
RadiusOfGyration#	Radius of gyration; measure of molecular size and distribution of mass.
InertialShapeFactor#	Shape descriptor derived from principal moments of inertia.
Eccentricity#	Shape descriptor indicating elongation of molecular structure.
Asphericity#	Shape descriptor indicating deviation from spherical shape.
SpherocityIndex#	Shape descriptor measuring closeness to a sphere.
PBF#	Plane of best fit; quantifies 3d drug-likeness of a molecule.

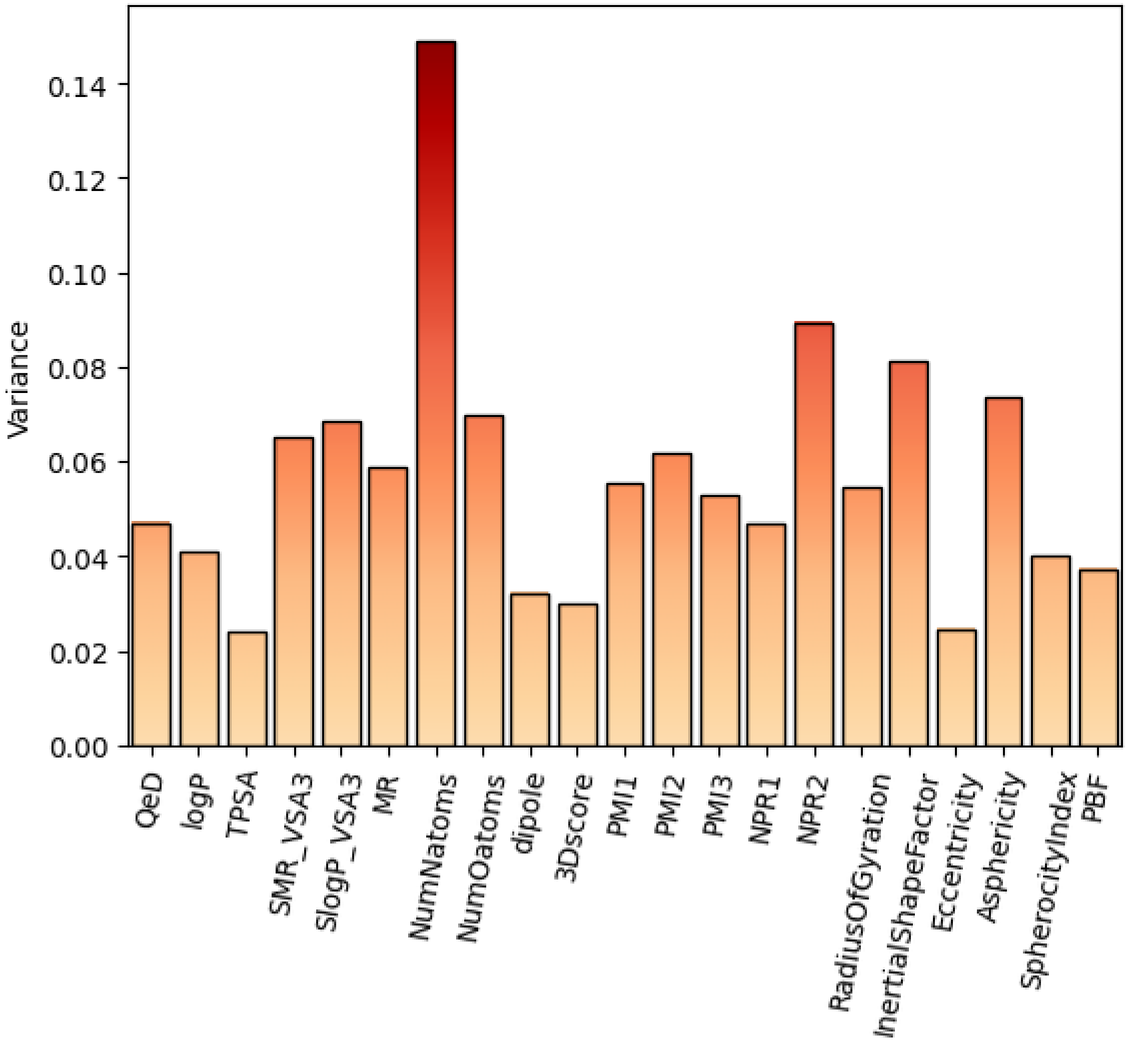

Figure 13. Variance values of 0 -1 normalized descriptors.

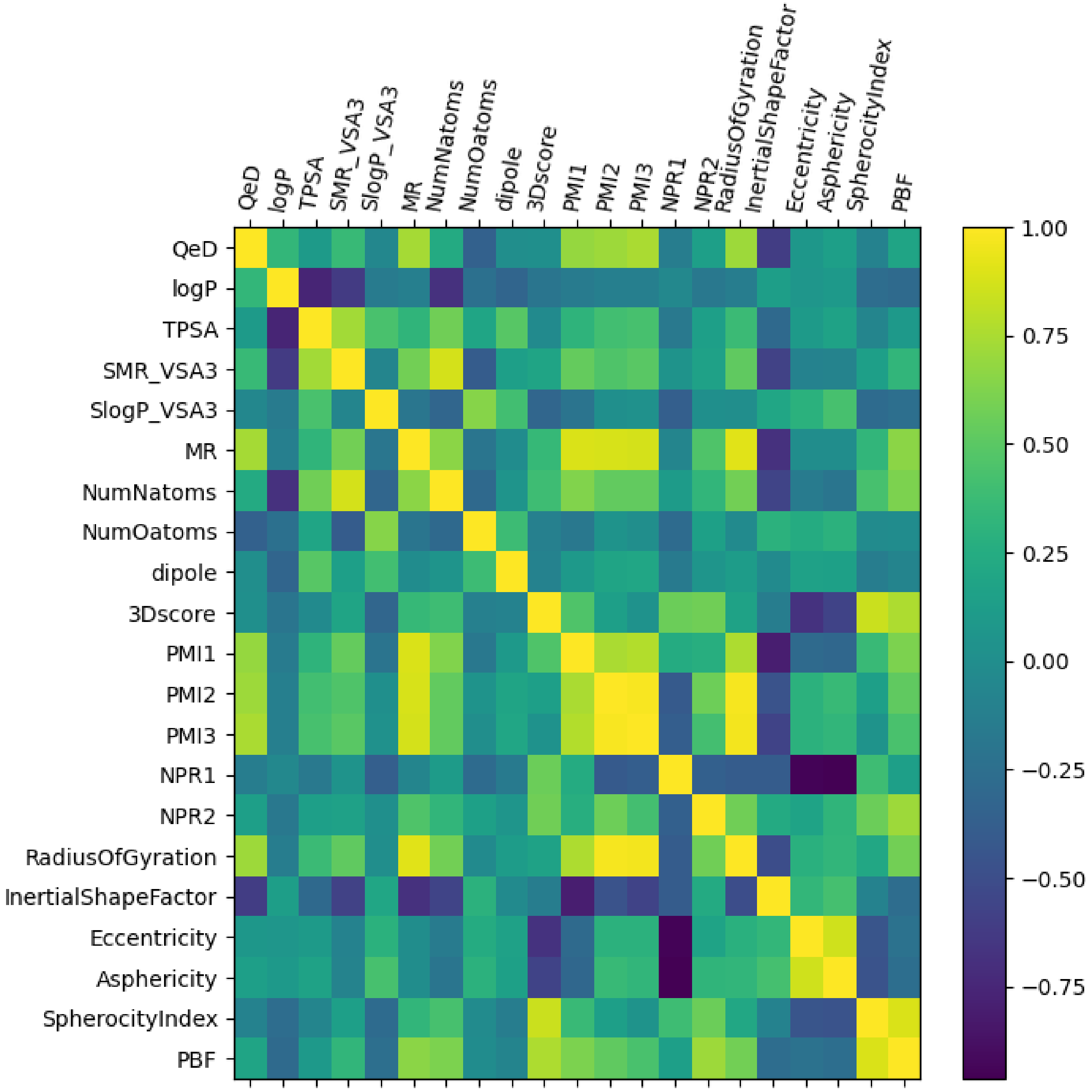

Figure 14. Correlation of each couple of descriptors.

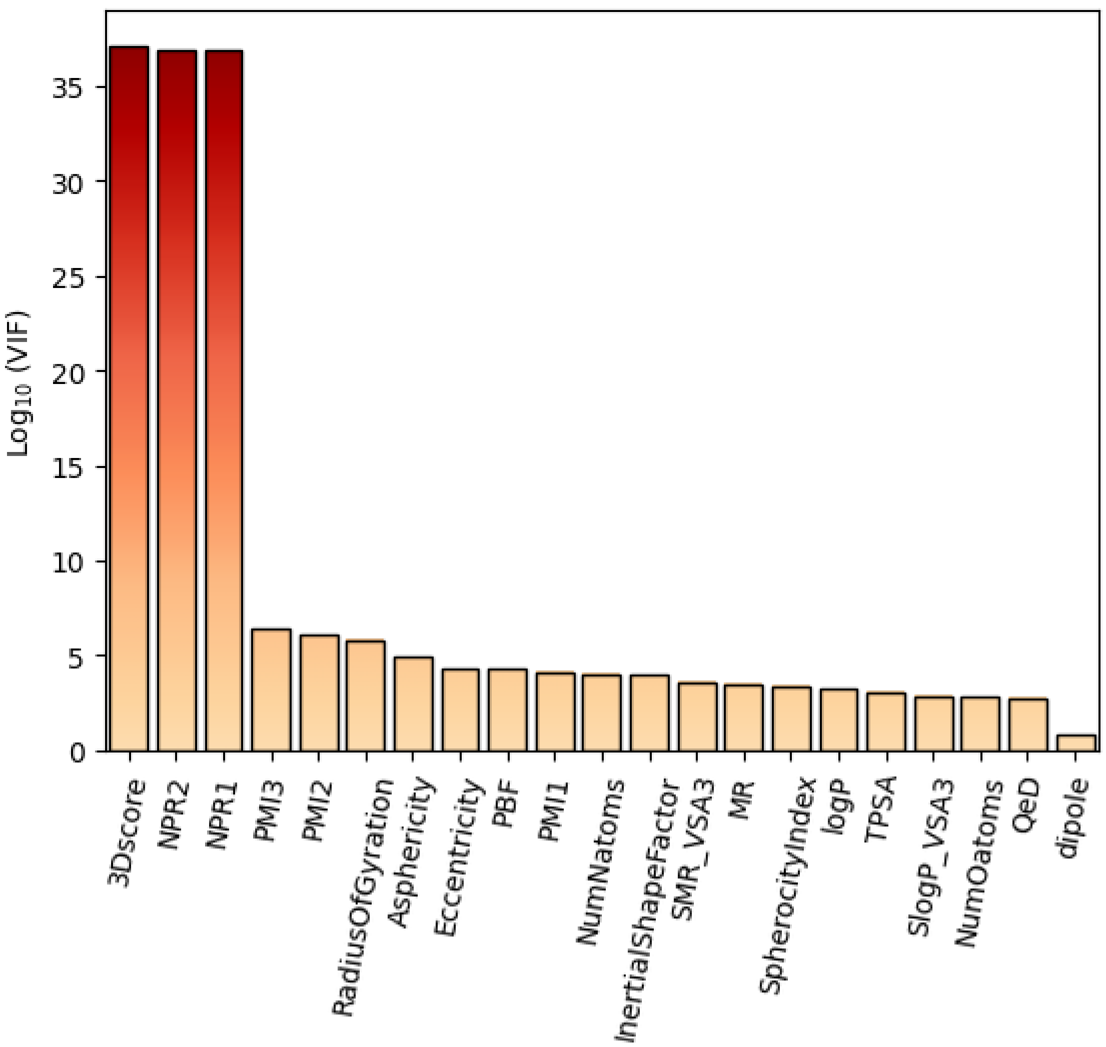

Figure 15. Logarithmic variance inflation factor of 0-1 normalized descriptors.

The descriptors shown in Table 1 have been adopted to characterize all organic cores by quantifying chemical-physical properties that depend on both conformation and 2D structure.

Observing the variances in Figure 13, it is possible to assume that some of the descriptors adopted can be filtered because they are poorly characterized due to their low variance.

Furthermore, Figure 14 shows that there are also correlations between descriptors, confirming the filtering process to be implemented. To implement the final filtering considering these observations, we decided to adopt the $\log_{10}$(VIF). From the values shown in Figure 15, we discarded the descriptors with values greater or equal than 5. Therefore, the descriptors *3Dscore, PMI2, PMI3, NPR1, NPR2, RadiusOfGyration* will not be considered in the Principal Component Analysis (PCA).

Principal component analysis

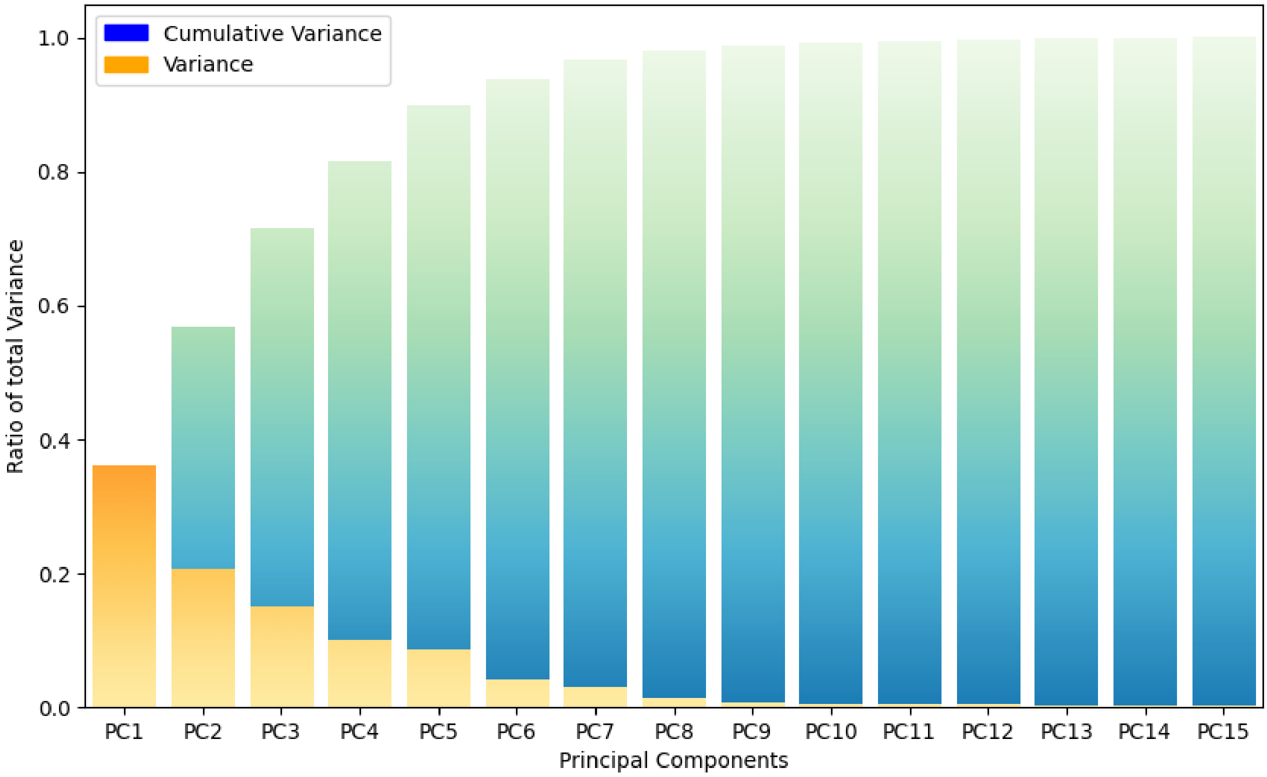

Figure 16. Variance and cumulative variance values of each principal component.

Principal Component Analysis (PCA) was performed on the generated molecular descriptor matrix. Prior to PCA, all descriptors were mean-centered and scaled to unit variance.

The PCA results (6) indicate that the first three principal components are sufficient to capture the majority of the variance (71.6%) in the dataset and provide a separation among descripted molecular patterns. On this basis, the 3D PCA space was selected as the most informative domain for subsequent clustering analyses.

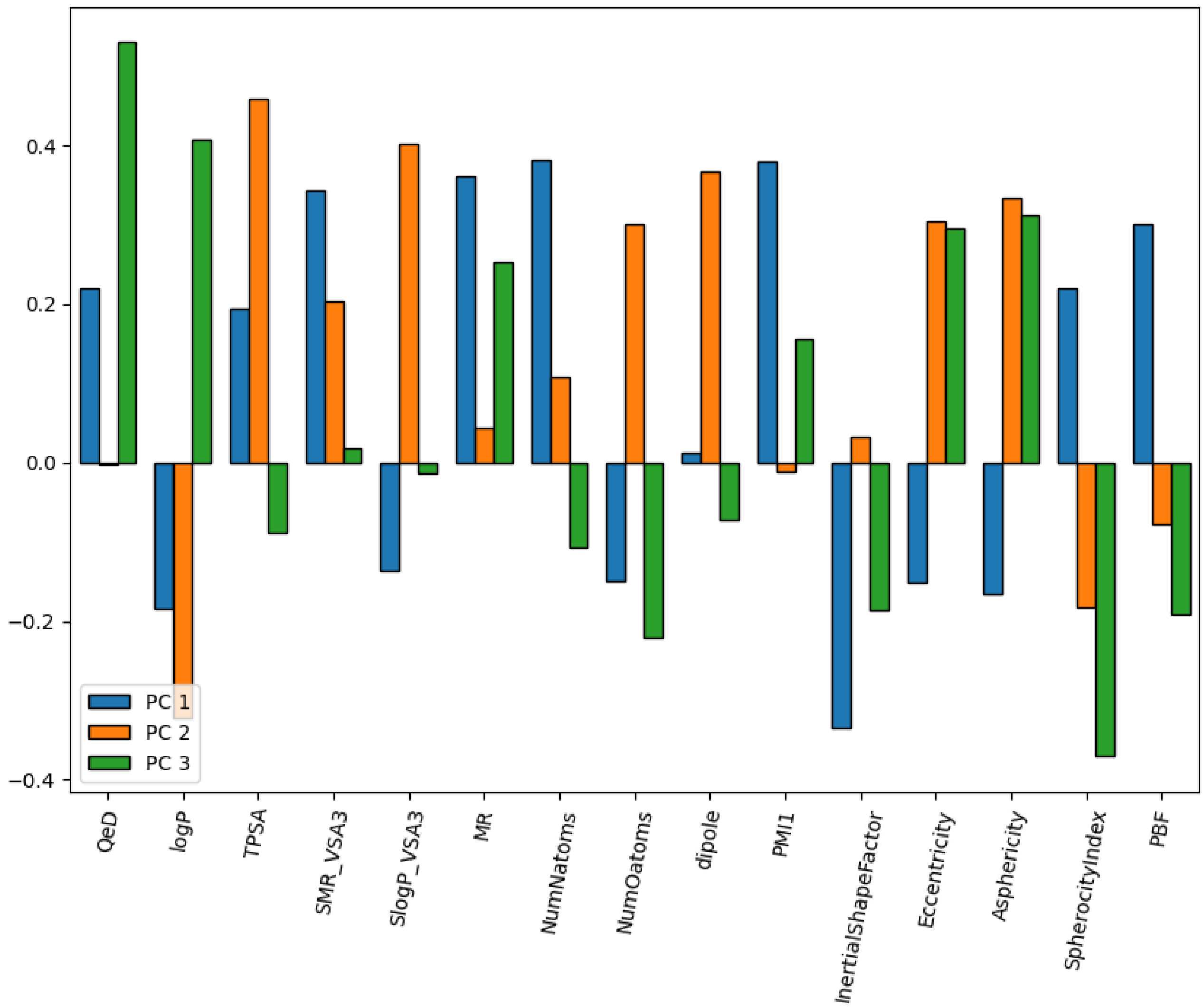

Figure 17. Loading values referring of top- three principal components.

Loading plots were examined to assess the contribution of individual descriptors to each principal component. These loadings (Figure 17) were used qualitatively to interpret the molecular features driving the separation in PCA space. Specifically, we can observe that positive PC 1 values lead to an increase in 3D drug-likeness (PBF).

Cluster analysis

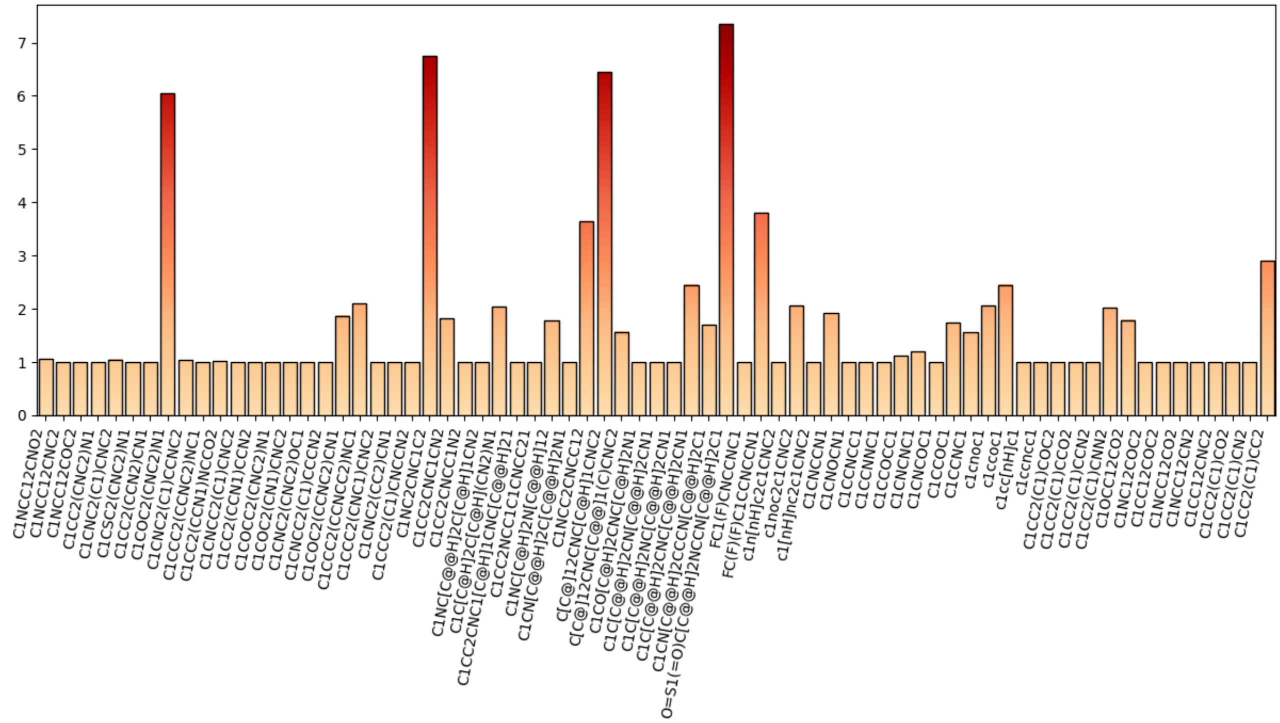

Figure 18. Absolute value of the negative outlier factor at distance of one neighbor.

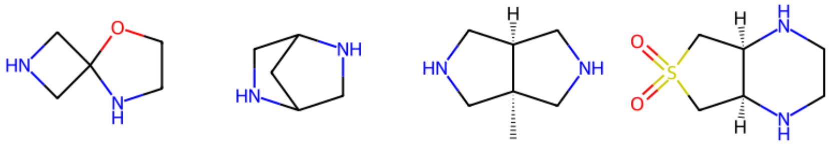

Figure 19. Organic cores not considered for clusterization phase (outliers).

Outlier detection was performed in the 3D PCA score space using a distance-based Negative Outlier Factor (NOF) approach. Specifically, for each molecular structure, the absolute value of the Negative Outlier Factor computed at a distance of one nearest neighbor was evaluated. This metric provides a local density estimation around each point: molecules with a markedly higher absolute NOF value exhibit a lower local density and are therefore more likely to represent structural or physicochemical outliers.

The analysis (Figure 18) enabled the identification of potentially anomalous organic cores, defined as those lying in sparsely populated regions of the 3D PCA space and exhibiting significantly elevated absolute NOF values. The structures with a NOF >=5 were flagged as outliers (Figure 19) for clustering approach ensuring that subsequent analyses were not biased by isolated or atypical chemotypes.

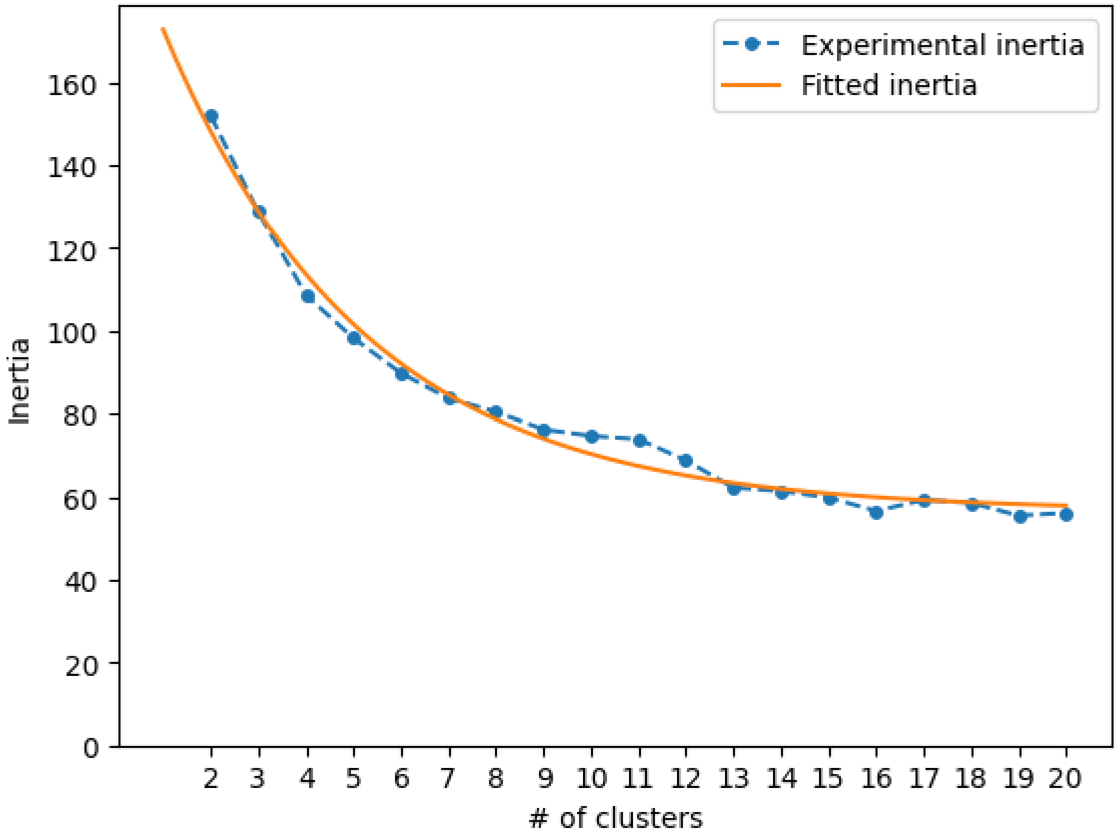

Figure 20. Inertia values of the k-Medoids algorithm varying the number of clusters.

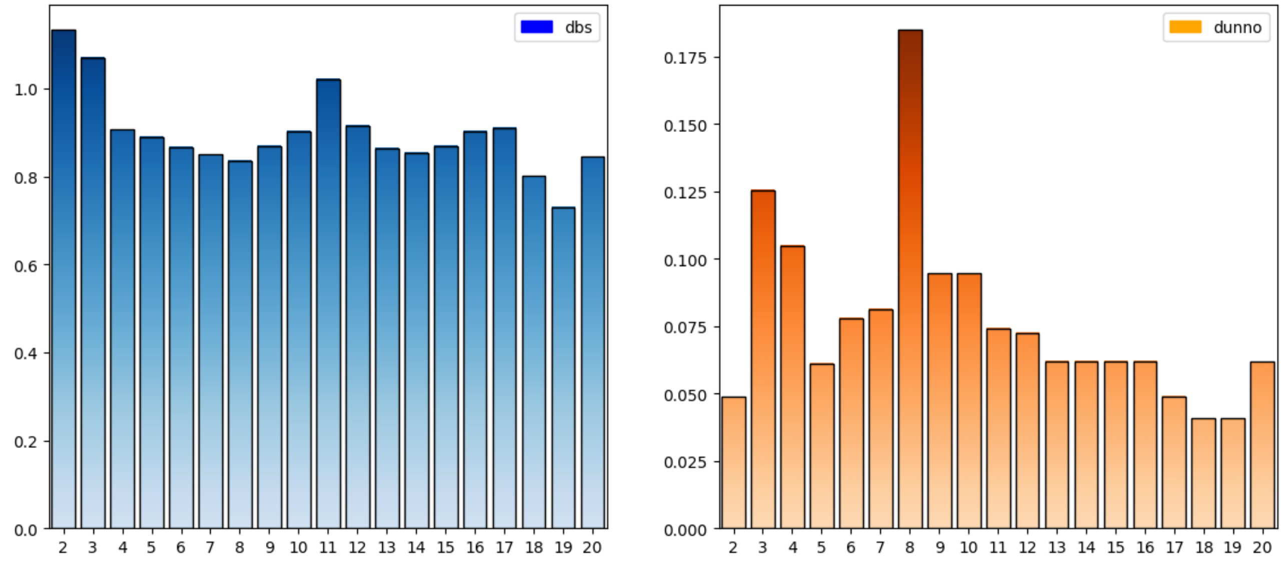

Figure 21. Davies-Bouldin score and dunno metric values of the k-Medoids algorithm.

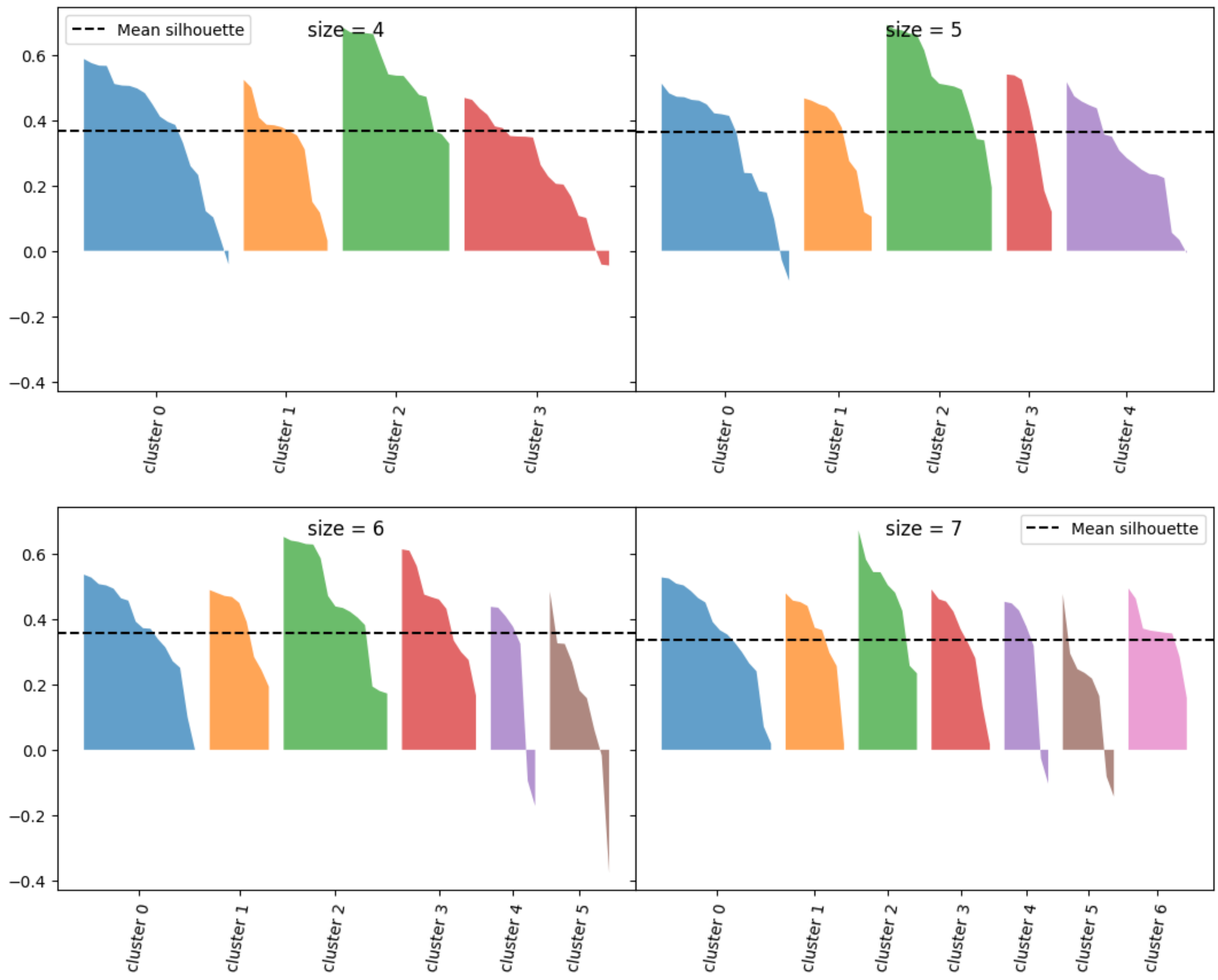

Figure 22. Blob plot of silhouette values.

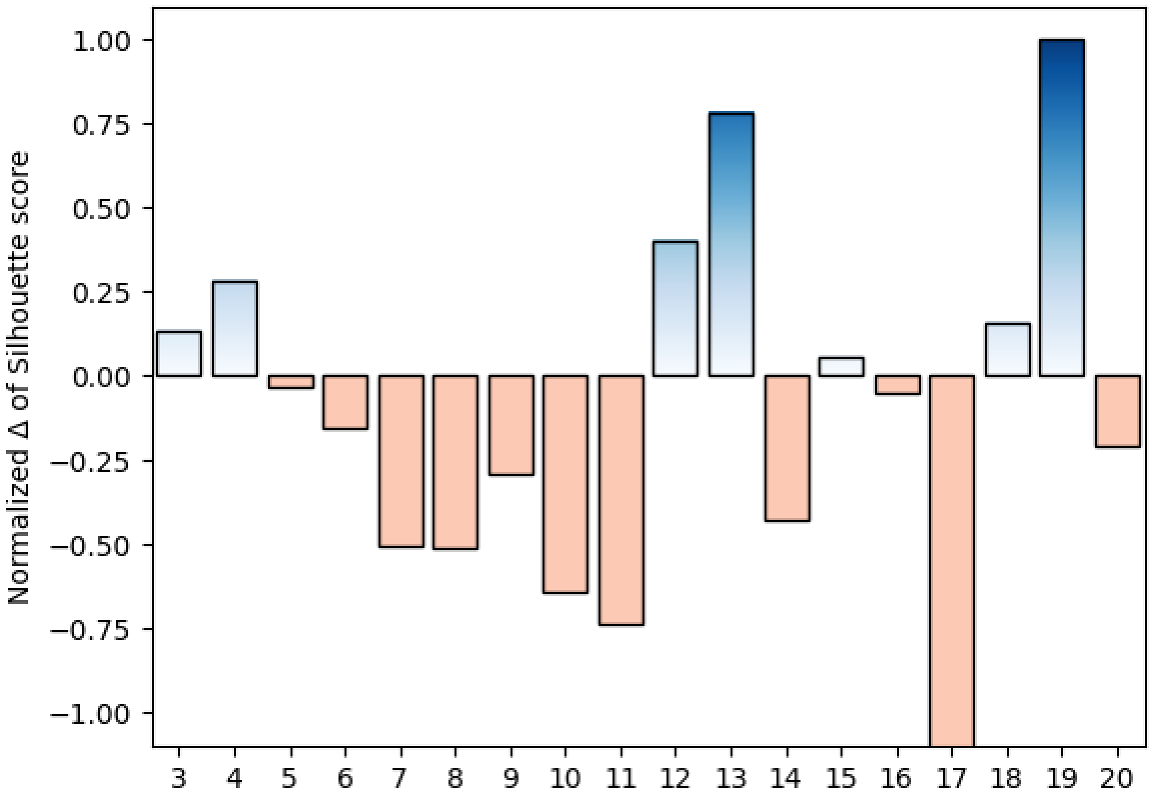

Figure 23. Bar plot of differences in mean silhouette values at the increase of the number of clusters.

Clustering was performed in the 3D PCA score space using the k-medoids algorithm ('alternate' implementation). To determine the optimal number of clusters, a silhouette analysis was conducted by computing the mean silhouette coefficient over a range of candidate cluster numbers.

The silhouette profile revealed a clear maximum at k = 5, indicating that a five-cluster partitioning provides the most coherent grouping of molecular structures within the PCA space.

13. References

[1] M. C. T. Fyfe, C. M. Rasamison, "Reductive Thiolation Approach to Pure Cyclobutyl Phenyl Sulfide" *Org. Prep. Proced. Int.* **2005**, 37, 194–197.

[2] M. Schäfer, T. Stünkel, C. Daniliuc, R. Gilmour, "Regio- and Enantioselective Intermolecular Aminofluorination of Alkenes via Iodine(I)/Iodine(III) Catalysis", *Angew. Chem. Int. Ed.,* **2022**, 61, e202205508

[3] T. Fujita, M. Takazawa, K. Sugiyama, N. Suzuki, J. Ichikawa, "Domino C-F Bond Activation of the CF3 Group: Synthesis of Fluorinated Dibenzo[a,c][7]annulenes from 2-(Trifluoromethyl)-1-alkenes and 2,2′-Diceriobiaryls" *Org. Lett.,* **2017**, 19, 588–591.

[4] M. Aelterman, T. Biremond, P. Jubault, T. Poisson, "Electrochemical Synthesis of gem-Difluoro- and γ-Fluoro-Allyl Boronates and Silanes", *Chem. Eur. J.*, **2022**, 28, e202202194.

[5] H. Kim, Y. Jung, S. H. Cho, "Defluorinative C–C Bond-Forming Reaction of Trifluoromethyl Alkenes with gem-(Diborylalkyl)lithiums" *Org. Lett.*, **2022**, 24, 2705–2710.

[6] B. S. Nader, J. A. Cordova, K. E. Reese, C. L. Powell, "A Novel Fluoride Ion Mediated Olefination of Electron-Deficient Aryl Ketones by Alkanesulfonyl Halides" *J. Org. Chem.,* **1994**, 59, 2898–2901.

[7] B. A. Sandoval, A. J. Meichan, T. K. Hyster, "Enantioselective Hydrogen Atom Transfer: Discovery of Catalytic Promiscuity in Flavin-Dependent ʼEneʼ-Reductases", *J. Am. Chem. Soc.*, **2017**, 139, 11313–11316.

[8] L. A. Bateman, T. B. Nguyen, A. M. Roberts, D. K. Miyamoto, W. M. Ku, T. R. Huffman, Y. Petri, M. J. Heslin, C. M. Contreras, C. F. Skibola, J. A. Olzmann, D. K. Nomura, "Chemoproteomics-enabled covalent ligand screen reveals a cysteine hotspot in reticulon 4 that impairs ER morphology and cancer pathogenicity", *Chem. Comm.,* **2017**, 53, 7234–7237.

[9] L. A. Bateman, T. B. Nguyen, A. M. Roberts, D. K. Miyamoto, W.-M. Ku, T. R. Huffman, Y. Petri, M. J. Heslin, C. M. Contreras, C. F. Skibola, J. A. Olzmann, D. K. Nomura, "Chemoproteomics-enabled covalent ligand screen reveals a cysteine hotspot in reticulon 4 that impairs ER morphology and cancer pathogenicity", *Chem. Comm.,* **2017**, 53, 7234–7237.

[10] K. Zhang, L. Chang, Q. An, X. Wang, Z. Zuo, "Dehydroxymethylation of alcohols enabled by cerium photocatalysis", *J. Am. Chem. Soc.*, **2019**, 141, 10556–10564.

[11] S. Lee, I. Shin, "Reversed-Polarity Synthesis of N-Sulfonyl Ketimines with Imidoylsilanes and Diaryliodonium Salts via Palladium-Catalyzed Reactions", *J. Org. Chem.,* **2022**, 87, 6552–6561.

[12] G. Mloston, H. Heimgartner, "The First Reaction of Dimethoxycarbene with an Imine Moiety" *Helv. Chim. Acta*, **2007**, 90, 1758-1764.

[13] R. N. Ram, A. A. Khan, "A Simple Method for the Preparation of N-Sulfonylimines by Direct Condensation of p-Toluenesulfonamide with Diaryl Ketones" *Synth. Commun.* **2001**, 31, 841–846.

[14] F. Soddu, I. Mahdi, M. C. Cabua, F. Secci, P. Natho, R. Tassoni, P. Dambruoso, E. Mesto, M. Colella, R. Luisi, "Generation and Use of Cyclopropenyllithium under Continuous Flow Conditions" *Org. Lett.* **2025**, 27, 5754–5759.

[15] P. Natho, M. Colella, A. Vicenti, G. Romanazzi, F. Ullah, N. S. Sheikh, A. J. P. White, F. Pasca, R. Luisi, "Shifting Lithium Amide Reactivity to the Radical Domain: Regioselective Radical C−H Functionalization of 3-Iodooxetane for the Synthesis of 1,5-Dioxaspiro[2.3]hexanes" *Angew. Chem. Int. Ed.*, **2025**, 64, e202424346.

[16] A. Sperga, T. Pfeifers, D. Zacs, J. Veliks, "Fluorohalomethylsulfonium Salts as a Fluorohalocarbene Source" *Org. Lett.*, **2024**, 26, 6482–6485.

[17] B. J. Moore, D. Willcox, "Dichloromethyl(diaryl) Sulfonium Salts as gem-Dichlorocyclopropanation Reagents" *Org. Lett.*, **2025**, 27, 1402–1406.

[18] APEX 2, version 2010.3-0, Bruker (2010), Bruker AXS Inc., Madison, Wisconsin, USA.

[19] SAINT, version V7.60A, Bruker (2009), Bruker AXS Inc., Madison, Wisconsin, USA

[20] SADABS, version 2008/1, Bruker (2008), Bruker AXS Inc., Madison, Wisconsin, USA

[21] Sheldrick, G.M. (2008) XPREP Version 2008/2. Bruker AXS Inc., Madison

[22] L. Palatinus, G. Chapuis J. Appl. Cryst. 2007, 40, 786-790

[23] R.I. Cooper, A. L. Thompson, D. J. Watkin J. Appl. Crystallogr. 2010, 43,1100-1107

[24] P. W. Betteridge, J. R. Carruthers, R. I. Cooper, K. Prout, & D. J. Watkin, J. Appl. Cryst. (2003), 36, 1487.

14. NMR spectra

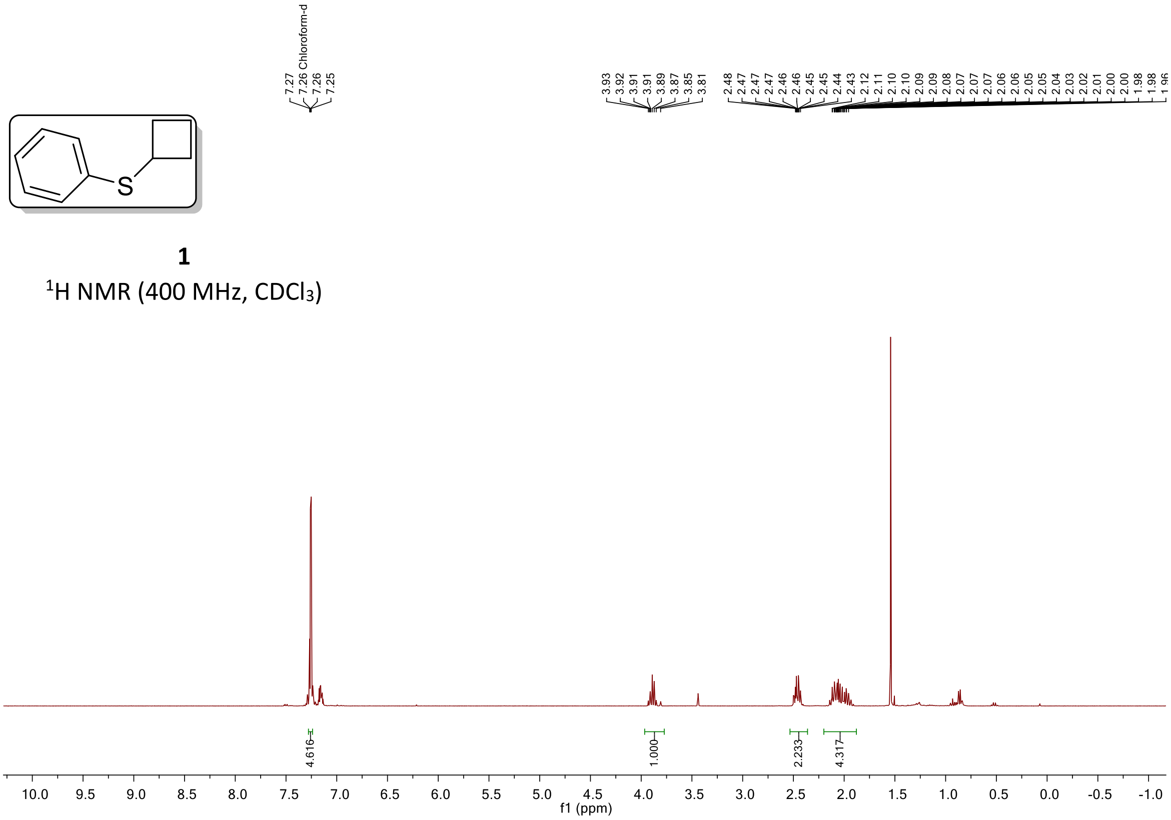

1

^{1}H NMR (400 MHz, $CDCl_3$)

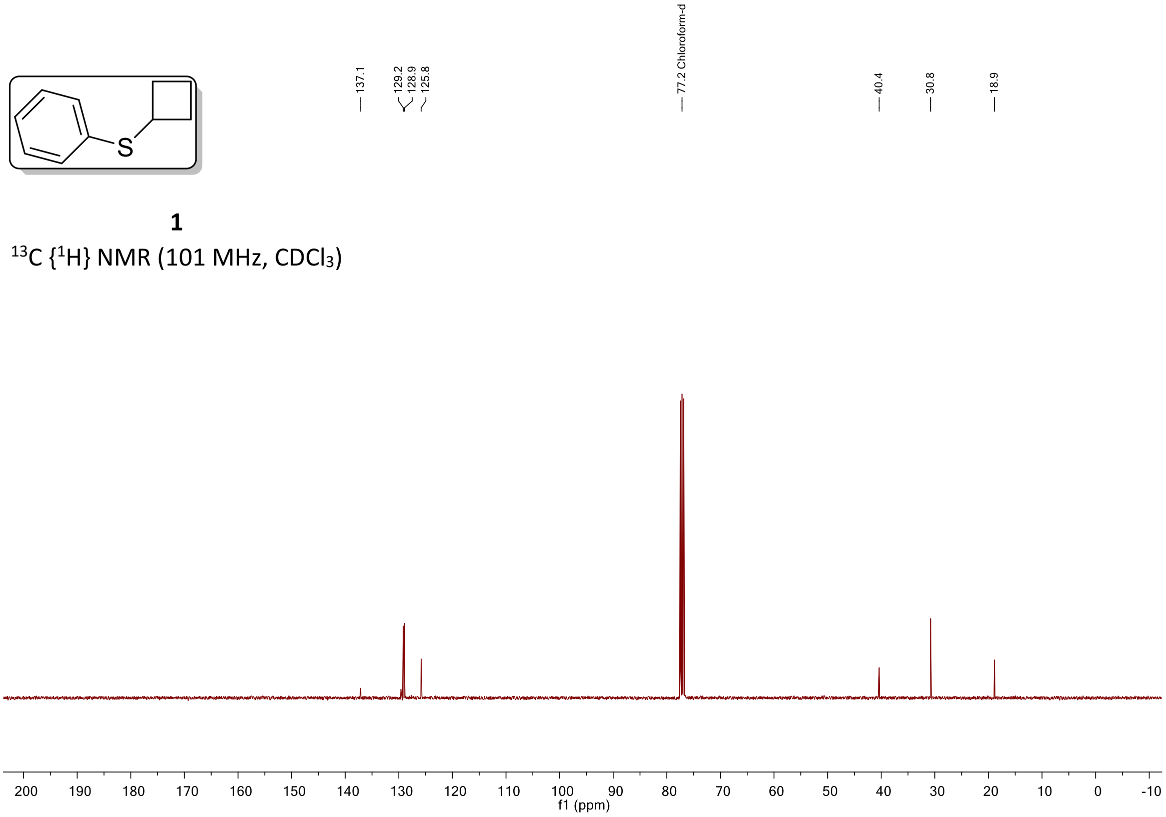

1

$^{13}C\{^{1}H\}$ NMR (101 MHz, $CDCl_3$)

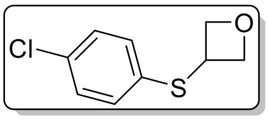

2

^{1}H NMR (400 MHz, $CDCl_3$)

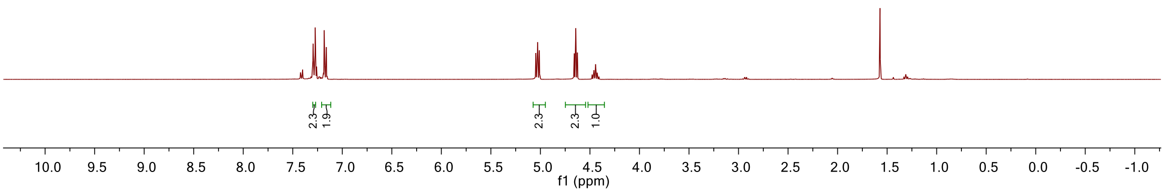

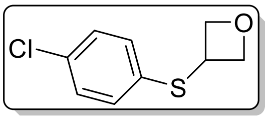

2

^{13}C {^{1}H} NMR (101 MHz, $CDCl_3$)

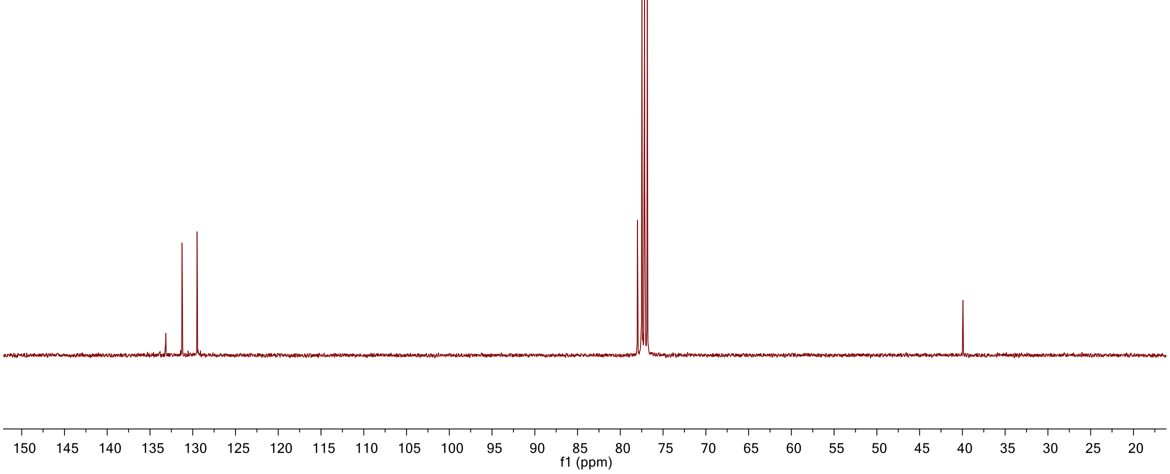

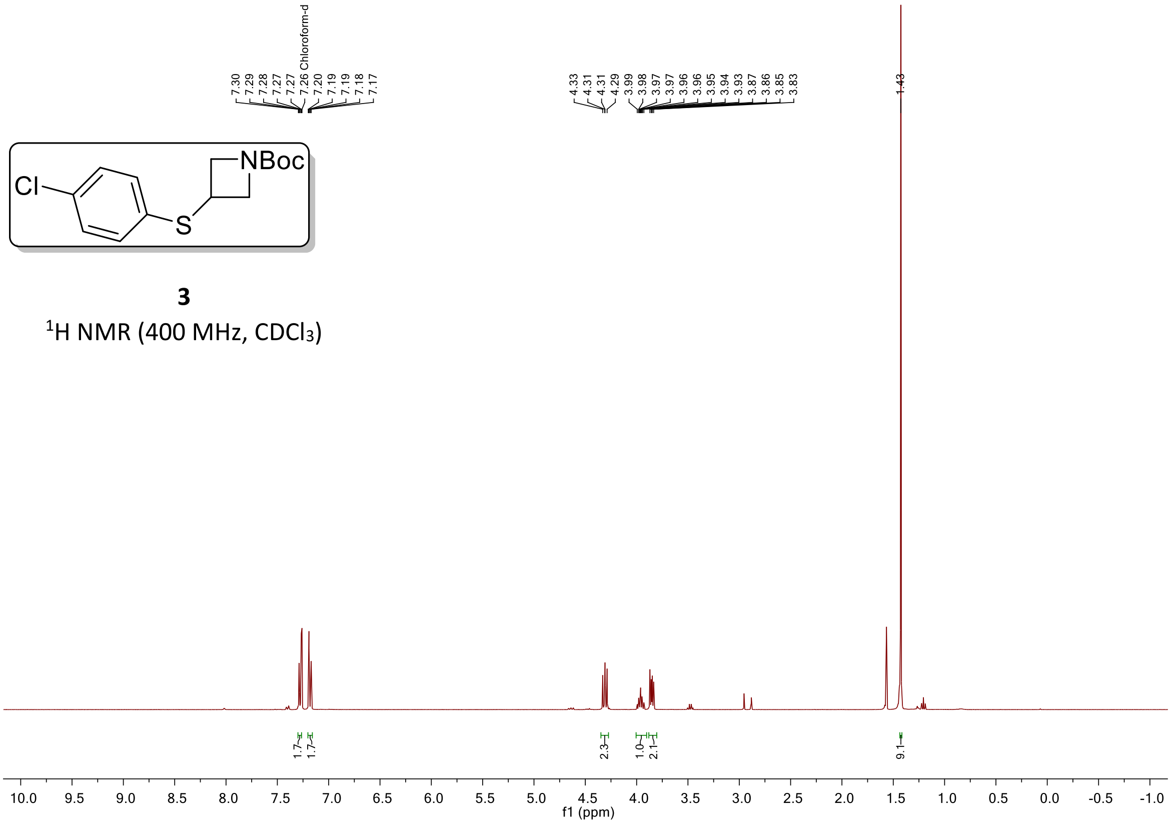

3

^{1}H NMR (400 MHz, $CDCl_3$)

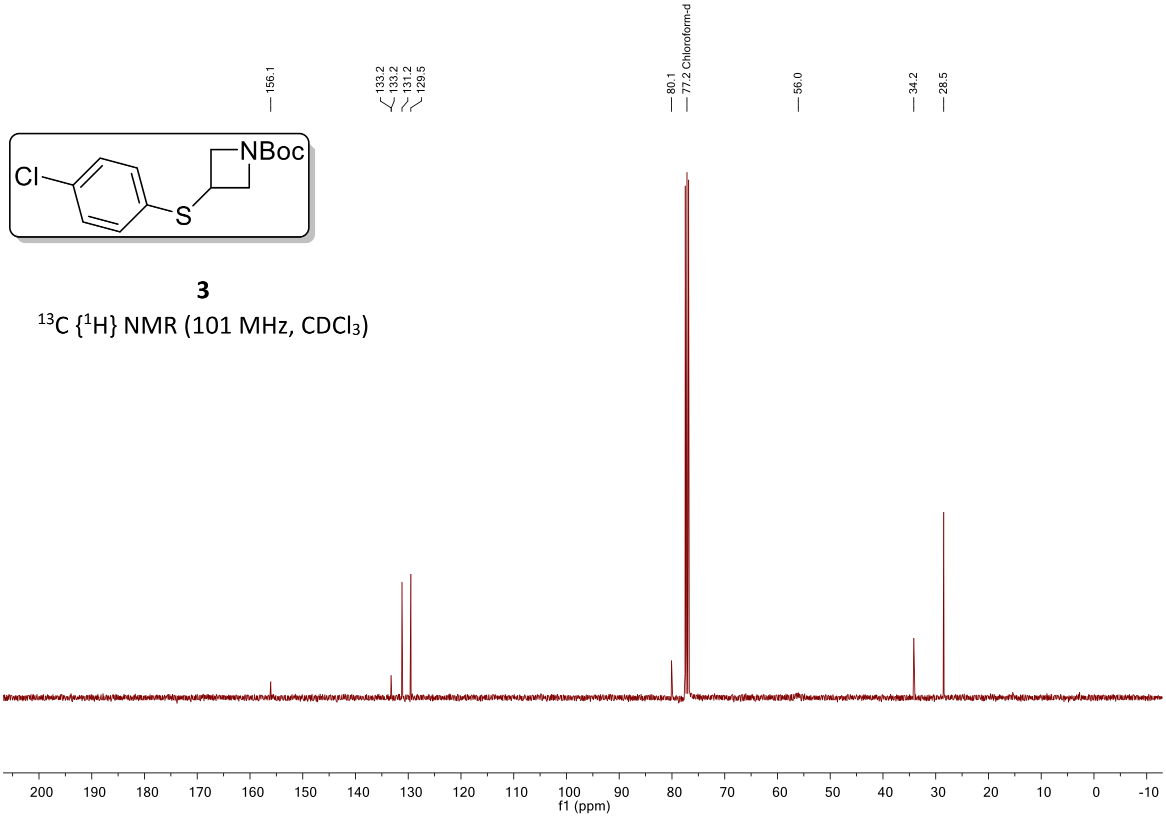

3

^{13}C {^{1}H} NMR (101 MHz, $CDCl_3$)

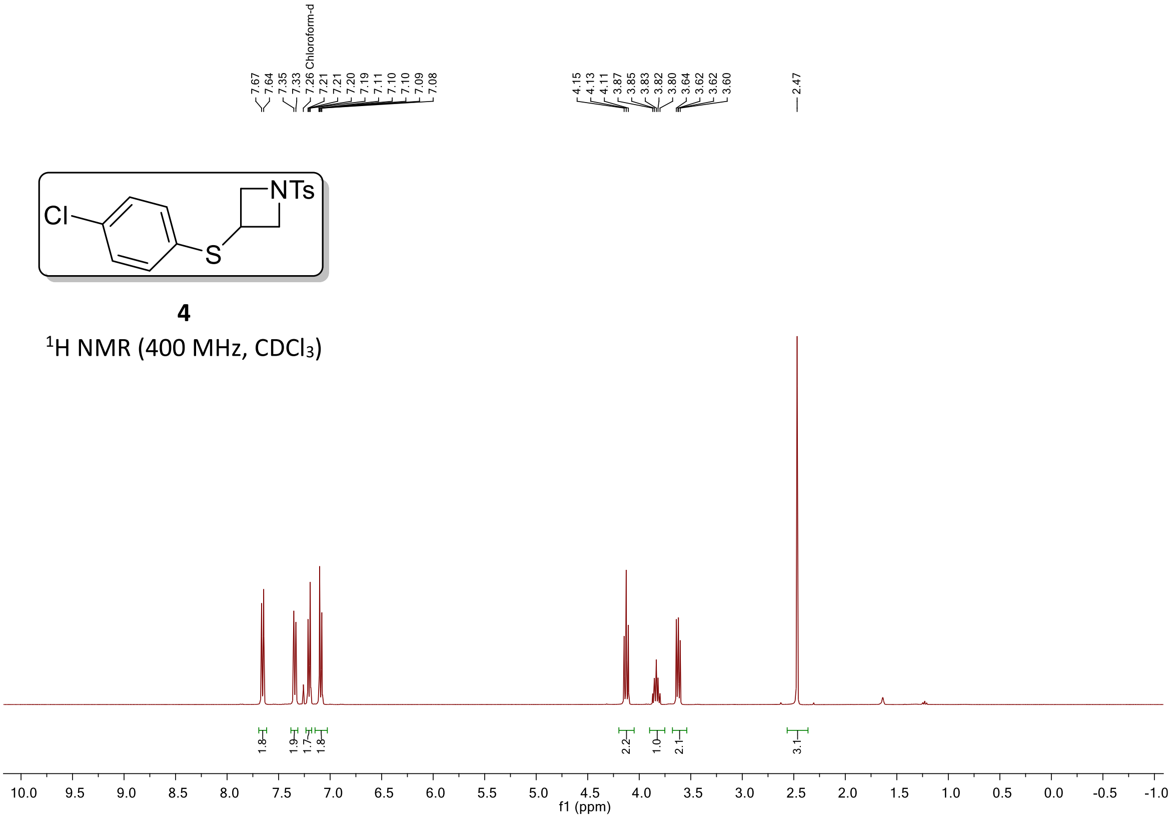

4

^{1}H NMR (400 MHz, $CDCl_3$)

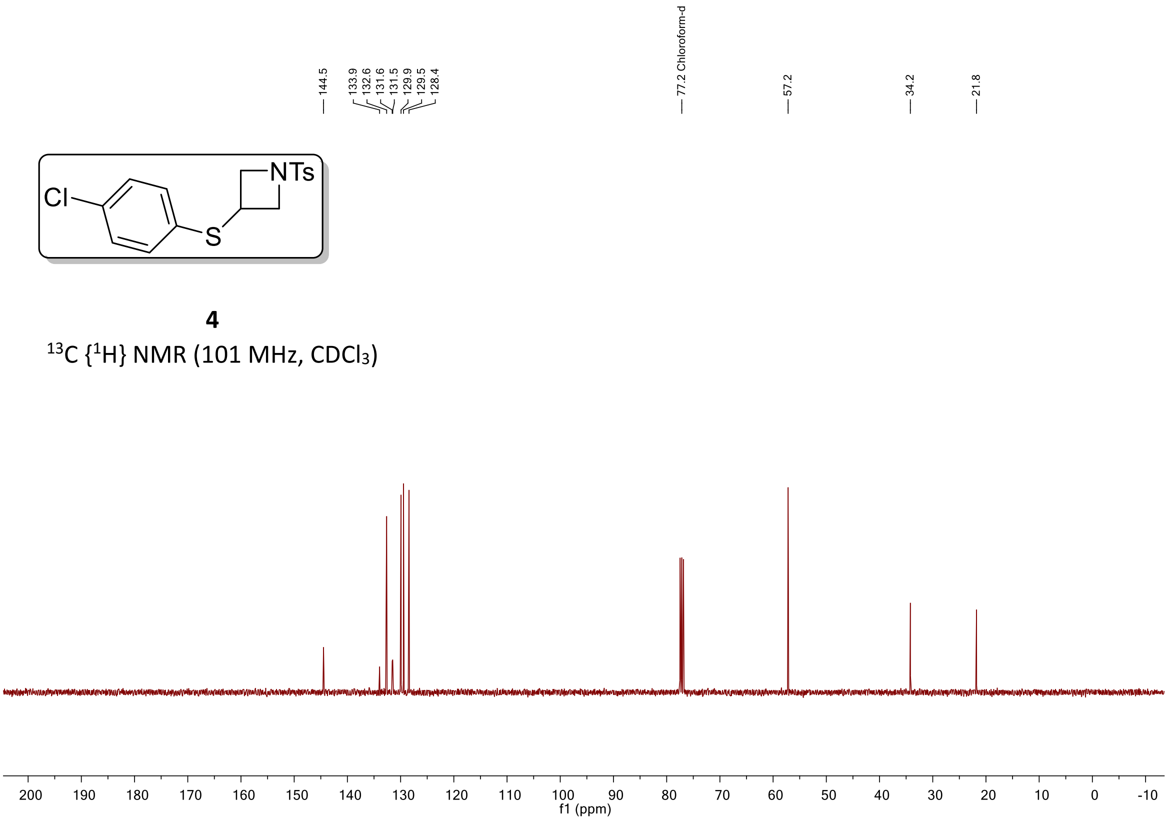

4

^{13}C {^{1}H} NMR (101 MHz, $CDCl_3$)

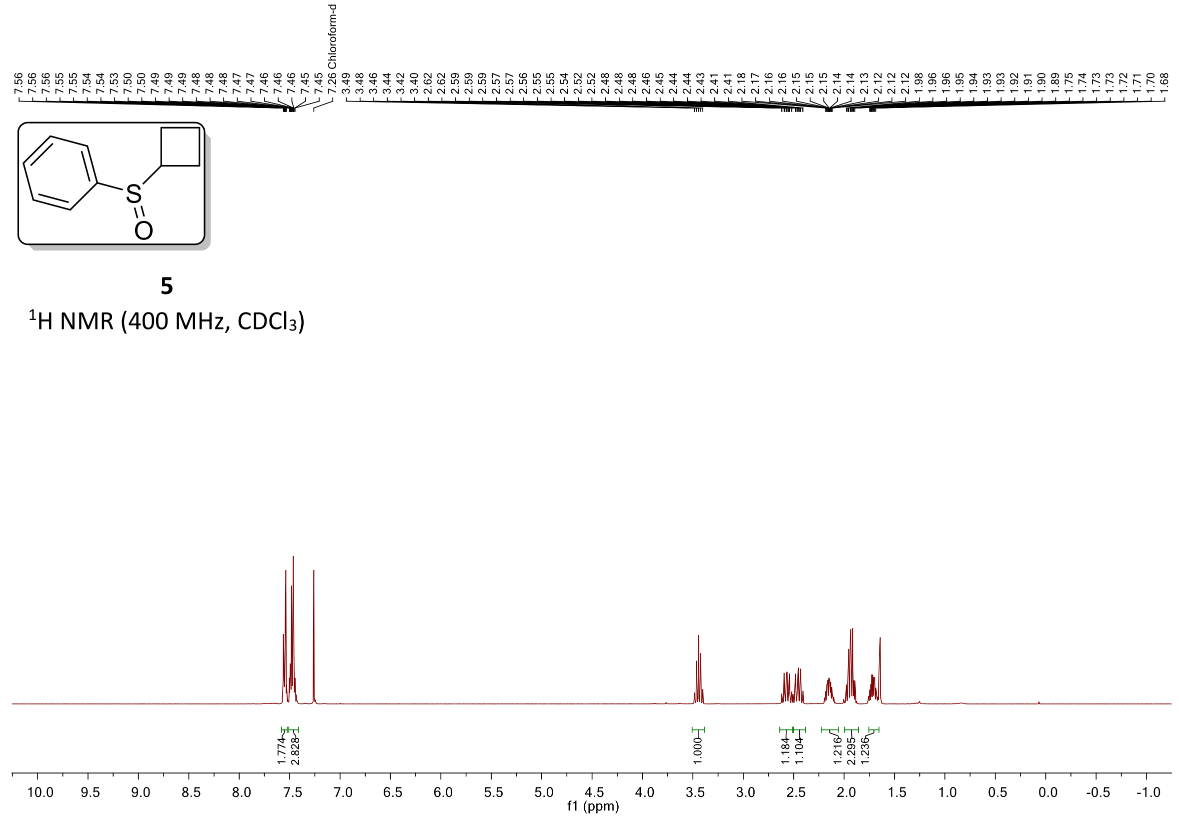

5

^{1}H NMR (400 MHz, $CDCl_3$)

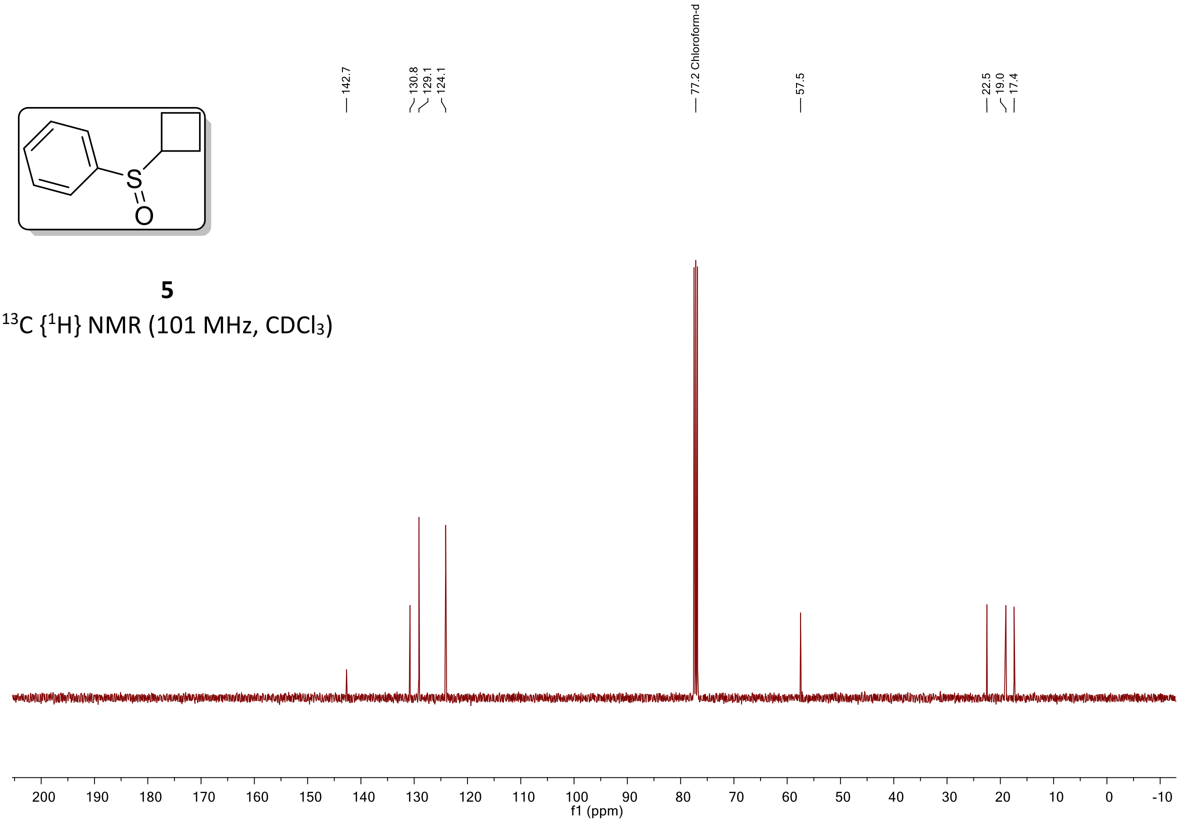

5

^{13}C {^{1}H} NMR (101 MHz, $CDCl_3$)

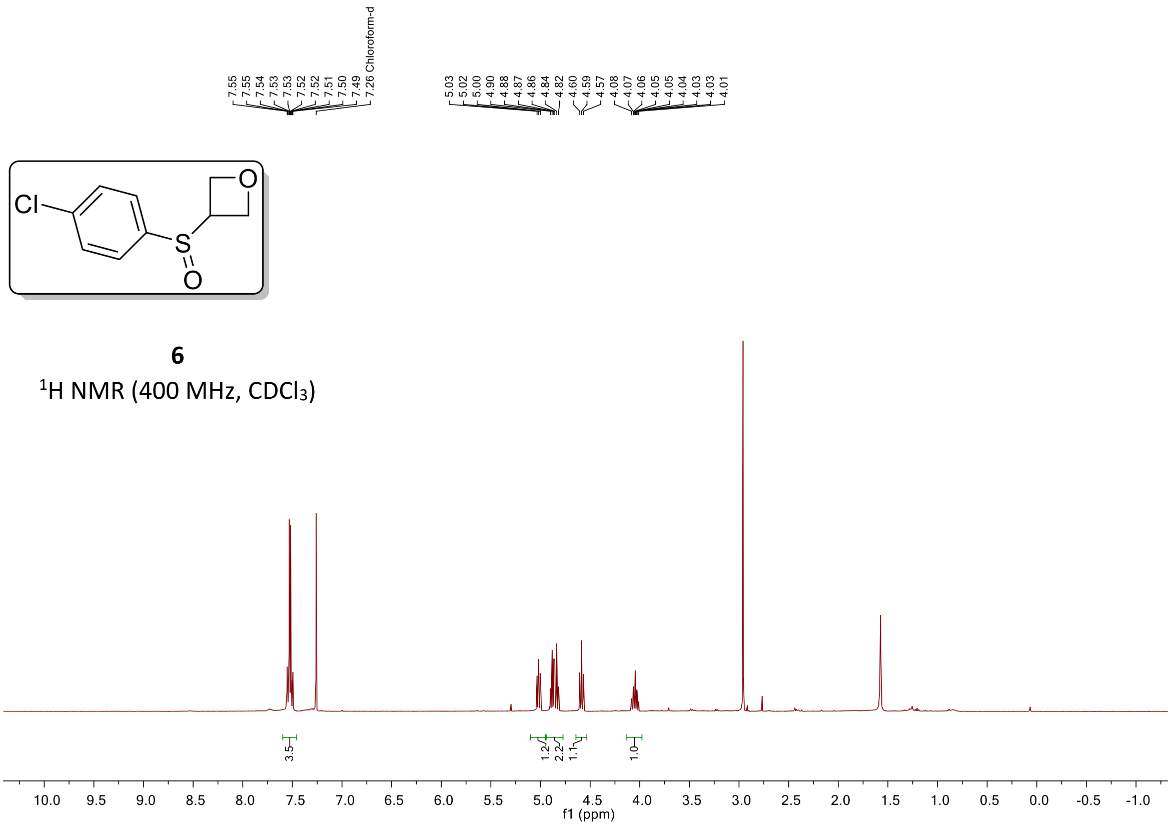

6

^{1}H NMR (400 MHz, $CDCl_3$)

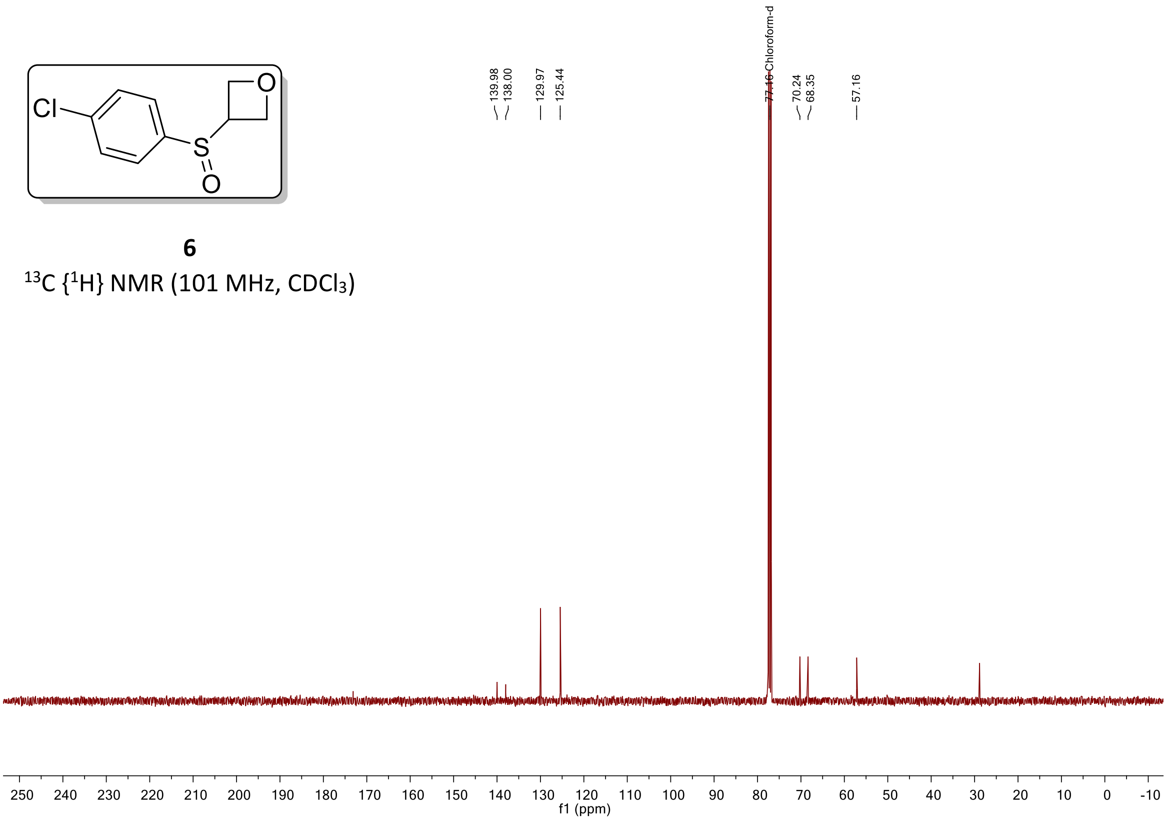

6

^{13}C {^{1}H} NMR (101 MHz, $CDCl_3$)

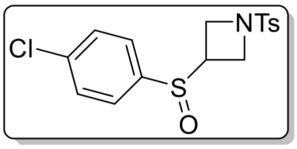

7

^{1}H NMR (400 MHz, $CDCl_3$)

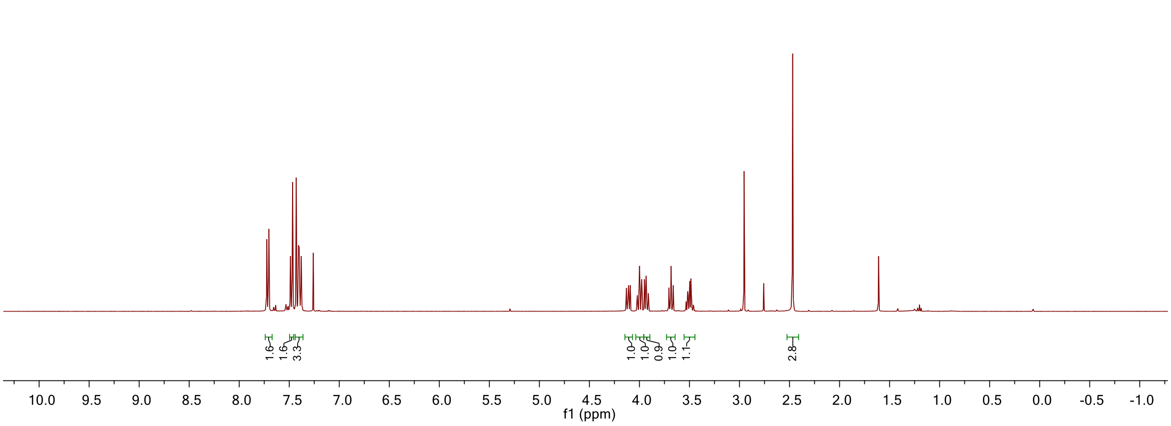

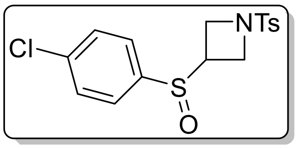

7

^{13}C {^{1}H} NMR (101 MHz, $CDCl_3$)

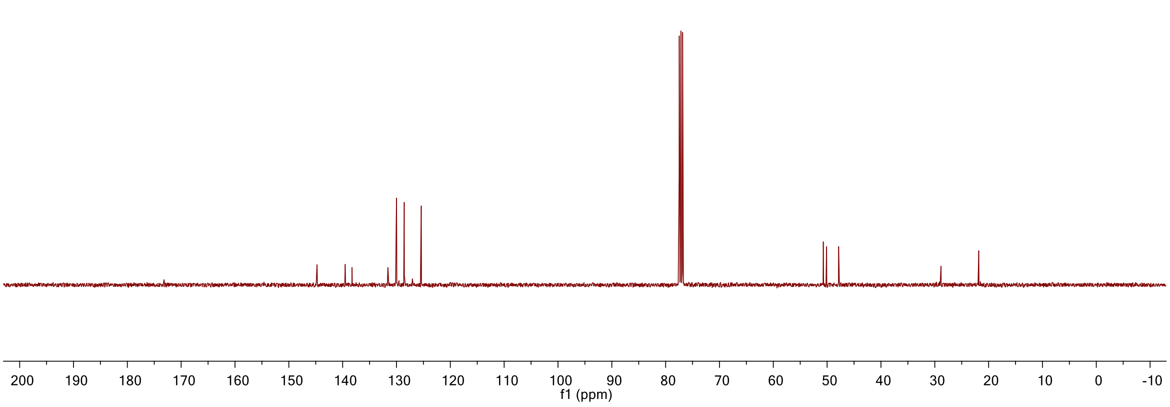

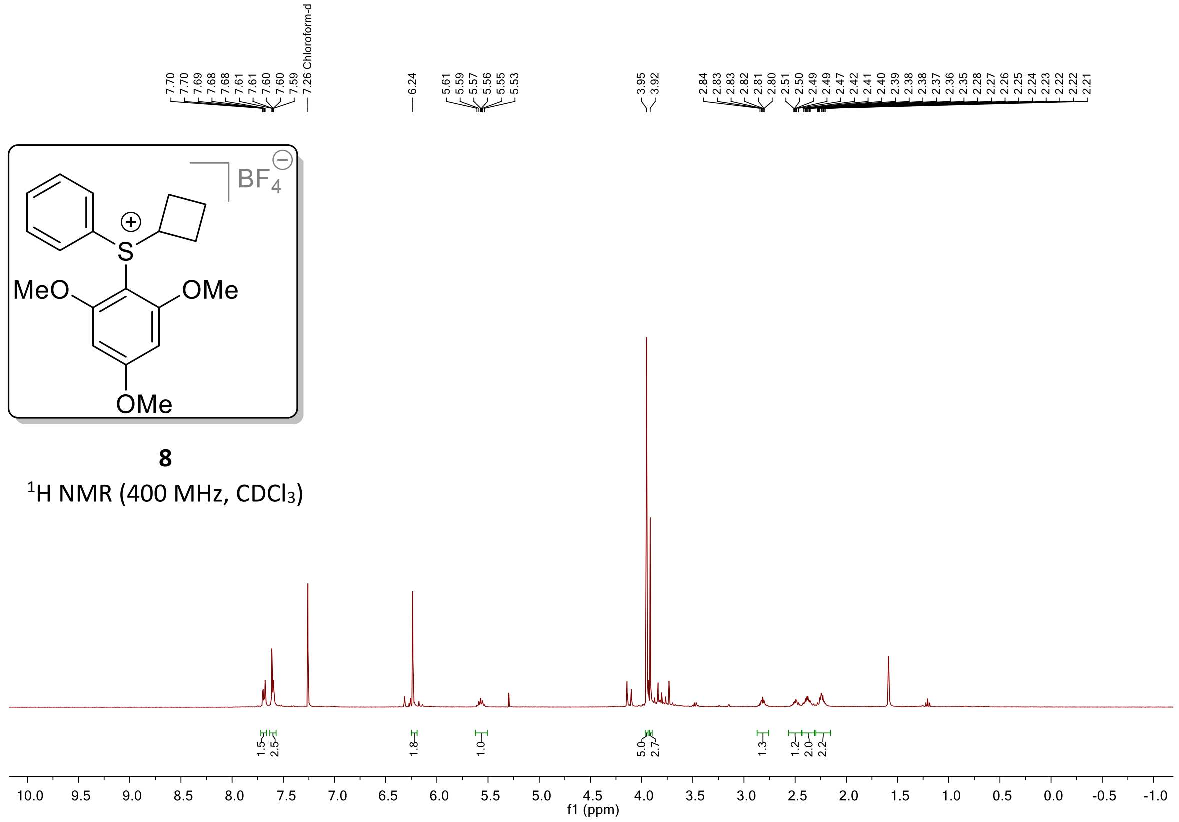

8

^{1}H NMR (400 MHz, $CDCl_3$)

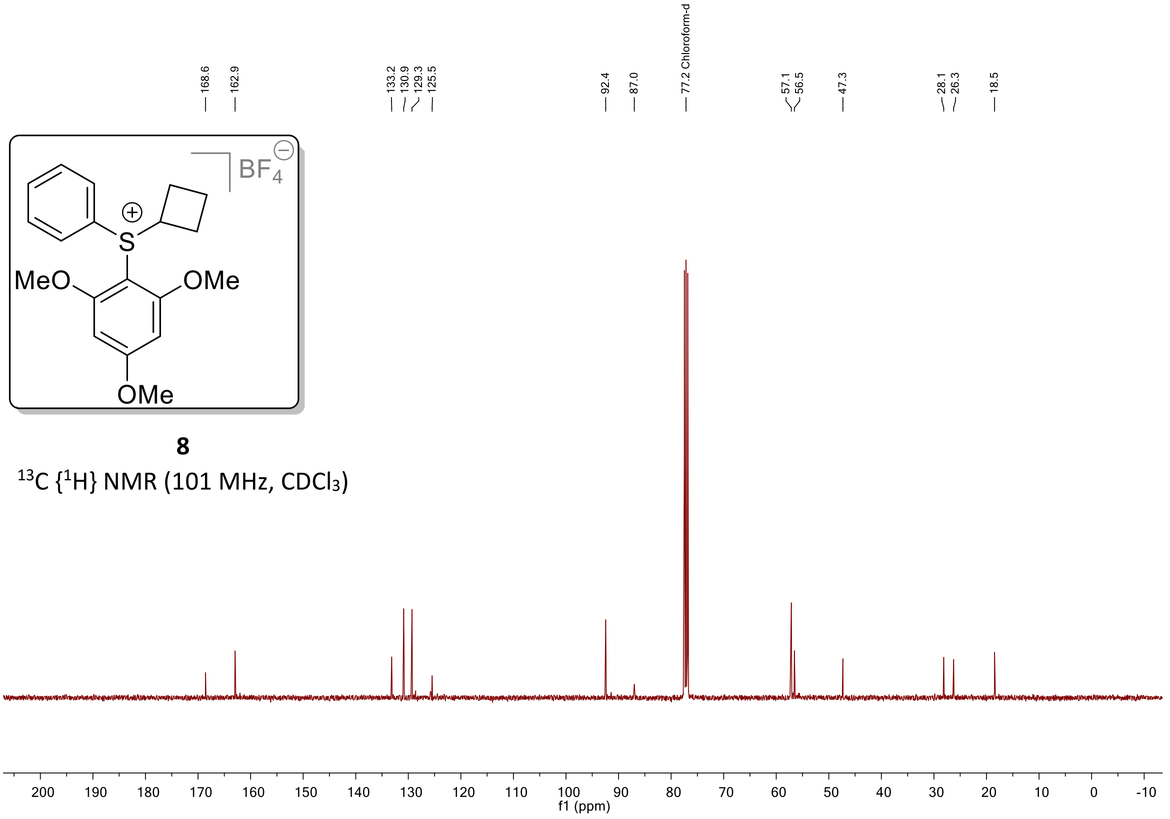

8

^{13}C {^{1}H} NMR (101 MHz, $CDCl_3$)

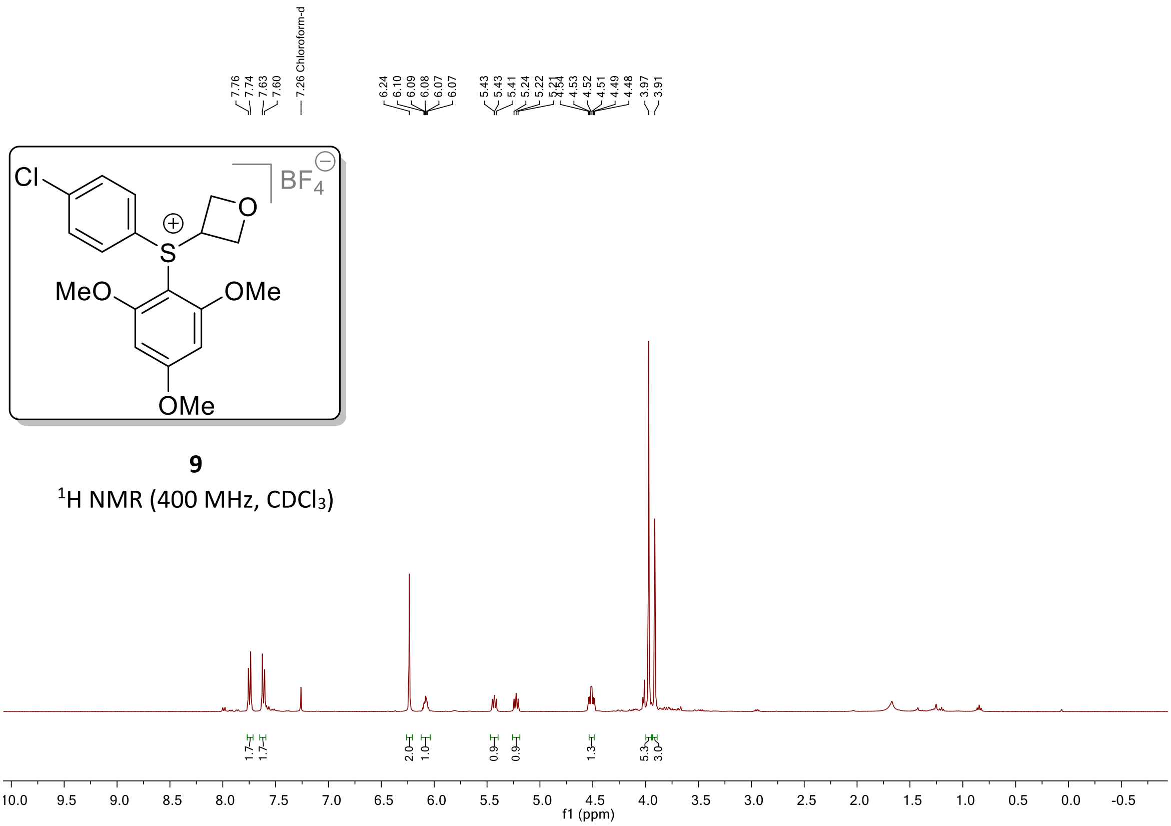

9

^{1}H NMR (400 MHz, $CDCl_3$)

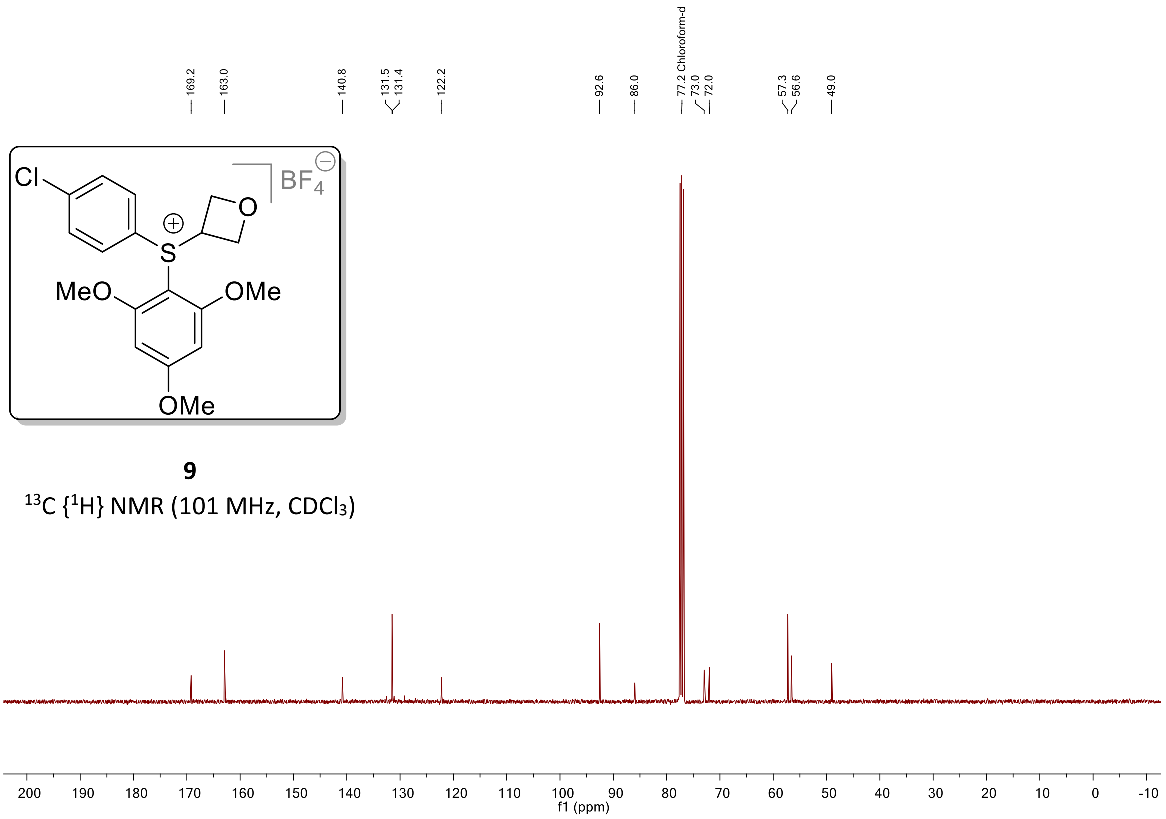

9

^{13}C {^{1}H} NMR (101 MHz, $CDCl_3$)

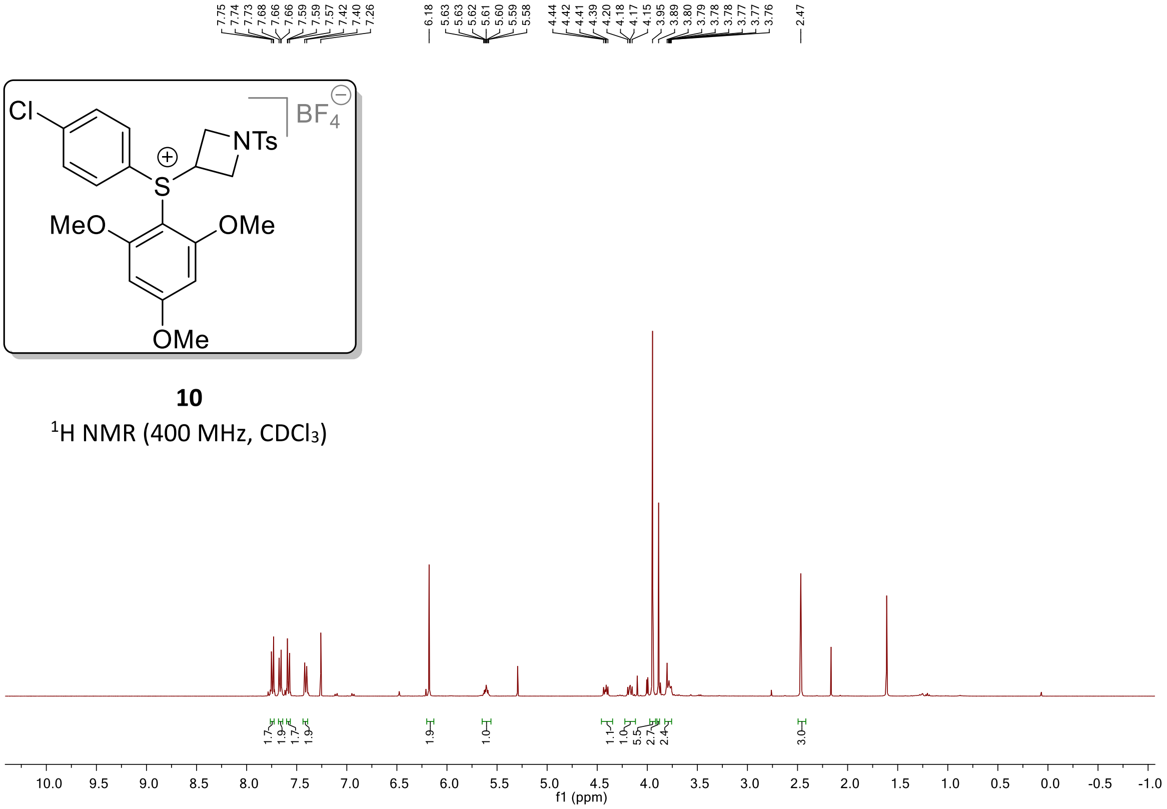

10

^{1}H NMR (400 MHz, $CDCl_3$)

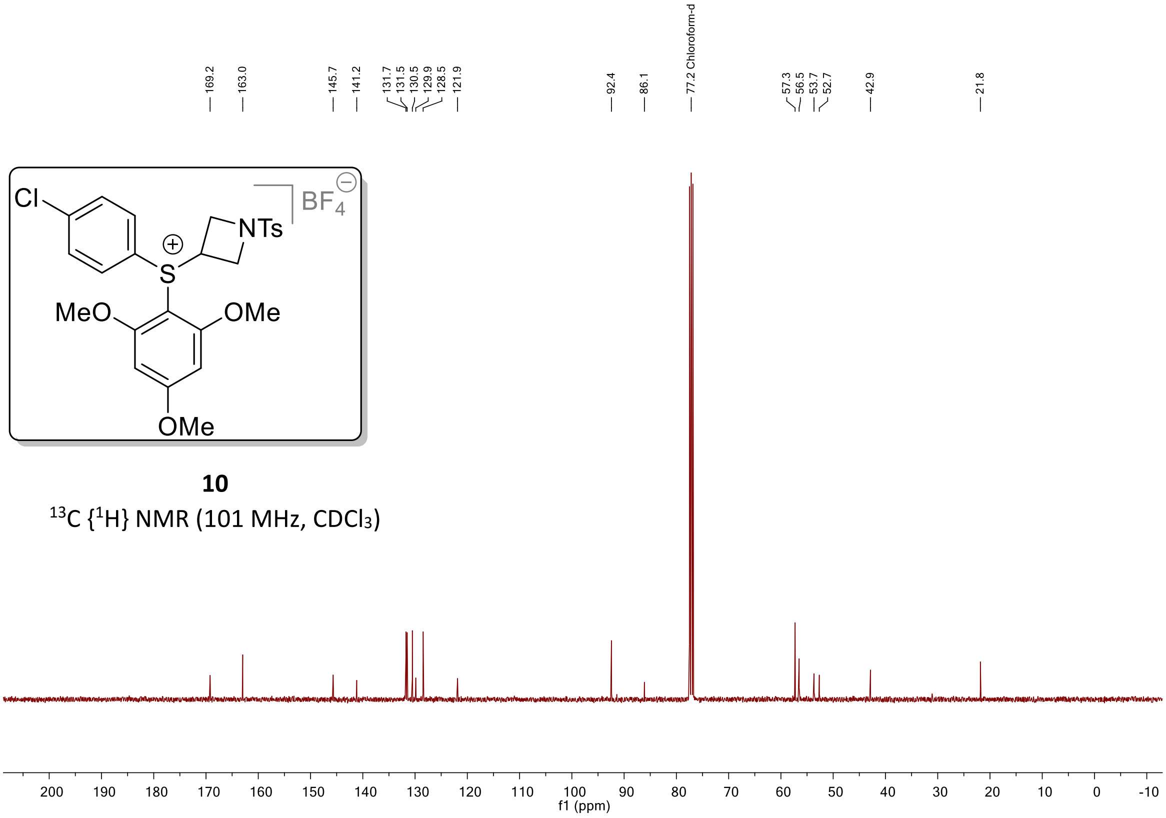

10

^{13}C {^{1}H} NMR (101 MHz, $CDCl_3$)

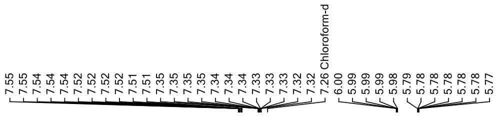

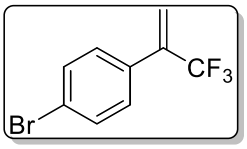

B

^{1}H NMR (400 MHz, $CDCl_3$)

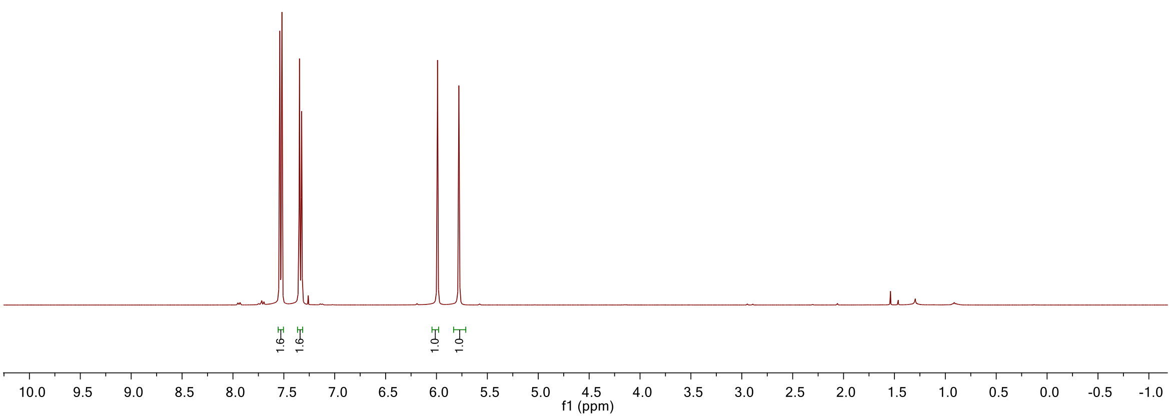

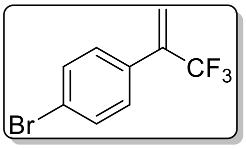

B

^{19}F NMR (377 MHz, $CDCl_3$)

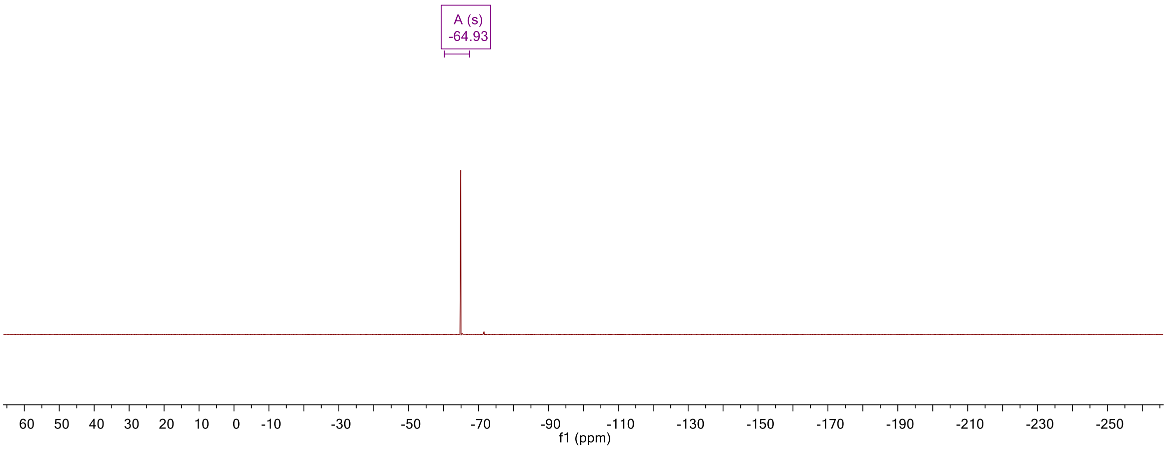

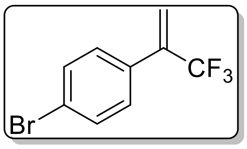

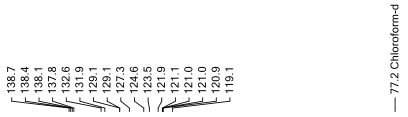

B

$^{13}C\{^{1}H\}$ NMR (101 MHz, $CDCl_3$)

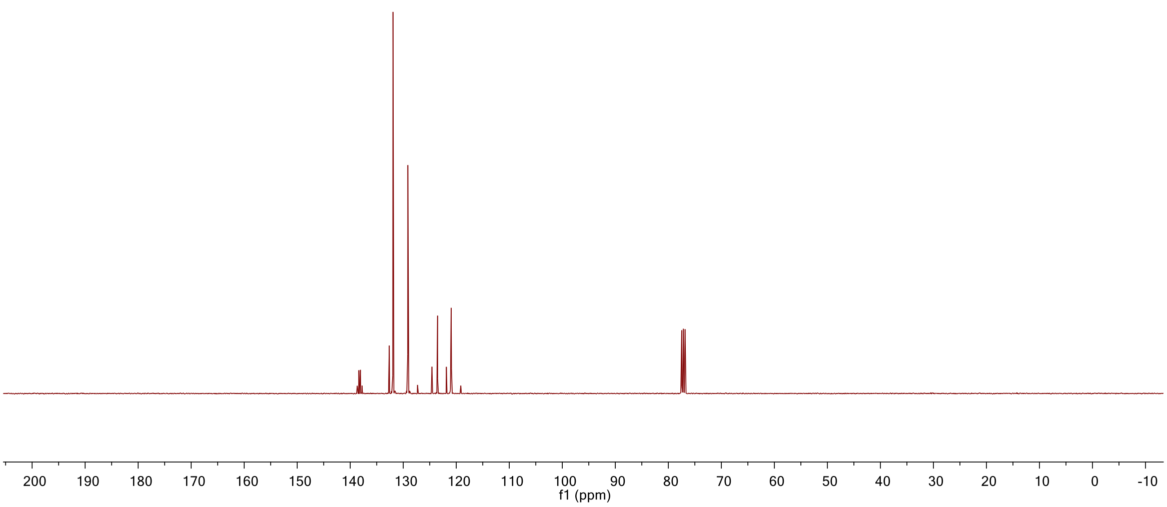

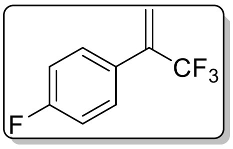

C

^{1}H NMR (400 MHz, $CDCl_3$)

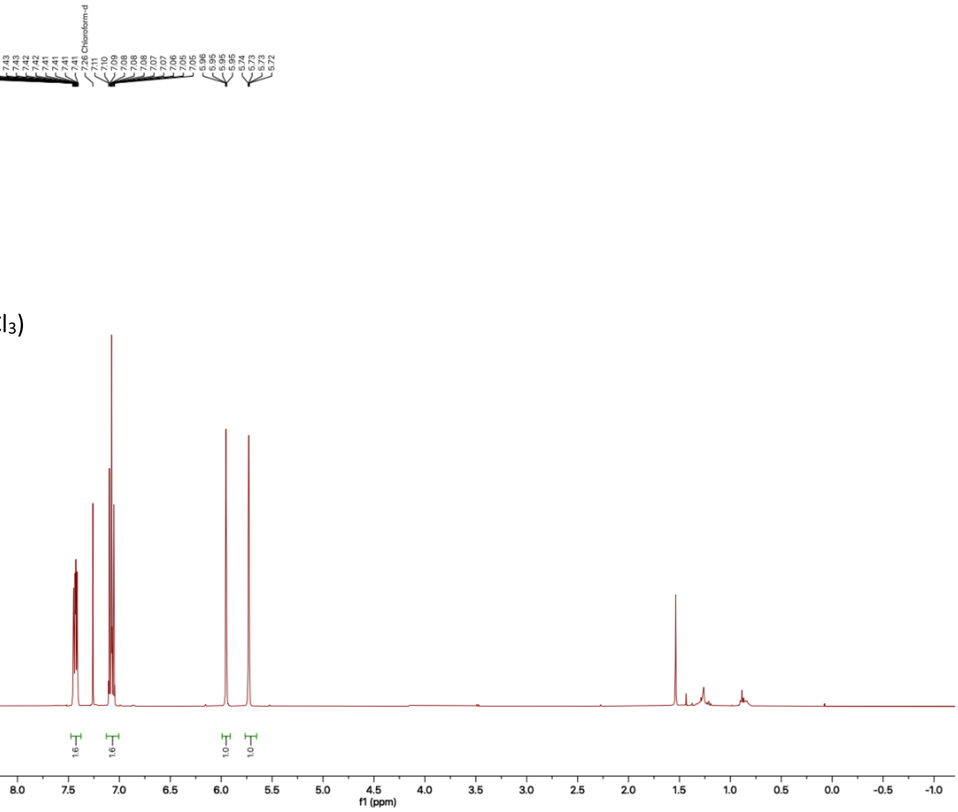

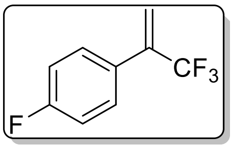

C

^{19}F NMR (377 MHz, $CDCl_3$)

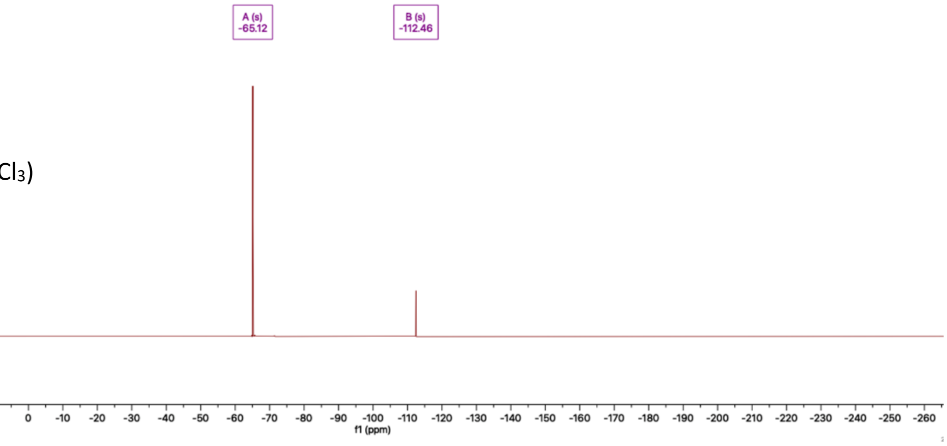

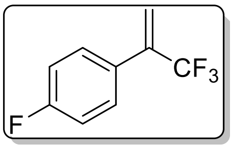

C

$^{13}C\{^{1}H\}$NMR (101 MHz, $CDCl_3$)

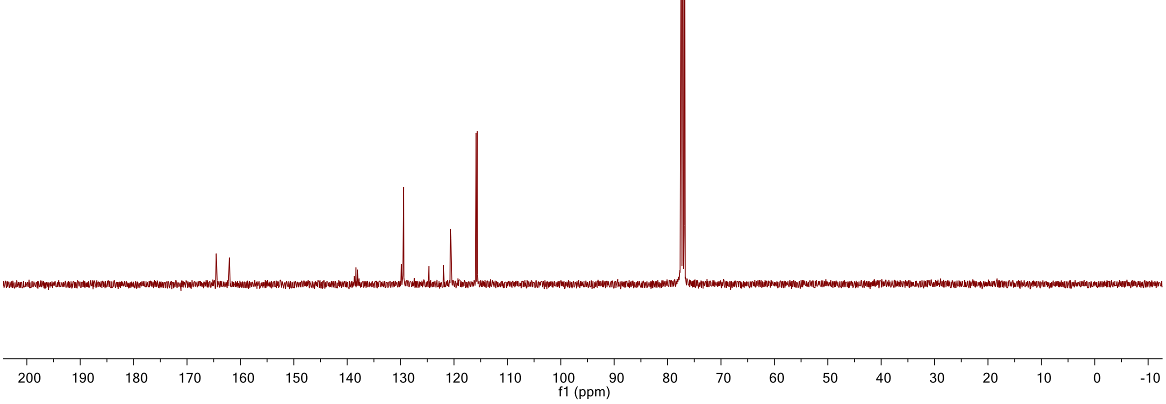

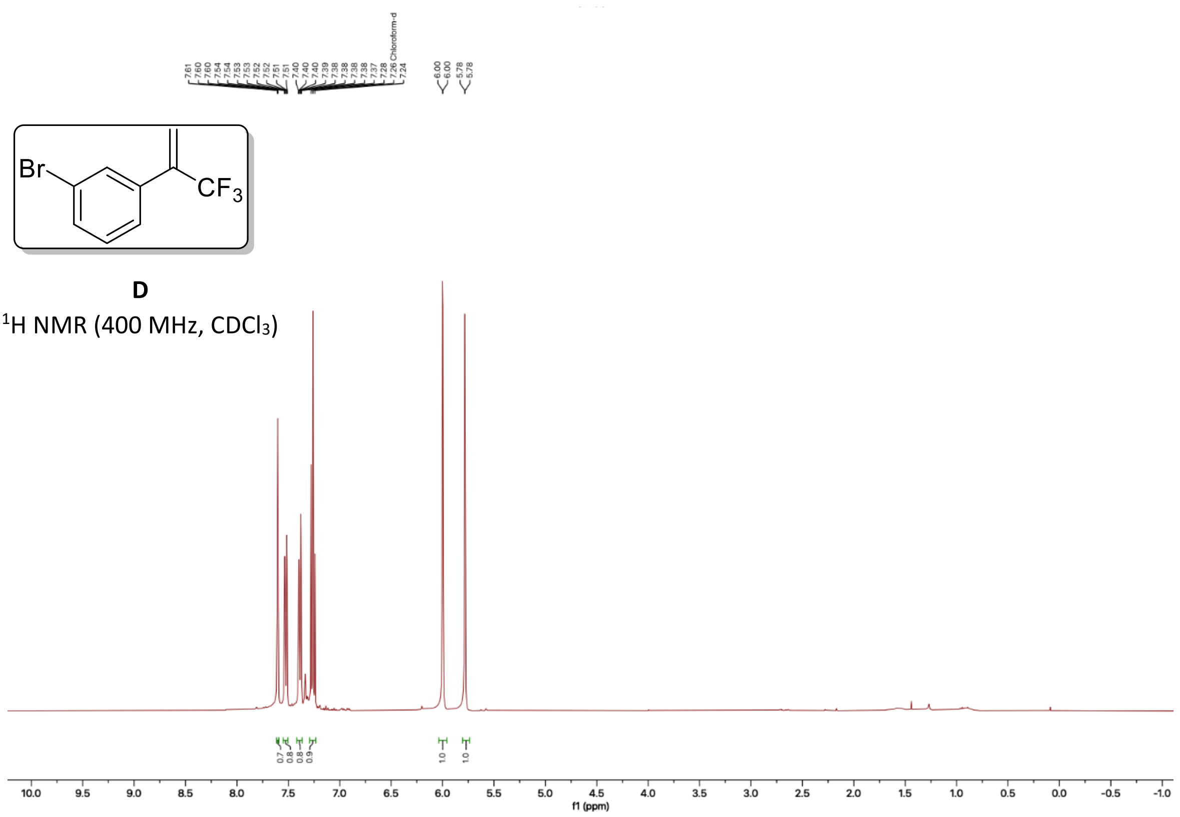

D

^{1}H NMR (400 MHz, $CDCl_3$)

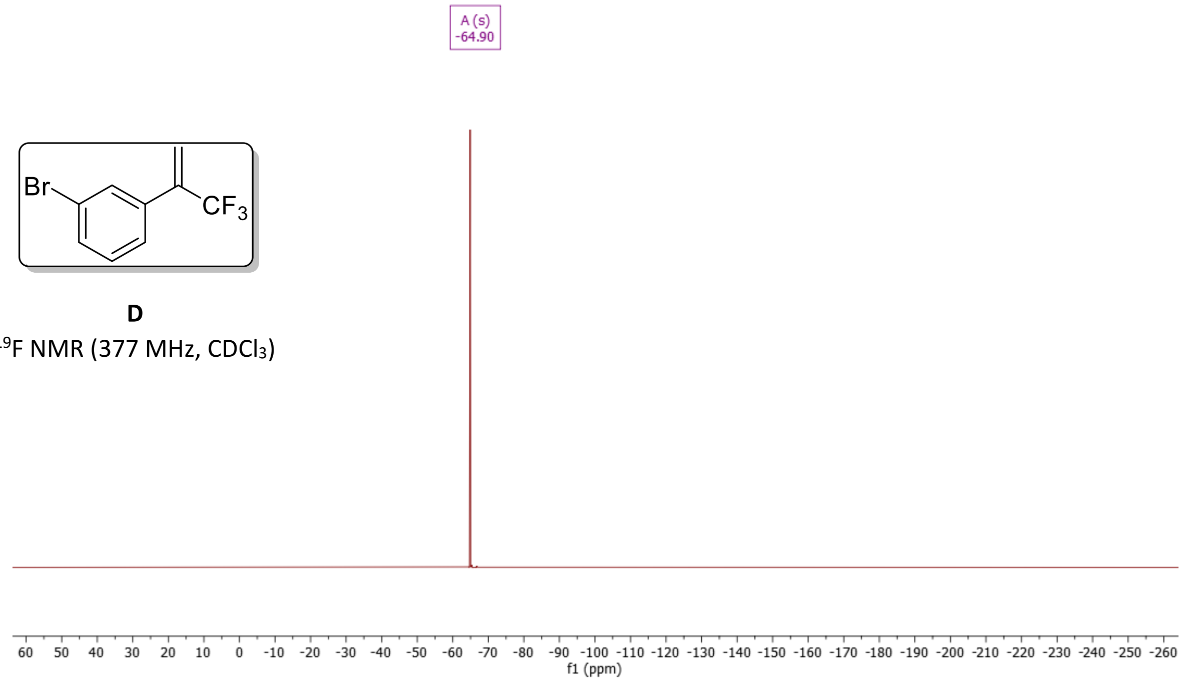

D

^{19}F NMR (377 MHz, $CDCl_3$)

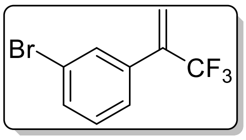

D

$^{13}C\{^{1}H\}$ NMR (101 MHz, $CDCl_3$)

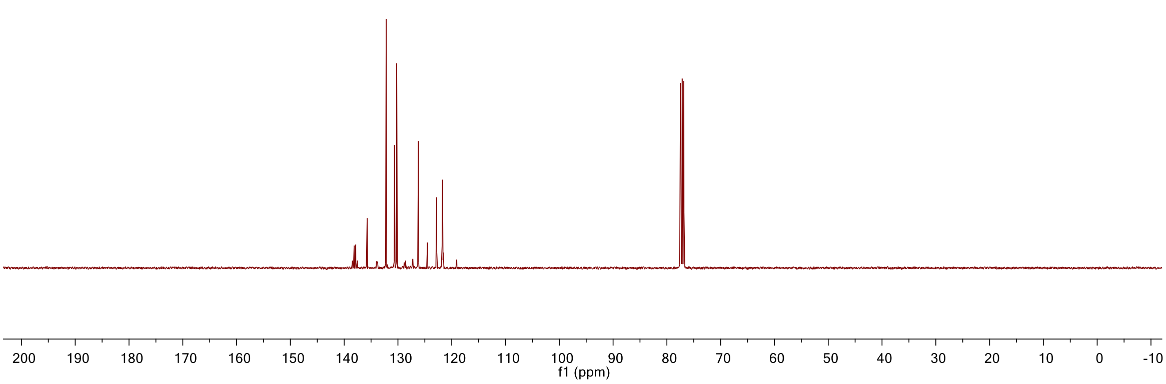

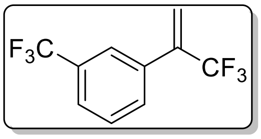

E

^{1}H NMR (400 MHz, $CDCl_3$)

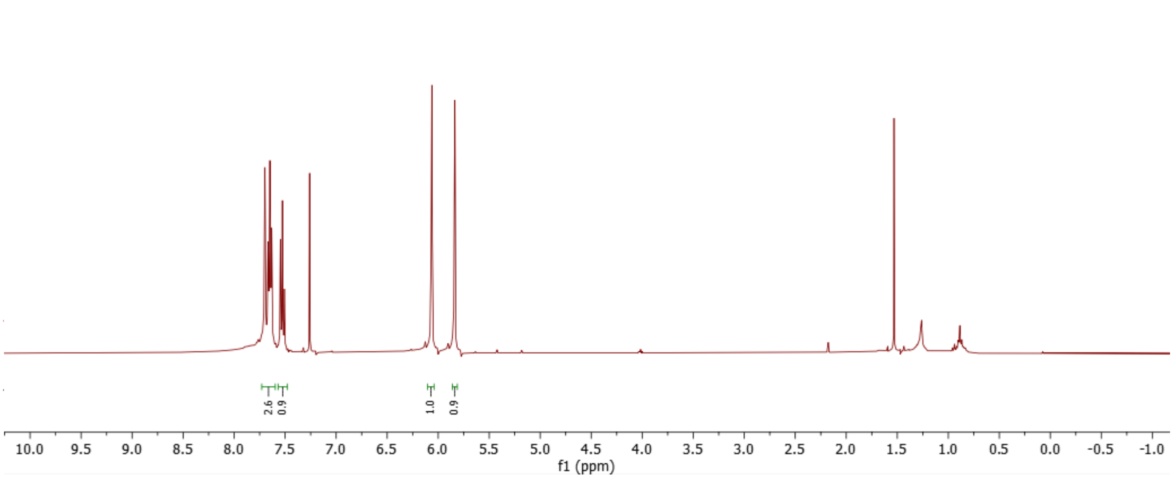

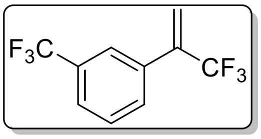

E

^{19}F NMR (377 MHz, $CDCl_3$)

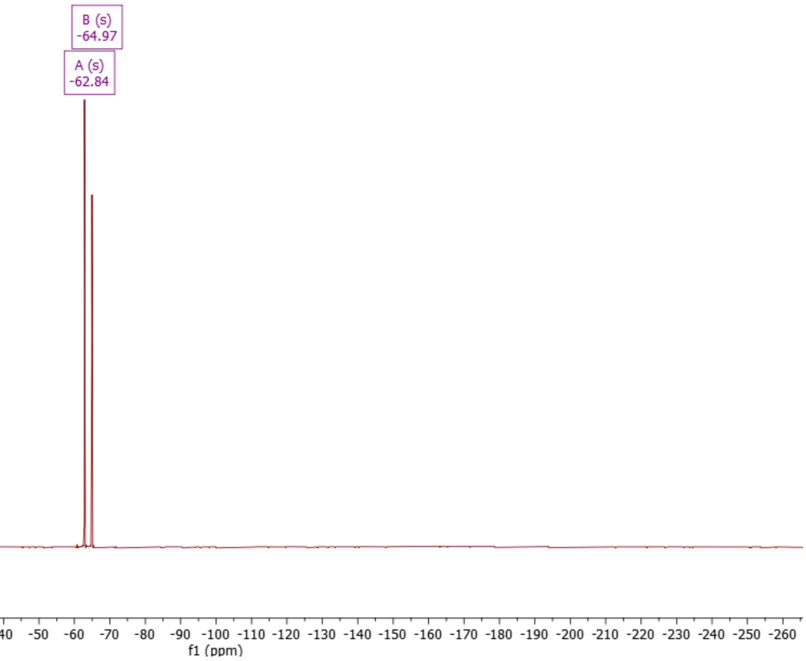

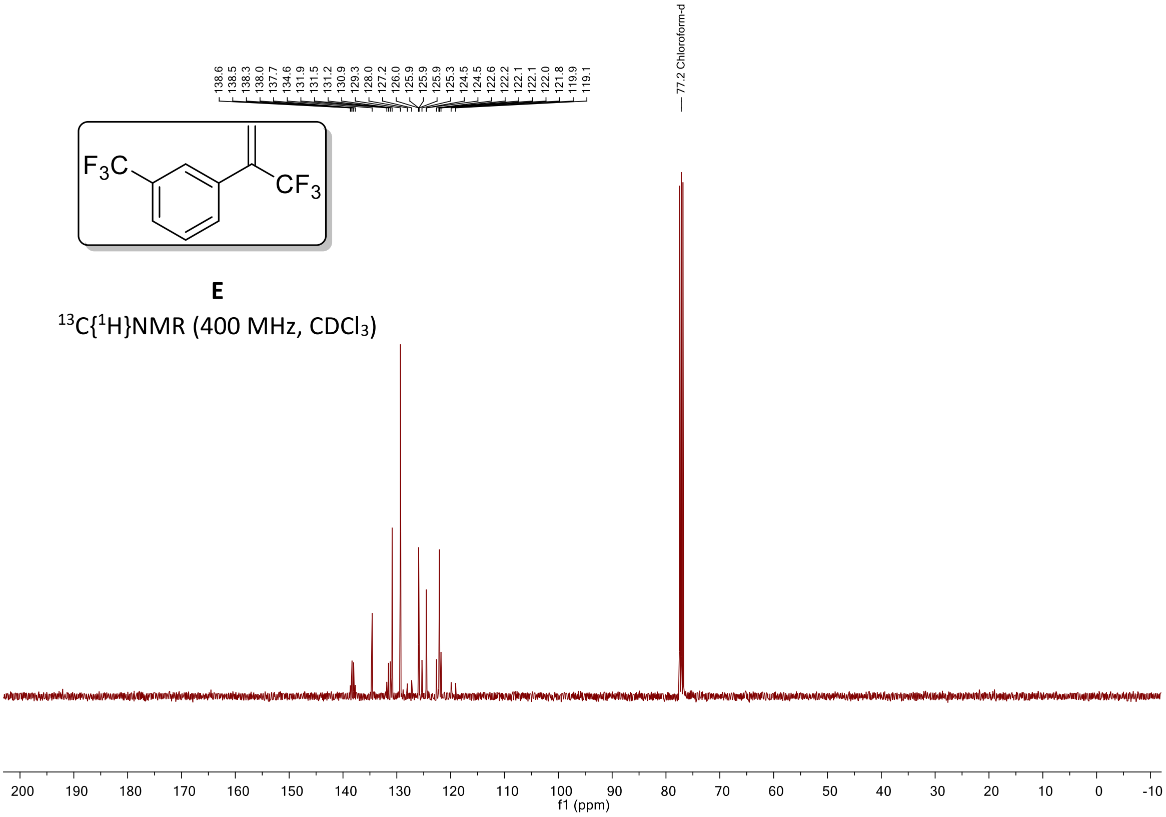

E

$^{13}C\{^1H\}$NMR (400 MHz, $CDCl_3$)

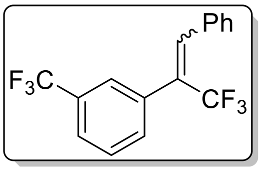

F

^{1}H NMR (400 MHz, $CDCl_3$)

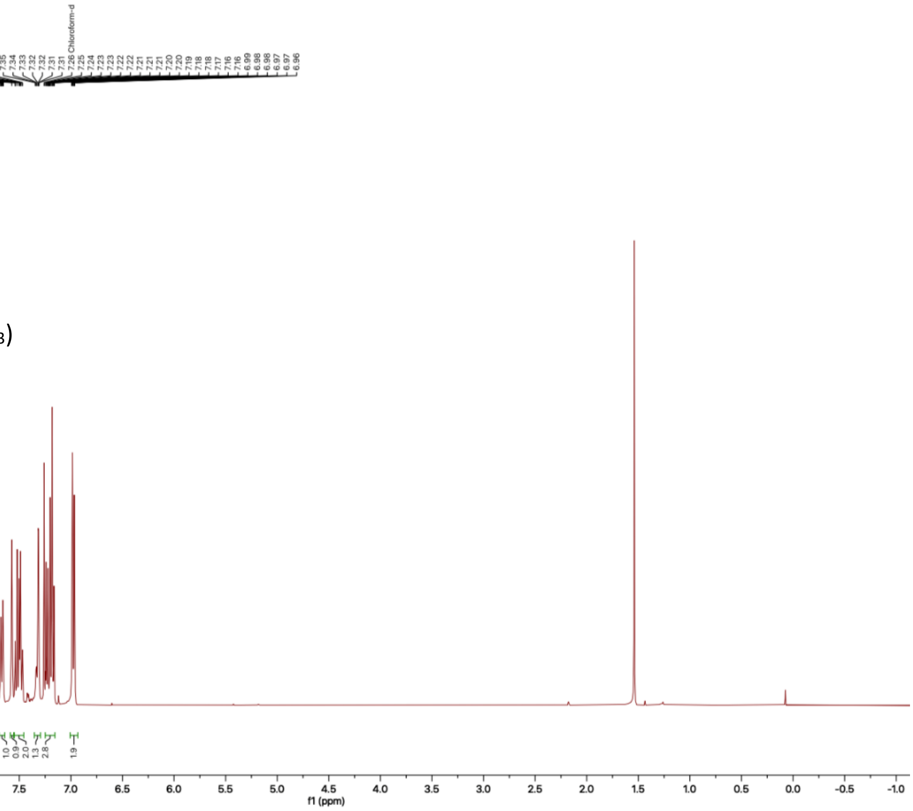

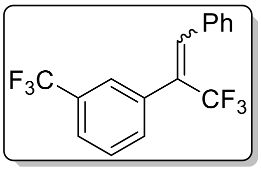

F

^{19}F NMR (377 MHz, $CDCl_3$)

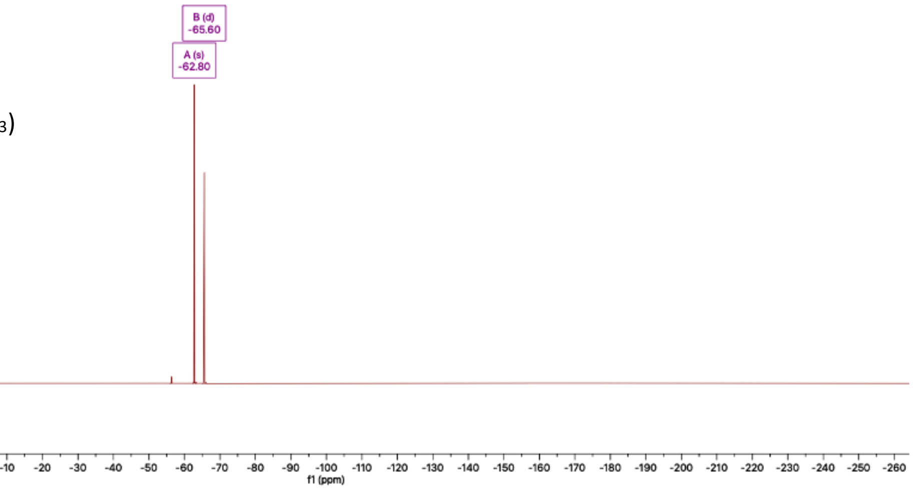

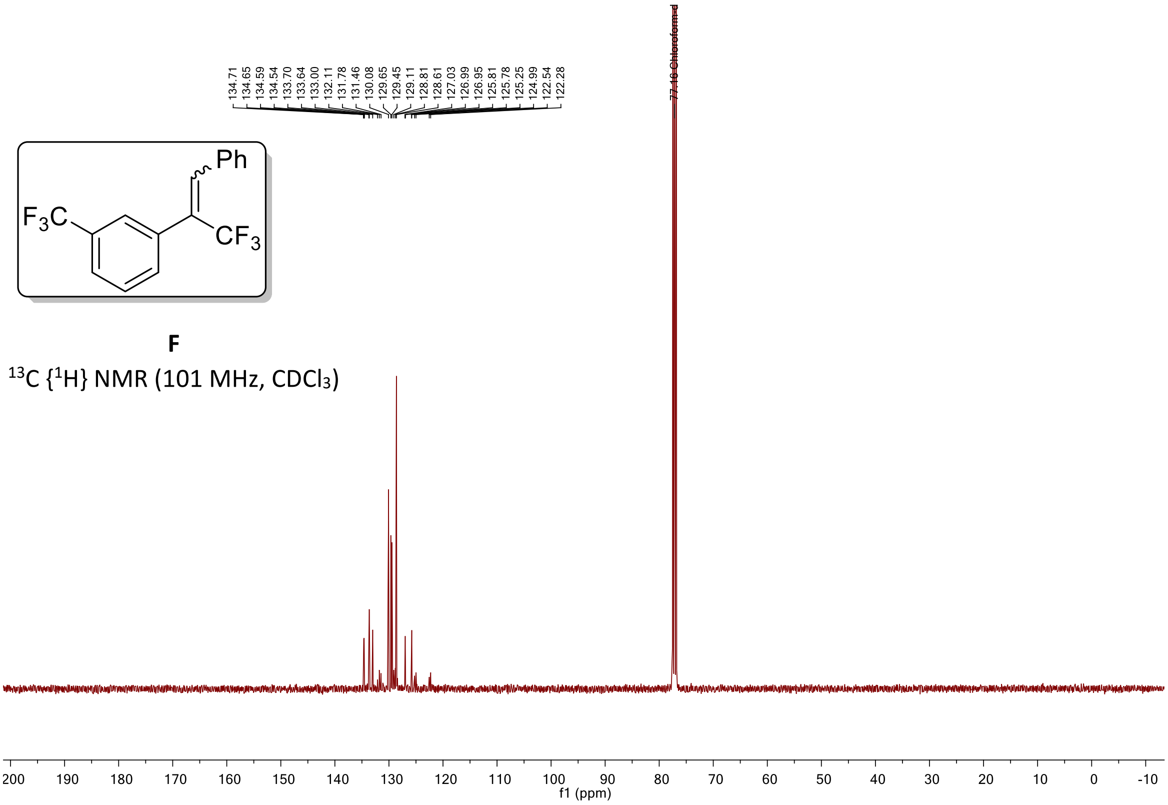

F

$^{13}C\ \{^1H\}$ NMR (101 MHz, $CDCl_3$)

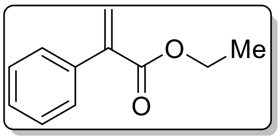

G

^{1}H NMR (400 MHz, $CDCl_3$)

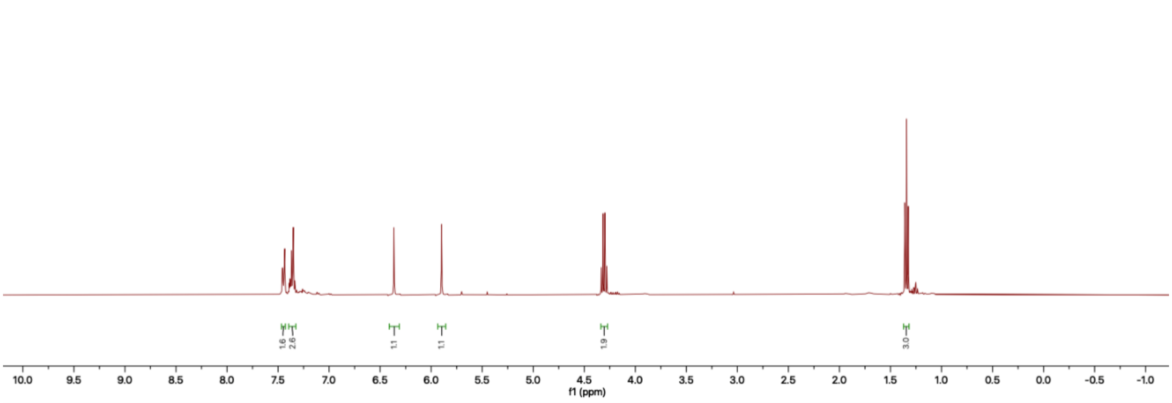

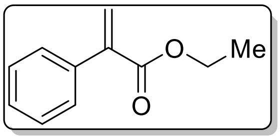

G

$^{13}C\ \{^{1}H\}$ NMR (101 MHz, $CDCl_3$)

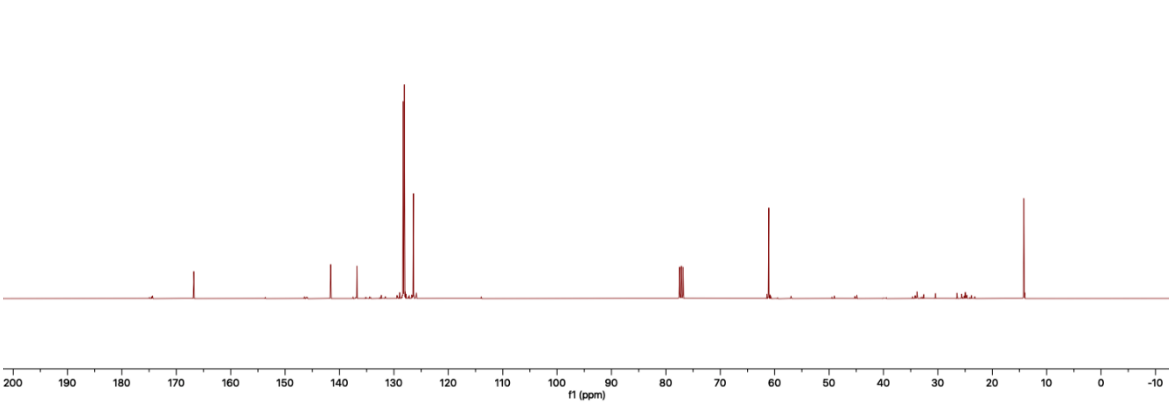

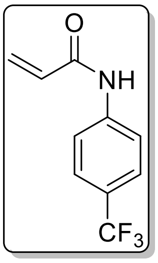

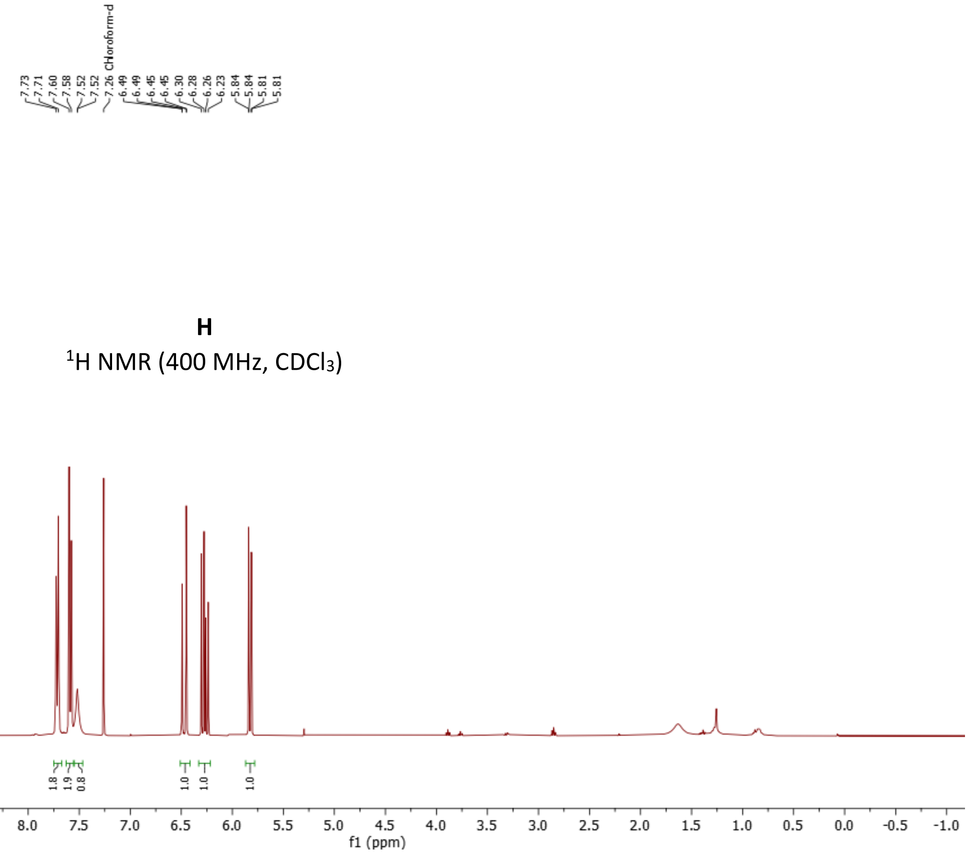

H

^{1}H NMR (400 MHz, $CDCl_3$)

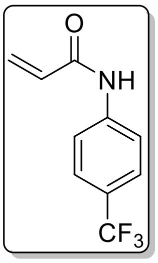

H

^{19}F NMR (377 MHz, $CDCl_3$)

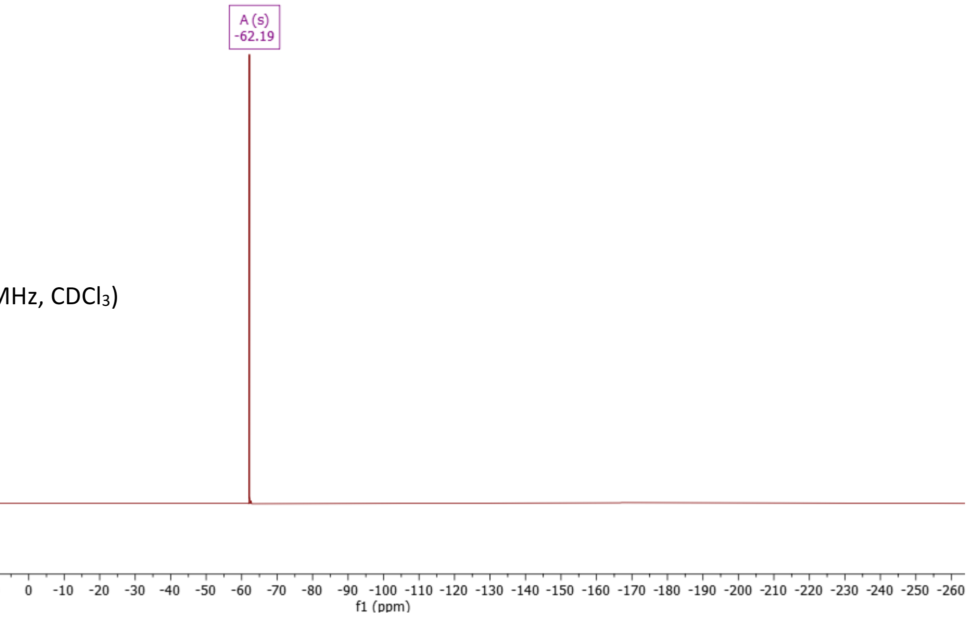

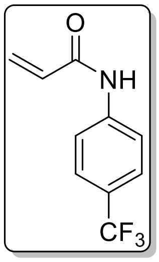

H

$^{13}C\{^{1}H\}$NMR (101 MHz, MeOD-d$_4$)

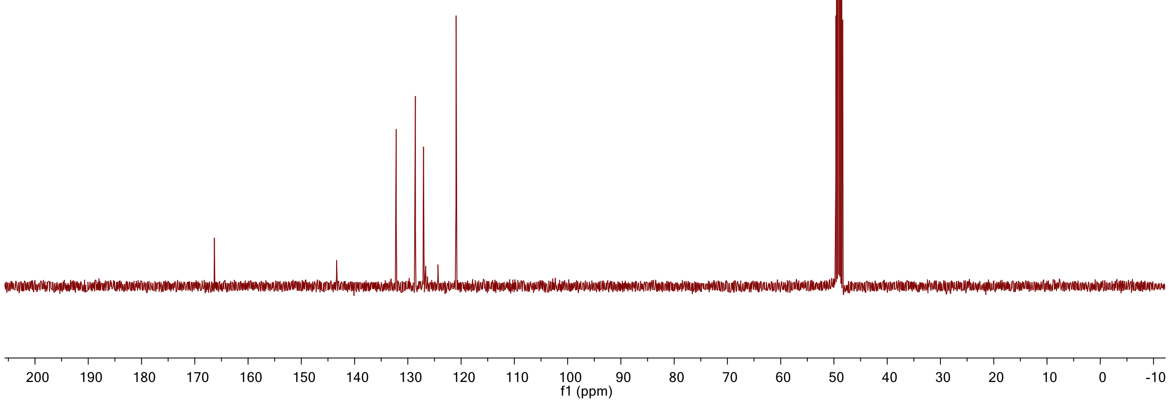

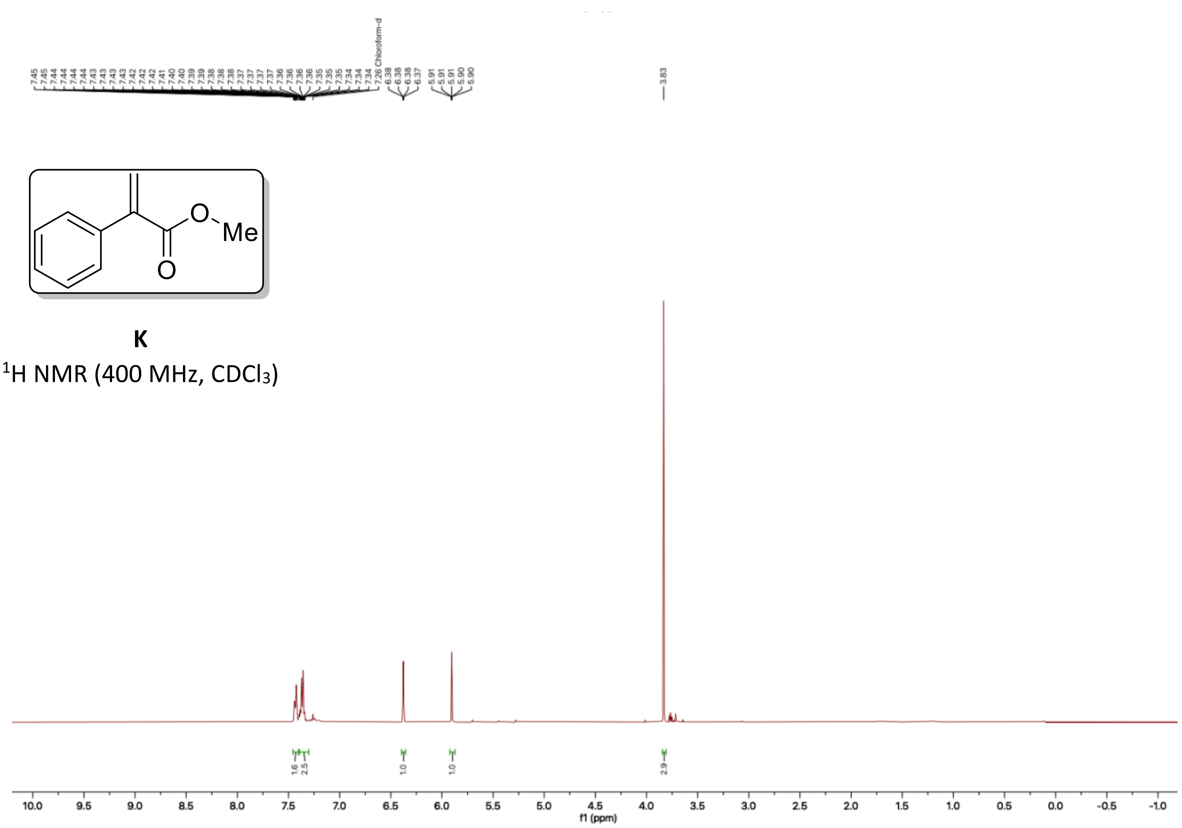

K

^{1}H NMR (400 MHz, $CDCl_3$)

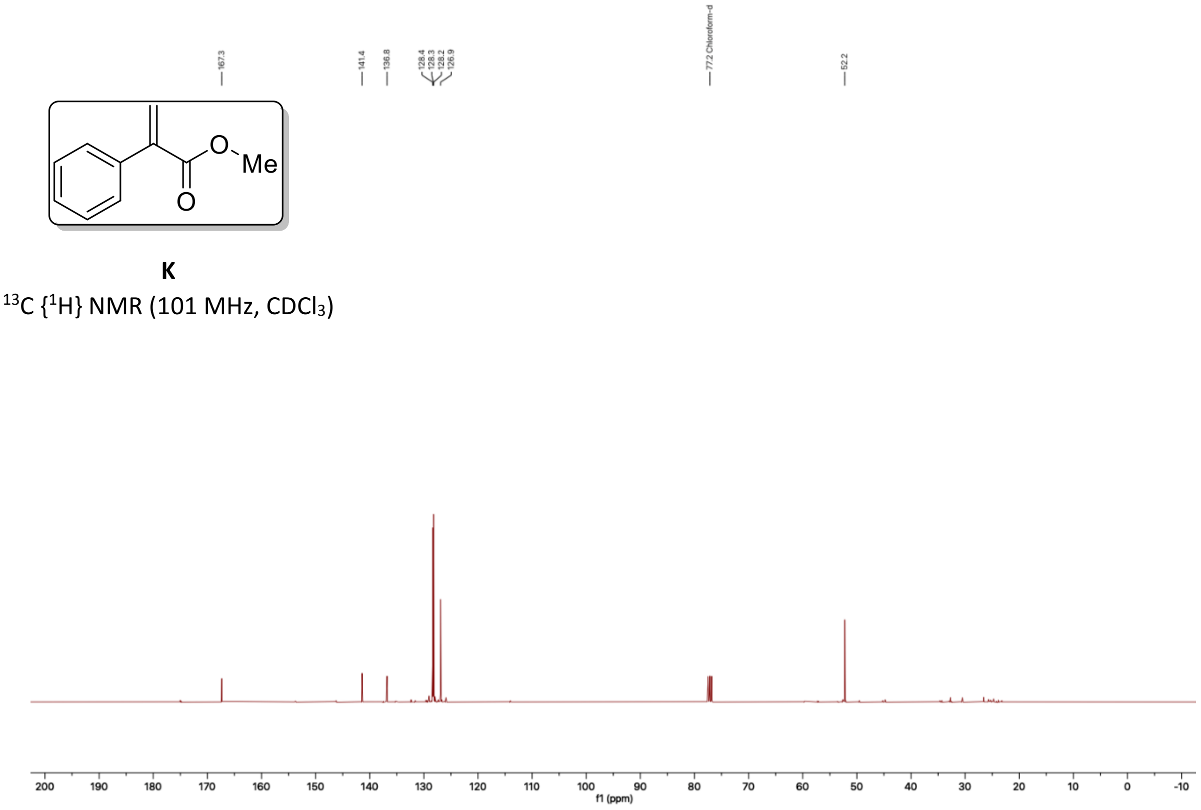

K

^{13}C {^{1}H} NMR (101 MHz, $CDCl_3$)

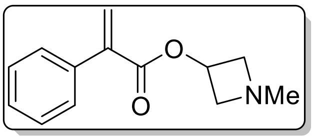

L

^{1}H NMR (400 MHz, $CDCl_3$)

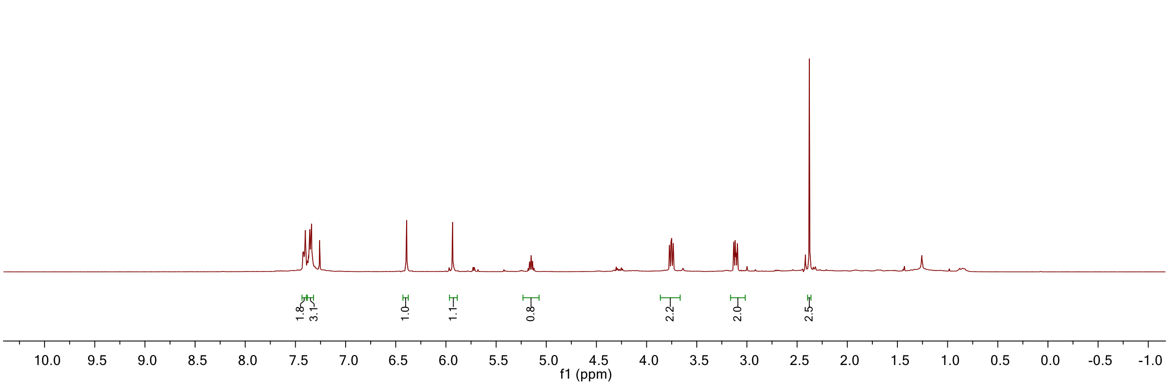

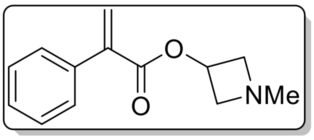

L

^{13}C {^{1}H} NMR (101 MHz, $CDCl_3$)

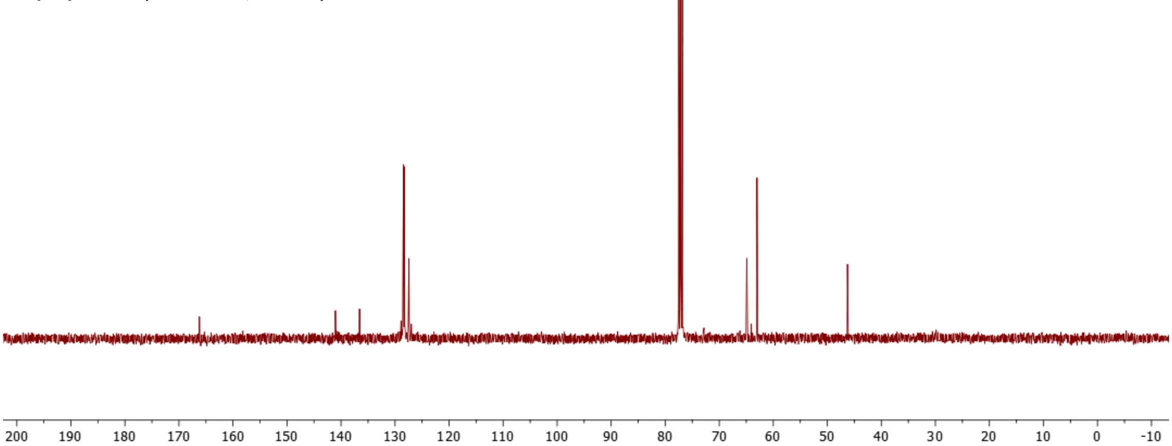

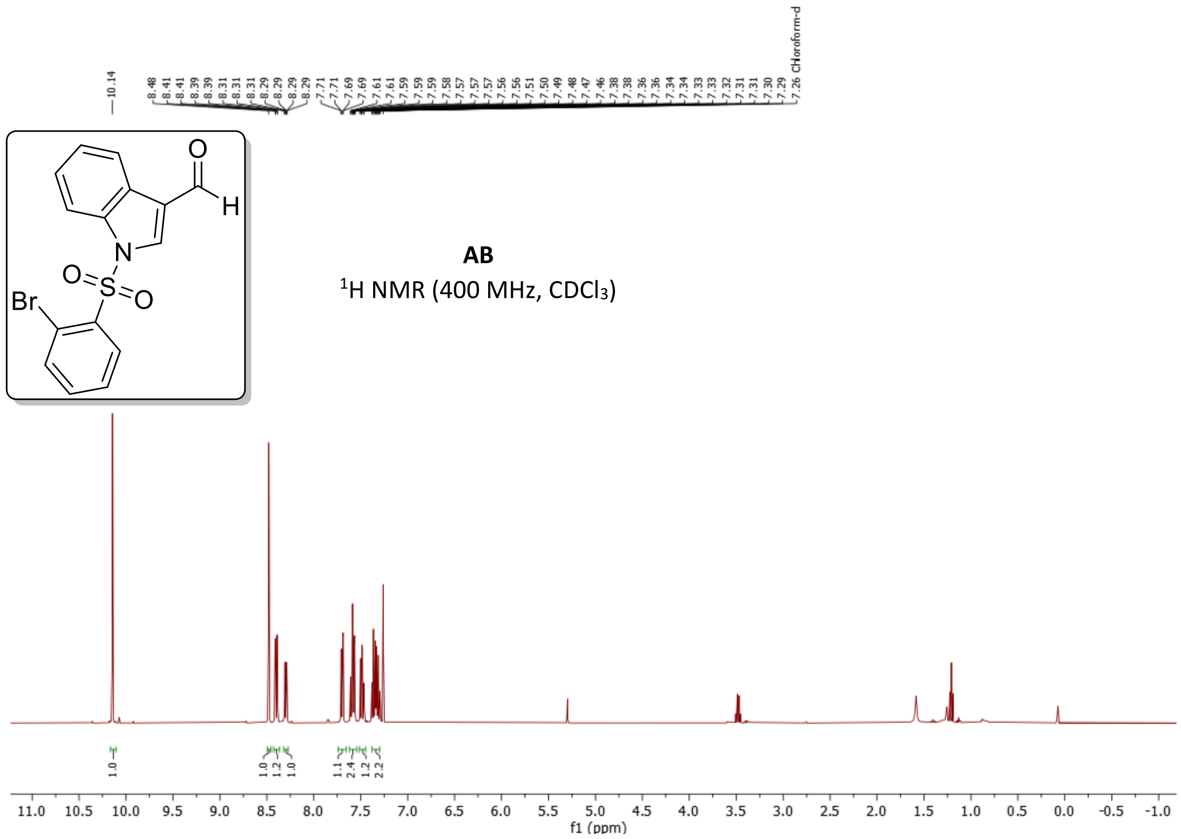

AB

^{1}H NMR (400 MHz, $CDCl_3$)

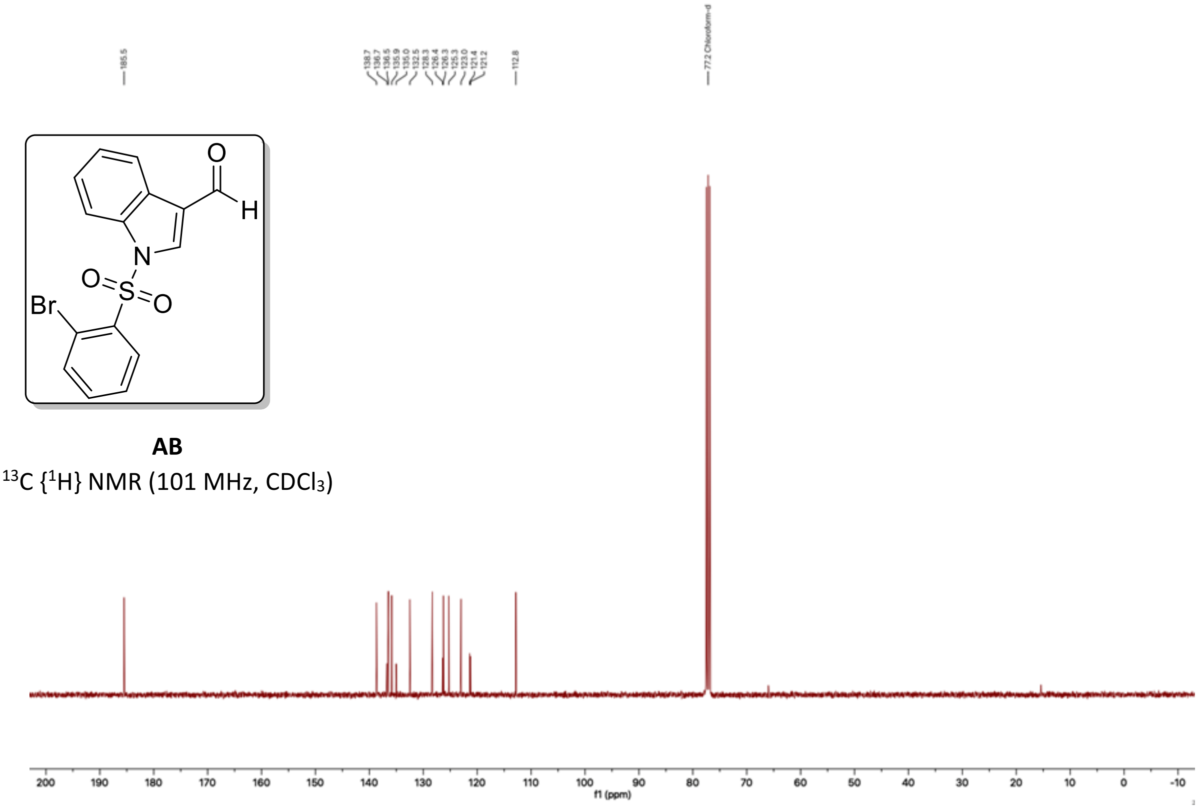

AB

^{13}C {^{1}H} NMR (101 MHz, $CDCl_3$)

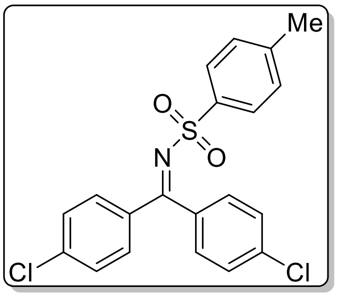

AE

^{1}H NMR (400 MHz, $CDCl_3$)

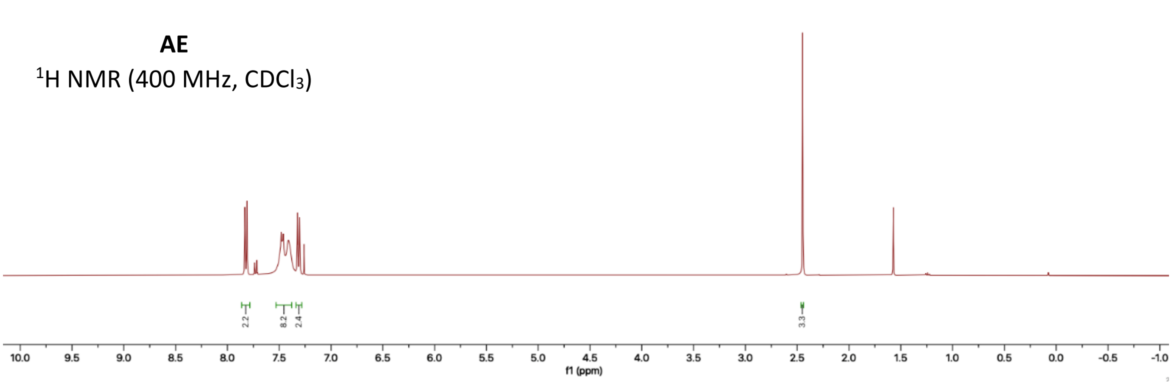

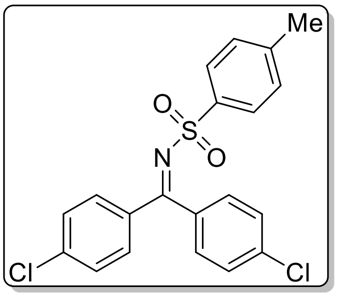

AE

^{13}C {^{1}H} NMR (101 MHz, $CDCl_3$)

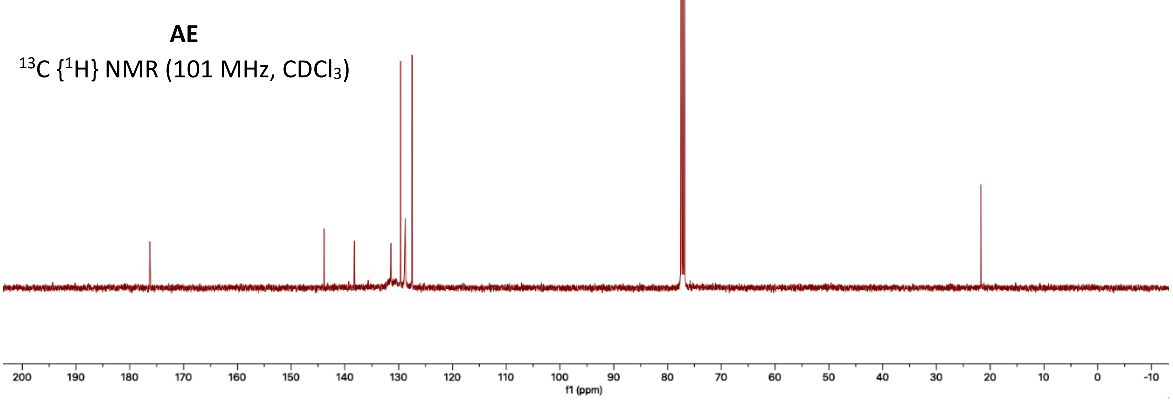

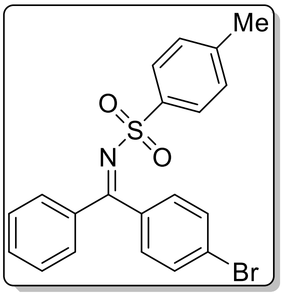

AF

^{1}H NMR (400 MHz, $CDCl_3$)

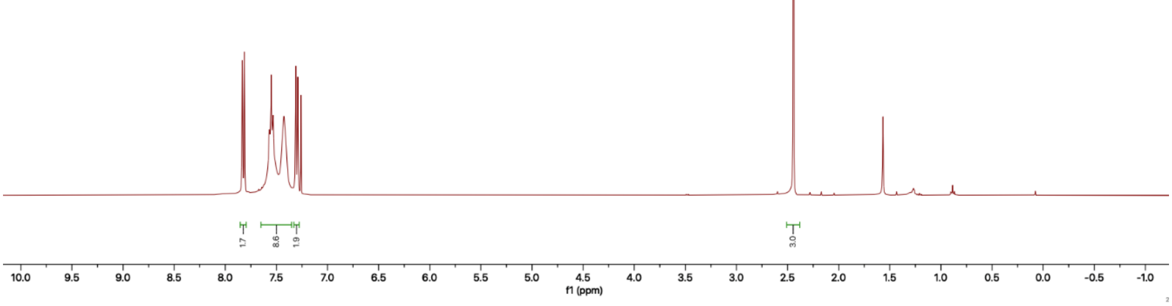

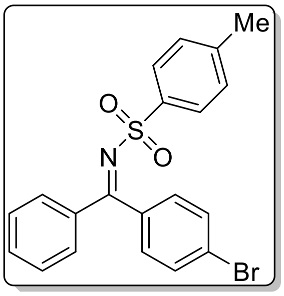

AF

^{13}C {^{1}H} NMR (101 MHz, $CDCl_3$)

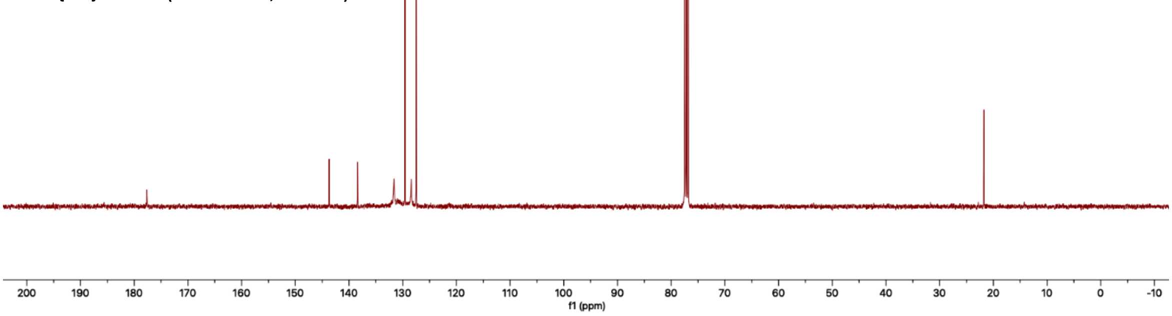

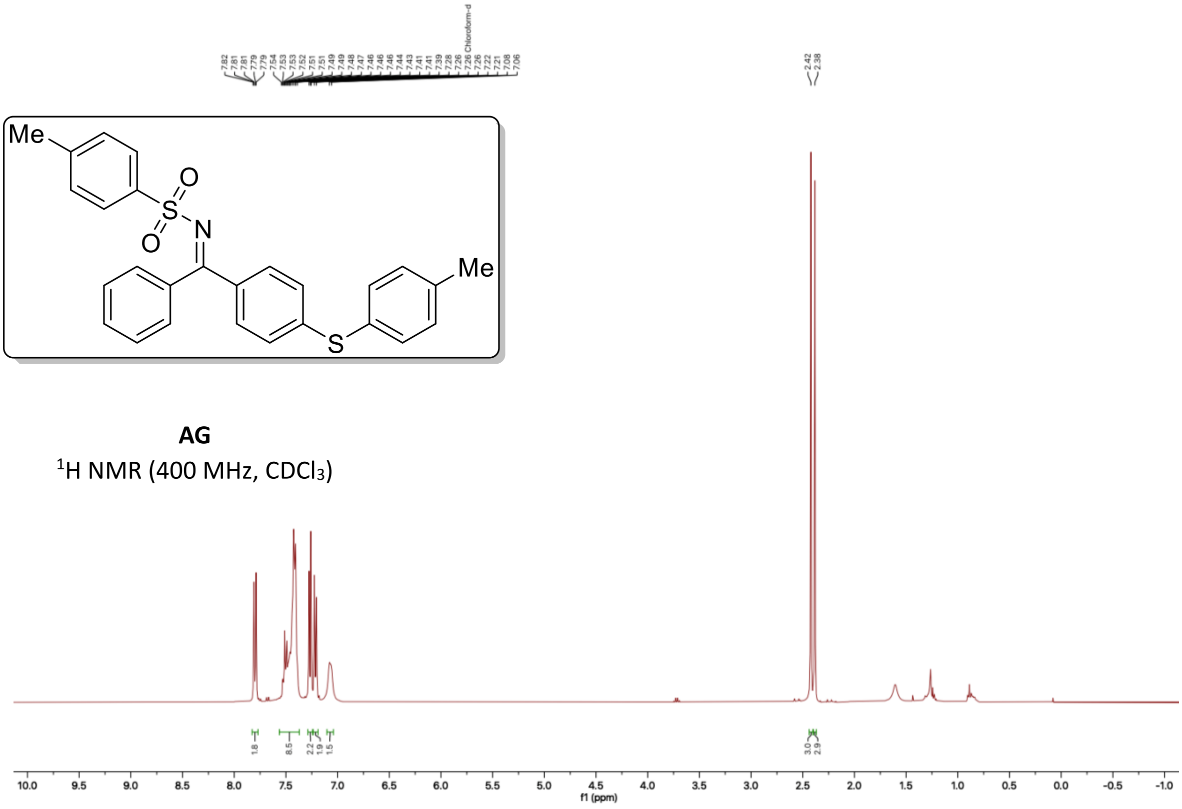

AG

^{1}H NMR (400 MHz, $CDCl_3$)

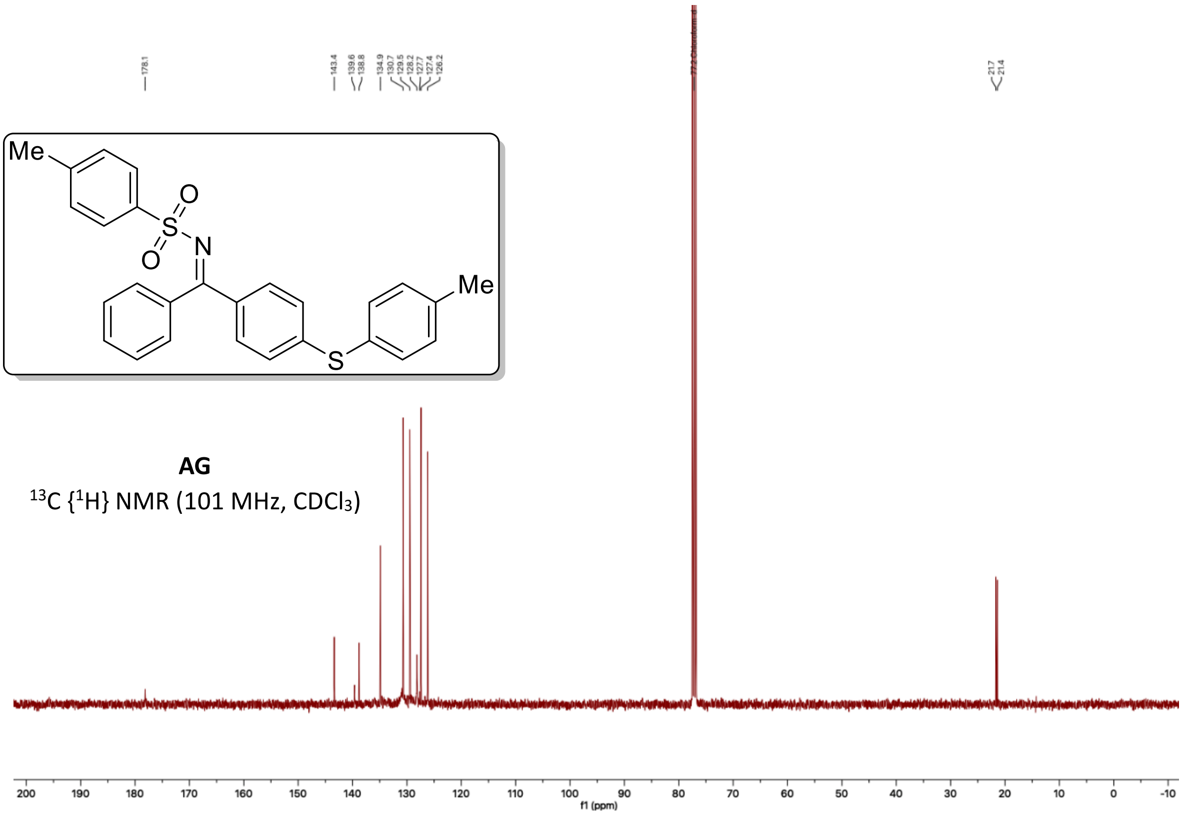

AG

^{13}C {^{1}H} NMR (101 MHz, $CDCl_3$)

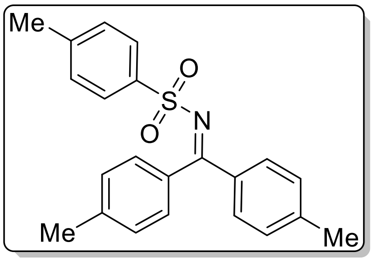

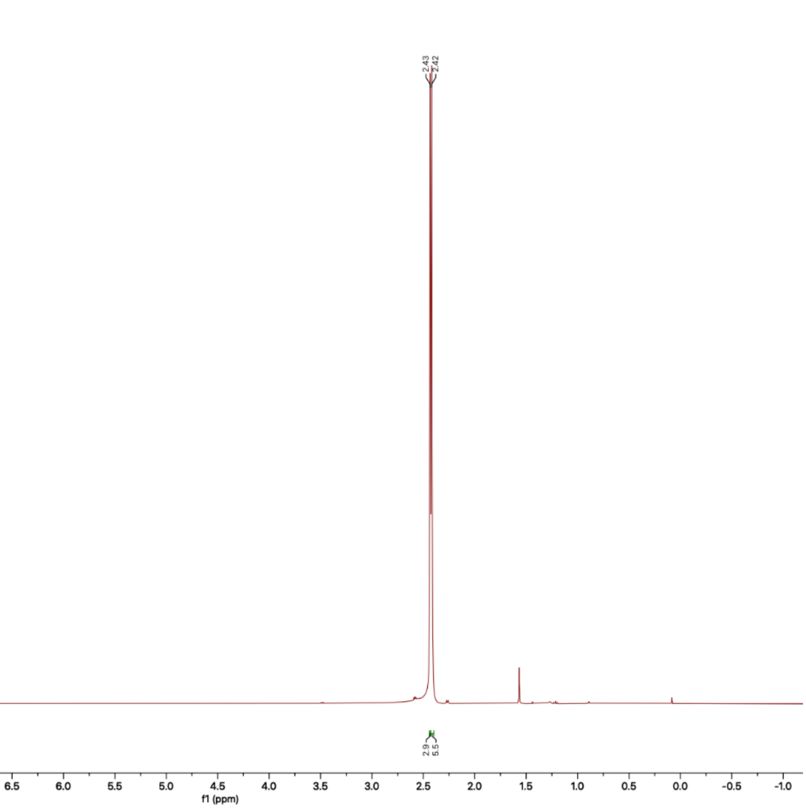

AH
^{1}H NMR (400 MHz, CDCl$_3$)

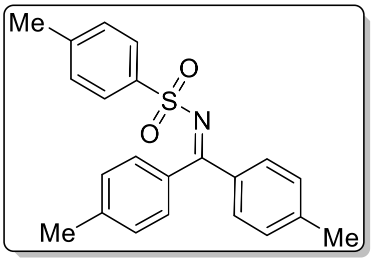

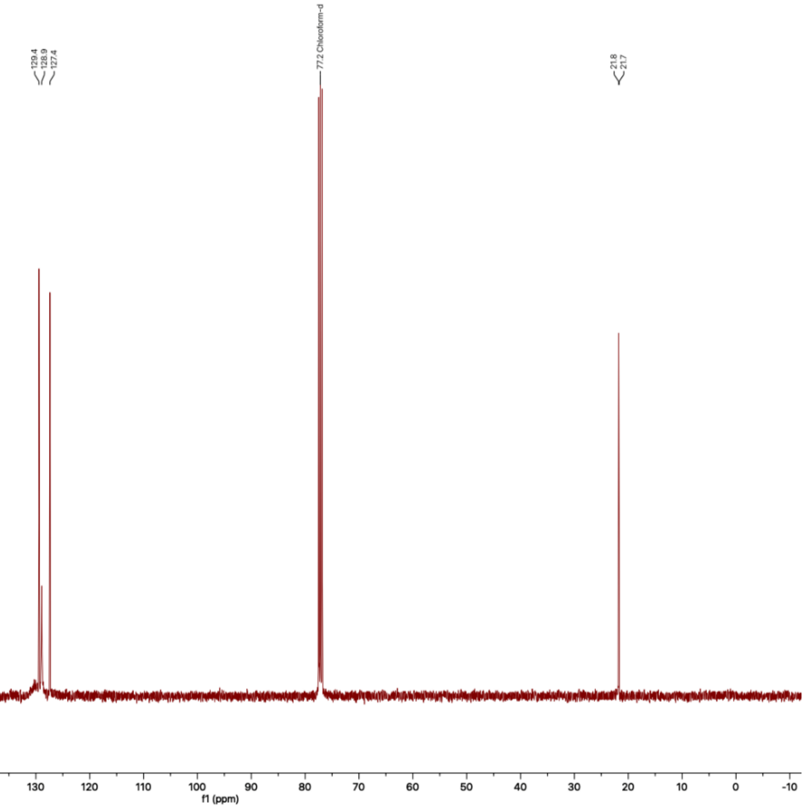

AH
^{13}C {^{1}H} NMR (101 MHz, CDCl$_3$)

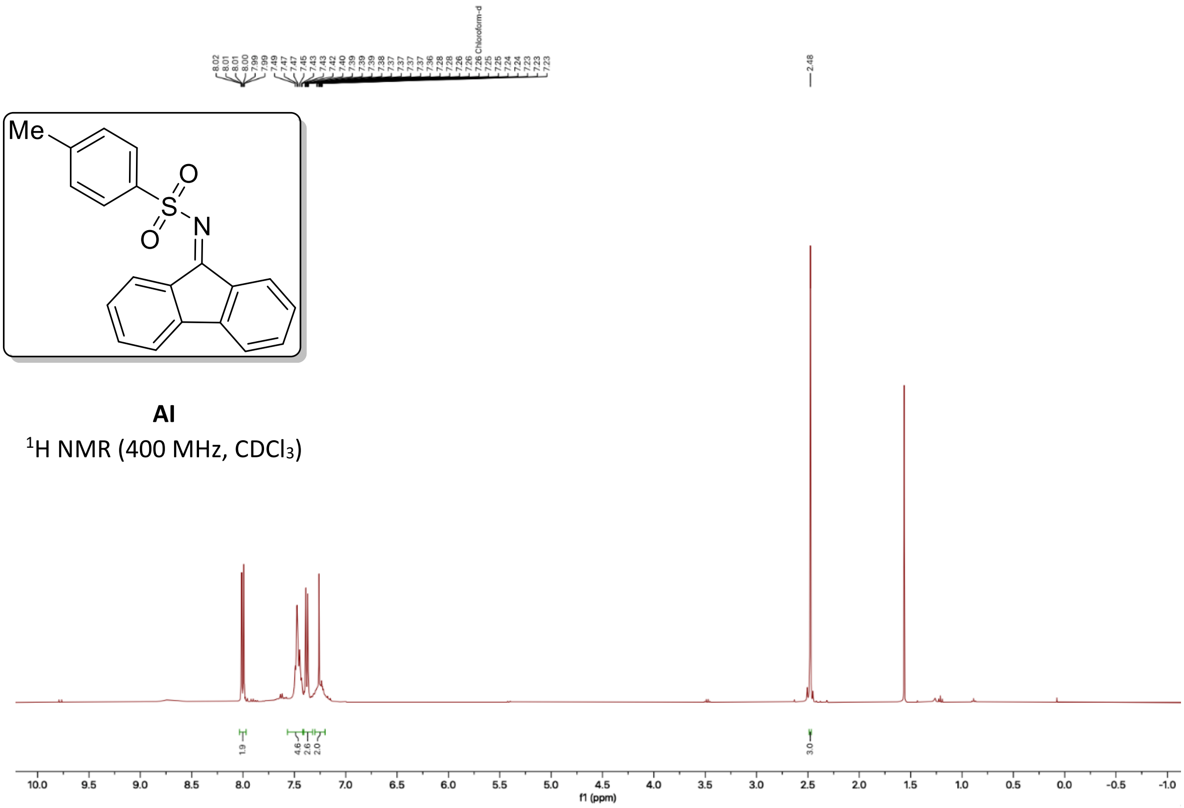

AI
^{1}H NMR (400 MHz, $CDCl_3$)

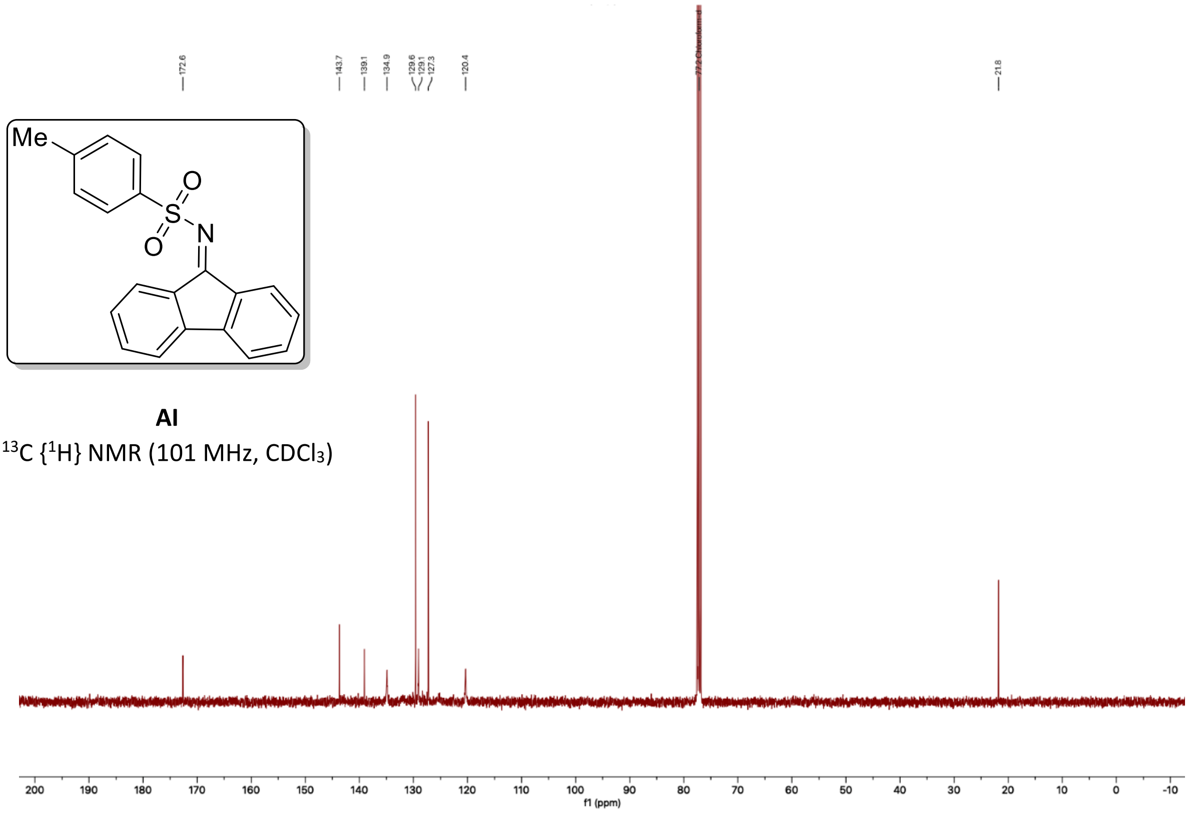

AI
^{13}C {^{1}H} NMR (101 MHz, $CDCl_3$)

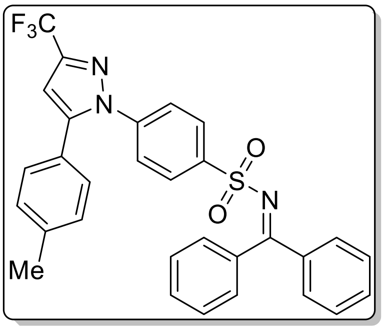

AJ

^{1}H NMR (400 MHz, $CDCl_3$)

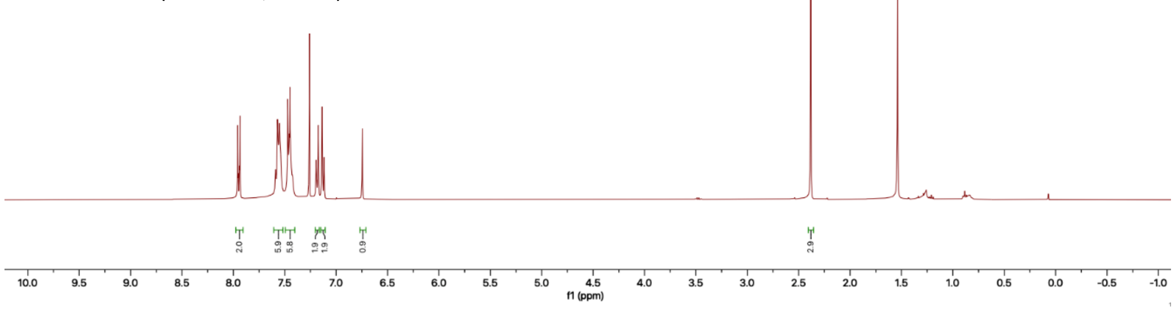

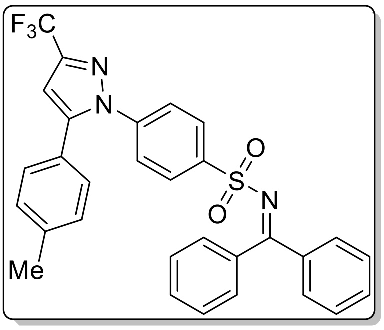

AJ

^{19}F NMR (377 MHz, $CDCl_3$)

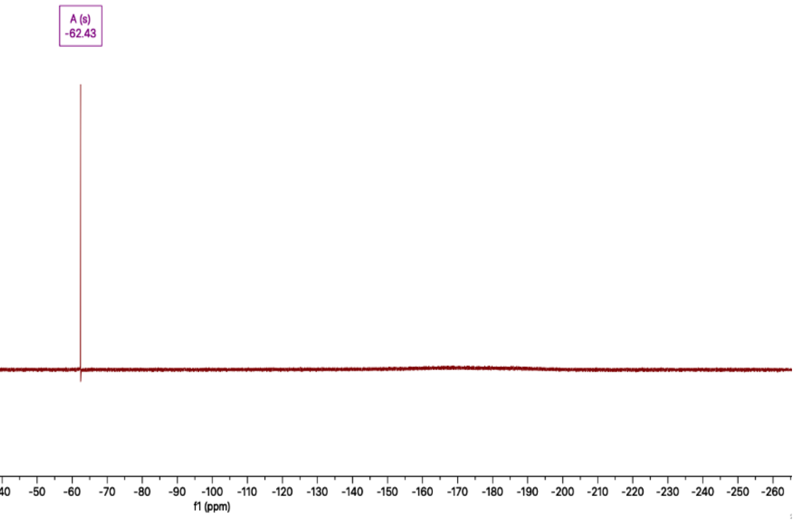

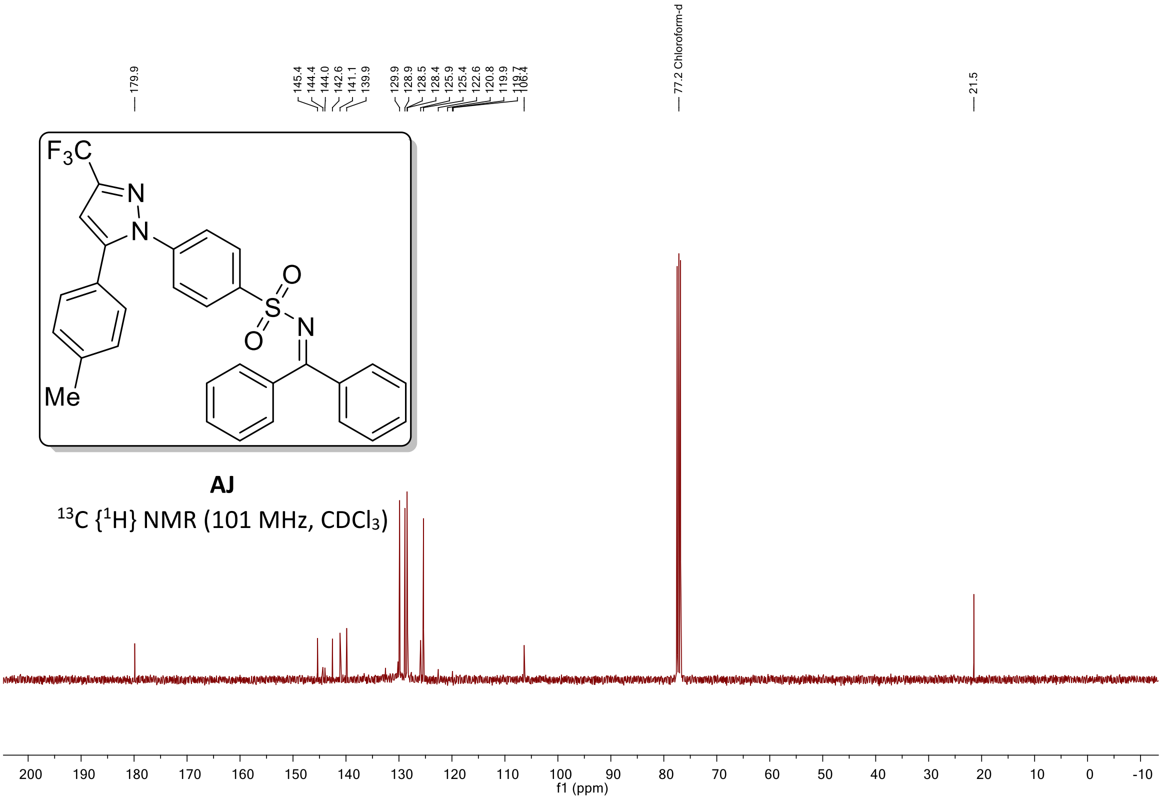

AJ

$^{13}C\ \{^1H\}$ NMR (101 MHz, $CDCl_3$)

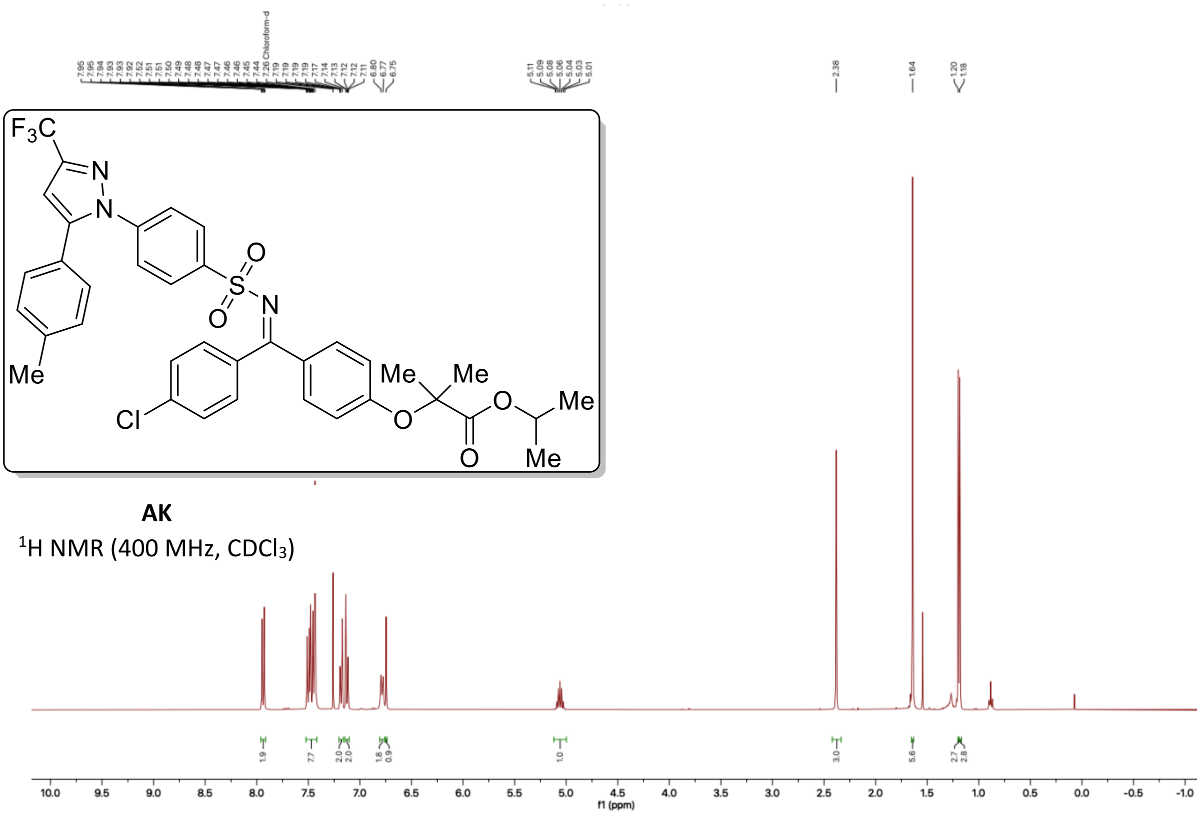

AK

^{1}H NMR (400 MHz, $CDCl_3$)

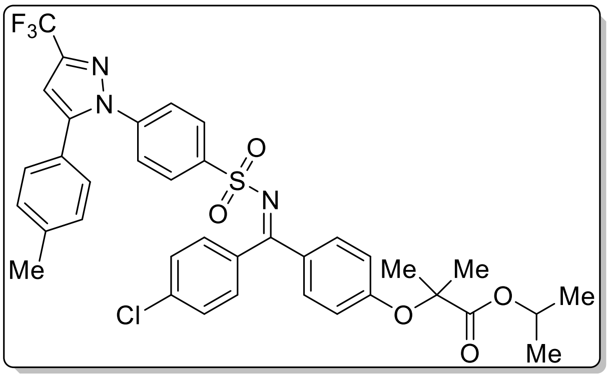

AK

^{19}F NMR (377 MHz, $CDCl_3$)

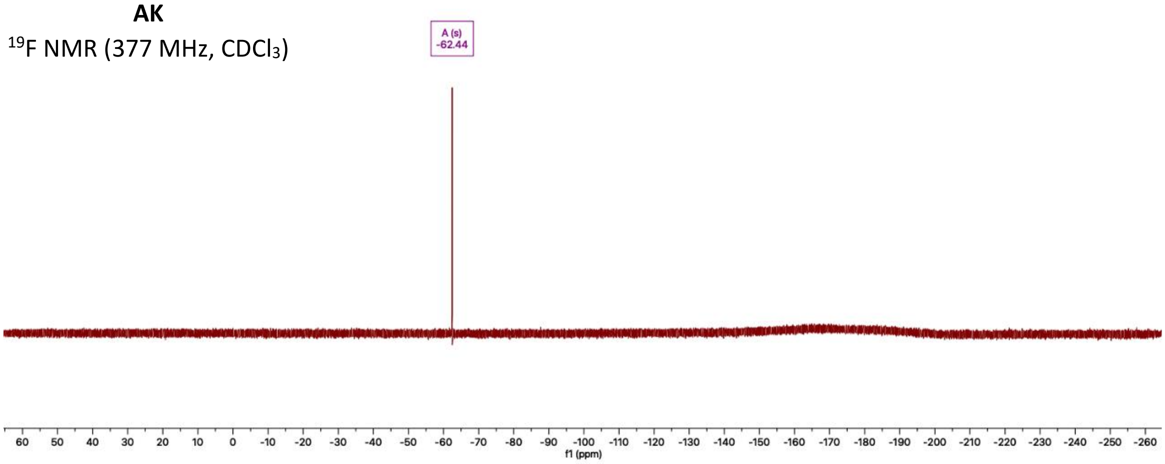

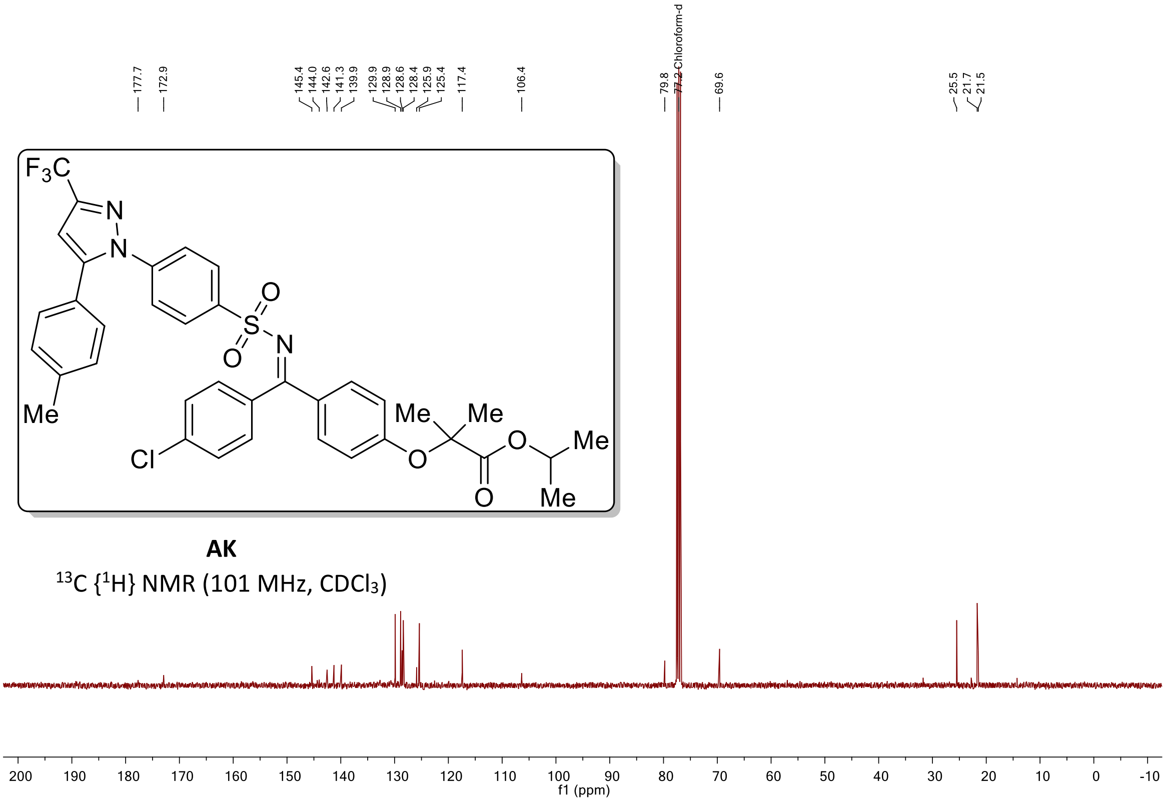

AK

$^{13}C\ \{^{1}H\}$ NMR (101 MHz, $CDCl_3$)

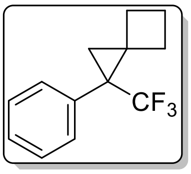

11

^{1}H NMR (400 MHz, CDCl$_3$)

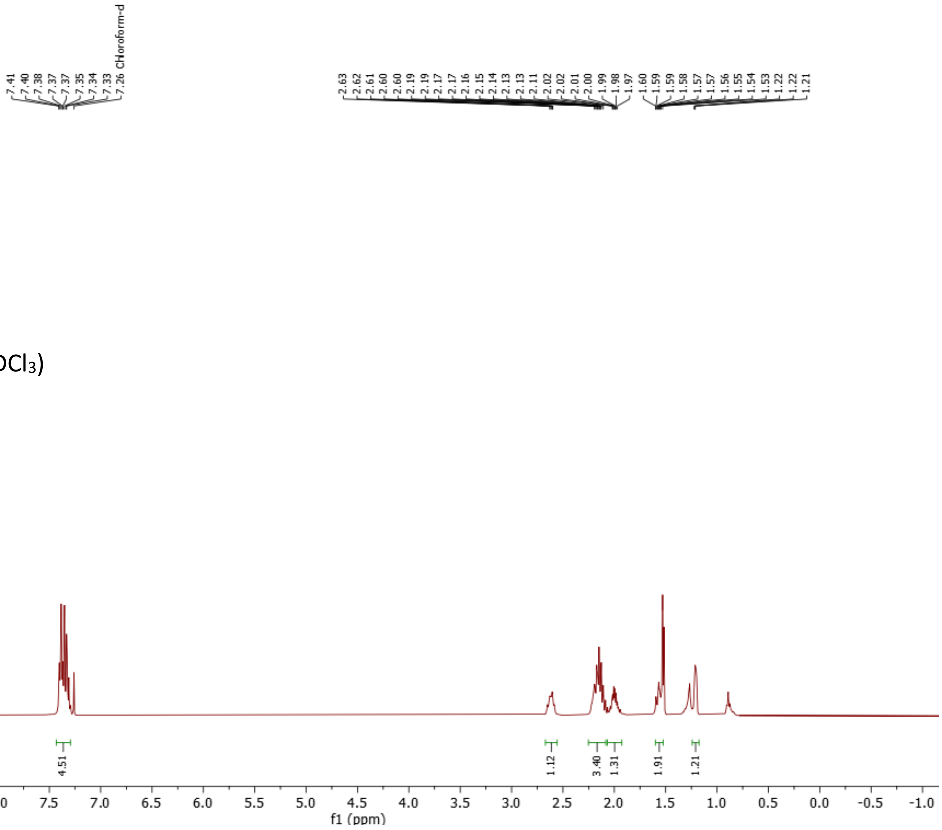

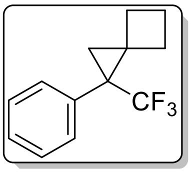

11

^{19}F NMR (377 MHz, CDCl$_3$)

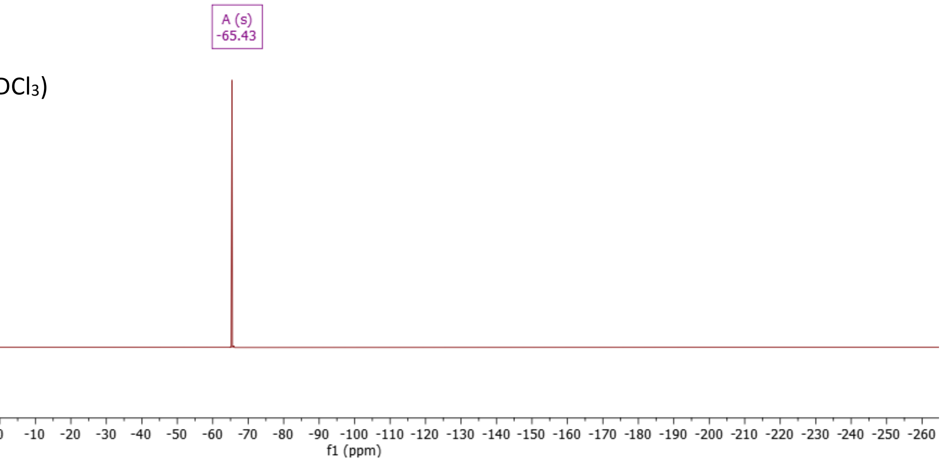

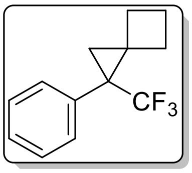

11

$^{13}C\{^{1}H\}$ NMR (101 MHz, $CDCl_3$)

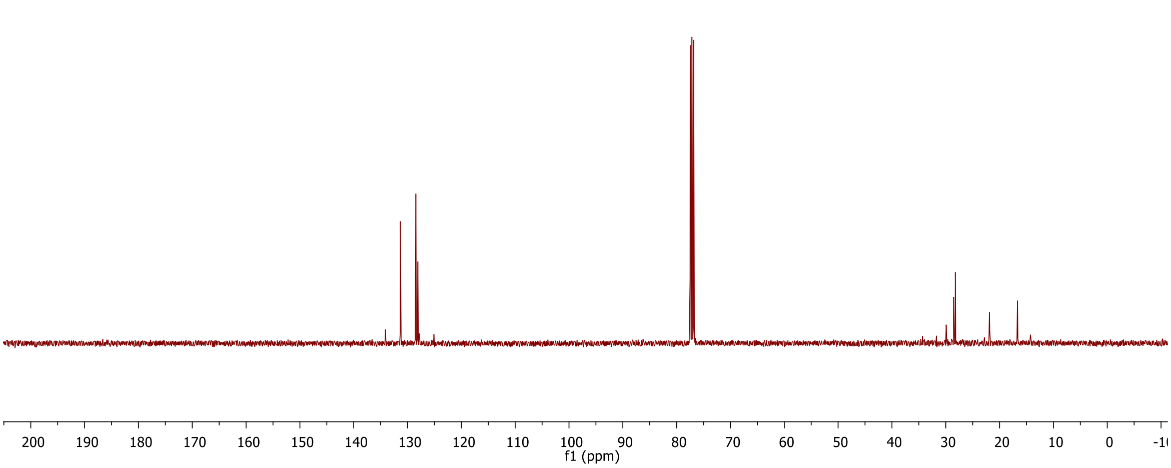

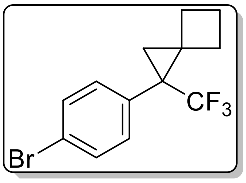

12

^{1}H NMR (400 MHz, $CDCl_3$)

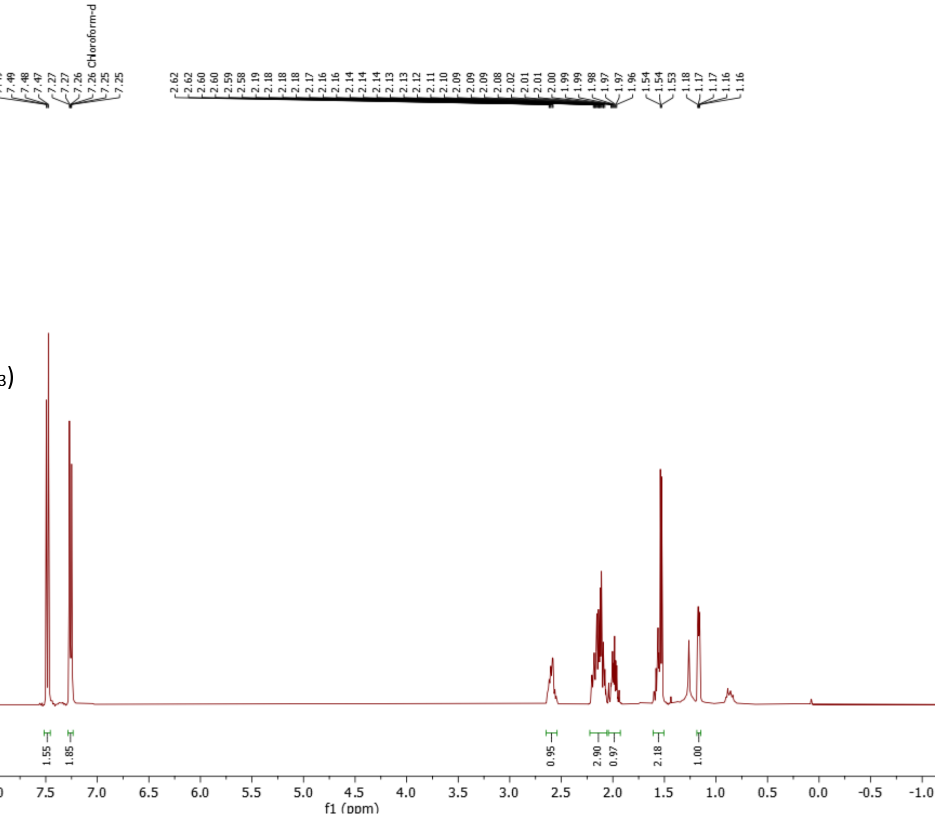

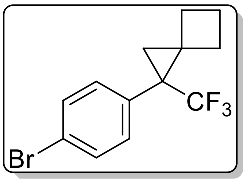

12

^{19}F NMR (377 MHz, $CDCl_3$)

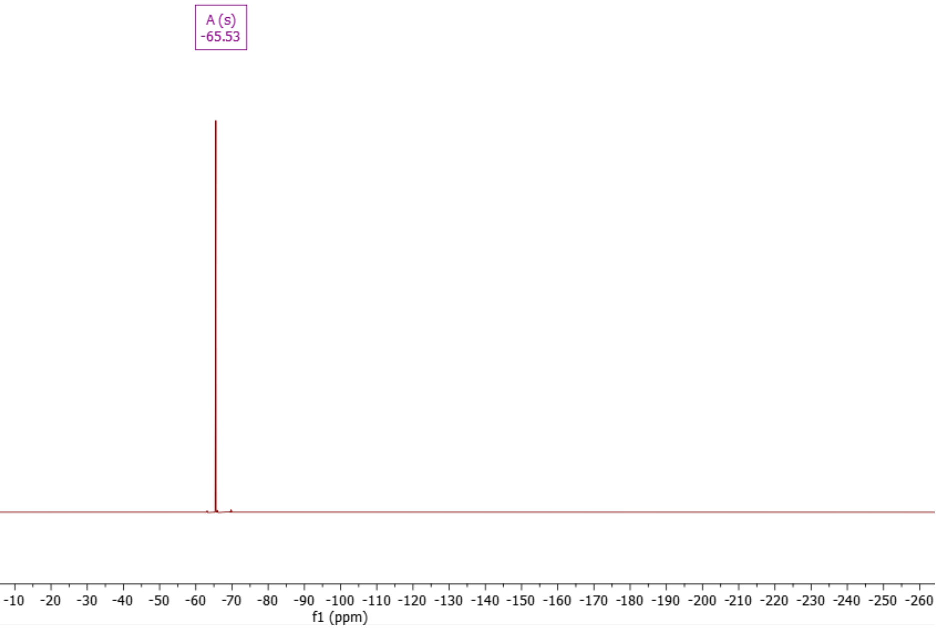

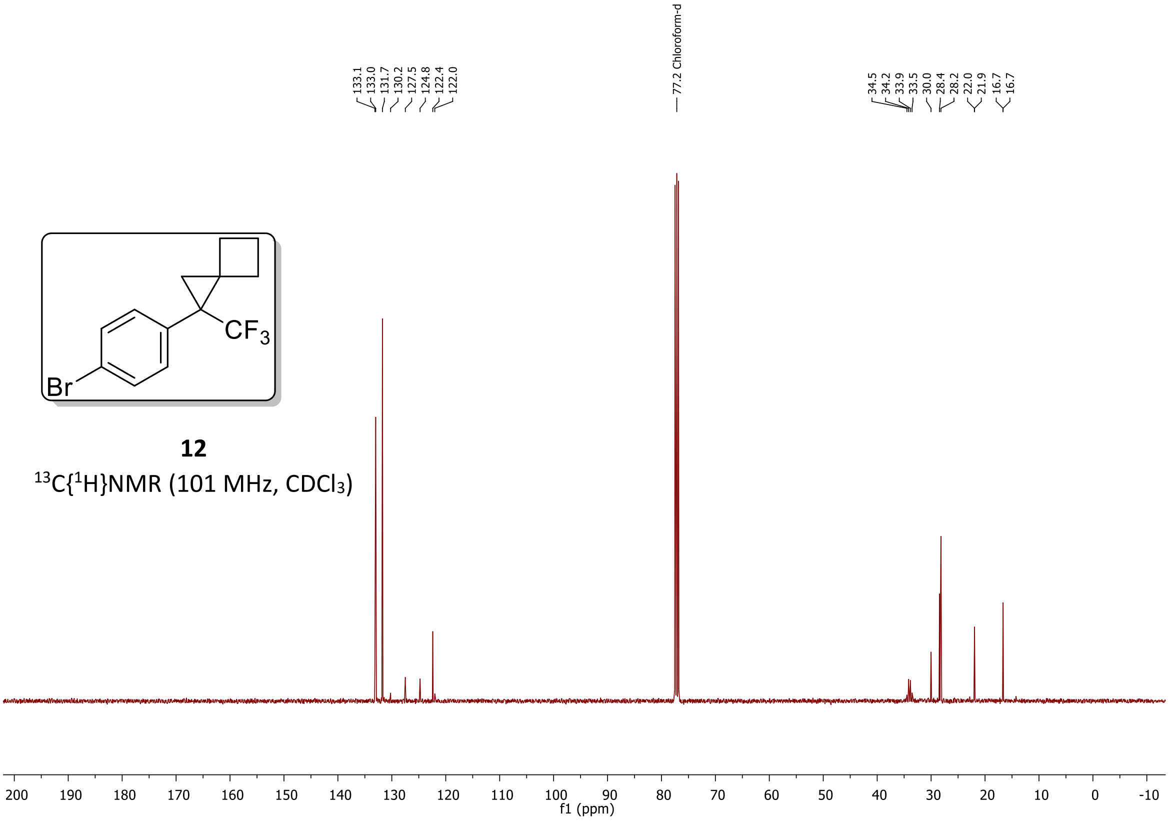

12

$^{13}C\{^{1}H\}$NMR (101 MHz, $CDCl_3$)

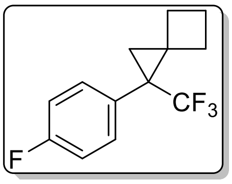

13

^{1}H NMR (400 MHz, $CDCl_3$)

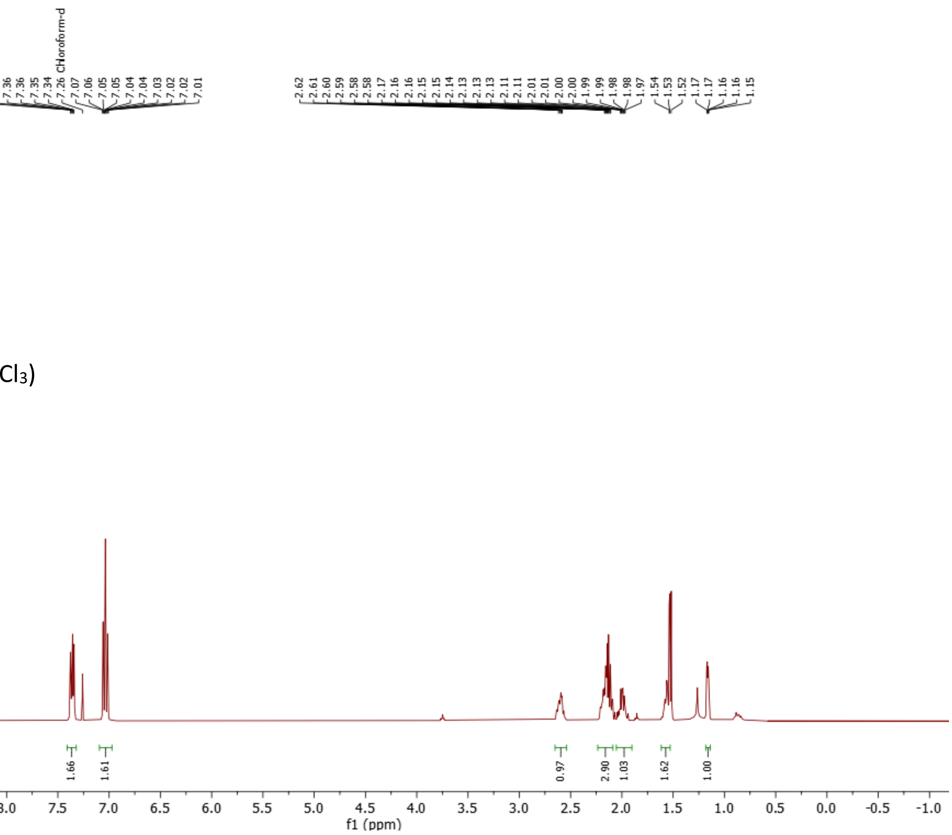

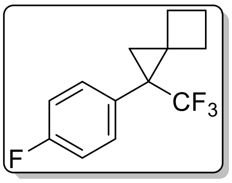

13

^{19}F NMR (377 MHz, $CDCl_3$)

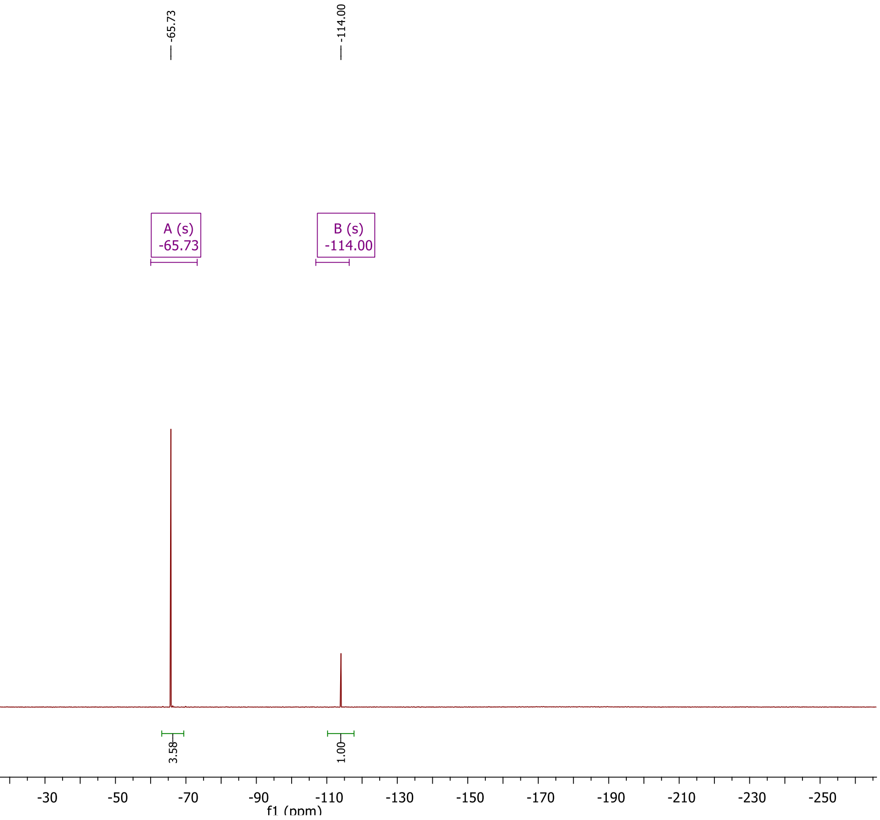

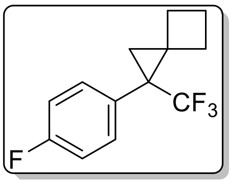

13

$^{13}C\{^1H\}$NMR (101 MHz, $CDCl_3$)

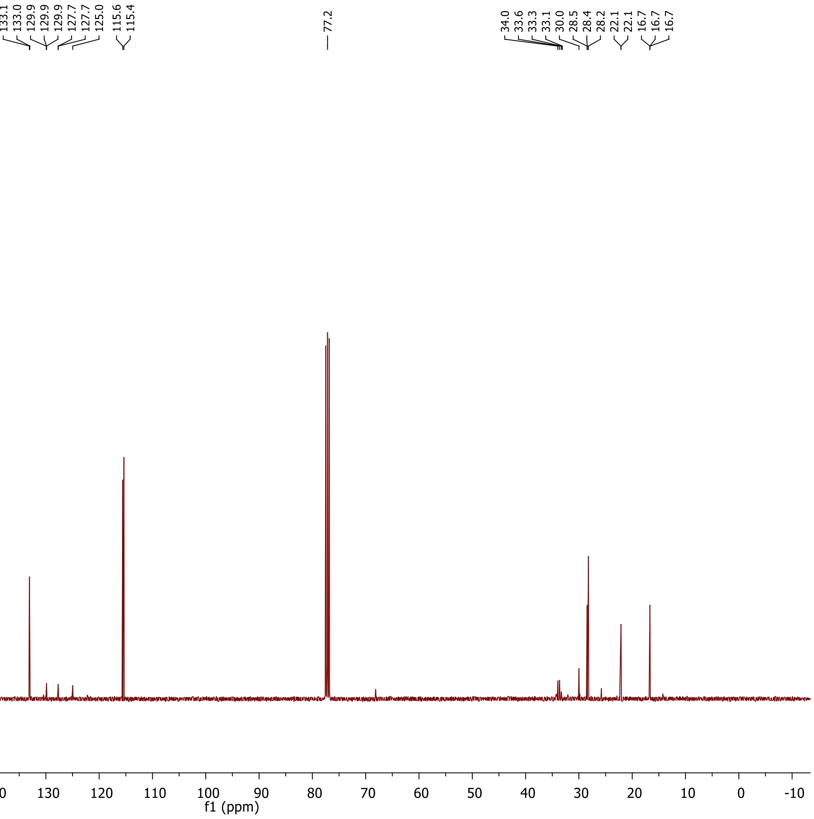

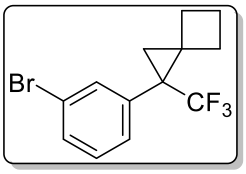

14

^{1}H NMR (400 MHz, $CDCl_3$)

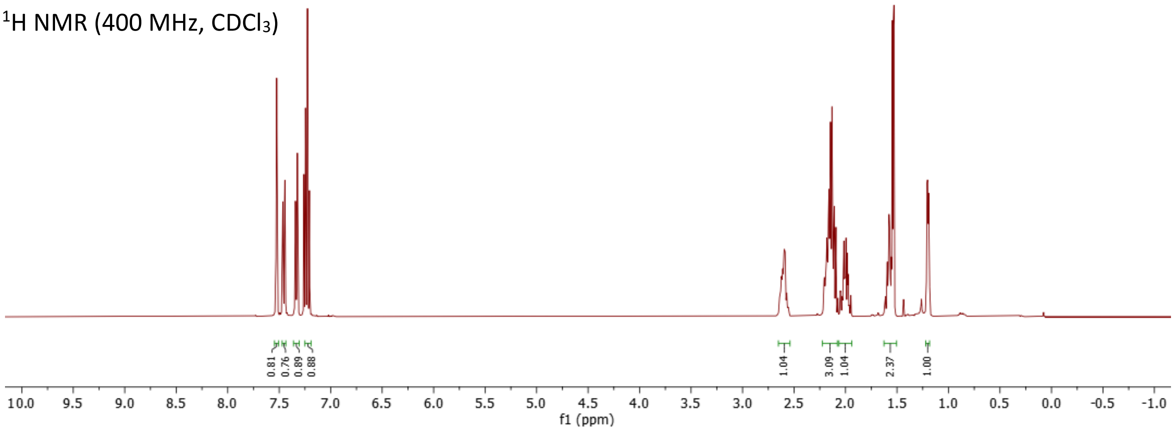

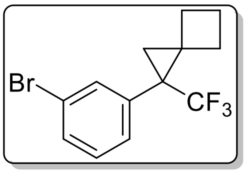

14

^{19}F NMR (377 MHz, $CDCl_3$)

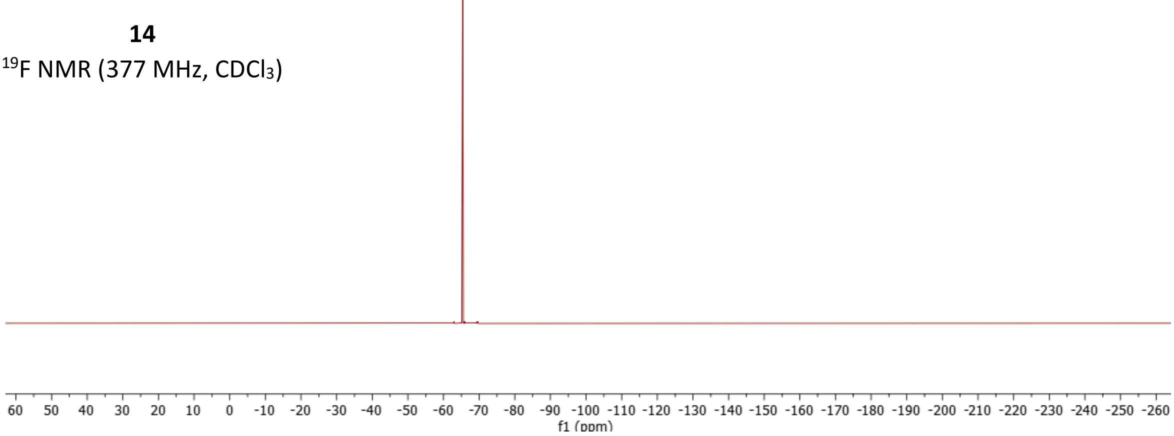

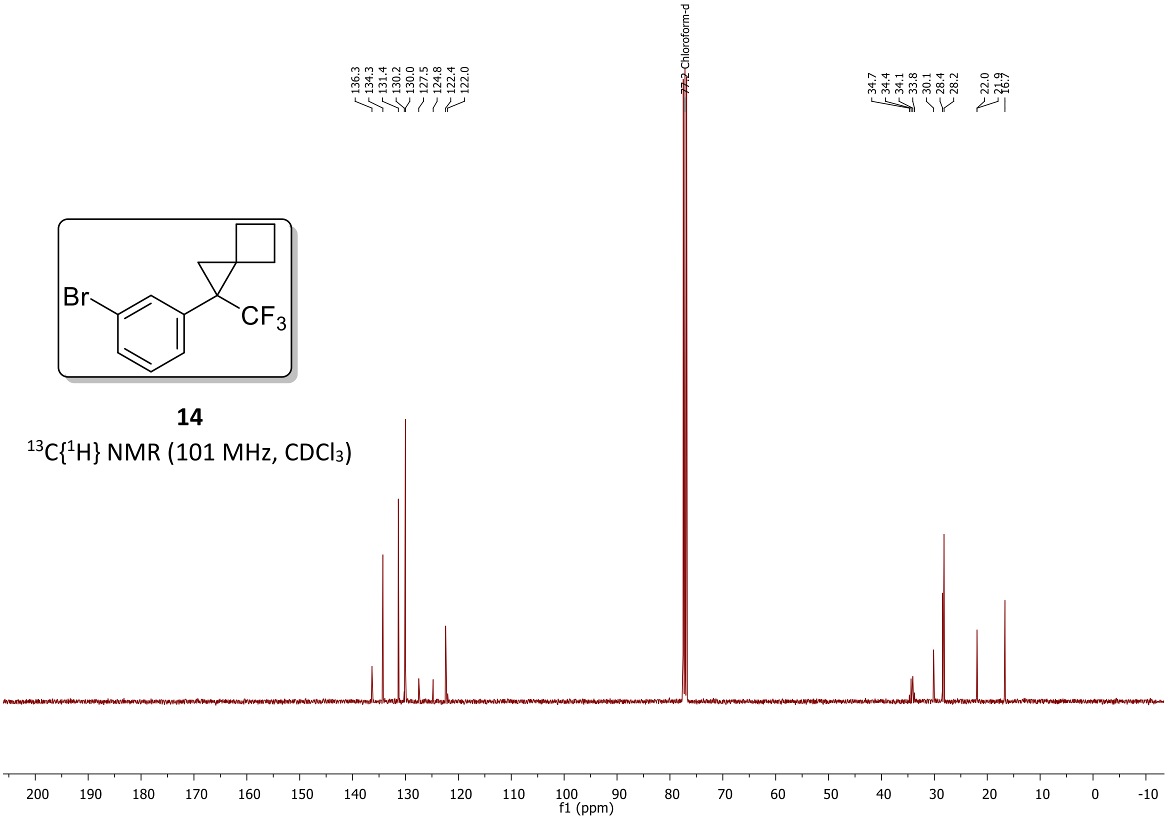

14

$^{13}C\{^{1}H\}$ NMR (101 MHz, $CDCl_3$)

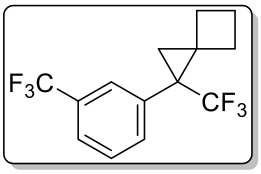

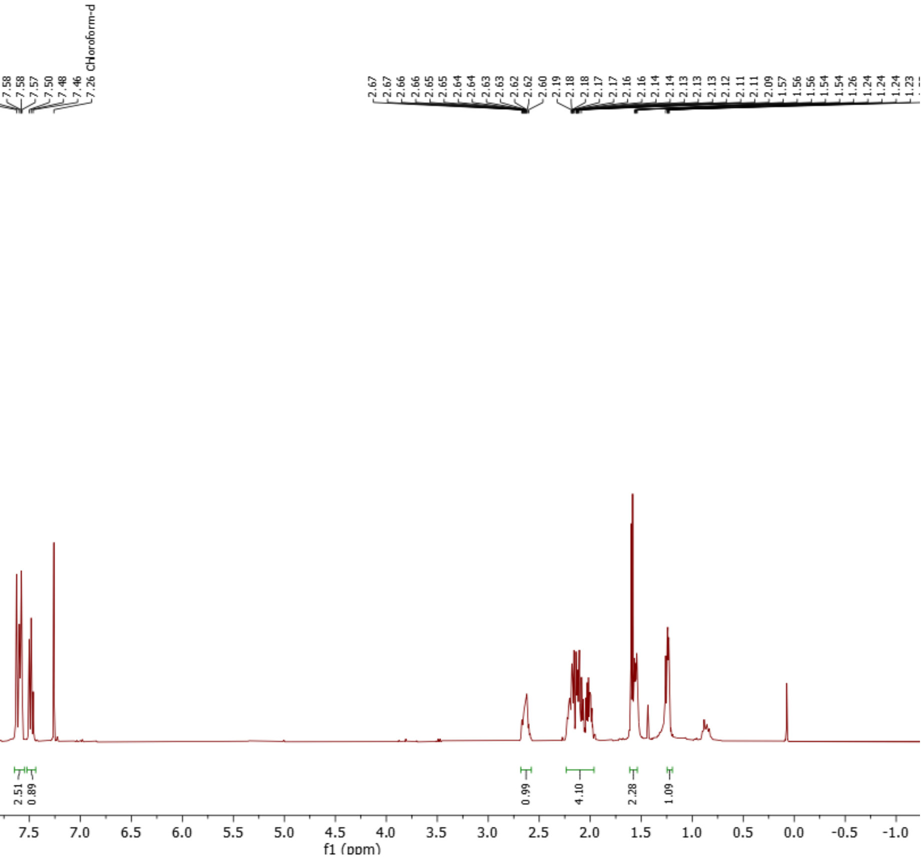

15

^{1}H NMR (400 MHz, $CDCl_3$)

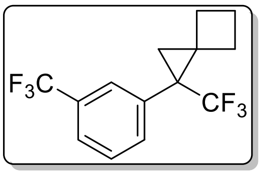

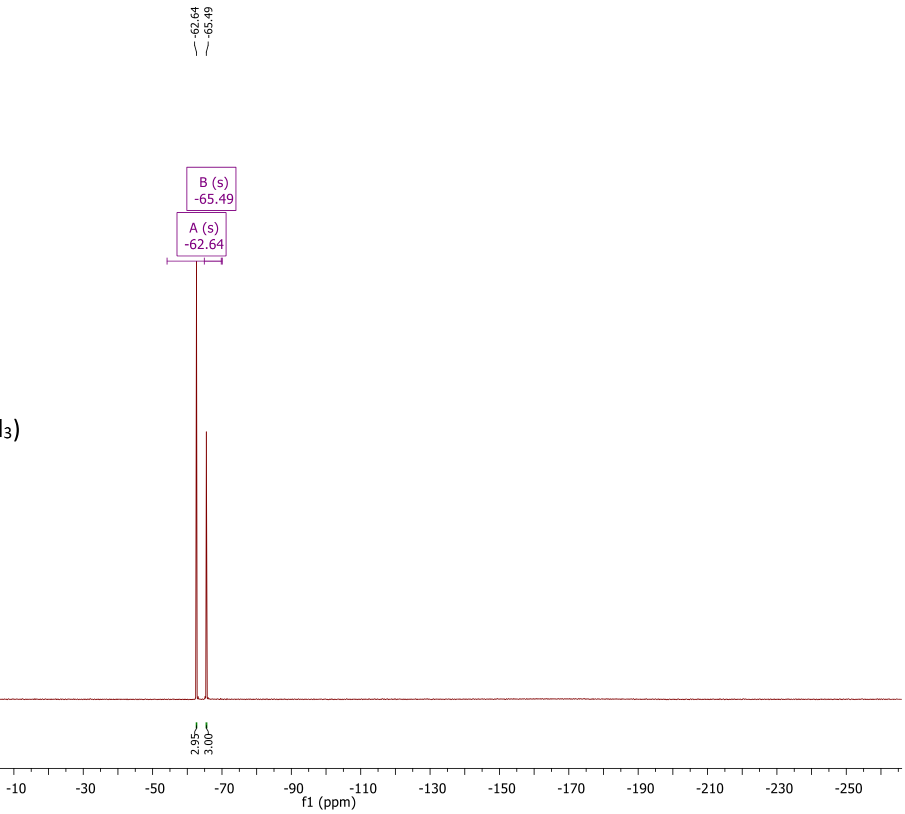

15

^{19}F NMR (377 MHz, $CDCl_3$)

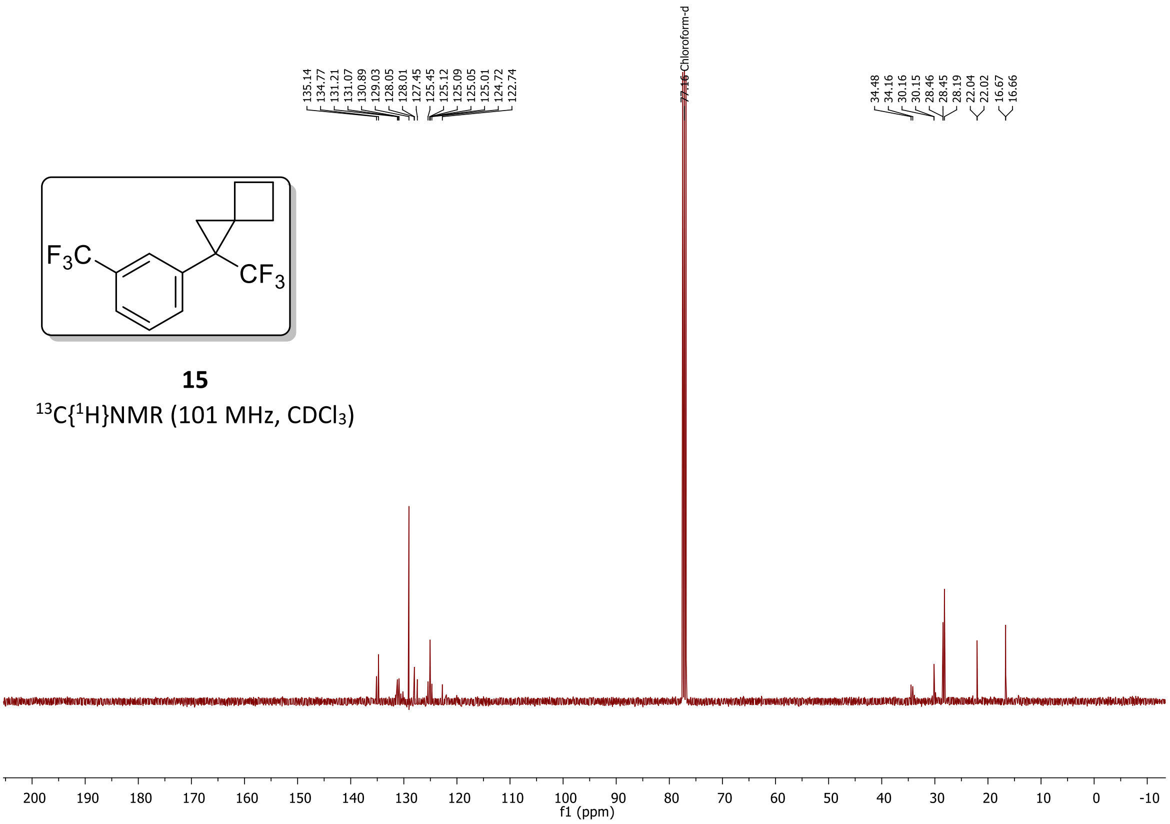

15

$^{13}C\{^{1}H\}$NMR (101 MHz, $CDCl_3$)

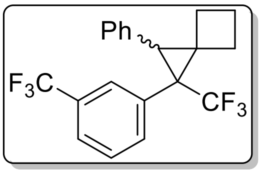

16

^{1}H NMR (400 MHz, $CDCl_3$)

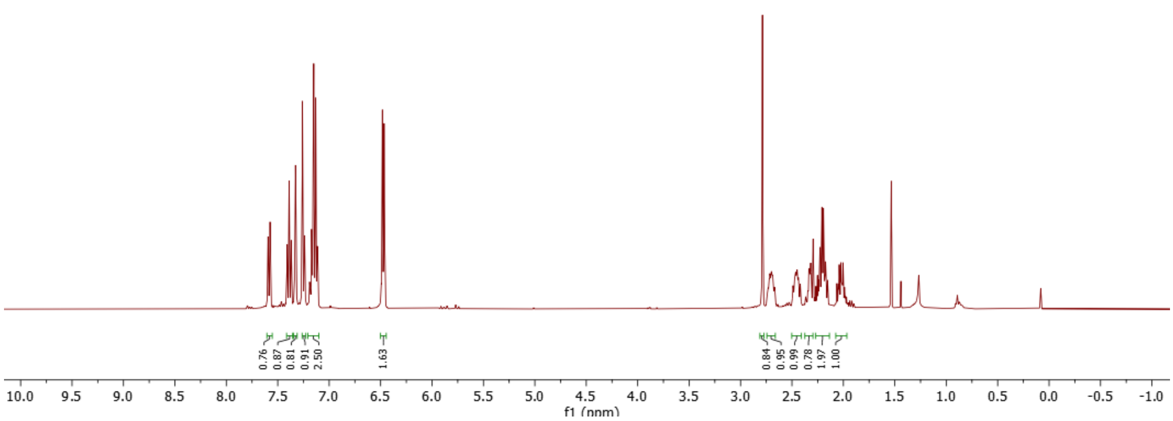

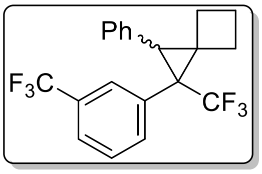

16

^{19}F NMR (377 MHz, $CDCl_3$)

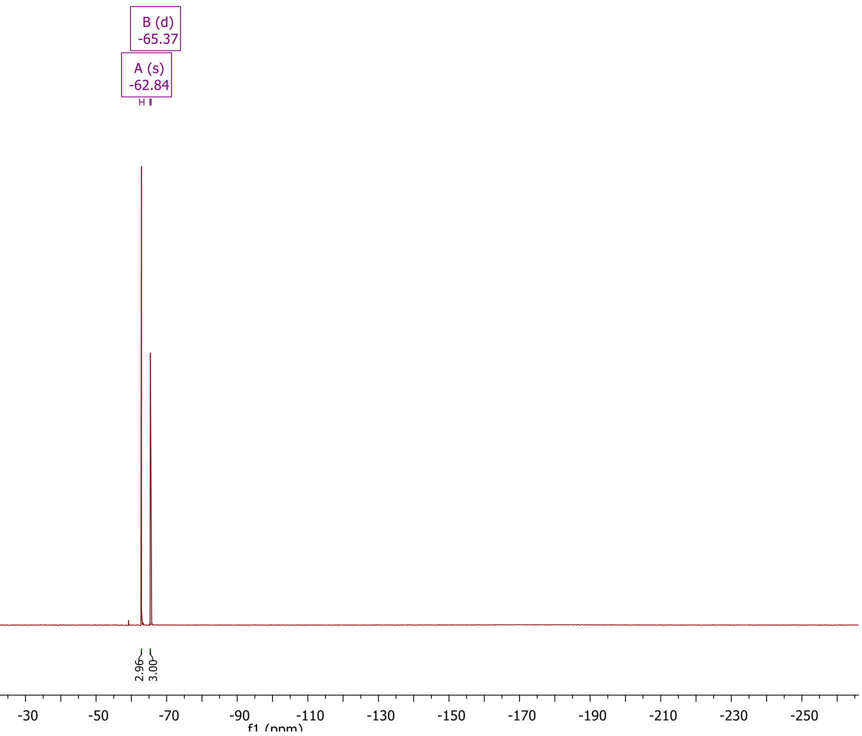

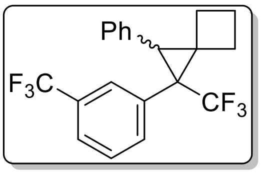

16

^{13}C {^{1}H} NMR (101 MHz, $CDCl_3$)

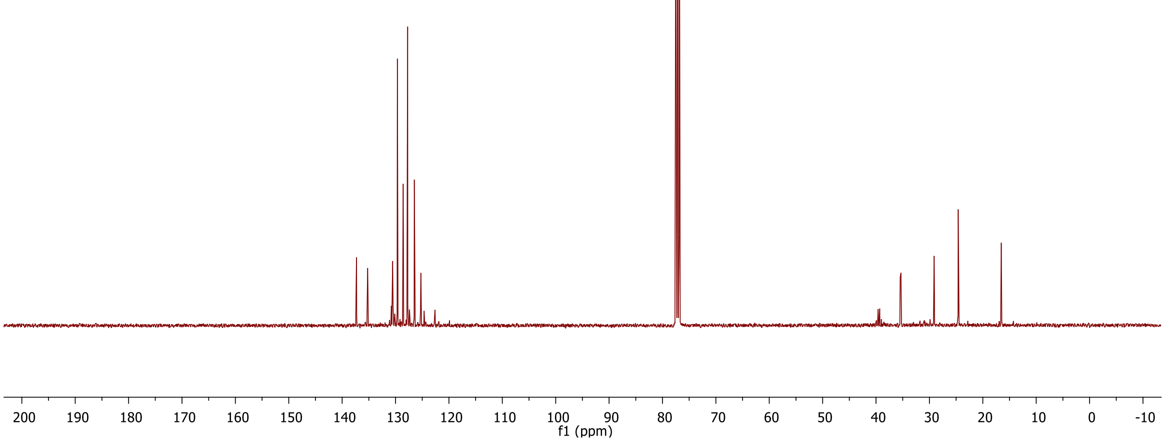

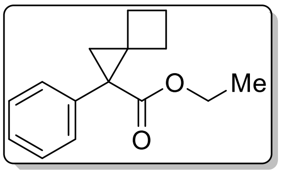

17

^{1}H NMR (400 MHz, $CDCl_3$)

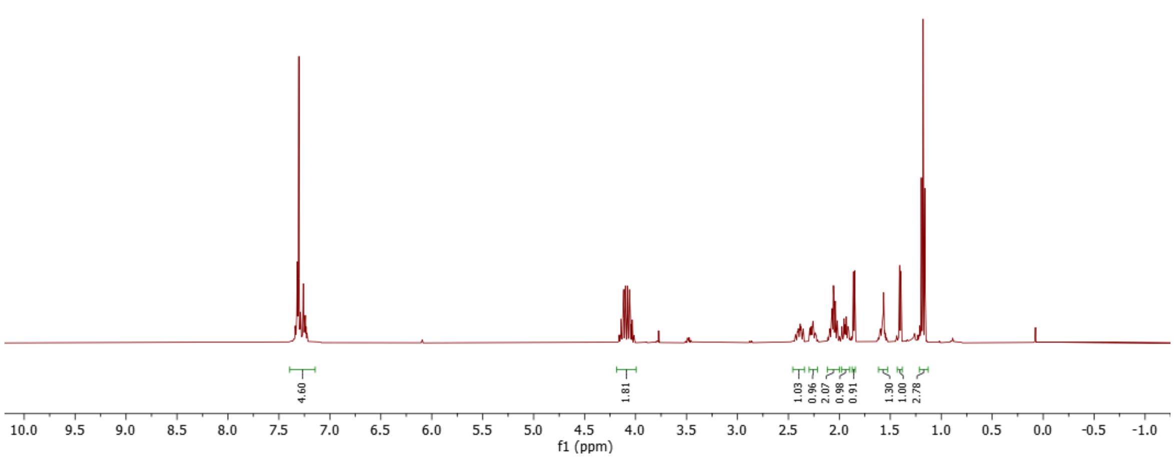

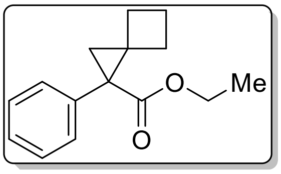

17

^{13}C {^{1}H} NMR (101 MHz, $CDCl_3$)

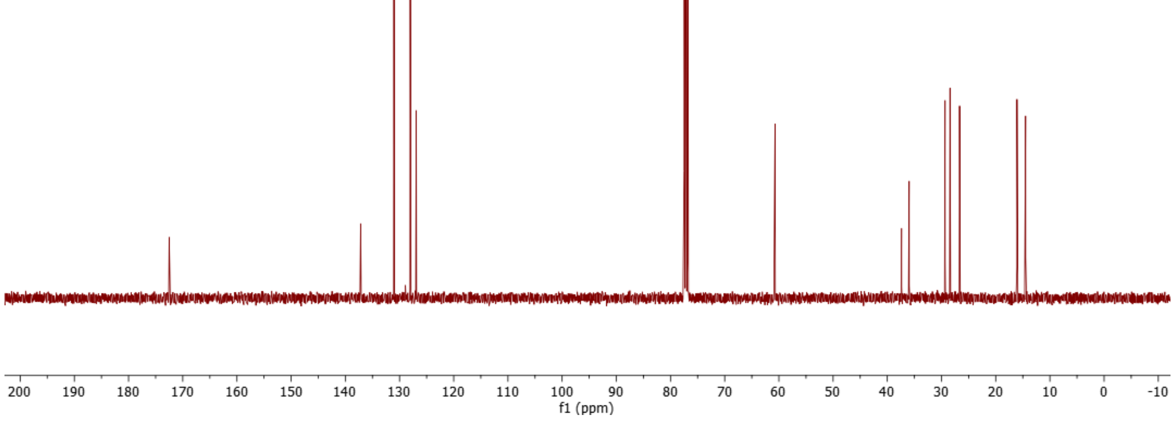

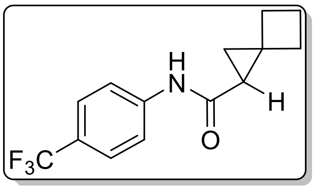

18
^{1}H NMR (400 MHz, $CDCl_3$)

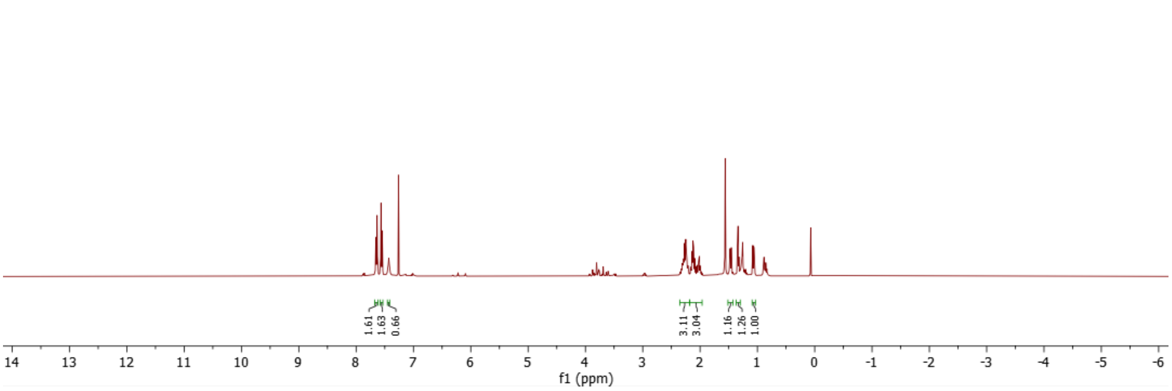

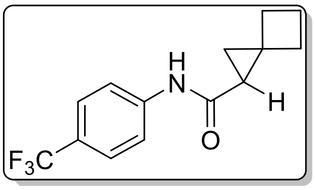

18
^{19}F NMR (377 MHz, $CDCl_3$)

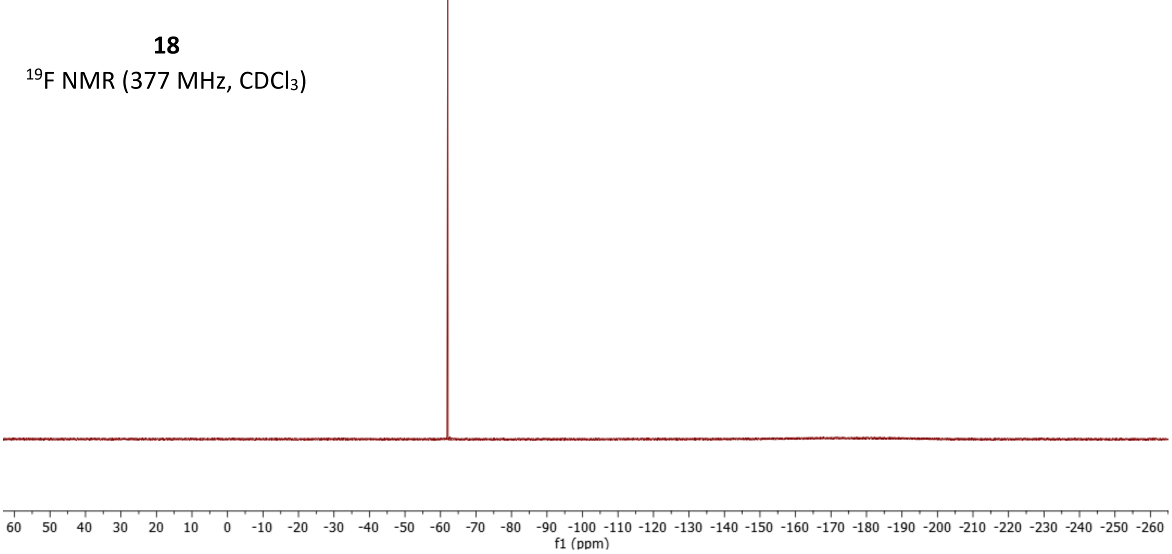

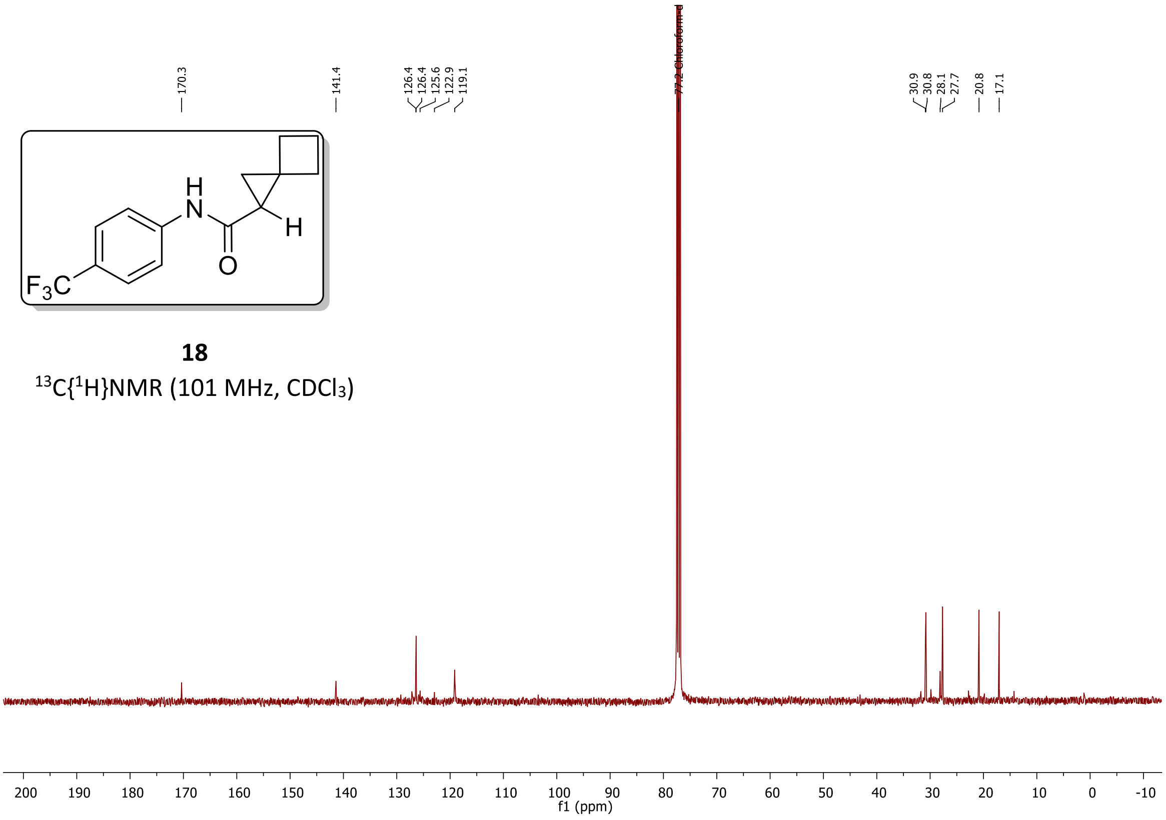

18
$^{13}C\{^{1}H\}$NMR (101 MHz, $CDCl_3$)

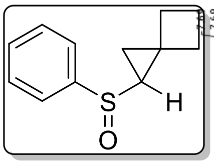

19

^{1}H NMR (400 MHz, $CDCl_3$)

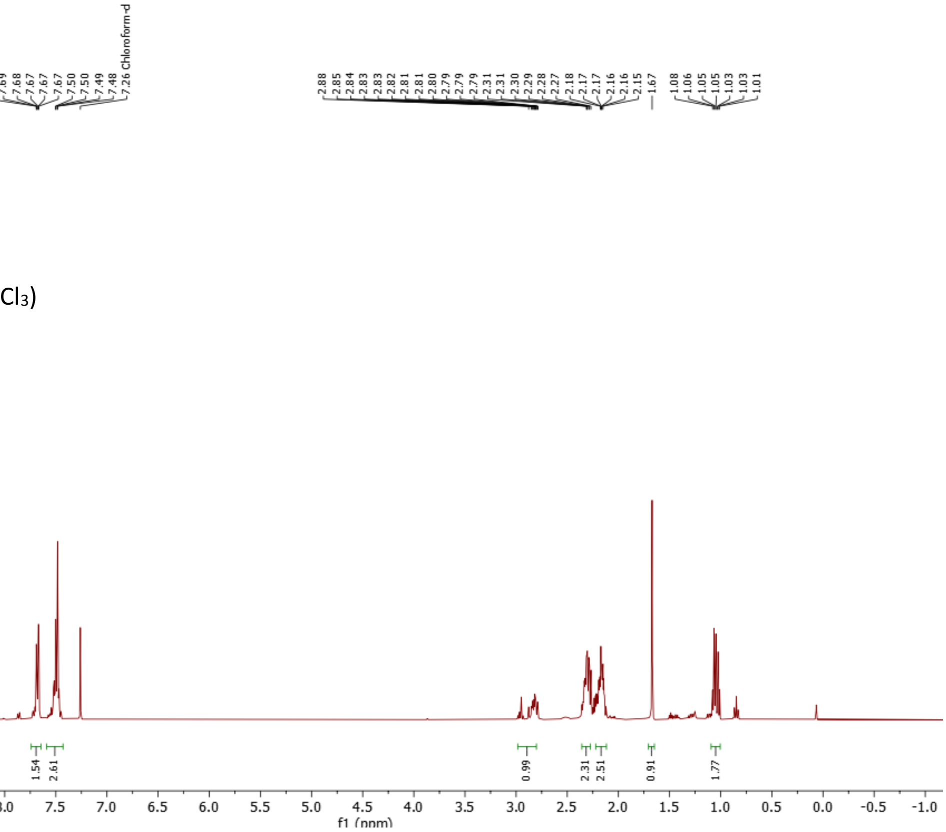

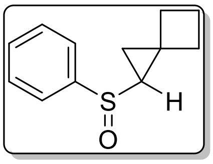

19

^{13}C {^{1}H} NMR (101 MHz, $CDCl_3$)

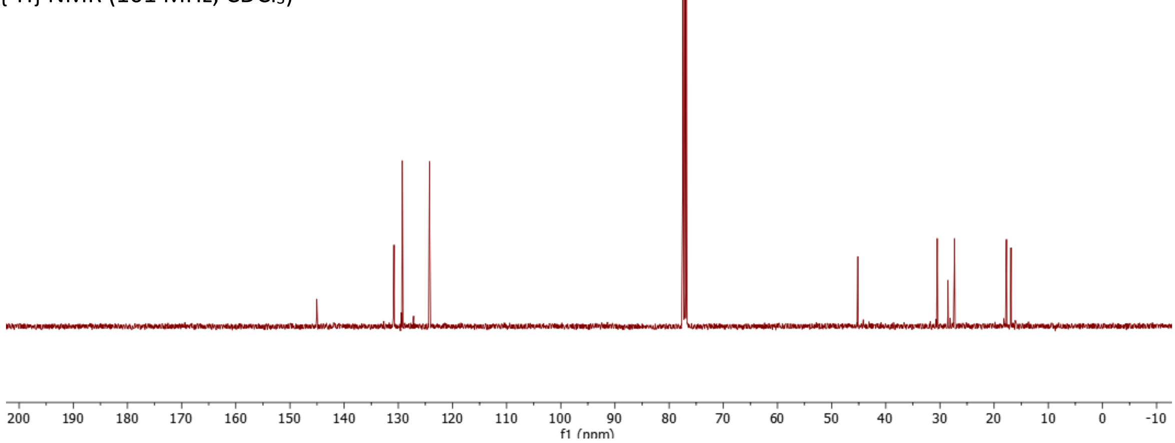

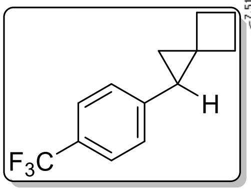

20

^{1}H NMR (400 MHz, $CDCl_3$)

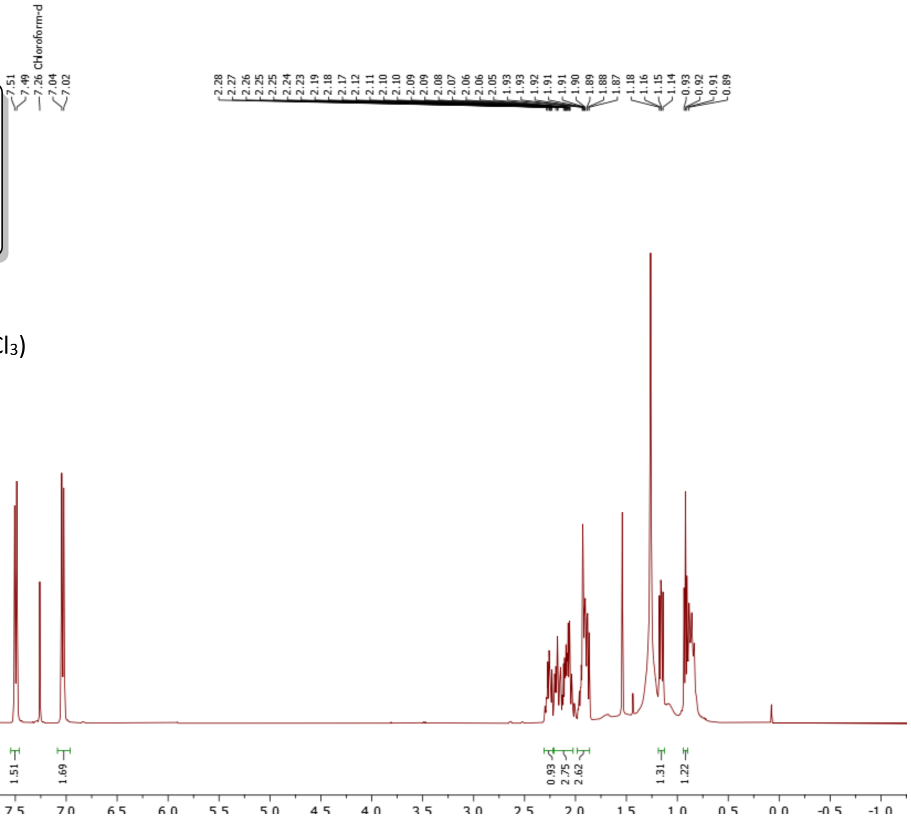

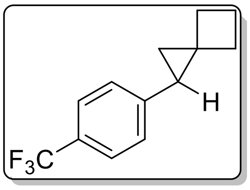

20

^{19}F NMR (377 MHz, $CDCl_3$)

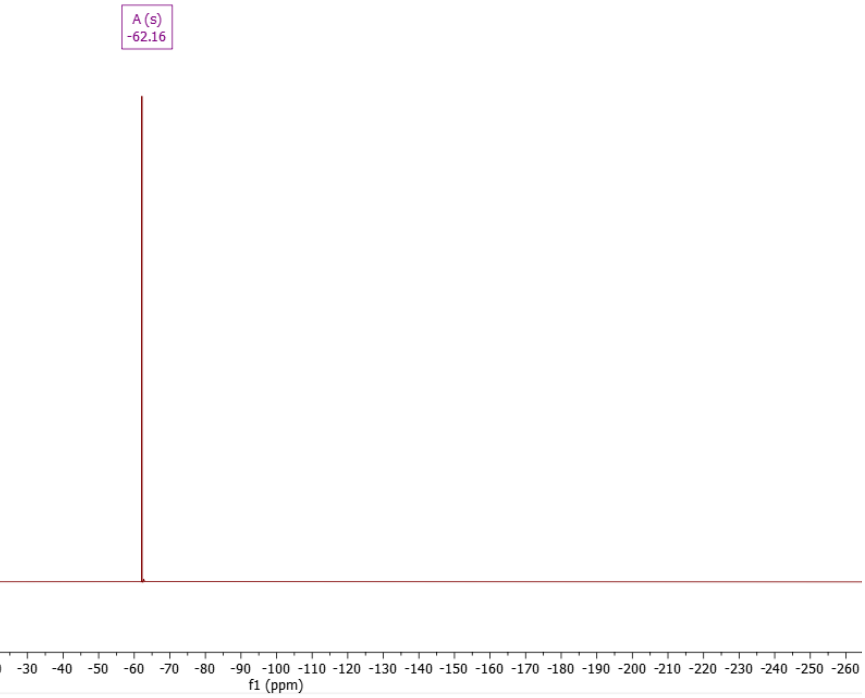

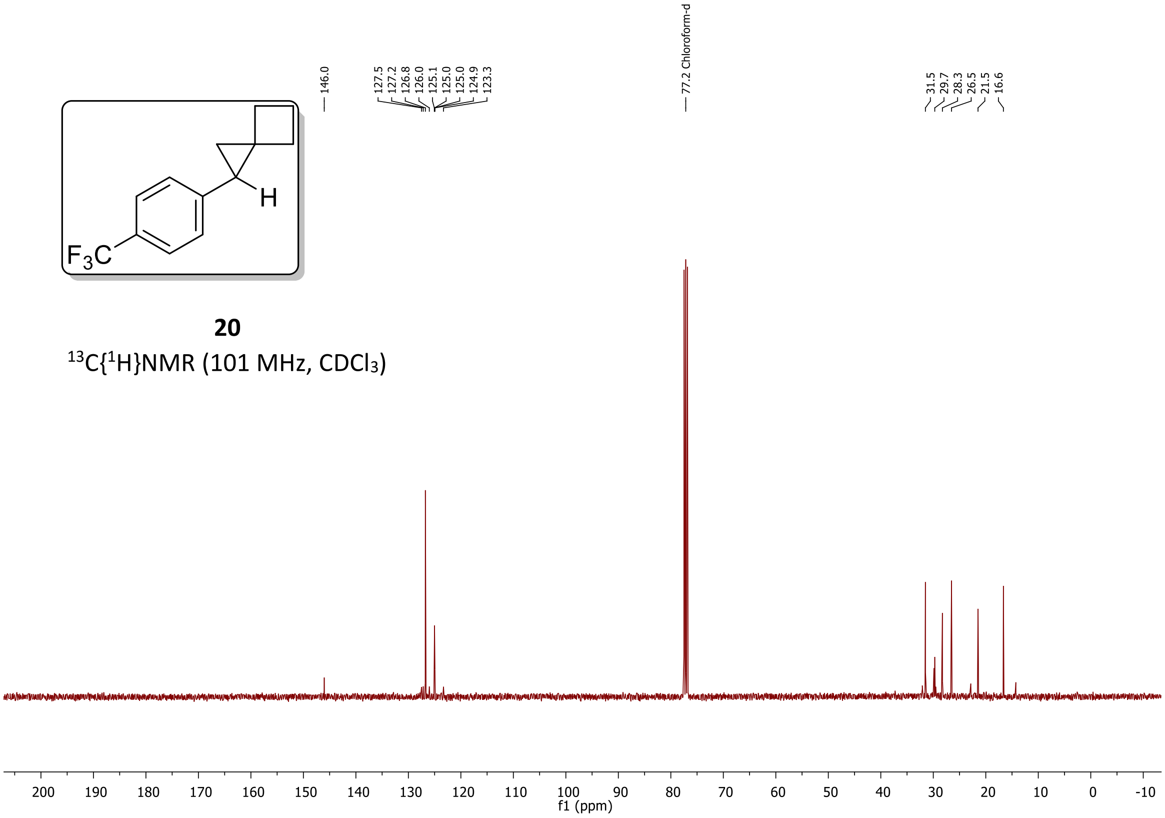

20

$^{13}C\{^1H\}$NMR (101 MHz, CDCl₃)

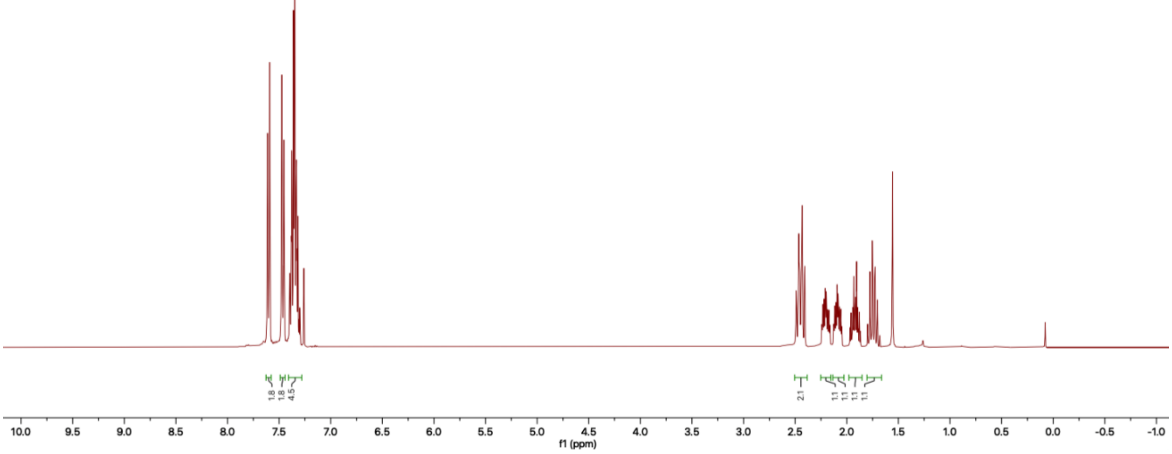

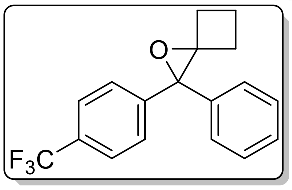

23

^{1}H NMR (400 MHz, $CDCl_3$)

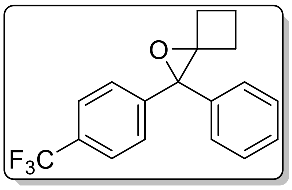

23

^{19}F NMR (377 MHz, $CDCl_3$)

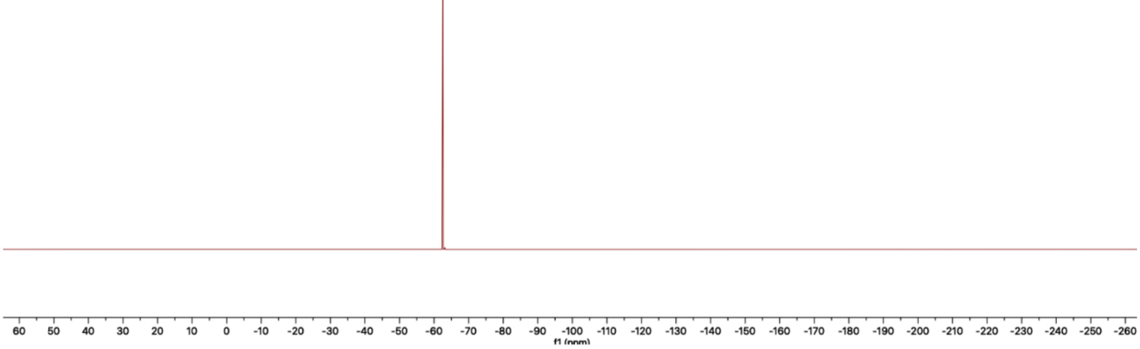

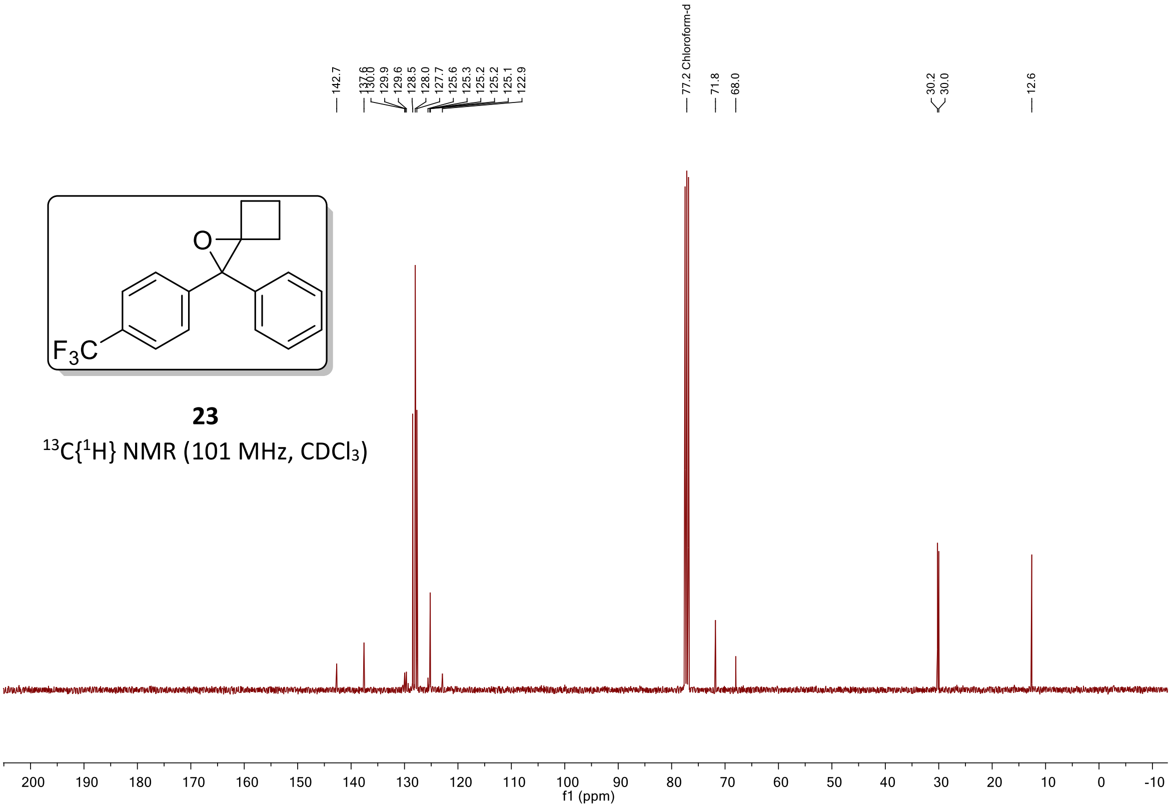

23

$^{13}C\{^{1}H\}$ NMR (101 MHz, $CDCl_3$)

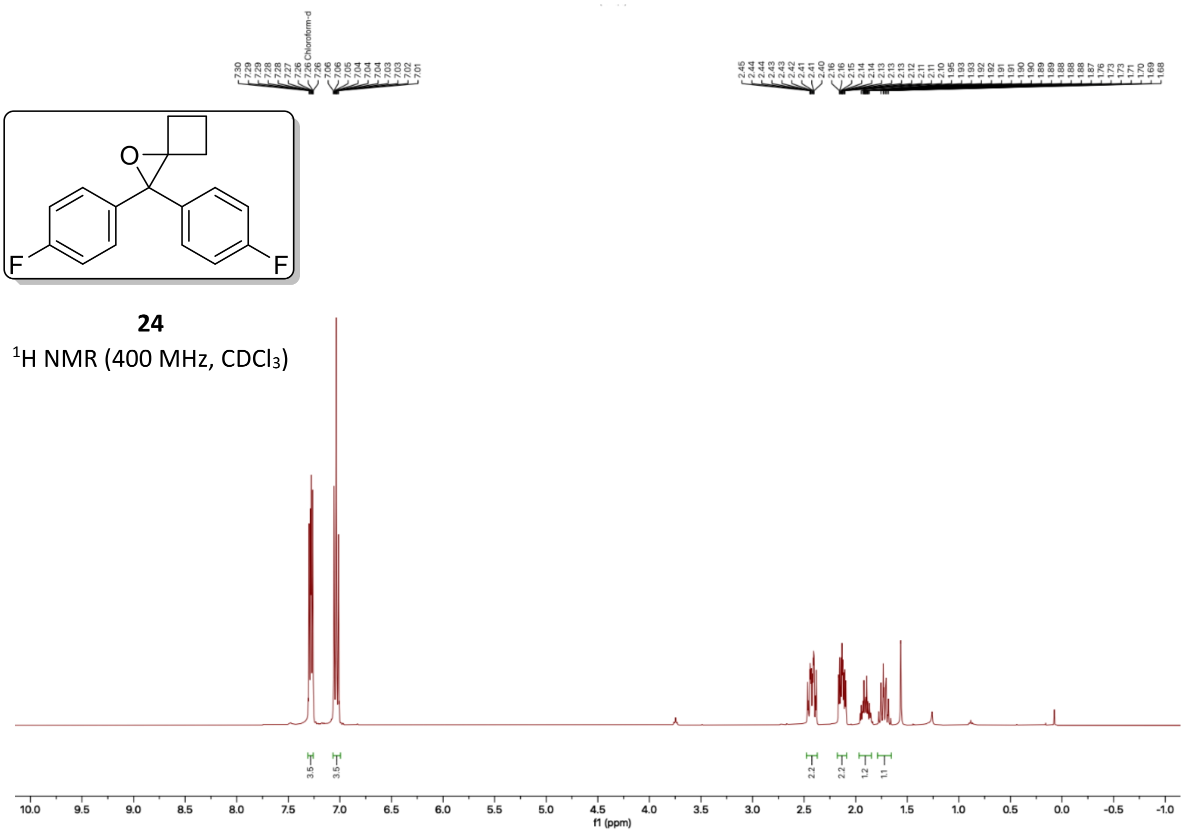

24

^{1}H NMR (400 MHz, $CDCl_3$)

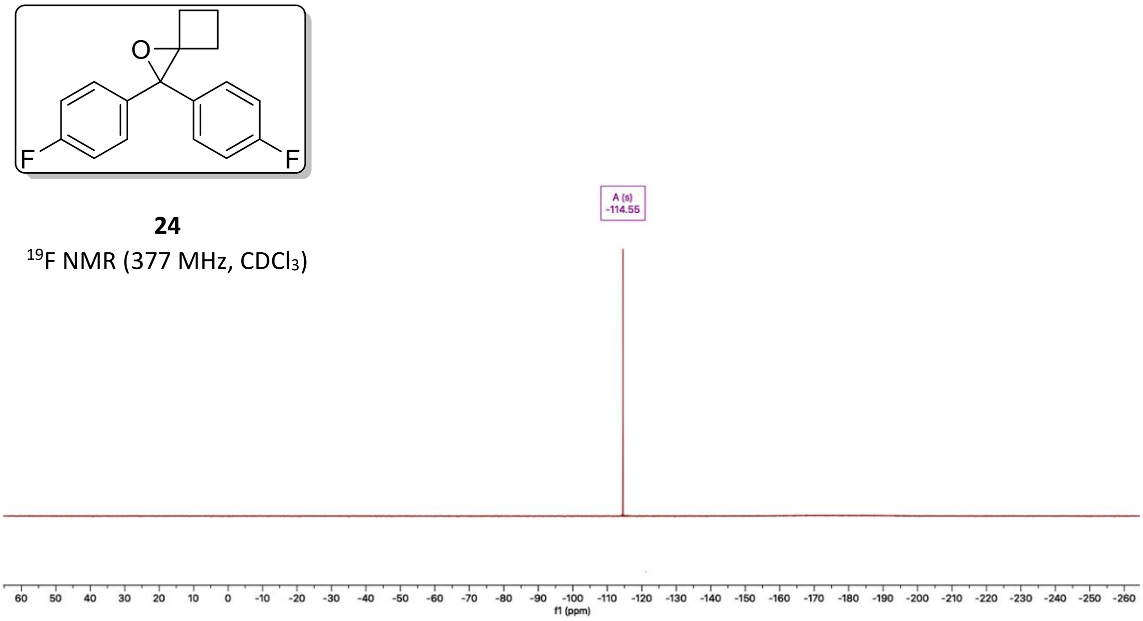

24

^{19}F NMR (377 MHz, $CDCl_3$)

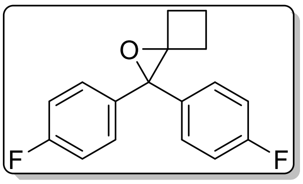

24

$^{13}C\{^{1}H\}$ NMR (101 MHz, $CDCl_3$)

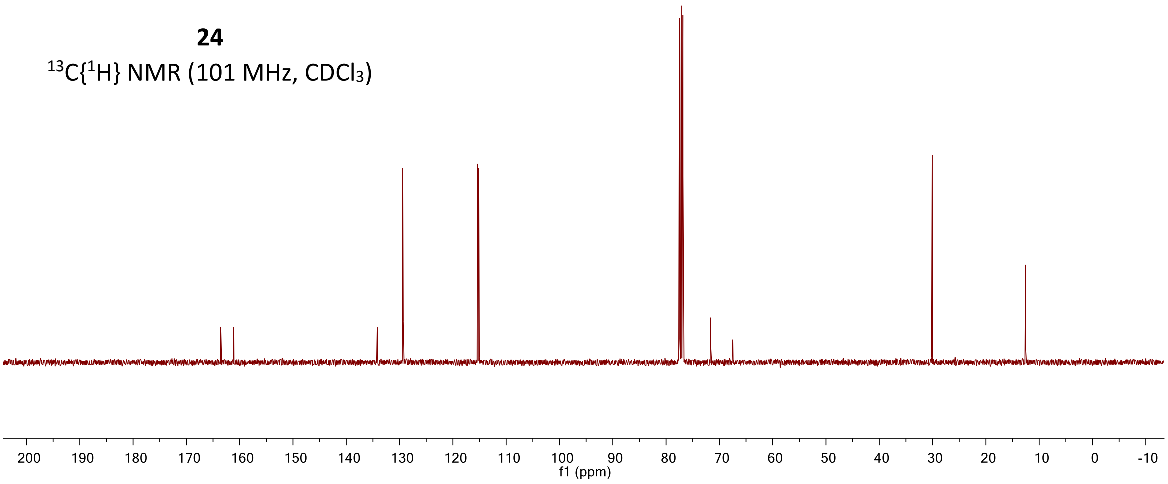

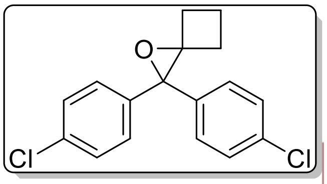

25

^{1}H NMR (400 MHz, $CDCl_3$)

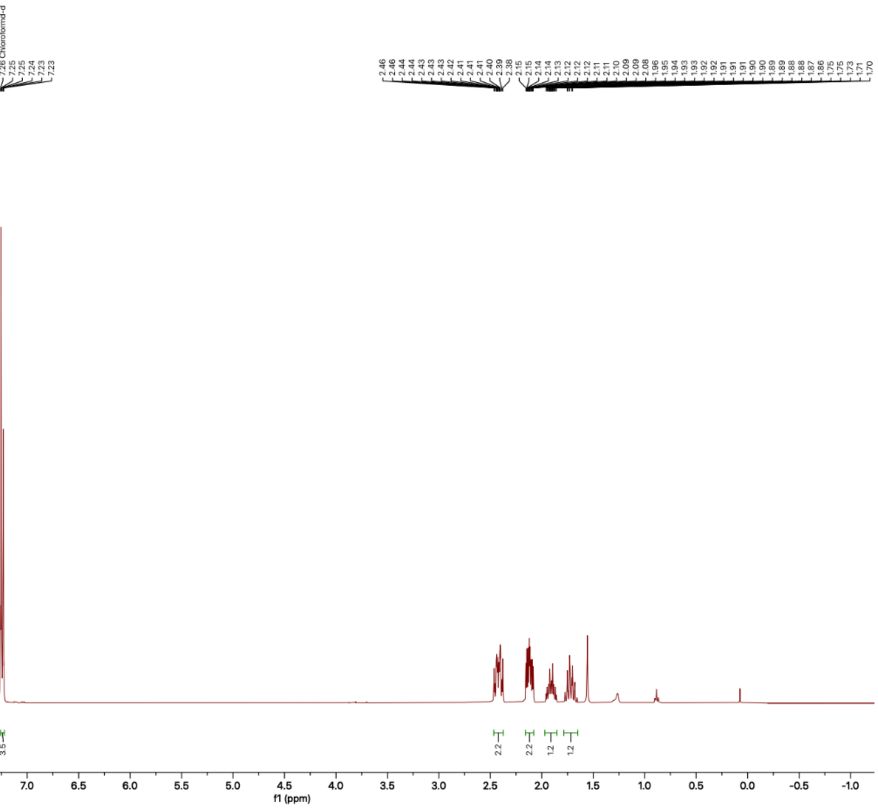

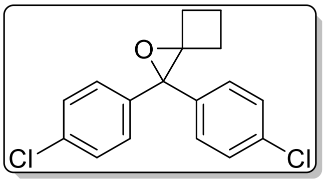

25

^{13}C {^{1}H} NMR (101 MHz, $CDCl_3$)

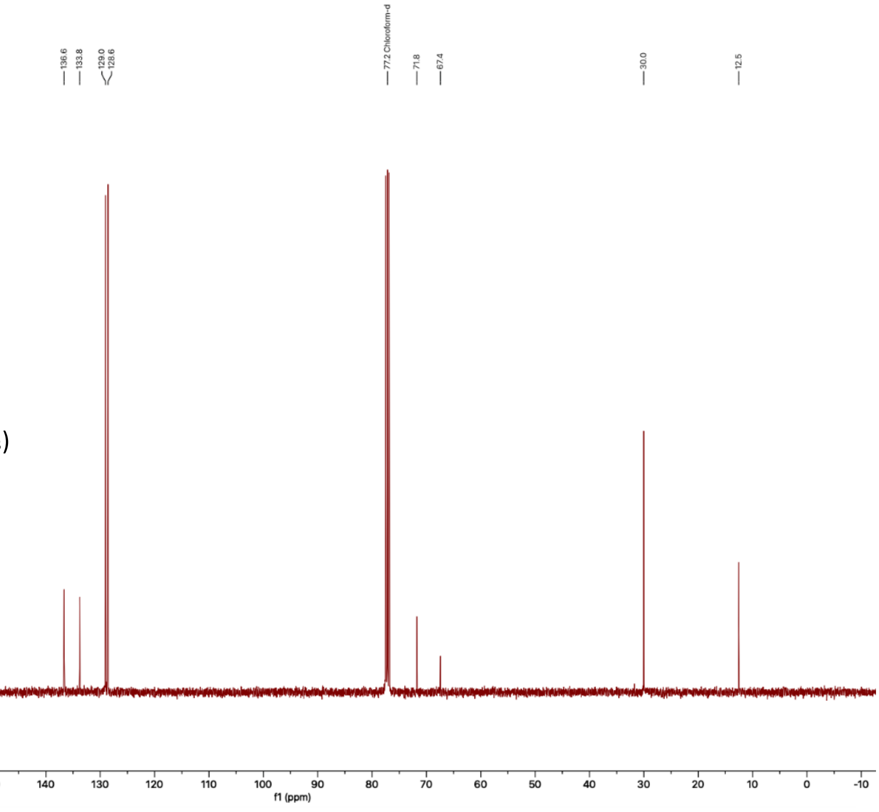

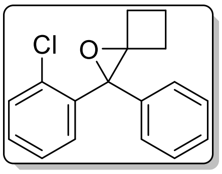

26

^{1}H NMR (400 MHz, $CDCl_3$)

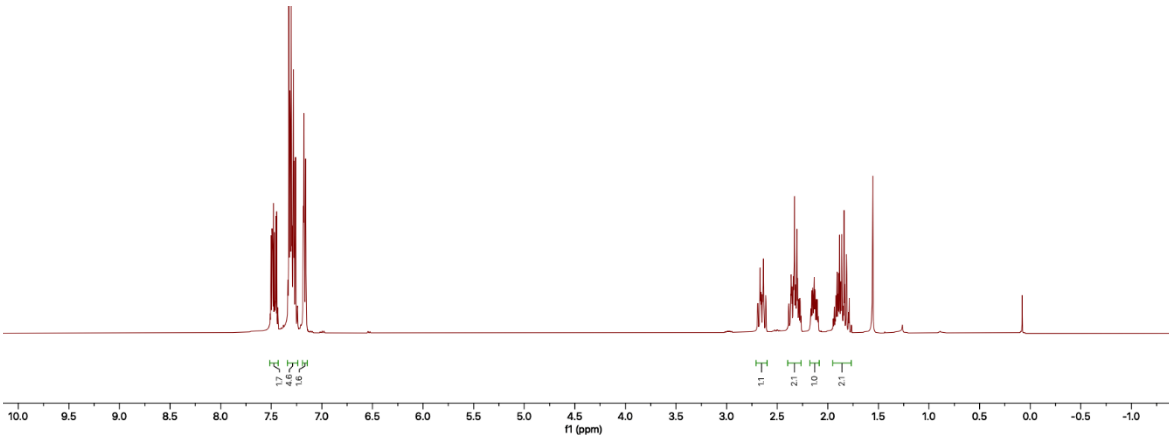

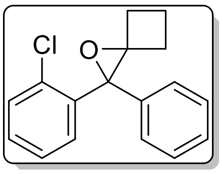

26

^{13}C {^{1}H} NMR (101 MHz, $CDCl_3$)

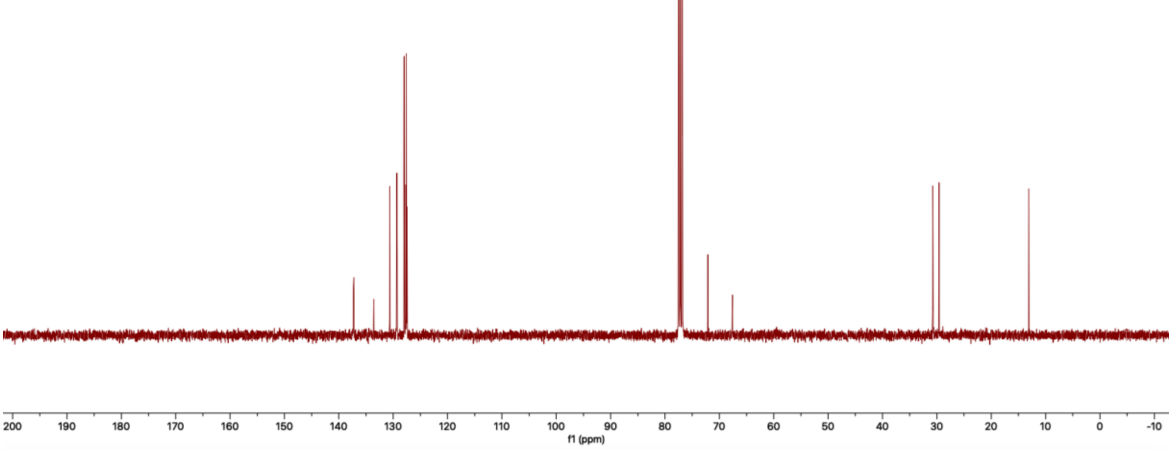

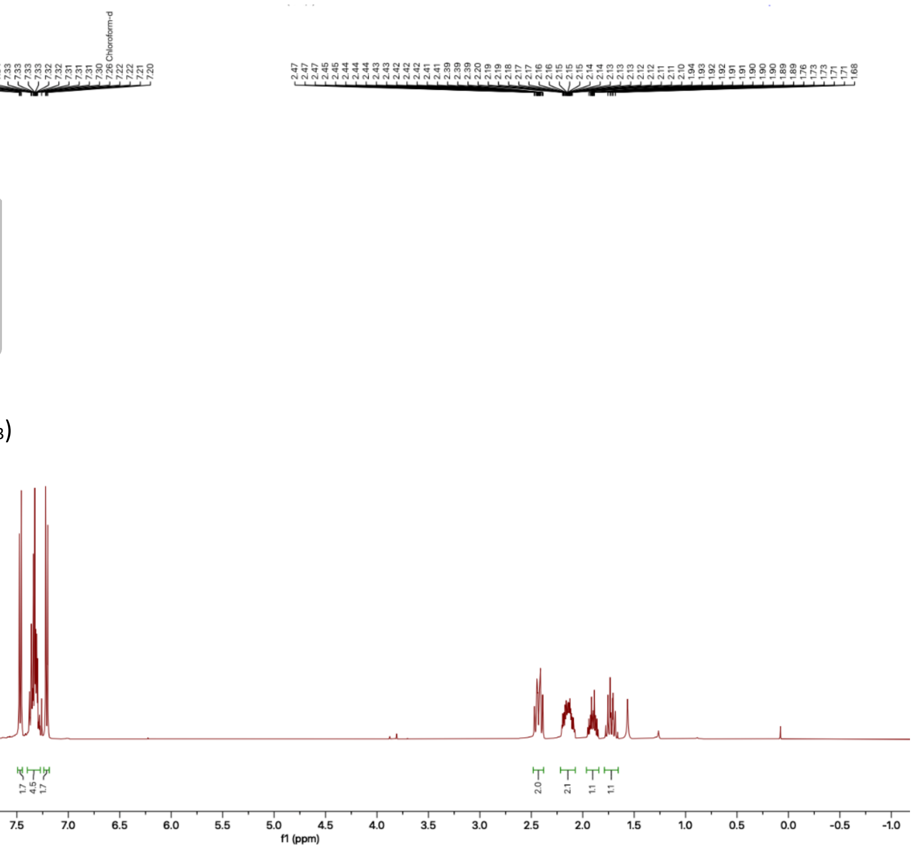

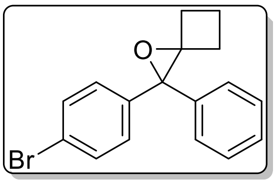

27
^{1}H NMR (400 MHz, $CDCl_3$)

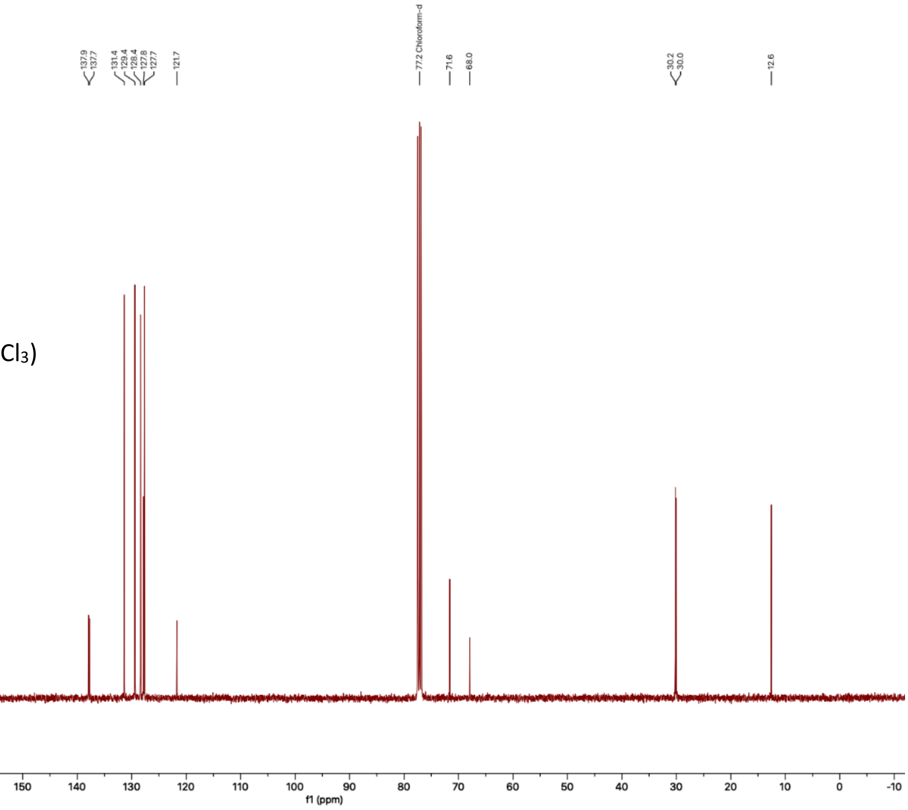

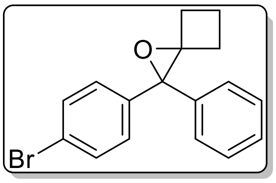

27
^{13}C {^{1}H} NMR (101 MHz, $CDCl_3$)

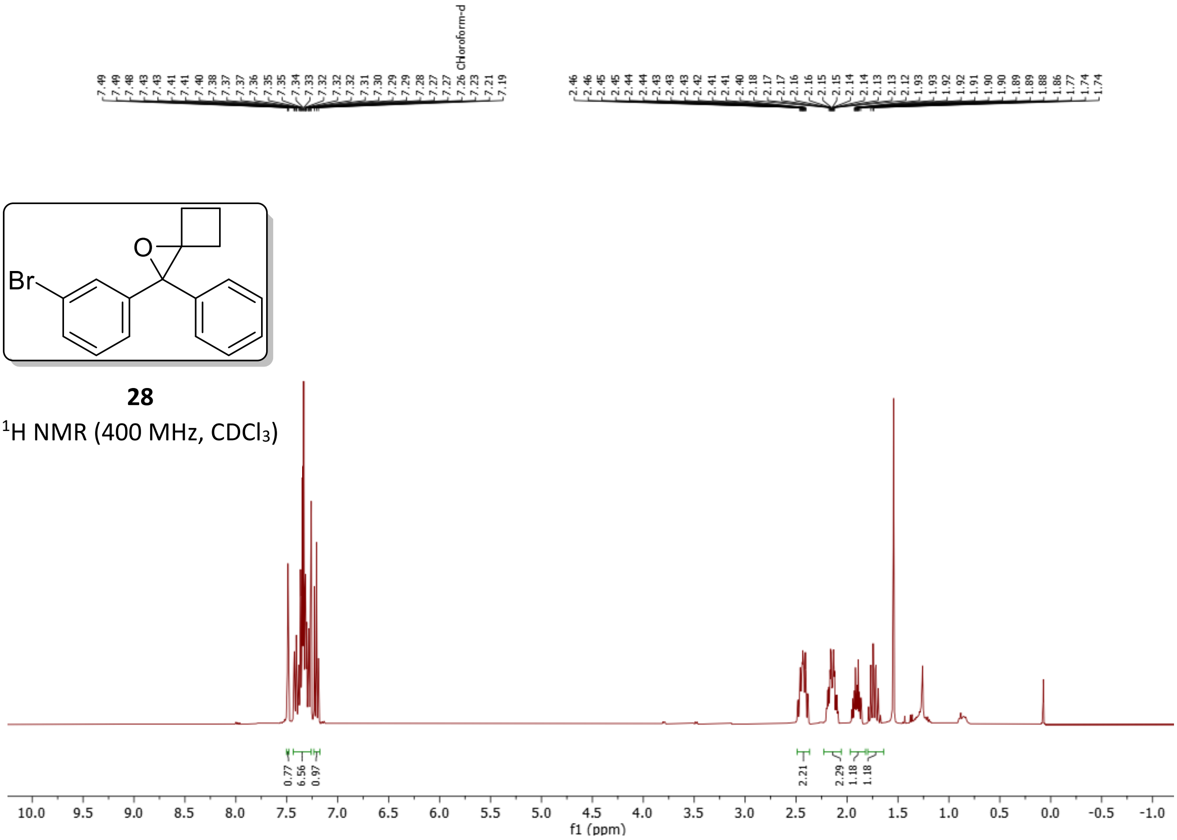

28

^{1}H NMR (400 MHz, CDCl$_3$)

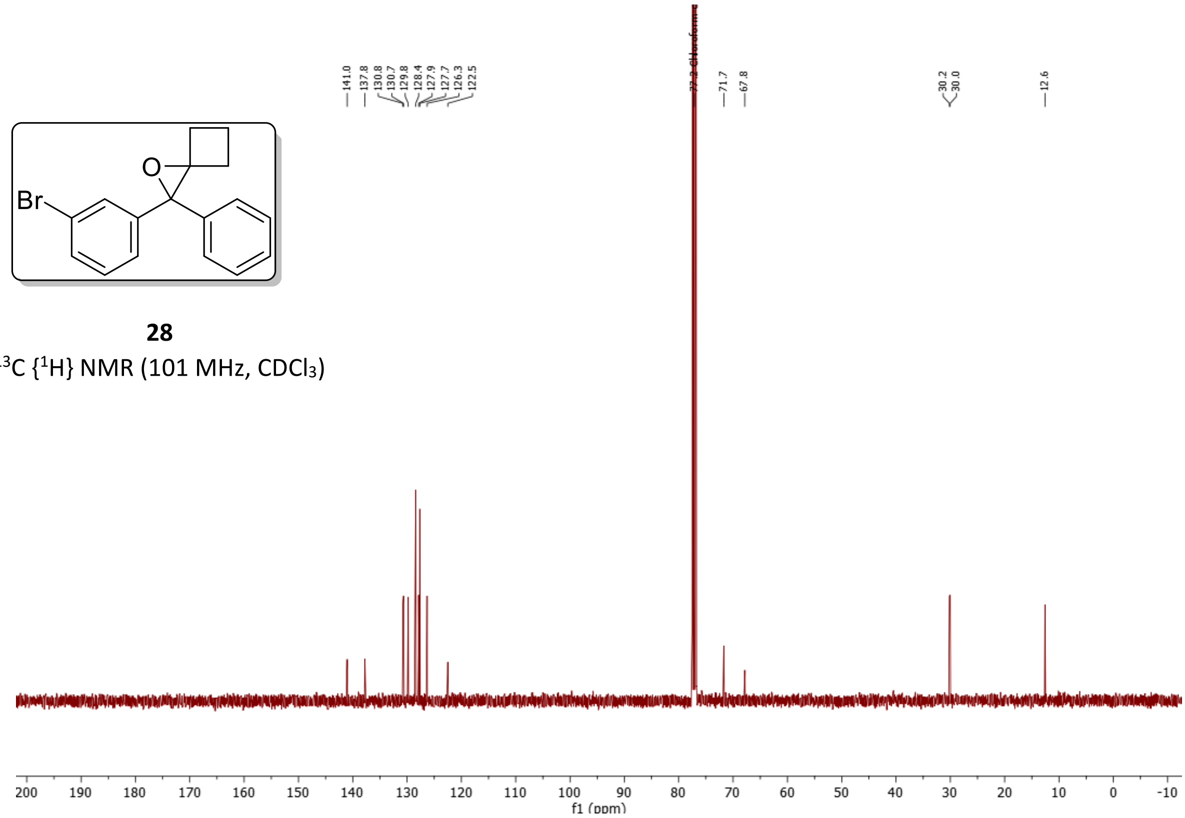

28

^{13}C {^{1}H} NMR (101 MHz, CDCl$_3$)

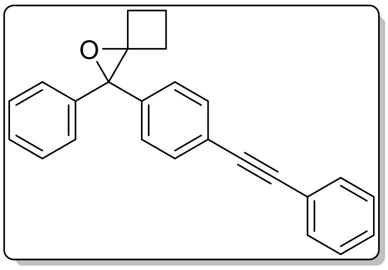

29

^{1}H NMR (400 MHz, $CDCl_3$)

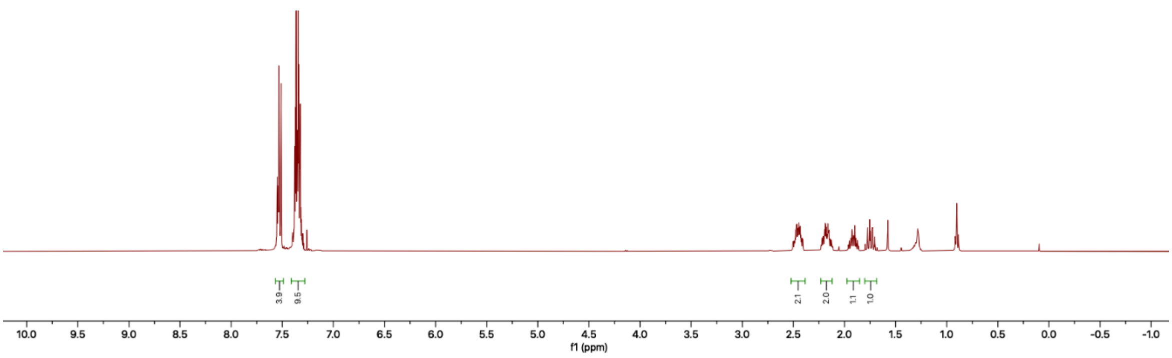

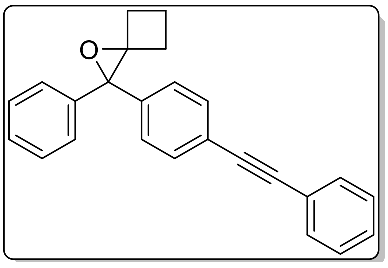

29

^{13}C {^{1}H} NMR (101 MHz, $CDCl_3$)

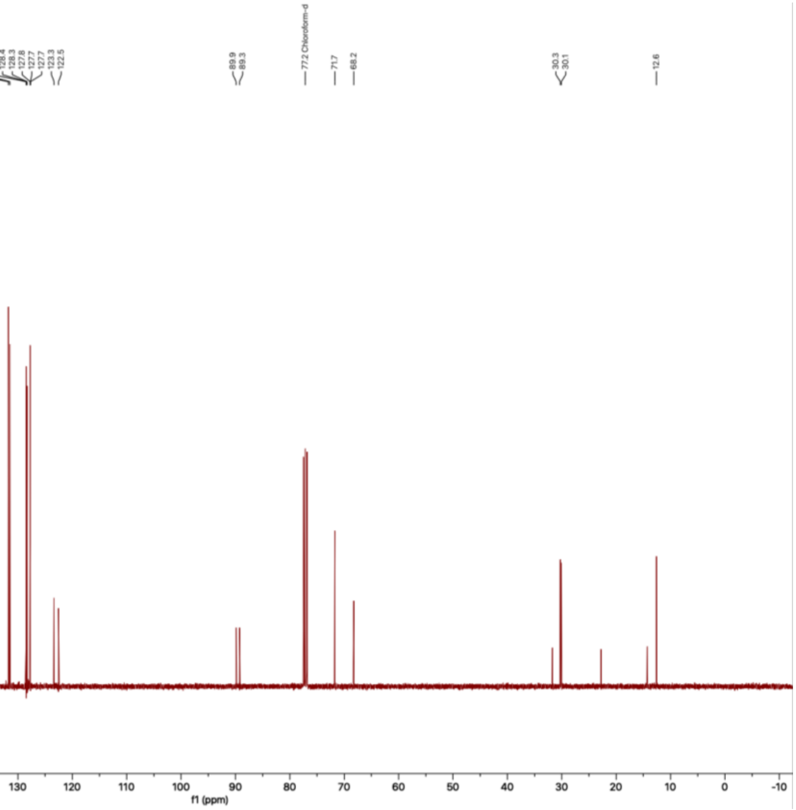

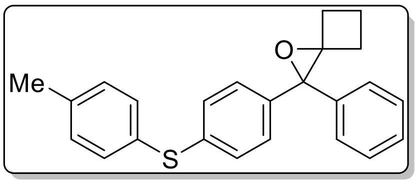

30

^{1}H NMR (400 MHz, $CDCl_3$)

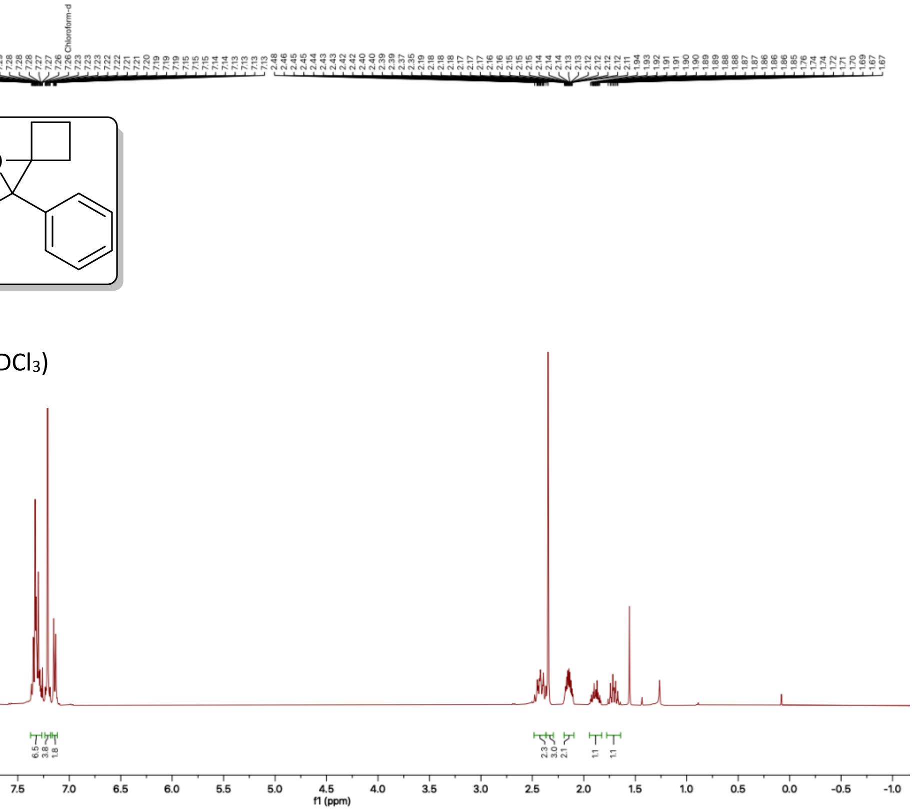

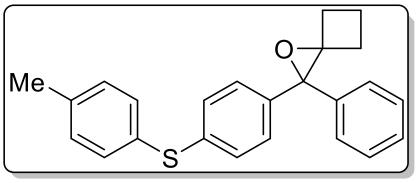

30

^{13}C {^{1}H} NMR (101 MHz, $CDCl_3$)

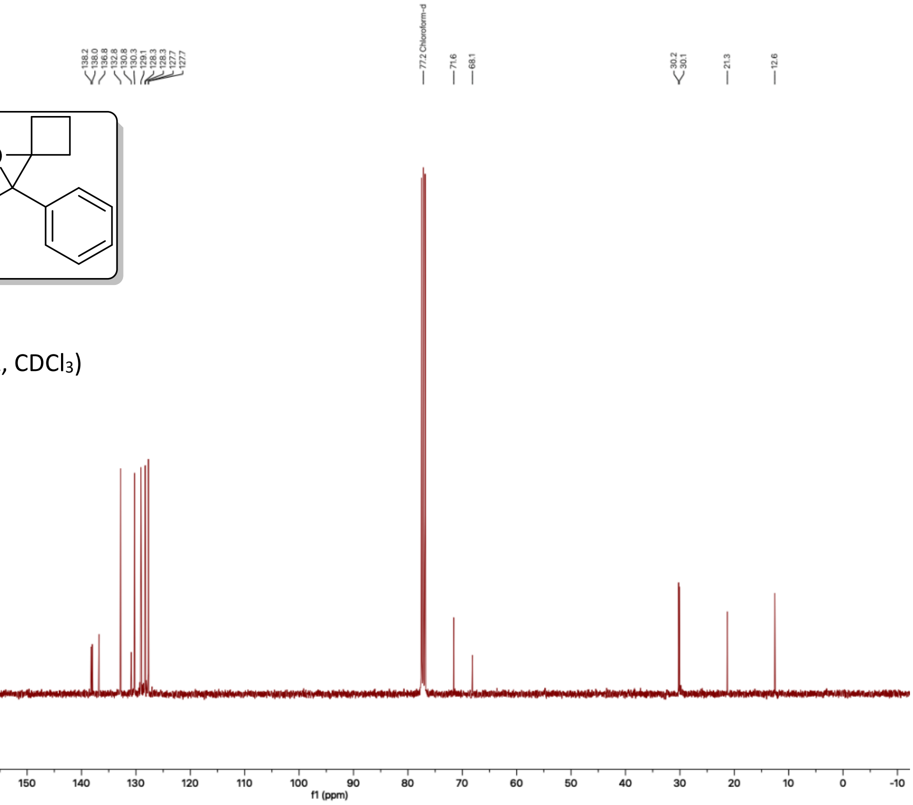

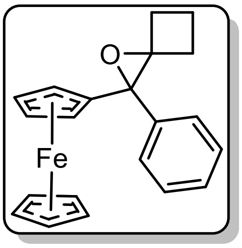

31 (crude)

^{1}H NMR (400 MHz, CDCl$_3$)

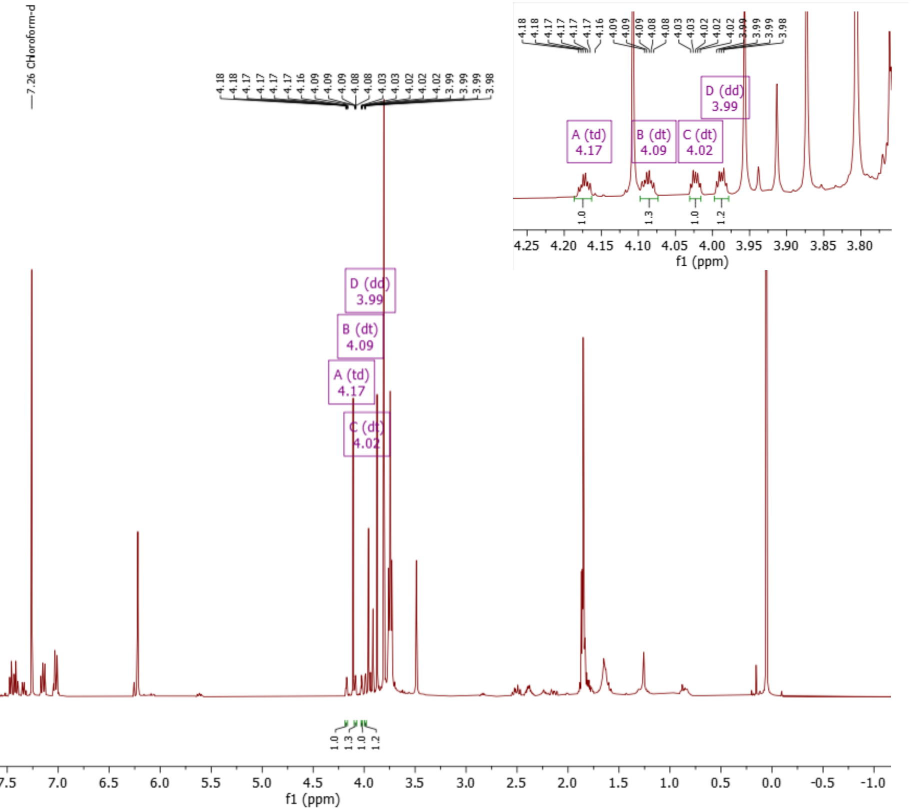

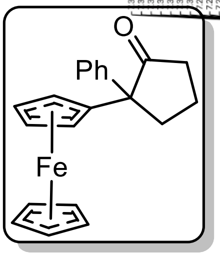

32

^{1}H NMR (400 MHz, CDCl$_3$)

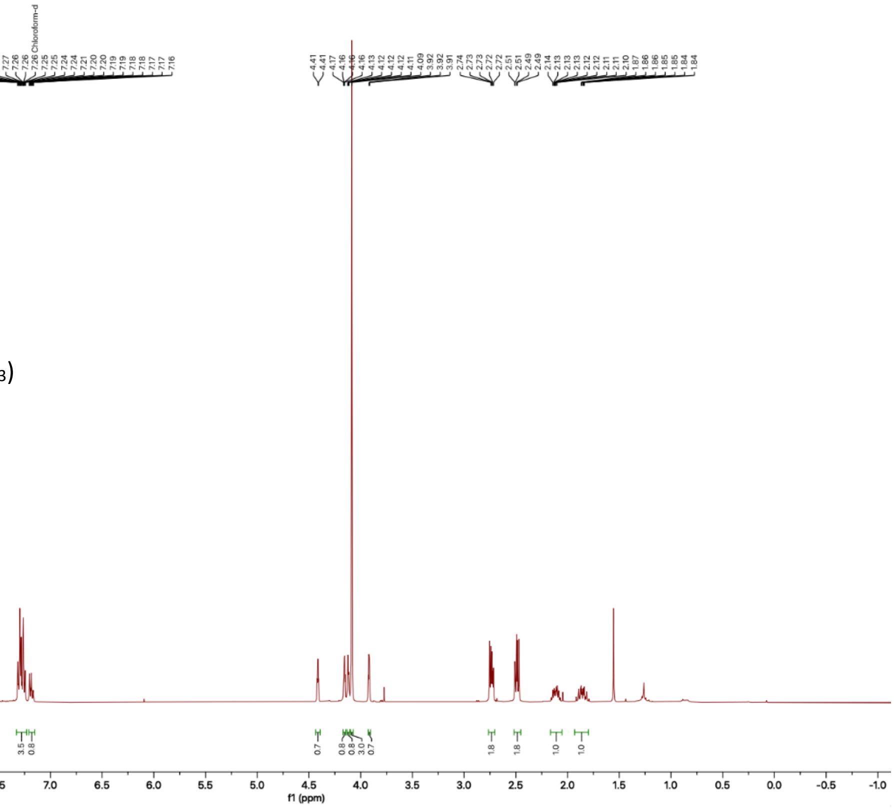

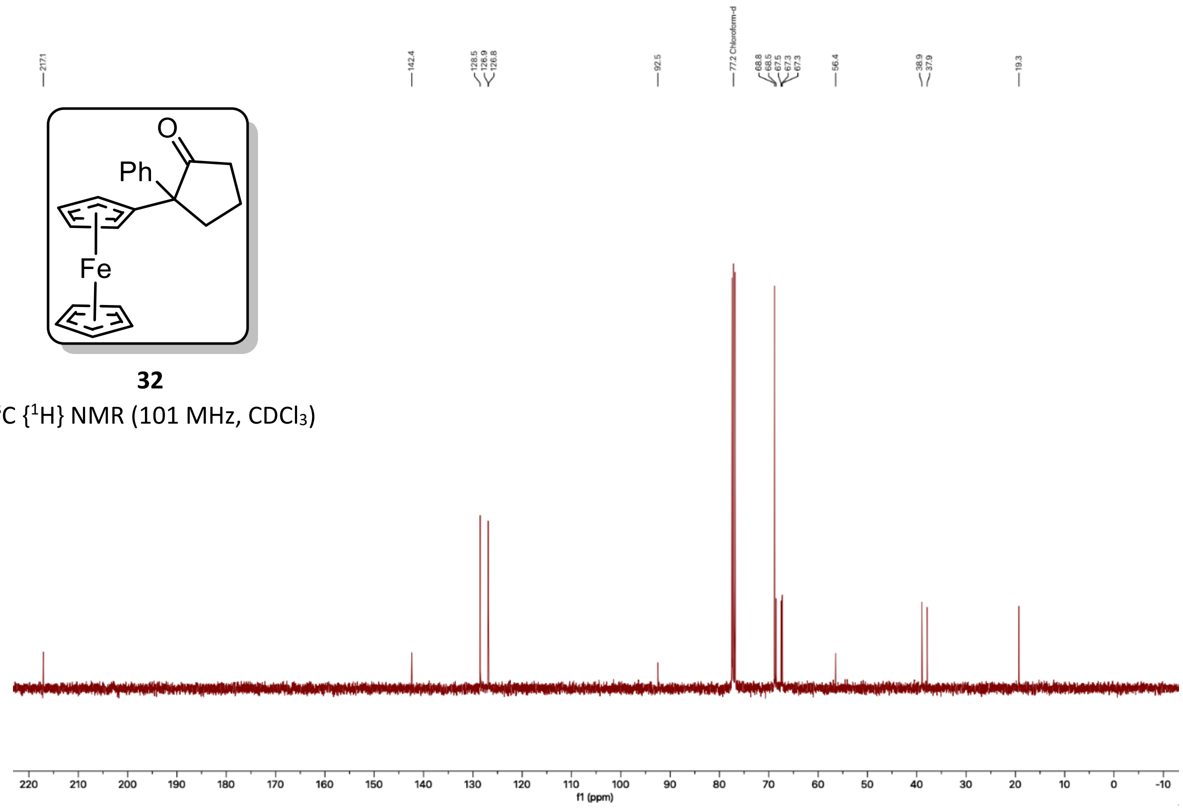

32

$^{13}C\ \{^{1}H\}$ NMR (101 MHz, $CDCl_3$)

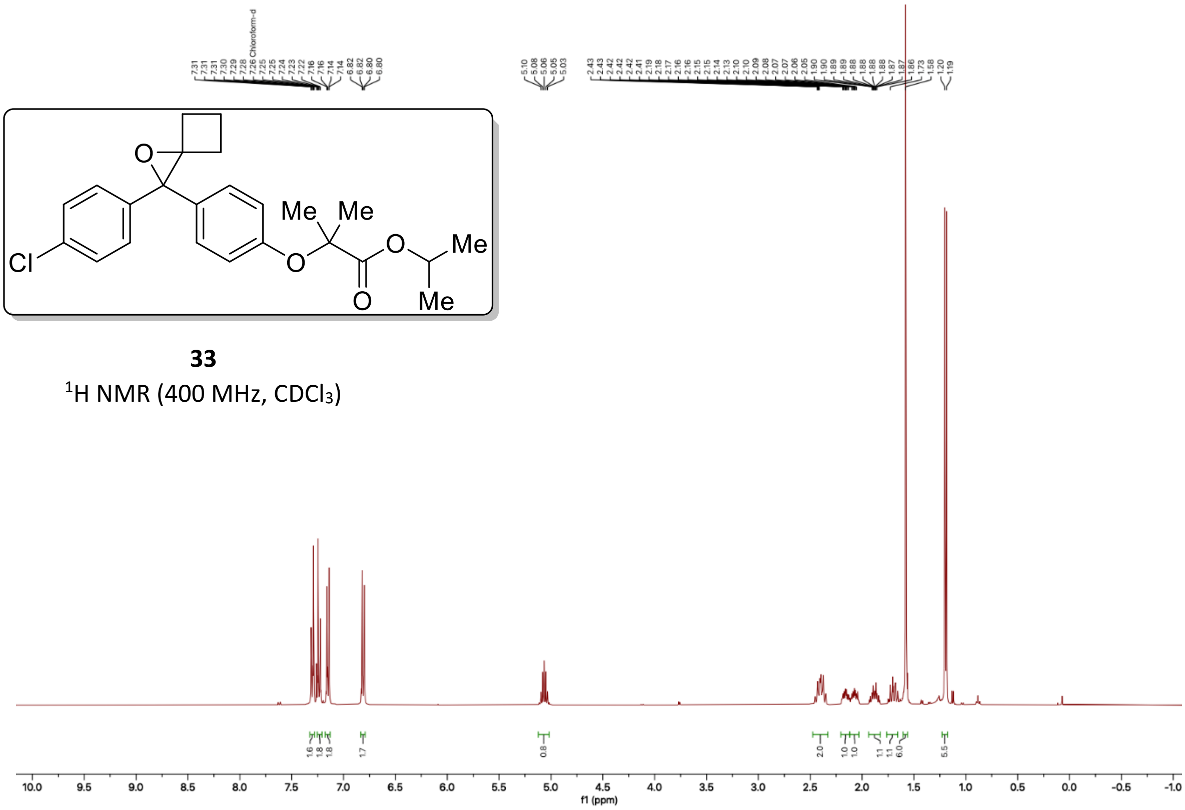

33
^{1}H NMR (400 MHz, $CDCl_3$)

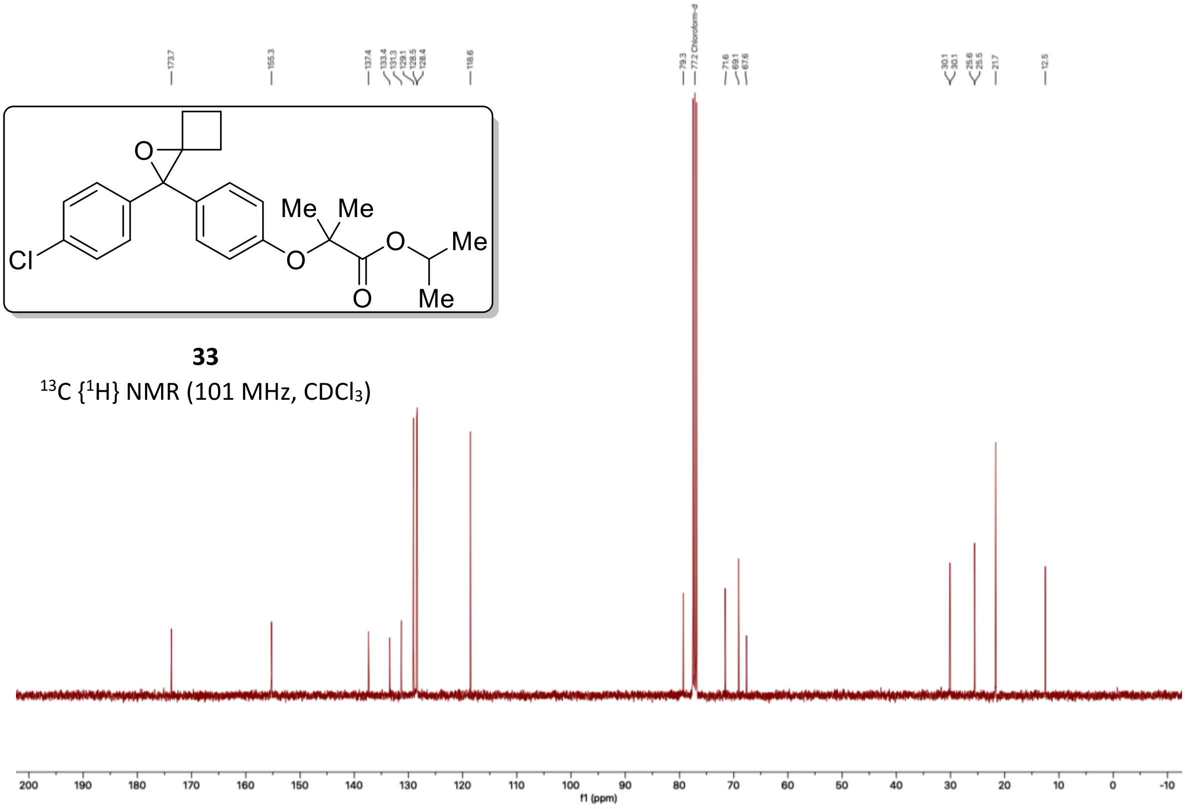

33
^{13}C {^{1}H} NMR (101 MHz, $CDCl_3$)

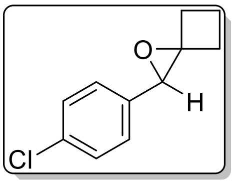

34

^{1}H NMR (400 MHz, $CDCl_3$)

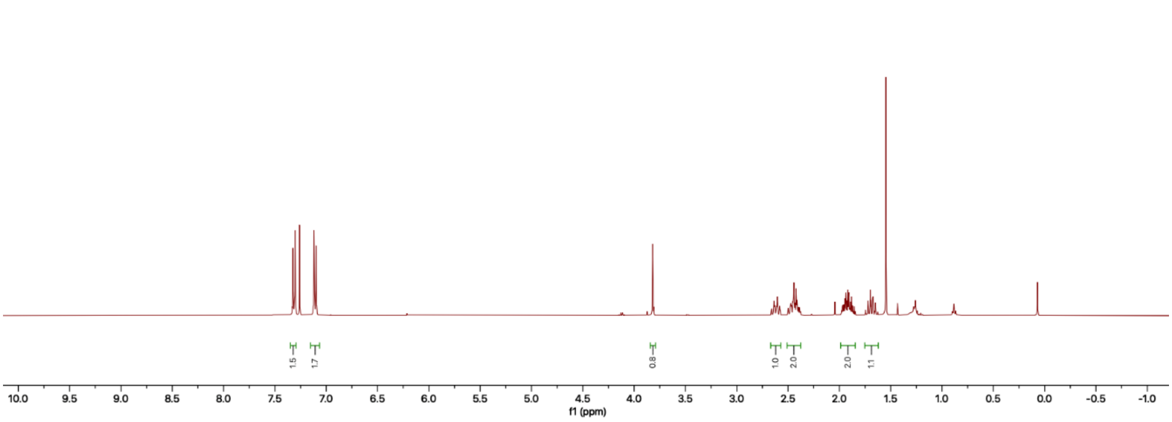

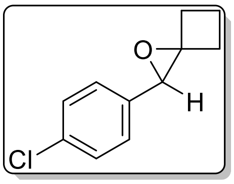

34

^{13}C {^{1}H} NMR (101 MHz, $CDCl_3$)

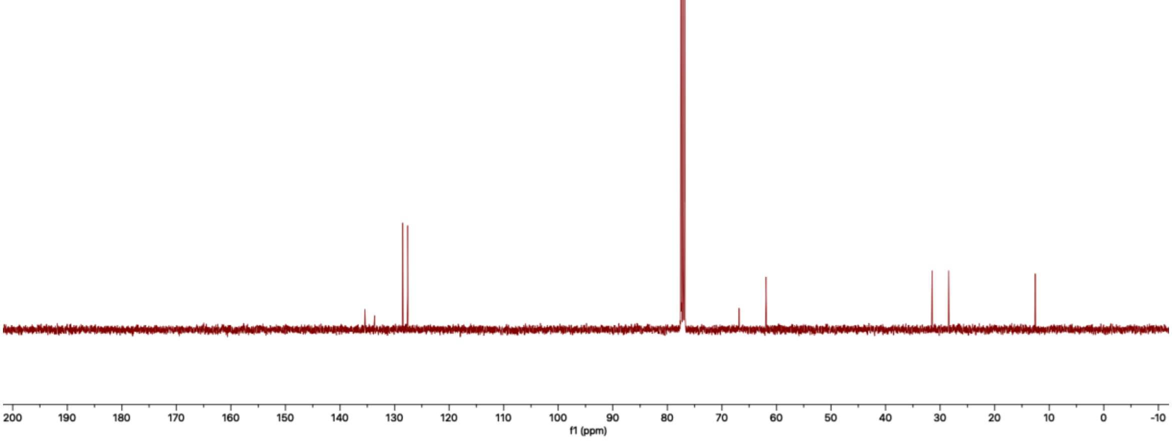

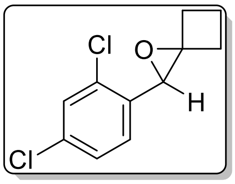

35

^{1}H NMR (400 MHz, $CDCl_3$)

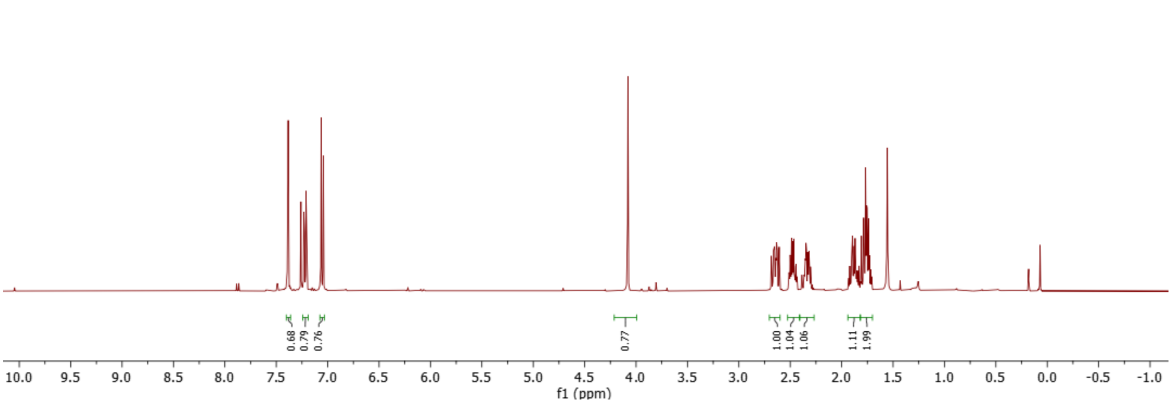

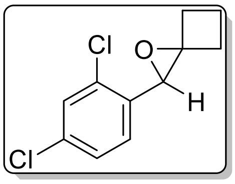

35

^{13}C {^{1}H} NMR (101 MHz, $CDCl_3$)

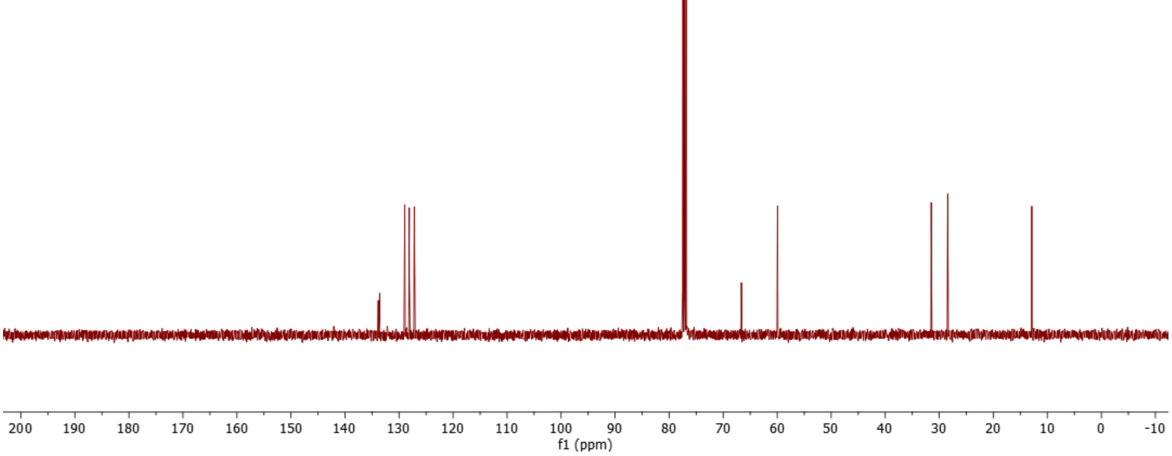

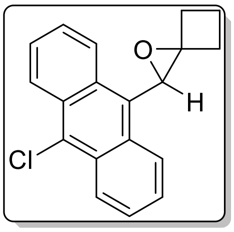

36

^{1}H NMR (400 MHz, $CDCl_3$)

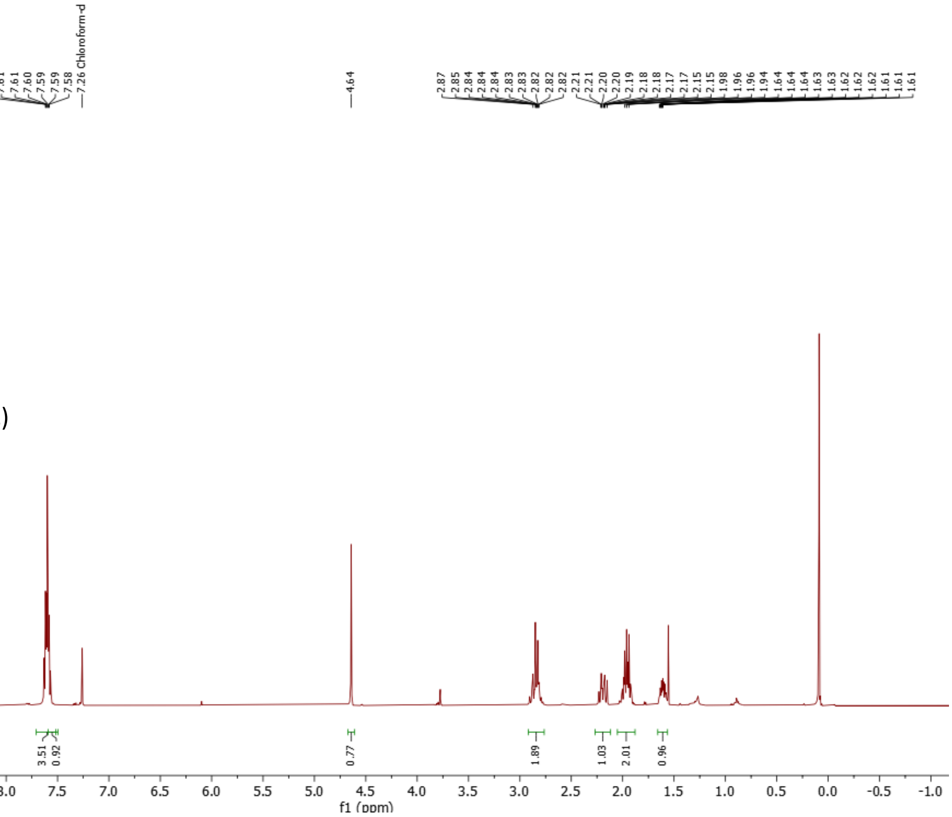

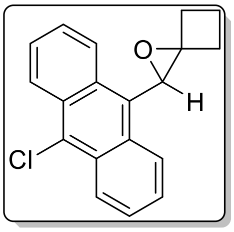

36

^{13}C {^{1}H} NMR (101 MHz, $CDCl_3$)

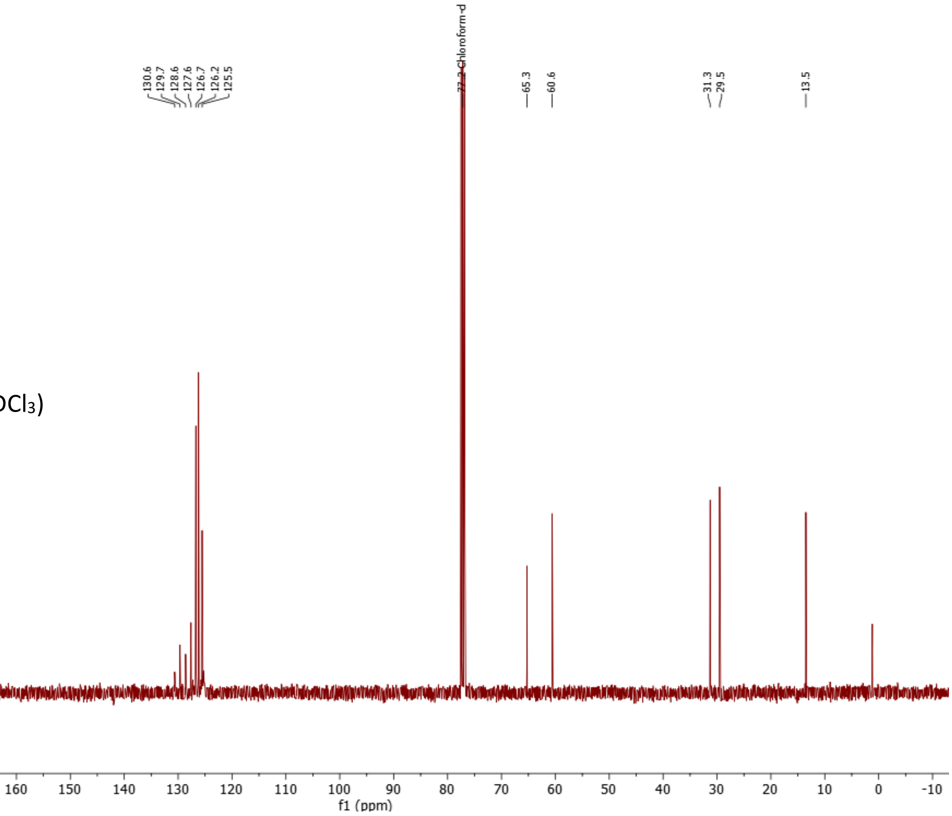

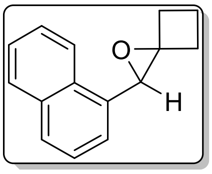

37

^{1}H NMR (400 MHz, $CDCl_3$)

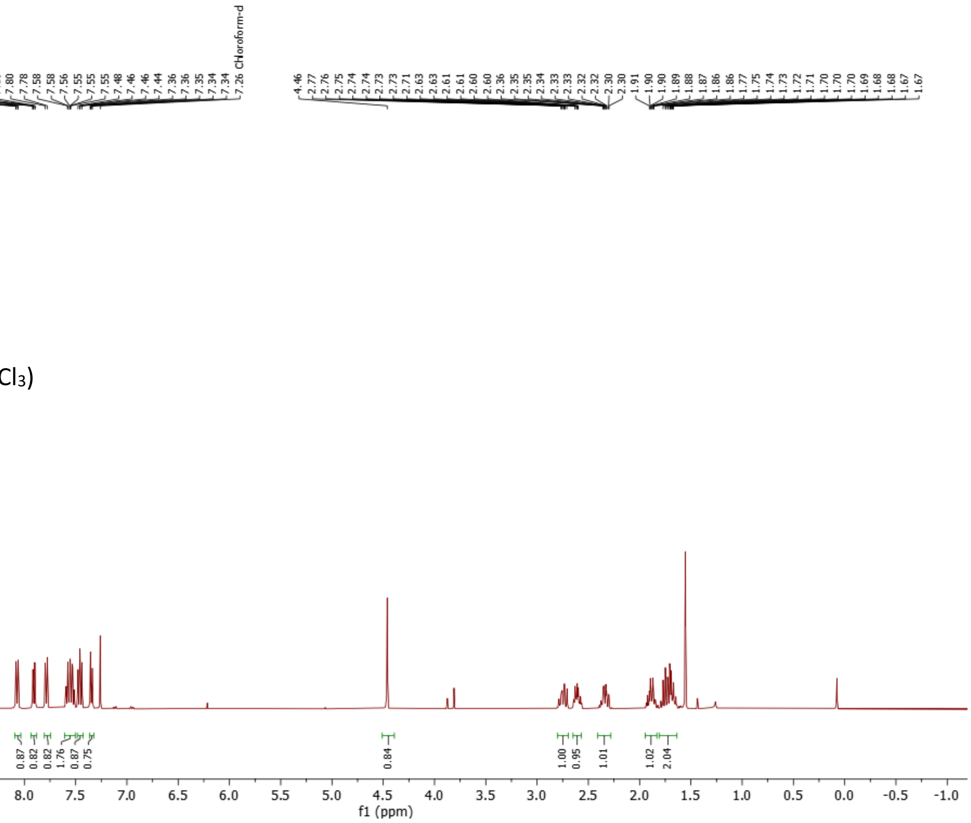

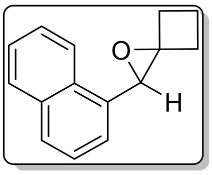

37

^{13}C {^{1}H} NMR (101 MHz, $CDCl_3$)

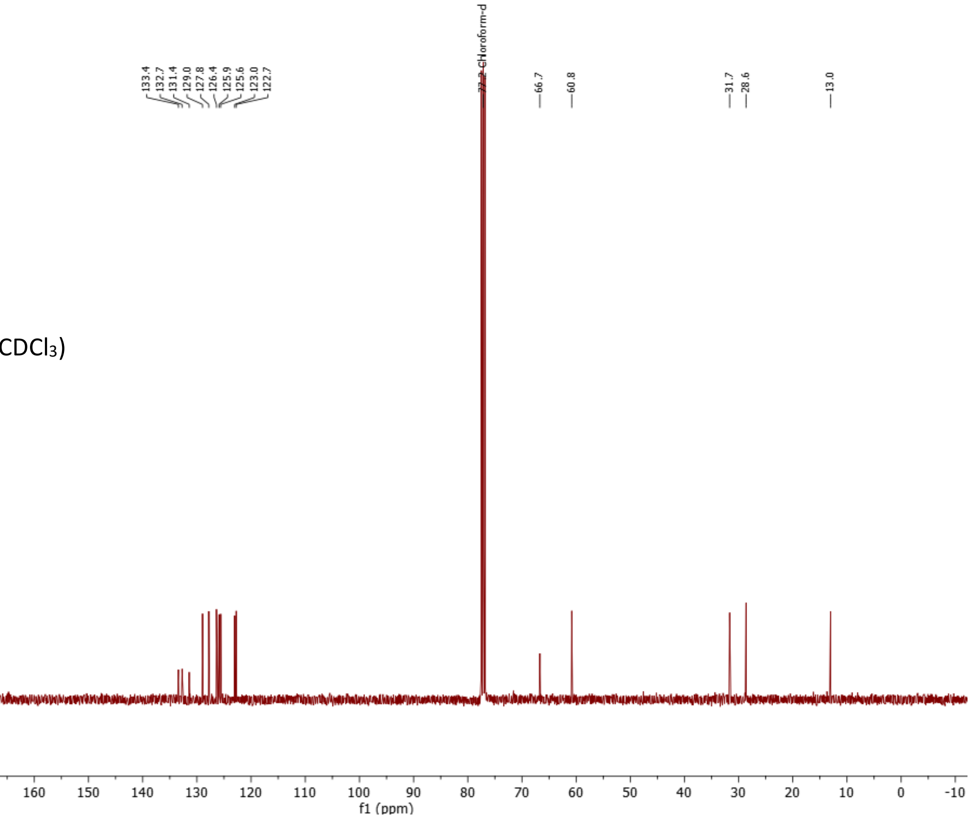

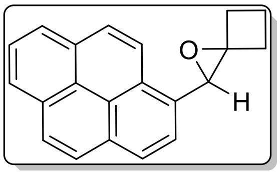

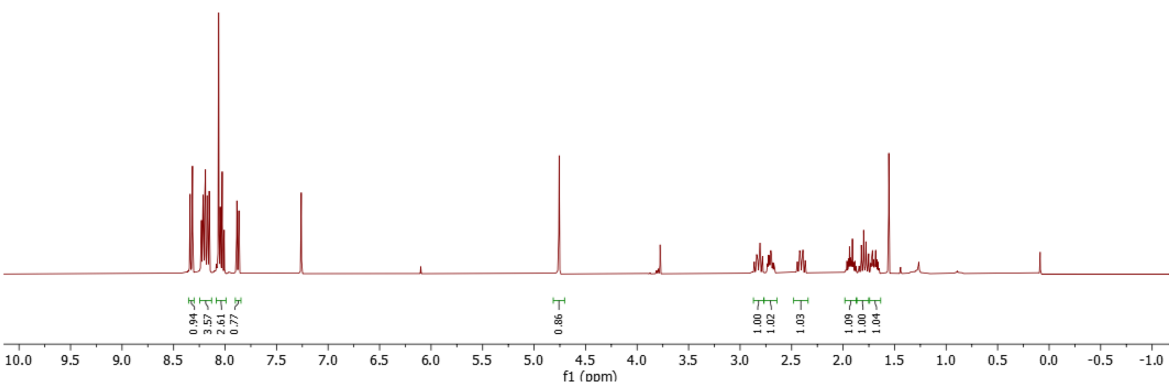

38

^{1}H NMR (400 MHz, $CDCl_3$)

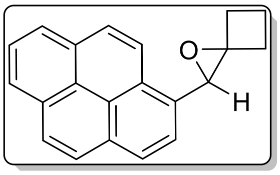

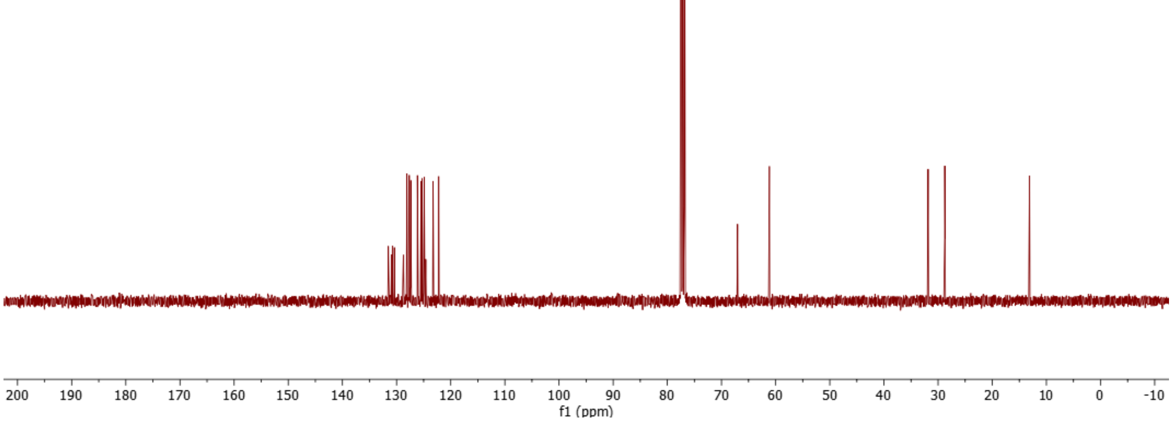

38

^{13}C {^{1}H} NMR (101 MHz, $CDCl_3$)

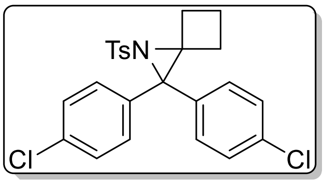

39

^{1}H NMR (400 MHz, $CDCl_3$)

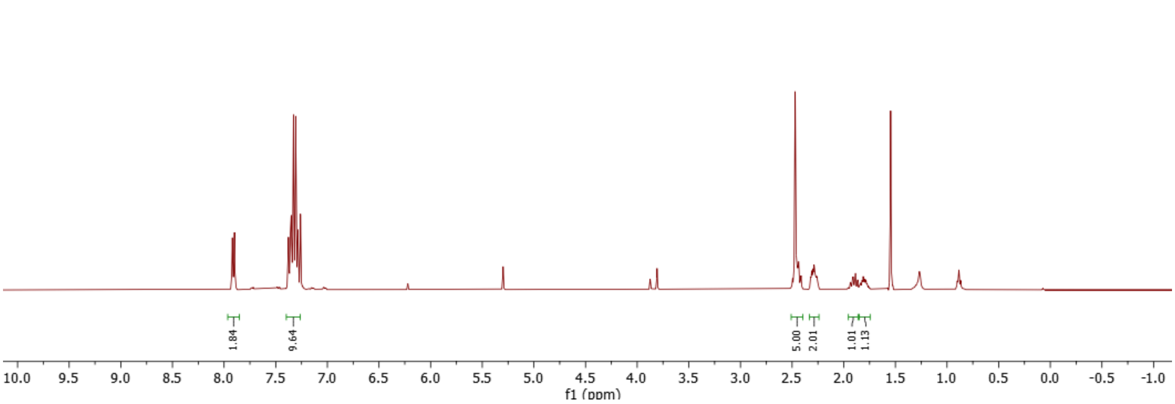

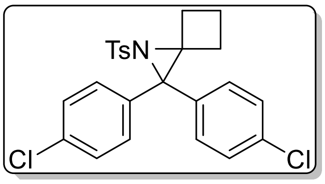

39

^{13}C {^{1}H} NMR (101 MHz, $CDCl_3$)

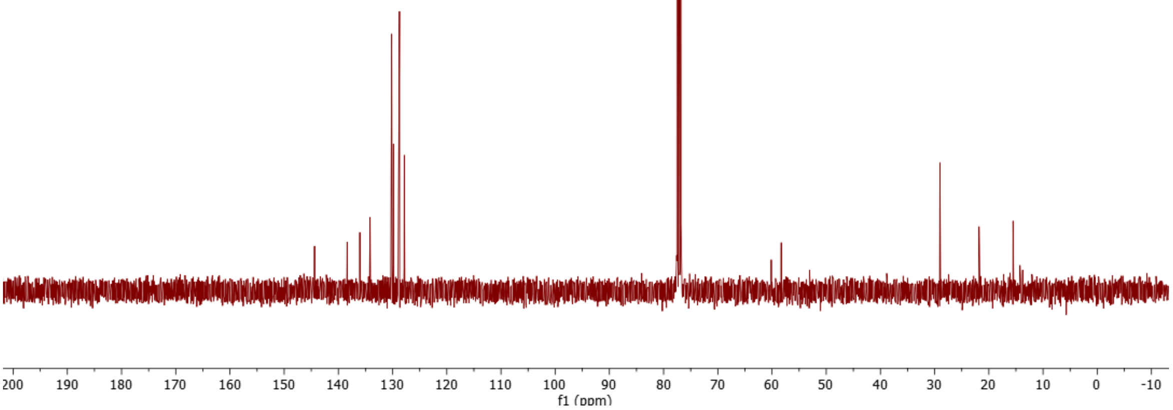

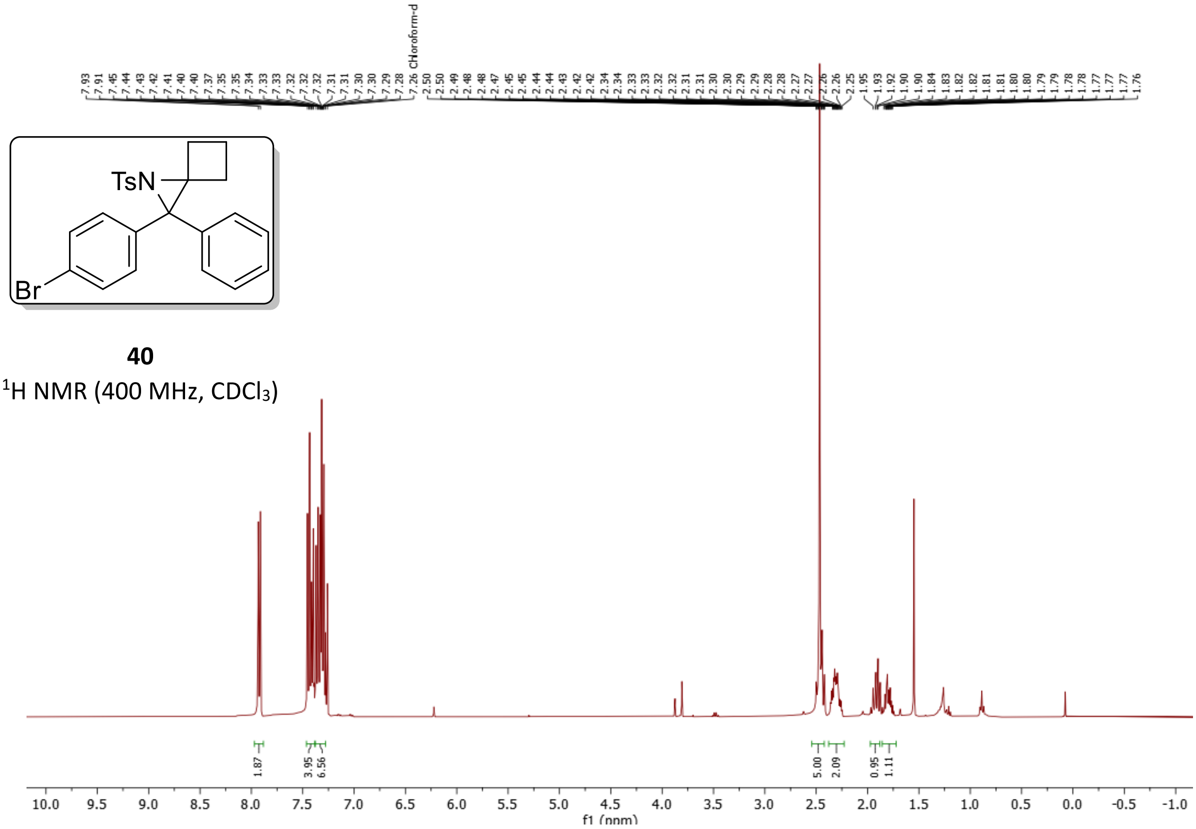

40

^{1}H NMR (400 MHz, $CDCl_3$)

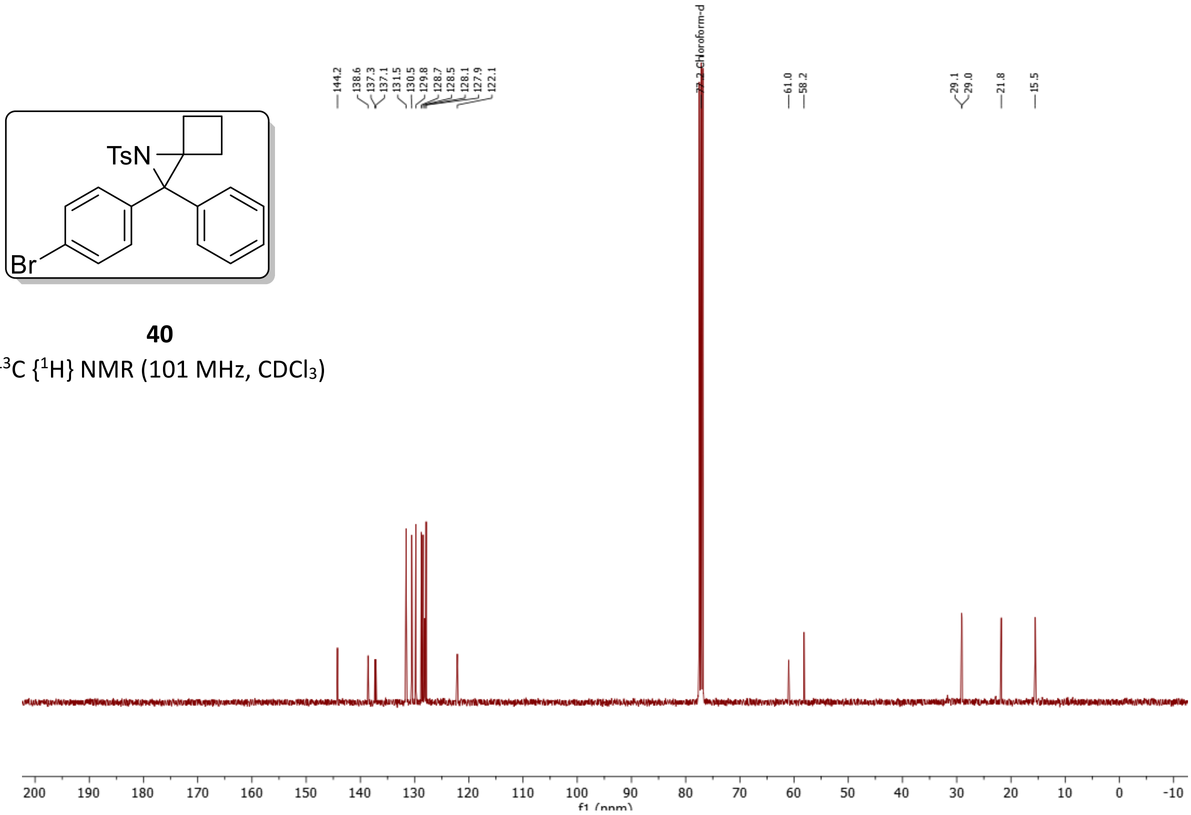

40

$^{13}C\ \{^{1}H\}$ NMR (101 MHz, $CDCl_3$)

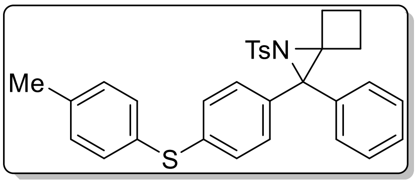

41

^{1}H NMR (400 MHz, CDCl$_3$)

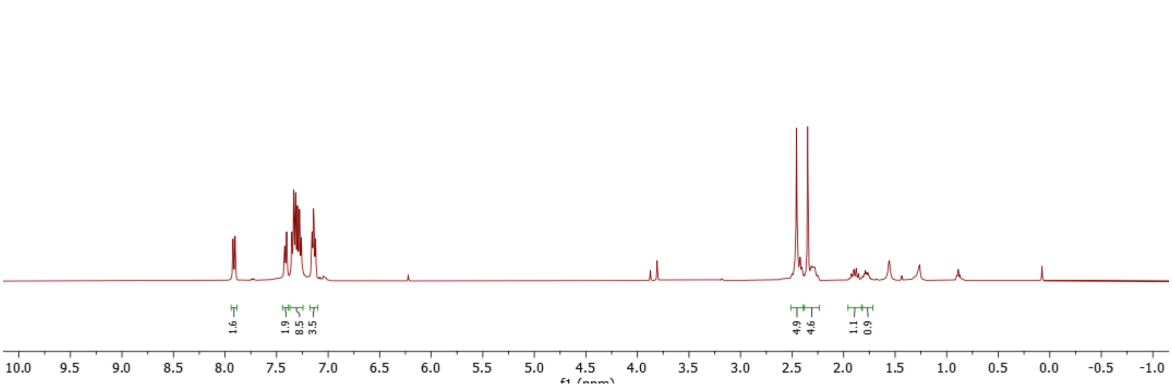

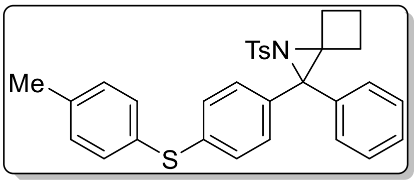

41

^{13}C {^{1}H} NMR (101 MHz, CDCl$_3$)

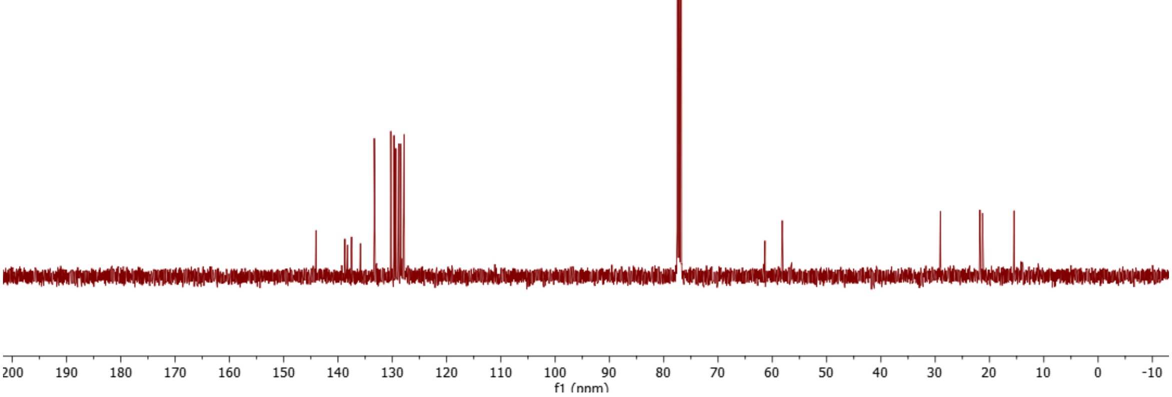

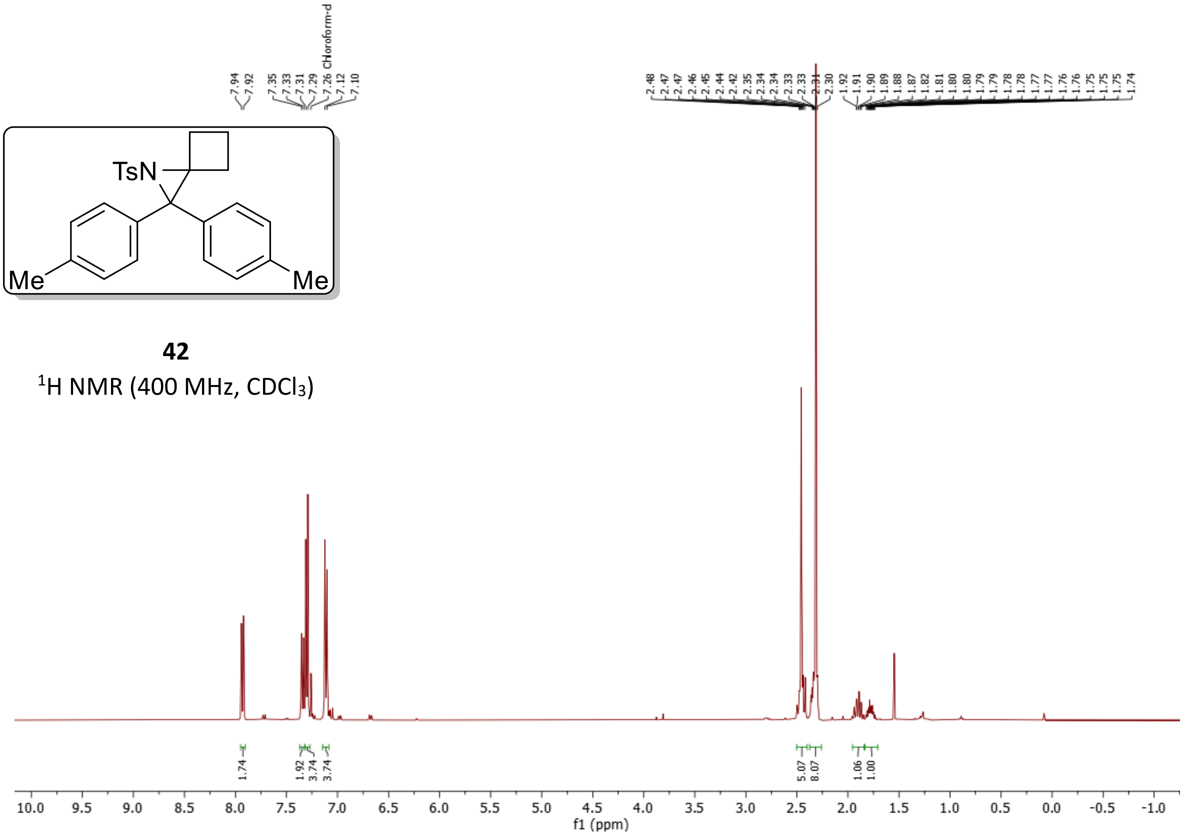

42

^{1}H NMR (400 MHz, $CDCl_3$)

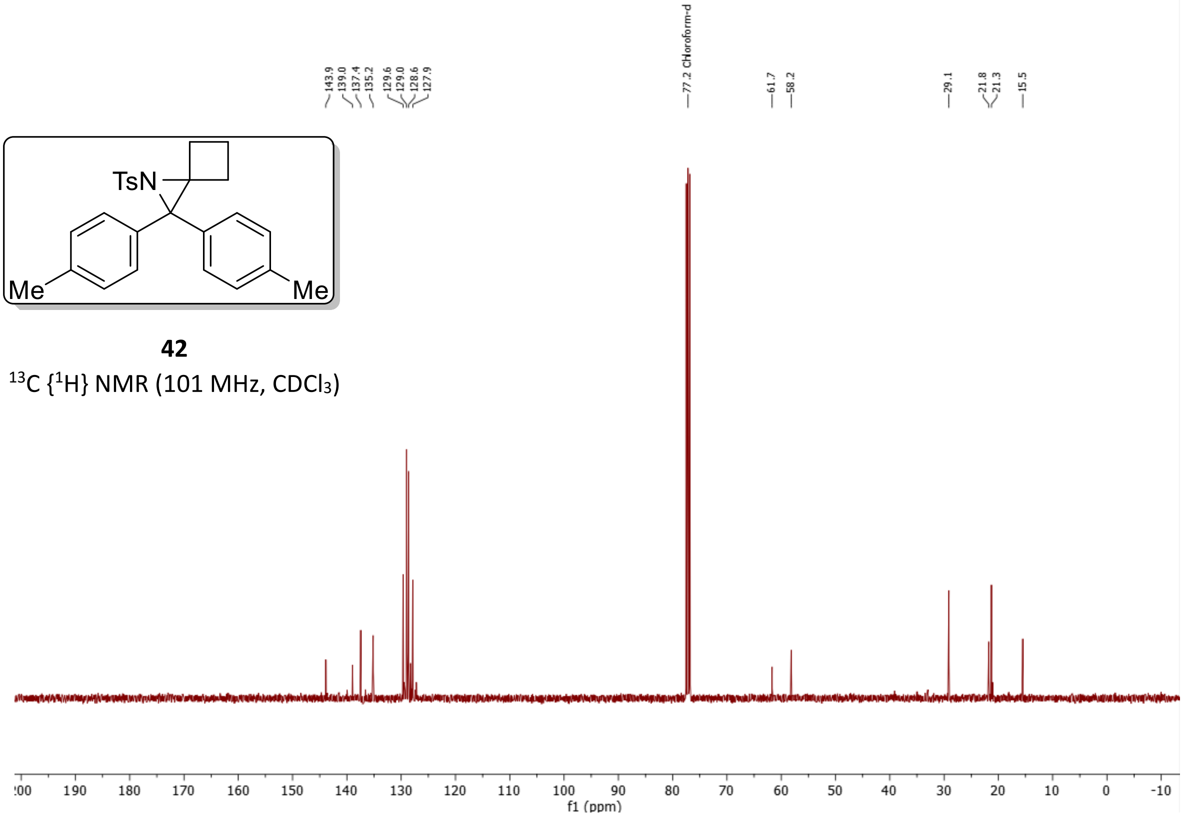

42

^{13}C {^{1}H} NMR (101 MHz, $CDCl_3$)

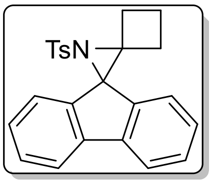

43

^{1}H NMR (400 MHz, $CDCl_3$)

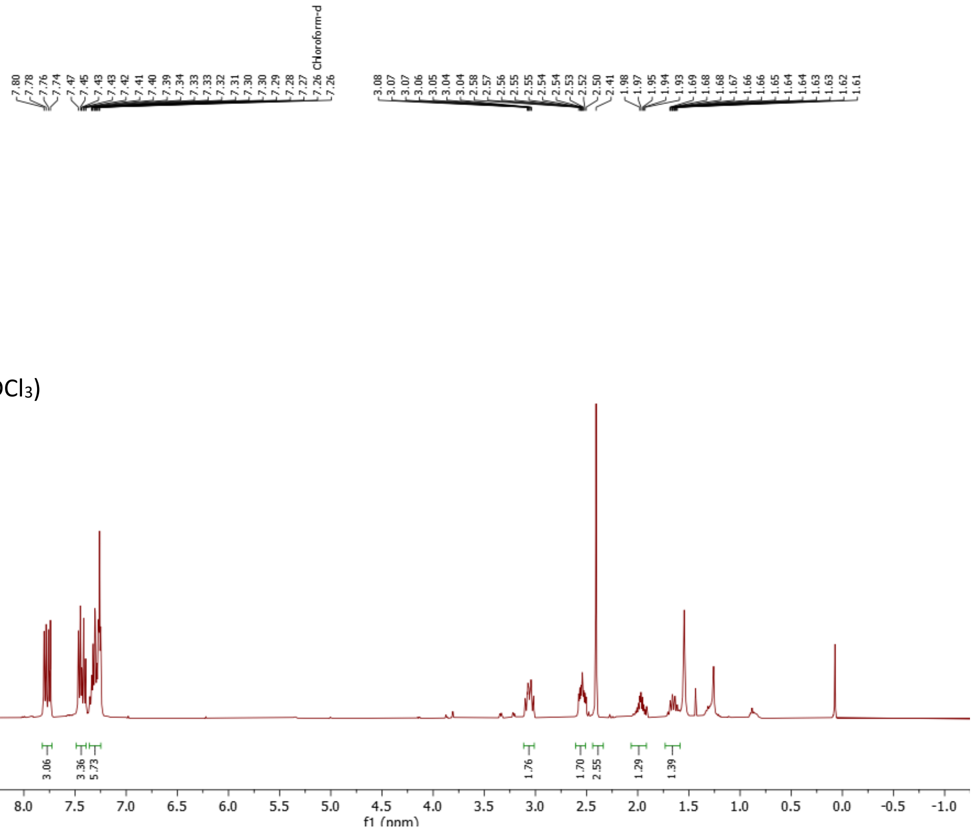

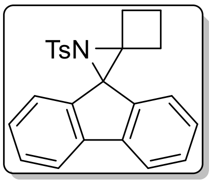

43

^{13}C {^{1}H} NMR (101 MHz, $CDCl_3$)

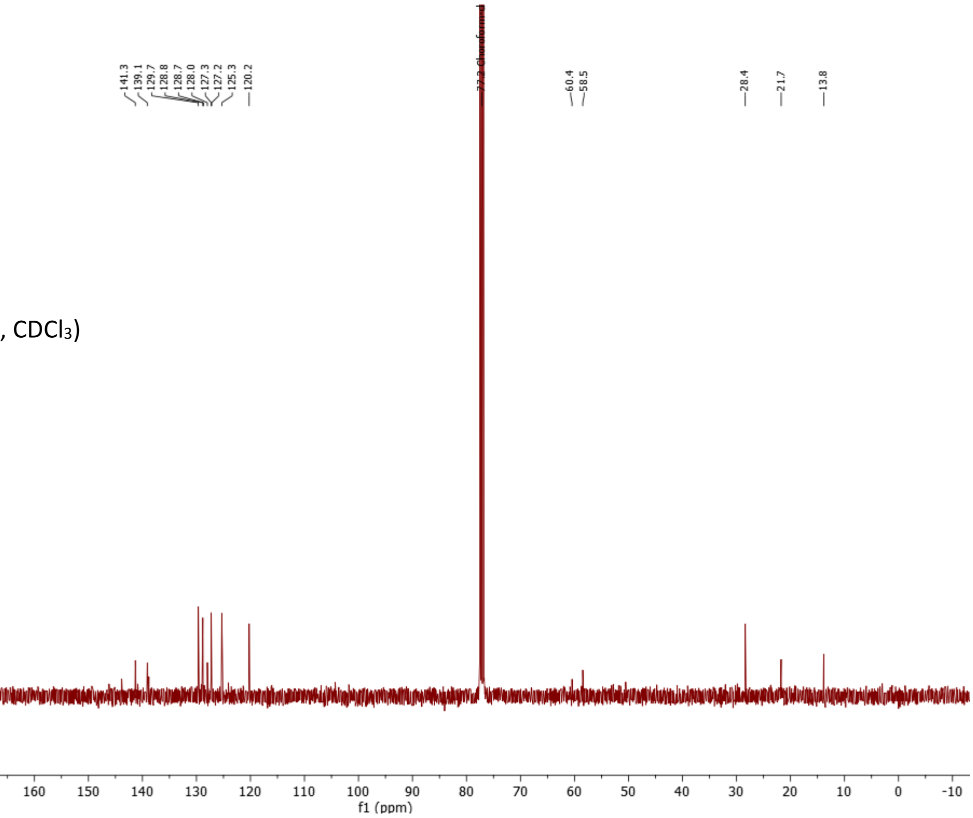

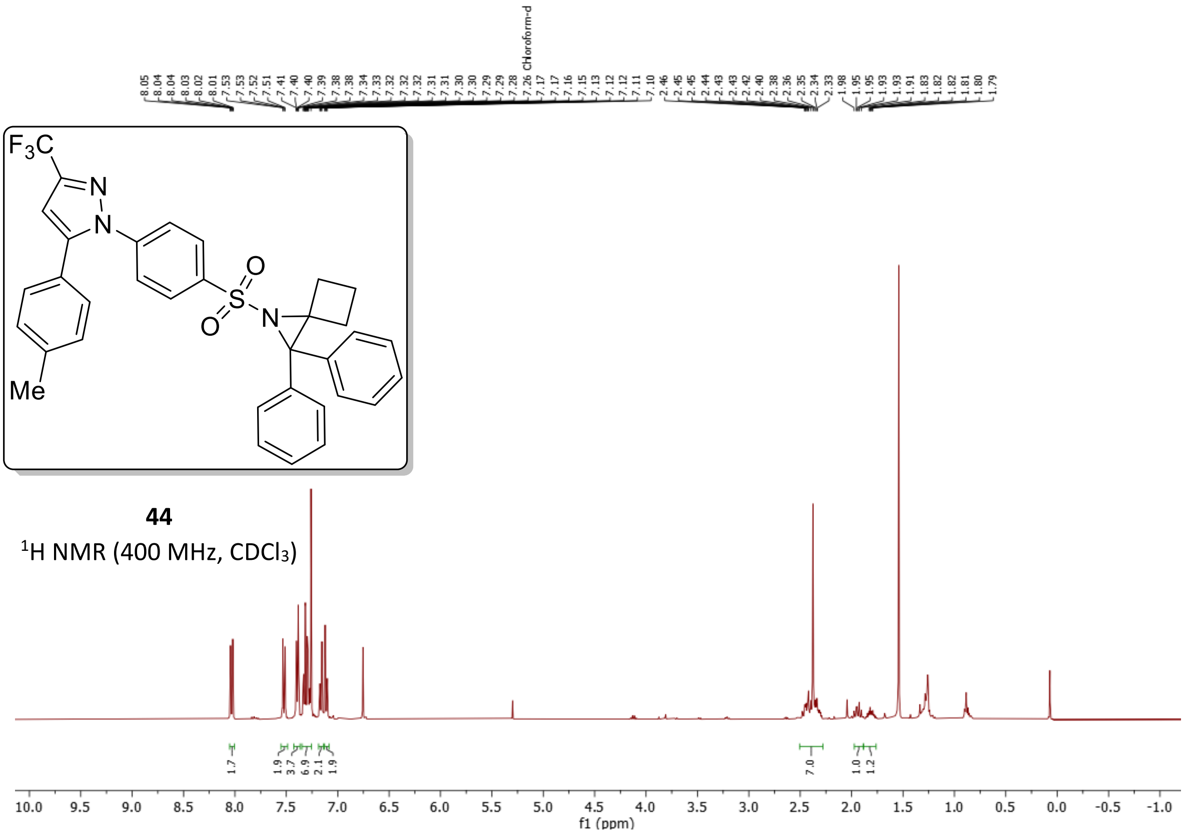
F3C
N
N
O
S
O
N
Me
44
1H NMR (400 MHz, CDCl3)
Chloroform-d
f1 (ppm)

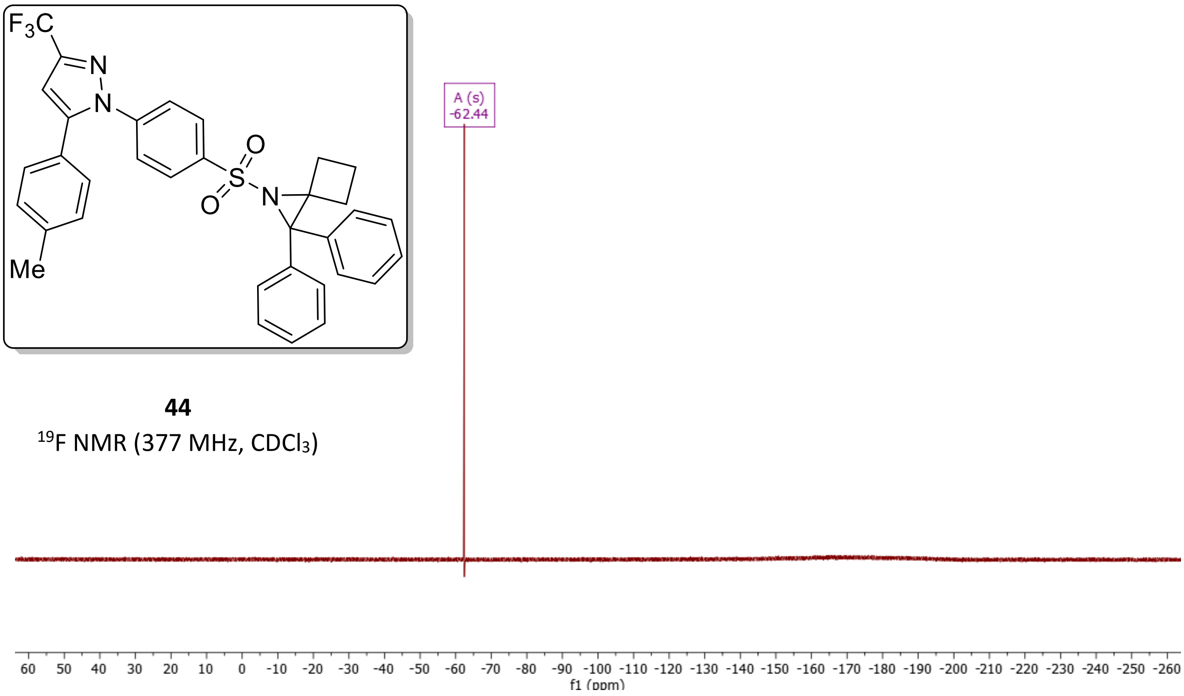
F3C
N
N
O
S
O
N
Me
44
19F NMR (377 MHz, CDCl3)
A (s)
-62.44
f1 (ppm)

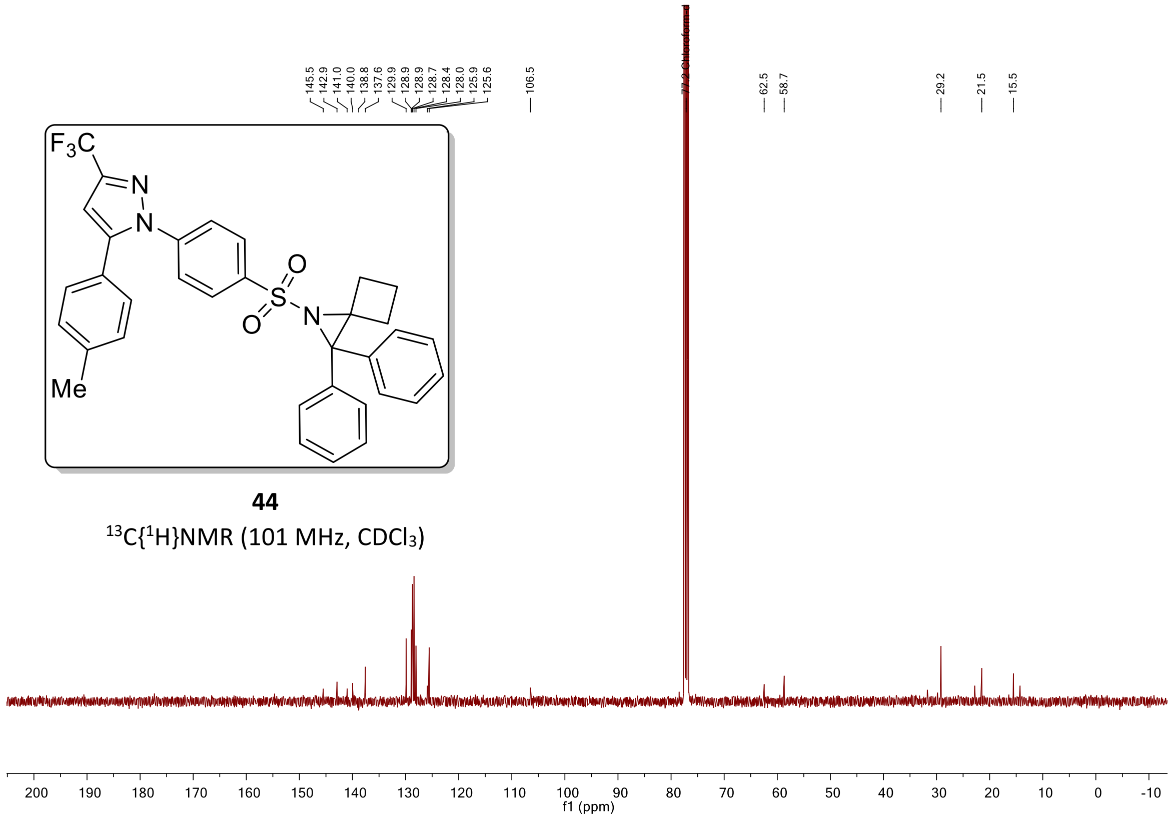
145.5
142.9
141.0
140.0
138.8
137.6
129.9
128.9
128.9
128.7
128.4
128.0
125.9
125.6
106.5
77.2 Chloroform-d
62.5
58.7
29.2
21.5
15.5
F3C
N
N
S
O
O
N
Me
44
13C{1H}NMR (101 MHz, CDCl3)
200 190 180 170 160 150 140 130 120 110 100 90 80 70 60 50 40 30 20 10 0 -10
f1 (ppm)

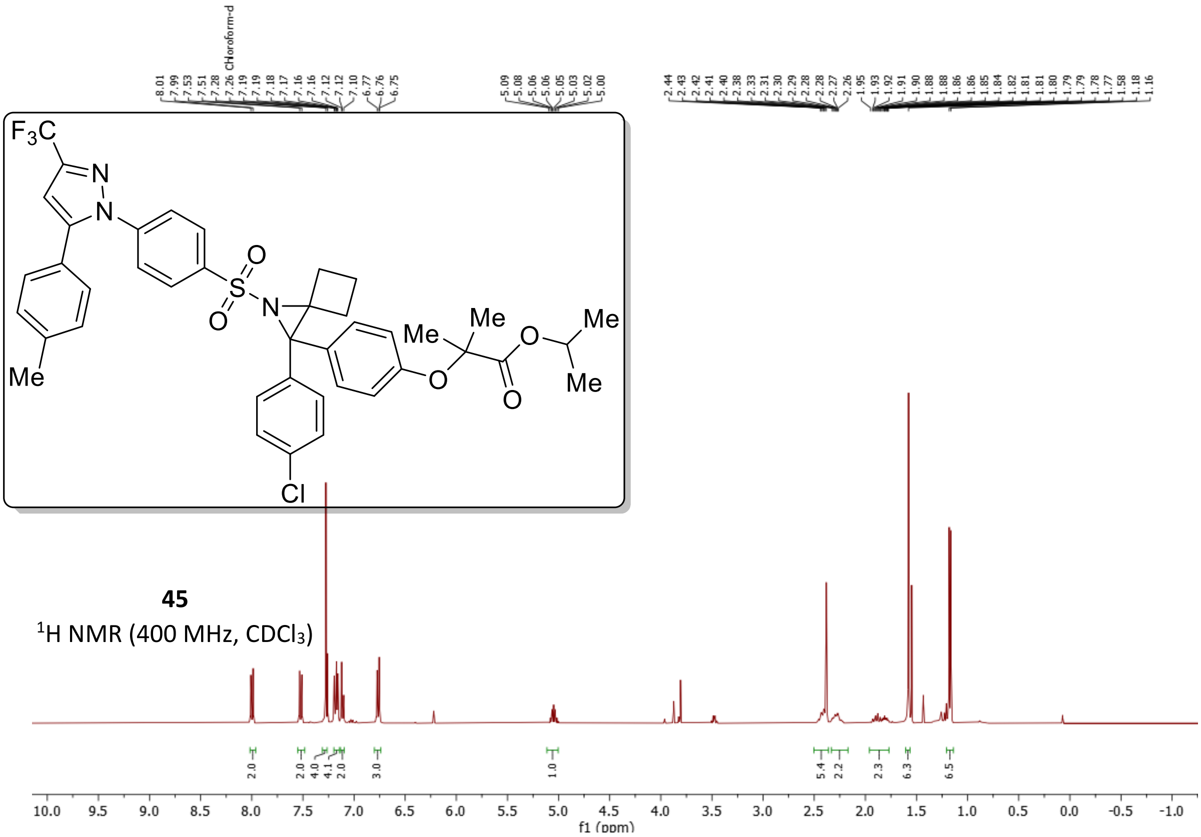

45

^{1}H NMR (400 MHz, $CDCl_3$)

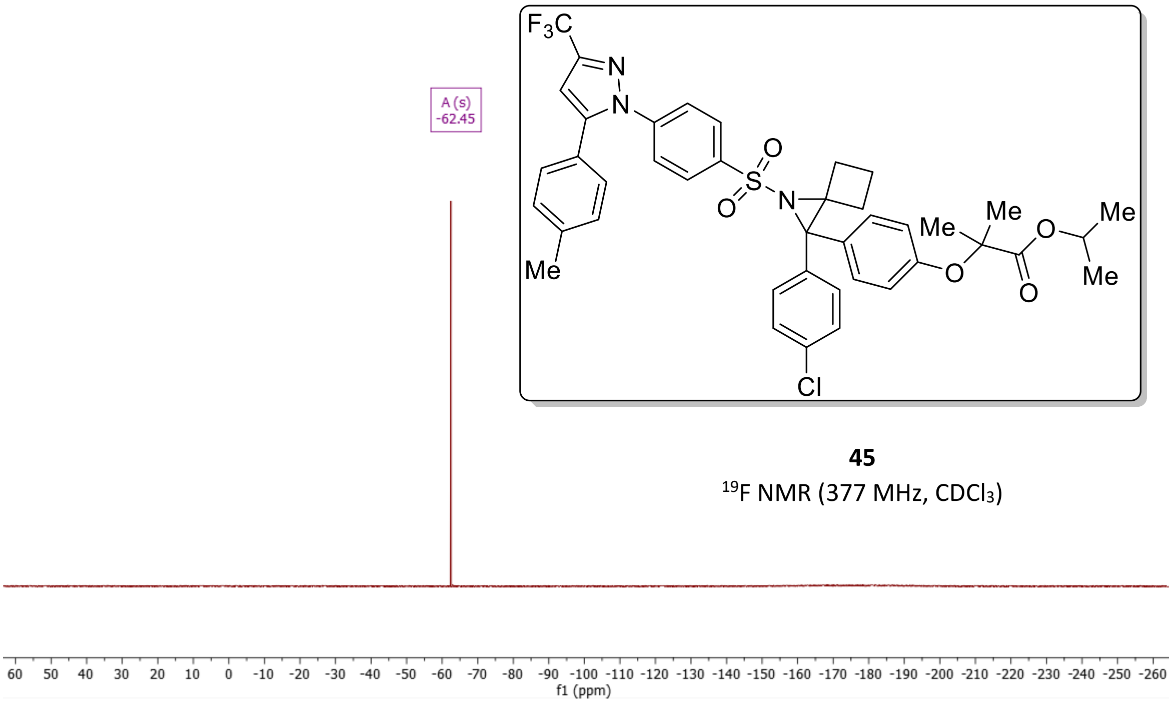

45

^{19}F NMR (377 MHz, $CDCl_3$)

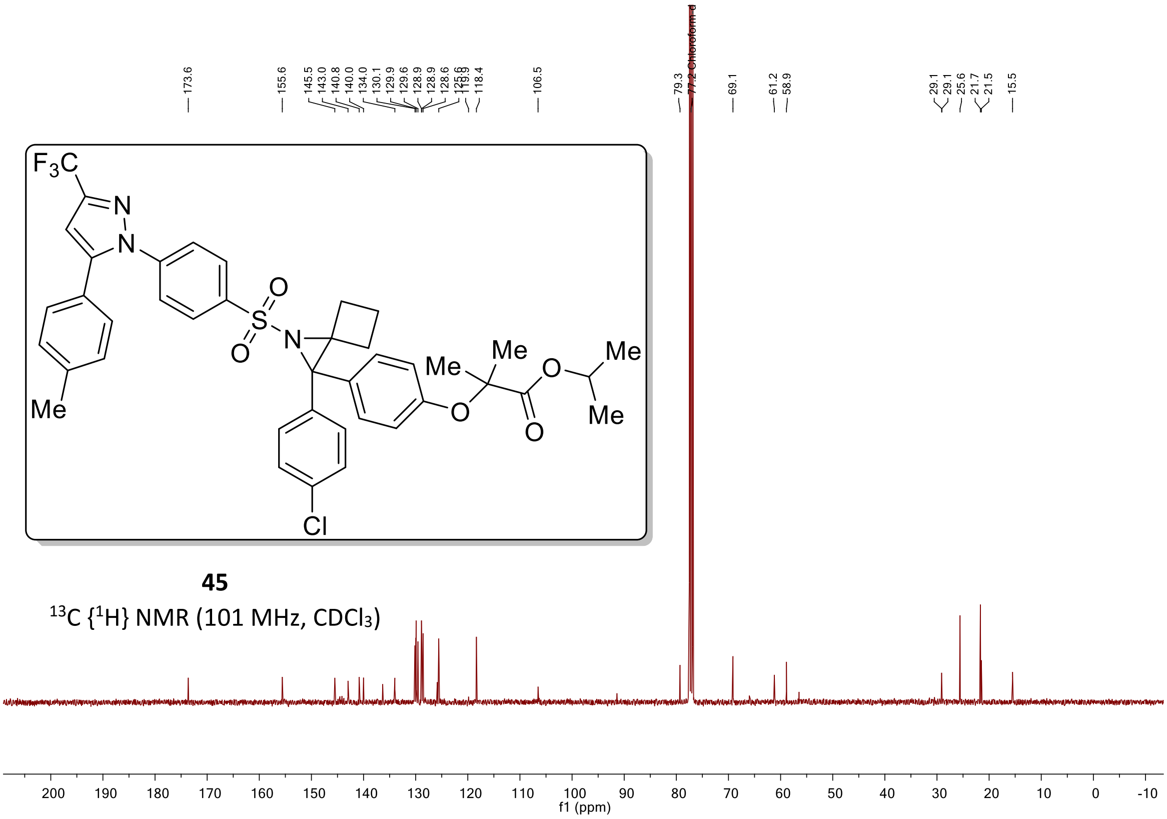

45

$^{13}C\{^1H\}$ NMR (101 MHz, $CDCl_3$)

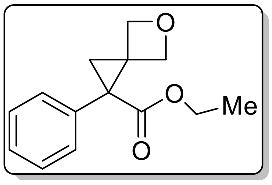

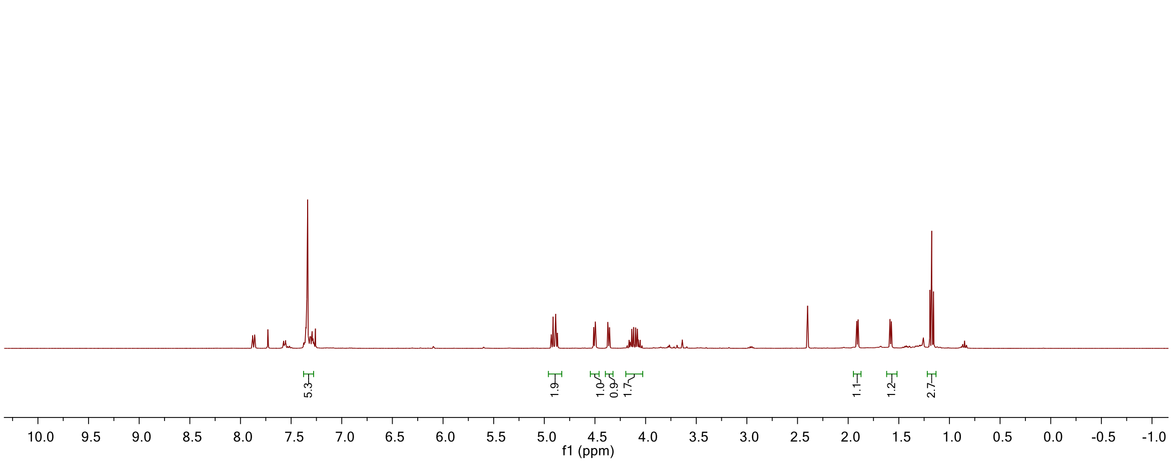

46

^{1}H NMR (400 MHz, $CDCl_3$)

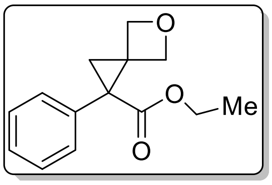

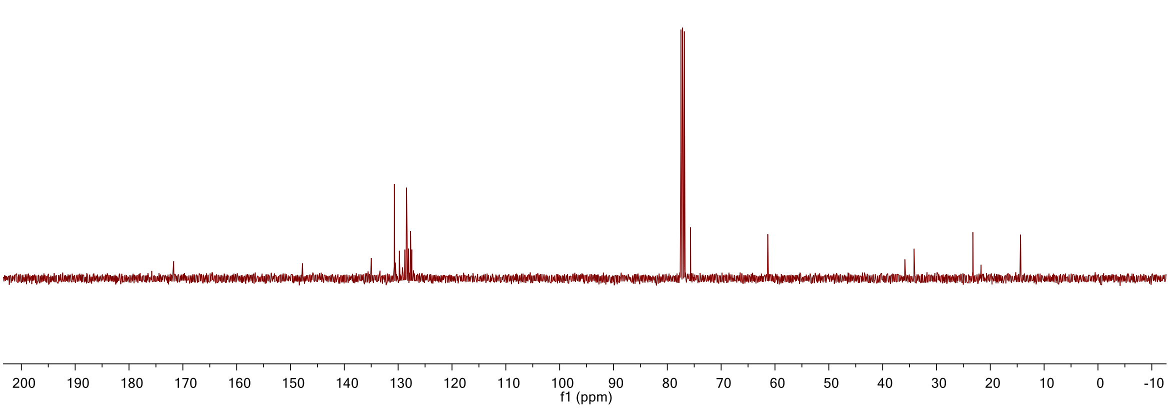

46

^{13}C {^{1}H} NMR (101 MHz, $CDCl_3$)

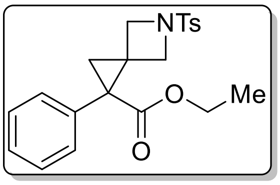

47

^{1}H NMR (400 MHz, $CDCl_3$)

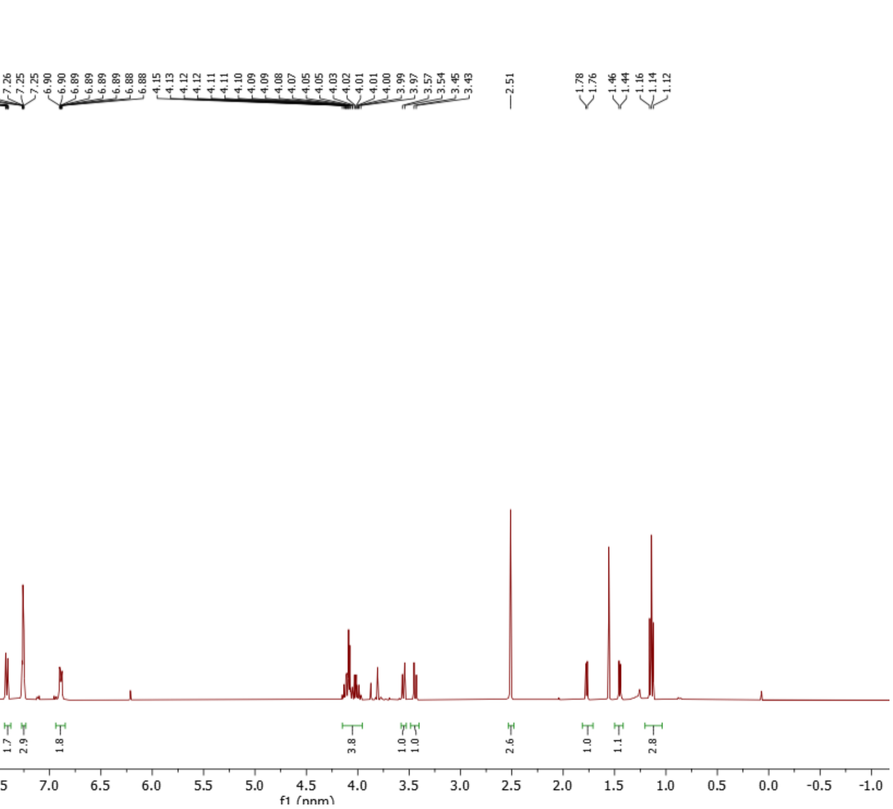

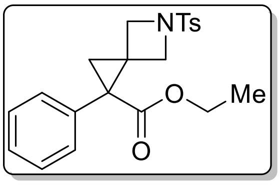

47

^{13}C {^{1}H} NMR (101 MHz, $CDCl_3$)

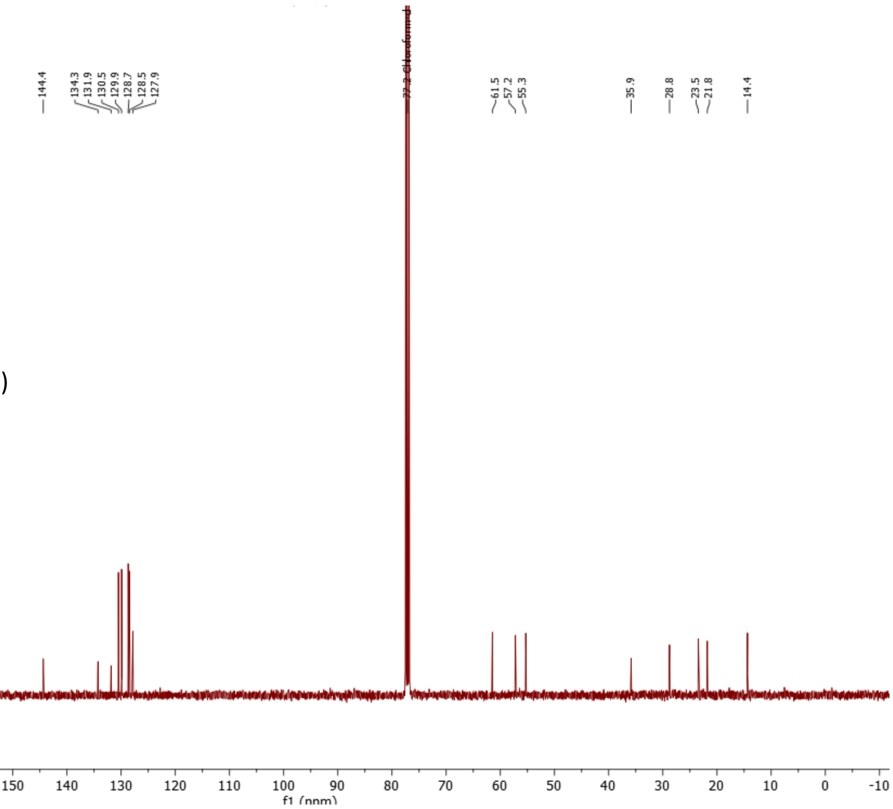

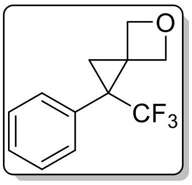

48

^{1}H NMR (400 MHz, $CDCl_3$)

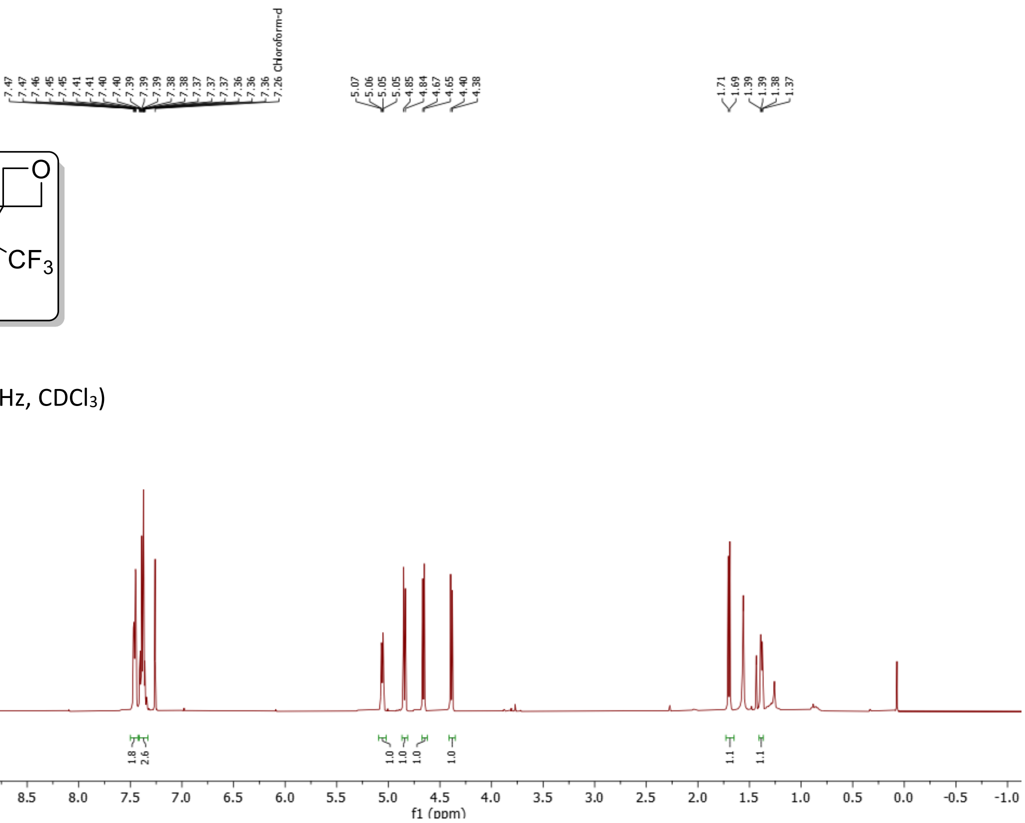

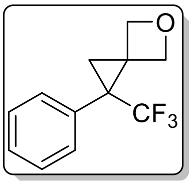

48

^{19}F NMR (377 MHz, $CDCl_3$)

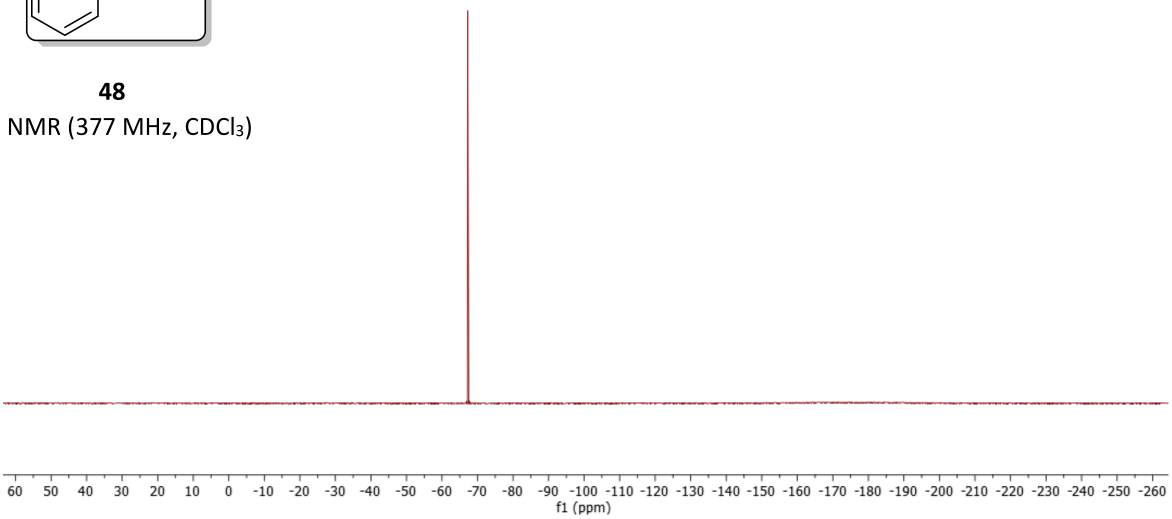

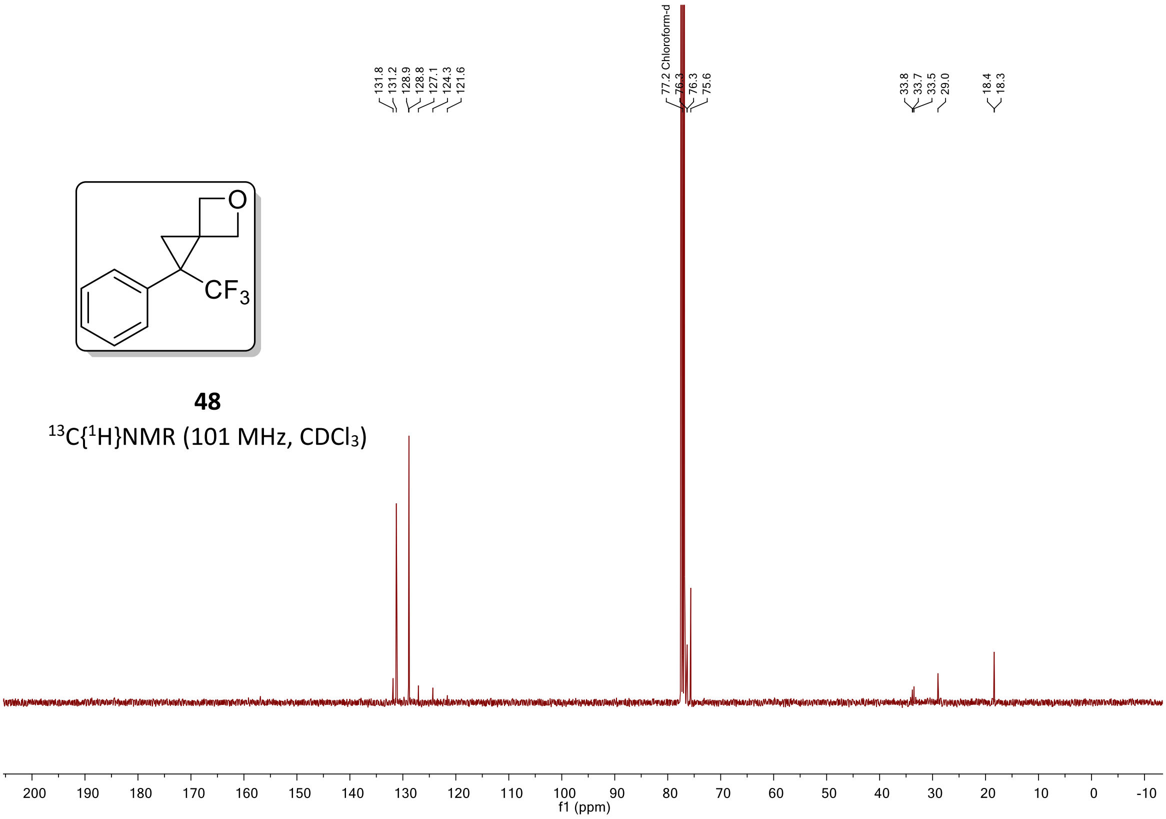

48

$^{13}C\{^1H\}$NMR (101 MHz, $CDCl_3$)

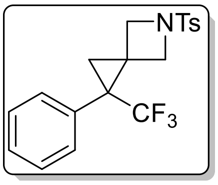

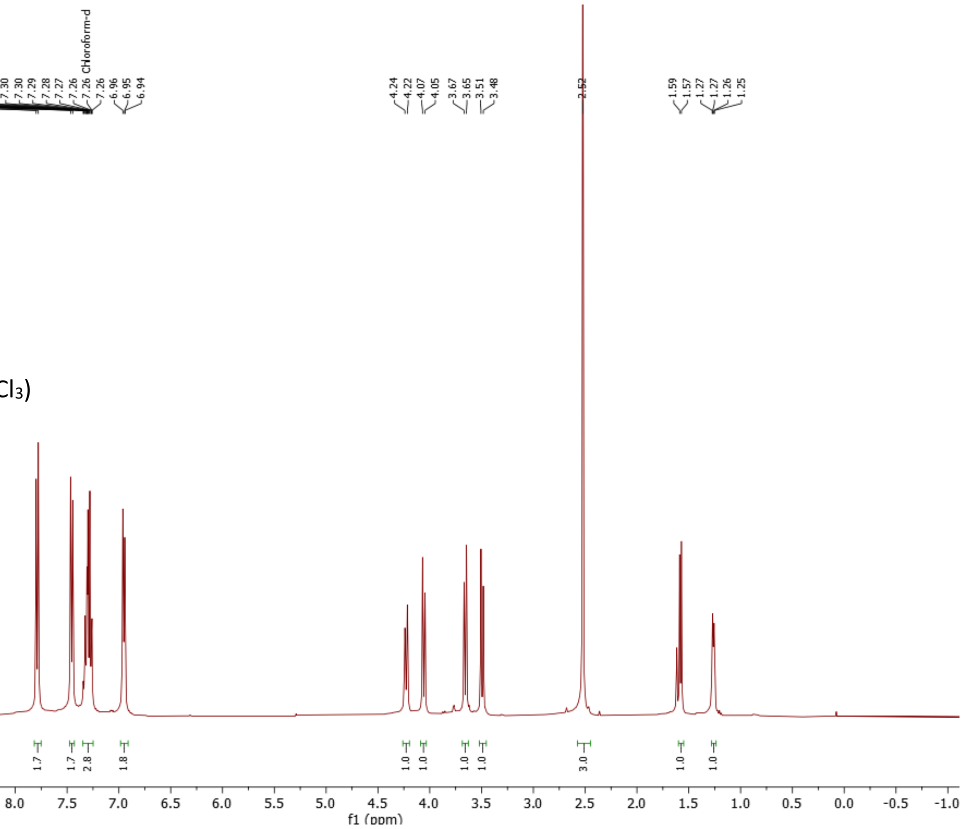

49

^{1}H NMR (400 MHz, $CDCl_3$)

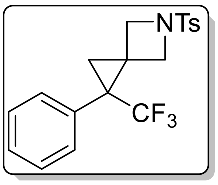

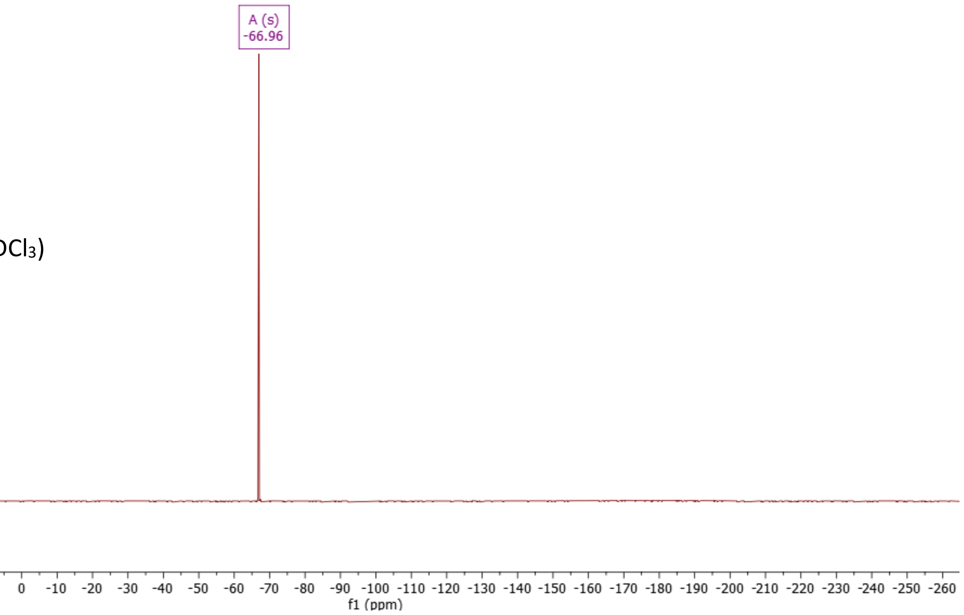

49

^{19}F NMR (377 MHz, $CDCl_3$)

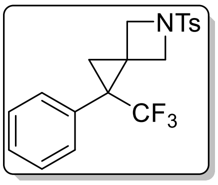

49

$^{13}C\{^1H\}$ NMR (101 MHz, $CDCl_3$)

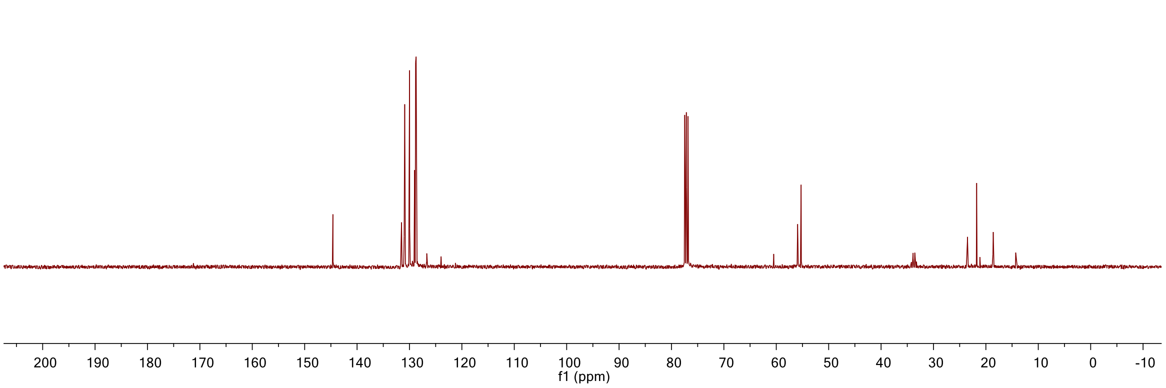

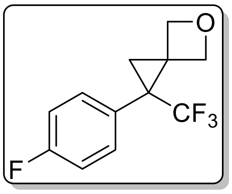

50

^{1}H NMR (400 MHz, $CDCl_3$)

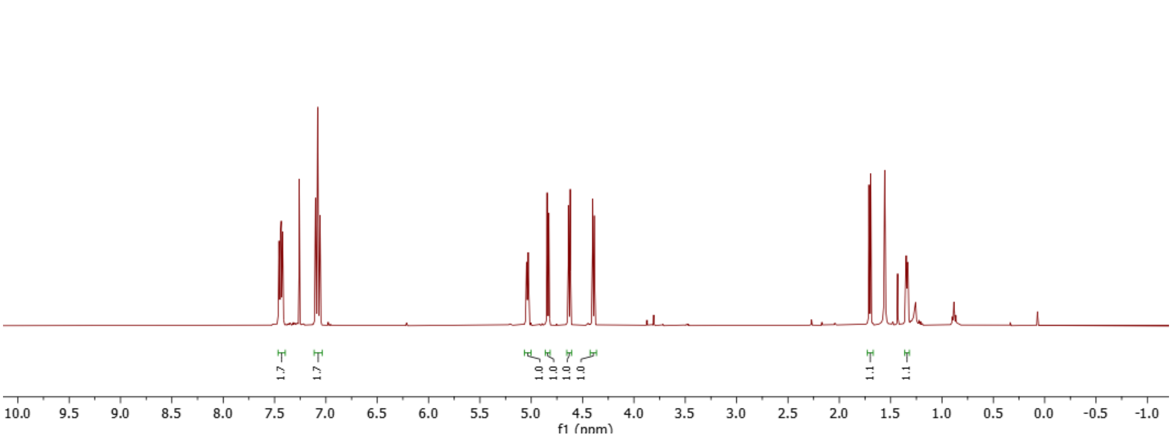

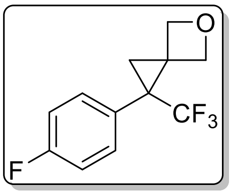

50

^{19}F NMR (377 MHz, $CDCl_3$)

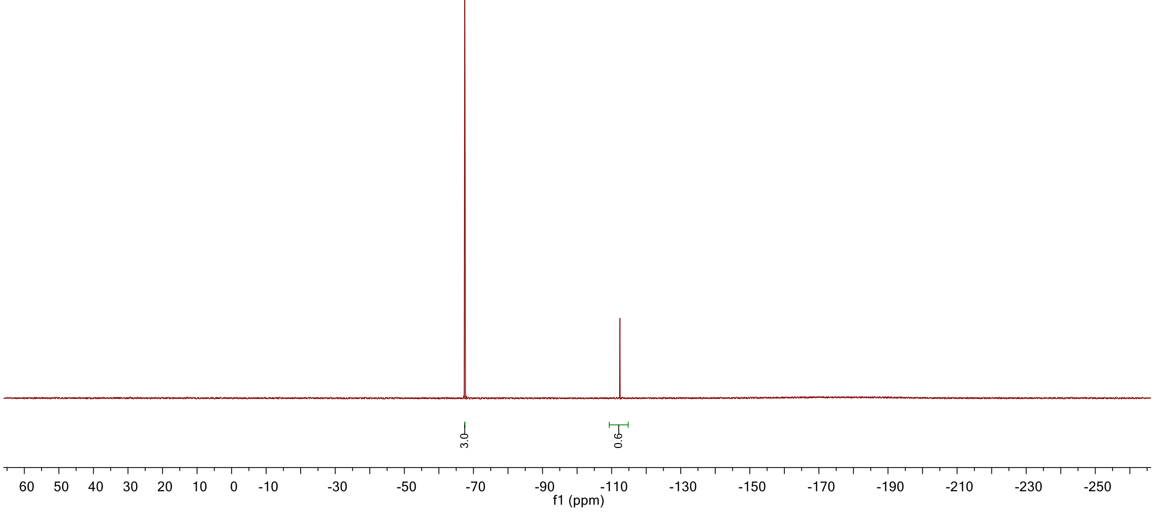

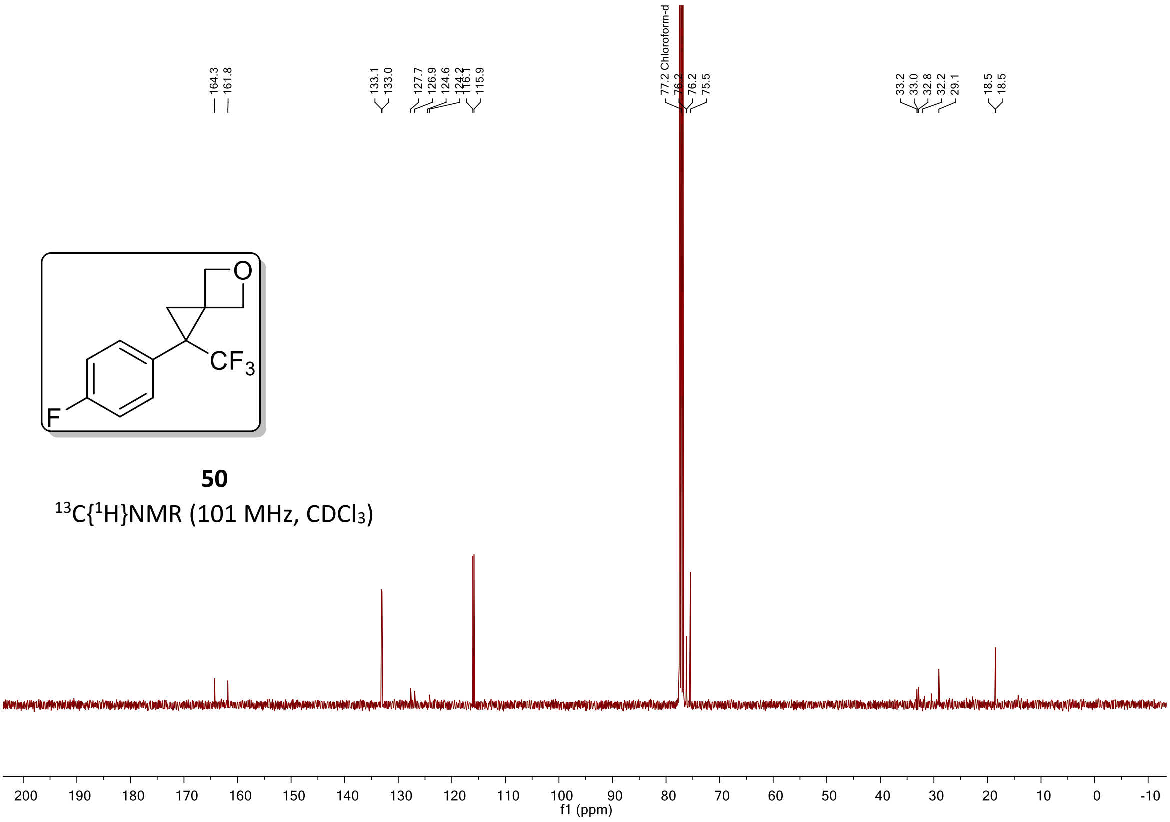

50

$^{13}C\{^{1}H\}NMR$ (101 MHz, $CDCl_3$)

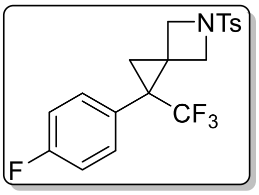

51

^{1}H NMR (400 MHz, $CDCl_3$)

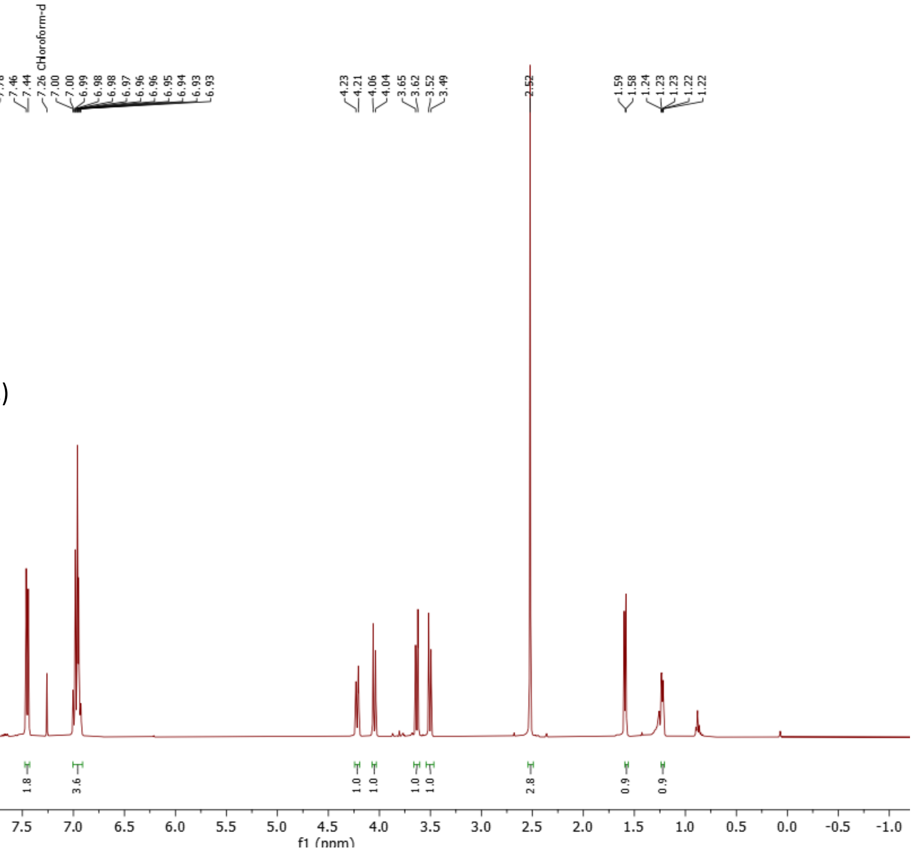

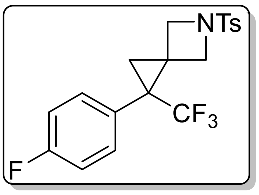

51

^{19}F NMR (377 MHz, $CDCl_3$)

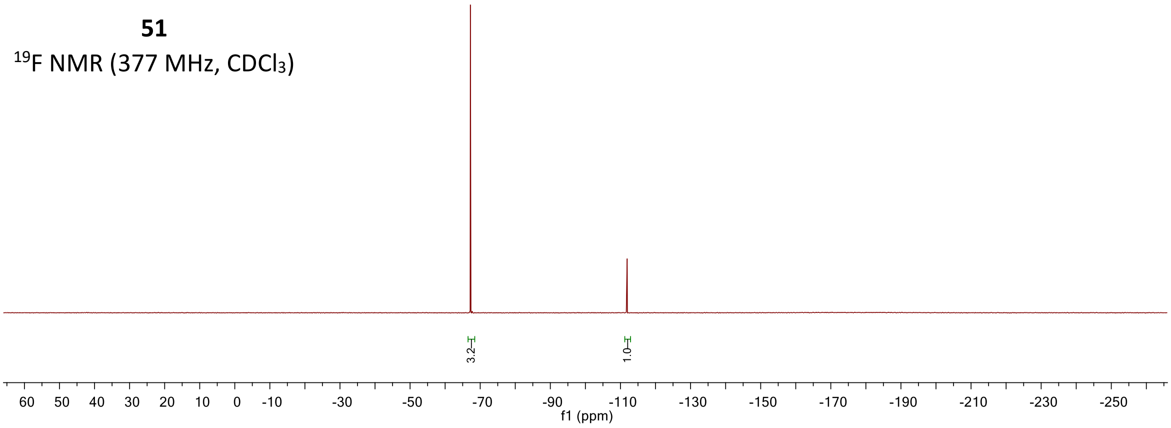

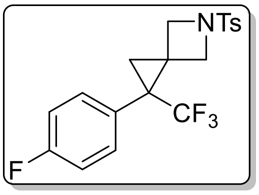

51

$^{13}C\{^1H\}$NMR (101 MHz, $CDCl_3$)

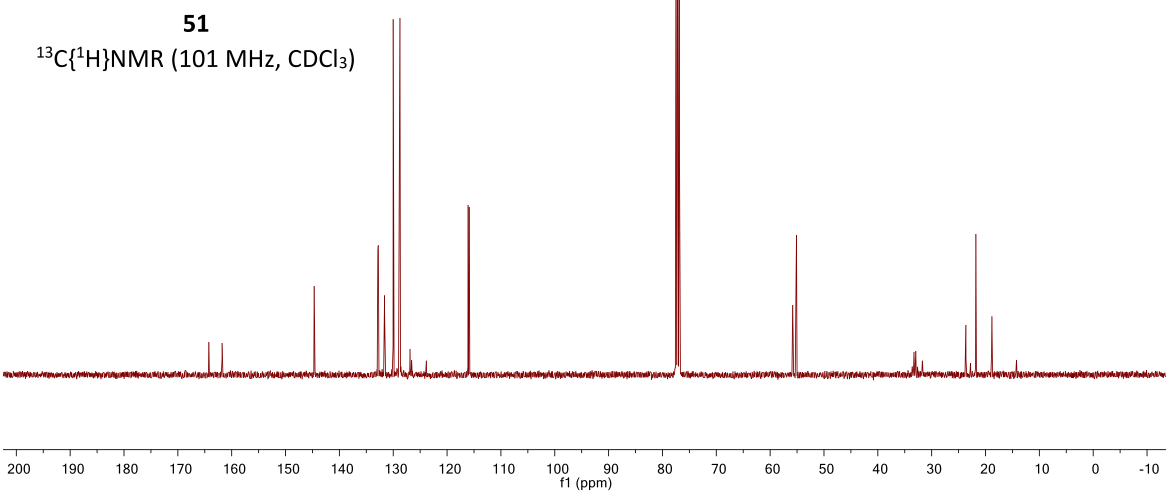

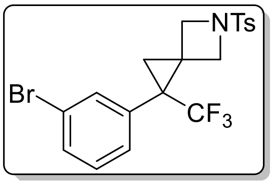

52

^1H NMR (400 MHz, $CDCl_3$)

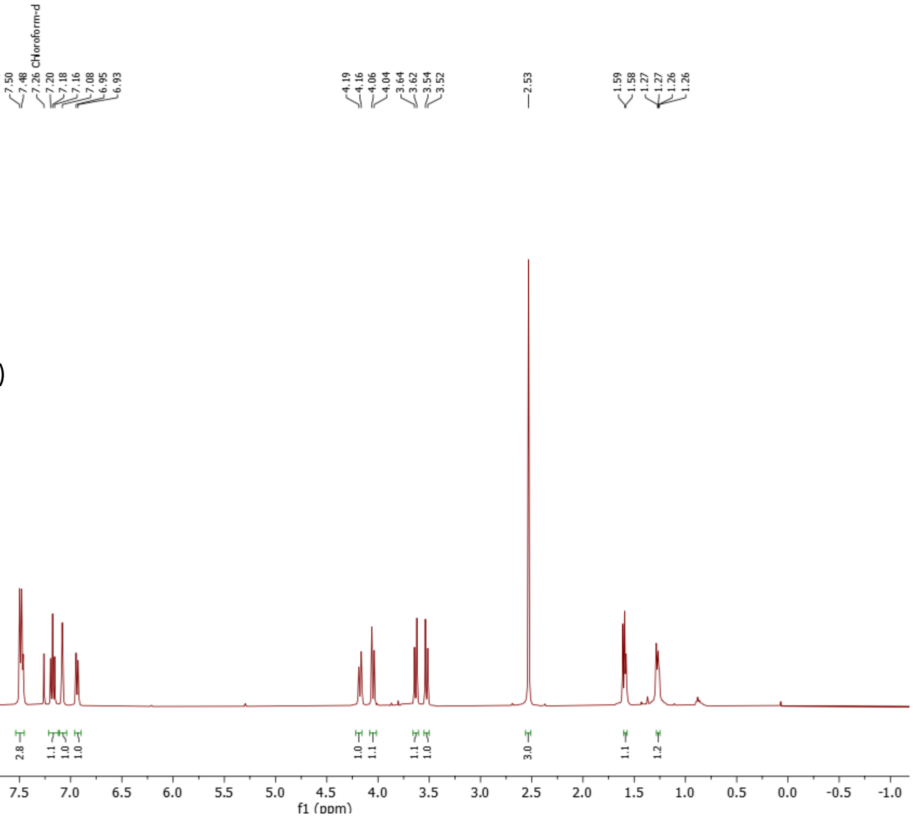

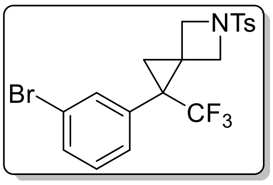

52

^{19}F NMR (377 MHz, $CDCl_3$)

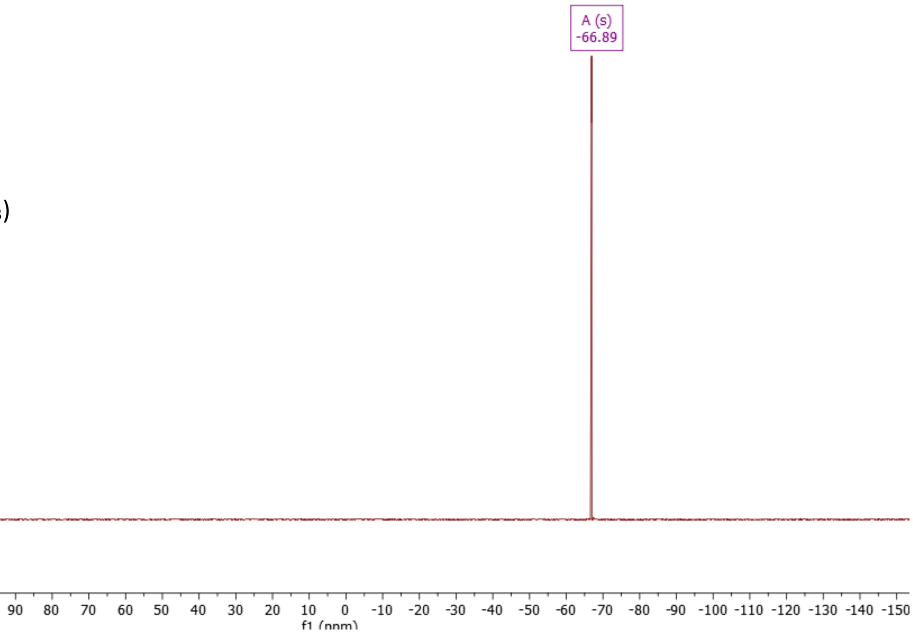

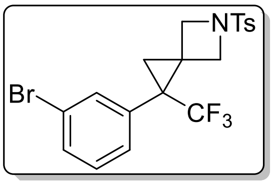

52

$^{13}C\{^{1}H\}$NMR (101 MHz, $CDCl_3$)

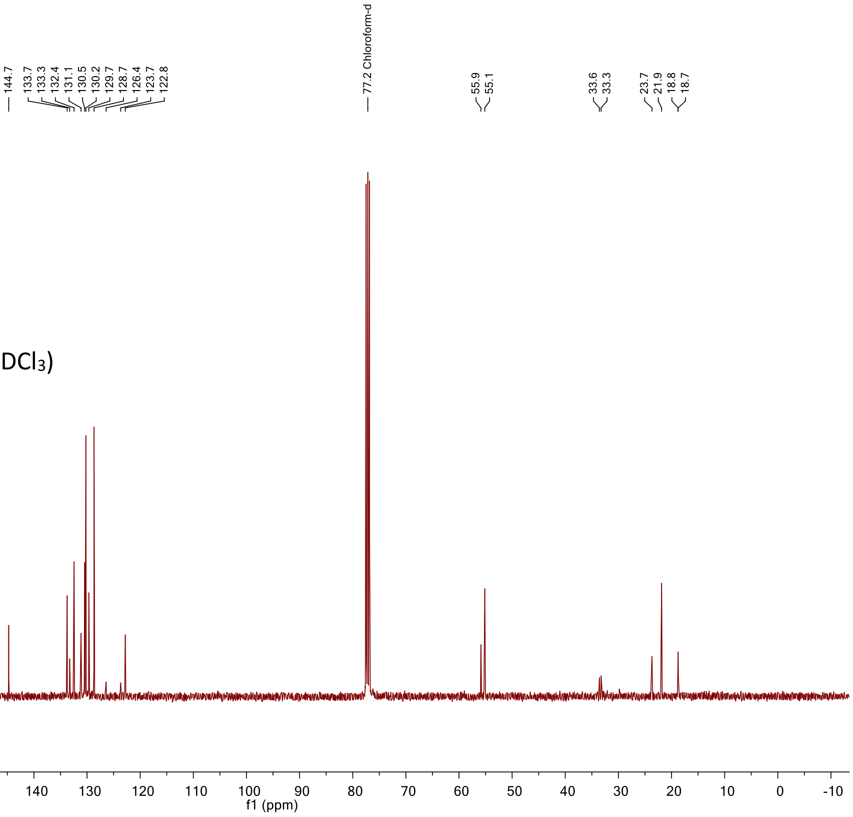

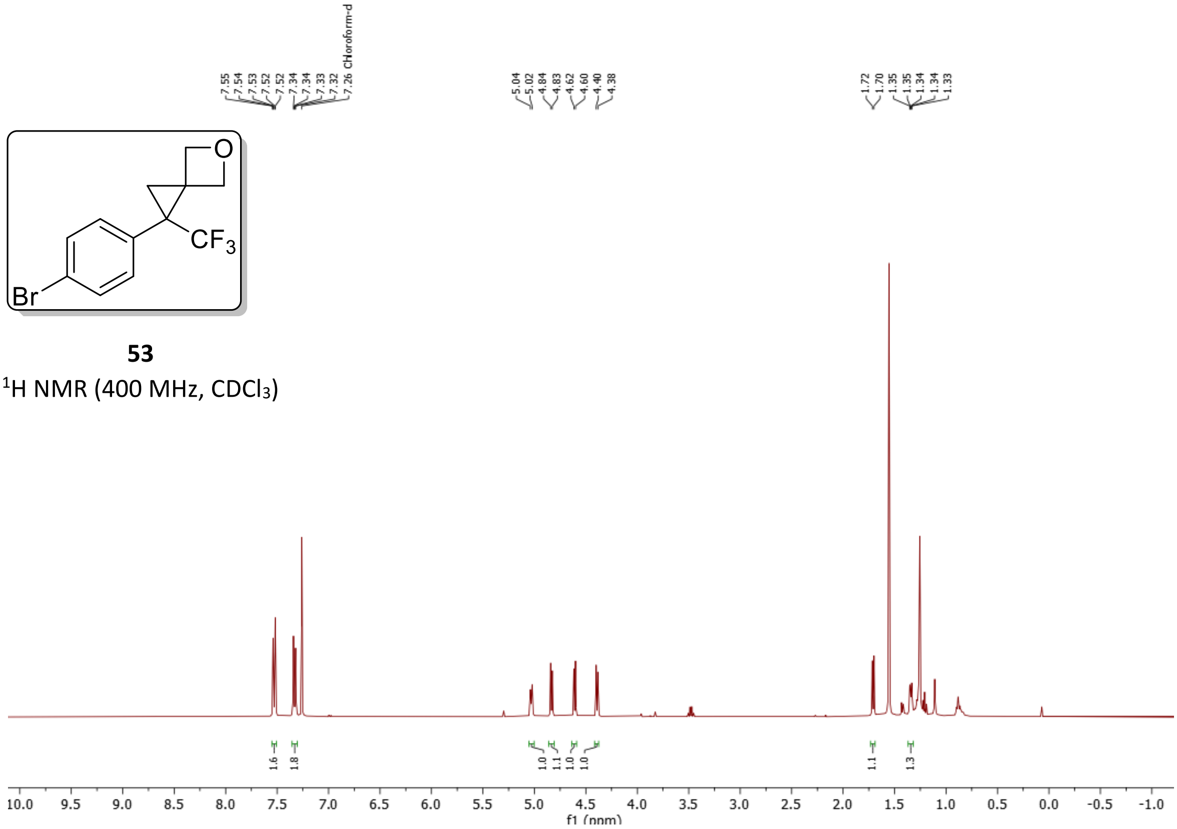

53

^{1}H NMR (400 MHz, $CDCl_3$)

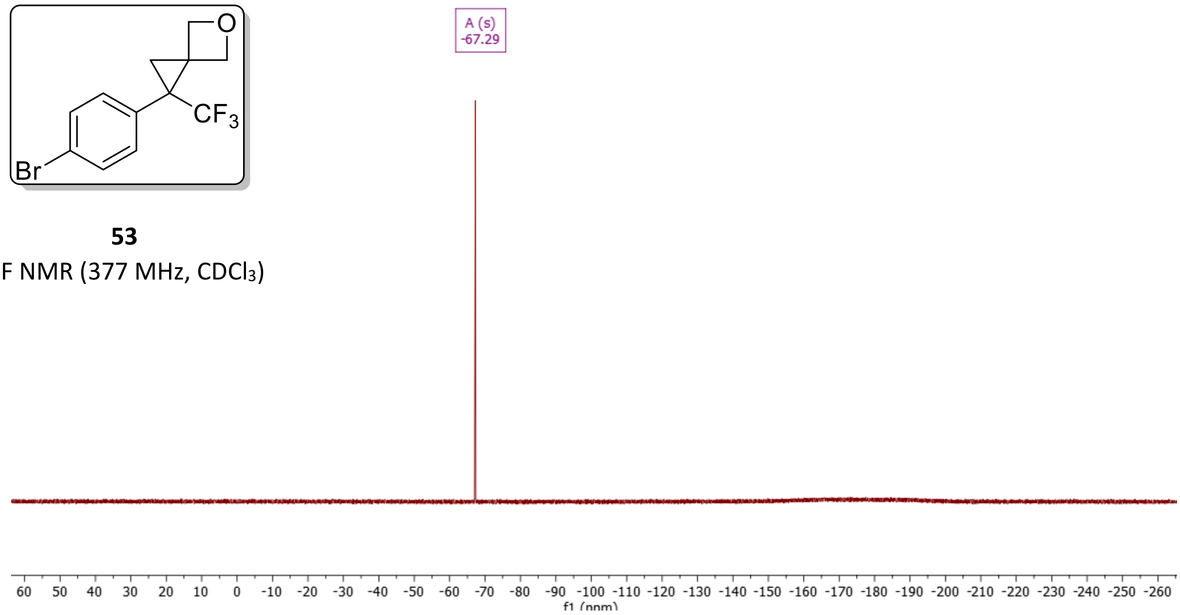

53

^{19}F NMR (377 MHz, $CDCl_3$)

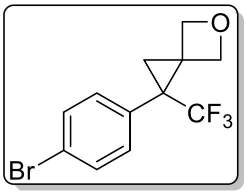

53

$^{13}C\{^1H\}$NMR (101 MHz, $CDCl_3$)

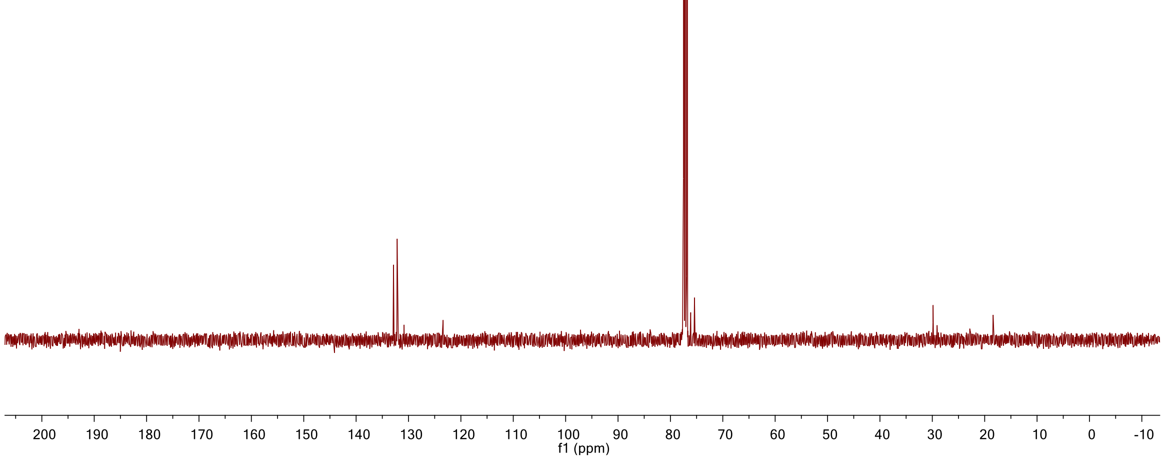

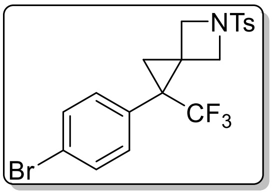

54

^{1}H NMR (400 MHz, $CDCl_3$)

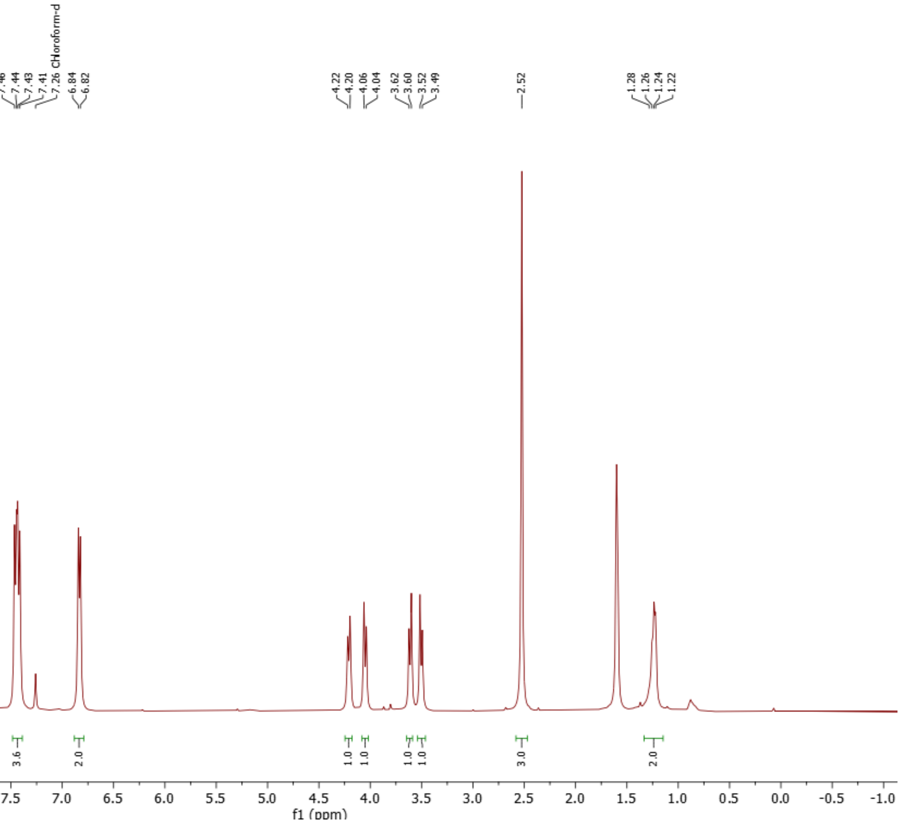

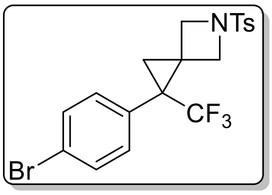

54

^{19}F NMR (377 MHz, $CDCl_3$)

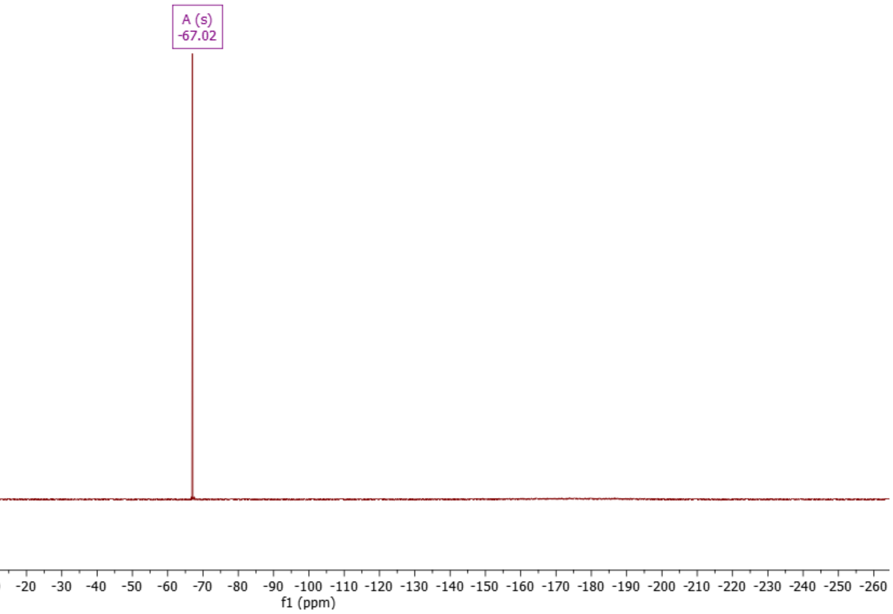

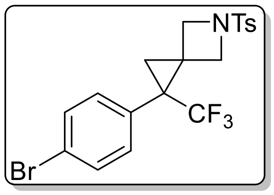

54

$^{13}C\{^{1}H\}$NMR (101 MHz, $CDCl_3$)

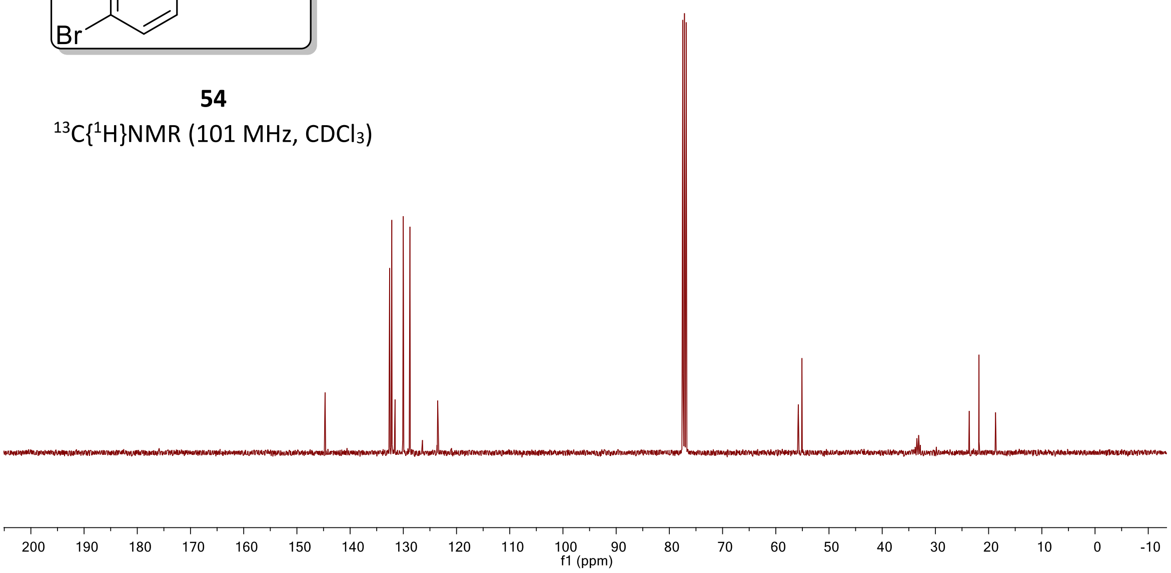

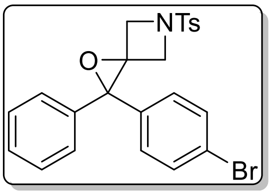

56

^{1}H NMR (400 MHz, $CDCl_3$)

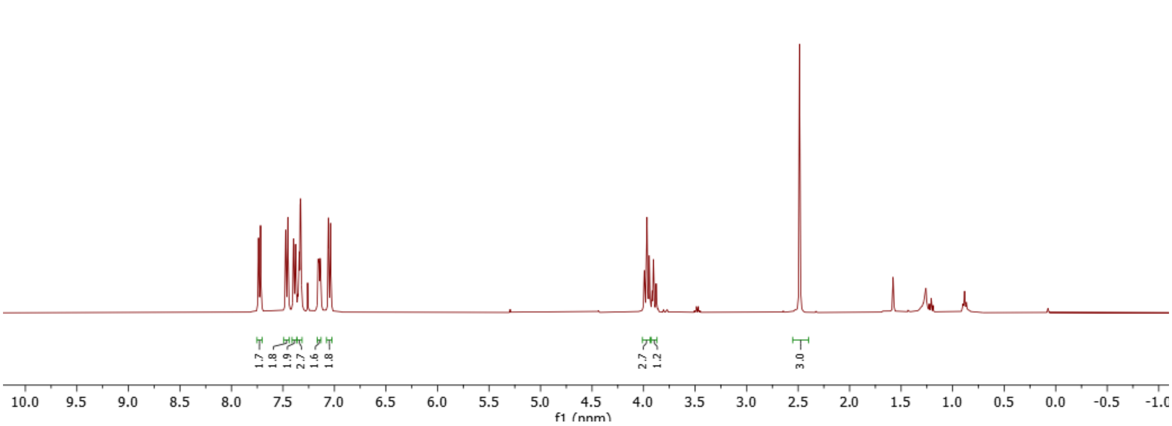

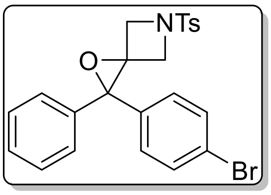

56

^{13}C {^{1}H} NMR (101 MHz, $CDCl_3$)

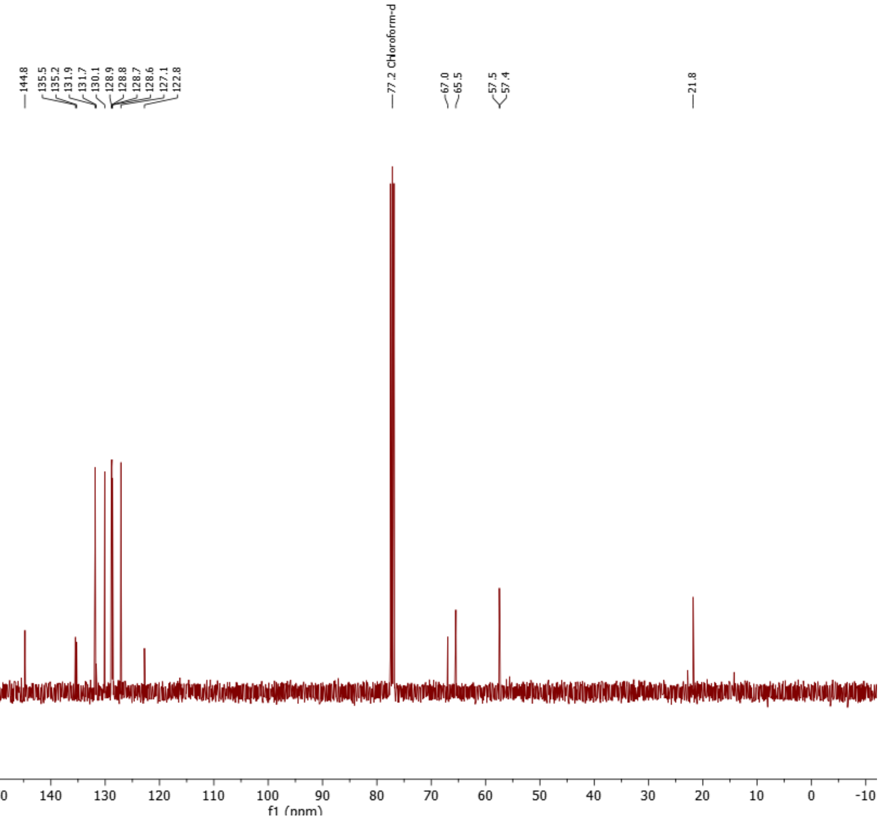

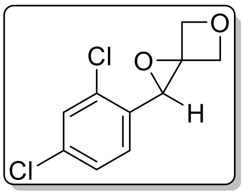

57
^{1}H NMR (400 MHz, $CDCl_3$)

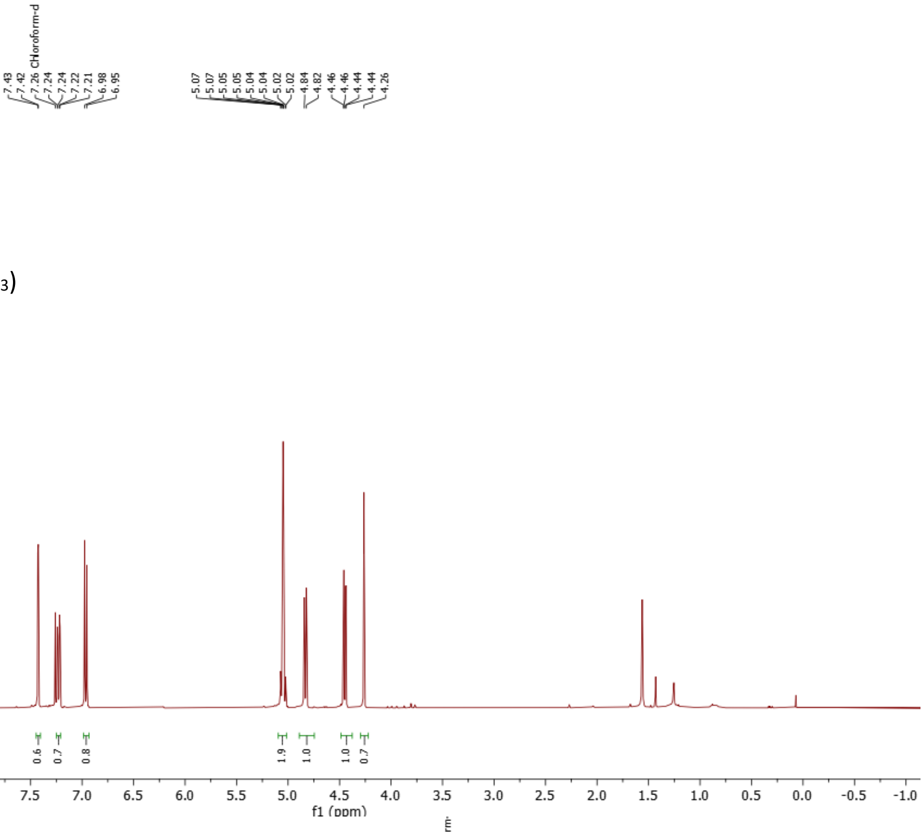

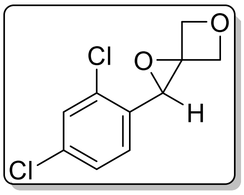

57
^{13}C {^{1}H} NMR (101 MHz, $CDCl_3$)

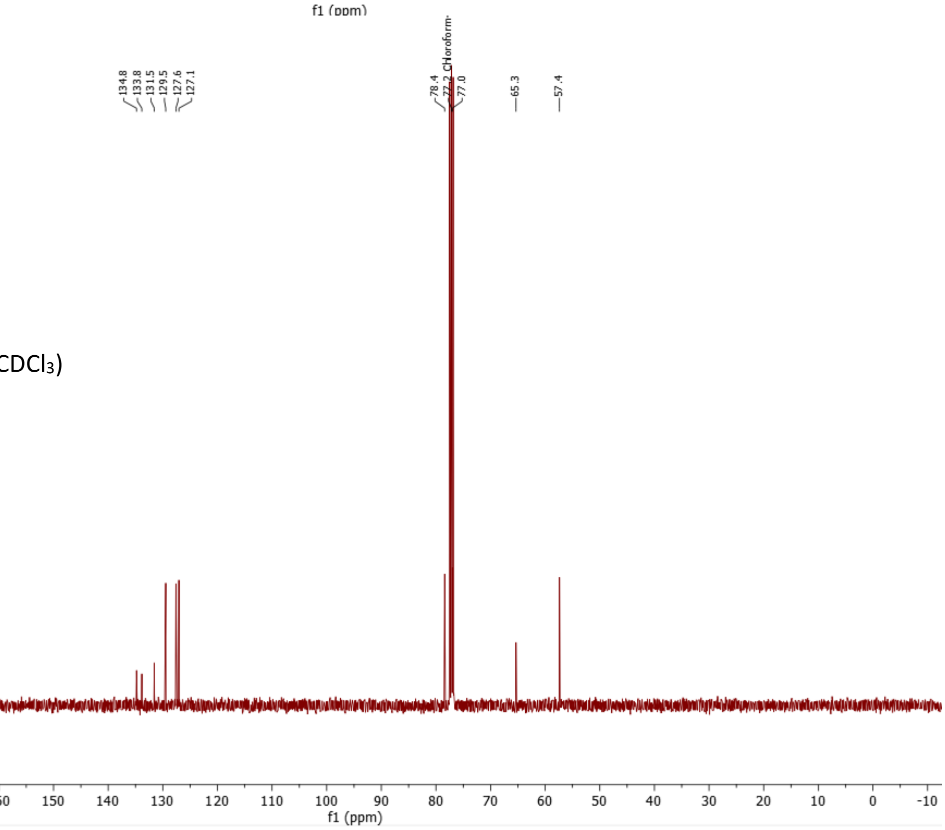

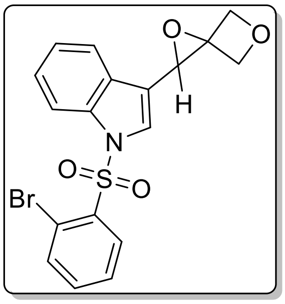

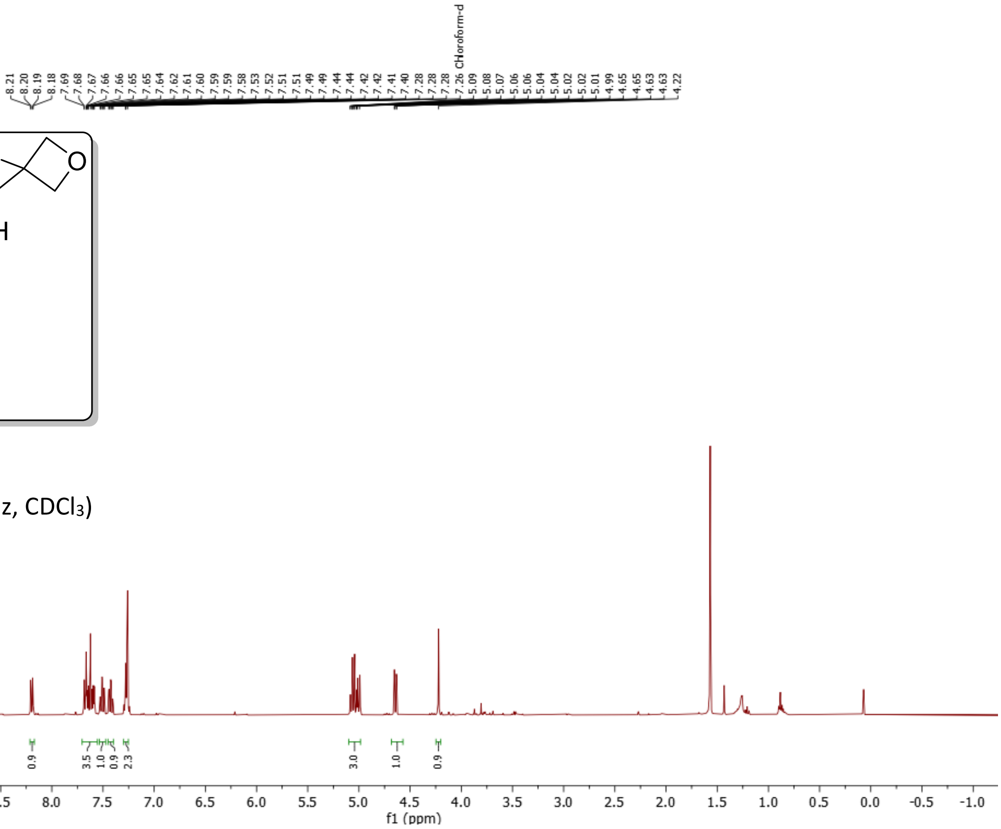

58

^{1}H NMR (400 MHz, $CDCl_3$)

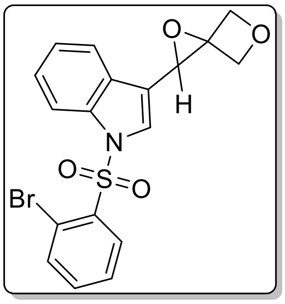

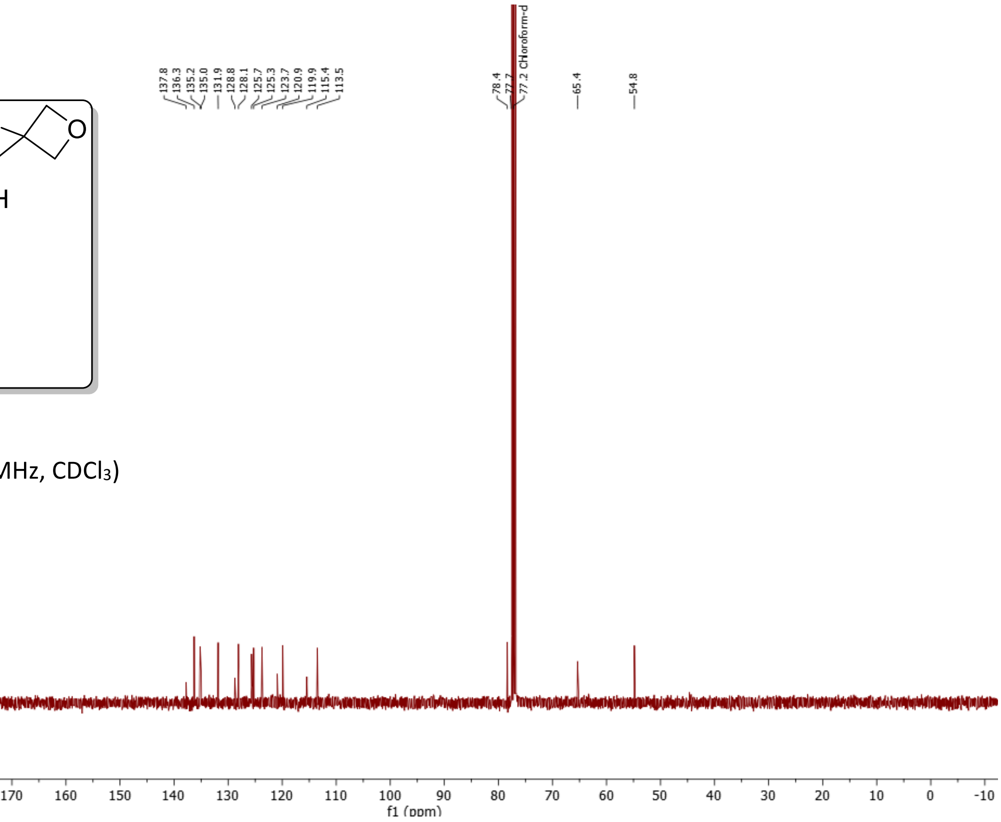

58

^{13}C {^{1}H} NMR (101 MHz, $CDCl_3$)

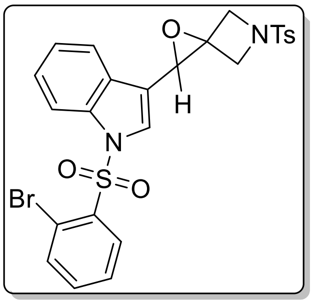

59

^{1}H NMR (400 MHz, $CDCl_3$)

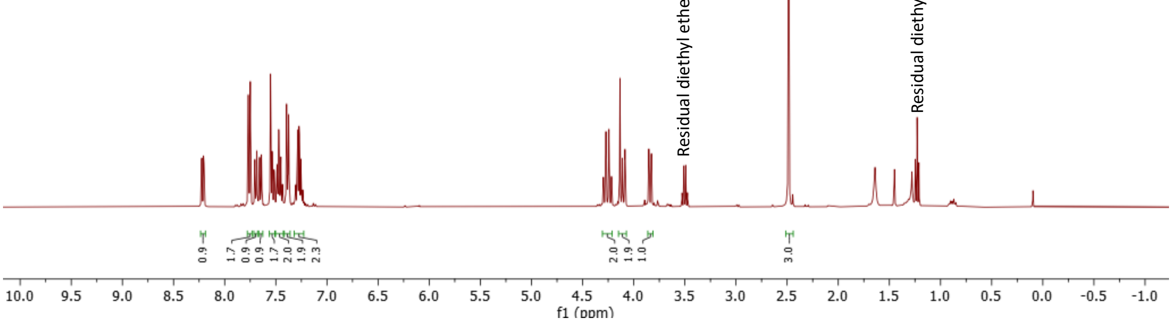

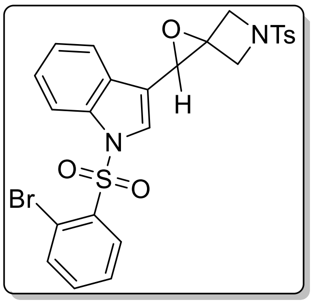

59

^{13}C {^{1}H} NMR (101 MHz, $CDCl_3$)

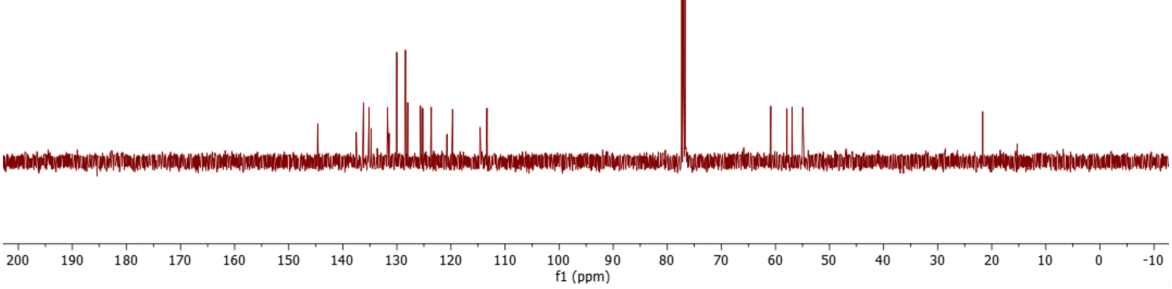

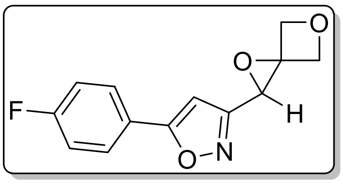

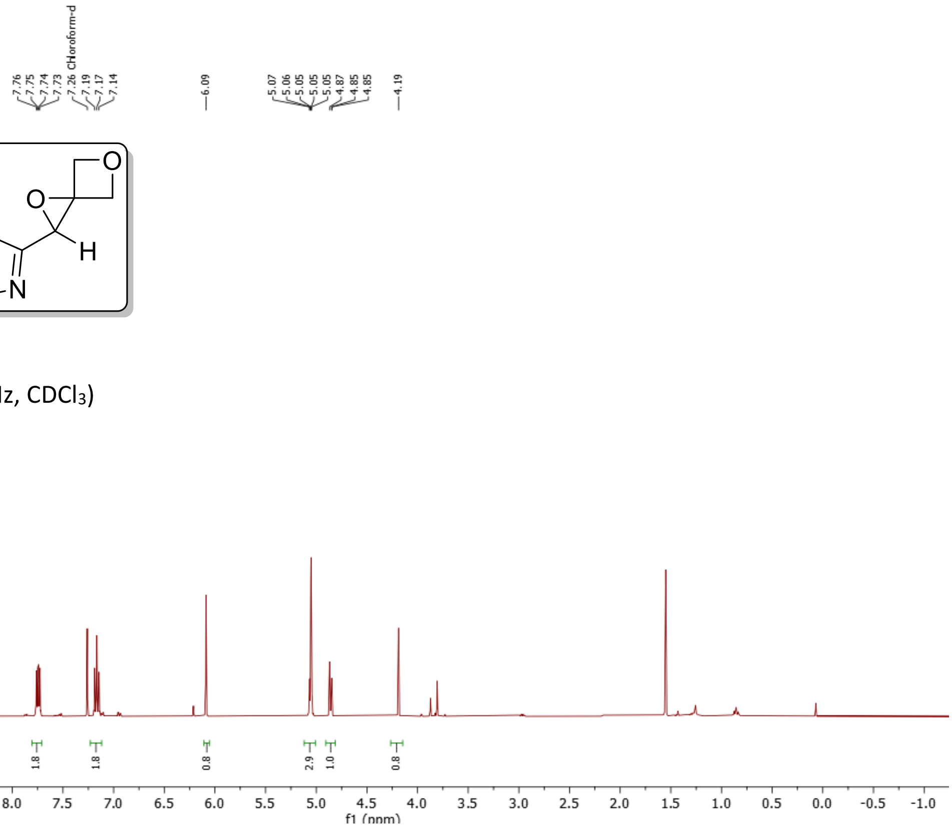

60
^{1}H NMR (400 MHz, $CDCl_3$)

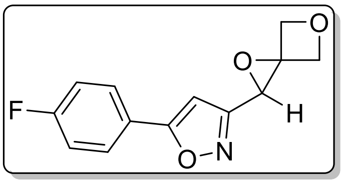

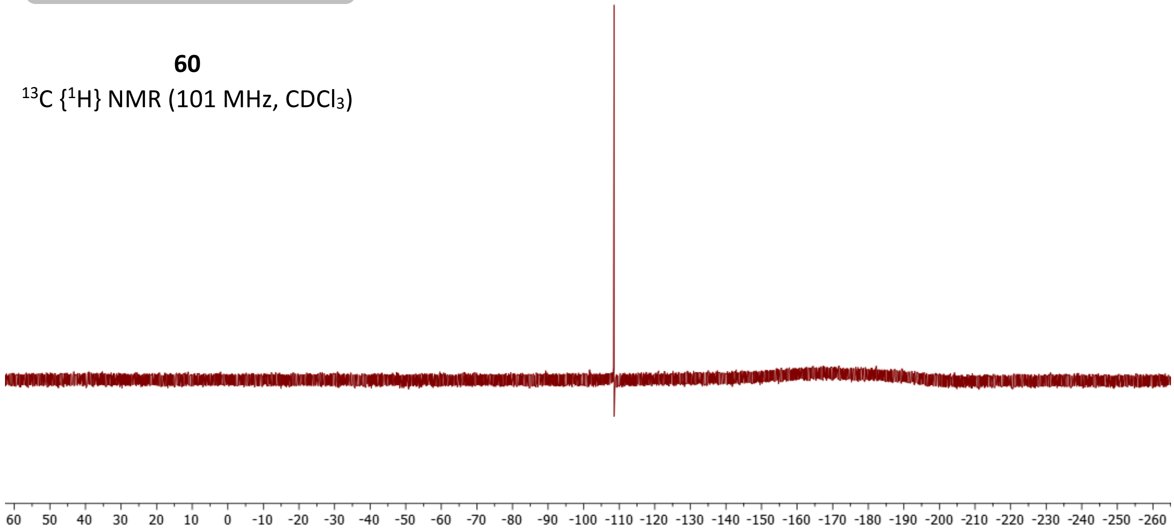

60
^{13}C {^{1}H} NMR (101 MHz, $CDCl_3$)

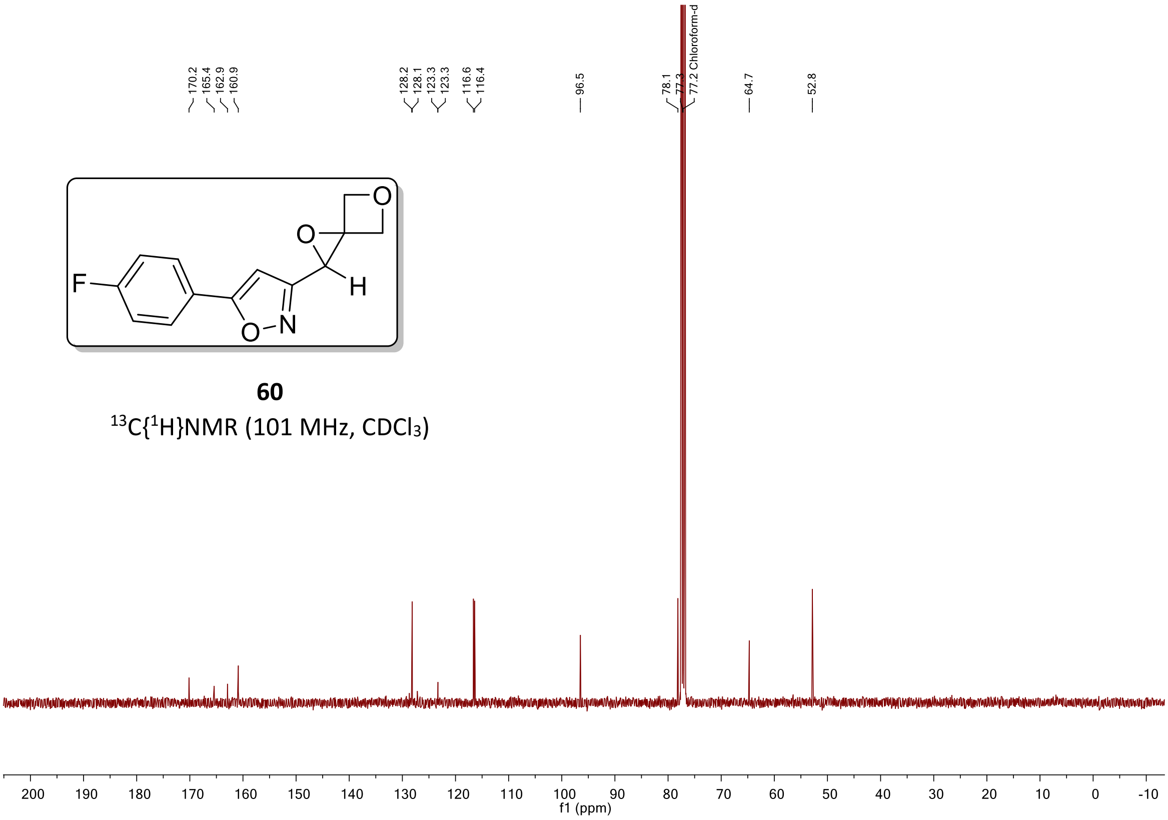

60

$^{13}C\{^{1}H\}$NMR (101 MHz, $CDCl_3$)

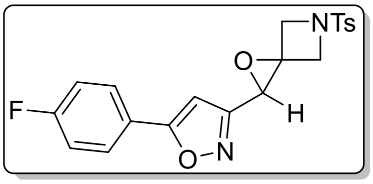

61

^{1}H NMR (400 MHz, $CDCl_3$)

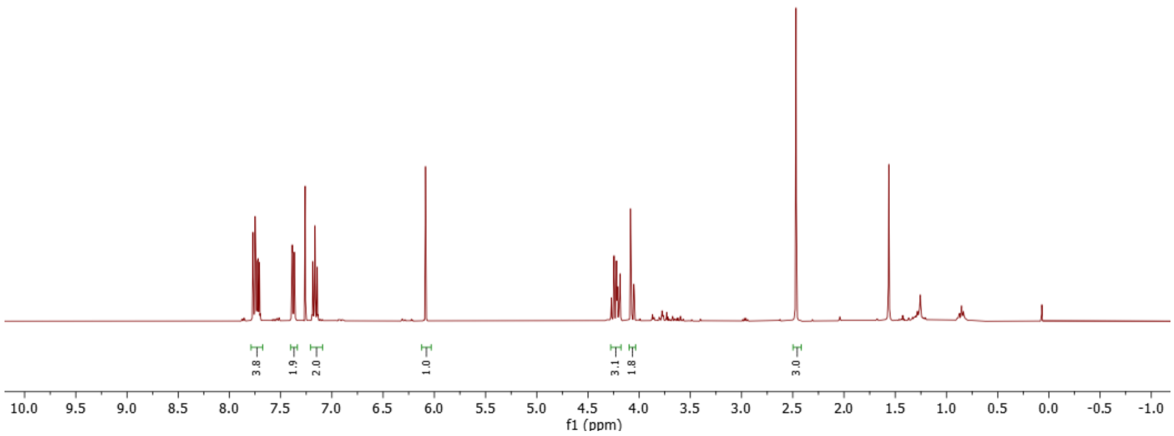

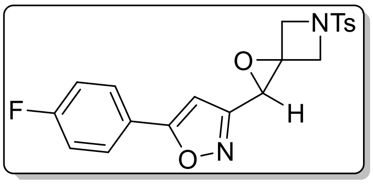

61

^{19}F NMR (377 MHz, $CDCl_3$)

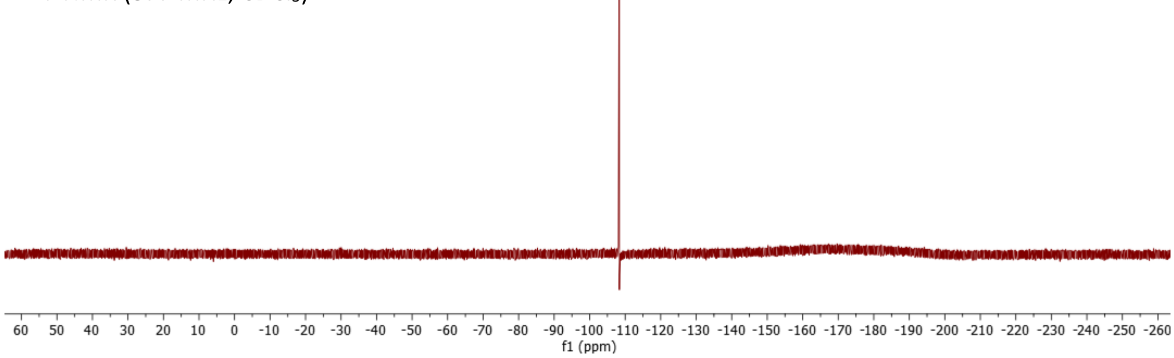

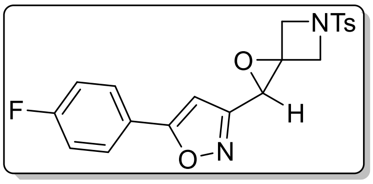

61

$^{13}C\ \{^1H\}$ NMR (101 MHz, $CDCl_3$)

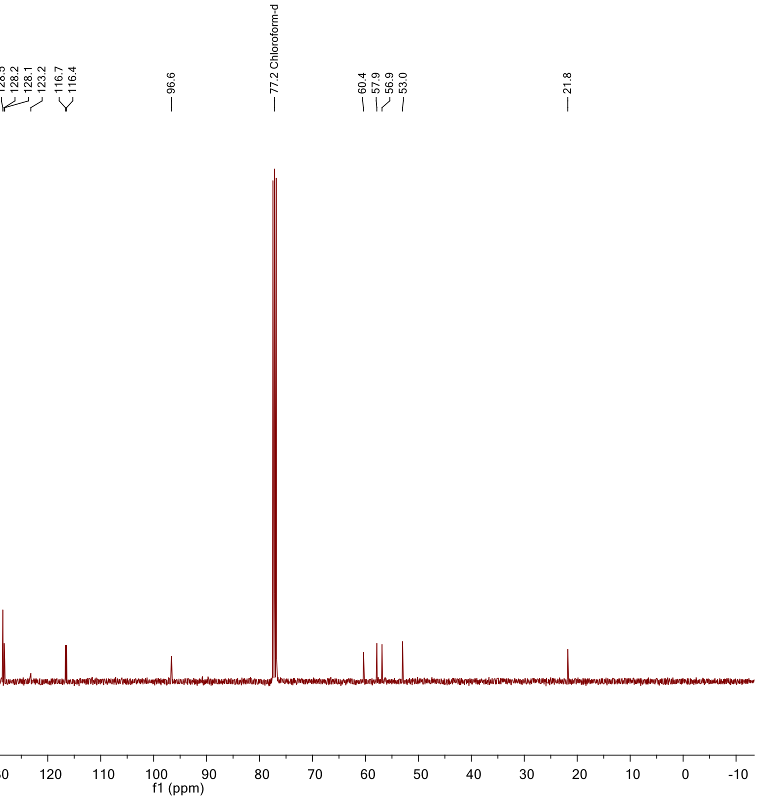

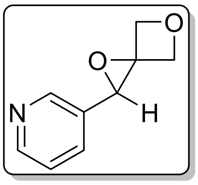

62

^{1}H NMR (400 MHz, $CDCl_3$)

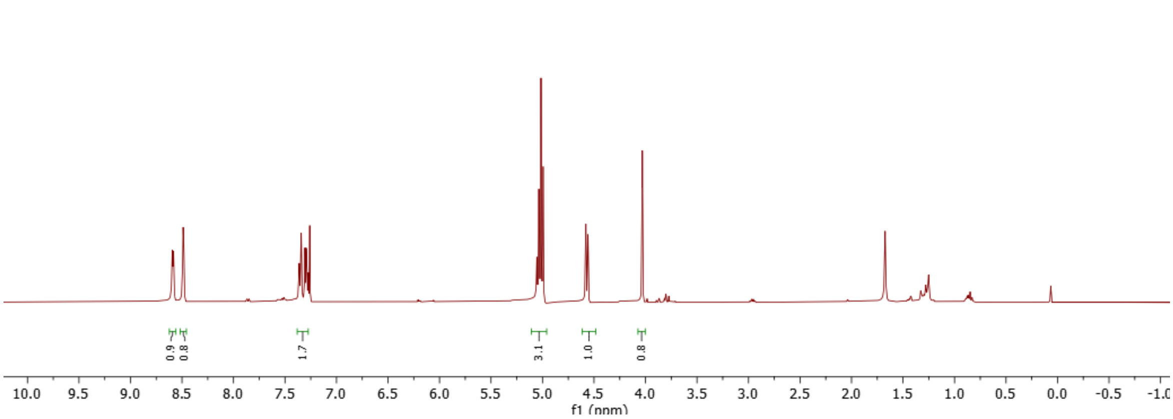

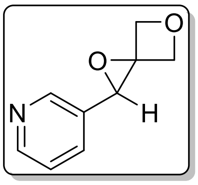

62

$^{13}C\{^{1}H\}$ NMR (101 MHz, $CDCl_3$)

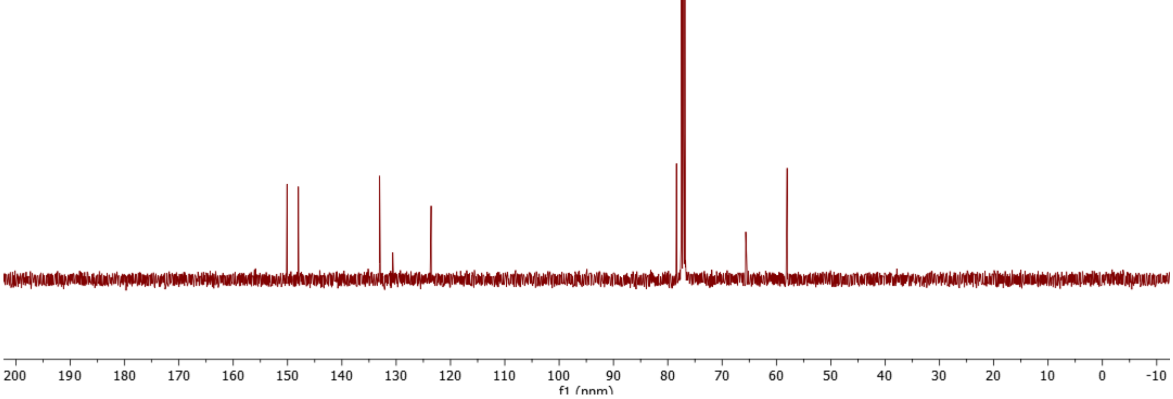

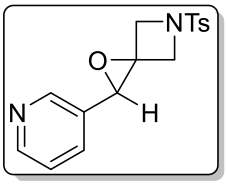

63

^{1}H NMR (400 MHz, $CDCl_3$)

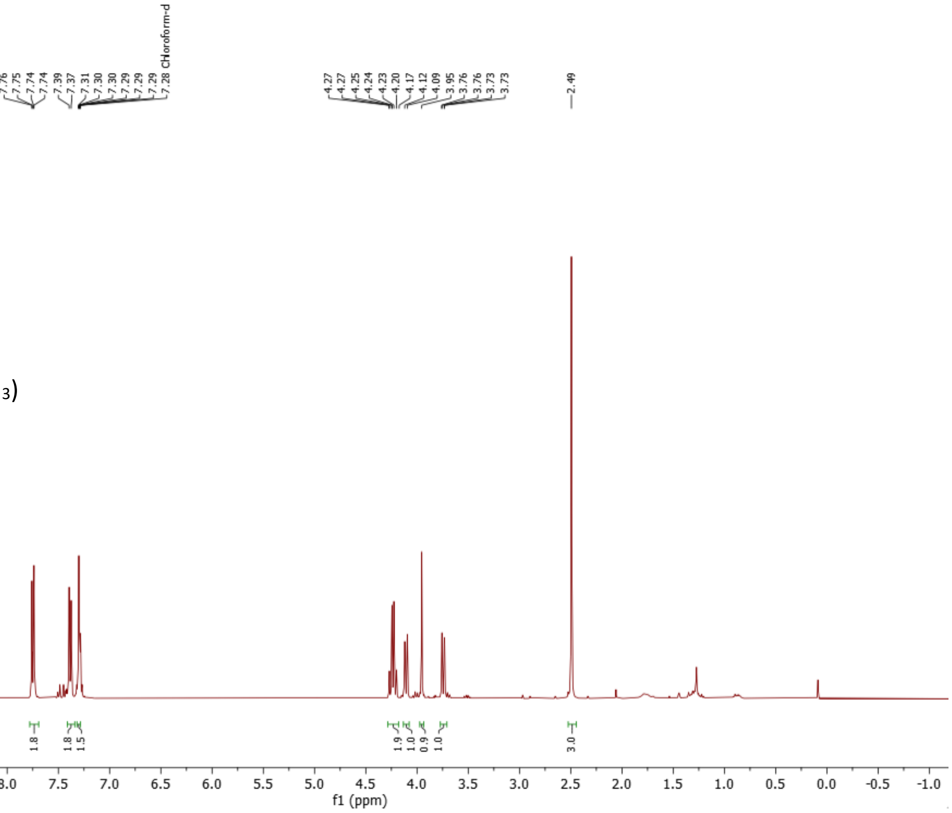

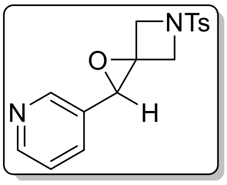

63

^{13}C {^{1}H} NMR (101 MHz, $CDCl_3$)

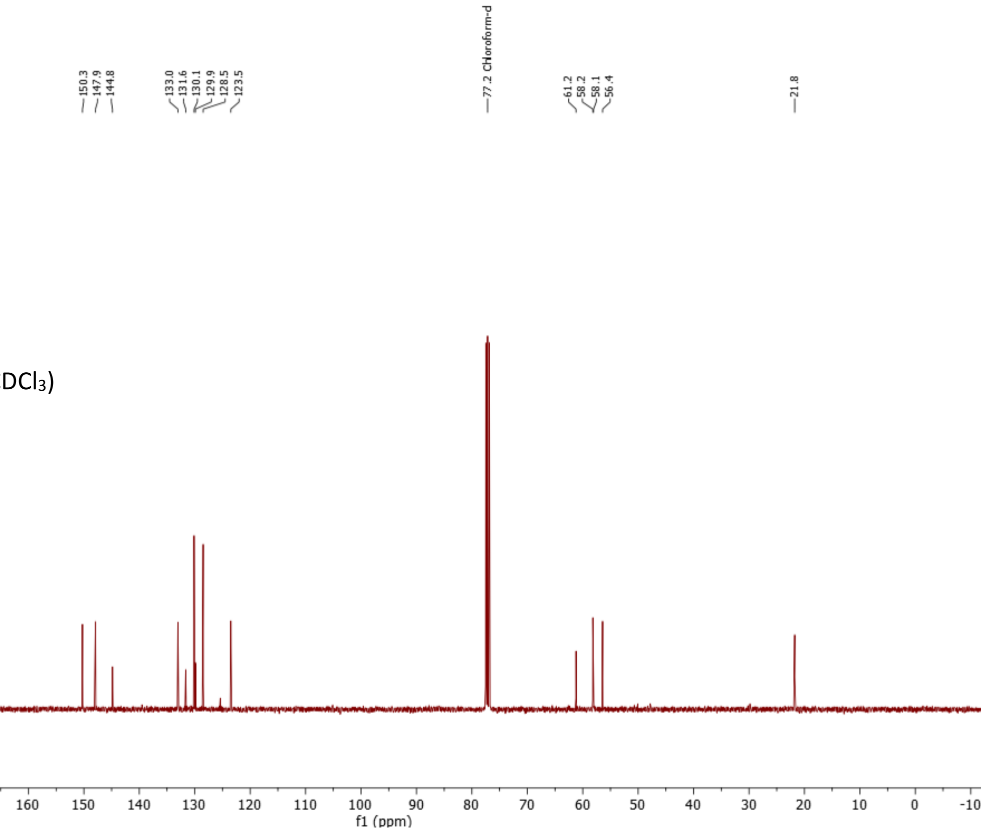

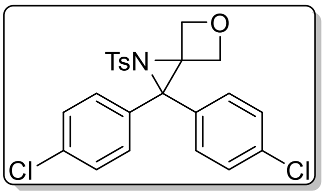

64

^{1}H NMR (400 MHz, $CDCl_3$)

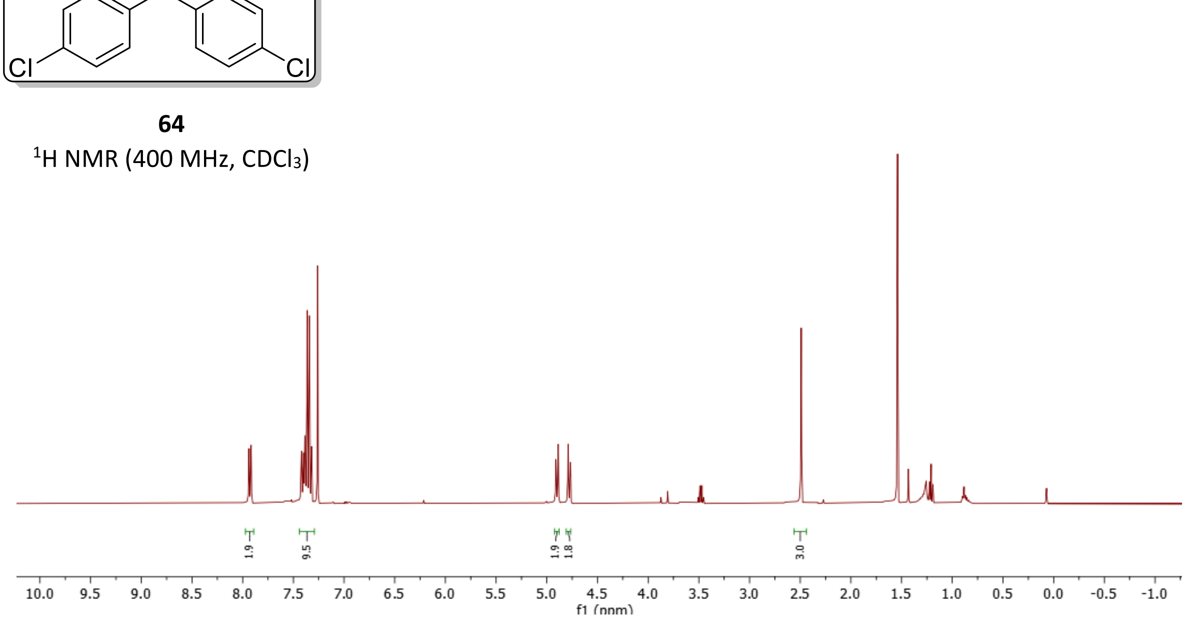

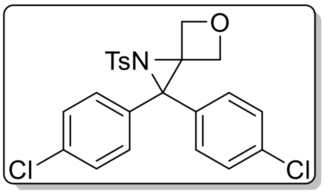

64

^{13}C {^{1}H} NMR (101 MHz, $CDCl_3$)

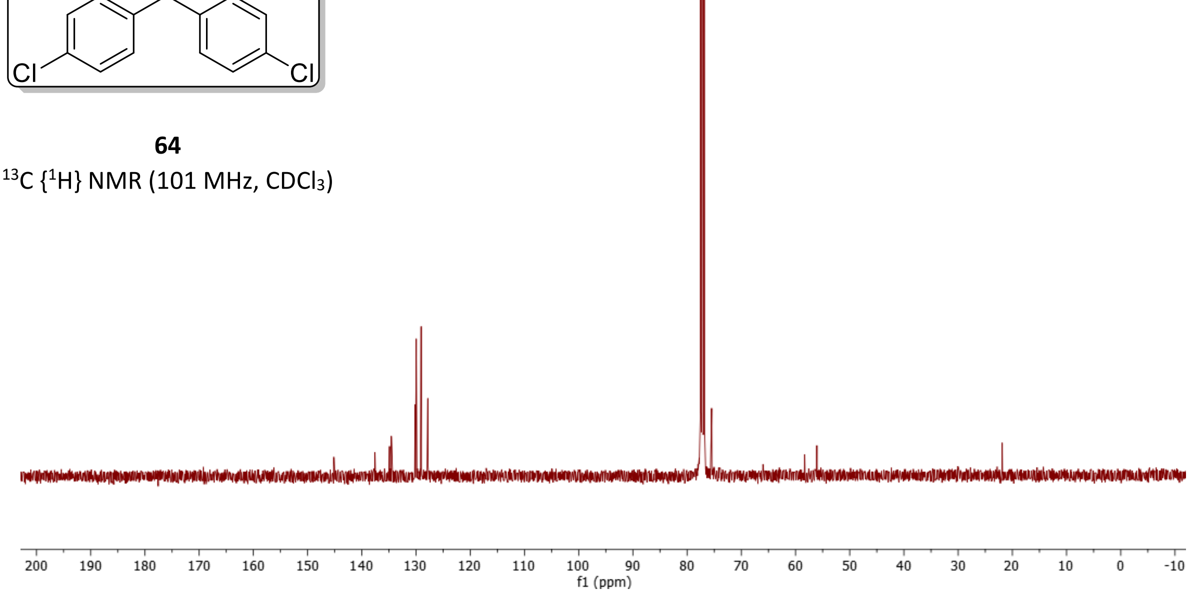

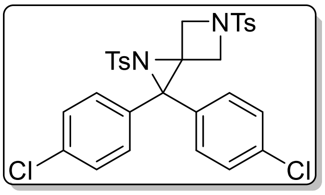

65

^{1}H NMR (400 MHz, $CDCl_3$)

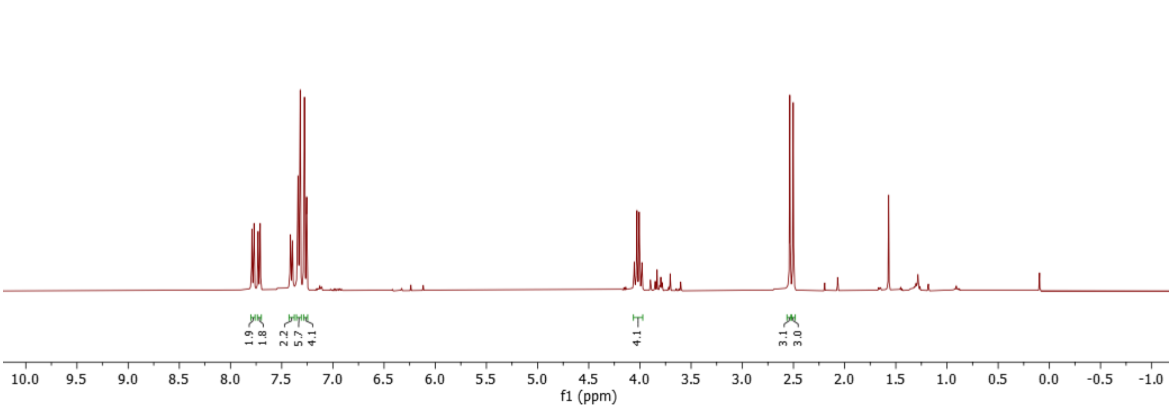

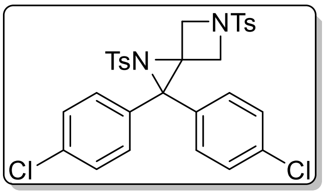

65

^{13}C {^{1}H} NMR (101 MHz, $CDCl_3$)

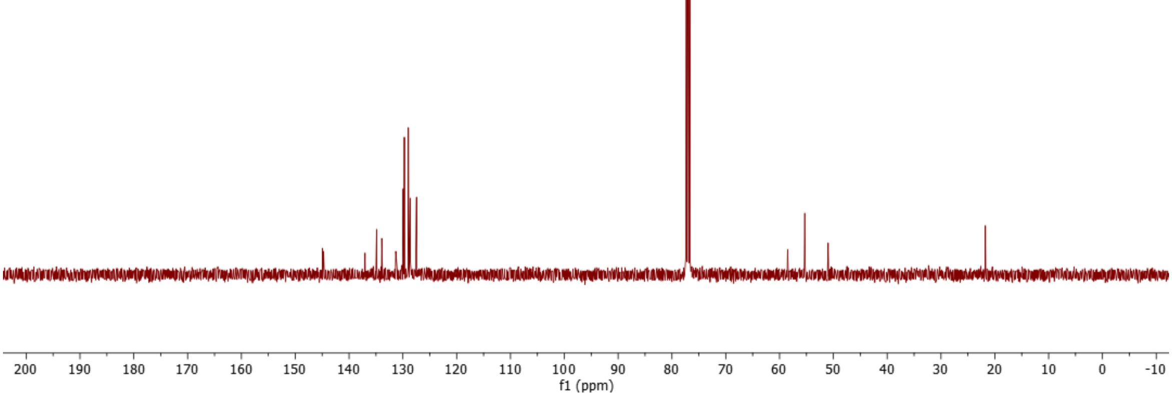

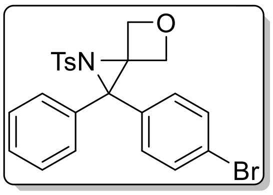

66

^{1}H NMR (400 MHz, $CDCl_3$)

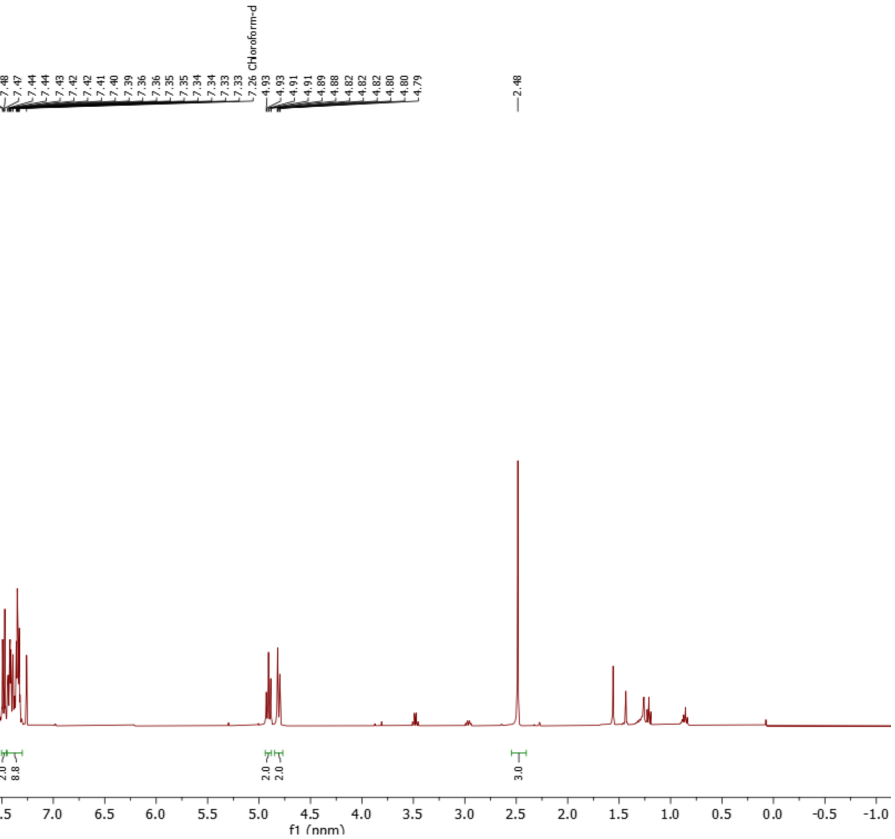

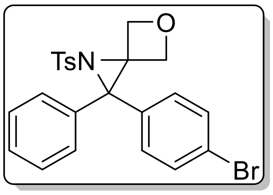

66

^{13}C {^{1}H} NMR (101 MHz, $CDCl_3$)

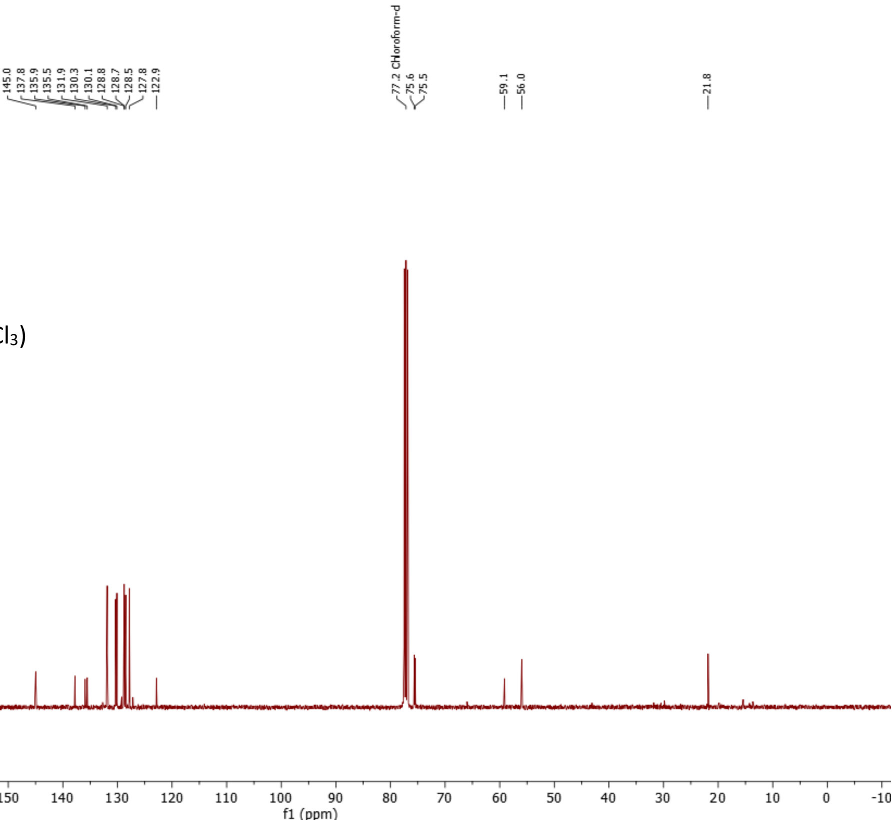

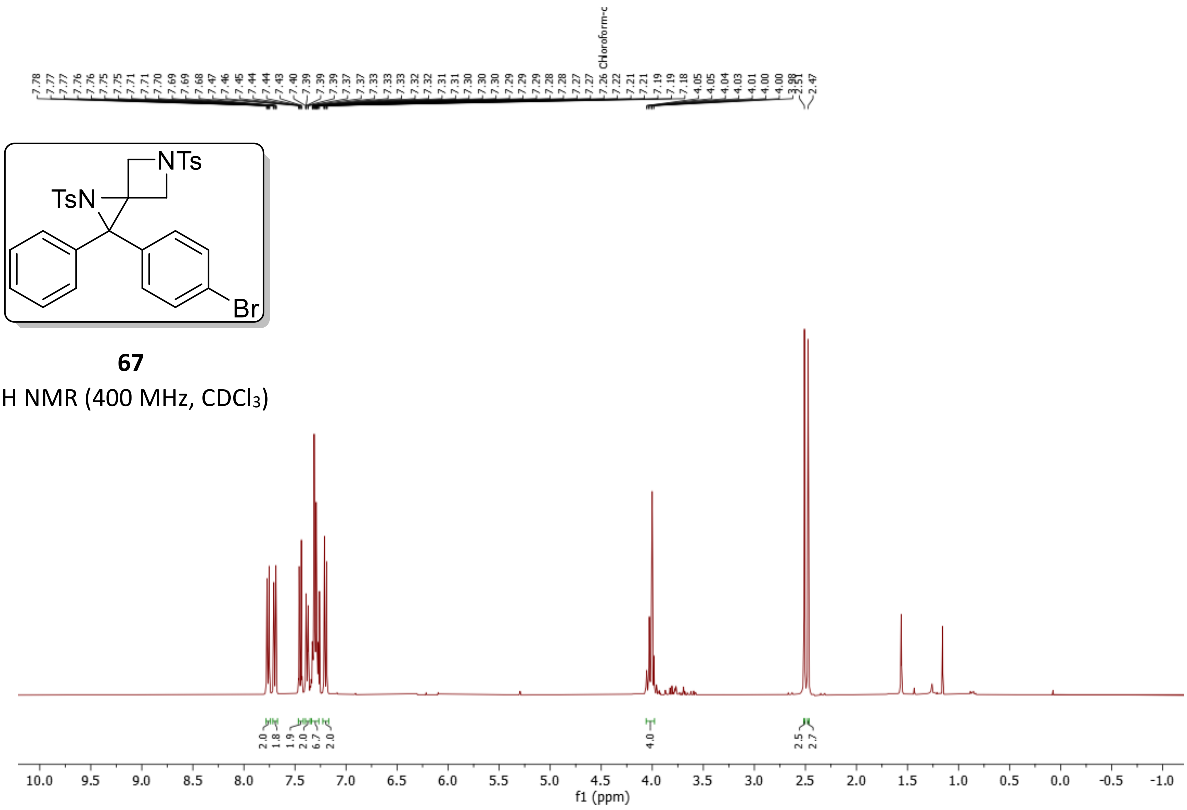

67

^{1}H NMR (400 MHz, $CDCl_3$)

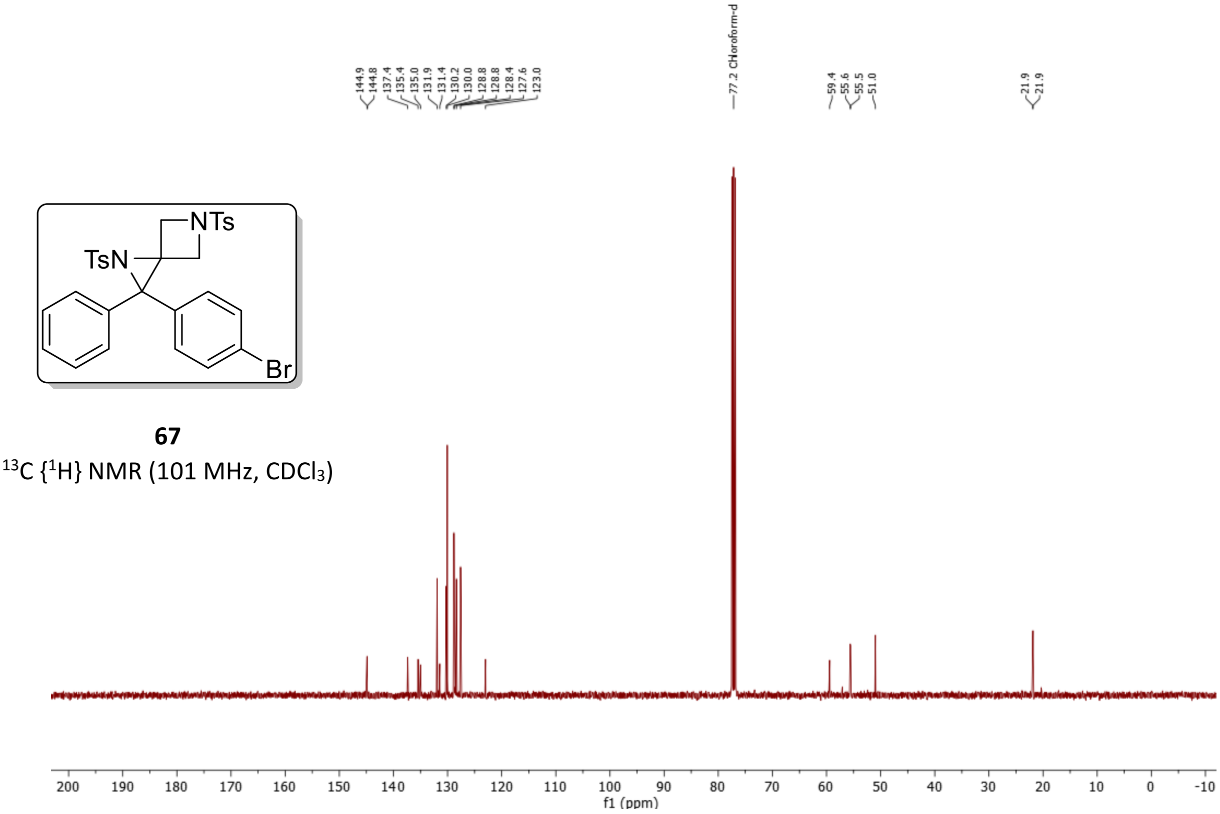

67

^{13}C {^{1}H} NMR (101 MHz, $CDCl_3$)

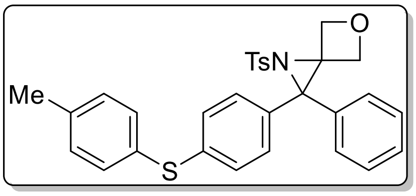

68

^{1}H NMR (400 MHz, $CDCl_3$)

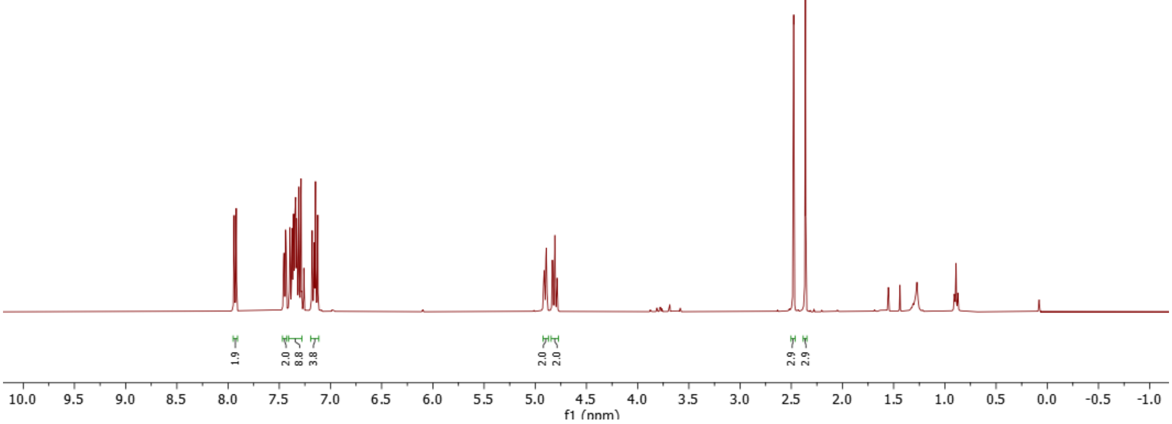

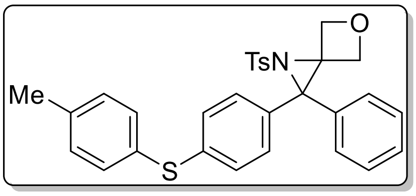

68

^{13}C {^{1}H} NMR (101 MHz, $CDCl_3$)

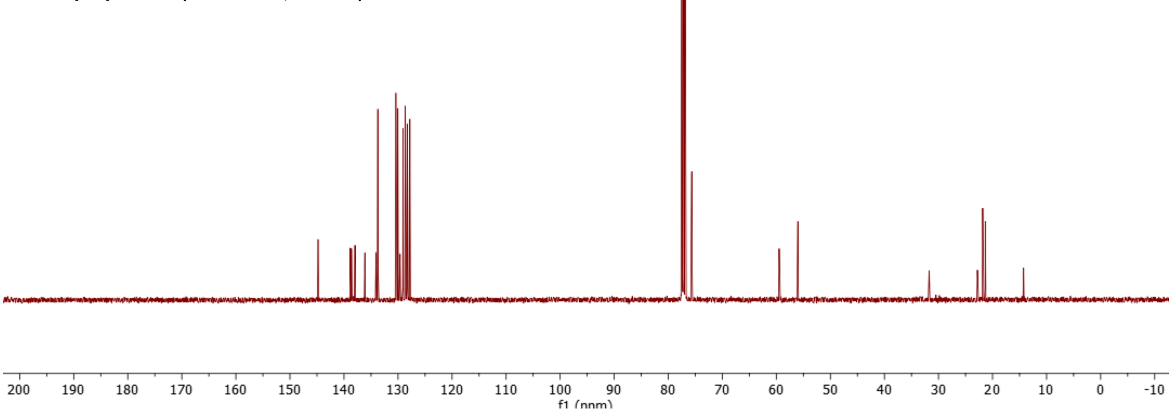

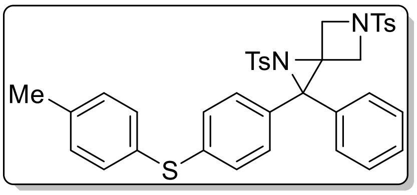

69

^{1}H NMR (400 MHz, $CDCl_3$)

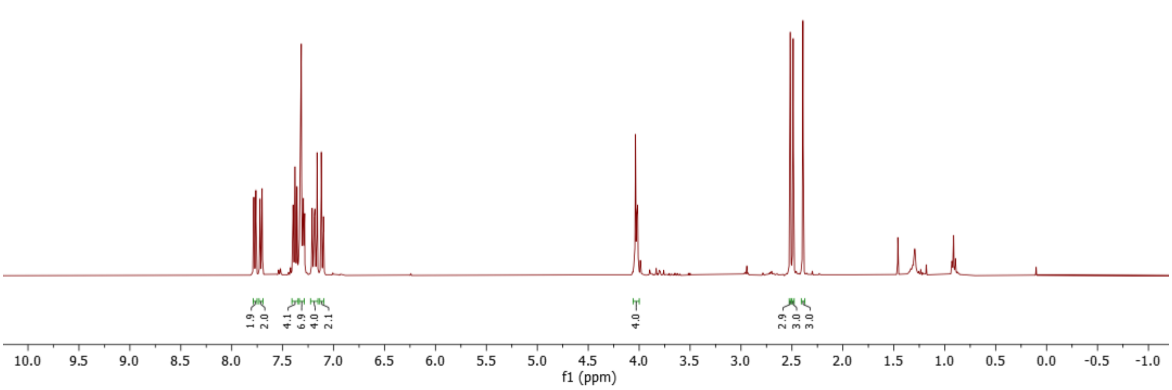

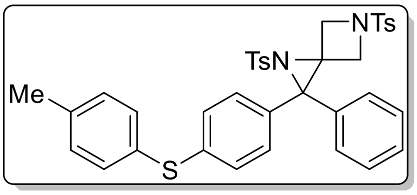

69

^{13}C {^{1}H} NMR (101 MHz, $CDCl_3$)

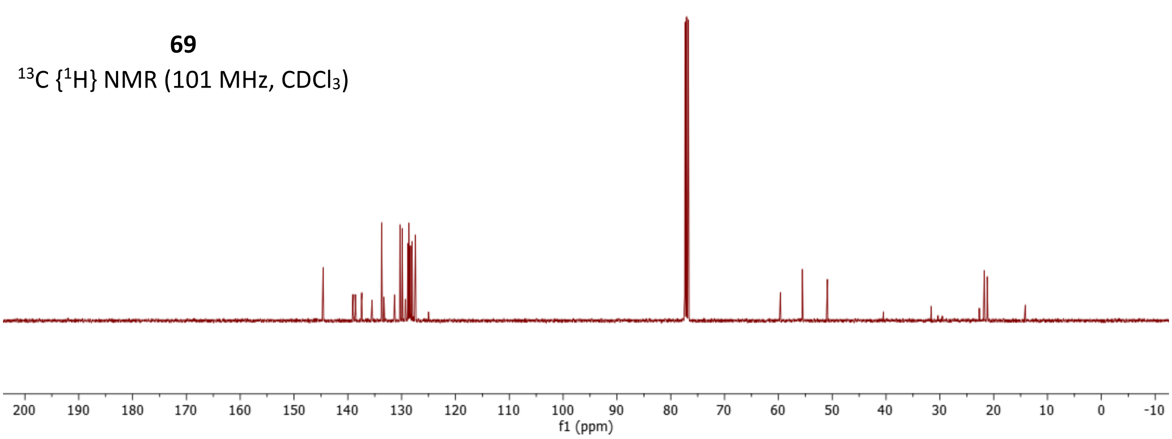

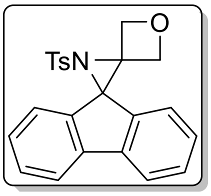

70

^{1}H NMR (400 MHz, $CDCl_3$)

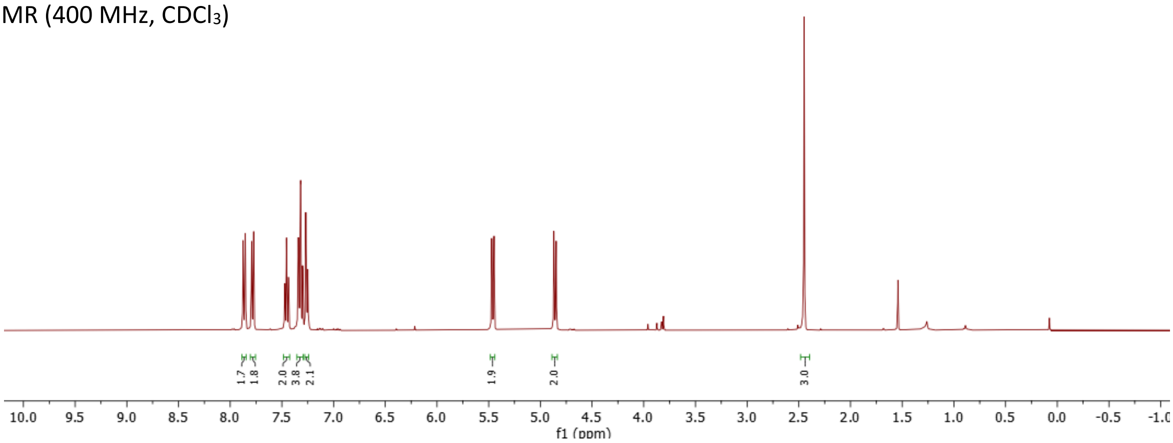

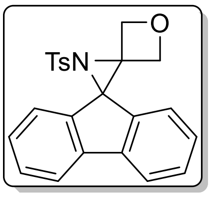

70

^{13}C {^{1}H} NMR (101 MHz, $CDCl_3$)

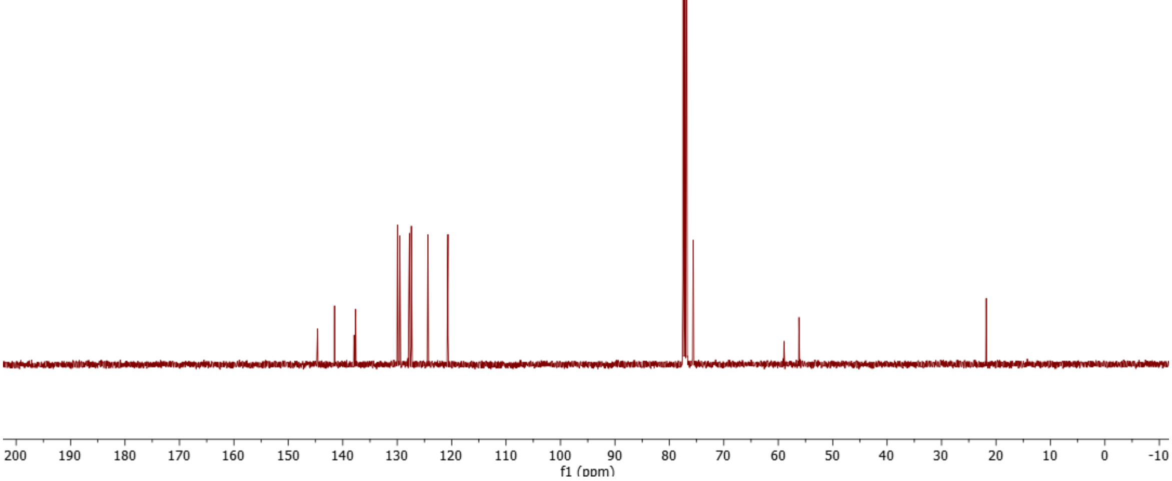

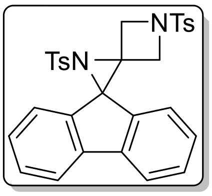

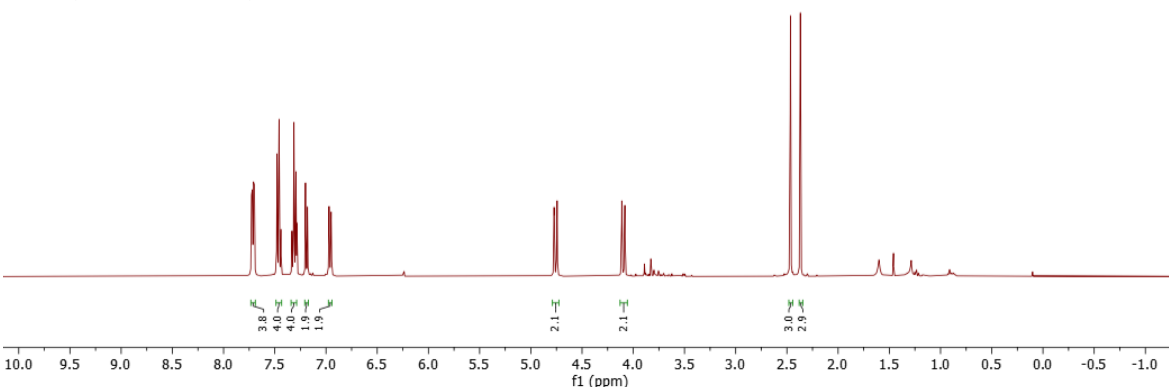

71

^{1}H NMR (400 MHz, $CDCl_3$)

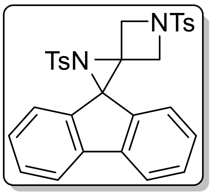

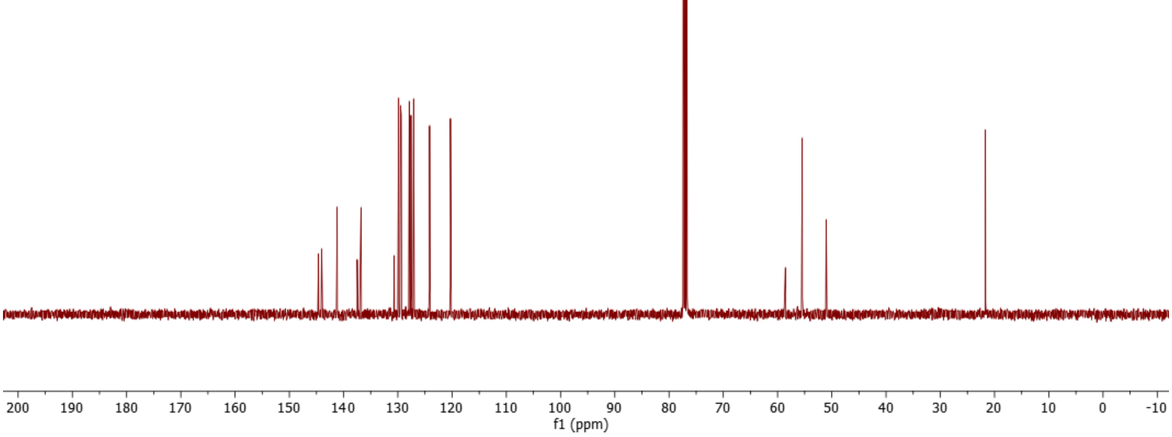

71

^{13}C {^{1}H} NMR (101 MHz, $CDCl_3$)

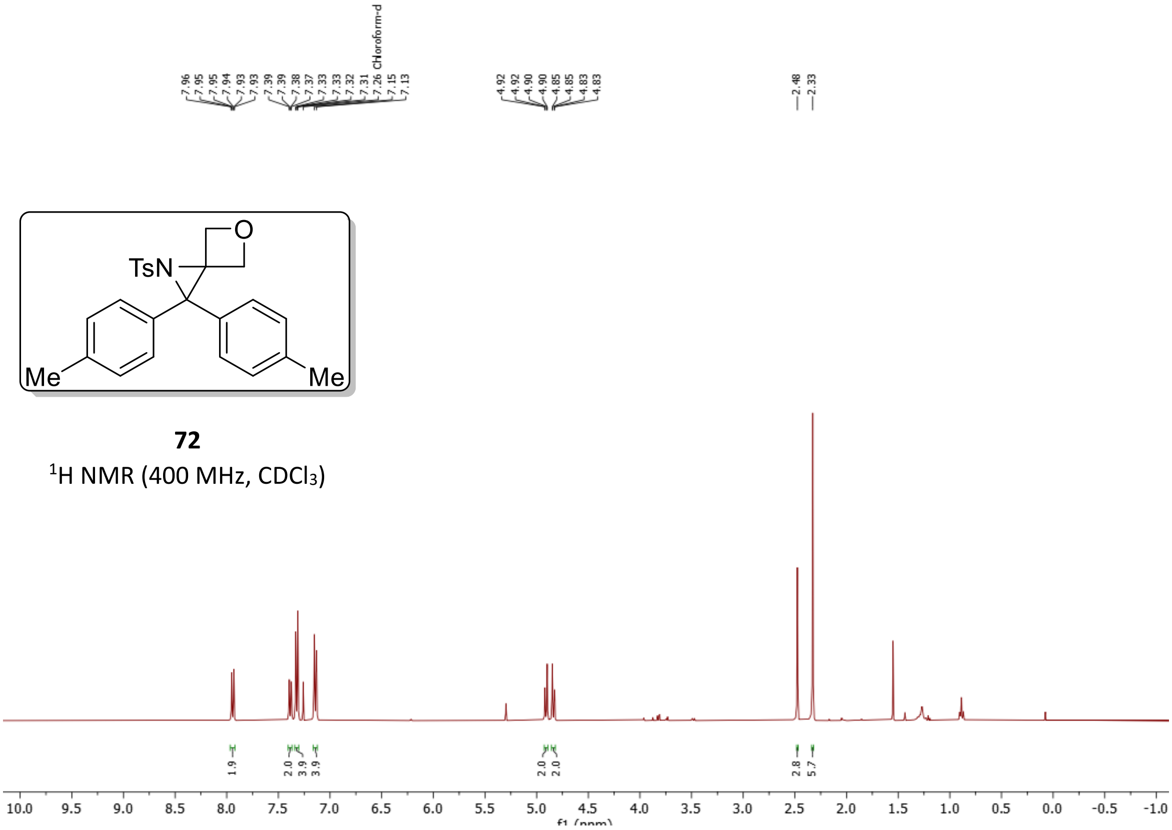

72

^{1}H NMR (400 MHz, $CDCl_3$)

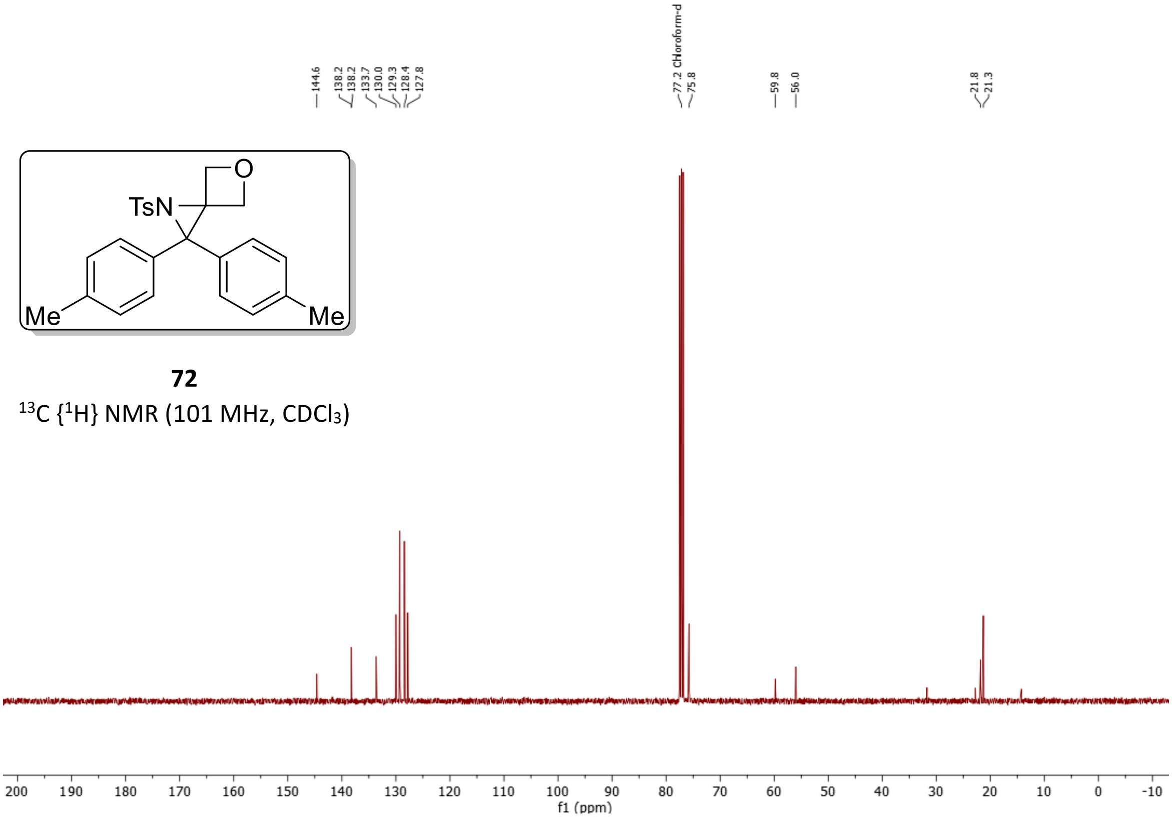

72

$^{13}C\{^{1}H\}$ NMR (101 MHz, $CDCl_3$)

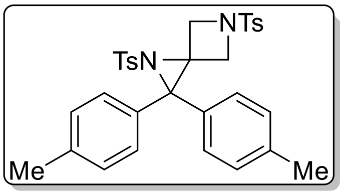

73

^{1}H NMR (400 MHz, $CDCl_3$)

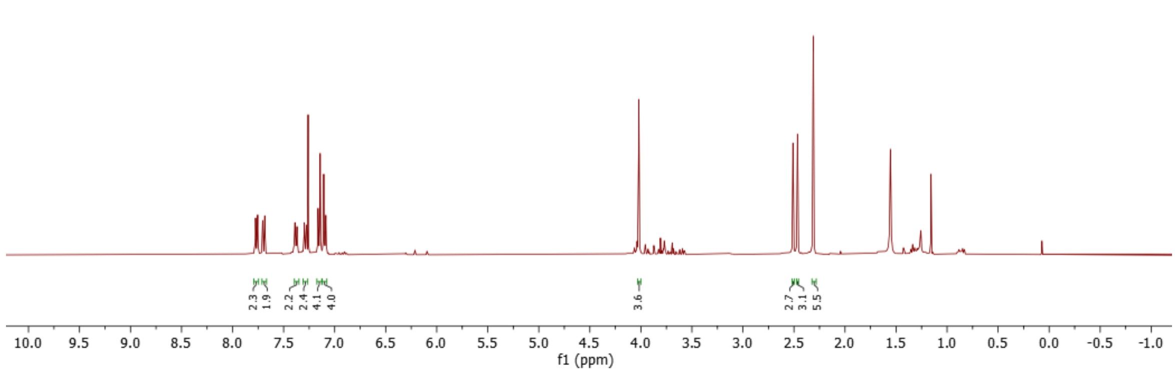

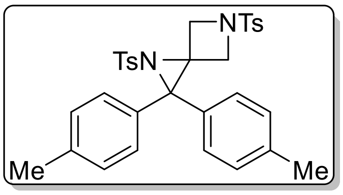

73

^{13}C {^{1}H} NMR (101 MHz, $CDCl_3$)

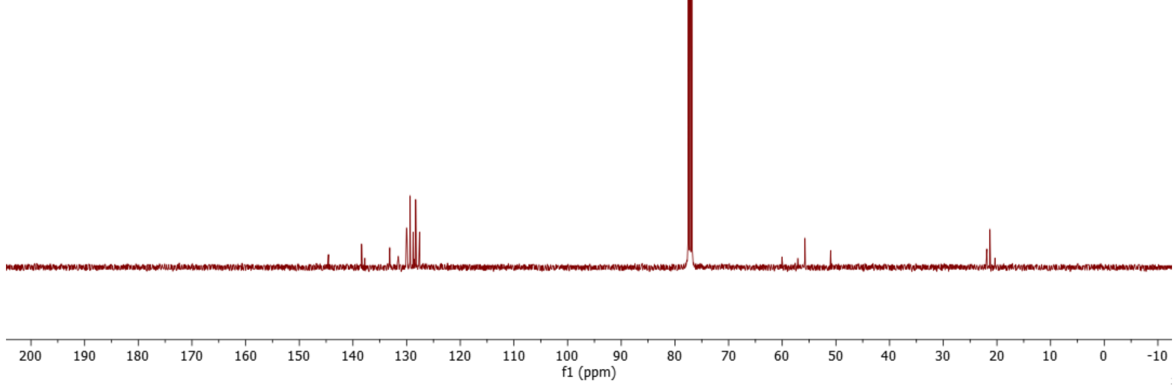

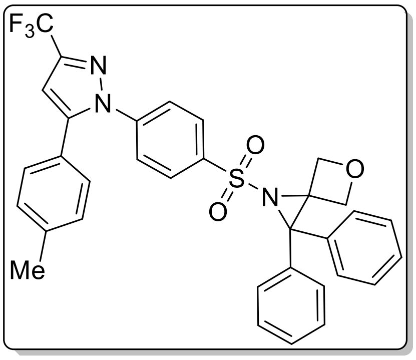

74

^{1}H NMR (400 MHz, $CDCl_3$)

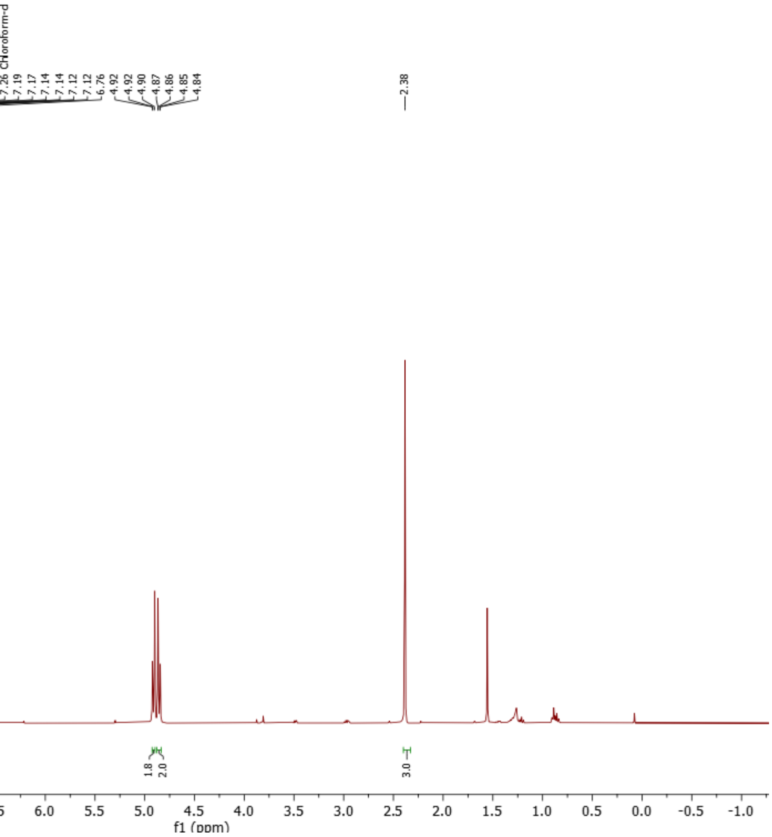

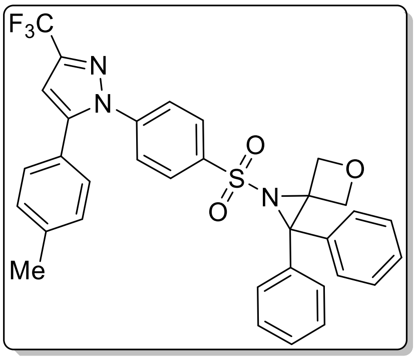

74

^{19}F NMR (377 MHz, $CDCl_3$)

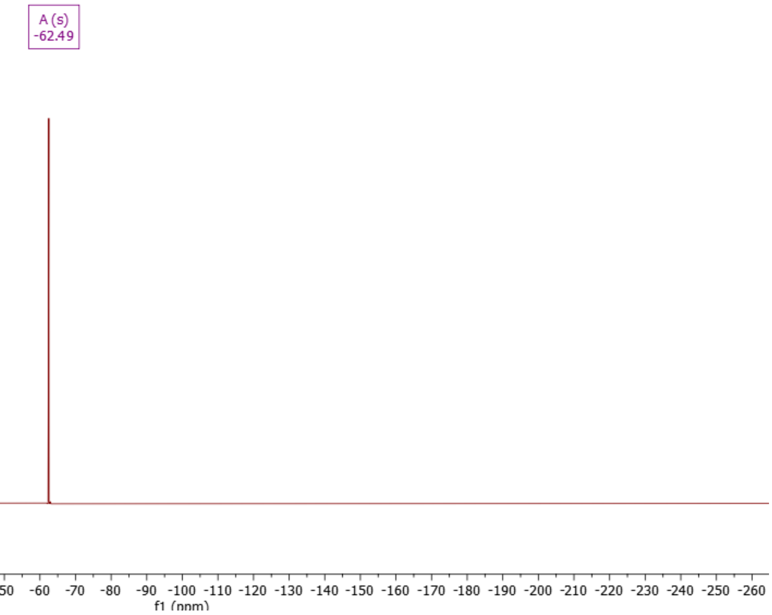

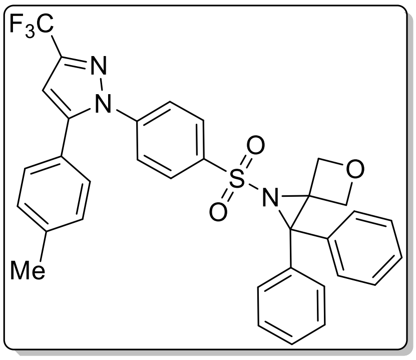

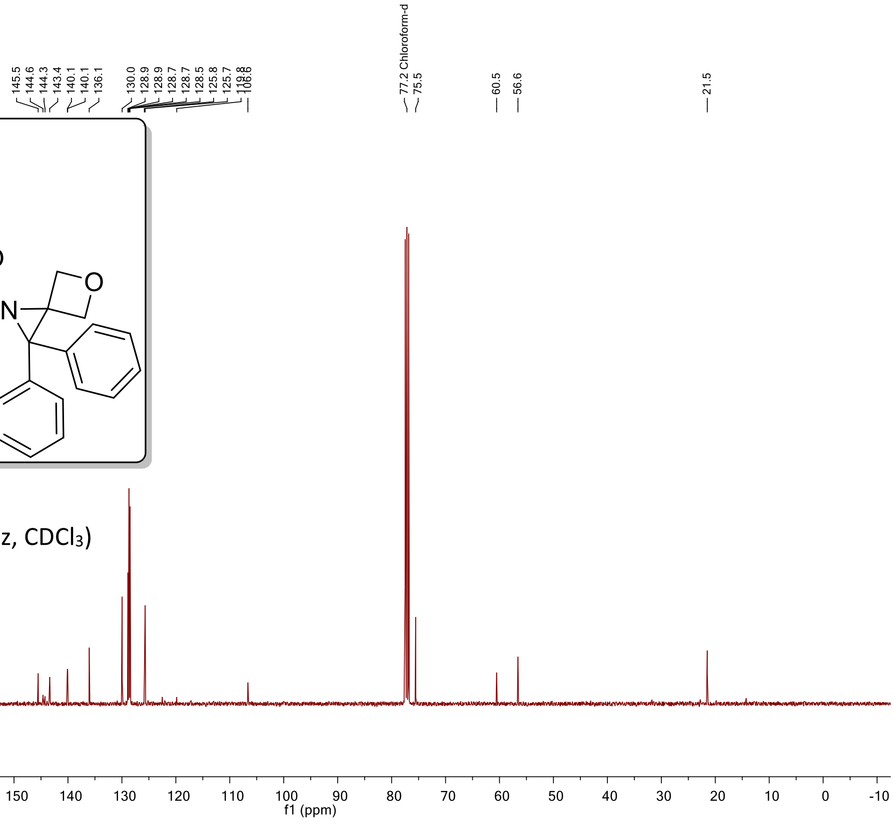

74

$^{13}C\{^1H\}$ NMR (101 MHz, $CDCl_3$)

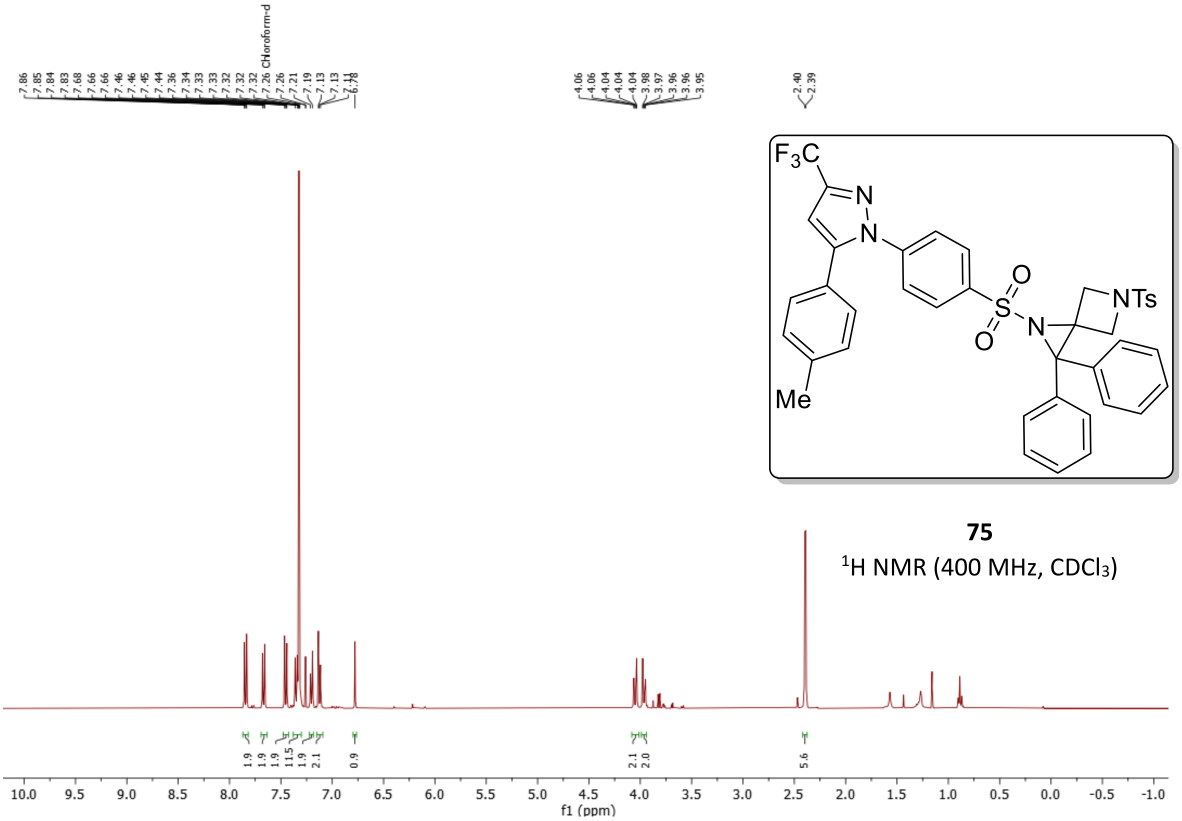

75

^{1}H NMR (400 MHz, $CDCl_3$)

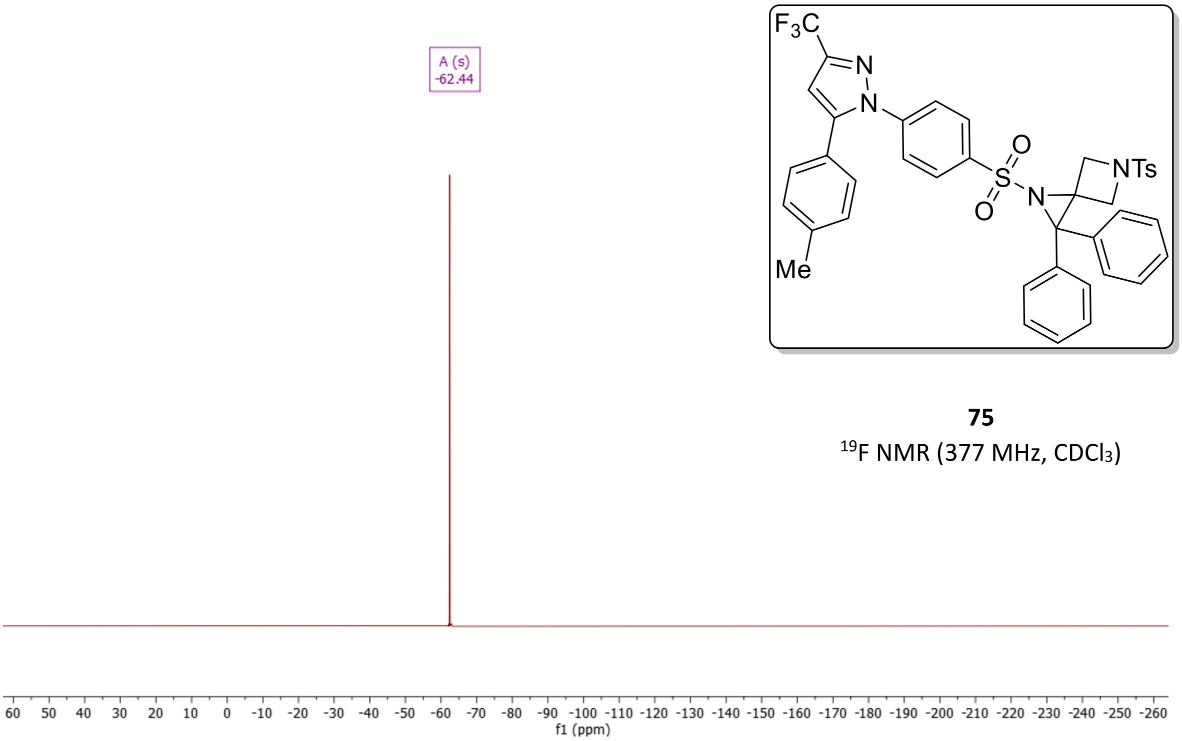

75

^{19}F NMR (377 MHz, $CDCl_3$)

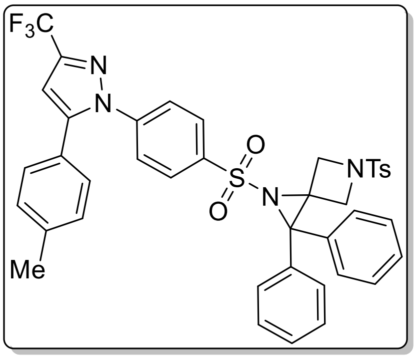

75

$^{13}C\{^{1}H\}$ NMR (101 MHz, $CDCl_3$)

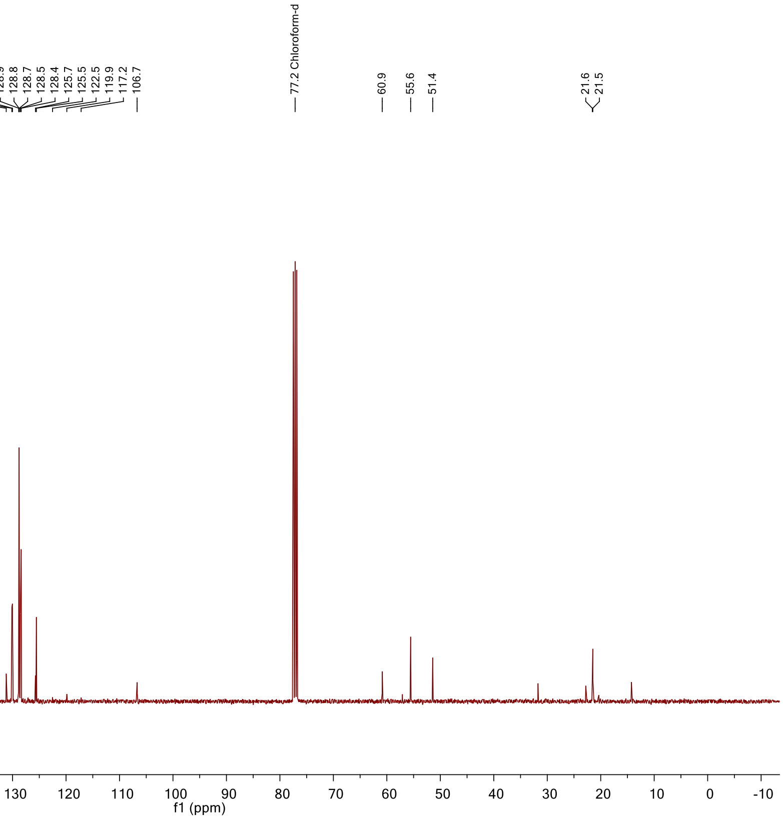

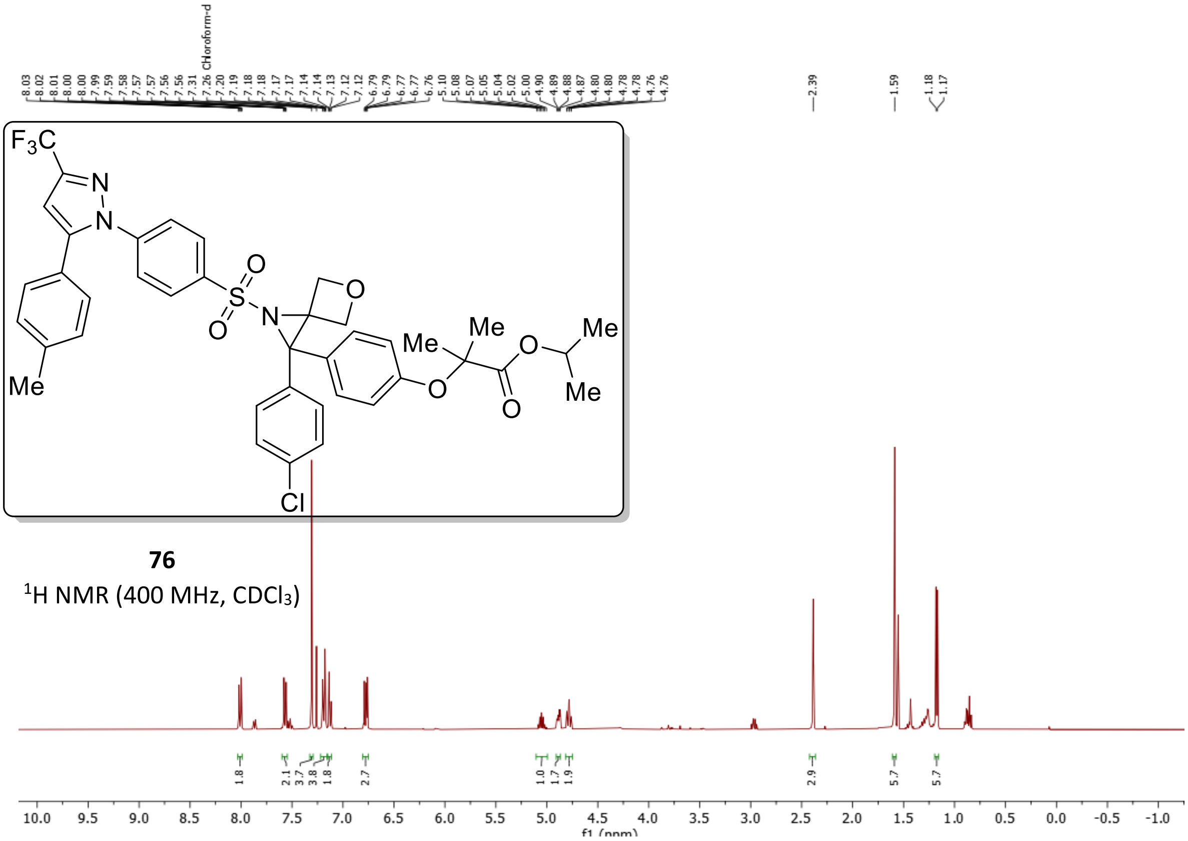

76

^{1}H NMR (400 MHz, $CDCl_3$)

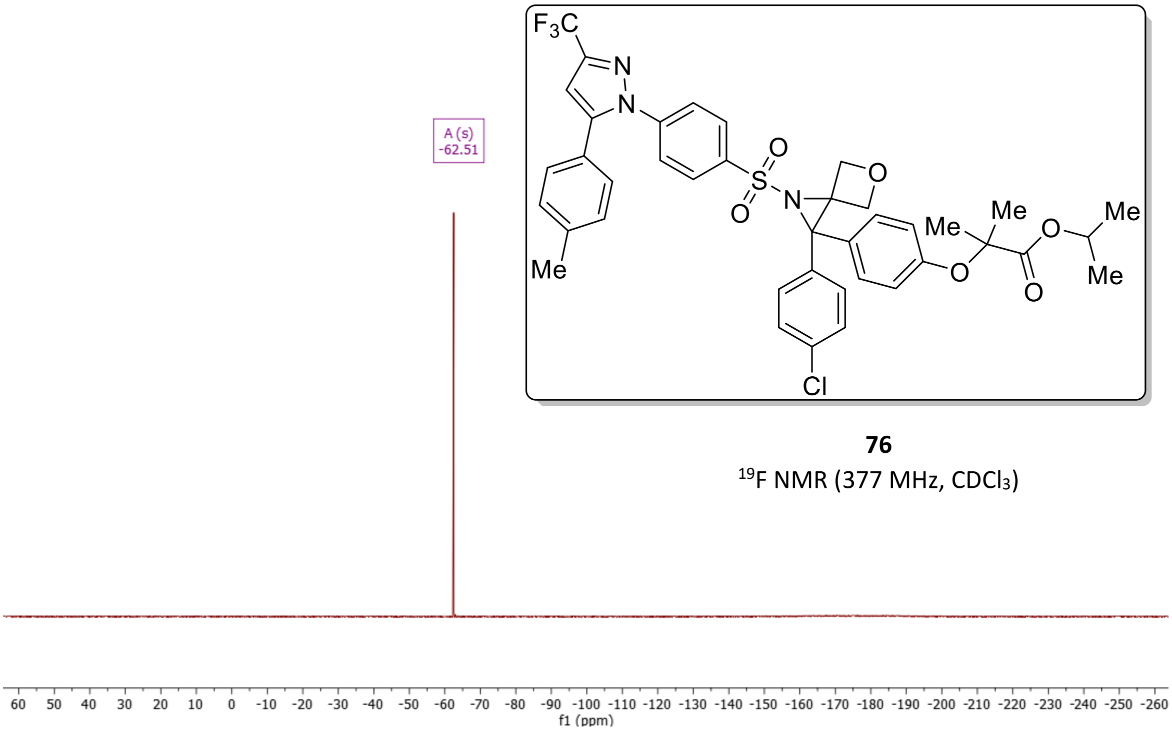

76

^{19}F NMR (377 MHz, $CDCl_3$)

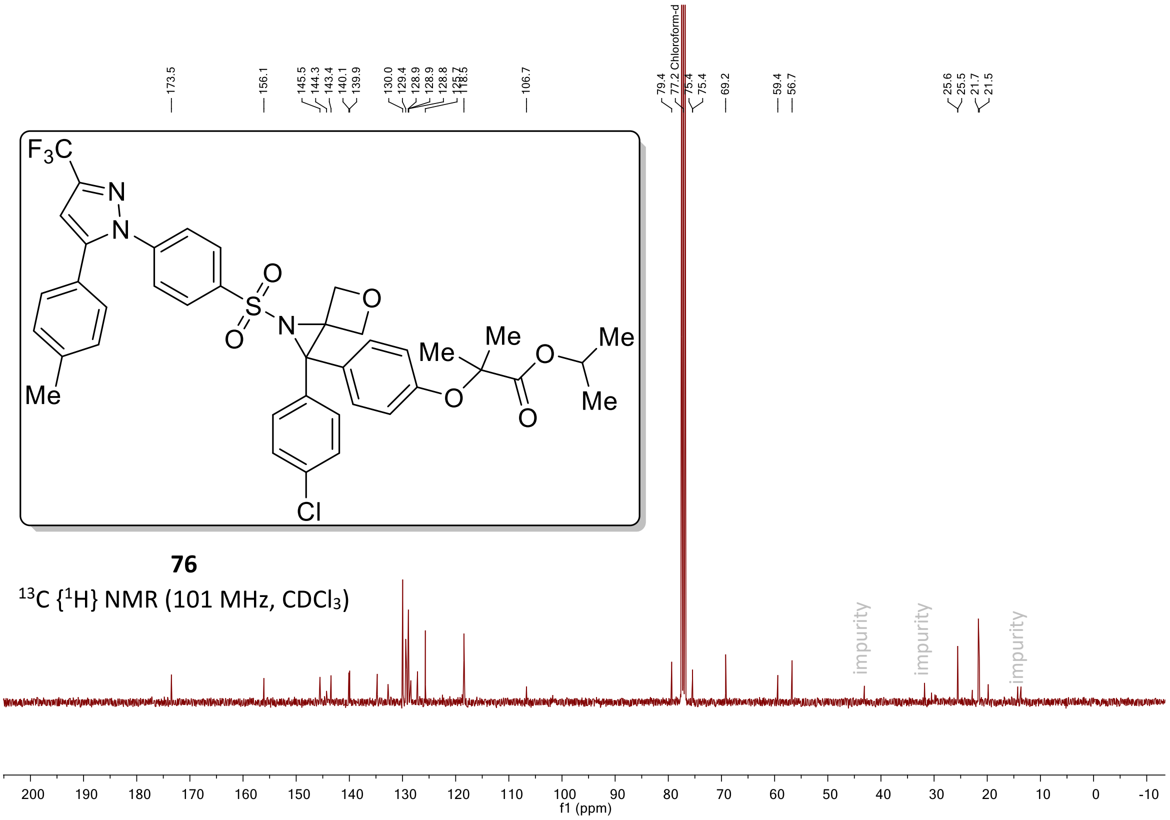

76

$^{13}C\{^{1}H\}$ NMR (101 MHz, $CDCl_3$)

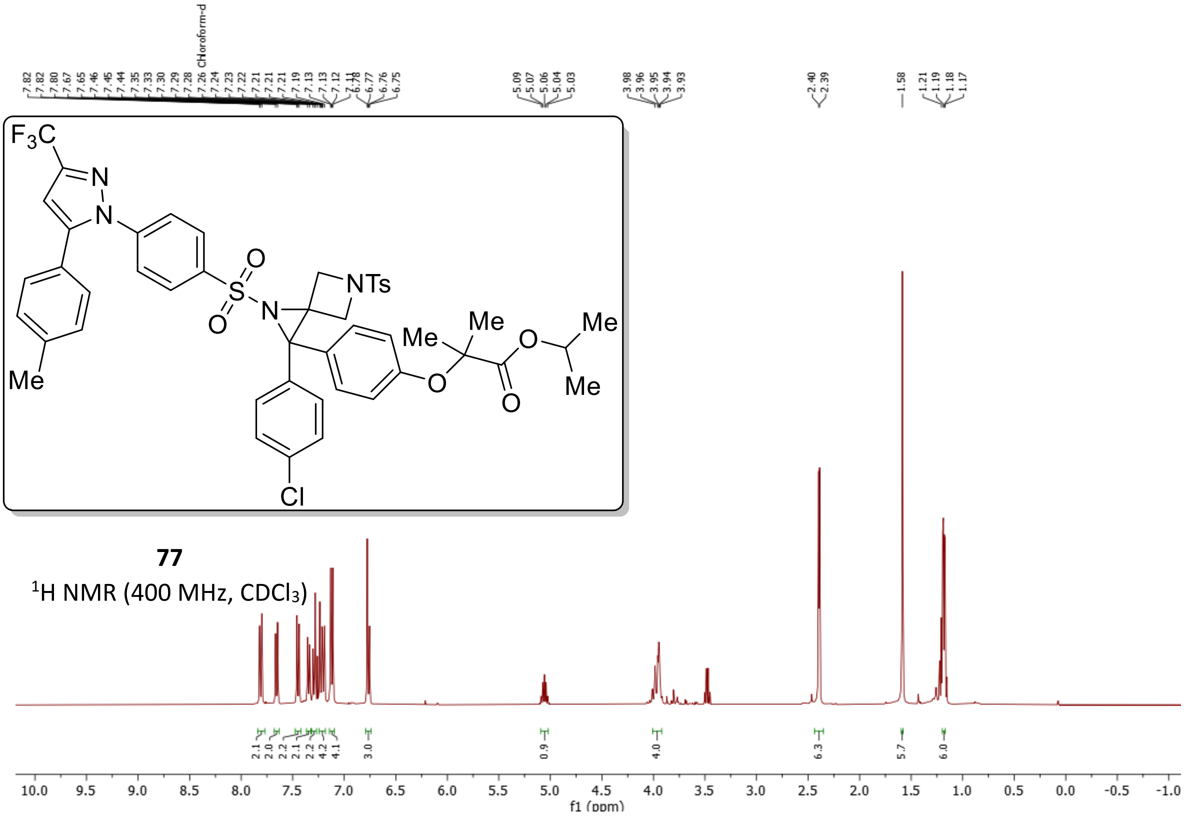

77

^{1}H NMR (400 MHz, $CDCl_3$)

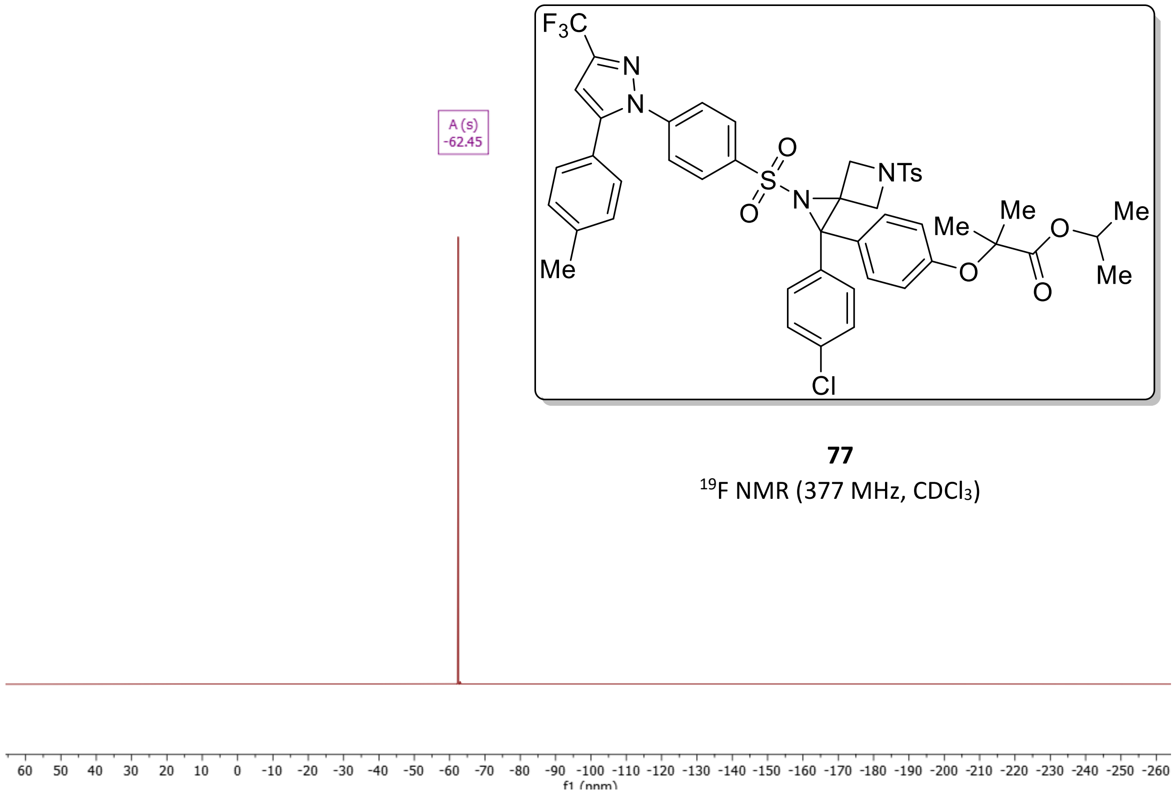

77

^{19}F NMR (377 MHz, $CDCl_3$)

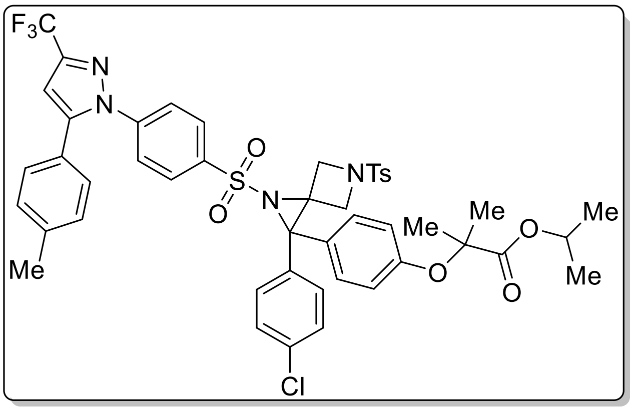

77

$^{13}C\ \{^{1}H\}$ NMR (101 MHz, $CDCl_3$)

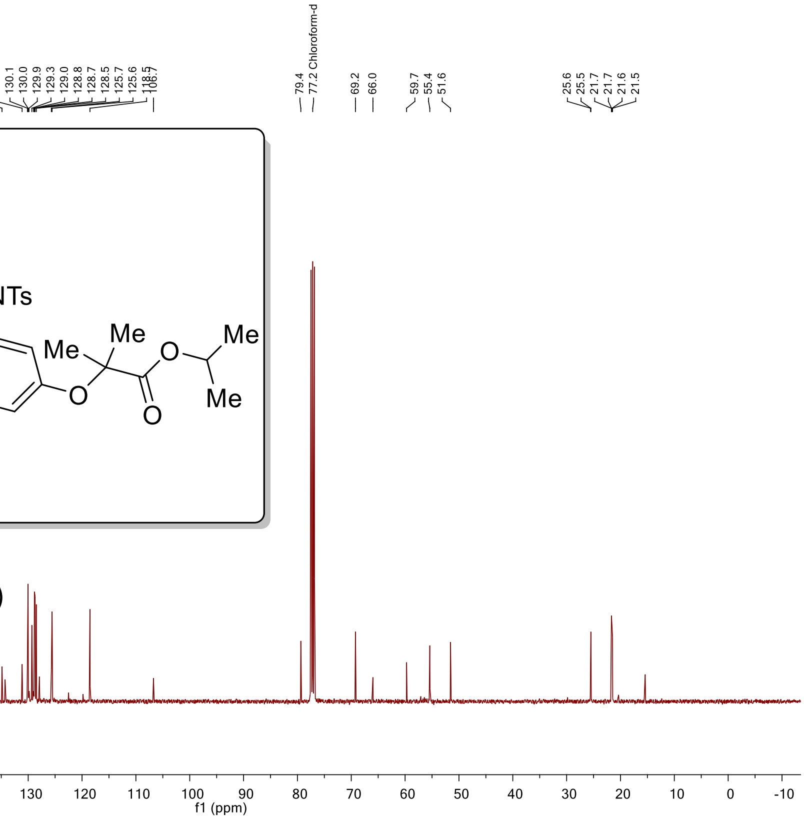

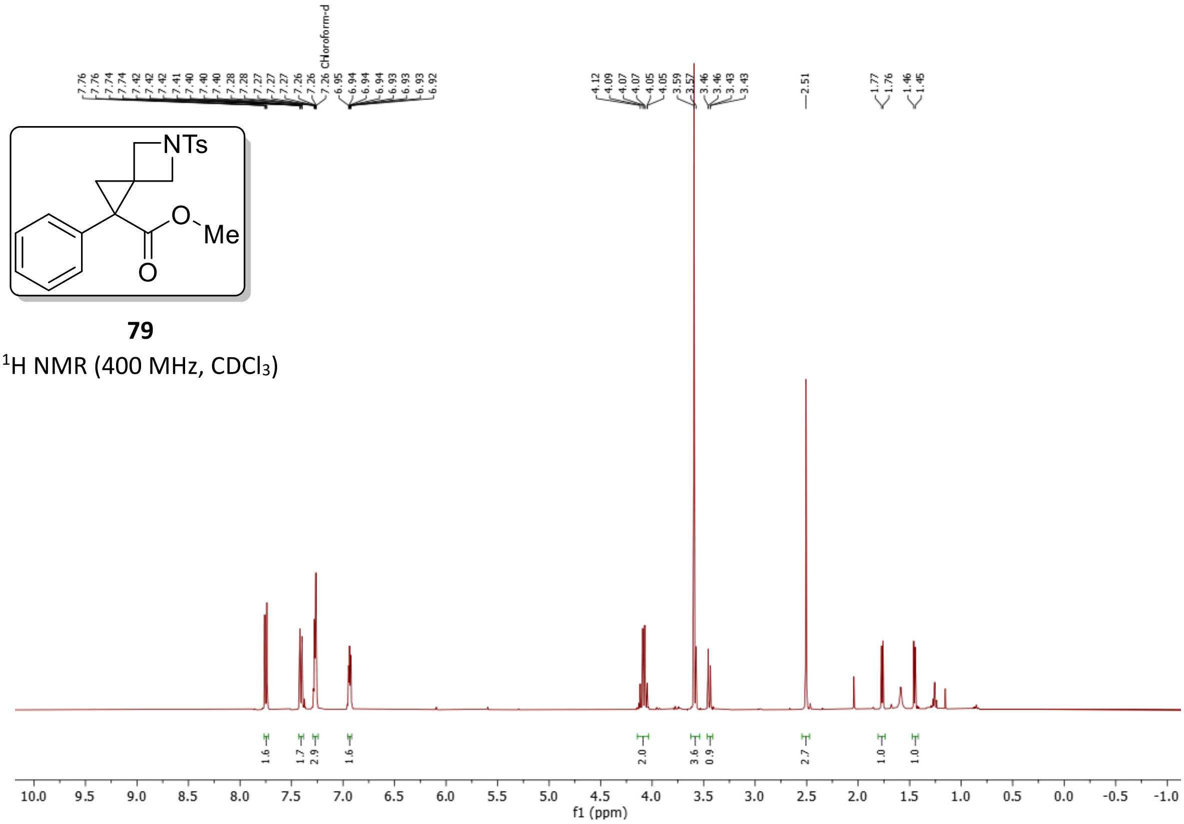

79

^{1}H NMR (400 MHz, $CDCl_3$)

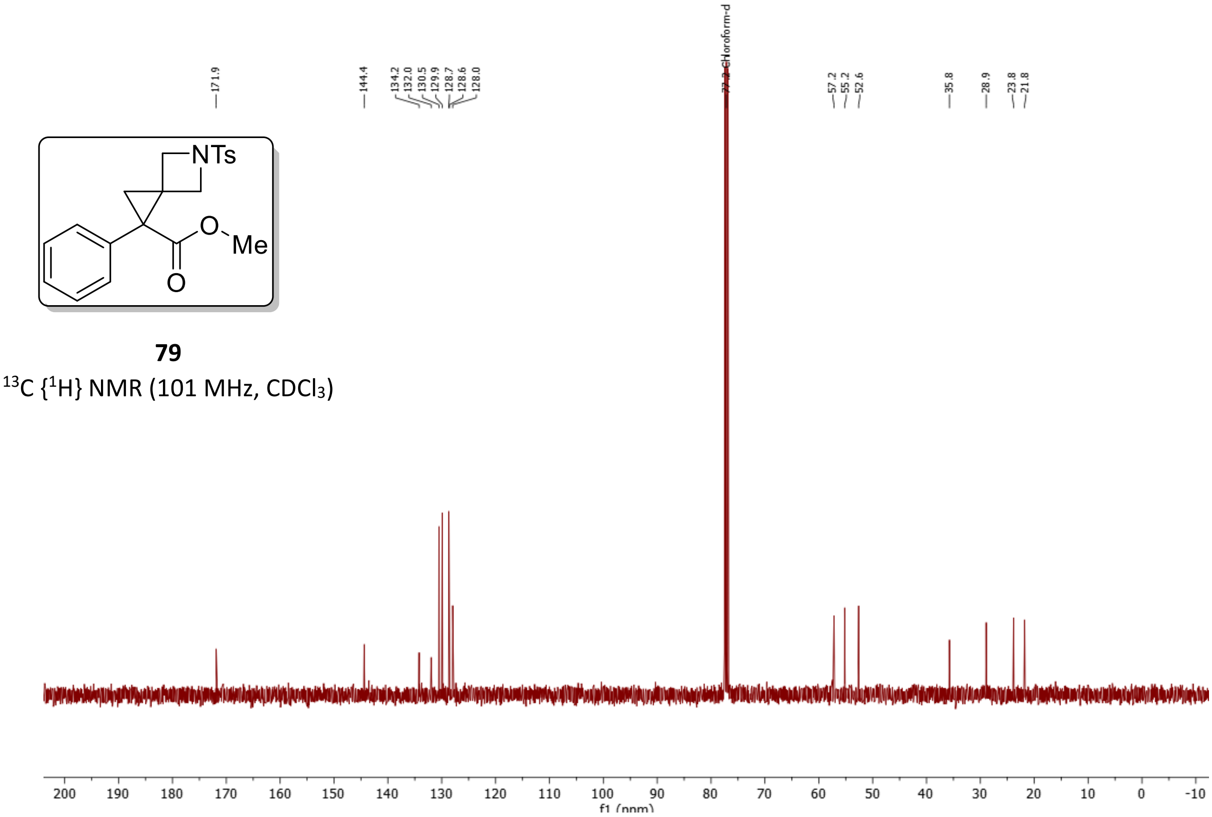

79

^{13}C {^{1}H} NMR (101 MHz, $CDCl_3$)

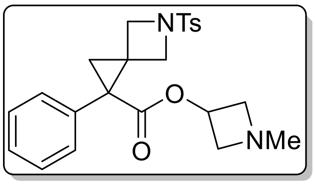

81

^{1}H NMR (400 MHz, $CDCl_3$)

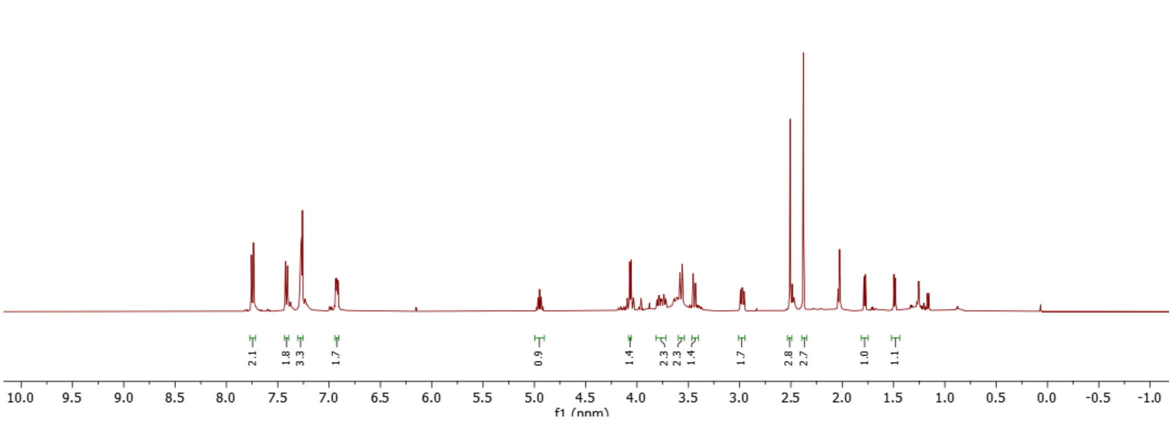

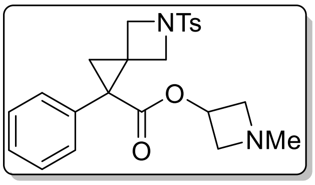

81

^{13}C {^{1}H} NMR (101 MHz, $CDCl_3$)

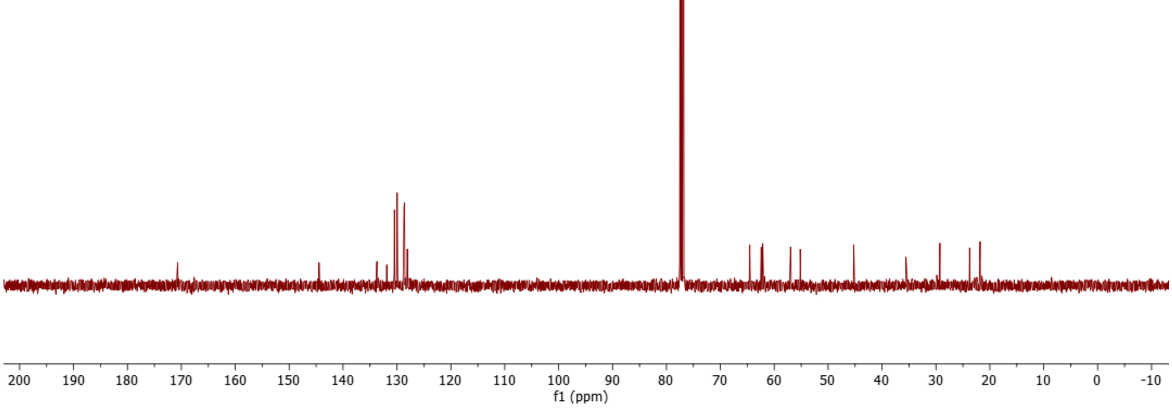

Supplement: Supplementary file 1 — Supporting information [file ANIE-65-e21633-s002.pdf]
